# Stabilizing ruthenium atom layer neighbors through molecular and metal support interactions for water electrolyzers

**DOI:** 10.1038/s41467-026-78037-3

**Published:** 2026-09-22

**Authors:** Seongbeen Kim, Hyunwoo Jun, Jihye Park, Sungjun Kim, Sunghyun Noh, Seung-Jae Shin, Bupmo Kim, Kug-Seung Lee, Jiwon Kim, Jaeho Byeon, Minkyeong Ban, Daeeun Choi, Hoijun Kim, Jong Hyun Jang, Wooyul Kim, Joonhee Moon, Jang Yong Lee, Hyungjun Kim, Jinwoo Lee

**Affiliations:** 1 Department of Chemical and Biomolecular Engineering, Korea Advanced Institute of Science and Technology (KAIST), Daejeon, Korea; 2 Department of Chemistry, Korea Advanced Institute of Science and Technology (KAIST), Daejeon, Korea; 3 Materials Science Division, Lawrence Berkeley National Laboratory, Berkeley, California USA; 4 Hydrogen Energy Research Center, Korea Research Institute of Chemical Technology (KRICT), Daejeon, Korea; 5 Department of Energy Engineering, Korea Institute of Energy Technology (KENTECH), Naju, Korea; 6 School of Energy and Chemical Engineering, Ulsan National Institute of Science and Technology (UNIST), Ulsan, Korea; 7 Pohang Accelerator Laboratory, Pohang University of Science and Technology (POSTECH), Pohang, Korea; 8 Center for Hydrogen and Fuel Cells, Korea Institute of Science and Technology (KIST), Seoul, Korea; 9 Research Center for Materials Analysis, Korea Basic Science Institute (KBSI), Daejeon, Korea; 10 Division of Chemical, Biomolecular and Energy Engineering, Konkuk University, Seoul, Korea

**Keywords:** Electrocatalysis, Molecular self-assembly, Hydrogen energy, Electrocatalysis

## Abstract

Designing catalysts that maximize both atomic efficiency and catalytic activity remains a central challenge in heterogeneous catalysis. While single-atom catalysts maximize noble metal utilization, their isolated structure limits the adsorption configurations of adsorbates, thereby diminishing their catalytic efficiency. We present a versatile platform based on atom-layer neighbors, where the proximity of Ru ensembles diversifies adsorption configurations while simultaneously enhancing adsorbate binding through ligand effects. By leveraging molecular interactions and strong metal-support interactions, we thermodynamically stabilize atomically thin structures comprising only a few Ru atoms, allowing the synthesis of size-tuned Ru atom-layer neighbors on WC_x_ supports. The optimized *H binding on size-tuned Ru ensembles, combined with *OH enrichment on WC_x_ supports, leads to competitive activity per unit noble metal cost in anion-exchange membrane water electrolyzers. Notably, our catalyst achieves the DOE 2026 performance target for PEMWE even at a lower temperature of 60 °C, with a Ru loading of 0.100 mg cm^−2^, below the DOE’s ultimate noble metal loading threshold. This atom-layer neighbor platform offers a general strategy for enhancing catalyst efficiency while maintaining high noble metal utilization.

## Abstract (web summary)

Developing robust, low-noble metal catalysts is essential for practical anion-exchange membrane water electrolysis. Here, the authors use molecular and metal-support interactions to stabilize size-tuned ruthenium atom-layer neighbors, enabling efficient and durable hydrogen production.

## Introduction

**Fig. 1:**
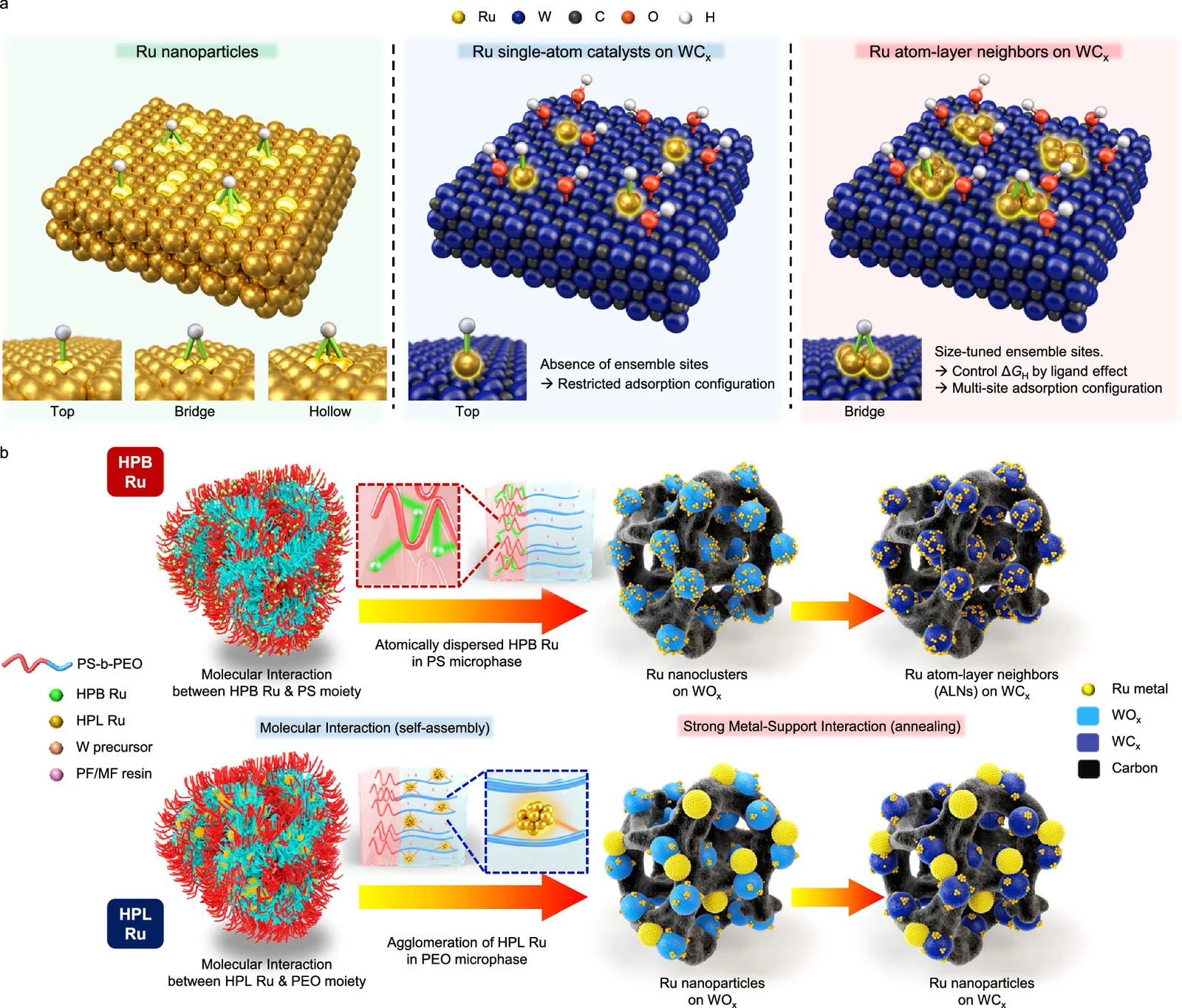
Concept of atom-layer neighbors and synthesis strategy via evaporation-induced self-assembly. **a** Schematic comparison of Ru nanoparticle (NP) catalysts, Ru single-atom catalysts (SACs), and Ru atom-layer neighbors (ALNs). **b** Schematic illustration of the thermodynamic stabilization strategy and the differences arising from varying block copolymer (BCP)-precursor systems. The illustration was created by ScienCG and is reproduced with permission.

Noble metal catalysts are widely used in various catalytic processes due to their distinct catalytic properties. However, their high cost and limited availability significantly increase the expense of industrial applications. To enhance the mass activity (activity per unit mass of noble metal), nanoparticle (NP) catalysts have been extensively developed and employed^1,2^. Similar to their bulk form, noble metal ensembles in NPs offer several strategic advantages for optimizing catalytic activity (Fig. 1a). First, they provide diverse adsorption sites, including top, bridge, and hollow sites, each exhibiting distinct catalytic activity due to variations in adsorbate binding characteristics^3–5^. Second, changes in coordinating metal atoms alter electronic structure (ligand effects), and the coordinating metal atoms themselves can serve as additional active sites for catalysis^6–10^. Despite these benefits, the presence of unexposed metal atoms substantially reduces the efficiency of noble metal utilization, and the sequential structure of NPs limits the selective enrichment of specific adsorption sites, which is essential for increasing catalytic activity.

In this context, single-atom catalysts (SACs) offer an alternative, as their isolated atomic structure maximizes noble metal utilization and provides a uniform adsorption site^11–13^. Owing to their isolated atomic structure, SACs exhibit high mass activity and enhanced selectivity in various catalytic processes^12–16^. However, the absence of ensemble sites restricts further improvement in catalytic activity and limits their broader applicability (Fig. 1a)^17–20^. First, SACs offer only isolated metal sites, which may not provide optimal adsorption configurations for a specific catalytic reaction. Second, strategies to control catalytic activity are largely limited to support modifications, which are challenging due to the high surface energy of noble metal SACs. Furthermore, coordinating anions rarely act as additional active sites in more complex catalysis. Therefore, there is a pressing need for catalyst platforms that feature size-tuned noble metal ensembles for optimal adsorbate binding while incorporating additional active sites near the primary ones, thereby enabling enhanced catalytic activity and durability under practical operating conditions.

Over the past several decades, extensive research on block copolymer (BCP) self-assembly has provided valuable theoretical and experimental insights into a wide array of morphologies^21–24^. By integrating BCP self-assembly with inorganic chemistry, researchers have developed a range of nanostructured inorganic materials, including mesoporous and hierarchically porous materials, as well as shape-controlled mesoporous particles^25–31^. These materials, characterized by their high specific surface area and good pore accessibility, have demonstrated high performance in energy devices, catalysis, biomedical applications, and sensors. A critical factor in self-assembly is the molecular interaction (MI) between the BCP and precursor (MI of [BCP-precursor]), which facilitates the selective incorporation of inorganic precursors into specific BCP microphases^26,32^. These interactions effectively mitigate the MI between precursors (MI of [precursor]), making self-assembly a robust strategy for distributing metal species within or on mesoporous scaffolds^32–35^. However, MIs alone are insufficient for achieving uniform distribution and stabilization of atomic-scale ensembles on the supports. In this regard, strong metal-support interactions (SMSIs) can provide an additional means to stabilize atomic-scale metal species on porous supports. Recent studies, including our previous work on stabilization of noble metal SACs over MoC_x_ supports, have demonstrated that SMSI-stabilized atomic-scale noble metal catalysts can exhibit enhanced activity and stability even under harsh anion-exchange membrane water electrolyzer (AEMWE) single-cell operation conditions^36^. These findings suggest that integrating MIs with SMSIs may provide an effective strategy for constructing highly active and durable noble metal electrocatalysts through stabilization of size-tuned metal ensembles.

AEMWEs have emerged as a promising technology for the sustainable production of green hydrogen, a key resource for operating fuel cells and synthesizing various industrial chemicals and products. Offering comparable cell performance to proton-exchange membrane water electrolyzers (PEMWEs)^36–40^, AEMWEs are more cost-effective owing to their alkaline operating environments, which allow the use of inexpensive cell components and non-noble metal electrocatalysts for the anode, eliminating the dependence on expensive Ir-based electrocatalysts (Supplementary Table 1)^41–43^. However, the sluggish kinetics of the alkaline hydrogen evolution reaction (HER) necessitate high loadings of noble metal (mainly Pt) to achieve competitive performance^38,44,45^. This sluggishness arises from inadequate interfacial water structure, which reduces the hydrogen bond (H-bond) connectivity^46,47^. Studies suggest that *OH species near active sites play significant roles in accelerating reaction kinetics. Specifically, *OH enriches the population of gap water, thereby increasing proton transport through strengthened H-bond networks^46,47^.

Here, we present a platform of Ru atom-layer neighbors (ALNs) on carbide supports for efficient electrocatalysis in AEMWEs (Fig. 1). This platform features size-tuned Ru ensembles with atom-layer thickness, realized by MIs and SMSIs. In alkaline HER, Ru ALNs on WC_x_ with optimized Ru ensemble size showed higher HER activity than commercial Pt/C, primarily because of both the optimized *H binding at the size-tuned Ru ALNs and the strengthened H-bond networks by *OH species on WC_x_. As a cathode electrocatalyst in practical AEMWEs, aRu3/WC_x_ achieved competitive specific activity (activity per unit noble metal cost) among reported AEMWE electrocatalysts and the long-term durability at an industrial current density, even with low noble metal loading of 0.10 mg_Ru_ cm^−2^.

## Results

### Thermodynamic stabilization through molecular and metal–support interactions

The alkaline HER is critical for both industrial applications and as a fundamental model for studying electrochemical reaction mechanisms. The alkaline HER requires two types of active sites in close proximity: one optimized for *H binding and the other for *OH binding^48^. This dual-site requirement makes it an ideal platform for evaluating the design characteristics of noble metal ALNs in electrocatalysis (Fig. 1a). Among noble metals, Ru is particularly well-suited for AEMWEs due to its adequate *H binding energy and high cost efficiency (~40 % of Pt; Supplementary Table 1). Transition metal carbides serve as effective functional supports for efficient alkaline HER. First, their favorable binding of oxygenated species provides sufficient *OH, a key adsorbate in various catalytic mechanisms^13,49^. Second, their strong affinity for noble metal species stabilizes the atomic structure of noble metal during both synthesis and catalytic processes^13,36,50^. Furthermore, their high electrical conductivity and chemical stability enhance the efficiency of electrocatalysis^49^.

**Fig. 2:**
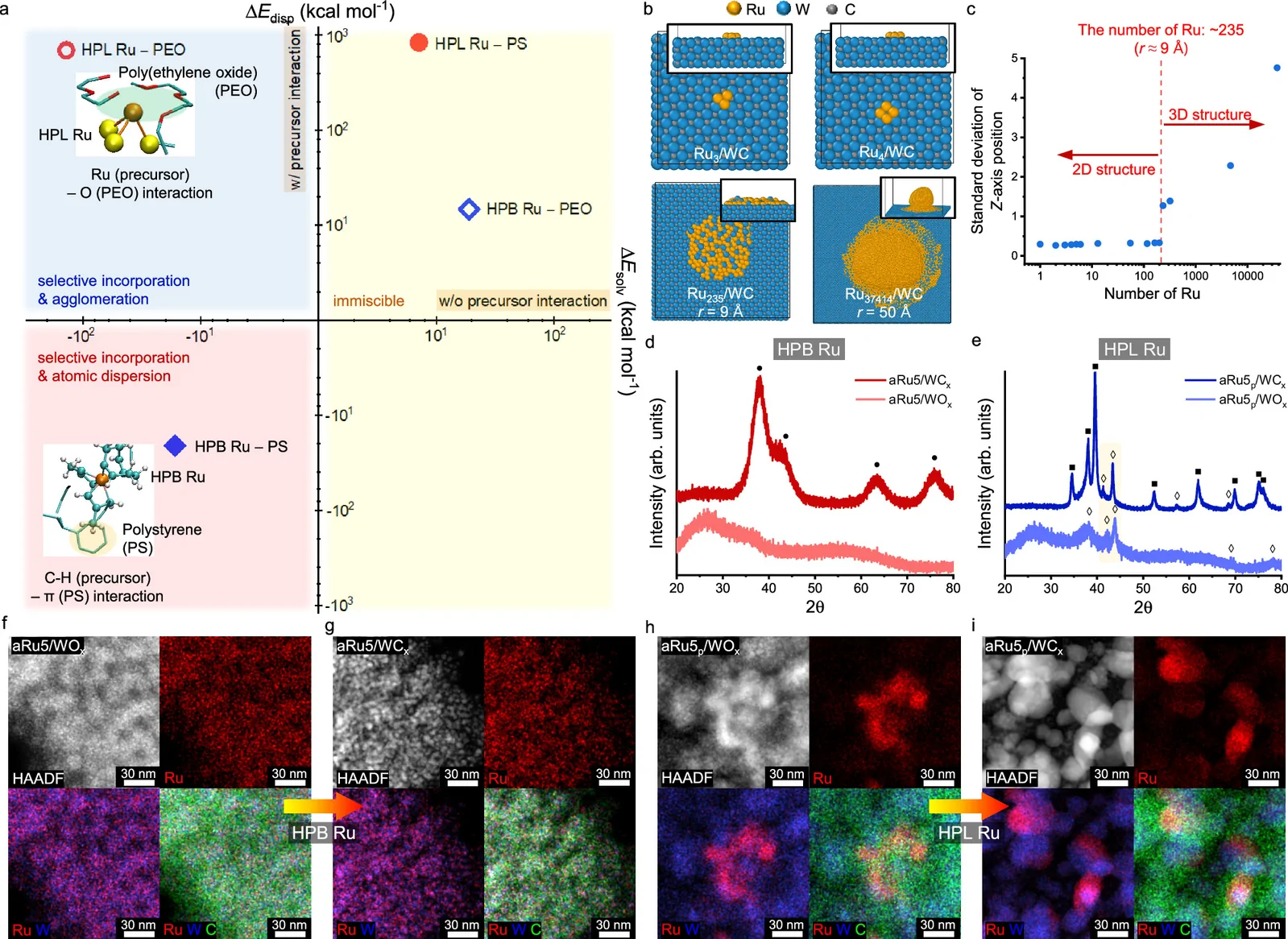
Thermodynamic stabilization strategy using molecular interactions (MIs) and strong metal-support interactions (SMSIs). **a** Relationship between the polymer solvation energy (Δ*E*_solv_) and dispersion propensity (Δ*E*_disp_) of [Ru precursor], as calculated using molecular dynamics (MD) simulations. **b** Top-view snapshots from MD simulations of the equilibrated Ru ensembles on WC (Ru_*N*_/WC) structures at 1200 K (orange: Ru, blue: W, gray: C). The insets show side views of the corresponding Ru_*N*_/WC structures. **c** Standard deviation of *Z*-axis positional distribution of Ru species in Ru_*N*_/WC structures as a function of the number of Ru atoms. XRD patterns of (**d**) samples prepared using hydrophobic Ru precursor (HPB Ru) (aRu5/WO_x_ and aRu5/WC_x_) and (**e**) samples prepared using hydrophilic Ru precursor (HPL Ru) (aRu5_p_/WO_x_ and aRu5_p_/WC_x_) (●: γ-WC [PDF 20-1316], ■: α-W_2_C [PDF 35-0776], ◊: Ru [PDF 88-1734]). The pale-yellow shaded regions highlight the crystalline Ru peaks. High-angle annular dark-field scanning transmission electron microscopy (HAADF-STEM) images and corresponding energy-dispersive spectroscopy (EDS) mappings of (**f**) aRu5/WO_x_, (**g**) aRu5/WC_x_, (**h**) aRu5_p_/WO_x_, and (**i**) aRu5_p_/WC_x_ (red: Ru, blue: W, green: C).

Interaction mediator-assisted evaporation-induced self-assembly (EISA) using the BCP of poly(ethylene oxide)-*block*-poly(styrene) (PEO-*b*-PS) was employed for the synthesis of Ru ALNs on WC_x_^32^. During the EISA, metal precursors were selectively incorporated into specific BCP microphases based on their hydrophilicity. Since a thorough understanding of MIs within EISA systems was essential to achieve atomic dispersion of noble metal precursors, molecular dynamics (MD) simulations were conducted for both hydrophobic Ru precursor (HPB Ru, Bis(2-methylallyl)(1,5-cyclooctadiene)ruthenium(II)) and hydrophilic Ru precursor (HPL Ru, Ruthenium(III) chloride hydrate) to predict the spatial positioning and dispersity of Ru precursors during EISA. The polymer solvation energy (Δ*E*_solv_) of each precursor was calculated to determine its location within the BCP. The calculations revealed that HPL Ru preferentially localizes within PEO microphases due to its favorable Δ*E*_solv_ for PEO (Supplementary Fig. 2, Supplementary Table 2). Conversely, HPB Ru localizes within PS microphases due to its favorable Δ*E*_solv_ for PS (Fig. 2a, Supplementary Figs. 2, Supplementary Table 2), consistent with the like-dissolves-like principle. The dispersion propensity of Ru precursors (Δ*E*_disp_) in the polymer matrix was calculated as the difference between Δ*E*_solv_ and the self-solvation energy (Supplementary Fig. 3, Supplementary Table 3). HPL Ru exhibited unfavorable Δ*E*_disp_ in the PEO matrix due to its strong intermolecular interactions, which can be attributed to its ionic nature. This unfavorable Δ*E*_disp_ predicts its aggregation within the PEO microphase. Conversely, HPB Ru showed favorable Δ*E*_disp_ in the PS matrix, where polymer-precursor interactions exceeded intermolecular interactions, enabling atomic dispersion of HPB Ru in the PS microphase (Fig. 2a). The predicted precursor-polymer interactions were further supported by thermogravimetry analysis with differential scanning calorimetry (TGA-DSC) measurements, which revealed distinct thermal behaviors for the precursor/polymer composites compared with the corresponding individual components (Supplementary Note 2).

To predict the atomic structure of Ru ensembles on WC (Ru_*N*_/WC, *N*: the number of Ru) after high-temperature *T* (high-*T*) annealing at 1173 K, using the number of Ru atoms (*N*_Ru_), MD simulations were performed at 1200 K. For Ru_1_/WC, the high surface energy of Ru SAC caused vigorous thermal migration onto the WC support (Supplementary Fig. 6a, Supplementary Movie 1a). From Ru_2_/WC to Ru_163_/WC, two-dimensional Ru ALNs (one-atom-thick layer) were thermodynamically stabilized by SMSI with WC. In this regime, the proximity of neighboring Ru significantly reduced the mobility of Ru ALNs (Fig. 2b, Supplementary Fig. 6b-j, Supplementary Movies 1–4, 5a). However, when *N*_Ru_ exceeded a critical threshold (*N*_Ru_ ≥ 235), the WC support could no longer stabilize Ru agglomerates as ALNs, resulting in the formation of three-dimensional Ru NPs (Fig. 2b, c, Supplementary Fig. 6k-m, Supplementary Movie 5b-d). By plotting the logarithm of *N*_Ru_ against the Ru-Ru coordination number (CN), we observed a transition in atomic structure from ALNs to sub-monolayer configurations and ultimately to NPs as *N*_Ru_ increased (Fig. 2c, Supplementary Fig. 7, Supplementary Table 6). These results indicated that only Ru_*N*_/WC below a critical size (*N*_Ru_ < 235) can form stable Ru ALNs on WC_x_.

To investigate the effects of BCP-precursor systems and SMSI with WC_x_, aRu*N*/WC_x_ (samples prepared via self-assembly using HPB Ru; N denotes the relative amount of Ru precursor) and aRu5_p_/WC_x_ (sample prepared via self-assembly using HPL Ru with a Ru content similar to that of aRu5/WC_x_) were synthesized. For comparison, Ru on WO_x_ (aRu5/WO_x_ or aRu5_p_/WO_x_) was obtained by annealing at a lower *T* of 873 K to isolate the SMSI effect between Ru and WC_x_ support. Additional comparison samples were prepared using incipient wetness impregnation (IWI) with HPB Ru (Ru/WC_x_) and HPL Ru (Ru_p_/WC_x_), followed by low-*T* reduction at 473 K under 10 v/v% H_2_/Ar (_H_2_) and high-*T* annealing at 1,173 K under Ar (_Ar). Particulate Ru samples on supports were also synthesized by chemical reduction (_R) (see *Catalyst synthesis* in *Methods*). Theoretical predictions indicated that the spatial positioning and dispersity of Ru precursors were governed by MIs within EISA systems. HPB Ru exhibited atomic dispersion in PS microphases, while HPL Ru formed aggregates in PEO microphases (Fig. 1b). During thermal treatment, HPB Ru reduced to sub-nanoclusters, which subsequently stabilized as ALNs via SMSI with WC_x_ due to their sufficiently small *N*_Ru_ (below the critical threshold). Furthermore, the limited mobility of Ru ensembles on WC_x_ allowed for systematic tuning of ensemble size by adjusting the Ru precursor amount (Supplementary Notes 1-3). In contrast, large agglomerates of HPL Ru formed large Ru NPs, unable to stabilize as ALNs due to their large *N*_Ru_.

To clarify the effect of MIs, Ru content in comparison samples was set to ~10 wt%, matching the Ru content in aRu5/WC_x_ (Supplementary Table 7). Samples synthesized via self-assembly exhibited mesoporous structures with three-dimensional pore networks and large mesopores, as confirmed by N_2_ physisorption, small-angle X-ray scattering (SAXS), and scanning electron microscopy (SEM) (Supplementary Figs. 8–20). The relative volume fractions of hydrophilic and hydrophobic microphases are critical determinants of mesoporous morphology, indicating that porosity depends not only on the precursor amount but also on its hydrophilicity^22^. Consequently, even with the same molar amounts of Ru precursor, samples prepared with HPB Ru exhibited distinct porosity compared to those prepared with HPL Ru (Supplementary Fig. 20). Consistent with previous reports, these results suggest that the chemical affinity of the Ru precursors governs their preferential interactions with specific BCP microphases during the EISA process, thereby influencing mesostructural correlations, structural ordering and porosity evolution (Supplementary Note 5)^51–53^.

X-ray diffraction (XRD) analysis revealed the presence of crystalline WC_x_ in all samples, except for aRu5/WO_x_ and aRu5_p_/WO_x_ (Fig. 2d, e, Supplementary Fig. 21, Supplementary Note 4). Notably, no crystalline Ru peaks were observed in either aRu5/WO_x_ or aRu5/WC_x_, whereas aRu5_p_/WO_x_ and aRu5_p_/WC_x_ exhibited clear crystalline Ru peaks. This suggested that the MI of [PS-HPB Ru] effectively mitigated the MI of [HPB Ru], thereby facilitating the stabilization of Ru species as Ru ALNs (Supplementary Note 4). High-angle annular dark-field scanning transmission electron microscopy (HAADF-STEM) with energy dispersive spectroscopy (EDS) mapping of aRu5/WO_x_ showed uniform Ru distribution without large agglomerates (Fig. 2f, Supplementary Fig. 22), while aRu5_p_/WO_x_ exhibited uneven Ru dispersion with large agglomerates (Fig. 2h, Supplementary Fig. 24). These results showed that selecting an appropriate BCP-precursor system was crucial. This choice was particularly important for achieving uniform atomic dispersion of Ru species, and it required a detailed understanding of MIs within EISA.

STEM-EDS of aRu5/WC_x_ revealed the uniform and selective dispersion of Ru ALNs on WC_x_ supports, showing that the SMSI with WC_x_ effectively prevented thermal sintering of Ru into NPs by maintaining Ru as ALNs (Fig. 2g, Supplementary Fig. 23). In comparison, aRu5_p_/WC_x_ showed large Ru agglomerates, indicating that the SMSI with WC_x_ was insufficient to stabilize large Ru agglomerates (exceeding the critical size threshold) as ALNs (Fig. 2i, Supplementary Fig. 25). Likewise, aRu*N*/WC_x_, synthesized via thermodynamic stabilization, exhibited the same features as aRu5/WC_x_ in STEM-EDS, demonstrating the versatility of thermodynamic stabilization strategy for a wide range of Ru contents (~10 wt%) (Supplementary Figs. 26–29). Furthermore, the Ru atomic ratio Ru/(Ru+W) obtained from EDS analysis was in close agreement with the X-ray fluorescence (XRF) results, indicating that Ru is uniformly distributed throughout the sample via MIs, leading to consistent values between bulk (XRF) and local (EDS) analyses (Supplementary Figs. 22–30, Supplementary Table 7). In contrast, XRD and STEM-EDS of IWI-prepared samples revealed that IWI alone was insufficient to stabilize Ru ALNs, as it lacked mechanisms to mitigate MI of [Ru precursor], resulting in the formation of large agglomerates before annealing (Supplementary Figs. 19, 29–32, Supplementary Notes 4, 6).

Z-contrast imaging using high-resolution HAADF-STEM is widely applied to elucidate the atomic structure of noble metal on supports, as the brightness scales with both the atomic number (*Z*) and the number of atoms overlapped along the electron-beam direction^54,55^. However, in the case of Ru ALNs on WC_x_, tungsten (*Z* = 74) possesses a much higher *Z* than ruthenium (*Z* = 44), resulting in stronger intensity and making it difficult to distinguish Ru from the underlying W in thick WC_x_ supports (>~2 nm). The simulated Z-contrast images for a single Ru atom and a single W defect on γ-WC ( ~ 0.6 nm thickness) exhibited similarly dark features (Supplementary Fig. 37a, b), indicating that such contrast cannot discriminate whether the dark spot originates from Ru or from a W vacancy. Furthermore, simulated Z-contrast images of Ru_*N*_ (*N* = 1-6) on γ-WC (~2 nm thickness) show nearly indistinguishable intensity profiles (Supplementary Fig. 38), suggesting that HAADF-STEM Z-contrast imaging is not suitable for resolving Ru ALN configurations on WC_x_ supports.

Consistent with these simulations, experimental Z-contrast images of both aRu0/WC_x_ (without Ru) and aRu3/WC_x_ exhibited similar distributions of bright (red circles) and dark spots (Supplementary Fig. 37c, d), confirming the intrinsic difficulty in distinguishing atomically dispersed Ru from local contrast variations within the WC_x_ lattice. In contrast, when Ru forms three-dimensional nanoclusters exceeding a critical size, the simulated image of Ru_235_/WC reveals distinguishable Ru features on the γ-WC lattice (Supplementary Fig. 39a) due to the increased Ru thickness and cumulative scattering along the beam path^55^. Similarly, experimental Z-contrast images of Ru/WC_x__R (Ru nanoclusters on WC_x_ supports, see *Catalyst Synthesis* in *Methods* and Supplementary Note 6) resolve sub-nanometer Ru aggregates (red circles, <1 nm) on WC_x_ supports (Supplementary Fig. 39b). These results collectively indicate that HAADF-STEM Z-contrast imaging can reliably identify three-dimensional Ru nanoclusters, but cannot distinguish two-dimensional Ru ALN configurations on heavy WC_x_ supports.

According to the Z-contrast image of aRu3/WC_x_, no three-dimensional sub-nanometer Ru aggregates were verified (Supplementary Fig. 37c). Electron energy-loss spectroscopy (EELS) line scans using atomic-resolution STEM across the Ru L-edge revealed a spatially compact grouping of Ru signals, typically consisting of two to three adjacent atomic-scale peaks, together with a cumulative increase in the integrated areal density along the scan path (Supplementary Fig. 40). This spatially correlated Ru signal evolution supports the presence of Ru ensemble sites on the WC_x_ supports rather than isolated single atoms or three-dimensional aggregates.

### Geometric and electronic structure of Ru atom-layer neighbors

**Fig. 3:**
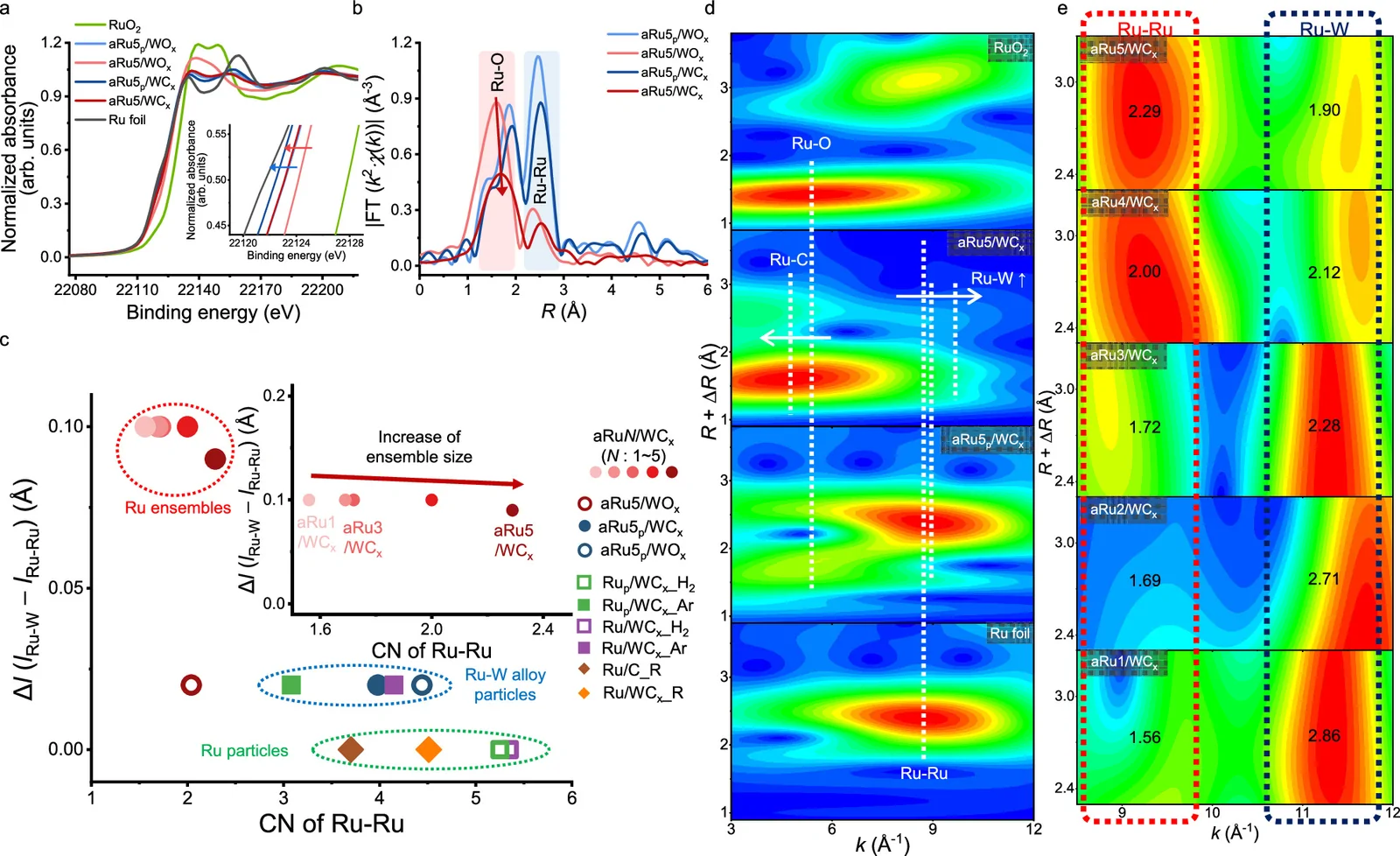
Alteration of the geometric and electronic structure of Ru ALNs. **a** X-ray absorption near-edge structure (XANES) spectra at the Ru K-edge (Inset: enlarged view of the selected region of the main panel). **b** *k*^2^-weighted extended X-ray absorption fine structure (EXAFS) Fourier transform magnitude in *R* space (*χ*(*R*)) at the Ru K-edge of samples prepared using hydrophilic Ru precursor (HPL Ru) or hydrophobic Ru precursor (HPB Ru) with different annealing *T* (red shaded region: Ru-O interactions, blue shaded region: Ru-Ru interactions). *k* denotes the photoelectron wavenumber, and *R* denotes the radial distance in Fourier-transformed EXAFS *R* space. **c** Difference between the fitted Ru-W and Ru-Ru interatomic distances as a function of the coordination number (CN) of Ru-Ru interactions, based on EXAFS fitting results for the second shell at the Ru K-edge. Δ*l* is defined as *l*_Ru-W_ – *l*_Ru-Ru_, where *l*_Ru-W_ and *l*_Ru-Ru_ are Ru-W and Ru-Ru interatomic distances, respectively (Inset: enlarged view of the selected region of the main panel). **d** Overview wavelet transforms of aRu5/WC_x_ and aRu5_p_/WC_x_ at the Ru K-edge. **e** Detailed wavelet transform analyses of aRu*N*/WC_x_ at the Ru K-edge second-shell region (red region: Ru-Ru interactions, blue region: Ru-W interactions). In d and e, *R* + Δ*R* denotes the radial coordinate used in the wavelet-transform maps. The inserted numbers indicate the CNs obtained from EXAFS fitting.

To investigate the electronic and geometric structures of Ru ALNs, X-ray photoelectron spectroscopy (XPS) and X-ray absorption spectroscopy (XAS) were performed. During the transition from aRu5/WO_x_ to aRu5/WC_x_, XPS showed a negative shift in Ru 3 *d* peaks (Supplementary Fig. 41a). Likewise, X-ray absorption near-edge structure (XANES) analysis at the Ru K-edge revealed a negative shift in the edge position for aRu5/WC_x_ compared to aRu5/WO_x_ (Fig. 3a). The edges of aRu*N*/WC_x_ aligned closely to that of metallic Ru (Supplementary Fig. 58a). These indicated that the SMSI with WC_x_ imparted metallic properties to Ru ALNs, which is distinct from those of comparison samples and previous findings of Ru SACs on various supports (Supplementary Figs. 41–58, Supplementary Note 7) ^39,56,57^.

The Fourier transforms of extended X-ray absorption fine structure (FT-EXAFS) with corresponding fitting results reveal that aRu*N*/WC_x_ was coordinated by ~4 Ru-C bonds (~0.1 Å longer than Ru-O) and ~2 Ru-W bonds, indicating that Ru ALNs were selectively stabilized on WC_x_ supports (Fig. 3b, c, Supplementary Figs. 59, 67, Supplementary Tables 8, 9). In contrast, aRu5_p_/WC_x_ and IWI-prepared samples exhibited ~2 Ru-O coordination in the first shell, which originated from surface oxygen bonds on Ru agglomerates^58^. Furthermore, the CN of Ru-Ru in aRu*N*/WC_x_ gradually increased with higher Ru contents, suggesting that the Ru precursor amount systematically controlled the ensemble size of Ru ALNs (Fig. 3c, Supplementary Fig. 67, Supplementary Table 9). The Ru-Ru CN for aRu*N*/WC_x_ ranged between 1.5 and 2.5, with distinct bond lengths of Ru-Ru (~2.74 Å) and Ru-W (~2.84 Å), which differed from those for Ru metal and Ru agglomerates in comparison samples (Fig. 3c, Supplementary Tables 8–11). The low Ru-Ru CN suggests that each Ru ALN consisted of six or fewer Ru atoms, and the distinct bond lengths likely result from two-dimensional atomic structures of Ru ALNs via SMSI with WC_x_ supports (Supplementary Table 6, Supplementary Note 8) ^59^. The FT-EXAFS with corresponding fitting for aRu5_p_/WC_x_ and IWI-prepared samples revealed the presence of large Ru or Ru-W alloy agglomerates (Fig. 3b, c, Supplementary Tables 8–10, Supplementary Note 8).

Because HAADF-STEM analyses provide highly local structural information, they cannot alone verify whether the Ru ALN configuration represents the dominant structure across the catalyst. To address this, we compared the structural parameters predicted by theory with those obtained from bulk-averaged EXAFS fitting. Because the DFT models represent idealized local structures and do not fully capture the heterogeneity and dynamic electrochemical environment of the catalyst under operating conditions, the calculations are used mainly to support trends and guide the interpretation of the experimental results. Density functional theory (DFT) calculations suggested that two-dimensional Ru_*N*_ clusters are thermodynamically more favorable than their three-dimensional counterparts (Supplementary Figs. 68, 69). This trend is consistent with our MD simulations, in which three-dimensional Ru_*N*_ clusters were spontaneously stabilized into ALN structure below a critical size (*N*_Ru_ < 235) (Supplementary Fig. 6, Supplementary Movies 1-5). The theoretically predicted Ru-Ru bond lengths of Ru_*N*_/WC_x_ (Supplementary Fig. 70) are consistent with those obtained from EXAFS fitting for aRu*N*/WC_x_ (~2.74 Å), but not with those of NP-based samples (~2.67 Å; Ru_p_/WC_x__H_2_, Ru/WC_x__H_2_, Ru/WC_x__R, Ru/C_R). This characteristic elongation of the Ru-Ru distance is a distinctive signature of the ALN geometry. Furthermore, the experimentally observed Ru-Ru bond length elongation is inconsistent with both residual RuO_x_ species and incompletely dispersed Ru clusters and is more consistent with Ru ALN structures stabilized through simultaneous Ru-C and Ru-W coordination on WC_x_ (Supplementary Note 9).

Furthermore, the ensemble-size-dependent variation in the Ru-W CN obtained theoretically using a cutoff of <2.90 Å closely follows the experimental trend observed for aRu*N*/WC_x_, whereas the trend calculated using a cutoff of <2.70 Å does not (Supplementary Fig. 80a, b). Consistently, the Ru-W bond length extracted from EXAFS for aRu*N*/WC_x_ (Supplementary Fig. 80e) differs from that of alloy-type Ru-W NP samples (~2.72 Å; aRu5_p_/WC_x_, Ru5_p_/WC_x__Ar, Ru5/WC_x__Ar), reflecting the distinct coordination environment imposed by the two-dimensional Ru ALN structure (Supplementary Note 8). The consistency between the theoretical trends and experimentally obtained structural parameters supports the assignment of Ru ALNs as a predominant structural motif in aRu*N*/WC_x_.

Taken together, the Ru ALN model is consistent with the experimentally observed structural fingerprints, including the characteristic Ru-Ru bond-length elongation, the distinctive Ru-W coordination environment, direct Ru-C anchoring interactions, and their ensemble size-dependent evolution. The consistency between theory and experiment across these independent structural descriptors supports that Ru species in aRu*N*/WC_x_ are predominantly stabilized as Ru atom-layer neighbors on WC_x_ supports (Supplementary Note 8).

In wavelet transforms of EXAFS (WT-EXAFS), aRu*N*/WC_x_ exhibited an intensity maximum at lower *k* compared to RuO_2_ and Ru agglomerates in comparison samples, within the *k* range of 3 to 6 Å^−1^ (Fig. 3d, Supplementary Figs. 84, 85). This lower *k* suggests that coordinating atoms in the first shell of aRu*N*/WC_x_ are lighter than oxygen, potentially indicating the presence of carbon (Supplementary Note 8)^60^. To further distinguish the contributions of Ru-Ru and Ru-W in the second shell of aRu*N*/WC_x_, a detailed WT-EXAFS analysis that offers higher resolution in *k*-space at the expense of *R*-space resolution was conducted (Fig. 3e, Supplementary Note 8). The lower *k* region (red square) corresponds to Ru-Ru, while the higher *k* region (blue square) corresponds to Ru-W. The relative intensity of Ru-Ru compared to Ru-W increased as Ru contents increased, which reflects the systematic tuning of ensemble size by adjusting the Ru contents. In conclusion, the thermodynamic stabilization strategy successfully stabilized Ru ALNs with distinct atomic structures via MIs and SMSI with WC_x_, enabling systematic control over the ensemble size of Ru ALNs.

### Origin of the enhanced kinetics for Ru atom-layer neighbors

**Fig. 4:**
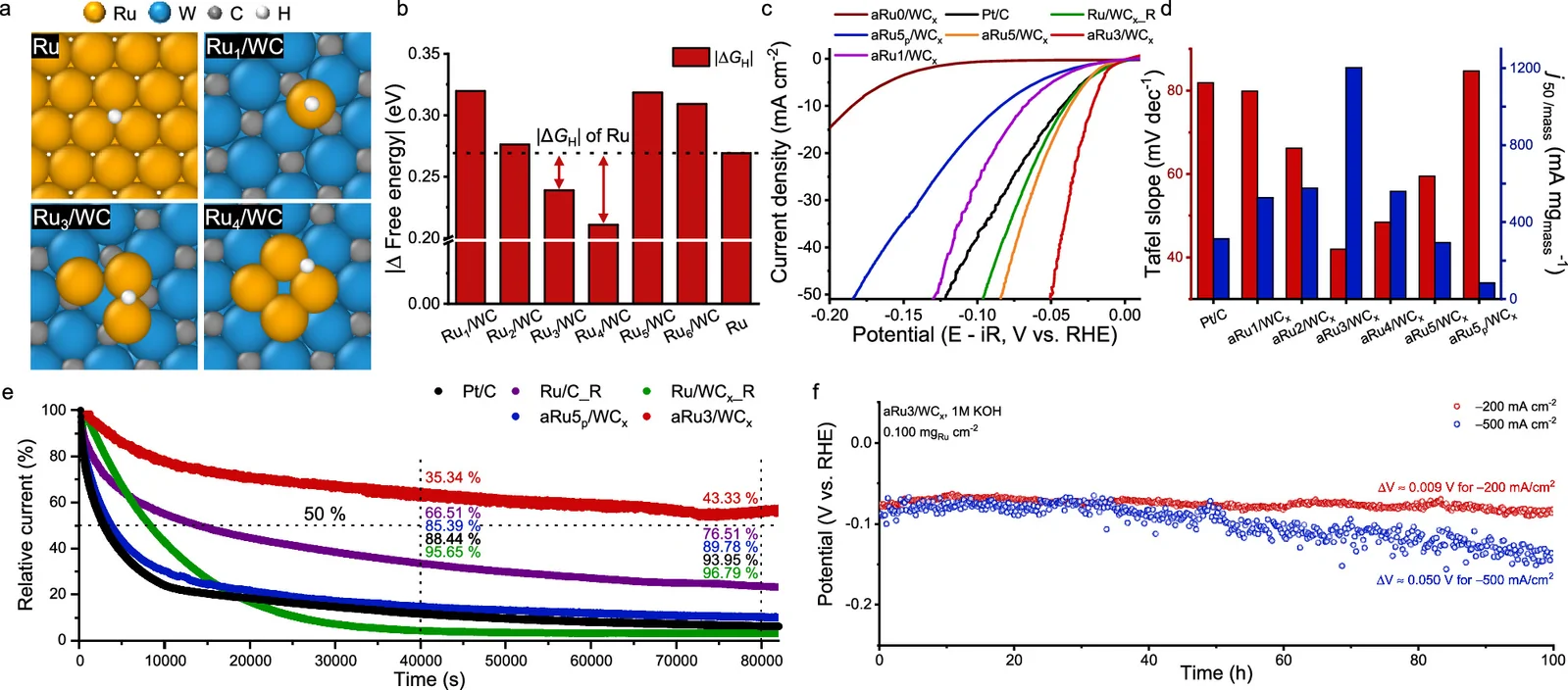
Alkaline HER performance and alteration of interfacial water structures. **a** Density functional theory (DFT)-optimized *H adsorption configuration on various catalysts. **b** Absolute values of Δ*G*_H_ for Ru ensembles on WC (Ru_*N*_/WC) structures. **c** Linear sweep voltammetry (LSV) curves, and **d** corresponding Tafel slope (red) and *j* per noble metal mass at −50 mV (vs. reversible hydrogen electrode (RHE)) for alkaline hydrogen evolution reaction (HER) in H_2_-saturated 1 M KOH solution. **e**, Relative current retention (%) during chronoamperometry at an overpotential of 50 mV over 80,000 s. **f** Chronopotentiometry for long-term durability test of aRu3/WC_x_ (0.100 mg_Ru_ cm^−2^) in 1 M KOH solution. The RDE polarization curves were iR-corrected using the EIS-derived uncompensated solution resistance measured before each experiment (*R*_u_ = 5.5 ± 1.5 Ω). The corresponding non-iR-corrected polarization curves are provided in Supplementary Fig. 91 f.

To evaluate the Gibbs free energy of hydrogen binding (Δ*G*_H_) based on the geometric structure of noble metal species, DFT calculations were performed. The ideal Δ*G*_H_ value for efficient HER is ~0 eV. Consistent with previous studies, hollow sites of Pt (111) and Ru (0001) provided the most stable *H adsorption, with Δ*G*_H_ for Ru (0001) calculated to be −0.269 eV (Fig. 4a, b, Supplementary Figs. 88, 90a, Supplementary Table 15). In contrast, isolated single-atom Ru on WC support (Ru_1_/WC) exhibited only top-site adsorption with Δ*G*_H_ of 0.320 eV. Ru ALNs on WC support enabled bridge-site adsorption of *H when neighboring Ru atoms were present. Notably, despite identical bridge-site adsorption, the Δ*G*_H_ of Ru ALNs varied with the number of Ru atoms in the Ru ALN, which can be attributed to the ligand effect from neighboring Ru atoms. In particular, Ru_3_/WC and Ru_4_/WC exhibited near ideal Δ*G*_H_ values of −0.239 eV and −0.211 eV, respectively. Furthermore, WC_x_ supports showed favorable Gibbs free energy for oxygenated species, such as *OH and *H_2_O, facilitating the enrichment of *OH species near *H binding sites (Supplementary Figs. 89, 90b, Supplementary Table 16).

Under the alkaline conditions (H_2_-saturated 1 M KOH), the HER activity of aRu*N*/WC_x_ showed a volcano-type dependence on the ensemble size of Ru ALNs, with aRu3/WC_x_ achieving the lowest overpotential for −10 mA cm^−2^ (*η*_10_) at 22 mV (potentials referenced to the reversible hydrogen electrode, RHE) and Tafel slope of 41.9 mV dec^−1^ (Fig. 4c, d, Supplementary Figs. 91, 92, Supplementary Tables 17, 18). Similarly, the trend in kinetic activity remained consistent at an identical Ru loading of 0.042 mg cm^−2^ (Supplementary Fig. 93). Furthermore, aRu3/WC_x_ (−1202.0 mA mg_mass_^−1^), showed a higher current density at an overpotential of 50 mV per noble metal mass (*j*_50/mass_) than commercial Pt/C, which displayed *η*_10_ of 42 mV, a Tafel slope of 81.9 mV dec^−1^, and *j*_50/mass_ of −312.0 mA mg_mass_^−1^ at 50 mV under identical noble metal loading (0.042 mg cm^−2^). DFT calculations (Fig. 4a) suggest that the enhanced activity of aRu3/WC_x_ within the aRu*N*/WC_x_ series is associated with the ensemble-size-dependent modulation of *H binding, consistent with Ru_3_/WC_x_ or Ru_4_/WC_x_-like local structures being major contributors to the observed activity (Supplementary Note 10). By contrast, Ru NPs and agglomerates in comparison samples exhibited lower performance due to unfavorable Δ*G*_H_ values or lower noble metal utilization efficiency of particulate Ru (Fig. 4c, d, Supplementary Figs. 91, 92, Supplementary Table 17). During the long-term chronoamperometry at an overpotential of 50 mV, aRu3/WC_x_ showed a smaller decrease in activity than the comparison catalysts tested under the same conditions, showing only about half the current density loss of commercial Pt/C and other comparison catalysts (Fig. 4e, Supplementary Fig. 95). To minimize the mechanical and hydrodynamic effects induced by electrode rotation, the durability test was conducted using aRu3/WC_x_ coated onto carbon paper, which is a typical substrate for durability assessment^39,61^. During the 100 h chronopotentiometry, aRu3/WC_x_ exhibited only minor voltage losses of Δ*V* ≈ 0.009 V at −200 mA cm^−2^ and Δ*V* ≈ 0.050 V at −500 mA cm^−2^ without any noticeable initial current decrease, owing to the SMSI between Ru ALNs and WC_x_ supports (Fig. 4f). The post-durability analyses confirmed that aRu3/WC_x_ preserved its mesoporous morphology and atomic-scale Ru dispersion after the long-term durability test, without forming the large Ru agglomerates (Supplementary Figs. 96, 97).

To examine the general applicability of the present ALN stabilization strategy, aPt3/WC_x_ and aIr3/WC_x_ were synthesized using hydrophobic Pt and Ir precursors, dimethyl(1,5-cyclooctadiene)platinum(II) (HPB Pt) and ((1,5-cyclooctadiene)-*η*^5^-indenyl)iridium(I) (HPB Ir), respectively, under identical noble metal molar loading conditions to those used for aRu3/WC_x_ (Supplementary Note 11). Structural analyses revealed that Ir could be stabilized as ALN-like structures on WC_x_ through the same stabilization mechanism, whereas Pt formed partially aggregated nanoclusters anchored on WC_x_ (Supplementary Figs. 98–105, Supplementary Table 19). These different stabilization behaviors suggest that thermodynamically favorable noble metal-WC_x_ interactions alone are insufficient for well-defined ALN formation, and that precursor-directed MIs during EISA are critical for suppressing premature aggregation and enabling surface confined ALN structures. The alkaline HER activities of the resulting catalysts were additionally evaluated at identical noble-metal loadings (42 μg cm^−2^). aPt3/WC_x_ exhibited an *η*_10_ of 26 mV and a Tafel slope of 25.8 mV dec^−1^, comparable to those of aRu3/WC_x_, whereas aIr3/WC_x_ showed relatively lower HER activity with *η*_10_ of 51 mV and a Tafel slope of 97.2 mV dec^−1^ (Supplementary Fig. 106).

**Fig. 5:**
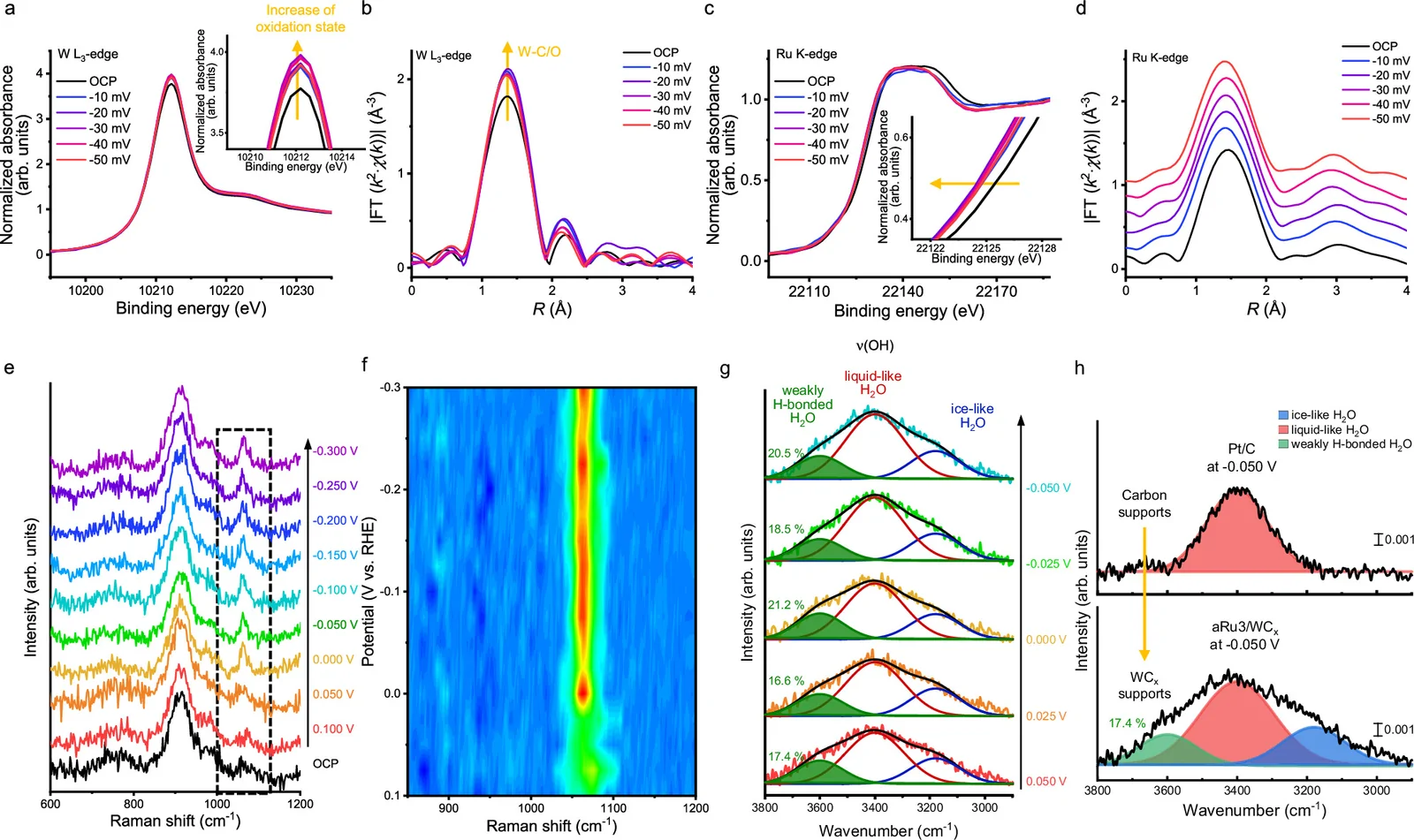
Dynamic modulation of the Ru ALNs-WC_x_ interfacial environment revealed by in situ spectroscopy. In situ (**a**) XANES (Inset: enlarged view of the selected region of the main panel) and (**b**) corresponding Fourier-transformed extended X-ray absorption fine structure (FT-EXAFS) spectra at the W L_3_-edge of aRu3/WC_x_ at different potentials. In situ (**c**) XANES (Inset: enlarged view of the selected region of the main panel) and (**d**) corresponding FT-EXAFS spectra at the Ru K-edge of aRu3/WC_x_ at different potentials. In (**b** and **d**), *R* denotes the radial distance in Fourier-transformed EXAFS *R* space. **e** In situ Raman spectra collected at sequential potentials (green shaded region: the coupled O=W···W-OH mode (~1060 cm^−1^)). **f** Difference contour map generated from the in situ Raman spectra, showing the potential-dependent evolution of Raman intensity. **g** In situ attenuated total reflection surface-enhanced infrared absorption spectroscopy (ATR-SEIRAS) spectra of aRu3/WC_x_ at sequential potentials. **h** Comparison of in situ ATR-SEIRAS spectra at -0.050 V for Pt/C and aRu3/WC_x_. All potentials are referenced to the reversible hydrogen electrode (RHE).

To reveal the origin of the enhanced kinetic activity of aRu3/WC_x_, in situ XAS analyses were conducted under alkaline HER conditions (Fig. 5a-d). To monitor the potential-dependent structural evolution, in situ XANES and FT-EXAFS spectra were collected at both the W L_3_-edge and Ru K-edge under a series of stepwise applied potentials, from open-circuit potential (OCP) to −50 mV (vs. RHE) with 10 mV intervals. At the W L_3_-edge, the white line intensity of aRu3/WC_x_ increased when an overpotential was applied compared to that at the OCP (Fig. 5a), indicating an increase in the oxidation state of W. Likewise, the intensity of the first-shell peak in the corresponding FT-EXAFS spectra also increased under HER operation (Fig. 5b), suggesting a rise in the W-O/C CN. These results imply that the surface of WC_x_ supports becomes enriched with oxygenated species (e.g., *OH or *O) during alkaline HER, consistent with previous reports^62,63^. In contrast, at the Ru K-edge, the edge position of aRu3/WC_x_ was negatively shifted relative to that at the OCP (Fig. 5c), indicating a partial reduction of Ru species due to *H adsorption on the Ru active sites^64^. Moreover, no noticeable change was observed in the FT-EXAFS spectra at the Ru K-edge (Fig. 5d), suggesting that the Ru ALN structure remained intact during HER, maintaining its ALN configuration throughout the reaction.

To identify the surface species formed on the WC_x_ supports, in situ Raman spectroscopy was performed at OCP and under sequentially applied potentials ranging from 0.100 V to −0.300 V (vs. RHE) with 0.050 V intervals (Fig. 5e, f). The intensity of the Raman band centered at ~1060 cm^−1^ increased as the potential decreased from 0.100 V to 0.000 V relative to that at the OCP, and then remained nearly constant upon further decrease to −0.300 V. According to previous reports, Raman bands observed around ~1060 cm^−1^ are typically assigned to coupled O = W···W-OH vibrations in tungsten-based compounds^65^. Combining the in situ Raman and in situ XAS results, it can be concluded that the surface of the WC_x_ supports becomes enriched with oxygenated species (e.g., *OH and *O) under alkaline HER conditions, consistent with the potential-dependent oxidation observed in the W L_3_-edge XAS. Such surface oxygenation behavior is consistent with previous reports that transition metal carbides possess inherently high oxygen affinity, leading to spontaneous adsorption of oxygenated species in the presence of water during the catalytic reactions^49,66^.

Previous research has identified that the depletion of water in the gap region, namely the gap water, causes sluggish alkaline HER kinetics by reducing the connectivity within the H-bond networks^46^. To investigate variations in the interfacial water structure across support materials, in situ attenuated total reflection surface-enhanced infrared absorption spectroscopy (ATR-SEIRAS) was employed. Peaks at 3600, 3400, and 3180 cm^−1^, assigned to the O-H stretching mode (ν_O-H_) of water, can be attributed to weakly hydrogen-bonded, liquid-like, and ice-like water, respectively^67^. According to the electric double layer-based model, the 3600 cm^−1^ band corresponds to water molecules located in the interfacial gap region with partially disrupted hydrogen bonding, which act as bridging species linking the nearest interfacial water species and the bulk electrolyte^46,68^. Therefore, an increased population of gap water facilitates proton transfer between bulk water and inner Helmholtz layer through an improved H-bond network. During alkaline HER, the proportion of gap water for aRu3/WC_x_ (17.4%) was significantly greater than that of Pt/C (Fig. 5g, h, Supplementary Fig. 107). This indicates that the *OH species on WC_x_ supports significantly enhance the H-bond network connectivity by increasing the gap water population, thereby accelerating HER kinetics compared to Pt/C with carbon supports (Supplementary Note 12).

The electrochemical characteristics of aRu3/WC_x_ are also distinct from those typically reported for hydrogen-spillover-dominated HER electrocatalysts^69^. Previous studies on hydrogen-spillover-based HER electrocatalysts have generally reported characteristic Tafel slopes of ~20−25 mV dec^−1^ associated with spillover-assisted HER kinetics^70,71^, whereas aRu3/WC_x_ exhibited a larger Tafel slope of 41.9 mV dec^-1^ (Supplementary Fig. 91). In addition, hydrogen-spillover-based electrocatalysts often exhibit pronounced electrochemical hydrogen desorption features originating from substantial populations of spilled-over hydrogen species on the support surface^71,72^. In contrast, aRu3/WC_x_ did not exhibit any noticeable hydrogen desorption peak in CV measurements (Supplementary Fig. 108), suggesting that substantial accumulation of spilled-over hydrogen species on the WC_x_ support does not occur in our system. Taken together, these results support a mechanism in which the WC_x_ support primarily enhances interfacial proton-transfer kinetics through H-bond network modulation, while the Ru ALNs remain the principal HER-active sites for hydrogen adsorption and H_2_ evolution.

To distinguish the effects of the active-site structure and WC_x_ support, additional DFT calculations were performed for Ru ensembles supported on graphene and γ-WC models, together with Bader charge and Δ*G*_H_ analyses (Supplementary Figs. 109, 110). Both the Bader charge and Δ*G*_H_ values varied with the Ru ensemble size on both supports, indicating that neighboring Ru-Ru interactions significantly influence the electronic structure and hydrogen adsorption energetics of Ru active sites. Furthermore, Ru ensembles on γ-WC consistently exhibited lower Bader charge values and Δ*G*_H_ values closer to the ideal hydrogen adsorption free energy than those on graphene, suggesting that additional electronic modulation can be induced through strong Ru-WC_x_ interactions. These results suggest that both the Ru ensemble structure and the WC_x_ support contribute to optimizing the hydrogen adsorption energetics for alkaline HER.

To experimentally distinguish the active-site structure effect and the WC_x_ support effect, atomically dispersed Ru clusters on N-functionalized carbon (aRu/NC) with a Ru-Ru CN of 1.92 comparable to that of aRu3/WC_x_ (CN = 1.72) were synthesized (Supplementary Figs. 111, 112). In addition, Ru NPs supported on WC_x_ and carbon (RuNP/WC_x_ and RuNP/C) were prepared to directly examine the support effect for similar NP active-site structures (Supplementary Figs. 113–115). During alkaline HER (Supplementary Fig. 116), aRu3/WC_x_ exhibited substantially higher HER activity (*η*_10_: 22 mV, Tafel slope: 41.9 mV dec^−1^) compared with aRu/NC (*η*_10_: 128 mV, Tafel slope: 105.5 mV dec^−1^), despite their similar Ru coordination environments. Likewise, RuNP/WC_x_ exhibited better HER activity (*η*_10_: 20 mV, Tafel slope: 30.8 mV dec^−1^) than RuNP/C (*η*_10_: 36 mV, Tafel slope: 52.1 mV dec^−1^), further confirming the beneficial role of the WC_x_ support. These results collectively indicate that enhanced HER activity of aRu3/WC_x_ originates from the cooperative effects of (i) optimized hydrogen adsorption energetics induced by both the Ru ensemble size and strong Ru-WC_x_ interactions and (ii) enhanced proton-transfer kinetics arising from the favorable interfacial water structure provided by the WC_x_ support compared with inert carbon supports (Fig. 5g, h, Supplementary Note 12).

At the same time, comparison between aRu/NC and RuNP/C further suggests that neighboring Ru-Ru interactions themselves also strongly influence HER activity under inert carbon supported conditions, as Ru NPs exhibited substantially higher activity than small Ru clusters supported on carbon. This trend is also consistent with the DFT results, which predict relatively non-ideal Δ*G*_H_ values for low-coordination Ru ensembles on inert carbon supports compared with Ru ensembles stabilized on γ-WC (Fig. 4a, b, Supplementary Figs. 109, 110). Although RuNP/WC_x_ and aRu3/WC_x_ possess different active-site geometries, both catalysts exhibited high HER activity owing to the beneficial Ru-WC_x_ interactions, together with the favorable interfacial water structure provided by the WC_x_ support. However, unlike Ru NPs, all Ru atoms in the Ru ALN structures are directly stabilized through chemical interactions with WC_x_, enabling substantially stronger SMSI stabilization and consequently greater durability during long-term HER operation (Supplementary Fig. 116d).

Therefore, the DFT calculations, together with experimental control studies, support that the enhanced alkaline HER activity of Ru ALNs originates from the cooperative contributions of (i) electronic structure modulation induced by both neighboring Ru-Ru interactions and strong Ru-WC_x_ interactions, (ii) adsorption-configuration changes enabled by neighboring Ru sites, and (iii) interfacial kinetic enhancement provided by the WC_x_ support.

To sum up, aRu3/WC_x_ exhibited higher kinetic activity than commercial Pt/C through two synergistic effects of dual-type sites (Ru ALNs for *H and WC_x_ supports for *OH) revealed by theoretical calculations and in situ spectroscopic analyses. First, the OH-enriched WC_x_ supports increased the population of gap water at the catalyst-electrolyte interface, which bridges the active sites and the bulk electrolyte, thereby restoring the H-bond network continuity and facilitating proton transfer across the interface. Second, the controlled ensemble size of Ru ALNs regulates the H-adsorption configuration and strength through cooperative geometric coupling among adjacent Ru atoms and electronic interactions with the underlying WC_x_ supports, maintaining a stable and highly active Ru configuration during alkaline HER operation. These cooperative effects provide a plausible explanation for the enhanced reaction kinetics of aRu3/WC_x_, as consistently demonstrated by the in situ XAS, Raman, and ATR-SEIRAS results (Fig. 5).

### Single-cell performances of Ru atom-layer neighbors

**Fig. 6:**
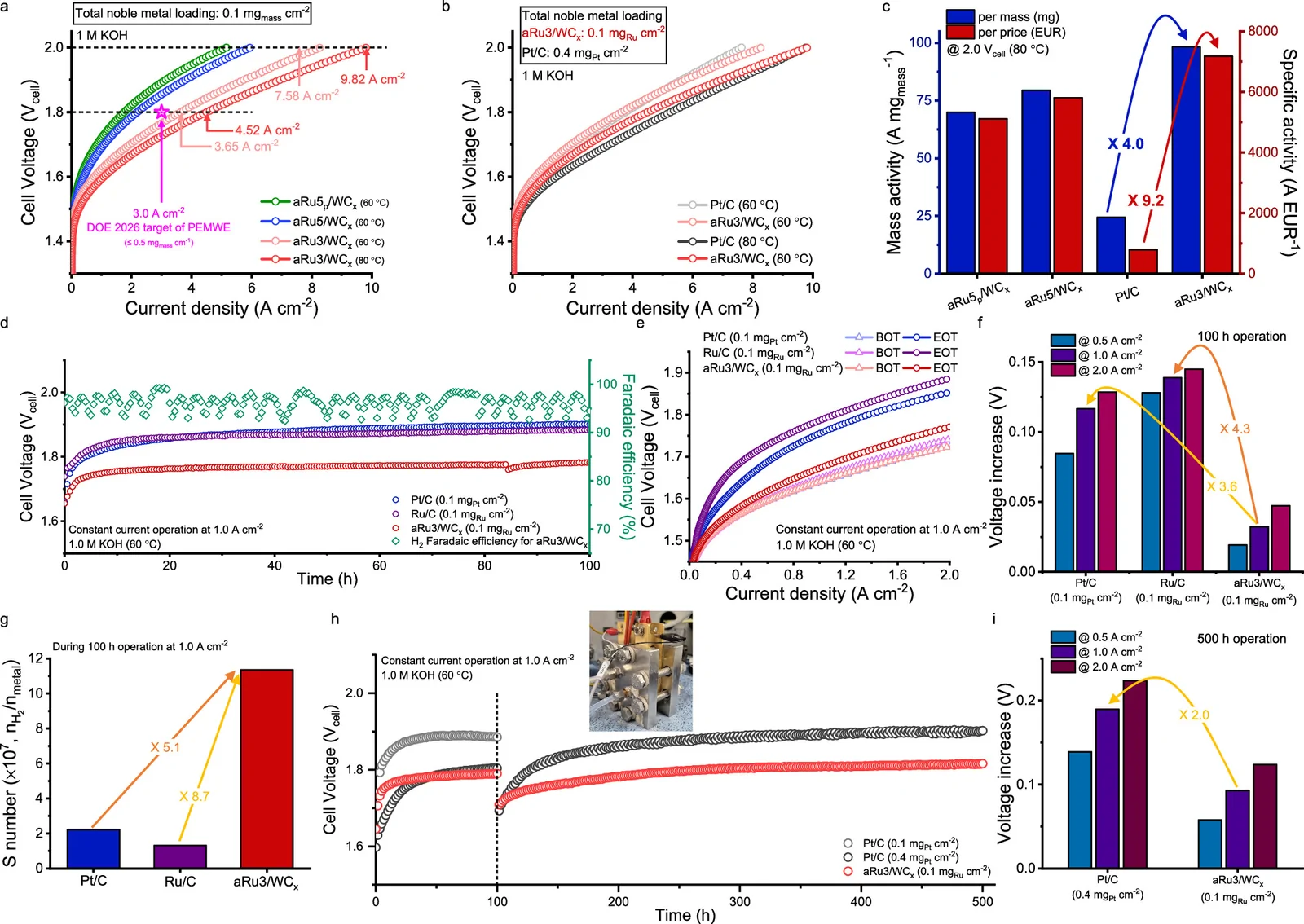
AEMWE performance and durability of Ru ALNs. **a**, Polarization curves of anion-exchange membrane water electrolyzers (AEMWEs) using aRu3/WC_x_ (0.10 mg_Ru_ cm^−2^) and Pt/C (0.10 mg_Pt_ cm^−2^) with 1 M KOH electrolyte at operating *T* of 60 °C and 80 °C. **b**, Polarization curves of AEMWEs using aRu3/WC_x_ (0.10 mg_Ru_ cm^−2^) and Pt/C (0.40 mg_Pt_ cm^−2^) under identical conditions. **c**, Comparison of mass activity and specific activity at 2.0 V_cell_ and 80 °C. The noble metal costs are detailed in Supplementary Table 1. **d**, Cell voltage profiles and corresponding H_2_ Faradaic efficiency during AEMWE operation (60 °C, 1 M KOH) at a constant current density of 1.0 A cm^−2^ for 100 h, with an identical noble metal loading of 0.10 mg_mass_ cm^−2^. **e**, Polarization curves of AEMWEs before (BOT) and after (EOT) 100 h operation at 1.0 A cm^−2^. **f**, Corresponding cell voltage increases after 100 h operation at 1.0 A cm^−2^. **g**, Comparison of S-number obtained during 100 h operation at 1.0 A cm^−2^. **h**, Long-term durability test of AEMWEs operated for 500 h at a constant current density of 1 A cm^−2^ and 60 °C, using aRu3/WC_x_ (0.10 mg_Ru_ cm^−2^, red) and commercial Pt/C (0.10 mg_Pt_ cm^−2^, gray; 0.40 mg_Pt_ cm^−2^, black). The inset shows a photograph of the AEMWE single-cell used for the long-term durability test. **i**, Corresponding cell voltage increase after 500 h operation at 1.0 A cm^−2^. The AEMWE polarization curves are shown without iR correction. All AEMWE voltages are reported as full-cell voltages (V_cell_).

aRu3/WC_x_ provides an effective catalyst architecture for efficient, low-noble metal AEMWEs. The ALN architecture of Ru ALNs combines the advantages of both SACs and NP catalysts to maximize Ru utilization while creating a favorable environment for the HER. To assess the practical applicability of aRu3/WC_x_, single-cell performance was investigated under practical AEMWE operating conditions using 1 M KOH. Among the aRu*N*/WC_x_-based AEMWEs, the aRu3/WC_x_ reached a current density of 9.82 A cm^-2^ at 2.0 V_cell_ and 80 °C with a low noble metal loading of 0.10 mg_Ru_ cm^-2^ (Fig. 6a, Supplementary Fig. 118, Supplementary Table 20). This result indicates that size-tuned Ru ALNs contribute to the increase in AEMWE performance under identical Ru loading (0.10 mg_Ru_ cm^-2^). Furthermore, the aRu3/WC_x_-based AEMWE with a lower noble metal loading (0.1 mg_Ru_ cm^-2^) achieved a comparable current density of 9.82 A cm^-2^ to the benchmark Pt/C-based AEMWE (9.77 A cm^-2^) with a higher noble metal loading of 0.4 mg_Pt_ cm^-2^ (Fig. 6b, Supplementary Figs. 117, 118, Supplementary Table 20). Owing to its lower noble metal loading and the comparatively lower cost of Ru, aRu3/WC_x_ achieved 4.0 times higher mass activity (98.2 A mg_mass_^−1^) and 9.2 times higher specific activity (activity per unit price of noble metal, 5880.9 A EUR^−1^) compared with Pt/C (Fig. 6c, Supplementary Table 20). At 1.8 V_cell_, aRu3/WC_x_ reached 4.52 A cm^-2^ at 80 °C and 3.65 A cm^-2^ at a milder operating *T* of 60 °C (Fig. 6a) despite its low noble metal loading of 0.10 mg_Ru_ cm^-2^, exceeding US DOE’s 2026 performance target (3.0 A cm^-2^ at 1.8 V_cell_) and ultimate noble metal loading target (0.125 mg cm^-2^) for PEMWEs^73^. Furthermore, the aRu3/WC_x_ cathode reached 3.10 A cm^-2^, exceeding the DOE 2026 target performance even with a commercial AEM and AEI (PiperION-A) at 60 °C (Supplementary Fig. 120). Among reported AEMWE catalysts, aRu3/WC_x_ demonstrated competitive specific activity and met both DOE targets even at low *T* of 60 °C (Supplementary Fig. 121 and Supplementary Table 21, DOE target of specific activity was calculated using Ru price).

To compare the activity and durability of aRu3/WC_x_ with those of commercial benchmark catalysts (Pt/C and Ru/C) at the single-cell level, AEMWE tests (1 M KOH, 60 °C) were performed under identical noble metal loading conditions (0.10 mg_mass_ cm^-2^). Among the tested cathodes, aRu3/WC_x_ delivered a current density of 8.30 A cm^−2^ at 2.0 V_cell_, compared with 7.23 A cm^-2^ for Pt/C and 6.58 A cm^−2^ for Ru/C (Supplementary Fig. 122). Furthermore, both Pt/C and Ru/C cathodes exhibited more pronounced degradation after 100 h operation at 1.0 A cm^−2^, as evidenced by larger differences between the polarization curves before (BOT) and after (EOT) testing (Fig. 6e) and greater voltage increases at each current density, compared to those of aRu3/WC_x_ (Fig. 6f). In particular, at 1 A cm^−2^, the voltage increase of aRu3/WC_x_ was approximately one-quarter of those for both Pt/C and Ru/C. To evaluate the durability of aRu3/WC_x_, the stability number (S-number) was determined by quantifying the generated H_2_ and the dissolved noble metal content during the 100 h durability test^74^. All samples exhibited nearly 100 % Faradaic efficiency for H_2_ based on real-time hydrogen quantification using a mass flow meter and gas chromatography (GC) analysis of the cathode outlet stream (Fig. 6d and Supplementary Figs. 123−126), indicating that the measured current predominantly originated from hydrogen evolution. At 1.0 A cm^−2^, aRu3/WC_x_ achieved an S-number of 1.136×10^8^, which is five times higher than that of Pt/C (2.221×10^7^) and nine times higher than that of Ru/C (1.311×10^7^) (Fig. 6g), indicating that the SMSI between the Ru ALNs and the WC_x_ supports contributes to the lower noble metal dissolution and voltage degradation of aRu3/WC_x_ under the tested AEMWE conditions.

To further evaluate the operational stability of the Ru ALNs under practical intermittent AEMWE conditions, intermittent start-stop cycling tests were additionally performed by alternating between 1.0 A cm^−2^ and open-circuit voltage (OCV) every 1 h for a total operation time of 100 h (Supplementary Fig. 127). aRu3/WC_x_ exhibited substantially smaller voltage degradation than commercial Pt/C even under repeated start-stop operation, indicating the high operational stability of the Ru ALN/WC_x_ system. In addition, inductively coupled plasma mass spectrometry (ICP-MS) analysis of dissolved metal species after the intermittent operation revealed no detectable Ru dissolution from aRu3/WC_x_, whereas measurable Pt dissolution was observed from commercial Pt/C (Supplementary Table 22). Although trace W dissolution was detected from aRu3/WC_x_, the dissolved amount was limited, indicating that the WC_x_ support remained largely stable during intermittent operation. These results further suggest that the Ru ALNs remain stably anchored on the WC_x_ support without significant detachment under practical alkaline AEMWE operating conditions.

To examine whether the high-current-density AEMWE performance originates from catalyst-dependent bubble behavior or mass transport effects, we systematically investigated the influence of catalyst structural features, including Ru ALN morphology, Ru aggregation, and support properties, on wettability, bubble evolution, and mass transport characteristics using aRu3/WC_x_, aRu5/WC_x_, aRu5_p_/WC_x_, commercial Ru/C and Pt/C cathodes. Most catalysts exhibited comparable contact angles (105.3 − 121.4°), except for aRu5_p_/WC_x_ (83.7°) (Supplementary Fig. 128). In addition, Pt/C and WC_x_-supported catalysts exhibited similarly rapid microbubble detachment behavior with no noticeable differences in bubble size or surface coverage during real-time optical visualization of hydrogen bubble evolution, whereas Ru/C showed localized bubble accumulation (Supplementary Fig. 129). Furthermore, multistep chronopotentiometry combined with EIS revealed comparable residual overpotentials for Pt/C and WC_x_-supported catalysts, while Ru/C exhibited a substantially larger residual contribution (Supplementary Fig. 130). Notably, aRu3/WC_x_ and aRu5/WC_x_ showed similar residual overpotentials, indicating that differences in Ru ALN morphology do not substantially affect mass-transport-related losses. These results demonstrate that the AEMWE performance of aRu3/WC_x_ does not primarily originate from bubble shielding or gas transport effects, but rather from the enhanced catalytic activity and favorable interfacial kinetics of the Ru ALNs on WC_x_.

The post-durability analyses of AEMWE operation revealed that both commercial Pt/C and Ru/C cathodes underwent severe NP agglomeration along with noticeable Fe/Ni deposition, originating from the migration and reduction of dissolved Fe/Ni ions from the anode (Supplementary Figs. 132, 133). The aRu3/WC_x_ cathode also exhibited Fe/Ni deposition (Supplementary Fig. 134), indicating that Fe/Ni redeposition is a common system-level degradation factor in AEMWE operation rather than a catalyst-specific phenomenon^75^. Although such Fe/Ni redeposition may partially contribute to active-site blocking and performance degradation, the markedly different durability behaviors among the investigated cathodes correlate more strongly with the degree of noble metal migration, aggregation, and dissolution. In this regard, aRu3/WC_x_ preserved the atomic structure of Ru species on the WC_x_ supports, showing only minor and localized Ru agglomeration even after prolonged operation (Supplementary Figs. 134, 135, Supplementary Note 13). These results indicate that the strong metal-support interaction between Ru ALNs and WC_x_ supports effectively suppresses Ru migration and aggregation, thereby contributing to the lower degradation of aRu3/WC_x_ compared with commercial nanoparticle catalysts. It should be noted that further mitigation of Fe/Ni redeposition requires advances in anode and membrane design beyond cathode catalyst optimization (Supplementary Note 13).

Even when compared with Pt/C cathodes with four times higher noble metal loading (0.40 mg_Pt_ cm^−2^), the aRu3/WC_x_ cathodes (0.10 mg_Ru_ cm^−2^) showed lower voltage degradation during 500 h operation at an industrially relevant current density of 1.0 A cm^−2^ (Fig. 6h and Supplementary Fig. 131). After 100 h, aRu3/WC_x_ retained comparable current density to Pt/C, and after 500 h, it exhibited a higher current density than Pt/C in the same voltage region (Supplementary Fig. 131c, d). Moreover, the voltage increase of the Pt/C cathode was approximately twice that of aRu3/WC_x_ after 500 h, despite having four times higher noble metal loading (Fig. 6i). These results indicate that the SMSI between Ru ALNs and the WC_x_ support contributes to the lower voltage degradation of aRu3/WC_x_ compared with Pt/C cathodes with higher noble metal loading under AEMWE operating conditions. Such activity-durability balance suggests the potential of aRu3/WC_x_ electrocatalysts for practical water electrolysis.

## Discussion

We introduced a platform of Ru ALNs that combines the advantages of SACs and NP catalysts. Their atomically thin structure, comprising a few Ru atoms, increases Ru utilization while enabling adsorbate binding modulation through the proximity of neighboring Ru atoms. By leveraging MIs and SMSIs, our thermodynamic stabilization strategy enabled the synthesis of size-tuned Ru ALNs on WC_x_ supports. The optimized aRu3/WC_x_ sample demonstrated enhanced alkaline HER activity owing to an optimized Δ*G*_H_ offered by size-tuned Ru ensembles and the enrichment of *OH species around these ensembles enabled by the WC_x_ supports. This *OH enrichment led to the acceleration of alkaline HER kinetics through enhancing the H-bond network. In AEMWE applications, aRu3/WC_x_ achieved competitive activity per unit noble metal cost among reported AEMWE electrocatalysts with a low Ru loading of only 0.10 mg cm^−2^. The ALNs platform offers an approach for catalysis applications requiring optimized ensemble sites with enhanced noble metal utilization while providing a strategy for tuning the performance of atomically dispersed catalysts.

## Methods

### Materials

Poly(ethylene oxide)-*block*-poly(styrene) (PEO-*b*-PS, *M*_n_: 50 kg mol^−1^, 10.0 wt% PEO) was synthesized via atom-transfer radical polymerization^76^, while phenol-formaldehyde resin was synthesized according to a reported procedure^76^. Phosphotungstic acid hydrate (H_3_PW_12_O_40_ ·xH_2_O, reagent grade), melamine-formaldehyde resin (poly(melamine-co-formaldehyde) methylated), ruthenium(III) chloride hydrate (RuCl_3_·xH_2_O, HPL Ru, 99.98%), and Nafion perfluorinated resin solution (5 wt% in lower aliphatic alcohols and water, contains 15 – 20% water) were purchased from Sigma-Aldrich. Sustainion® XA-9 alkaline ionomer (5 wt% in ethanol) was purchased from Dioxide Materials, and Bis(2-methylallyl)(1,5-cyclooctadiene)ruthenium(II) (HPB Ru, 97%) from Strem Chemicals. Additional materials included tetrahydrofuran (stabilized, 99.5%, J.T.Baker), hydrochloric acid (35.0 – 37.0 wt%), isopropanol (99.5%, JUNSEI), and 1 M KOH solutions, all purchased from SAMCHUN Pure Chemicals.

### Catalyst synthesis

#### Synthesis via self-assembly (aRu*N*/WC_x_, Thermodynamic stabilization)

Samples of aRu*N*/WC_x_ were synthesized using a modified interaction-mediator-assisted evaporation-induced self-assembly (IM-EISA) method^32^. In aRu*N*/WC_x_ notation, N indicates the molar ratio of the Ru precursor (either HPL Ru or HPB Ru) to the W precursor (phosphotungstic acid), with values defined as *N* = 0 for 0, *N* = 1 for 0.5, *N* = 2 for 1, *N* = 3 for 2, *N* = 4 for 3, *N* = 5 for 4. For the synthesis, 0.1 g of phenol-formaldehyde resin, 0.06 g of melamine-formaldehyde resin, and 0.2 g of PEO-*b*-PS were dissolved in 30 mL of THF in a vial and stirred vigorously for 1 h to obtain a clear solution. Next, 0.3 g of phosphotungstic acid was added to the solution and fully dissolved by sonication (30 min) followed by stirring (30 min). For *N* = 5, either 133.1 mg of HPB Ru (for aRu5/WC_x_) or 93.9 mg of HPL Ru (for aRu5_p_/WC_x_) was dissolved in 5 mL of tetrahydrofuran, then added to the mixture and dissolved through sonication (30 min) and stirring (1 h). The final solution was transferred to a petri dish and covered with a slightly gapped second petri dish, then the solvent was evaporated at 323 K for 12 h. The resulting film was aged further at 373 K for 48 h, peeled off, and finely ground manually in an agate mortar for 10 min. The powder was annealed in a tube furnace under Ar flow (200 sccm) at 723 K for 3 h (heating rate of 1 K min^−1^), 873 K (1 K min^−1^), and 1,173 K for 6 h (5 K min^−1^), then cooled to room temperature (*RT*, 298 K, 25 °C;5 K min^−1^). After cooling, the sample was passivated under O_2_ (2 sccm) and Ar (200 sccm) flow for 12 h, followed by O_2_ (4 sccm) and Ar (200 sccm). The powder was then finely ground in an agate mortar for 10 min.

#### Synthesis of aRu5/WO_x_ and aRu5_p_/WO_x_

For these samples, intended to prevent carburization of the W species, a lower-*T* annealing procedure than that used for aRu*N*/WC_x_ was applied. The synthesis steps were identical prior to annealing. The powder was annealed in a tube furnace under Ar flow (200 sccm) at 723 K for 3 h (1 K min^−1^), and 873 K for 6 h (1 K min^−1^), then cooled to *RT* (5 K min^−1^). Following cooling, the sample was passivated under O_2_ (2 sccm) and Ar (200 sccm) flow for 12 h, followed by O_2_ (4 sccm) and Ar (200 sccm) flow for another 12 h, and then finely ground in an agate mortar for 10 min.

#### Synthesis via incipient wetness impregnation (IWI) with low-*T* reduction

Generally, to prevent thermal sintering of Ru species, low-*T* reduction was employed for synthesizing SACs. For control experiments, Ru/WC_x__H_2_ (HPB Ru) and Ru_p_/WC_x__H_2_ (HPL Ru) were prepared via IWI with a target Ru loading of ~10 wt% (equivalent Ru loading to aRu5/WC_x_). For each synthesis, 0.176 g of HPB Ru (Ru/WC_x__H_2_) or 0.124 g of HPL Ru (Ru_p_/WC_x__H_2_) was dissolved in 30 mL of tetrahydrofuran, sonicated for 10 min, and stirred for 10 min to obtain a homogeneous solution. Next, 0.5 g of aRu0/WC_x_ (samples without Ru content) was added to the solution, dispersed by sonication for 10 min, then stirred at 323 K until complete solvent evaporation. The resulting powder was dried at 323 K under vacuum for 12 h. It was then annealed in a tube furnace under H_2_ (20 sccm) and Ar (180 sccm) flow at 473 K for 2 h (1 K min^−1^), then cooled to *RT* (5 K min^−1^). After cooling to *RT*, the sample was passivated under O_2_ (2 sccm) and Ar (200 sccm) flow for 12 h, followed by O_2_ (4 sccm) and Ar (200 sccm) flow for 12 h, and finely ground in an agate mortar for 10 min.

#### Synthesis via IWI with high-*T* annealing

In control experiments, Ru/WC_x__Ar (HPB Ru) and Ru_p_/WC_x__Ar (HPL Ru) were synthesized by IWI with a target Ru loading of ~10 wt% (equivalent Ru loading to aRu5/WC_x_). The synthesis steps were identical to the above process until the annealing stage. The powder was annealed in a tube furnace under Ar (200 sccm) flow at 1,173 K for 6 h (5 K min^−1^), then cooled to *RT* (5 K min^−1^). Upon reaching *RT*, the sample was passivated under O_2_ (2 sccm) and Ar (200 sccm) flow for 12 h, followed by O_2_ (4 sccm) and Ar (200 sccm) flow for 12 h, and finely ground in an agate mortar for 10 min.

#### Synthesis of particulate Ru samples by chemical reduction

For control experiments, Ru/WC_x__R and Ru/C_R samples were prepared by chemically reducing HPL Ru with NaBH_4_, targeting a Ru loading of ~20 wt% (equivalent noble metal loading to commercial Pt/C). For this synthesis, 167 mg of HPL Ru was dissolved in 60 mL of ethanol, sonicated for 10 min, and stirred for 30 min to obtain a homogeneous solution. Next, 0.3 g of aRu0/WC_x_ (Ru/WC_x__R) or Vulcan XC 72 R (Ru/C_R) was added and dispersed by sonicating for 10 min, followed by vigorous stirring. Separately, 0.6 g of NaBH_4_ was dissolved in 50 mL of ethanol and stirred to obtain a homogeneous solution, then added dropwise to the mixture solution and stirred vigorously for 4 h. The resulting powder was centrifuged at 5,588 × g (7000 rpm) for 5 min and washed with water and ethanol multiple times. The final powder was dried at 323 K under vacuum for 12 h, then finely ground in an agate mortar for 10 min.

#### Synthesis of aRu/NC

For control experiments, aRu/NC was prepared by IWI using soft-nitrided Vulcan XC 72R^77^. For the synthesis, 27.2 mg of HPL Ru was dissolved in 20 mL of ethanol, sonicated for 10 min, and stirred for 10 min to obtain a homogeneous solution. Next, 0.2 g of soft-nitrided carbon was added to the solution, dispersed by sonication for 10 min, then stirred at 323 K until complete solvent evaporation. The resulting powder was dried at 323 K under vacuum for 12 h. It was then annealed in a tube furnace under H_2_ (20 sccm) and Ar (180 sccm) flow at 473 K for 2 h (1 K min^−1^), then cooled to *RT* (5 K min^−1^). After cooling to *RT*, the sample was passivated under O_2_ (2 sccm) and Ar (200 sccm) flow for 12 h, and finely ground in an agate mortar for 10 min.

#### Synthesis of RuNP/WC_x_ and RuNP/C

0.16 g of HPL Ru was dissolved in 20 mL of ethylene glycol in a vial. Separately, 0.4 g of NaOH was dissolved in 20 mL of ethylene glycol in a three-neck round-bottom flask. After complete dissolution, the two solutions were combined and vigorously stirred for 1 h under continuous Ar purging to remove dissolved oxygen. The resulting solution was then heated to 433 K for 3 h (4 K min^−1^), followed by natural cooling to *RT*. A stable dispersion of Ru NPs in ethylene glycol was obtained. For ligand removal and support immobilization, 5 mL of Ru NP dispersion was mixed with 30 mL of 1.0 M HCl solution in a conical tube after ultrasonication of the dispersion. The mixture was centrifuged at 16,421 × g (12,000 rpm) for 10 min, and the supernatant was discarded. This washing process was repeated three times using a total of 15 mL of Ru NP dispersion. Subsequently, the precipitate was additionally washed with 30 mL and then 10 mL of 1.0 M HCl solution via centrifugation at 16,421 × g (12,000 rpm) for 10 min to collect the Ru NPs at the bottom of the tube. After acid washing, 117 mg of support materials (WCx or Vulcan XC 72 R) and 5 mL of acetone were added to the conical tube. The resulting mixture was ultrasonicated for 1 h in an ice bath while preventing solvent evaporation. The obtained catalyst powder was collected by centrifugation at 5,588 × g (7000 rpm) for 5 min and dried under vacuum at 323 K overnight. The dried samples were subsequently annealed in a tube furnace under H_2_ (20 sccm) and Ar (180 sccm) flow at 473 K for 2 h (1 K min^−1^), followed by an additional 2 h under pure Ar (200 sccm). The samples were then cooled to *RT* (5 K min^−1^) and passivated under O_2_ (2 sccm) and Ar (200 sccm) flow for 12 h.

#### Synthesis of aPt3/WC_x_ and aIr3/WC_x_

The synthetic procedure was identical to that used for aRu3/WC_x_, except that dimethyl(1,5-cyclooctadiene)platinum(II) (HPB Pt, 69.4 mg) and ((1,5-cyclooctadiene)-*η*^5^-indenyl)iridium(I) (HPB Ir, 86.4 mg) were employed as noble metal precursors for aPt3/WC_x_ and aIr3/WC_x_, respectively. The noble metal-to-W molar ratio was maintained identical to that used for aRu3/WC_x_.

### Characterizations

X-ray diffraction (XRD) patterns were analyzed using a D8 advanced X-ray diffractometer (Bruker) equipped with Cu Kα radiation (*λ* = 1.541 Å). Field-emission scanning electron microscopy (FE-SEM) images were acquired with a SU8230 scanning electron microscope (Hitachi). High-resolution transmission electron microscopy (TEM), high-angle annular dark field scanning transmission electron microscopy (HAADF-STEM), energy-dispersive spectroscopy (EDS) mapping, and electron energy-loss spectroscopy (EELS) line scans were conducted on a Tecnai G2 F30 S-Twin transmission electron microscope (FEI) with an accelerating voltage of 300 kV. EELS spectra were acquired with an energy dispersion of 0.6 eV channel^−1^ over an energy-loss range of 1850 eV. During line acquisition, the probe was rastered along the selected direction with a spatial step size of 0.2 Å, and spectra were recorded sequentially at each pixel to construct the Ru L-edge and W M-edge areal-density profiles. N_2_ adsorption-desorption isotherms at 77 K were measured using a Tristar II 3020 physisorption analyzer (Micromeritics). Elemental analysis for C, H, N, and S was performed on a FlashSmart elemental analyzer (Thermo Fisher Scientific), while O content was determined using a Flash 2000 series elemental analyzer (Thermo Scientific). Metal mass ratios were quantified using a ZSX Primus II X-ray Fluorescence Spectrometer (Rigaku). For simultaneous TGA-DSC analysis, solvent-cast precursor/polymer composites were prepared by dissolving PEO and HPL Ru in ethanol or PS and HPB Ru in THF, followed by solvent evaporation at 50 °C. Simultaneous thermogravimetric analysis and differential scanning calorimetry (TGA-DSC) were performed using a LABSYS Evo thermal analyzer (Setaram). The samples were heated from *RT* to 900 °C at a heating rate of 10 °C min^−1^ under Ar flow. Small-angle X-ray scattering (SAXS) measurements were performed at the 4 C SAXS beamline at the Pohang Accelerator Laboratory (PAL), Korea. The X-ray energy was set to 10.0 keV, and the sample-to-detector distance was 4 m. The magnitude of the scattering vector (*q*) was calibrated using silver behenate (Tokyo Chemical Industry, TCI), while Ti-SBA−15 (Sigma-Aldrich) was employed as a structural reference material. Scattered X-rays were recorded using a two-dimensional CCD detector (Rayonix SX165, Evanston). The 2D scattering images were circularly averaged with respect to the beam center to obtain one-dimensional profiles and subsequently normalized to the transmitted X-ray beam intensity. The SAXS intensity profiles and Kratky plots were presented within the experimentally accessible *q* range of 0.67−1.4 nm^−1^. X-ray absorption spectroscopy (XAS) spectra at the Ru K-edge and W L_3_-edge were acquired at the 8 C Nano XAFS beamline at PAL, Korea. XAS measurements were performed in transmission and fluorescence modes using pelletized catalyst powders. Ru and W metal references were measured concurrently to calibrate each sample. Energy calibration, background subtraction, and normalization of XAS spectra were processed with Athena from the Demeter software package, while Artemis of the Demeter software package was used for the fitting of Fourier-transformed *k*^2^-weighted extended X-ray absorption fine structure (EXAFS) spectra. *S*_0_^2^ values were determined by fitting Ru and W standards, and *k*^2^-weighted EXAFS wavelet transforms (WT-EXAFS) were analyzed using the Morlet wavelet transform with the *η*·*σ* value of 10 for overviews and ~2 for detailed views^78^.

### Electrochemical measurements

Electrochemical measurements were performed using a Reference 600 potentiostat (Gamry Instruments) in a three-electrode configuration. An Hg/HgO reference electrode with 1 M NaOH solution was used, while a graphite rod served as the counter electrode to prevent Pt contamination on the working electrode. For reference electrode calibration, Pt wires as working and counter electrodes were used in H_2_-saturated 1 M KOH solution, resulting in a shift of -0.928 V ± 0.001 (vs. RHE), based on five independent measurements. All potentials were converted to the RHE scale using the following relation: *E*_RHE_ = *E*_Hg/HgO_ + 0.928 V. All synthesized catalysts were finely milled using zirconia balls in a laboratory jar mill.

A 5 mm glassy carbon rotating disk electrode (RDE) was polished on spec-cloth with 0.3 and 0.05 μm alumina suspensions to a mirror finish prior to catalyst ink loading. 40 mg of Vulcan XC 72 R was uniformly dispersed in 20 mL of isopropanol by sonicating for 1 h. The catalyst ink was prepared by dispersing 6.5 mg of catalyst in 368 μL of DI water, 32 μL of Sustainion® XA-9 ionomer solution, and 1600 μL of Vulcan-dispersed isopropanol via sonication for 2 h, yielding a catalyst loading of ~0.6 mg cm^−2^. For the generalized comparison, commercial Pt/C (HISPEC 3000 Johnson Matthey Co.) loaded RDE was applied with widely used conditions^79^. For commercial Pt/C, 3.4 mg of Pt/C was dispersed in 370 μL of DI water, 30 μL of Nafion solution, and 1600 μL of Vulcan-dispersed isopropanol with sonication for 2 h, yielding a Pt loading of 0.042 mg cm^−2^ (the nominal loading of Pt)^79^. A 12 μL aliquot of catalyst ink was deposited on the RDE rotating at 150 rpm and dried at ambient conditions with the RDE rotating at 400 rpm. To achieve uniform coating, this procedure was repeated three times for synthesized catalysts and twice for commercial Pt/C.

Polytetrafluoroethylene (PTFE) cell and bubbler were treated in a 333 K water bath for 1 h to eliminate impurities prior to each measurement^79^. Commercial 1 M KOH solution was used as received and stored in a sealed container at *RT*. Fresh electrolyte was taken from the stock solution before each experiment. H_2_-saturated 1 M KOH solution was used as the electrolyte for alkaline conditions, and all applied potentials were converted to the reversible hydrogen electrode (RHE) scale based on calibrated results. The nominal pH of the 1 M KOH electrolyte was approximately 14. All electrochemical measurements were conducted after vigorous purging with H_2_ (99.999 %) for at least 10 min. Prior to measurements, cyclic voltammetry was performed at 1600 rpm with a scan rate of 100 mV s^−1^ in the potential range of -50 to +600 mV for 20 cycles to activate and clean the catalyst surface electrochemically in an H_2_-saturated electrolyte^32^. Polarization curves were acquired using linear sweep voltammetry (LSV) at 1600 rpm and a scan rate of 1 mV s^−1^ to reduce capacitive currents (with iR correction). Electrochemical impedance spectroscopy (EIS) was conducted from 100 kHz to 100 mHz with a 5 mV amplitude at -40 mV. The uncompensated solution resistance (*R*_u_) for RDE measurements was determined before each measurement using the Get *R*_u_ function in the Gamry software, which estimates *R*_u_ from EIS. The measured *R*_u_ values were 5.5 ± 1.5 Ω, and the individually measured *R*_u_ value for each experiment was used for iR correction of the corresponding RDE polarization curve. For durability tests in RDE setup, chronoamperometry was performed at -50 mV and 1600 rpm over 80,000 s. For durability test with carbon paper substrate, the aRu3/WC_x_ catalyst was spray-coated onto carbon paper (Sigracet 39 BB, Fuel Cell Store) and subjected to a 100 h chronopotentiometry in 1 M KOH using a three-electrode setup. Tafel slopes were derived from HER polarization curves obtained by a multi-step chronopotentiometry approach, with HER overpotential recorded after holding for 20 s at intervals of log [*j* (mA cm^−2^)] by 0.05 to minimize the capacitive current effects.

### Computational details

#### Ru precursor solvation energy calculations

We optimized the geometries of the HPL and HPB Ru precursors using density functional theory (DFT) with the B3LYP-D3^80^ functional and the LACVP*^81^ basis set, as implemented in the Jaguar software package^82^. Based on the optimized geometries, we then conducted molecular dynamics (MD) simulations to calculate the solvation energies of the Ru precursors in poly(styrene) and poly(ethylene oxide), respectively. We carried out MD simulations using the large-scale atomistic modeling massively parallelized simulation (LAMMPS)^83^ code. The Ru precursors were treated as rigid bodies, while the interactions between the polymers and between the precursors and polymers were described using the DREIDING^84^ force field. Additionally, DFT-calculated electrostatic potential (ESP) charges were assigned to each atom. For the poly(styrene) (PS) and poly(ethylene oxide) (PEO) systems, we optimized the trimer conformations respectively and utilized the central monomer to determine the ESP charge of the polymer.

We prepared four systems to calculate the polymer and self-solvation energies for each Ru precursor;

i) a single Ru precursor molecule (HPL or HPB) in a simulation cell of 30 Å × 30 Å × 30 Å (Supplementary Fig. 1a), ii) a condensed phase of Ru precursor (HPL or HPB), consisting of 10 molecules in the simulation cell of 17.0 Å × 17.0 Å × 17.0 Å or 11.3 Å × 11.3 Å × 11.3 Å, respectively, (Supplementary Fig. 1b), iii) a single Ru precursor molecule solvated by either PEO or PS polymers (Supplementary Fig. 1c), and iv) a polymer melt with no Ru precursor (PEO or PS) (Supplementary Fig. 1d)

The ensemble average MD energies of above systems are labeled as ${E}_{{{{\rm{Ru}}}}}$, ${E}_{n\cdot {{{\rm{Ru}}}}}$, ${E}_{{{{\rm{Ru@polymer}}}}}$, and ${E}_{{{{\rm{polymer}}}}}$. Then, the polymer and self-solvation energies of the Ru precursor are respectively defined by

1 $$\Delta {E}_{{{{\rm{solv}}}}}={E}_{{{{\rm{Ru@polymer}}}}}-{E}_{{{{\rm{polymer}}}}}-{E}_{{{{\rm{Ru}}}}}$$

2 $$\Delta {E}_{{{{\rm{solv}}}}}^{{{{\rm{self}}}}}=\frac{{E}_{n\cdot {{{\rm{Ru}}}}}}{n}-{E}_{{{{\rm{Ru}}}}}$$

where n is the number of Ru precursors in the simulation cell for (2), i.e., n=10. Considering that the dispersion propensity of the Ru precursor in the polymer matrix is determined by the energetic competition between the polymer solvation and self-solvation, $\Delta {E}_{{{{\rm{disp}}}}}$ is defined by

3 $$\Delta {E}_{{{{\rm{disp}}}}}=\Delta {E}_{{{{\rm{solv}}}}}-\Delta {E}_{{{{\rm{solv}}}}}^{{{{\rm{self}}}}}$$

In our simulations, the polymer chain was modeled to have a chain length of *N* = 100. To efficiently sample the polymer conformations, we employed our recently developed threading subunits for polymers (TSP) method^85^. This method accelerates the otherwise sluggish equilibration process by pre-equilibrating the monomeric units to develop local solvation shells around the solutes, followed by connecting (threading) them into polymers. More details on this simulation method can be found in our previous publication^85^, and in Supplementary Fig. 1.

After constructing the initial structures for each of the four systems, we heated them from 100 K to 300 K over 100-ps in canonical (NVT) ensemble simulations using Nosé–Hoover thermostat^86^. This was followed by 3-ns isothermal-isobaric (NPT) ensemble simulations using the Parrinello–Rahman barostat^87^, and then 6-ns of NVT equilibration. The energies obtained by averaging the last 1-ns of the simulations were used for computing the solvation energies.

### Ru dispersion simulation on WC surface

DFT calculations for periodic systems were carried out using the Vienna ab initio simulation package^88^ (VASP). The core electrons were treated with the projector augmented wave (PAW) method, and the plane-wave cutoff energy was set to 500 eV. The revised Perdew–Burke–Ernzerhof (RPBE) functional^89^ was used as the exchange–correlation functional. Thresholds for the electronic and ionic convergences were set as 10^–5^ and 10^–4^ eV, respectively. We optimized the bulk structure of face-centered cubic (fcc) WC, using a 10 × 10 × 10 Monkhorst-Pack k-point mesh. Based on the fully optimized bulk structures, we constructed a four-layer 2 × 2 (100) slab model with a 20 Å vacuum layer in z-direction. For the slab model, the Brillouin zone was sampled using a Γ-centered 4 × 4 × 1 mesh. During the optimization of slab models, two bottommost layers were fixed, and a dipole correction was applied along the z-direction. This optimized WC surface conformation was then used in the subsequent MD simulations.

To accurately model the interatomic potential between Ru atoms, we employed the Embedded Atom Method (EAM)^90^ potential, which effectively accounts for many-body interactions. For the pairwise interactions between W, C, and Ru, we first adsorbed a single Ru atom on the WC (100) and performed DFT optimization to determine the equilibrium position. We then displaced the Ru atom along the z-direction and obtained the potential energy profile. The van der Waals parameters were fitted to align with the DFT-optimized minimum position and well depth (Supplementary Fig. 5a). The same procedure was applied to the WC (100) slab model with a carbon defect (Supplementary Fig. 5b).

Our parameter sets were further validated by comparing the bulk modulus of WC, the lattice parameters for the WC, and the cohesive energy of Ru (Supplementary Table 4). Our fitted parameters well reproduced the bulk modulus and lattice parameter of WC and cohesive energy of Ru within 1.8 % error. To calculate the bulk modulus of WC, small perturbations were applied to the optimized lattice parameters, with the volume changes of $\pm 1\%$ around the equilibrium structure. The resulting pressure change with respect to the volume change was then used to compute the bulk modulus (*K*) following Eq. (4).

4 $$K=-V\frac{{dP}}{{dV}}$$

The cohesive energy of Ru atoms was calculated from DFT and MD simulations using the following formula, where *n* is the number of atoms in bulk system:

5 $${E}_{{{{\rm{coh}}}}}={E}_{{{{\rm{atom}}}}}-\frac{{E}_{{{{\rm{bulk}}}}}}{n}$$

After setting the simulation parameters, we modeled the system with varying numbers of Ru atoms (# Ru: 1 ~ 37414) adsorbed on the WC surface. The DFT-optimized slab structures were used to construct supercells for MD simulations, with sizes of 4 ×4 (# Ru: 1 ~ 116), 8 ×8 (# Ru: 163 ~ 235), and 24 ×24 (# Ru: 321 ~ 37414) (Supplementary Table 5).

Each system was then simulated using the following simulation procedure:

i) Position spherical Ru NPs with varying numbers near the WC surface.
ii) NVT heating from 50 K to 1173 K for 200-ps with a 5-fs timestep.
iii) NVT equilibration for 5-ns at 1173 K.
iv) NVT production for 10-ns at 1173 K.

Using the last 5-ns of 10-ns trajectory, we analyzed the CNs of Ru atoms, by calculating the radial distribution function (RDF) and integrating up to the first minimum (3 Å) of the RDF peak. To evaluate the one-dimensional, two-dimensional nature of the Ru NPs, we examined the standard deviation of the z coordinate distribution of the Ru atoms over the last 5-ns period.

### Electrochemical reaction free energy calculation

To evaluate the water dissociation energy and hydrogen adsorption energy on different surfaces, the Gibbs free energy change (Δ*G*) was calculated using Eq. (6).

6 $$\Delta G=\Delta {E}_{{{{\rm{SCFE}}}}}+\Delta {E}_{{{{\rm{ZPE}}}}}-T\Delta S$$

Here, Δ*E*_SCFE_, Δ*E*_ZPE_, and *T*Δ*S* represent the differences in the DFT self-consistent-field energy, zero-point energy, and entropic contribution, respectively. The partial Hessian diagonalization method was used to evaluate Δ*E*_ZPE_ and the vibrational entropy of *T*Δ*S*, and the translational and rotational entropy contributions were included only for the molecules in the gas phase using particle-in-a-box and rigid-rotor partition functions, respectively. Using the computational hydrogen electrode (CHE) scheme, the chemical potential of a proton–electron pair was set to (0.5 Δ*G*_H2_ – *eU*_SHE_). Also, details of the cell sizes and *k*-point sampling for the various systems are provided in Supplementary Table 14.

### Support induced stabilization energy

To assess the relative stabilization of quasi-planar (2D) monolayer-like Ru islands with respect to compact (3D) Ru clusters on WC, the support-induced stabilization energy was evaluated according to Eq. (7)

7 $$\Delta {E}_{{{{\rm{stab}}}}}^{{{{\rm{X}}}}}={E[{{{{\rm{Ru}}}}}_{N}/{{{\rm{WC}}}}]}^{{{{\rm{X}}}}}-E\left[{{{\rm{WC}}}}\right]-E\left[{{{{\rm{Ru}}}}}_{N}\right],{{{\rm{X}}}}\in \left\{2D,3D\right\}$$

where ${{{{\rm{Ru}}}}}_{N}$ denotes a Ru cluster composed of *N* Ru atoms, $E\left[{{{{\rm{Ru}}}}}_{N}/{{{\rm{WC}}}}\right]$ denotes the total energy of ${{{{\rm{Ru}}}}}_{N}$ cluster of morphology X adsorbed on the WC support, $E\left[{{{\rm{WC}}}}\right]$ is the energy of the clean WC support, and $E\left[{{{{\rm{Ru}}}}}_{N}\right]$ is the energy of the isolated Ru_*N*_ cluster of the 3D morphology. All calculated energies include solvation corrections in water.

### STEM Z contrast image calculation

The HAADF-STEM Z-contrast images were simulated using the abTEM^91^ package in Python, based on the DFT relaxed geometries for the Ru_1_–Ru_6_ clusters and the MD-equilibrated structures for the Ru_55_ and Ru_236_ models. For comparison between single Ru atom and a W defect models, we constructed a WC slab with a thickness of 0.6 nm. For size-dependent STEM image comparison, we built models with WC slabs of ~ 20 Å thickness, with Ru clusters adsorbed on the top surface, and a 20 Å vacuum layer introduced along the beam direction to prevent spurious interactions between periodic images. The electron–sample interaction potential was constructed using the independent atom model (IAM) with a real-space sampling of 0.03 Å and a slice thickness of 1.0 Å. A convergent electron probe with an accelerating voltage of 200 kV and a convergence semi-angle of 25 mrad was employed. The HAADF detector was modeled with inner and outer collection angles of 70 and 200 mrad, respectively.

### Surface-enhanced infrared absorption spectroscopy (SEIRAS)

In situ attenuated total reflection-SEIRAS (ATR-SEIRAS) measurements were conducted using a catalyst/Au-coated Si prism working electrode (Veemex, incident angle of 60° and 2 cm in diameter), with a three-electrode spectroelectrochemical cell comprising two compartments. The Au thin film, prepared on the Si prism through electrodeless plating, served as the active SEIRAS substrate, with the catalyst ink spray-coated onto this surface^92^. Catalyst inks were prepared by dispersing 0.825 mg of aRu3/WC_x_ or 0.425 mg of Pt/C in 7.5 µL of Nafion solution and 200 µL of IPA. Pt wire functioned as the counter electrode, while an Ag/AgCl (Basi, 3 M NaCl) electrode served as the reference electrode. The spectroelectrochemical cell was integrated into a Fourier transform infrared (FT-IR) spectrophotometer (VERTEX 80 v, Bruker) equipped with a mercury cadmium telluride detector and a variable angle specular reflectance accessory (VeemaxIII, Pike Technologies). Spectroscopic data were collected in absorbance mode with a spectral resolution of 4 cm^−1^, using Ar-saturated 0.1 M KOH solution as an electrolyte. Background spectra were obtained at open circuit voltage (OCV).

### In situ X-ray absorption spectroscopy (XAS) measurement

In situ XAS spectra were collected at the 8 C Nano XAFS beamline at Pohang Accelerator Laboratory (PAL), Korea. The working electrode was prepared by spray-coating aRu3/WC_x_ catalyst ink onto carbon paper with targeted loading (Sigracet 39 BB, wet-proofed, Fuel Cell Store). The catalyst ink was prepared by dispersing 50 mg of aRu3/WC_x_ and 5 mg of Vulcan XC 72 R in 800 μL of DI water, 800 μL of ethanol, 1600 μL of isopropanol, and 116 μL of HQPC-TMA-2.4 (5 wt% ethanol) solution, with sonication for 2 h. In situ XAS measurements were conducted in a three-electrode spectroelectrochemical flow cell using Ar-saturated 1 M KOH electrolyte at a flow rate of 0.1 mL min^−1^. A graphite rod and Hg/HgO electrode were employed as the counter and reference electrodes, respectively. XANES spectra were obtained during chronoamperometry in both transmission and fluorescence modes. The potential was sequentially set to OCV, −10, −20, −30, −40, and −50 mV (vs. RHE) to investigate structural and electronic changes during the alkaline HER, with each potential maintained for at least 10 min to ensure steady-state conditions. Data processing was conducted using Athena from the Demeter software package.

### In situ Raman measurement

In situ Raman spectra were acquired using a confocal Raman microscope (Ramantouch, Nanophoton) at the Korea Basic Science Institute (KBSI, QM12), Daejeon. A 532 nm laser with an excitation power of 38.0 mW was employed. The crystalline silicon peak at 520 cm^−1^ was used as an internal reference for wavenumber calibration. A diffraction grating with 600 grooves mm^−1^ and a LU Plan Fluor 20× objective lens (Nikon, NA = 0.45) were used, yielding a laser spot size of approximately 1 μm on the electrode surface. The scattered light was collected using a CCD detector (1340 × 400 pixels) operated in low-noise mode at a readout speed of 2 MHz, with the CCD temperature maintained at −70 °C. For electrochemical in situ Raman measurement, a conventional three-electrode cell was employed, consisting of an aRu3/WC_x_-based working electrode, a Pt wire counter electrode, and a Hg/HgO reference electrode. The electrolyte was 1 M KOH. The Raman system was coupled with an SP2 potentiostat. Raman spectra were continuously recorded during potential sweeps from the OCP to the HER region to monitor structural and interfacial changes in real time.

### AEMWE single-cell assembly and electrochemical measurement

Homemade anion-exchange membrane (HQPC-TMA-2.4 AEM, 30 μm) and ionomer dispersion (5 wt% HQPC-TMA-2.4 in isopropanol aqueous solution) were used for AEMWE single-cell measurements^44^. Before cell assembly, the membrane was pre-treated in 1 M KOH for 2 h, with the solution refreshed every 0.5 h. Porous transport electrodes (PTEs) with an active area of 5 cm^2^ were fabricated using a spray-coating method. For the anode PTE, a homemade NiFe alloy was used as the OER electrocatalyst^38^. The anode catalyst ink was prepared by mixing 50.0 mg of the catalyst powder with 100.0 mg of 5 wt% ionomer dispersion in a solution containing 0.3 mL of DI water and 1.5 mL of isopropanol, then homogenizing the mixture in an ultrasonic bath for 0.5 h. The prepared ink was then sprayed onto the 300 μm-thick Ni fiber paper (Dioxide Materials) to achieve an active material loading of 1.0 mg_metal_ cm^−2^. For the cathode PTE, synthesized aRu*N*/WC_x_ were used as the HER electrocatalysts. The cathode catalyst ink was prepared by mixing 50.0 mg of the catalyst with 6.5 mg of Vulcan XC 72 R and 100.0 mg of 5 wt% ionomer dispersion in a solution containing 0.3 mL of DI water, 0.9 mL of ethanol, and 0.9 mL of isopropanol. After ultrasonicating the ink for 0.5 h, the prepared ink was sprayed onto a carbon paper with a microporous layer (JNT-30-A3, JNTG) to achieve an active material loading of 0.1 mg_Ru_ cm^−2^. For benchmarking, cathode PTEs using commercial Pt/C (20 wt% Pt on carbon black, HiSPECH 3000, Johnson Matthey Co.) were also prepared using the same fabrication process, with an active material loading of 0.4 mg_Pt_ cm^−2^ and ionomer-to-catalyst weight ratio of 0.3. After the catalyst coating process, the prepared PTEs were dried for 12 h to evaporate the residual solvents in the catalyst layer.

A custom-made cell was used for AEMWE performance evaluation. The membrane-electrode assembly was fabricated by sandwiching the membrane between the anode and cathode PTEs. The MEA was then sealed with two PTFE-film gaskets, and the cell was assembled at a torque of 7.35 N·m. The resulting cells were integrated into an AEMWE test station (CNL Energy), and the electrochemical characterization was conducted using a potentiostat (HCP-803, Bio-Logic). After preconditioning the cell by circulating a 1 M KOH solution at 60 °C and a flow rate of 10 mL min^−1^ for 1 h, AEMWE performance was measured at operating *T* of 60 and 80 °C. The KOH solution was preheated to match the cell temperature. *I*–*V* polarization curves were recorded through the LSV within the voltage range of 1.3–2.0 V with a scan rate of 5 mV s^−1^. Corresponding EIS measurements were performed at 1.0 A cm^−2^ under frequencies ranging from 0.1 Hz to 100 kHz with amplitudes of 8% of the operating *j*. The in situ durability test was performed under dry cathode conditions with a constant *j* of 1.0 A cm^−2^ and a *T* of 60 °C for 500 h. After the short-term (100 h) and long-term (500 h) durability test, the end-of-test (EOT) performance was measured using a fresh electrolyte in the same manner as the beginning-of-test (BOT) performance for degradation analysis.

To evaluate operational stability under practical intermittent AEMWE conditions, start-stop cycling tests were additionally performed by alternating between galvanostatic operation at 1.0 A cm^−2^ and OCV every 1 h for a total operation time of 100 h. After the intermittent operation test, distilled water in the cathode-side trap bottle (1 L) was recirculated through the cathode compartment for 1 h to collect dissolved metal species remaining in the system. The collected solution was subsequently analyzed using ICP-MS to quantify dissolved Ru, W, and Pt species.

### Faradaic efficiency calculation

Faradaic efficiency (FE) was calculated by comparing the experimentally measured hydrogen production with the theoretical hydrogen yield based on Faraday’s law following Eq. (8):

8 $${{{\rm{FE}}}}\left(\%\right)=\frac{{v}_{{{{\rm{H}}}}2,{{{\rm{measured}}}}}}{{v}_{{{{\rm{H}}}}2,{{{\rm{theoretical}}}}}}$$

For experimental quantification (*v*_H2, measured_), the cathode vent stream was passed through a trap bottle containing 500 mL of distilled water and subsequently through a gas–liquid separator maintained below 0 °C using a recirculating chiller, thereby removing residual water vapor from the gas stream. The resulting dry gas stream was then measured using a mass-flow meter calibrated for H_2_ at 0 °C and 1 atm.

The theoretical volume of H_2_ (*v*_H2, theoretical_) can be calculated using Eq. (9):

9 $${v}_{{{{\rm{H}}}}2,{{{\rm{theoretical}}}}}=\frac{I\times t\times {V}_{m}}{n\times F}$$

where the *I* is the current obtained, *t* is time of experiment, *V*_m_ is the molar volume of H_2_ at 0 °C and 1 atm. The number of electrons required for producing 1 mol of H_2_ is (*n* = 2), and *F* is Faraday’s constant (96,485 C mol^−1^).

### S-number calculation

The S-number calculation was performed based on the hydrogen generated at the cathode during 100 h AEMWE operation under dry cathode condition, in combination with the quantification of dissolved noble metals following Eq. (10).

10 $${{{\rm{S}}}}{{{\rm{\hbox{-}}}}}{{{\rm{number}}}}=\frac{{n}_{{{{\rm{H}}}}2}}{{n}_{{{{\rm{metal}}}}}}$$

where *n*_H2_ is the molar amount of total hydrogen generated in a given time (calculated from the total charge of the stability test multiplied by the Faradaic efficiency) and *n*_metal_ is the total metals dissolved in the electrolyte.

For the quantification of dissolved noble metals (*n*_metal_), the cathode vent stream was first passed through a trap bottle containing 500 mL of distilled water, which served to capture the small fraction of dissolved noble metals entrained in the effluent during AEMWE operation.

After the 100 h operation, in order to collect any residual dissolved noble metal ions remaining inside the cathode compartment, the distilled water from the trap bottle was recirculated through the cathode for 1 h. This procedure ensured complete leaching of the residual noble metal ions into the solution. The collected solution was subsequently analyzed by ICP–MS, and the measured concentration was used to determine the total amount of noble metals dissolved during the test.

### Overpotential breakdown

Polarization curves for overpotential breakdown analysis were obtained using multistep chronopotentiometry (CP) with current densities ranging from 0.02 to 5.0 A cm^−2^. The applied current density was sequentially increased from 0.02 to 0.25 A cm^−2^ (0.02, 0.04, 0.05, 0.075, 0.10, 0.15, 0.20, and 0.25 A cm^−2^), followed by increments of 0.5 A cm^−2^ up to 5.0 A cm^−2^. Each current step was maintained for 1 min.

The ohmic resistance at each current density was determined by electrochemical impedance spectroscopy (EIS), and the corresponding iR-corrected polarization curves were obtained by subtracting the ohmic contribution. The kinetic overpotential was extracted from the Tafel region (0.02−0.2 A cm^−2^), where mass transport effects are negligible. The residual overpotential was calculated by subtracting the ohmic and kinetic contributions from the total cell voltage. The residual overpotential is mainly associated with mass transport limitations, including gas-bubble accumulation and reactant/product transport within the catalyst layer.

### In situ dynamic bubble behavior of catalyst layers

Real-time imaging of hydrogen bubble evolution was conducted under alkaline HER conditions using a custom-built transparent electrochemical cell with a three-electrode configuration. The working electrodes were prepared by spray coating catalyst inks onto non-porous Grafoil substrates with a noble metal loading of 0.10 mg_mass_ cm^−2^. Bubble evolution behavior was monitored under galvanostatic operation at 10 mA cm^−2^ using a digital optical microscope positioned perpendicular to the electrode surface. Time-resolved optical images were recorded to analyze bubble nucleation, growth, detachment, and surface coverage.

### Statistical analysis

Unless otherwise stated, electrochemical polarization curves and derived electrochemical values are presented using one representative dataset. For AEMWE single-cell measurements, repeated independent measurements were performed to check reproducibility, and the representative polarization curve and derived performance metrics were obtained from the dataset showing median performance among the repeated measurements. These representative values are reported without error bars. When repeated measurements are summarized statistically, the results are reported as mean ± standard deviation, with the number of independent measurements indicated in the corresponding figure caption.

## Supplementary information

Supplementary Information

Description of Additional Supplementary Files

Supplementary Data 1

Supplementary Movie 1

Supplementary Movie 2

Supplementary Movie 3

Supplementary Movie 4

Supplementary Movie 5

Supplementary Movie 6

Supplementary Movie 7

Supplementary Movie 8

Transparent Peer Review file

## Source data

Source Data

## Data Availability

Source data are provided with this paper. The source data generated in this study for the main figures are provided in the Source Data file for the main figures. The source data generated in this study for the Supplementary Figs. are provided in the Source Data file for the supplementary information. The structural models used for DFT calculations and MD simulations are provided in Supplementary Data 1. The remaining data supporting the findings of this study are available within the article and the Supplementary Information. Additional data supporting the findings of this study are available from the corresponding authors upon request. Source data are provided with this paper.
