# Supplementary material for: Stabilizing ruthenium atom layer neighbors through molecular and metal support interactions for water electrolyzers
Source: Nat Commun. 2026 Sep 22;17:11021. doi: 10.1038/s41467-026-78037-3 (PMC13687079; doi:10.1038/s41467-026-78037-3)
Supplement: Supplementary file 1 [file 41467_2026_78037_MOESM12_ESM.pdf]

nature portfolio

Peer Review File

Stabilizing Ruthenium Atom Layer Neighbors through Molecular and Metal Support Interactions for Water Electrolyzers

Corresponding Author: Professor Jinwoo Lee

This file contains all reviewer reports in order by version, followed by all author rebuttals in order by version.

Version 0:

Reviewer comments:

Reviewer #1

(Remarks to the Author)
The manuscript presents a sophisticated synthesis strategy utilizing block copolymer precursors and strong metal-support interactions (SMSI) to stabilize Ruthenium atom-layer neighbors (ALNs) on WCx supports. The reported electrocatalytic performance for the HER reaction in alkaline media is impressive, particularly the potential utility in AEMWE. However, despite the promising data and the huge load of work, the manuscript in its current form falls short of the rigorous standards required for publication in Nature Communications due to several fundamental concerns. Therefore, a major revision is required.
Major comments:
1. The EXAFS fitting reports a Ru-W bond length of ~2.84 Å, which is significantly longer than the typical Ru-W bond in bulk alloys (~2.72 Å). The authors use this to rule out alloying. 2.84 Å distance must suggests a distinct specific adsorption geometry, possibly Ru atoms sitting on the hollow sites of the W-terminated surface or bridging sites. Providing a specific DFT-optimized structural model that precisely matches this 2.84 Å bond length would strengthen the structural claim of the sample.
2. The EXAFS data shows a Ru-C/O coordination number of ~4 with a bond length of ~2.10 Å. The distinction between Ru-C (from the WC support) and Ru-C (from residual polymer carbon or surface contamination) is critical. Since the synthesis involves carbonization of a polymeric-precursor, how can the authors indentify the Ru is primarily anchored to the WC lattice carbon rather than being encapsulated in an amorphous carbon shell (graphitic layers)?
3. The authors admit that HAADF-STEM Z-contrast is difficult to distinguish Ru due to the W background and the 2D atom-layer nature is largely inferred from EXAFS Ru-Ru CN (< 2.5) and simulations. However, a CN of ~2.5 could also correspond to a very small 3D tetrahedral cluster. Therefore, further evidence should be provided.
4. While MD simulation is convincing, direct experimental evidence of this partitioning in the polymer melt before pyrolysis is missing. Investigating the polymer-precursor interaction via TG-DSC would experimentally validate the solvation energy calculations and strengthen the synthesis mechanism.
5. The manuscript highlights superior durability against Fe/Ni deposition compared to Pt/C but the mechanism is unclear. Clarifying where the impurities go (using high-res EDS mapping or other techniques) would reveal the true mechanism of this durability.
6. The authors propose a dual-site mechanism (*H on Ru, *OH on WC). However, whether *H adsorbed on Ru spill over to the WC support is unclear. This could dynamically alter the active site density. The authors should comment on whether this pathway exists and contributes to the high activity.
Minor comments:
1. Please revise the layout of the figures in the main manuscript to enhance the readability, figures in the current form are too crowded.
2. Please optimize Figure 2 e and f by deleting the contour lines. The spectrums in their current forms are too hard to read and identify.

Reviewer #2

(Remarks to the Author)
The recommendation is to accept the manuscript with minor revisions. The paper extensively describes a novel approach for

realizing HER catalyst materials for AEMWE by widening the concept of single-atom-catalysts to small ensembles of atoms forming Ru-based atom-layer-neighbours dispersed onto WCX substrates. Authors in-depth combine computational methods, microscopy, and x-ray-based investigation techniques to completely characterise and optimise the fabrication process as well as to fully characterise the structural and chemical catalyst composition. Furthermore, catalyst activity and stability are investigated and the novel catalyst proposed is compared with state of art catalyst materials, showing remarkable performances in terms of both catalyst activity and stability. The proposed research is meaningful and it is in line with the editorial line of the journal. Nonetheless, before considering the paper for publication, the following points should be refined:

1. Acronyms should be better defined, capitalizing the proper letters once the acronym is defined.
2. While acronyms of techniques are commonly found and known by the readers, the acronym NM (Noble Metal) sounds odd; to improve manuscript readability, Authors are suggested to replace it with its textual version.
3. At page 3 of the pdf file of the manuscript there is a remarkable logical jump in between the first and the second paragraph. Authors might consider improving this feature, but this advice can be considered optional.
4. In Figure 1a, the first image on the left is hardly readable, as bonds between Ru nanoparticle surface and H atoms are barely distinguishable from the background, and “top”, “bridge”, and “hollow” configurations cannot be properly identified. Image outlook should be improved; moreover, legend arrangement should be improved, if possible.
5. At page 15 of the manuscript, reference document for the DOE target must be provided.
6. At page 16, section “references” should be moved at the end of the manuscript.
7. At page 22, basic SAXS experimental setup details should be provided, such as: energy used, calibrant, and sample to detector distance.
8. In SI, as did for XPS and XAS, SAXS data analysis should be detailed. In fact, SAXS patterns are represented as Kratky plots (I(q) * q2 vs q) instead of standard scattering pattern (I(q) vs q), but this choice has not been explained. Moreover, Supplementary Figure 13 is show without being fully commented. Basic information, such as: (i) what is represented in the area highlighted by pale yellow, (ii) how different sample porosity reflects on scattering patterns, and (iii) why samples show different evolution at the higher q-values in Kratky plots and why the information in this part of the graph can be neglected, should be qualitatively introduced at least. In these regards, Authors should also apply the same considerations when commenting Supplementary Figure 18, where Kratky plots are directly compared with samples pore size distribution). Authors should comment why SAXS analyses were made via model-free approach instead of using an analytical model for retrieve pore size distribution.

Reviewer #3

(Remarks to the Author)
This manuscript reports a “Ru atomic-layered nanonetwork (ALN)” platform supported on WCx for alkaline HER and AEMWE cathodes. The authors claim that a thermodynamic stabilization strategy enables size-tunable Ru ensembles with high utilization, and that synergistic dual-site catalysis (Ru sites for *H adsorption and WCx for *OH adsorption) improves interfacial water structure and kinetics. The topic is timely and relevant, and the electrochemical and device-level performances appear competitive. However, several key claims (especially regarding structural assignment of Ru ALNs and the interpretation of OH enrichment or gap water) rely on indirect evidence and potentially over-interpreted spectroscopy. Additional validation and more rigorous control experiments are needed before the conclusions can be fully supported. Addressing the following suggestions will render the manuscript suitable for publication in this journal.

1.The manuscript claims that Ru ALNs represent an intermediate morphology between SACs and nanoparticles. However, the Ru–Ru coordination number (CN = 1.5–2.5) derived from EXAFS fitting alone is not sufficient to uniquely support a “layered adjacent” configuration. Such CN values could also arise from a statistical mixture of dimers/trimers and ultrasmall 3D Ru clusters. Can the authors provide direct and statistically meaningful structural evidence (e.g., interatomic distance histogram analysis from a large population of HAADF-STEM images, 4D-STEM, iDPC-STEM, or electron tomography) to demonstrate that Ru species indeed form a quasi-2D adjacent/layered ensemble rather than 3D stacked clusters?
2.Given the strong scattering contribution from the WCx support and the high-temperature reduction process, how can the authors exclude that the Ru–Ru EXAFS contribution originates from residual RuOx precursor or incompletely dispersed Ru species? It would be important to include reference fitting/LCF analysis against RuO_2 and Ru metal standards, and to explicitly discuss whether Ru–O contributions are present or suppressed (Fig.2f).
3.Does this high activity primarily stem from the electronic neighbor effect between Ru-Ru atoms, or from the strong modulation of the Ru electronic structure by the WCx support? We recommend that the authors supplement their work with a set of control experiments: synthesize Ru structures with similar coordination numbers on inert supports (e.g., carbon) or synthesize Ru nanoparticles on the same support. Employ DFT calculations to explicitly determine the relative contributions of the support effect and the spatial proximity effect to the hydrogen adsorption free energy change (ΔGH^*).
4.Under high current density operation, evaluating the Faraday efficiency of hydrogen production is crucial. It is recommended that the author employ gas chromatography (GC) or the displacement gas collection method to monitor hydrogen production at the AEMWE outlet in real time and calculate Faraday efficiency.
5.Under ultra-high current densities, bubble coverage and gas transport limitations often dominate the apparent cell voltage and can obscure intrinsic catalytic activity. Can the authors clarify whether the Ru ALN morphology leads to any measurable difference in gas bubble behavior compared to conventional Ru nanoparticles (e.g., bubble detachment frequency, bubble size distribution, or gas coverage)? If possible, please provide high-speed visualization or contact angle measurements, as well as iR-compensated polarization curves or EIS analysis to support that the improved performance is not primarily governed by bubble shielding or mass transport effects.
6.Is this synthetic strategy universally applicable to other platinum group metals (e.g., Ir, Pt) or non-precious metals (e.g., Ni, Co, Mo)? Furthermore, given the high molecular weight of BCP, how can the uniformity of the Ru ALN structure be ensured during carbonization without local collapse?

7.How does the carrier tolerate high potentials in an alkaline environment? Could oxidation occur, leading to Ru ALN detachment? Please supplement with ICP-MS analysis data of the electrolyte following accelerated degradation testing (ADT) to confirm the dissolution loss rates of Ru and W elements under simulated real-world conditions (including start-stop cycles).
8.Some figures (e.g., Fig.2) lack sufficient “white space” between sub-panels, leading to a cluttered appearance. I recommend re-aligning these panels to a standard grid to improve the reader's navigation through the structural characterization data. In Fig.4, the in-situ Raman and ATR-SEIRAS waterfall plots are currently overcrowded. Using a consistent color gradient for potential increments and providing difference spectra would significantly enhance the clarity of the mechanistic insights. The figures appear cluttered and lack the professional polish required for clear communication of complex data.

Version 1:

Reviewer comments:

Reviewer #1

(Remarks to the Author)
The authors have addressed all the concerns raised by the reviewers in a satisfactory manner. I have no further comments and recommend acceptance of the manuscript in its current form.

Reviewer #2

(Remarks to the Author)
After the revision process, paper quality highly improved and the evidenced lacks were correctly addressed. The manuscript can be considered suitable for publication.

Reviewer #3

(Remarks to the Author)
The authors have responded point-by-point to all comments and completed the required supplementary tests and analyses. All issues have been properly resolved, and the revised version is acceptable for publication.

Open Access This Peer Review File is licensed under a Creative Commons Attribution 4.0 International License, which permits use, sharing, adaptation, distribution and reproduction in any medium or format, as long as you give appropriate credit to the original author(s) and the source, provide a link to the Creative Commons license, and indicate if changes were made.
In cases where reviewers are anonymous, credit should be given to 'Anonymous Referee' and the source.
The images or other third party material in this Peer Review File are included in the article’s Creative Commons license, unless indicated otherwise in a credit line to the material. If material is not included in the article’s Creative Commons license and your intended use is not permitted by statutory regulation or exceeds the permitted use, you will need to obtain permission directly from the copyright holder.

To view a copy of this license, visit https://creativecommons.org/licenses/by/4.0/

Reviewer #1 (Remarks to the Author):

The manuscript presents a sophisticated synthesis strategy utilizing block copolymer precursors and strong metal-support interactions (SMSI) to stabilize Ruthenium atom-layer neighbors (ALNs) on WC_x supports. The reported electrocatalytic performance for the HER reaction in alkaline media is impressive, particularly the potential utility in AEMWE. However, despite the promising data and the huge load of work, the manuscript in its current form falls short of the rigorous standards required for publication in Nature Communications due to several fundamental concerns. Therefore, a major revision is required.

Reply

We sincerely thank the reviewer for the careful evaluation of our manuscript and for recognizing both the significance of the synthesis strategy and the promising electrocatalytic performance of the Ru ALN/WC_x catalysts. We also appreciate the reviewer's constructive comments regarding the structural assignment of the Ru ALNs, the origin of the Ru-C coordination environment, the possibility of three-dimensional Ru clusters, the experimental validation of the precursor-partitioning mechanism, the durability mechanism during AEMWE operation, and the proposed HER reaction pathway.

Following the reviewer's suggestions, we performed extensive additional theoretical, spectroscopic, microscopic, electrochemical, and thermal analyses to address these concerns. Specifically, we (i) carried out additional DFT calculations and coordination-environment analyses to examine the origin of the experimentally observed Ru-W coordination and long Ru-W bond length, (ii) performed high-resolution STEM-EDS analyses, additional DFT calculations, and control-sample studies to clarify the origin of the Ru-C coordination environment, (iii) investigated the possibility of small three-dimensional Ru clusters through DFT calculations, HAADF-STEM simulations, and experimentally verified nanocluster/nanoparticle control samples, (iv) experimentally validated the precursor-polymer interactions underlying the molecular interaction calculations and MD simulations using additional TG-DSC measurements, (v) clarified the durability mechanism through expanded post-durability analyses and discussion of Fe/Ni redeposition and catalyst structural stability, and (vi) further discussed the HER mechanism by evaluating the possible contribution of hydrogen spillover and comparing the electrochemical characteristics with previously reported spillover-based HER systems. In addition, the figures throughout the manuscript were substantially reorganized to improve readability and presentation clarity.

We believe that these additional investigations have significantly strengthened the mechanistic understanding, structural assignment, and experimental support for the conclusions presented in this work. We are grateful for the reviewer's insightful comments, which have substantially improved the quality and rigor of the manuscript.

Major comments:

1. The EXAFS fitting reports a Ru-W bond length of ~2.84 Å, which is significantly longer than the typical Ru-W bond in bulk alloys (~2.72 Å). The authors use this to rule out alloying. 2.84 Å distance must suggests a distinct specific adsorption geometry, possibly Ru atoms sitting on the hollow sites of the W-terminated surface or bridging sites. Providing a specific DFT-optimized structural model that precisely matches this 2.84 Å bond length would strengthen the structural claim of the sample.

Reply

We thank the reviewer for this insightful comment regarding the interpretation of the experimentally observed Ru-W bond length (~2.84 Å) and the importance of establishing a structurally consistent DFT model. We fully agree that identifying adsorption geometries capable of reproducing the experimentally observed Ru-W coordination environment would further strengthen the structural assignment of the Ru ALN/WC_x system.

As suggested by the reviewer, the experimentally observed Ru-W bond length, which is substantially longer than that of bulk Ru-W alloy structures (~2.72 Å), may in principle originate from specific surface adsorption geometries such as bridge-site or hollow-site adsorption on W-terminated WC surfaces. To examine this possibility, we additionally performed DFT calculations for Ru1–Ru6 ensembles on both γ-WC and δ-WC surfaces using various adsorption configurations, including bridge- and hollow-site structures (**Supplementary Figs. 77, 78**). For each ensemble size, the energetically most favorable adsorption structures were selected for further analyses.

The calculated average Ru-W bond lengths for the optimized Ru ensembles on γ-WC and δ-WC surfaces were consistently shorter than the experimentally fitted Ru-W bond length of the Ru ALN/WC_x samples (~2.84 Å) and instead were much closer to the Ru-W distances characteristic of alloy-like structures (~2.72 Å) (**Supplementary Fig. 79**). These results suggest that simple bridge- or hollow-site adsorption models on ideal W-terminated WC surfaces are unlikely to fully account for the experimentally observed long Ru-W bond length in the Ru ALN/WC_x system.

We further note that direct quantitative agreement between experimentally fitted EXAFS parameters and idealized DFT models is intrinsically challenging because the experimental samples contain structural heterogeneities that are difficult to fully capture in periodic DFT calculations, including nanocrystallinity of WC_x domains, local disorder, surface defects, nonstoichiometric carbide environments, surface oxidation/hydroxylation under ambient conditions, and distributions of Ru ensemble configurations. Therefore, rather than relying solely on exact agreement in absolute bond-length values, we focused on whether each structural model could reproduce the experimentally observed trend in Ru-W coordination as a function of Ru ensemble size.

To address this point, we compared the experimental Ru-W coordination number trends with DFT-derived Ru-W coordination environments using two cutoff distances (<2.75 Å and <2.90 Å) corresponding to alloy-like and Ru ALN-like Ru-W coordination regimes, respectively

(**Supplementary Fig. 80**). Notably, only the Ru ALN/WC_x model reproduced the experimentally observed coordination trend as the Ru ensemble size increased, whereas Ru ensembles adsorbed on γ-WC and δ-WC surfaces did not show meaningful agreement with the experimental tendency under either cutoff condition.

Although the exact origin of the experimentally observed Ru-W bond elongation requires further investigation, it may be associated with the unique surface-confined coordination environemtn of Ru ALNs, where Ru species are simultaneously stabilized through Ru-W and Ru-C interactions on nanocrystalline WC_x rather than within a lattice-constrained metallic alloy framework.

Taken together, these results indicate that the experimentally observed long Ru-W bond length (~2.84 Å) is difficult to explain using conventional surface-adsorbed Ru ensemble or alloy-like models alone. Instead, the combined experimental and theoretical analyses consistently support that the experimentally observed ~2.84 Å Ru-W coordination environment is more consistent with the Ru ALN/WC_x configuration proposed in this work than conventional alloy-like or simple surface-adsorbed Ru structures.

Revision

To further investigate the structural origin of the experimentally observed long Ru-W bond length (~2.84 Å), we additionally performed DFT calculations for Ru1-Ru6 ensembles adsorbed on γ-WC and δ-WC surfaces using various adsorption geometries, including bridge- and hollow-site configurations. We further compared the experimentally observed Ru-W coordination trends with those predicted by different structural models to determine whether conventional surface-adsorbed Ru ensembles could account for the experimentally observed EXAFS results. The additional analyses demonstrate that simple bridge- or hollow-site adsorption models on ideal WC surfaces do not reproduce the experimentally observed Ru-W bond length and coordination trends, whereas the proposed Ru ALN/WC_x model exhibit substantially better agreement with the experimental observations.

(Page 8-9 in the Supplementary Note 8 of the revised Supplementary Information)

To further examine the origin of the experimentally observed Ru-W bond length (~2.84 Å), additional DFT calculations were performed for Ru1-Ru6 ensembles adsorbed on W-terminated γ-WC and δ-WC surfaces using various adsorption geometries, including bridge- and hollow-site configurations (Supplementary Figs. 77, 78). For all ensemble sizes, the energetically most favorable structures exhibited average Ru-W bond lengths substantially shorter than the experimentally fitted value and instead closer to those expected for alloy-like Ru-W environments (Supplementary Fig. 79). These results suggest that simple surface-adsorbed Ru ensembles on ideal WC surfaces are unlikely to fully account for the experimentally observed long Ru-W bond length.

Because experimentally fitted EXAFS parameters represent averaged local environments in nanocrystalline WC_x containing defects, local disorder, nonstoichiometric carbide domains, and distributions of Ru ensemble configurations, direct quantitative agreement with idealized periodic DFT models is not necessarily expected. Therefore, rather than focusing solely on absolute bond-length agreement, we compared the experimental Ru-W coordination trends with those predicted by different structural models. Notably, only the Ru ALN/WC_x model reproduced the experimentally observed evolution of Ru-W coordination with increasing Ru ensemble size, whereas Ru ensembles adsorbed on γ-WC and δ-WC surfaces did not exhibit meaningful agreement with the experimental trend (Supplementary Fig. 80). These results further support that the experimentally observed ~2.84 Å Ru-W coordination environment is more consistent with the Ru ALN/WC_x configuration than with conventional alloy-like or simple surface-adsorbed Ru structures. The elongated Ru-W bond length may originate from the unique surface-confined coordination environment of Ru ALNs, where Ru species are simultaneously stabilized through Ru-W and Ru-C interactions on nanocrystalline WC_x rather than within a lattice-constrained metallic alloy framework.

To provide direct theoretical comparisons between the proposed Ru ALN/WC_x structure and conventional surface-adsorbed Ru ensemble models, we additionally included DFT analyses of adsorption-site stability, optimized Ru ensemble structures on γ-WC and δ-WC surfaces, and corresponding Ru-W bond-length comparisons with experimentally fitted values.

(Page 95-97 in the revised Supplementary Information)

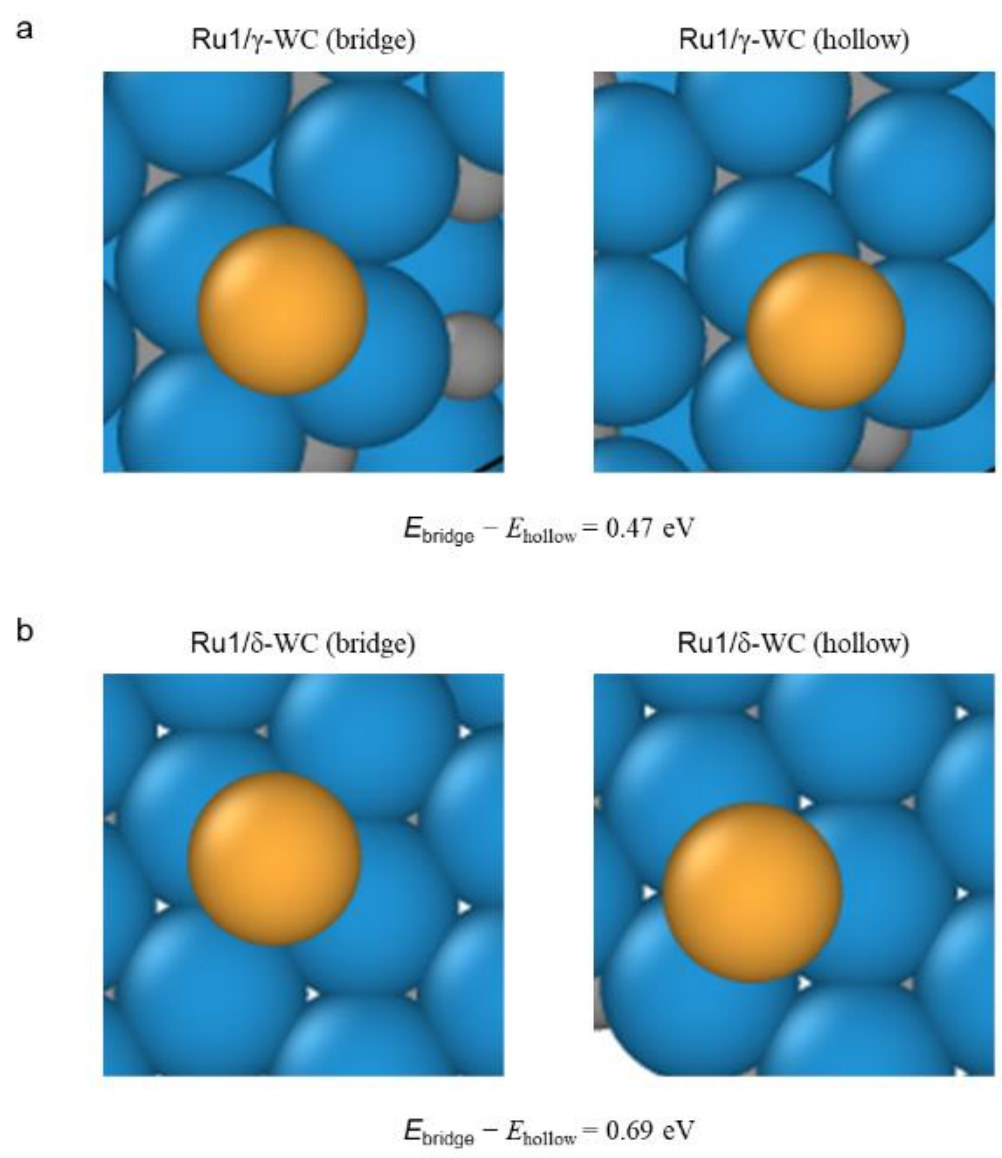

Supplementary Fig. 77 | Energetic preference of Ru single atoms on γ-WC or δ-WC surfaces. a-b, DFT-optimized structures of Ru single atoms adsorbed at bridge and hollow sites on (**a**) γ-WC, and (**b**) δ-WC supports, together with the corresponding free energy differences ($E_{bridge} - E_{hollow}$).

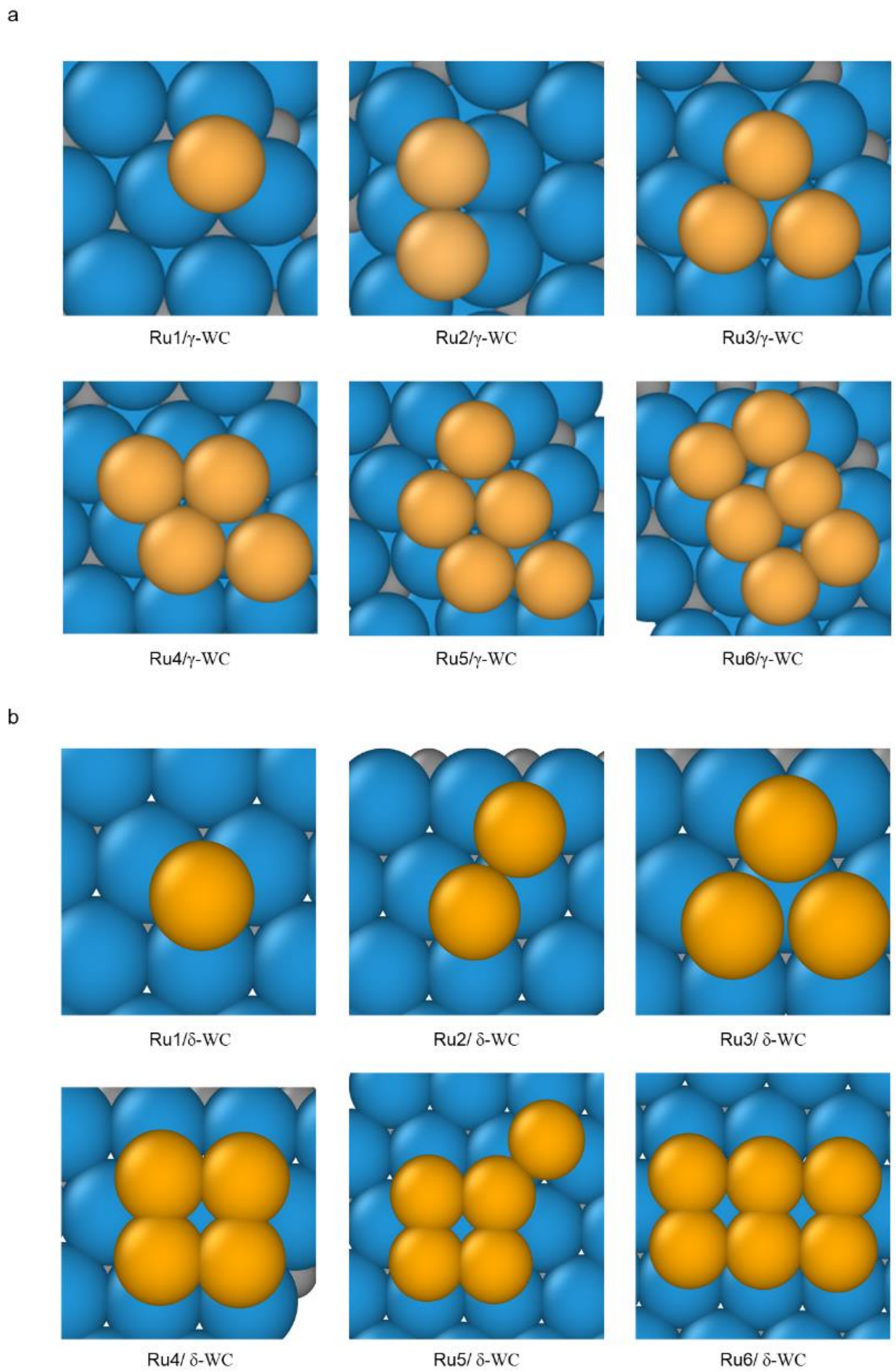

Supplementary Fig. 78 | DFT-optimized structures of Ru ensemble structures on γ-WC or δ-WC surfaces. a-b, DFT-optimized structures of Ru1, Ru2, Ru3, Ru4, Ru5, and Ru6 ensembles adsorbed on (**a**) γ-WC, and (**b**) δ-WC supports. Representative adsorption-site stability comparisons between bridge and hollow configurations are provided for Ru single atoms in Supplementary Fig. R1_1_1.

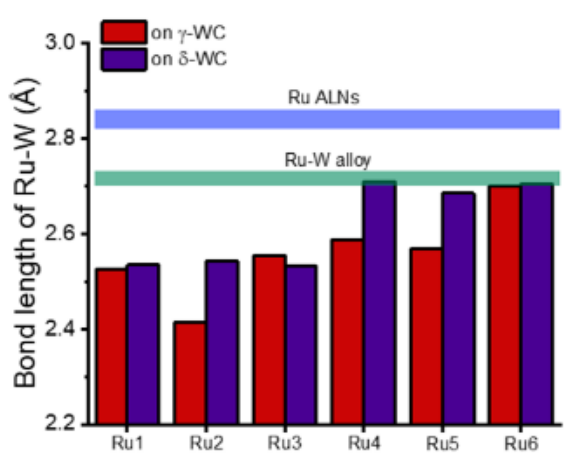

Supplementary Fig. 79 | Comparison of Ru-W coordination environments between DFT models and experimentally fitted structures. Average Ru-W bond lengths obtained from DFT-optimized Ru1, Ru2, Ru3, Ru4, Ru5, and Ru6 ensembles on γ-WC and δ-WC supports. The blue shaded region represents the experimentally fitted Ru-W bond length range for Ru ALN/WC_x samples (aRuN/WC_x), whereas the green shaded region corresponds to Ru-W alloy-like samples (a$Ru5_p$/WO_x, a$Ru5_p$/WC_x, Ru_p/WC_x_Ar, and Ru/WC_x_Ar).

To further evaluate whether different structural models can produce the experimentally observed Ru-W coordination evolution with increasing Ru ensemble size, we additionally compared the EXAFS-derived Ru-W coordination numbers with DFT-calculated Ru-W coordination environments for Ru ALN/WC_x, γ-WC supported Ru ensembles, and δ-WC supported Ru ensembles.

(Page 98 in the revised Supplementary Information)

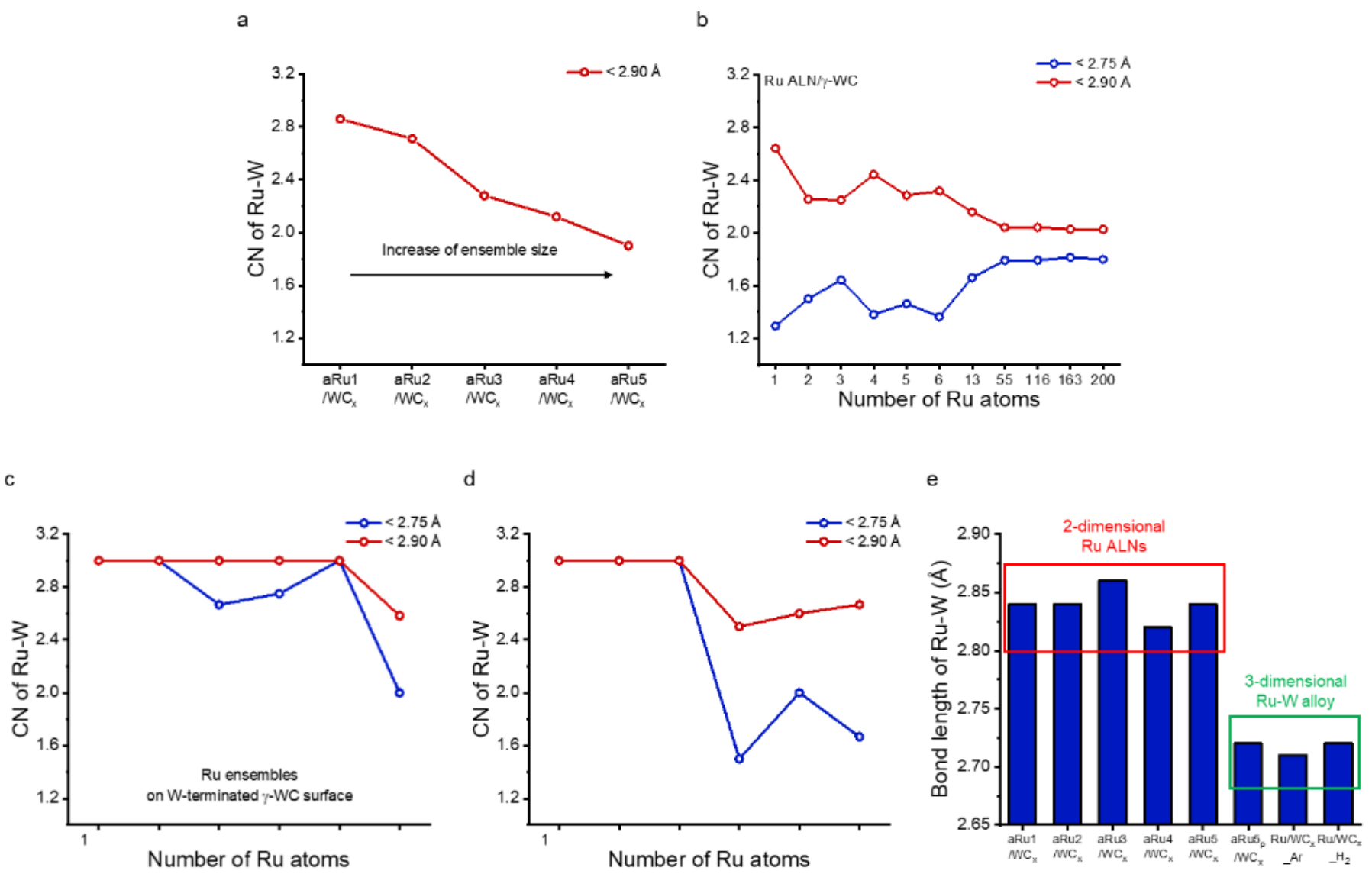

Supplementary Fig. 80 | Comparison between experimental and theoretical Ru-W coordination environments supporting the Ru ALN structure. a, Experimental Ru-W CNs obtained from EXAFS fitting for aRuN/WC_x (N = 1-5). **b-d,** DFT-calculated Ru-W CNs for (**b**) Ru ALN/WC_x models, (**c**) Ru ensembles on W-terminated γ-WC, and (d) Ru ensembles on W-terminated δ-WC, determined using cutoff distances of <2.75 Å (blue) and <2.90 Å (red). **e,** Comparison of experimentally fitted average Ru-W bond lengths. Samples assigned to Ru ALN/WC_x structures (red box) exhibited longer Ru-W bond lengths (~2.84 Å), whereas Ru-W alloy-like samples (green box) showed shorter Ru-W bond lengths (~2.70 Å).

2. The EXAFS data shows a Ru-C/O coordination number of ~4 with a bond length of ~2.10 Å. The distinction between Ru-C (from the WC support) and Ru-C (from residual polymer carbon or surface contamination) is critical. Since the synthesis involves carbonization of a polymeric-precursor, how can the authors identify the Ru is primarily anchored to the WC lattice carbon rather than being encapsulated in an amorphous carbon shell (graphitic layers)?

Reply

We thank the reviewer for this insightful comment regarding the origin of the Ru-C coordination observed by EXAFS and the possibility that Ru species may be stabilized by polymer-derived carbon species or graphitic carbon shells rather than being directly anchored on WC_x supports. We fully agree that distinguishing these possibilities is important for correctly identifying the local coordination environment of the Ru ALNs.

First, the EXAFS results provide direct evidence that the Ru species are closely associated with WC_x supports rather than being predominantly stabilized by carbonaceous environments. In all aRuN/WC_x samples, substantial Ru-W coordination was observed simultaneously with Ru-C coordination with Ru-W coordination numbers (CNs) exceeding unity and Ru-W bond lengths of approximately 2.84 Å (**Supplementary Tables 8, 9**). If the Ru species were predominantly stabilized by polymer-derived carbon shells or graphitic layers, direct Ru-W coordination would be expected to be negligible. Moreover, the experimentally observed Ru-W distance corresponds to a direct local coordination environment between Ru and W atoms. The presence of one or more graphitic carbon layers between Ru species and WC_x domains would be expected to result in substantially longer Ru-W distances or the absence of measurable Ru-W coordination. Therefore, the simultaneous observation of Ru-C and R-W coordination strongly supports direct stabilization of Ru species at the WC_x surface rather than preferential anchoring on polymer-derived carbon species, surface carbon contaminants, or encapsulation within graphitic carbon shells.

To further verify the spatial distribution of Ru species, we additionally performed high-resolution STEM-EDS analyses focusing separately on WC_x particles and carbon-rich regions (**Supplementary Figs. 71-74**). In all examined regions, the EDS elemental mappings showed that Ru signals spatially overlapped with W-containing domains. Furthermore, line-scan analyses consistently revealed that Ru signals were selectively observed in regions where W signals were present, whereas the Ru intensity became negligible in carbon-rich regions regardless of whether the measurements were performed on WC_x particles or carbon-rich areas. These observations indicate that Ru species are preferentially associated with WC_x domains rather than carbonaceous regions, providing direct microscopic evidence that Ru stabilization occurs predominantly on WC_x supports.

To further examine whether the observed Ru-C coordination could originate primarily from carbonaceous environments, we additionally performed DFT calculations for Ru3 and Ru4 ensembles adsorbed on graphene surfaces and confined between graphene layers, representing Ru species stabilized on graphitic carbon and encapsulated within graphitic shells, respectively

(**Supplementary Fig. 75**). Experimentally, the Ru ALN/WC_x samples exhibit elongated Ru-Ru bond lengths (~2.74 Å) compared with bulk metallic Ru (~2.68 Å), indicating a unique coordination environment associated with Ru ALN stabilization on WC_x. Consistent with this tendency, Ru ensembles stabilized on γ-WC also exhibited elongated Ru-Ru bond lengths relative to bulk Ru (**Supplementary Figs. 76, 78**). In contrast, both carbon-supported and graphitic shell-confined Ru ensembles exhibited substantially shorter Ru-Ru bond lengths than those observed experimentally for the Ru ALN WC_x samples (**Supplementary Figs. 75, 76**). These results indicate that carbonaceous environments cannot reproduce the experimentally observed Ru-Ru bond length elongation characteristic of the Ru ALN/WC_x system, whereas the γ-WC supported Ru ensembles exhibit a trend consistent with the experimental observations.

This tendency was further supported experimentally using Ru atomic clusters stabilized on N-functionalized carbon (aRu/NC) (**Supplementary Figs. 111, 112**). Although aRu/NC exhibited a Ru-Ru coordination number (CN = 1.92) comparable to that of aRu3/WC_x (CN = 1.72), EXAFS fitting revealed a substantially shorter Ru-Ru bond length of 2.61 Å (**Supplementary Table 19**), which is even shorter than that of bulk metallic Ru (~2.68 Å). This experimentally observed tendency is fully consistent with the DFT prediction that Ru ensembles stabilized in carbon-based environments exhibit contracted Ru-Ru bond lengths, whereas Ru ensembles stabilized on WC_x exhibit elongated Ru-Ru bond lengths relative to bulk Ru. Therefore, both the theoretical calculations and the experimental aRu/NC control sample consistently demonstrate that carbonaceous environments do not reproduce the characteristic Ru-Ru bond length elongation observed for the Ru ALN/WC_x structures.

Taken together, the combined EXAFS, STEM-EDS, DFT, and control-sample analyses consistently indicate that the Ru species in aRuN/WC_x are primarily anchored at the WC_x surface through direct Ru-W and Ru-C coordination environments rather than being preferentially stabilized by polymer-derived carbon species, surface carbon contaminants, or graphitic carbon shells. While minor contributions from surface carbon species cannot be completely excluded, several independent observations collectively support this conclusion: (i) substantial Ru-W coordination is consistently observed by EXAFS, indicating direct association between Ru species and WC_x supports; (ii) STEM-EDS analyses reveal that Ru species are selectively correlated with W-containing domains rather than carbon-rich regions; and (iii) only WC_x-supported Ru ensembles reproduce the characteristic Ru-Ru bond length elongation observed experimentally, where as carbon-based environments consistently yield contracted Ru-Ru bond lengths. These results therefore provide strong evidence that the first-shell Ru-C coordination predominantly originates from carbide-derived carbon atoms participating in the Ru ALN/WC_x interfacial structure.

Revision

To clarify the origin of the first-shell Ru-C coordination observed by EXAFS and address the reviewer's concern regarding possible stabilization of Ru species by polymer-derived carbon species,

surface carbon contaminations, or graphitic carbon shells, we performed additional microscopic, theoretical and control-sample investigations.

First, high-resolution STEM-EDS analyses were conducted on both WC_x particles and carbon-rich regions to directly examine the spatial distribution of Ru species relative to W- and C-containing domains. Second, DFT calculations were performed for Ru ensembles adsorbed on graphene surfaces and confined between graphene layers to evaluate whether carbonaceous environments can reproduce the experimentally observed structural characteristics of Ru ALNs. Third, a control catalyst consisting of Ru atomic clusters stabilized on N-functionalized carbon (aRu/NC) was synthesized and characterized to experimentally compare the Ru coordination environment with that of aRu3/WC_x. Based on these additional analyses, Supplementary Note 8 was expanded to discuss the origin of the Ru-C coordination observed in the Ru ALN/WC_x system.

The corresponding discussions, figures, captions, and experimental details have been added or revised as follows.

(Page 9 in the Supplementary Note 8 of the revised Supplementary Information)

To further clarify whether the first-shell Ru-C coordination originated from carbide-derived carbon in WC_x or from carbonaceous environments such as polymer-derived carbon species, surface carbon contaminants, or graphitic carbon shells, additional EXAFS, STEM-EDS, DFT, and control-sample analyses were performed. In all aRuN/WC_x samples, substantial Ru-W coordination was observed together with Ru-C coordination, with Ru-W coordination numbers exceeding unity and Ru-W bond lengths of approximately 2.84 Å (Supplementary Tables 8, 9). If the Ru species were predominantly stabilized by carbonaceous environments rather than WCx domains, direct Ru-W coordination would be expected to be negligible. High-resolution STEM-EDS analyses further showed that Ru signals were selectively correlated with W-containing domains, whereas Ru intensity became negligible in carbon-rich regions, regardless of whether the analyses were performed on selected WC_x particles or carbon-rich areas (Supplementary Figs. 71-74). These results indicate that Ru species are preferentially associated with WC_x domains rather than carbonaceous regions.

Additional DFT calculations were performed for Ru3 and Ru4 ensembles adsorbed on graphene surfaces or confined between graphene layers to model possible carbon-derived coordination environments (Supplementary Fig. 75). Experimentally, the Ru ALN/WC_x samples exhibited elongated Ru-Ru bond lengths (~2.74 Å) compared with bulk metallic Ru (~2.68 Å), and this tendency was reproduced by Ru ensembles stabilized on γ-WC but not by carbon-supported or graphitic shell-confined Ru ensembles (Supplementary Fig. 76). Consistently, the aRu/NC control sample, which contains Ru atomic clusters stabilized on N-functionalized carbon with a Ru-Ru coordination number comparable to that of aRu3/WC_x, exhibited a contracted Ru-Ru bond length of 2.61 Å (Supplementary Figs. 111, 112, Supplementary Table 19). Taken together, these results indicate that carbonaceous environments do not reproduce the characteristic Ru-Ru bond length elongation of Ru ALN/WC_x supporting that the first shell Ru-C coordination predominantly originates from carbide-derived carbon atoms participating in the Ru ALN/WC_x interfacial structure.

(Page 89-94 in the revised Supplementary Information)

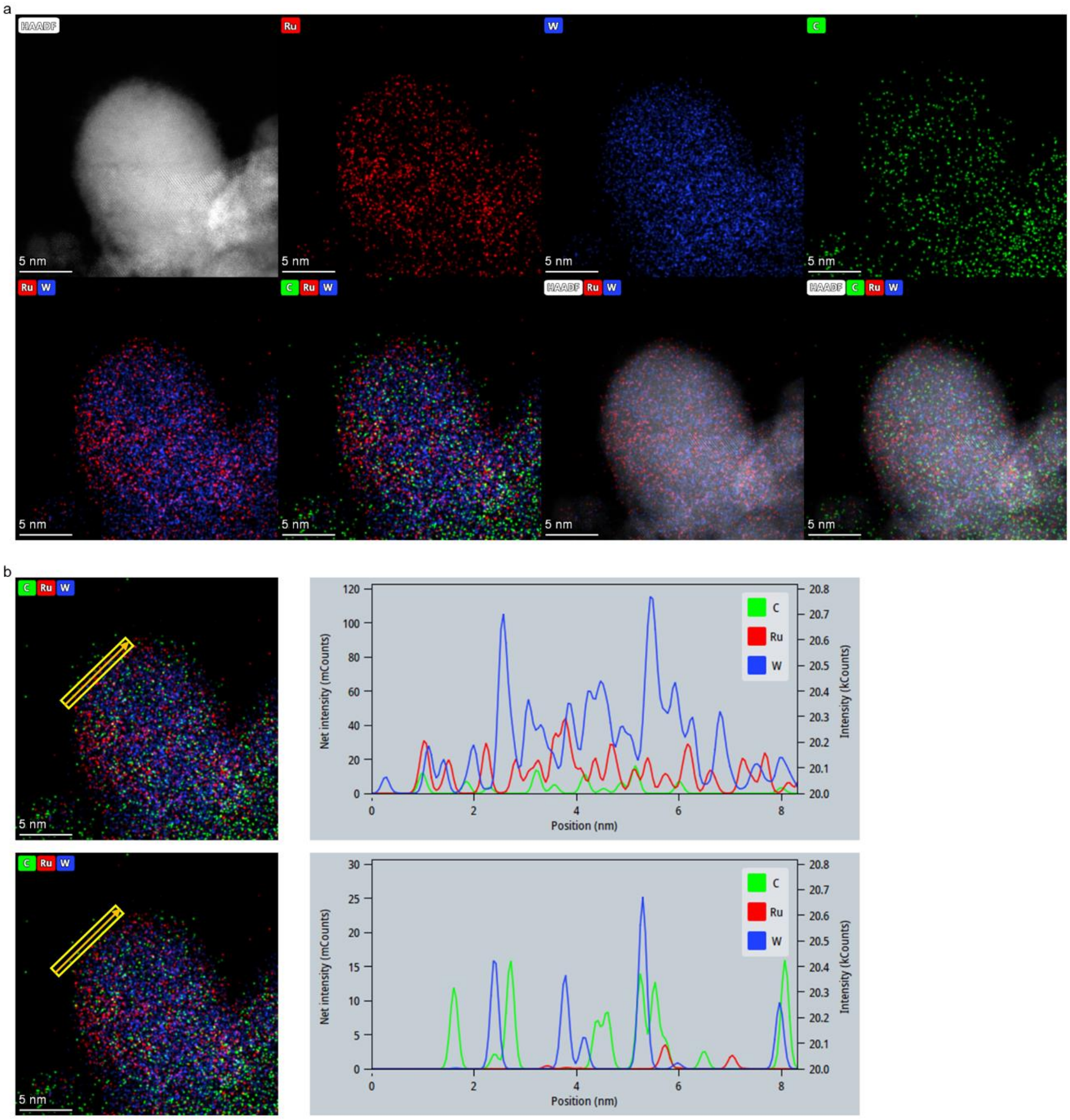

Supplementary Fig. 71 | High-resolution STEM-EDS analysis of selected WCx particles in aRu3/WC$_x$. a, HAADF-STEM image and the corresponding EDS elemental mappings of C (green), W (blue), Ru (red) for a representative WC$_x$ particle in aRu3/WC$_x$, together with the overlaid elemental distributions. **b,** Representative EDS line-scan profiles acquired along the directions indicated by the yellow rectangles

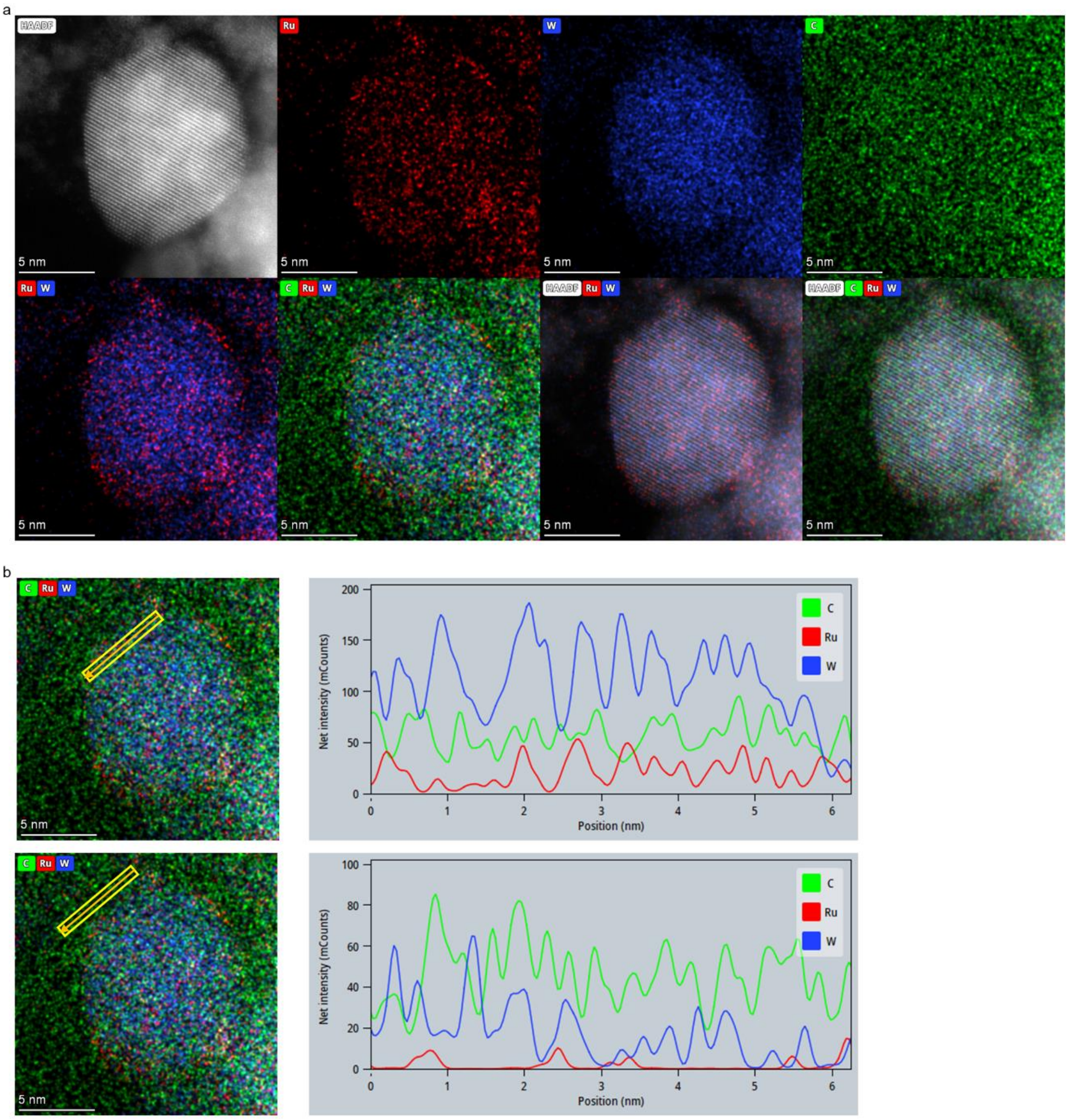

Supplementary Fig. 72 | High-resolution STEM-EDS analysis of selected WCx particles in aRu3/WC$_x$. a, HAADF-STEM image and the corresponding EDS elemental mappings of C (green), W (blue), Ru (red) for a representative WC$_x$ particle in aRu3/WC$_x$, together with the overlaid elemental distributions. **b,** Representative EDS line-scan profiles acquired along the directions indicated by the yellow rectangles

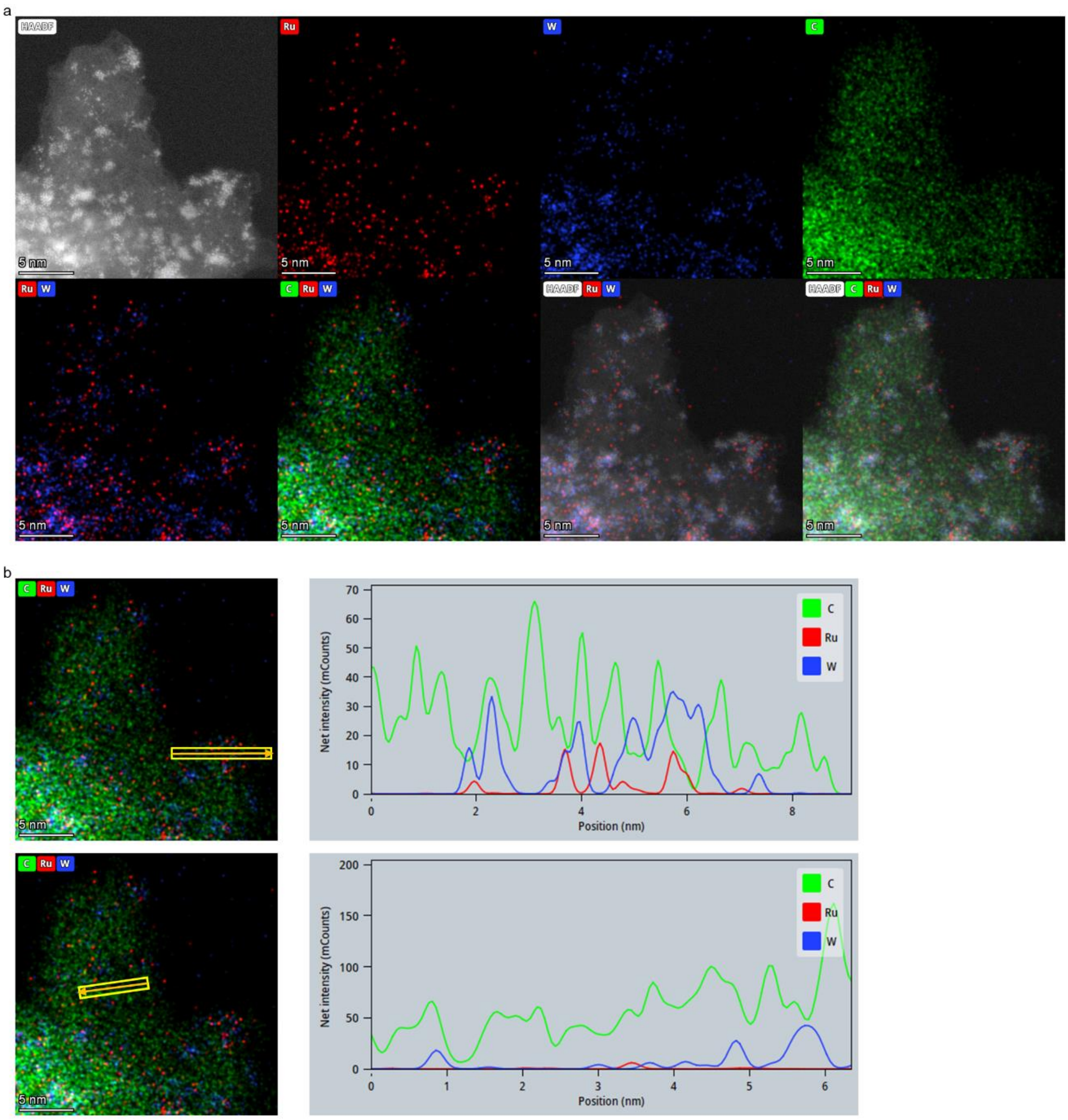

Supplementary Fig. 73 | High-resolution STEM-EDS analysis of selected carbon-rich regions in aRu3/WC$_x$. a, HAADF-STEM image and the corresponding EDS elemental mappings of C (green), W (blue), Ru (red) for a representative carbon-rich region in aRu3/WC$_x$, together with the overlaid elemental distributions. **b,** Representative EDS line-scan profiles acquired along the directions indicated by the yellow rectangles

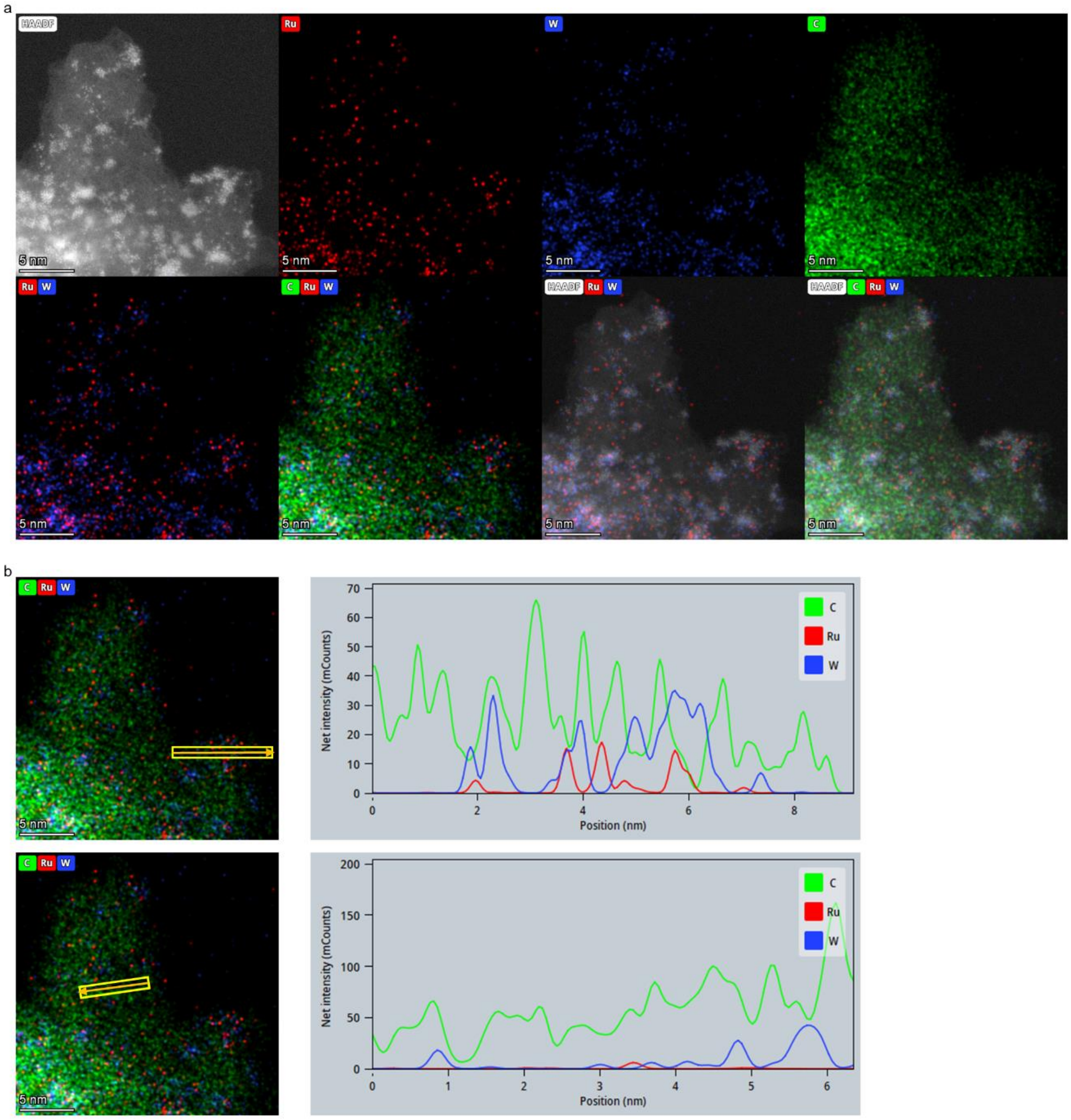

Supplementary Fig. 74 | High-resolution STEM-EDS analysis of selected carbon-rich regions in aRu3/WC$_x$. a, HAADF-STEM image and the corresponding EDS elemental mappings of C (green), W (blue), Ru (red) for a representative carbon-rich region in aRu3/WC$_x$, together with the overlaid elemental distributions. **b,** Representative EDS line-scan profiles acquired along the directions indicated by the yellow rectangles

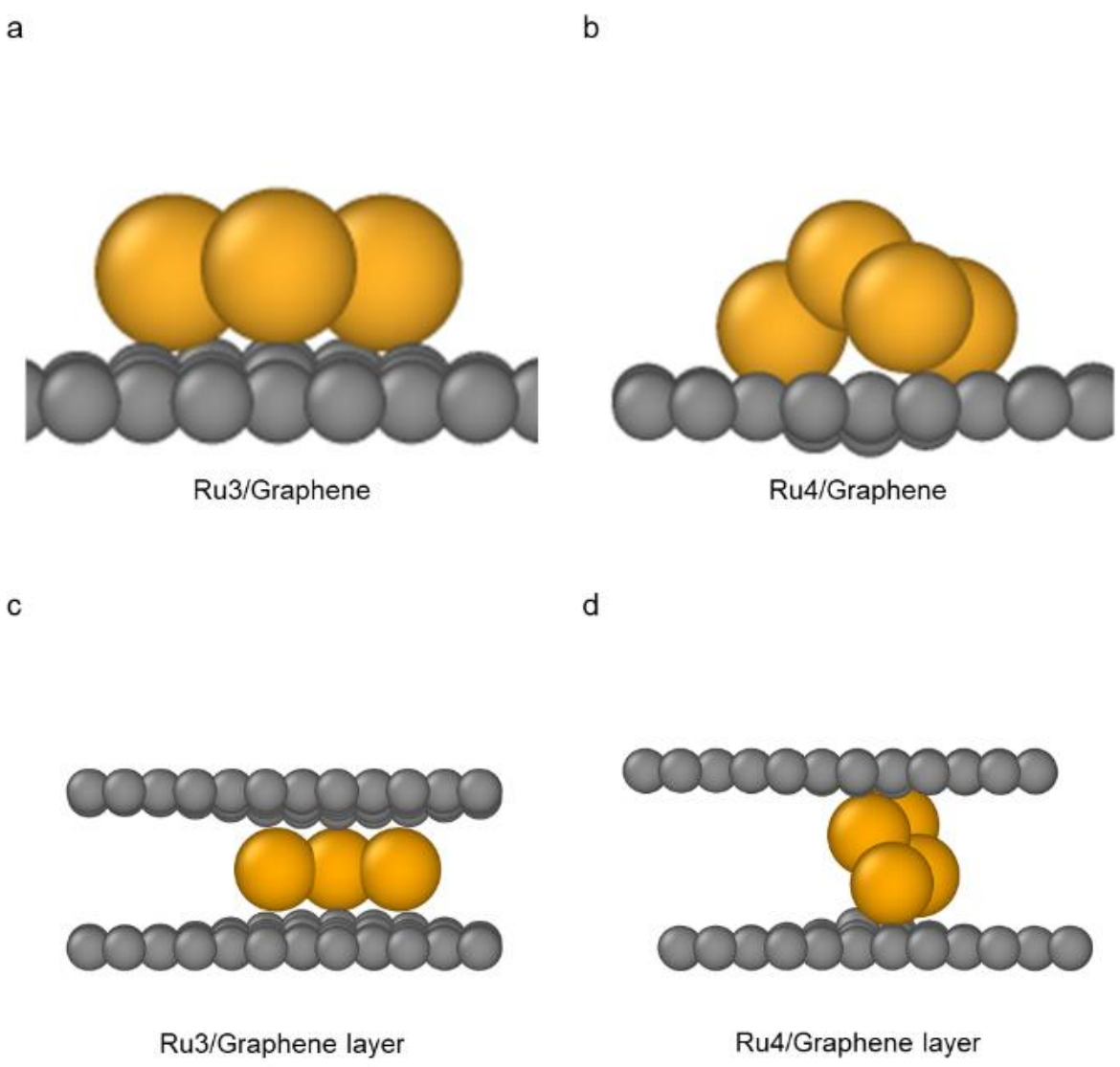

Supplementary Fig. 75 | DFT models representing possible carbon-derived coordination environments of Ru ensembles. a-b, DFT-optimized structures of Ru3 and Ru4 ensembles adsorbed on graphene surfaces. **c-d,** DFT-optimized structures of Ru3 and Ru4 ensembles confined between graphene layers.

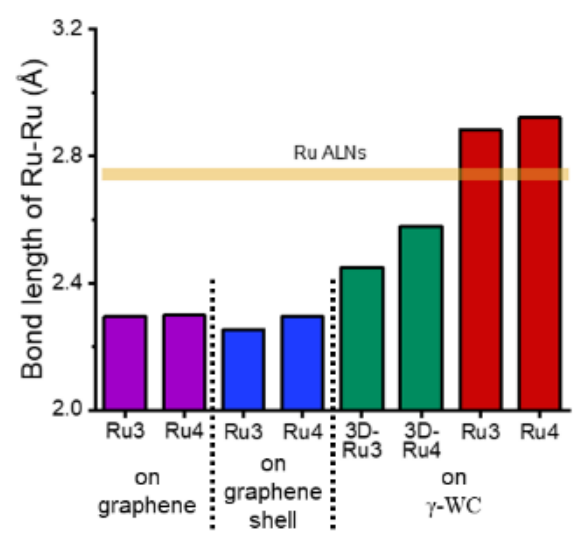

Supplementary Fig. 76 | Comparison of Ru-Ru bond lengths for Ru ensembles stabilized in different coordination environments. Average Ru-Ru bond lengths obtained from DFT-optimized Ru3 and Ru4 ensembles adsorbed on graphene surface (purple), Ru3 and Ru4 ensembles confined between graphene layers (blue), three-dimensional Ru3 and Ru4 ensembles on γ-WC supports (green), and Ru3 and Ru4 ALN stabilized on γ-WC supports (red). The orange shaded region represents the experimentally fitted Ru-Ru bond length range of the Ru ALN/WC_x samples (aRuN/WC_x).

(Page 129-130, 172 in the revised Supplementary Information)

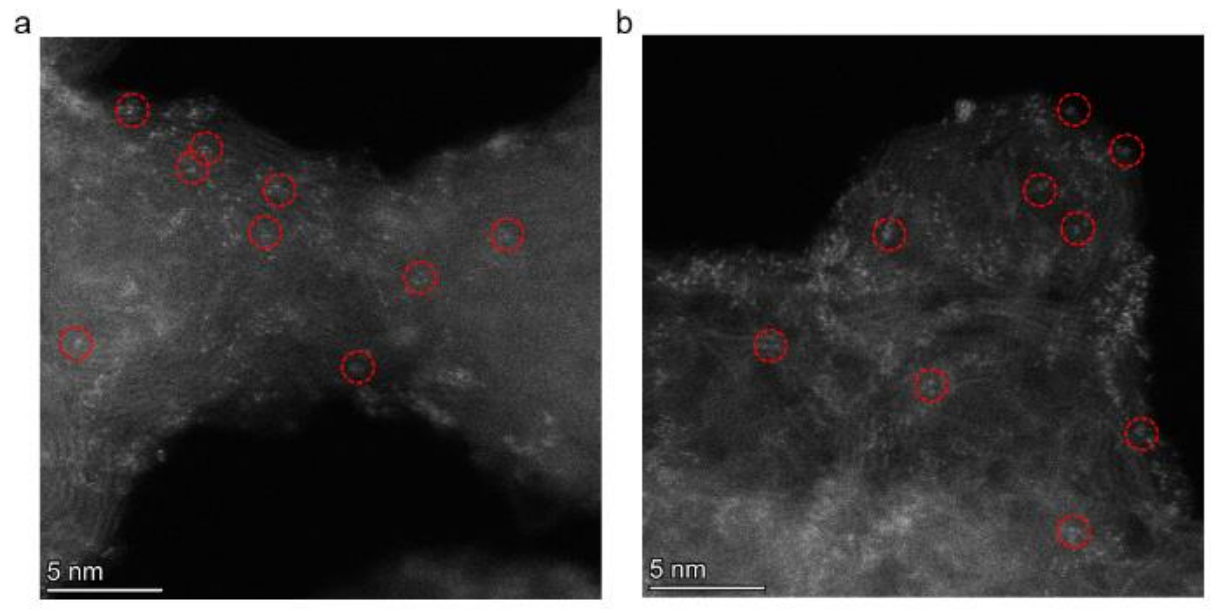

Supplementary Fig. 111 | Atomic-scale structure of aRu/NC. a-b, Representative HAADF-STEM images of aRu/NC (red dotted circles: Ru clusters composed of fewer than 10 Ru atoms)

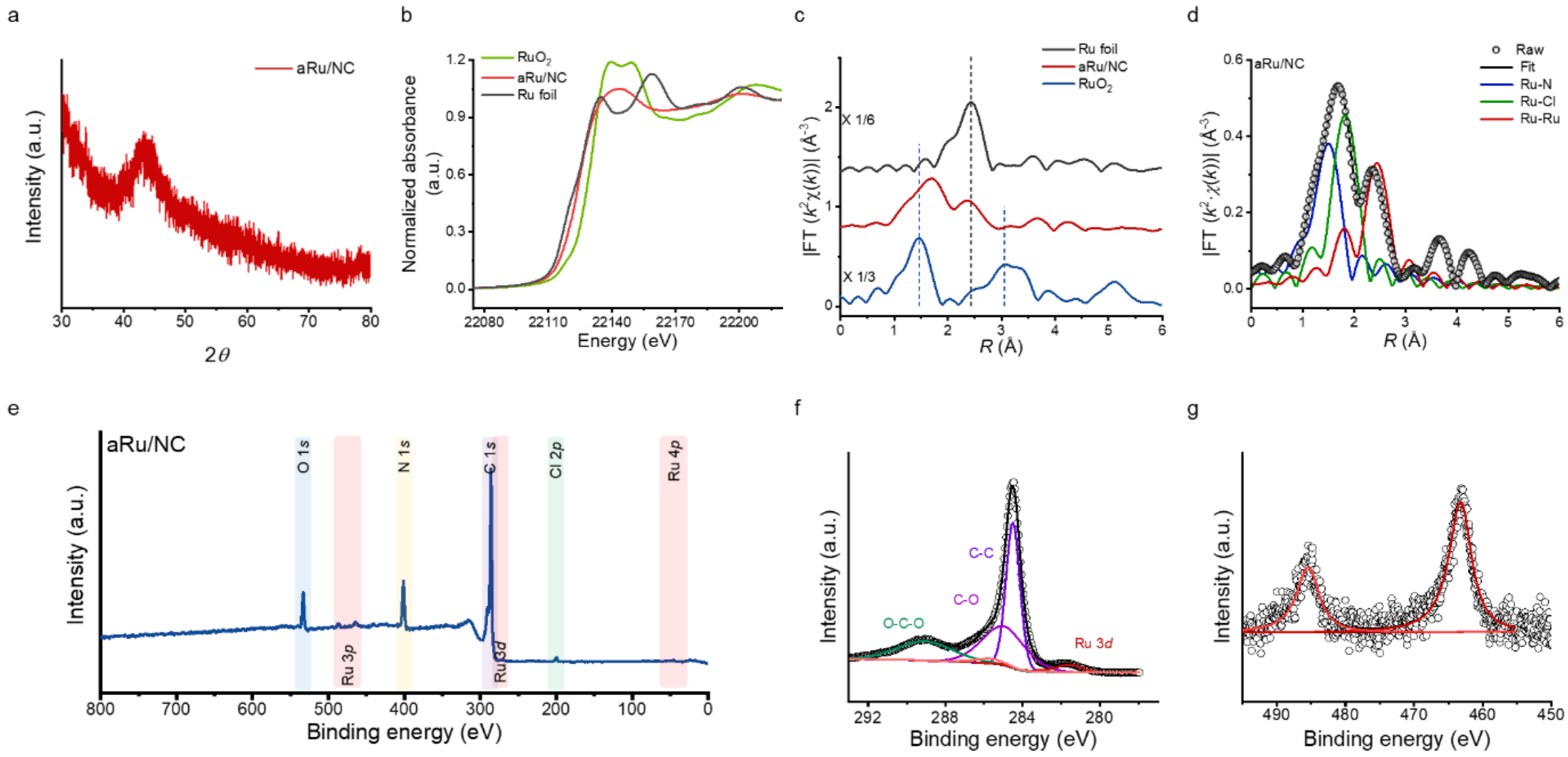

Supplementary Fig. 112 | Geometric and electronic structure of aRu/NC. a, XRD pattern of aRu/NC. **b-d,** XAS analyses of aRu/NC at the Ru K-edge, including (**b**) XANES spectra, (**c**) k^2-weighted FT-EXAFS spectra, and (**d**) corresponding FT-EXAFS fitting results. **e-g,** XPS analyses of aRu/NC, including (**e**) survey spectrum, (**f**) C 1s spectrum, and (**g**) Ru 3p spectrum.

Supplementary Table 19 | Structural parameters extracted from EXAFS fitting analyses. Structural parameters extracted from the Ru K-edge, Pt L_2-edge, Ir L_2-edge EXAFS fitting results. CN: coordination number, R: interatomic distance, and σ^2: Debye-Waller factor. The goodness of fit is represented by the R-factor. (weighting: k^2)

Sample	Shell	CN	R (Å)	σ^2 (10^{-3} Å^2)	R-factor
Ru	Ru-Ru	6*	2.65 ± 0.01	2.56	0.007
	Ru-Ru	6*	2.70 ± 0.01		
RuO_2	Ru-O	2*	1.94 ± 0.01	0.73	0.014
	Ru-O	4*	1.98 ± 0.01		
Ru/WC_x_R	Ru-O	2.06 ± 0.33	1.97 ± 0.01	5.69	0.012
	Ru-Ru	4.51 ± 0.41	2.66 ± 0.01	6.93	
Ru/C_R	Ru-O	2.79 ± 0.37	1.97 ± 0.01	6.95	0.014
	Ru-Ru	3.70 ± 0.41	2.67 ± 0.01	6.45	
RuNP/WC_x	Ru-O	2.93 ± 0.19	1.94 ± 0.01	5.87	0.003
	Ru-Cl	0.86 ± 0.14	2.30 ± 0.01		
	Ru-Ru	2.27 ± 0.23	2.66 ± 0.01	5.74	
RuNP/C	Ru-O	4.63 ± 0.19	1.97 ± 0.00	17.23	0.001
	Ru-Cl	1.45 ± 0.14	2.27 ± 0.01		
	Ru-Ru	2.35 ± 0.11	2.66 ± 0.00	5.00	
aRu/NC	Ru-N	1.53 ± 0.06	2.01 ± 0.00	1.75	0.001
	Ru-Cl	1.06 ± 0.03	2.33 ± 0.00		
	Ru-Ru	1.92 ± 0.08	2.61 ± 0.00	8.14	
aPt3/WC_x**	Pt-C	1.59 ± 0.30	2.08 ± 0.02	2.16	0.009
	Pt-Pt	4.10 ± 0.34	2.75 ± 0.01	2.07	
	Pt-W	1.93 ± 0.37	2.88 ± 0.01		
aIr3/WC_x**	Ir-C	2.46 ± 0.08	2.11 ± 0.00	2.69	0.001
	Ir-Ir	1.87 ± 0.09	2.73 ± 0.00	1.49	
	Ir-W	1.06 ± 0.09	2.88 ± 0.01		

Structural parameters extracted from the EXAFS fitting of reference metal foil. CN is the coordination number; R is the interatomic distance; σ^2 is the Debye-Waller factor (a measure of the static and thermal disorder in absorber-scatterer distance); ΔE_0 is edge energy shift (the zero kinetic energy difference between experiment and theoretical model); R-factor value is related with the goodness of the fitting.
* Value was assigned in curving fitting based on standard structure
** For Pt and Ir, EXAFS fitting was conducted at the L_2-edge to avoid spectral overlap between the L_3-edge and the W L_2-edge. S_0^2 value was obtained from reference foil measurements at the corresponding L_2-edge
R_{bkg} was set to 1.0 in background removal to remove the noise oscillation below 1 Å in R-space
S_0^2 value (0.744) was calculated from the fitting of metal foil with standard metal structure

(Page 22 in the *Experimental* section of the revised Manuscript)

Synthesis of aRu/NC. For control experiments, aRu/NC were prepared by IWI using soft-nitrided Vulcan XC 72R (*J. Am. Chem. Soc.*, **2016**, 138, 4718-4721). For the synthesis, 27.2 mg of HPL Ru was dissolved in 20 mL of ethanol, sonicated for 10 min, and stirred for 10 min to obtain a homogeneous solution. Next, 0.2 g of soft-nitrided carbon was added to the solution, dispersed by sonication for 10 min, then stirred at 323 K until complete solvent evaporation. The resulting powder was dried at 323 K under vacuum for 12 h. It was then annealed in a tube furnace under H_2 (20 sccm) and Ar (180 sccm) flow at 473 K for 2 h (1 K min^{-1}), then *RT* (5 K min^{-1}). After cooling to *RT*, the sample was passivated under O_2 (2 sccm) and Ar (200 sccm) flow for 12 h, and finely ground in an agate mortar for 10 min.

3. The authors admit that HAADF-STEM Z-contrast is difficult to distinguish Ru due to the W background and the 2D atom-layer nature is largely inferred from EXAFS Ru-Ru CN (< 2.5) and simulations. However, a CN of ~2.5 could also correspond to a very small 3D tetrahedral cluster. Therefore, further evidence should be provided.

Reply

We thank the reviewer for this insightful comment. We agree that a Ru-Ru coordination number (CN) of ~2.5 alone does not uniquely distinguish a two-dimensional Ru ALN structure from a very small three-dimensional Ru cluster, such as a tetrahedral Ru4 ensemble. Therefore, additional theoretical and experimental analyses were performed to evaluate this possibility.

First, we explicitly constructed a tetrahedral Ru4 model on γ-WC and performed DFT geometry optimization while maintaining the three-dimensional tetrahedral motif (**Supplementary Fig. 81**). To examine whether such a structure could be directly identified experimentally, HAADF-STEM image simulations were additionally performed using a 10-layer γ-WC slab (**Supplementary Fig. 82**). The simulated images indicate that a tetrahedral Ru4 cluster of this size would be extremely difficult to distinguish from small Ru ensembles based solely on projected HAADF-STEM contrast. Therefore, we agree with the reviewer that HAADF-STEM imaging alone cannot conclusively exclude the presence of very small three-dimensional Ru clusters.

Instead, we examined structural descriptors that can be directly compared between experiment and theory. The DFT-optimized tetrahedral Ru4 structure exhibited an average Ru-Ru bond length of 2.58 Å (**Supplementary Fig. 81**), substantially shorter than the experimentally observed Ru-Ru bond lengths of the Ru ALN/WC_x samples (~2.74 Å). Similarly, DFT calculations for the three-dimensional Ru3 ensemble on γ-WC exhibited an average Ru-Ru bond length of 2.45 Å (**Supplementary Figs. 68, 70**), significantly shorter than both bulk metallic Ru (2.68 Å) and experimentally observed Ru ALN/WC_x values (**Supplementary Fig. 76**). These results demonstrate that the presence of the WC support alone does not lead to Ru-Ru bond length elongation. Rather, elongation is observed only when the Ru ensembles are stabilized in the ALN configuration through strong interactions with WC_x. In contrast, three-dimensional Ru ensembles, even when supported on γ-WC, consistently exhibit contracted Ru-Ru bond lengths. Therefore, only the Ru ALN models reproduce the experimentally observed Ru-Ru bond length elongation, whereas compact three-dimensional Ru clusters do not.

This conclusion is further supported by experimental control samples. As discussed in our response to Comment 2, aRu/NC was synthesized as a representative Ru atomic-cluster catalyst stabilized on N-functionalized carbon (**Supplementary Fig. 111, 112**). Although aRu/NC exhibited a Ru-Ru coordination number (1.92) comparable to that of aRu3/WC_x (1.72), EXAFS fitting revealed a contracted Ru-Ru bond length of 2.61 Å, which is even shorter than that of bulk metallic Ru (~2.68 Å) (**Supplementary Table 19**). This tendency is fully consistent with the DFT results showing that Ru ensembles retaining compact three-dimensional geometries exhibit contracted Ru-Ru bond lengths, whereas Ru ensembles stabilized as Ru ALNs through strong interactions with WC_x exhibit

elongated Ru-Ru bond lengths. Thus, both the DFT models and experimentally observed three-dimensional Ru atomic clusters consistently exhibit contracted Ru-Ru bond lengths, indicating that the characteristic Ru-Ru bond length elongation observed for the Ru ALN/WC_x samples is not a general feature of small Ru clusters but rather a distinctive structural signature of the Ru ALN configuration.

We further compared aRu5/WC_x with experimentally verified Ru nanoparticle and nanocluster samples on WC_x supports (aRu0/WC_x), including RuNP/WC_x and Ru/WC_x_R. In RuNP/WC_x, sub-2 nm Ru nanoparticles were identified by distinct Z-contrast features in HAADF-STEM images (**Supplementary Fig. 113**). Likewise, Ru/WC_x_R exhibited discernible Ru nanoclusters smaller than 1 nm despite their extremely small size (**Supplementary Figs. 39**), indicating that small three-dimensional Ru species can, at least partially, be visualized under the present imaging conditions. EXAFS fitting showed that the Ru nanoparticles in RuNP/WC_x possessed a Ru-Ru coordination number of 2.27, comparable to that of aRu5/WC_x (2.29). However, unlike RuNP/WC_x and Ru/WC_x_R, no analogous bright nanoparticle- or nanocluster-like features were observed for aRu5/WC_x in either the representative HAADF-STEM image (**Supplementary Fig. 23**) or the high-resolution HAADF-STEM image (**Supplementary Fig. 83**). Furthermore, the Ru nanoparticles exhibited a Ru-Ru bond length of ~2.66 Å, slightly shorter than that of bulk metallic Ru (~2.68 Å) and substantially shorter than those observed for the Ru ALN/WC_x samples. Thus, while HAADF-STEM imaging alone cannot completely exclude the presence of extremely small three-dimensional Ru species, the observation of discernible sub-nanometer Ru nanoclusters in Ru/WC_x_R suggests that a substantial population of analogous three-dimensional Ru nanoclusters in aRu5/WC_x would likely generate at least some detectable contrast features. However, no such features were observed. Together with the contracted Ru-Ru bond lengths consistently observed for the nanoparticle and nanocluster control samples, these results contrast sharply with the pronounced Ru-Ru bond length elongation observed for aRu5/WC_x. This behavior is therefore more consistent with the Ru species in aRu5/WC_x being stabilized as Ru ALNs rather than as three-dimensional Ru clusters.

Taken together with the results presented in our responses to Comments 1 and 2, the experimentally observed combination of (i) elongated Ru-Ru bond lengths, (ii) distinct Ru-W coordination environments characterized by Ru-W bond lengths of ~2.84 Å and Ru-W coordination numbers greater than unity, and (iii) Ru-C coordination associated with WC_x stabilization is most consistently explained by the Ru ALN structure proposed in this work (**Supplementary Note 8**). Therefore, although Ru–Ru coordination number alone cannot exclude very small three-dimensional clusters, the collective experimental and theoretical evidence strongly supports the assignment of the Ru species as Ru ALNs rather than tetrahedral Ru clusters or Ru nanoparticles.

Revision

To address the reviewer's concern that a Ru-Ru coordination number (CN) of ~2.5 may also be consistent with a very small three-dimensional Ru cluster rather than a two-dimensional Ru ALN structure, we performed additional theoretical simulations, microscopic analyses, and control-sample studies.

First, a tetrahedral Ru4 cluster model was constructed on γ-WC and analyzed by DFT calculations and HAADF-STEM image simulations to evaluate whether a compact three-dimensional Ru cluster could reproduce the experimentally observed structural characteristics. Second, additional high-resolution HAADF-STEM analyses were performed to further examine the morphology of aRu5/WC_x. Third, two independent control systems were investigated: (i) atomically dispersed Ru clusters supported on N-functionalized carbon (aRu/NC) and (ii) Ru nanoparticle and Ru nanocluster catalysts supported on WC_x (RuNP/WC_x and Ru/WC_x_R). These control samples were used to compare the Ru-Ru coordination environment, Ru-Ru bond length, and microscopic features with those of the Ru ALN/WC_x samples. Based on these additional analyses, Supplementary Note 8 was expanded to discuss the structural distinction between Ru ALNs and compact three-dimensional Ru clusters.

The corresponding discussions, figures, captions, tables, and experimental details have been added or revised as follows.

(Page 10-11 in the Supplementary Note 8 of the revised Supplementary Information)

To further evaluate whether the small Ru−Ru CNs of aRuN/WC_x could originate from compact three-dimensional Ru clusters rather than two-dimensional Ru ALNs, additional DFT and STEM-simulation analyses were performed. A tetrahedral Ru4 ensemble was constructed on γ-WC and optimized while maintaining the three-dimensional tetrahedral motif (Supplementary Fig. 81). HAADF-STEM image simulations using a 10-layer γ-WC slab indicated that such a small tetrahedral Ru4 cluster is difficult to distinguish unambiguously from small Ru ensembles based solely on projected HAADF-STEM contrast (Supplementary Fig. 82). However, the DFT-optimized tetrahedral Ru4 structure exhibited an average Ru-Ru bond length of 2.58 Å, substantially shorter than the experimentally observed Ru-Ru bond lengths of the Ru ALN/WC_x samples (~2.74 Å). Similarly, the three-dimensional Ru3 ensemble on γ-WC exhibited an average Ru-Ru bond length of 2.45 Å, shorter than both bulk metallic Ru (2.68 Å) and the experimentally observed Ru ALN/WC_x values (Supplementary Figs. 68, 70). These results indicate that the WC support alone does not induce Ru-Ru bond length elongation; rather, elongation is reproduced only when Ru ensembles are stabilized in the ALN configuration through strong interactions with WC_x.

This structural assignment was further supported by experimental control samples. The aRu/NC control catalyst, containing Ru atomic clusters stabilized on N-functionalized carbon with a Ru-Ru CN comparable to that of aRu3/WC_x, exhibited a contracted Ru-Ru bond length of 2.61 Å (Supplementary Figs. 111, 112, Supplementary Table 19). In addition, experimentally verified Ru nanoparticle and nanocluster samples, including RuNP/WC_x and Ru/WC_x_R, showed identifiable nanoparticle- or nanocluster-like contrast features in HAADF-STEM images, whereas no analogous features were observed for aRu5/WC_x (Supplementary Figs. 23, 38, 83, 113). The Ru nanoparticles also exhibited a Ru-Ru bond length of ~2.66 Å, slightly shorter than that of bulk metallic Ru and substantially shorter than those of Ru ALN/WC_x samples. Therefore, although Ru-Ru CN alone cannot exclude very small three-dimensional Ru clusters, the combination of elongated Ru-Ru bond lengths, distinct Ru-W coordination,

and the absence of nanoparticle-like contrast features supports the assignment of aRuN/WC_x as Ru ALN structures rather than compact three-dimensional Ru clusters or nanoparticles.

(Page 99-100 in the revised Supplementary Information)

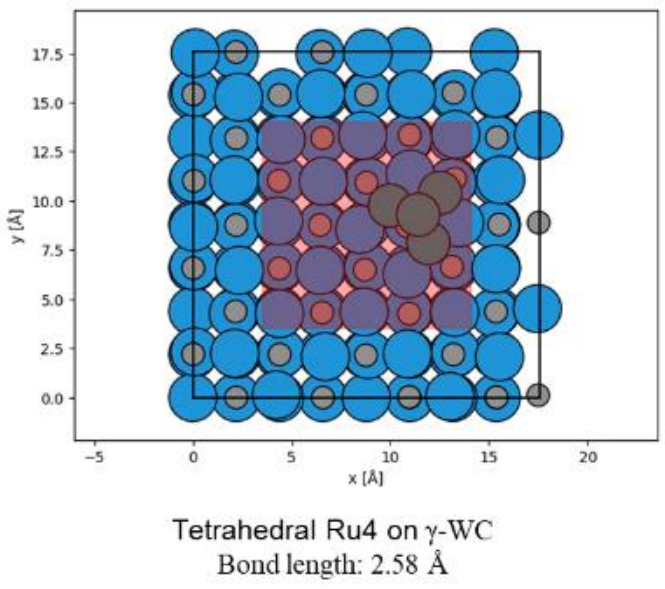

Supplementary Fig. 81 | DFT-optimized tetrahedral Ru4 ensemble on γ-WC. DFT-optimized structure of a three-dimensional tetrahedral Ru4 ensemble on γ-WC. The calculated average Ru-Ru bond length is 2.58 Å.

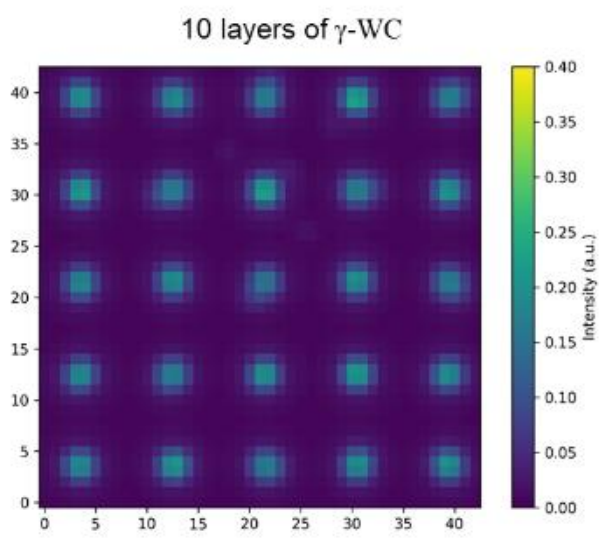

Supplementary Fig. 82 | Simulated HAADF-STEM images of a tetrahedral Ru4 ensembles on γ-WC. Simulated HAADF-STEM image of three-dimensional tetrahedral Ru4 ensembles on 10 layers of γ-WC slab.

(Page 101 in the revised Supplementary Information)

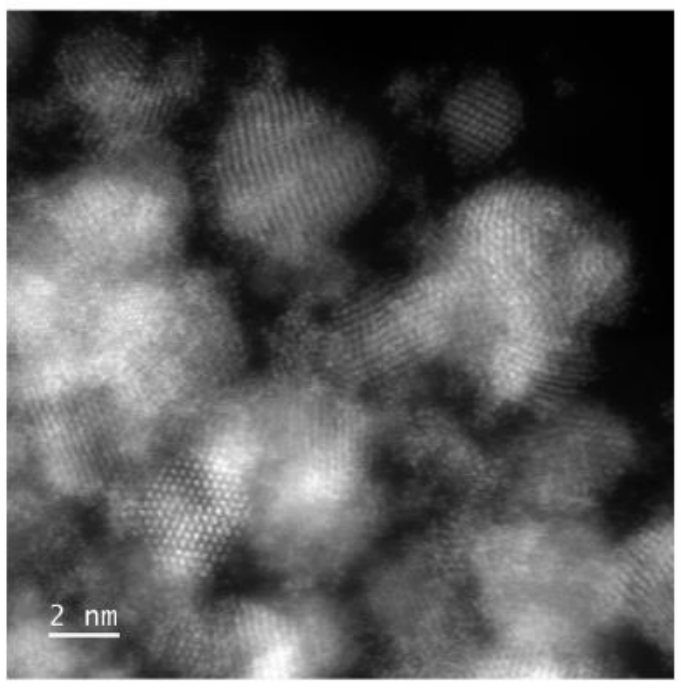

Supplementary Fig. 83 | High-resolution HAADF-STEM analysis of aRu5/WC$_x$. Representative high-resolution HAADF-STEM image of aRu5/WC$_x$.

(Page 129-133, 172 in the revised Supplementary Information)

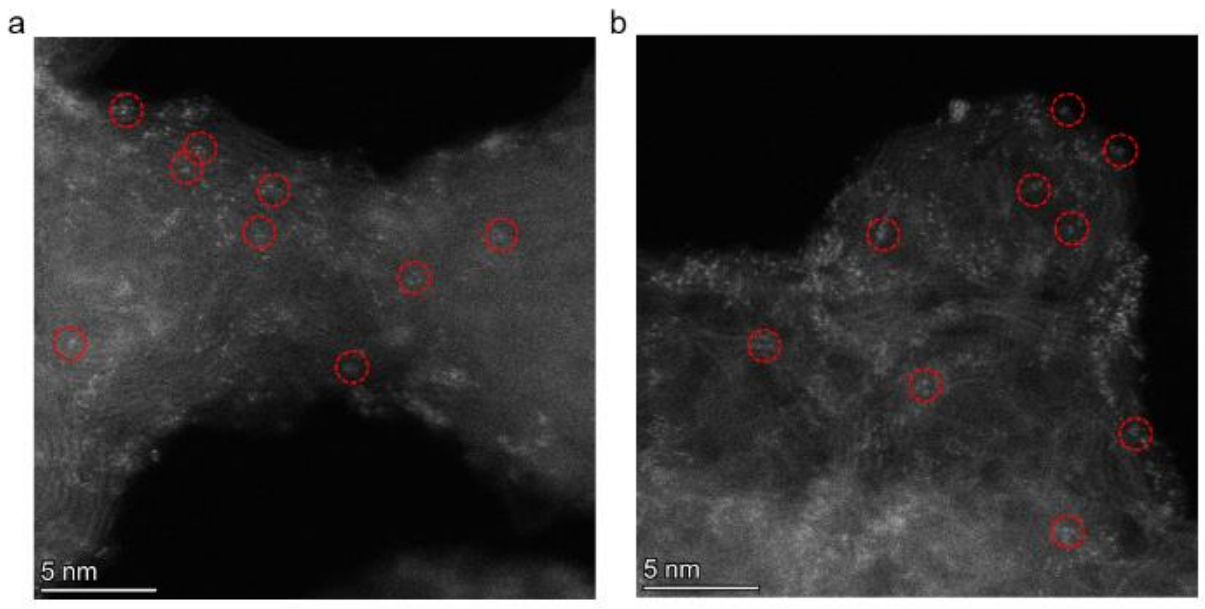

Supplementary Fig. 111 | Atomic-scale structure of aRu/NC. a-b, Representative HAADF-STEM images of aRu/NC (red dotted circles: Ru clusters composed of fewer than 10 Ru atoms)

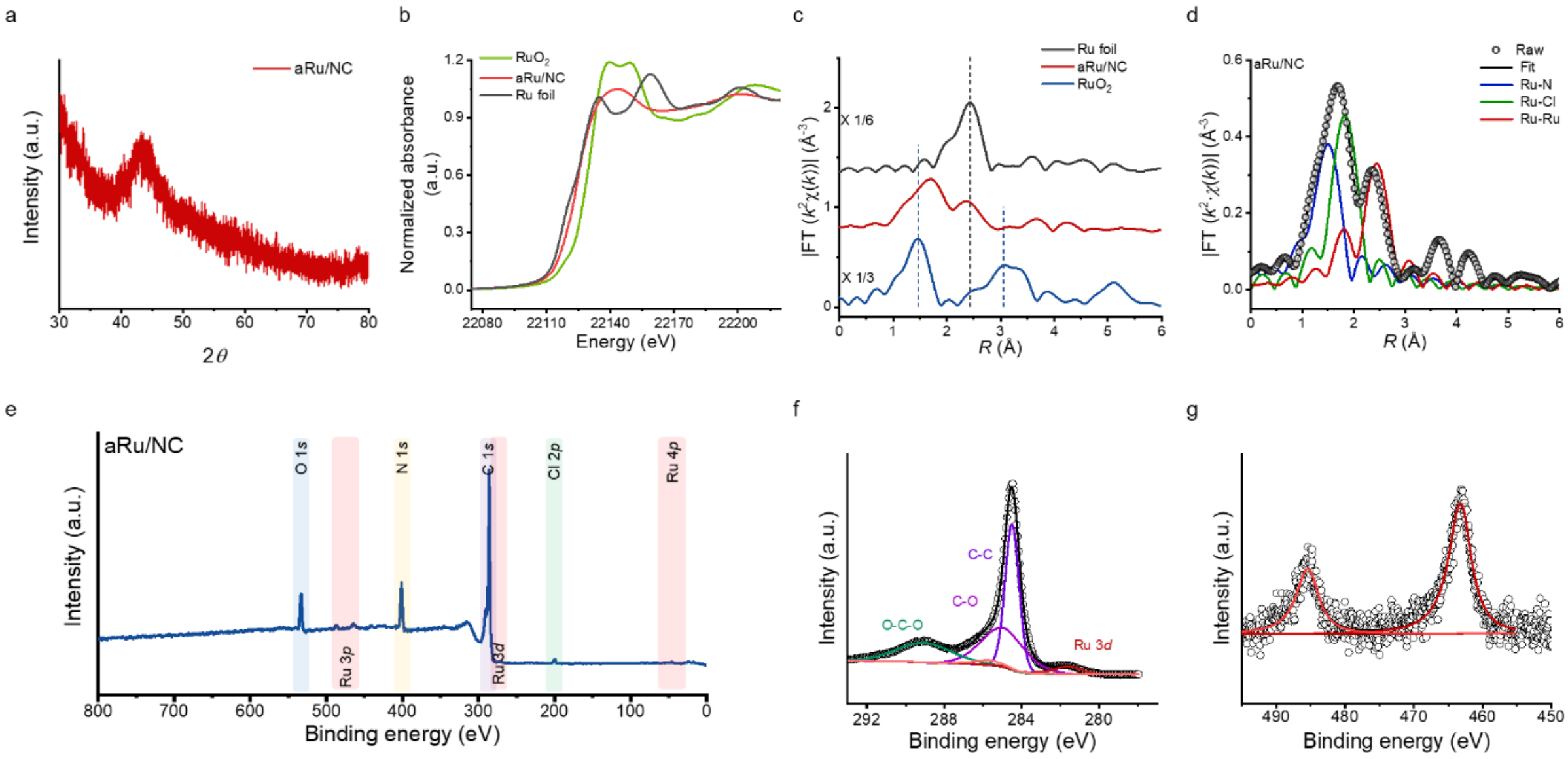

Supplementary Fig. 112 | Geometric and electronic structure of aRu/NC. a, XRD pattern of aRu/NC. **b-d,** XAS analyses of aRu/NC at the Ru K-edge, including (**b**) XANES spectra, (**c**) k^2-weighted FT-EXAFS spectra, and (**d**) corresponding FT-EXAFS fitting results. **e-g,** XPS analyses of aRu/NC, including (**e**) survey spectrum, (**f**) C 1s spectrum, and (**g**) Ru 3p spectrum.

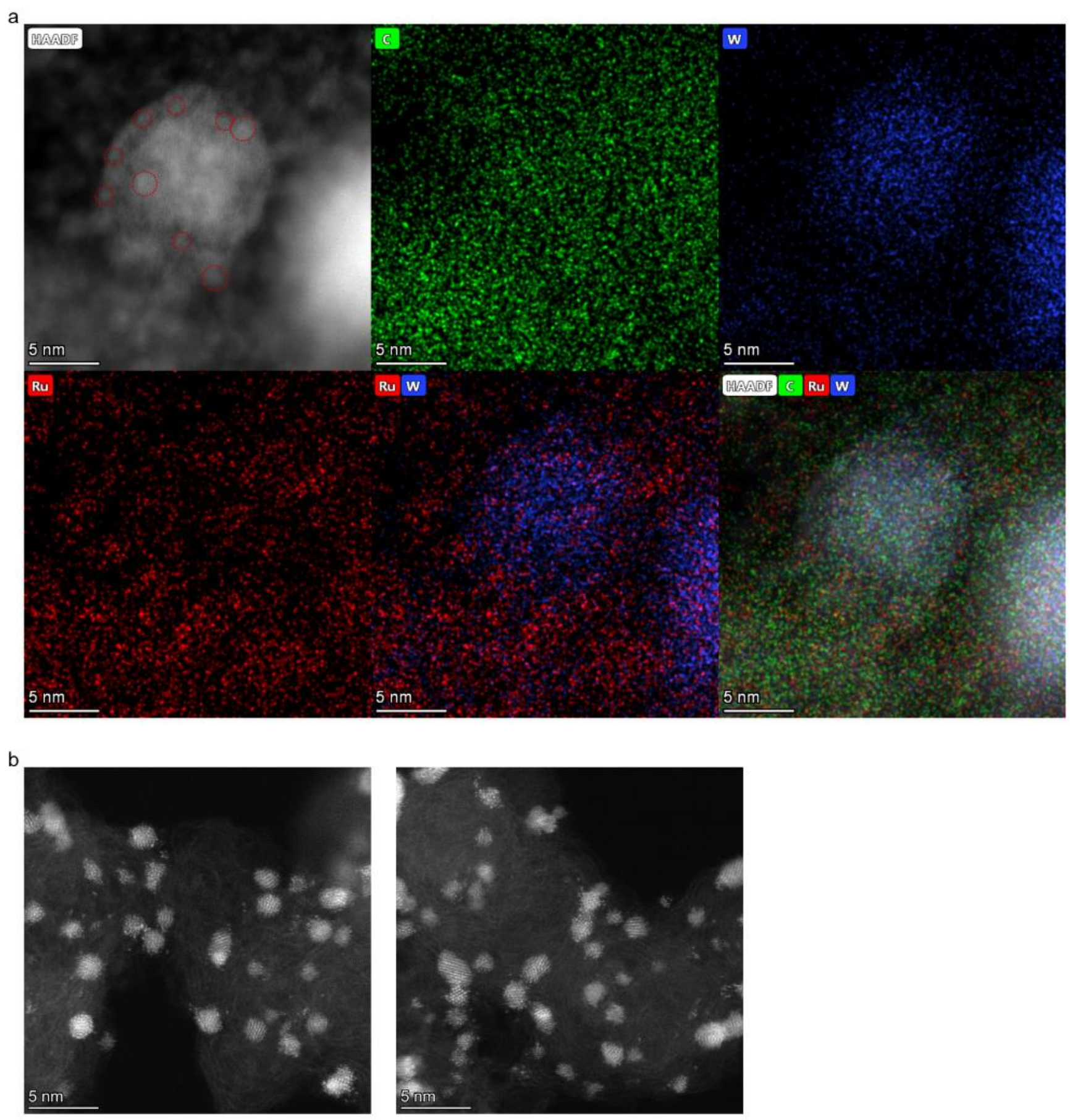

Supplementary Fig. 113 | STEM analysis of RuNP/WC$_x$ and RuNP/C. a, HAADF-STEM image and the corresponding EDS elemental mappings of RuNP/WC$_x$ for C (green), W (blue), Ru (red), along with the overlaid composite map (red dotted circle: Ru NPs). **b,** HAADF-STEM images of RuNP/C.

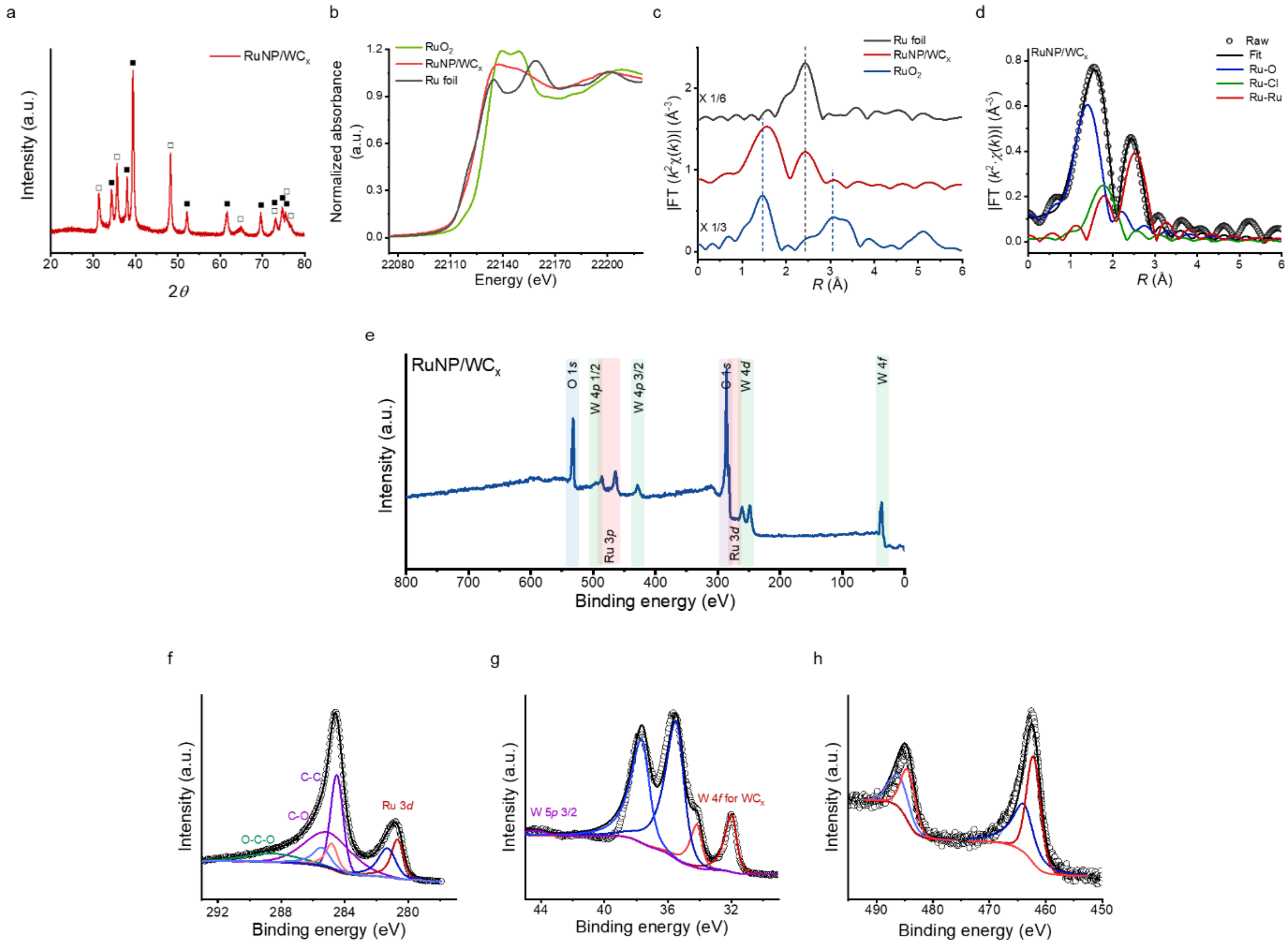

Supplementary Fig. 114 | Geometric and electronic structure of RuNP/WC$_x$. a, XRD pattern of RuNP/WC$_x$. **b-d,** XAS analyses of RuNP/WC$_x$ at the Ru K-edge, including (**b**) XANES spectra, (**c**) k^2-weighted FT-EXAFS spectra, and (**d**) corresponding FT-EXAFS fitting results. **e-h,** XPS analyses of RuNP/WC$_x$, including (**e**) survey spectrum, (**f**) C 1s spectrum, (**g**) W 4f spectrum, and (**h**) Ru 3p spectrum.

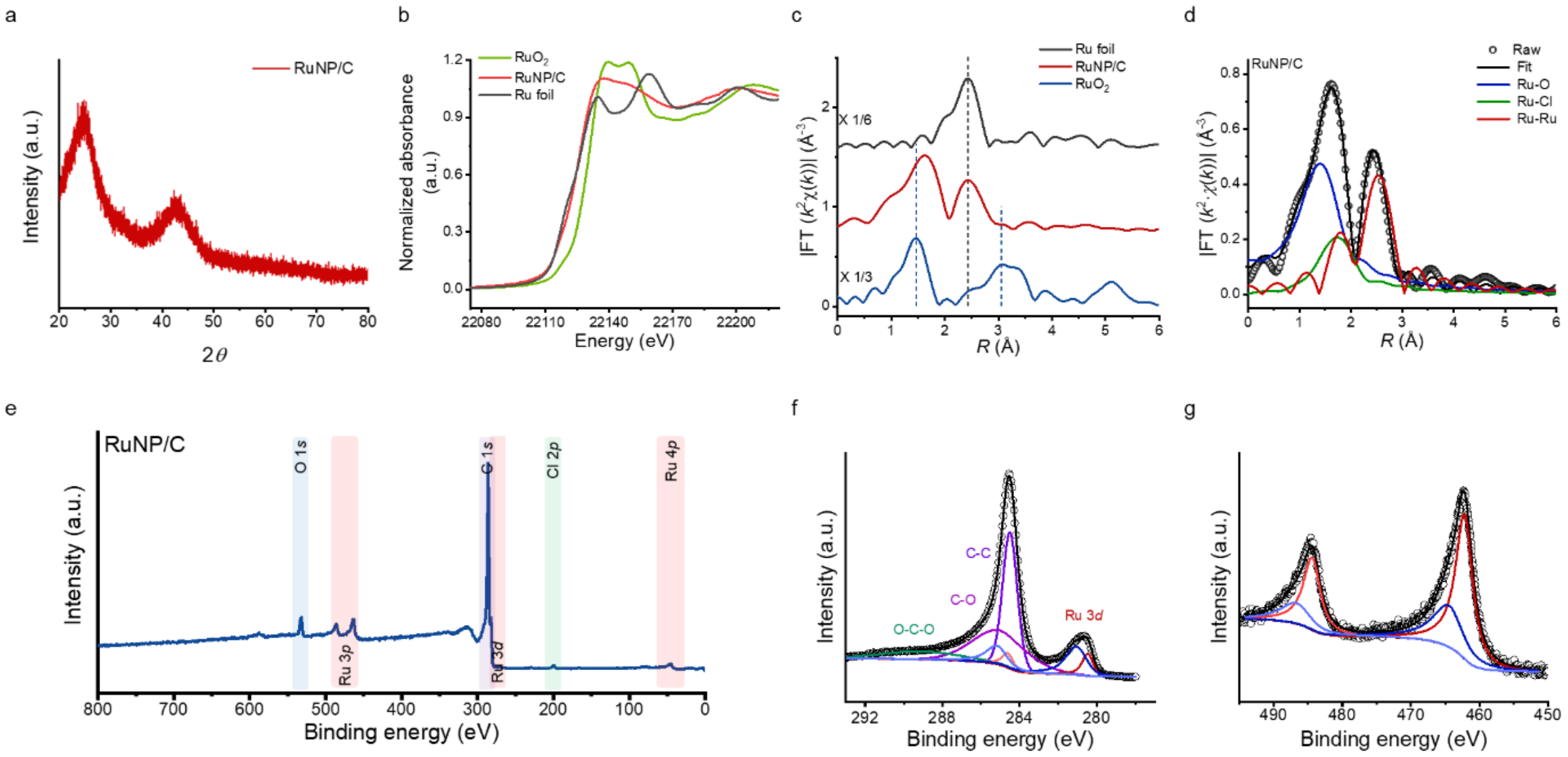

Supplementary Fig. 115 | Geometric and electronic structure of RuNP/C. a, XRD pattern of RuNP/C. **b-d,** XAS analyses of RuNP/C at the Ru K-edge, including (**b**) XANES spectra, (**c**) k^2-weighted FT-EXAFS spectra, and (**d**) corresponding FT-EXAFS fitting results. **e-h,** XPS analyses of RuNP/C, including (**e**) survey spectrum, (**f**) C 1s spectrum, and (**g**) Ru 3p spectrum.

Supplementary Table 19 | Structural parameters extracted from EXAFS fitting analyses. Structural parameters extracted from the Ru K-edge, Pt L_2-edge, Ir L_2-edge EXAFS fitting results. CN: coordination number, R: interatomic distance, and σ^2: Debye-Waller factor. The goodness of fit is represented by the R-factor. (weighting: k^2)

Sample	Shell	CN	R (Å)	σ^2 (10^{-3} Å^2)	R-factor
Ru	Ru-Ru	6*	2.65 ± 0.01	2.56	0.007
	Ru-Ru	6*	2.70 ± 0.01		
RuO_2	Ru-O	2*	1.94 ± 0.01	0.73	0.014
	Ru-O	4*	1.98 ± 0.01		
Ru/WC_x_R	Ru-O	2.06 ± 0.33	1.97 ± 0.01	5.69	0.012
	Ru-Ru	4.51 ± 0.41	2.66 ± 0.01	6.93	
Ru/C_R	Ru-O	2.79 ± 0.37	1.97 ± 0.01	6.95	0.014
	Ru-Ru	3.70 ± 0.41	2.67 ± 0.01	6.45	
RuNP/WC_x	Ru-O	2.93 ± 0.19	1.94 ± 0.01	5.87	0.003
	Ru-Cl	0.86 ± 0.14	2.30 ± 0.01		
	Ru-Ru	2.27 ± 0.23	2.66 ± 0.01	5.74	
RuNP/C	Ru-O	4.63 ± 0.19	1.97 ± 0.00	17.23	0.001
	Ru-Cl	1.45 ± 0.14	2.27 ± 0.01		
	Ru-Ru	2.35 ± 0.11	2.66 ± 0.00	5.00	
aRu/NC	Ru-N	1.53 ± 0.06	2.01 ± 0.00	1.75	0.001
	Ru-Cl	1.06 ± 0.03	2.33 ± 0.00		
	Ru-Ru	1.92 ± 0.08	2.61 ± 0.00	8.14	
aPt3/WC_x**	Pt-C	1.59 ± 0.30	2.08 ± 0.02	2.16	0.009
	Pt-Pt	4.10 ± 0.34	2.75 ± 0.01	2.07	
	Pt-W	1.93 ± 0.37	2.88 ± 0.01		
aIr3/WC_x**	Ir-C	2.46 ± 0.08	2.11 ± 0.00	2.69	0.001
	Ir-Ir	1.87 ± 0.09	2.73 ± 0.00	1.49	
	Ir-W	1.06 ± 0.09	2.88 ± 0.01		

Structural parameters extracted from the EXAFS fitting of reference metal foil. CN is the coordination number; R is the interatomic distance; σ^2 is the Debye-Waller factor (a measure of the static and thermal disorder in absorber-scatterer distance); ΔE_0 is edge energy shift (the zero kinetic energy difference between experiment and theoretical model); R-factor value is related with the goodness of the fitting.
* Value was assigned in curving fitting based on standard structure
** For Pt and Ir, EXAFS fitting was conducted at the L_2-edge to avoid spectral overlap between the L_3-edge and the W L_2-edge. S_0^2 value was obtained from reference foil measurements at the corresponding L_2-edge
R_{bkg} was set to 1.0 in background removal to remove the noise oscillation below 1 Å in R-space
S_0^2 value (0.744) was calculated from the fitting of metal foil with standard metal structure

(Page 22-23 in the *Experimental* section of the revised Manuscript)

Synthesis of aRu/NC. For control experiments, aRu/NC were prepared by IWI using soft-nitrided Vulcan XC 72R (*J. Am. Chem. Soc.*, **2016**, 138, 4718-4721). For the synthesis, 27.2 mg of HPL Ru was dissolved in 20 mL of ethanol, sonicated for 10 min, and stirred for 10 min to obtain a homogeneous solution. Next, 0.2 g of soft-nitrided carbon was added to the solution, dispersed by sonication for 10 min, then stirred at 323 K until complete solvent evaporation. The resulting powder was dried at 323 K under vacuum for 12 h. It was then annealed in a tube furnace under H_2 (20 sccm) and Ar (180 sccm) flow at 473 K for 2 h (1 K min^{-1}), then *RT* (5 K min^{-1}). After cooling to *RT*, the sample was passivated under O_2 (2 sccm) and Ar (200 sccm) flow for 12 h, and finely ground in an agate mortar for 10 min.

Synthesis of RuNP/WC_x and RuNP/C. 0.16 g of HPL Ru was dissolved in 20 mL of ethylene glycol in vial. Separately, 0.4 g of NaOH was dissolved in 20 mL of ethylene glycol in three-neck round-bottom flask. After complete dissolution, the two solutions were combined and vigorously stirred for 1 h under continuous Ar purging to remove dissolved oxygen. The resulting solution was then heated to 433 K for 3 hr (4 K min^{-1}), followed by naturally cooling to *RT*. A stable dispersion of Ru NPs in ethylene glycol was obtained. For ligand removal and support immobilization, 5 mL of Ru NP dispersion was mixed with 30 mL of 1.0 M HCL solution in a conical tube after ultrasonication of the dispersion. The mixture was centrifuged, and the supernatant was discarded. This washing process was repeated three times using a total of 15 mL of Ru NP dispersion. Subsequently, the precipitate was additionally washed with 30 mL and then 10 mL of 1.0 M HCl solution via centrifugation to collect the Ru NPs at the bottom of the tube. After acid washing, 117 mg of support materials (WCx or Vulcan XC 72R) and 5 mL of acetone were added to the conical tube. The resulting mixture was ultrasonicated for 1 h in an ice bath while preventing solvent evaporation. The obtained catalyst powder was collected by centrifugation and dried under vacuum at 323 K overnight. The dried samples were subsequently annealed in a tube furnace under H_2 (20 sccm) and Ar (180 sccm) flow at 473 K for 2 h (1 K min^{-1}), followed by an additional 2 h under pure Ar (200 sccm). The samples were then cooled to *RT* (5 K min^{-1}) and passivated under O_2 (2 sccm) and Ar (200 sccm) flow for 12 h.

4. While MD simulation is convincing, direct experimental evidence of this partitioning in the polymer melt before pyrolysis is missing. Investigating the polymer-precursor interaction via TG-DSC would experimentally validate the solvation energy calculations and strengthen the synthesis mechanism.

Reply

We thank the reviewer for this valuable suggestion. We agree that experimental evidence for precursor-polymer interactions would further strengthen the molecular interaction analysis underlying the proposed precursor-partitioning mechanism. Following the reviewer's recommendation, we performed additional TG-DSC analyses to experimentally investigate the interactions between the Ru precursors and the corresponding polymer domains prior to pyrolysis.

To mimic the precursor-containing polymer environment used during synthesis, solvent-cast precursor/polymer composites were prepared by completely dissolving PEO and HPL Ru in ethanol or PS and HPB Ru in THF, followed by solvent evaporation at 50 °C. The precursor-to-polymer ratios were identical to those used during the actual synthesis (PEO 100 mg + HPL Ru 47.0 mg and PS 100 mg + HPB Ru 66.6 mg). TG-DSC measurements were subsequently performed under Ar atmosphere from room temperature to 900 °C at a heating rate of 10 °C min^{-1} (**Supplementary Fig. 4**).

For the PEO/HPL Ru system, the thermal decomposition behavior of the composite differed substantially from those of pure PEO and pure HPL Ru. The TG profile of the composite could not be described as a simple superposition of the individual components and exhibited distinct mass-loss events at temperatures different from those of either precursor alone. Consistent changes were also observed in the DSC profile, where the characteristic thermal transitions of both PEO and HPL Ru shifted upon composite formation. In particular, the thermal event observed for HPL Ru around 200 °C shifted to higher temperature in the PEO/HPL Ru composite, whereas the characteristic thermal transition of PEO near 400 °C shifted to lower temperature. Such simultaneous shifts in the thermal responses of both components further support the presence of appreciable molecular interactions between HPL Ru and PEO.

Similarly, the PS/HPB Ru composite exhibited markedly different TG and DSC behaviors compared with those of pure PS and HPB Ru. In particular, the sharp thermal event observed for HPB Ru around 100 °C disappeared in the composite, while the thermal transition associated with PS shifted to lower temperature. These pronounced changes in both TG and DSC profiles similarly support the presence of appreciable molecular interactions between HPB Ru and PS.

Importantly, these observations provide experimental support for the molecular interaction calculations and MD simulations presented in **Supplementary Note 1**. The experimentally observed changes in thermal behavior indicate that the precursor molecules interact with the corresponding polymer domains rather than behaving independently within the polymer matrix, providing experimental support for the precursor-polymer interactions underlying the partitioning behavior proposed in this work.

In addition, the proposed partitioning mechanism is consistent with previous reports employing block copolymer-directed self-assembly. Ruthenium chloride hydrate (HPL Ru) has been widely used in block copolymer systems because of its favorable interactions with PEO domains, enabling the synthesis of ordered mesoporous materials (*Nat. Commun.*, **2023,** 14, 4182, *Nat. Commun.,* **2026**, 17, 3752, *Small*, **2022**, 18, 2104204). Likewise, hydrophobic organometallic precursors have been shown to preferentially associate with hydrophobic PS domains during block copolymer self-assembly (*ACS Nano*, **2012**, 6, 6870–6881, *ACS Nano*, **2013**, 7, 1036–1044). Notably, Bis(2-methylallyl)(1,5-cyclooctadiene)ruthenium(II) (HPB Ru) has also been successfully incorporated into mesostructured materials through interactions with hydrophobic PS domains during one-pot synthesis (*J. Mater. Chem. A*, **2015**, 3, 23725–23731). Therefore, both the additional TG-DSC analyses and the established literature precedents support the precursor-partitioning mechanism proposed in this work.

Revision

To experimentally validate the precursor-polymer interactions underlying the molecular interaction calculations and MD simulations, additional thermogravimetric analysis with differential scanning calorimetry (TGA-DSC) measurements were performed using solvent-cast precursor/polymer composites. The thermal behaviors of the precursor/polymer composites were compared with those of the corresponding individual polymers and Ru precursors. Based on these additional analyses, a new supplementary note discussing the precursor-polymer interactions and their relevance to the proposed precursor-partitioning mechanism has been added.

(Page 6 in the revised Manuscript)

The predicted precursor-polymer interactions were further supported by thermogravimetry analysis with differential scanning calorimetry (TGA-DSC) measurements, which revealed distinct thermal behaviors for the precursor/polymer composites compared with the corresponding individual components (Supplementary Note 2).

(Page 2-3 in the Supplementary Note 2 of the revised Supplementary Information)

Supplementary Note 2 | TG-DSC analysis of precursor-polymer interactions

To experimentally investigate the precursor-polymer interactions underlying the molecular interaction analysis and MD simulations, TGA-DSC (Thermogravimetry analysis with differential scanning calorimetry) analyses were performed using solvent-cast precursor/polymer composites. For the measurements, PEO and HPL Ru were completely dissolved in ethanol, while PS and HPB

Ru were completely dissolved in THF, followed by solvent evaporation at 50 °C. The precursor-to-polymer ratios were identical to those used during the synthesis of the block copolymer-directed Ru ALN/WC_x catalysts. TGA-DSC measurements were conducted under Ar atmosphere from room temperature to 900 °C at a heating rate of 10 °C min^{-1} (Supplementary Fig. 4).

For the PEO/HPL Ru system, the TG profile of the composite exhibited thermal decomposition behavior distinct from those of pure PEO and pure HPL Ru. The composite could not be described as a simple superposition of the individual components and displayed characteristic mass-loss events at temperatures different from those of either constituent. Consistent changes were also observed in the DSC profile. In particular, the thermal event associated with HPL Ru near 200 °C shifted to higher temperature in the composite, whereas the thermal event associated with PEO near 400 °C shifted to lower temperature. These simultaneous changes indicate the presence of appreciable molecular interactions between HPL Ru and PEO.

A similar tendency was observed for the PS/HPB Ru system. Compared with pure PS and pure HPB Ru, the PS/HPB Ru composite exhibited markedly different TG and DSC behaviors. The sharp thermal event observed for HPB Ru near 100 °C disappeared upon composite formation, while the thermal transition associated with PS shifted to lower temperature. These pronounced changes in thermal behavior support the presence of appreciable molecular interactions between HPB Ru and PS.

The observed thermal behavior demonstrates that the precursor molecules do not behave independently within the polymer matrix but instead interact with the corresponding polymer domains. These results provide experimental support for the molecular interaction calculations and MD simulations presented in Supplementary Note 1 and are consistent with the proposed precursor-partitioning mechanism during the block copolymer self-assembly process.

The observed precursor-polymer interactions are also consistent with previous reports employing block copolymer-directed self-assembly. Ruthenium chloride hydrate (HPL Ru) has been widely utilized in block copolymer systems because of its favorable interactions with PEO domains, enabling the synthesis of ordered mesoporous materials (*Nat. Commun.,* **2023,** 14:4182, *Nat. Commun.,* **2026,** 17:3752, *Small,* **2022,** 18, 2104204). Likewise, hydrophobic organometallic precursors have been reported to preferentially associate with hydrophobic PS domains during block copolymer self-assembly (*ACS Nano,* **2012,** 6, 6870-6881, *ACS Nano,* **2013,** 7, 1036-1044). In particular, HPB Ru has been successfully incorporated into mesostructured materials through interactions with hydrophobic polymer domains during one-pot synthesis (*J. Mater. Chem. A*, **2015,** 3, 23725-23731). Therefore, both the TG-DSC analyses and the established literature precedents support the precursor-partitioning mechanism proposed in this work.

(Page 22 in the revised Supplementary Information)

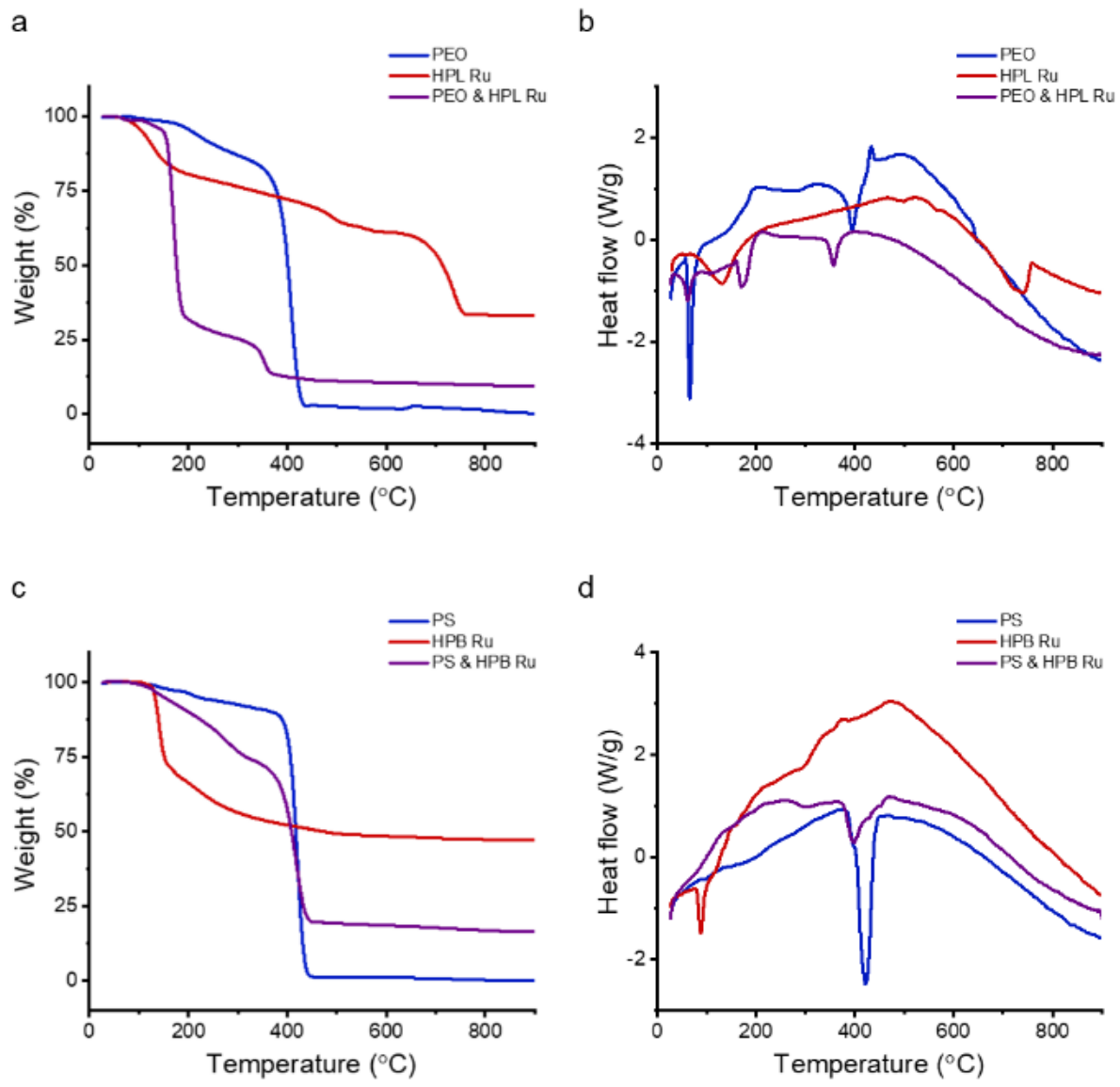

Supplementary Fig. 4 | TG-DSC analyses of polymer-precursor interactions. a,b, Thermogravimetric (TG) and differential scanning calorimetry (DSC) profiles of PEO, HPL Ru precursor, and solvent-cast PEO/HPL Ru composite. **c,d,** TG and DSC profiles of PS, HPB Ru precursor, and solvent-cast PS/HPB Ru composite.

(Page 24 in the *Experimental* section of the revised Manuscript)

For simultaneous TGA-DSC analysis, solvent-cast precursor/polymer composites were prepared by dissolving PEO and HPL Ru in ethanol or PS and HPB Ru in THF, followed by solvent evaporation at 50 °C. Simultaneous thermogravimetric analysis and differential scanning calorimetry (TGA-DSC) were performed using a LABSYS Evo thermal analyzer (Setaram). The samples were heated from room temperature to 900 °C at a heating rate of 10 °C min^{-1} under Ar flow.

5. The manuscript highlights superior durability against Fe/Ni deposition compared to Pt/C but the mechanism is unclear. Clarifying where the impurities go (using high-res EDS mapping or other techniques) would reveal the true mechanism of this durability.

Reply

First, we sincerely apologize for any confusion caused by our previous description regarding the degradation mechanism and Fe/Ni deposition behavior during AEMWE operation.

We would like to clarify that we did not intend to claim that the superior durability of aRu3/WC_x originates from suppression of Fe/Ni deposition. In fact, as shown in **Supplementary Fig. 134**, Fe/Ni species were also deposited on the aRu3/WC_x cathode after 100 h AEMWE durability test, similar to the commercial Pt/C and Ru/C cathodes (**Supplementary Figs. 132, 133**). Therefore, Fe/Ni crossover and redeposition were observed for all cathodes investigated in this work.

Instead, the key distinction is that the aRu3/WC_x catalyst largely preserved the atomic-scale Ru structure on the WC_x support even after prolonged operation (**Supplementary Figs. 134, 135, Supplementary Note 13**), whereas commercial Pt/C and Ru/C catalysts underwent severe nanoparticle agglomeration under identical AEMWE conditions (**Supplementary Figs. 132, 133**). HAADF-STEM images and corresponding EDS elemental mappings after the durability test (**Supplementary Figs. 132-134**) revealed pronounced nanoparticle aggregation for Pt/C and Ru/C together with Fe/Ni deposition, while aRu3/WC_x maintained highly dispersed Ru species with only minor and localized aggregation. The EDS elemental mappings further indicate that the crossover-derived Fe/Ni species are broadly redeposited on all investigated cathode catalyst layers and may partially contribute to active site blocking and performance degradation. Nevertheless, the substantially different durability behaviors among the investigated catalysts correlate more strongly with the degree of nanoparticle migration and agglomeration, suggesting that suppression of catalyst agglomeration is the dominant factor differentiating the superior long-term stability of aRu3/WC_x from conventional nanoparticle catalysts.

Importantly, this interpretation is consistent with a recent report by Kwon and co-workers (*ACS Energy Lett.*, **2025**, 10, 3647-3654), which demonstrated that the initial degradation of AEMWEs primarily originates from cathodic instability rather than anodic degradation. In particular, their operando three-electrode analyses identified Pt nanoparticle agglomeration as the dominant contributor to the rapid increase in cathodic kinetic and residual overpotentials, whereas impurity deposition alone did not fully explain the degradation behavior. Their study further showed that Fe/Cr deposition occurred under both stable and unstable cathodic environments, while the degradation trend more strongly correlated with Pt agglomeration behavior. These findings strongly support our interpretation that structural instability of nanoparticle catalysts is the primary origin of the severe degradation observed for commercial Pt/C and Ru/C cathodes.

To further support that cathode degradation is primarily governed by structural instability of the nanoparticles, we additionally provide reviewer-only data obtained using a commercial Pt/C cathode coupled with a stainless-steel felt anode, which is known to exhibit high electrochemical and

structural stability under AEMWE conditions. This configuration minimizes the contribution from anode degradation and allows the observed durability trend to be attributed mainly to changes within the cathode catalyst layer. Under these controlled conditions, the Pt/C cathode still exhibited significant voltage decay (**Fig. R1a, b**). Post-mortem TEM analysis further revealed pronounced morphological changes in the catalyst layer. While the pristine Pt/C catalyst showed well-dispersed Pt nanoparticles on the carbon support, the end-of-test sample exhibited clear nanoparticle detachment, carbon exposure, and significant Pt agglomeration (**Fig. R1c, d**). These results further support that cathodic nanoparticle instability can serve as a dominant degradation pathway even when the contribution from anode degradation is minimized.

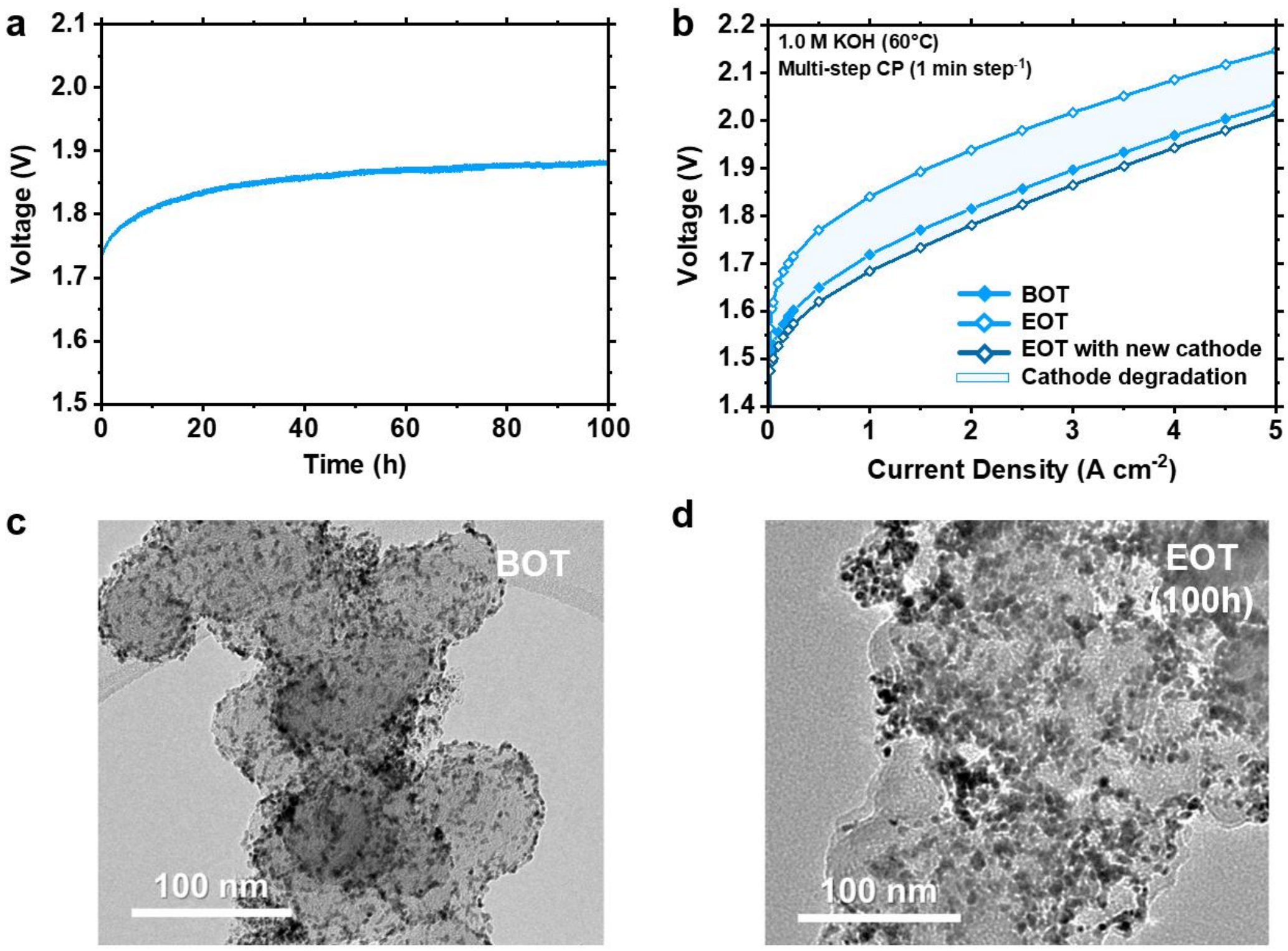

Fig. R1 | Cathode degradation behavior in AEMWEs using commercial Pt/C. (**a**) Voltage profile during the 100 h durability test at 1.0 A cm^{-2} in 1.0 M KOH at 60 ℃. (**b**) Polarization curves measured before the durability test (BOT), after the durability test (EOT), and after replacement of the degraded cathode with a fresh Pt/C cathode while maintaining the same cell components. TEM images of the commercial Pt/C catalyst at (**c**) the beginning-of-test (BOT) and (**d**) at the end-of-test (EOT) conditions.

Note. These data are part of an independent study currently under preparation and are therefore not included in the present manuscript. They are provided here as reviewer-only information to further clarify the cathode degradation behavior observed in our AEMWE testing system.

Consistently, in our work, the significantly improved durability of aRu3/WC_x is closely associated with the strong metal-support interaction (SMSI) between the Ru ALNs and WC_x supports, which effectively suppresses Ru migration and aggregation during prolonged AEMWE operation. This interpretation is further supported by the S-number analysis, where aRu3/WC_x exhibited an S-number approximately five times higher than Pt/C and nine times higher than Ru/C (**Fig. 6h**), indicating substantially suppressed noble-metal degradation and dissolution during 100 h durability test. The substantially smaller voltage increase observed for aRu3/WC_x during both 100 h and 500

h durability test (**Fig. 6g, j**) is therefore attributed primarily to the preserved atomic scale Ru active sites enabled by the SMSI-stabilized Ru ALN structure.

We agree with the reviewer that Fe/Ni redeposition remains an important issue for achieving highly durable practical AEMWE systems. However, because these impurities originate mainly from anode dissolution and subsequent crossover through the membrane/electrolyte interface, mitigation of Fe/Ni contamination requires coordinated advances in anode catalyst stability, membrane engineering, and overall cell-component durability in addition to cathode catalyst design. Therefore, while the present work focuses on stabilizing cathode active sites through SMSI between Ru ALN and WC_x supports, further system-level engineering will also be important for minimizing impurity crossover in practical long-term AEMWE operation.

To avoid further misunderstanding, we have revised the related discussions in the revised Manuscript and Supplementary Information to more clearly distinguish the effects of Fe/Ni deposition from the intrinsic structural stabilization effect of the Ru ALN/WC_x system.

Revision

Additional discussions were added in the revised Manuscript and Supplementary Information to clarify the dominant degradation mechanism during AEMWE operation and to more clearly distinguish the effects of Fe/Ni redeposition from noble metal migration, aggregation, and dissolution. The revised text emphasizing the role of the strong metal-support interaction (SMSI) between Ru ALNs and WCx supports in suppressing Ru aggregation and degradation is highlighted in green.

(Page 19 in the revised Manuscript)

The post-durability analyses of AEMWE operation revealed that both commercial Pt/C and Ru/C cathodes underwent severe NP agglomeration along with noticeable Fe/Ni deposition, originating from the migration and reduction of dissolved Fe/Ni ions from the anode (Supplementary Figs. 132, 133). The aRu3/WC_x cathode also exhibited Fe/Ni deposition (Supplementary Fig. 134), indicating that Fe/Ni redeposition is a common system-level degradation factor in AEMWE operation rather than a catalyst-specific phenomenon (*ACS Energy Lett.*, **2025**, 10, 3647-3654). Although such Fe/Ni redeposition may partially contribute to active-site blocking and performance degradation, the markedly different durability behaviors among the investigated cathodes correlate more strongly with the degree of noble metal migration, aggregation, and dissolution. In this regard, aRu3/WC_x preserved the atomic structure of Ru species on the WC_x supports, showing only minor and localized Ru agglomeration even after prolonged operation (Supplementary Figs. 134, 135, Supplementary Note 13). These results indicate that the strong metal-support interaction between Ru ALNs and WC_x supports effectively suppresses Ru migration and aggregation, thereby providing

superior cathode durability compared with commercial nanoparticle catalysts. It should be noted that further mitigation of Fe/Ni redeposition requires advances in anode and membrane design beyond cathode catalyst optimization (Supplementary Note 13).

(Page 18 in the Supplementary Note 13 of the revised Supplementary Information)

As previously reported by Kwon and co-workers, both impurity redeposition and cathodic nanoparticle instability contribute to the initial performance degradation commonly observed in AEMWE systems (*ACS Energy Lett.*, **2025**, 10, 3647-3654). In particular, their operando three-electrode analyses identified Pt nanoparticle agglomeration as the dominant contributor to the rapid increase in cathodic kinetic and residual overpotentials during the early operation stage, whereas impurity deposition alone did not fully account for the degradation behavior.

(Page 18 in the Supplementary Note 13 of the revised Supplementary Information)

although Fe/Ni redeposition may partially contribute to active site blocking and initial performance degradation in AEMWEs, the substantially different durability behaviors among the investigated cathodes correlate more strongly with the degree of noble metal migration, aggregation, and dissolution.

6. The authors propose a dual-site mechanism (*H on Ru, *OH on WC). However, whether *H adsorbed on Ru spill over to the WC support is unclear. This could dynamically alter the active site density. The authors should comment on whether this pathway exists and contributes to the high activity.

Reply

We thank the reviewer for this insightful comment regarding the possible involvement of hydrogen spillover in the enhanced HER activity of the Ru ALN/WC_x system. We would first like to clarify that the mechanism proposed in our manuscript is not based on hydrogen spillover (H-spillover) from Ru ALN to WC_x followed by H_2 evolution on the support surface. Instead, our interpretation is that Ru ALNs serve as the primary *H adsorption and H_2 evolution sites, while the OH-enriched WC_x support facilitates proton-transfer kinetics from the bulk electrolyte to the electrode-electrolyte interface through modulation of the interfacial hydrogen-bond (H-bond) network.

As discussed in our manuscript and previous reports (*Nat. Catal.*, **2022**, 5, 900-911, *Angew. Chem. Int. Ed.*, **2025**, 64, e202415447), alkaline HER kinetics can become sluggish because depletion of interfacial "gap water" disrupts the H-bond network required for efficient proton transfer through the Grotthuss mechanism. In this framework, oxygenated species such as surface -OH groups can enhance H-bond connectivity between bulk water and the interfacial region, thereby accelerating proton transfer from the bulk electrolyte to the electrode-electrolyte interface and facilitating HER at nearby Ru active sites. In our system, we propose that the OH-enriched WC_x support facilitates interfacial proton transfer by enhancing H-bond network connectivity, while H_2 evolution itself predominantly occurs on the Ru ALN sites.

Nevertheless, as suggested by the reviewer, we agree that the possibility of H-spillover contributing to the HER process cannot be completely excluded; however, our results suggest that it is unlikely to be the dominant origin of the enhanced HER activity.

According to previous studies on H-spillover-based HER electrocatalysts (*Acc. Chem. Res.*, **2024**, 57, 895-904), the HER pathway generally involves (i) Volmer adsorption of *H on metal sites, (ii) spillover of *H from the metal to the support surface, and (iii) Heyrovsky-type H_2 evolution on the support. Such systems typically exhibit characteristic Tafel slopes close to ~20–25 mV dec^{-1} owing to the H-spillover-based HER kinetics (*Acc. Chem. Res.*, **2024**, 57, 895-904, *Nat. Commun.*, **2021**, 12:3502, *Appl. Catal. B environ.*, **2024**, 341, 123275, *Angew. Chem. Int. Ed.*, **2024**, 63, e202315633, *Nat. Commun.*, **2022**, 13:1189). However, aRu3/WC_x exhibited a substantially larger Tafel slope of 41.9 mV dec^{-1} (**Supplementary Fig. 91**), indicating HER kinetics distinct from those typically associated with H-spillover-based HER electrocatalysts.

In addition, previous reports have shown that H-spillover-based electrocatalysts often exhibit pronounced electrochemical hydrogen desorption features originating from large populations of spilled-over *H species on the support surface, together with characteristic scan-rate-dependent *H desorption behavior in *operando* cyclic voltammetry (CV) measurements (*ACS Energy Lett.*, **2022**, 7, 1330-1337, *Appl. Catal. B Environ.,* **2024**, 341, 123275). In contrast, aRu3/WC_x did not exhibit

any noticeable hydrogen desorption peak in CV measurements over a wide scan-rate range (**Supplementary Fig. 108**), suggesting that substantial accumulation of spilled-over hydrogen species on the WC_x support does not occur in our system.

Therefore, although H-spillover-related contributions cannot be completely ruled out, the electrochemical characteristics of aRu3/WC_x are not consistent with those typically observed in H-spillover-dominated HER electrocatalysts. Instead, our results more strongly support a mechanism in which the WC_x support primarily enhances interfacial proton-transfer kinetics through H-bond network modulation, while the Ru ALNs remain the principal HER-active sites for hydrogen adsorption and H_2 evolution. Therefore, the available kinetic and electrochemical evidence consistently indicates that H-spillover is unlikely to be the dominant origin of the enhanced HER activity in aRu3/WC_x.

Revision

To clarify that the enhanced HER activity of aRu3/WC_x does not primarily originate from a hydrogen-spillover-dominated mechanism, we additionally discussed the electrochemical characteristics of aRu3/WC_x in comparison with previously reported hydrogen-spillover-based HER electrocatalysts. Specifically, we analyzed the Tafel kinetics and electrochemical hydrogen desorption behavior of aRu3/WC_x and clarified that the WC_x support mainly promotes interfacial proton-transfer kinetics through H-bond network modulation, while the Ru ALNs remain the principal HER-active sites for hydrogen adsorption and H_2 evolution.

(Page 15 in the revised Manuscript)

The electrochemical characteristics of aRu3/WC_x are also distinct from those typically reported for hydrogen-spillover-dominated HER electrocatalysts (*Acc. Chem Res.,* **2024**, 57, 895-904). Previous studies on hydrogen-spillover-based HER electrocatalysts have generally reported characteristic Tafel slopes of ~20−25 mV dec^{-1} associated spillover-assisted HER kinetics (*Nat. Commun.*, **2021**, 12:3052, *Appl. Catal. B: Environ.,* **2024**, 341, 123275), whereas aRu3/WC_x exhibited a larger Tafel slope of 41.9 mV dec^{-1} (Supplementary Fig. 91). In addition, hydrogen-spillover-based electrocatalysts often exhibit pronounced electrochemical hydrogen desorption features originating from substantial populations of spilled-over hydrogen species on the support surface (*Appl. Catal. B: Environ.,* **2024**, 341, 123275, *ACS Energy Lett.,* **2022**, 7, 1330-1337). In contrast, aRu3/WC_x did not exhibit any noticeable hydrogen desorption peak in CV measurements (Supplementary Fig. 108), suggesting that substantial accumulation of spilled-over hydrogen species on the WC_x support does not occur in our system. Taken together, these results support a mechanism in which the WC_x support primarily enhances interfacial proton-transfer kinetics through H-bond network modulation, while the Ru ALNs remain the principal HER-active sites for hydrogen adsorption and H_2 evolution.

To further summarize the proposed alkaline HER mechanism, we additionally clarified the respective roles of the WC_x support and Ru ALNs in regulating interfacial proton-transfer kinetics and hydrogen evolution.

(Page 12 in the revised Supplementary Information)

Taken together, these results indicate that the WC_x support primarily enhances proton-transfer kinetics from the bulk electrolyte to the electrode-electrolyte interface by improving interfacial H-bond network connectivity, while the Ru ALNs remain the principal HER-active sites for hydrogen adsorption and H_2 evolution.

To further support the discussion regarding the absence of dominant hydrogen-spillover behavior, we additionally provided scan-rate-dependent cyclic voltammetry analyses for electrochemical hydrogen desorption behavior of aRu3/WC_x.

(Page 126 in the revised Supplementary Information)

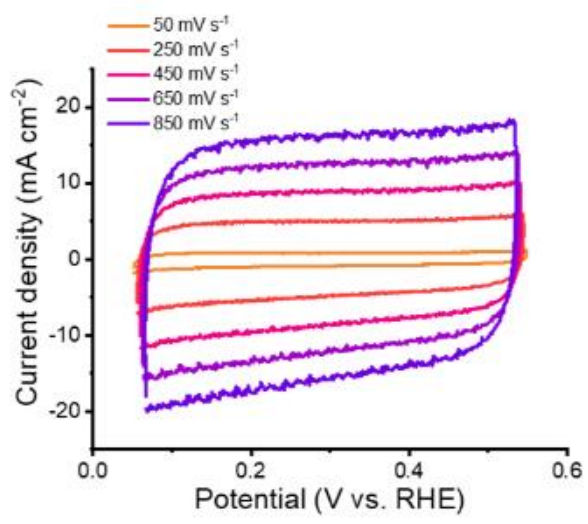

Supplementary Fig. 108 | Electrochemical *H desorption behavior of aRu3/WC_x. CV profiles of aRu3/WC_x catalyst with the scan rate from 50 to 850 mV s^{-1} in Ar-saturated 1.0 M KOH.

Minor comments:

1.Please revise the layout of the figures in the main manuscript to enhance the readability, figures in the current form are too crowded.

Reply

We thank the reviewer for this helpful comment. To enhance readability and improve the overall presentation quality, the figures throughout the revised manuscript were substantially reorganized. The panel layouts were redesigned with increased white space, clearer visual hierarchy, improved alignment, and simplified visual presentation to reduce the crowded appearance and facilitate interpretation of the structural and mechanistic data.

In particular, the original Figure 1 was divided into revised Figures 1 and 2 to improve figure navigation and presentation clarity. The original Figure 2 was reorganized as revised Figure 3 with simplified WT-EXAFS visualizations and improved panel arrangement for clearer presentation of the XAS analyses. In addition, the *in-situ* spectroscopy figure was reorganized as revised Figure 5 with improved layout organization, consistent color presentation, and the addition of a difference contour map derived from the *in-situ* Raman spectra to more clearly visualize the potential-dependent spectral evolution and dynamic interfacial changes during electrochemical operation.

Revision

The figures throughout the revised manuscript were substantially reorganized to improve readability, white space, panel alignment, visual hierarchy, and overall presentation clarity.

The original Figure 1 was divided into revised Figures 1 and 2 with reorganized layouts and clearer visual hierarchy for improved navigation of the conceptual and structural characterization data.

(Page 4 in the revised Manuscript)

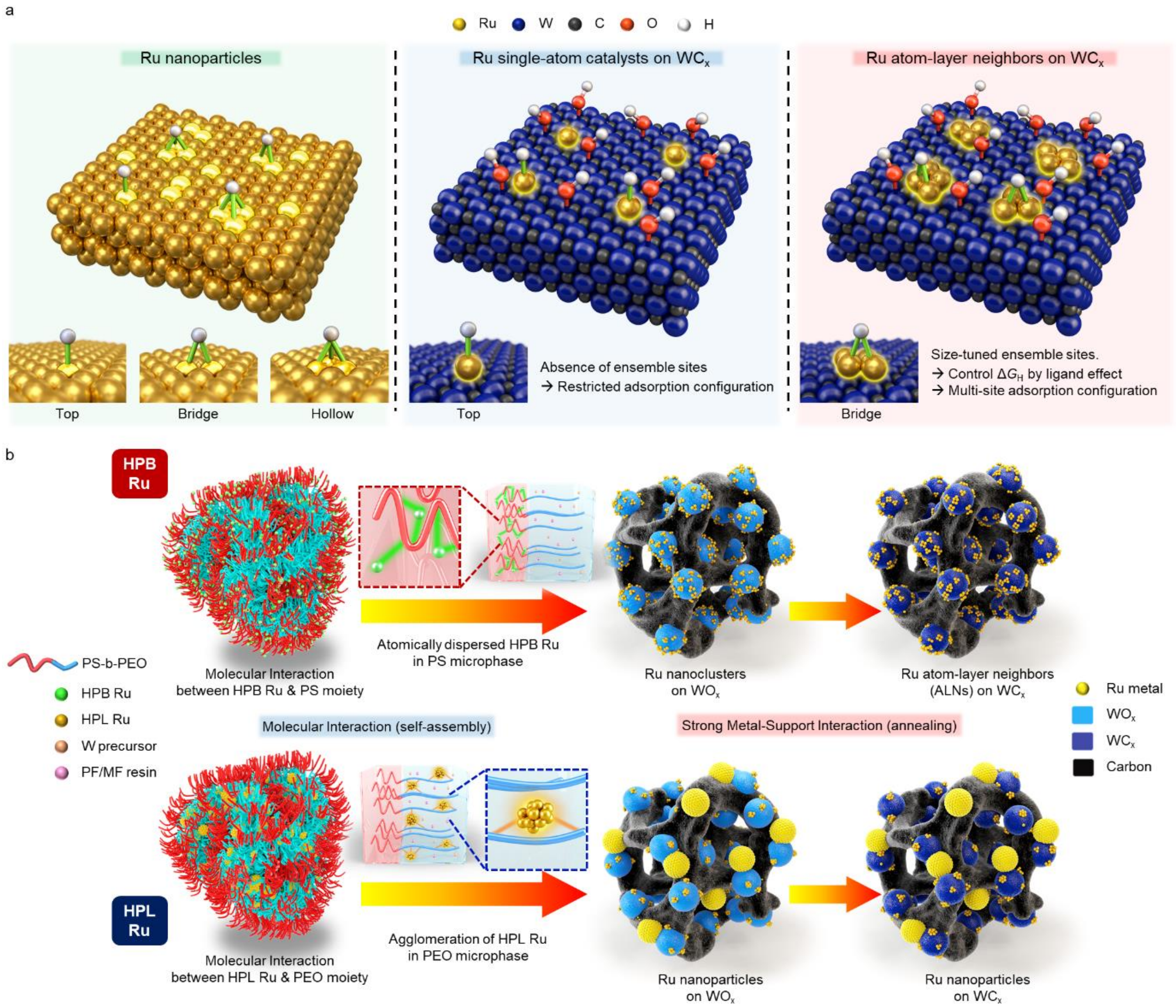

Fig. 1 | Concept of atom-layer neighbors and synthesis strategy via evaporation-induced self-assembly. a, Schematic comparison of Ru nanoparticle (NP) catalysts, Ru single-atom catalysts (SACs), and Ru atom-layer neighbors (ALNs). **b,** Schematic illustration of the thermodynamic stabilization strategy and the differences arising from varying BCP-precursor systems.

(Page 5 in the revised Manuscript)

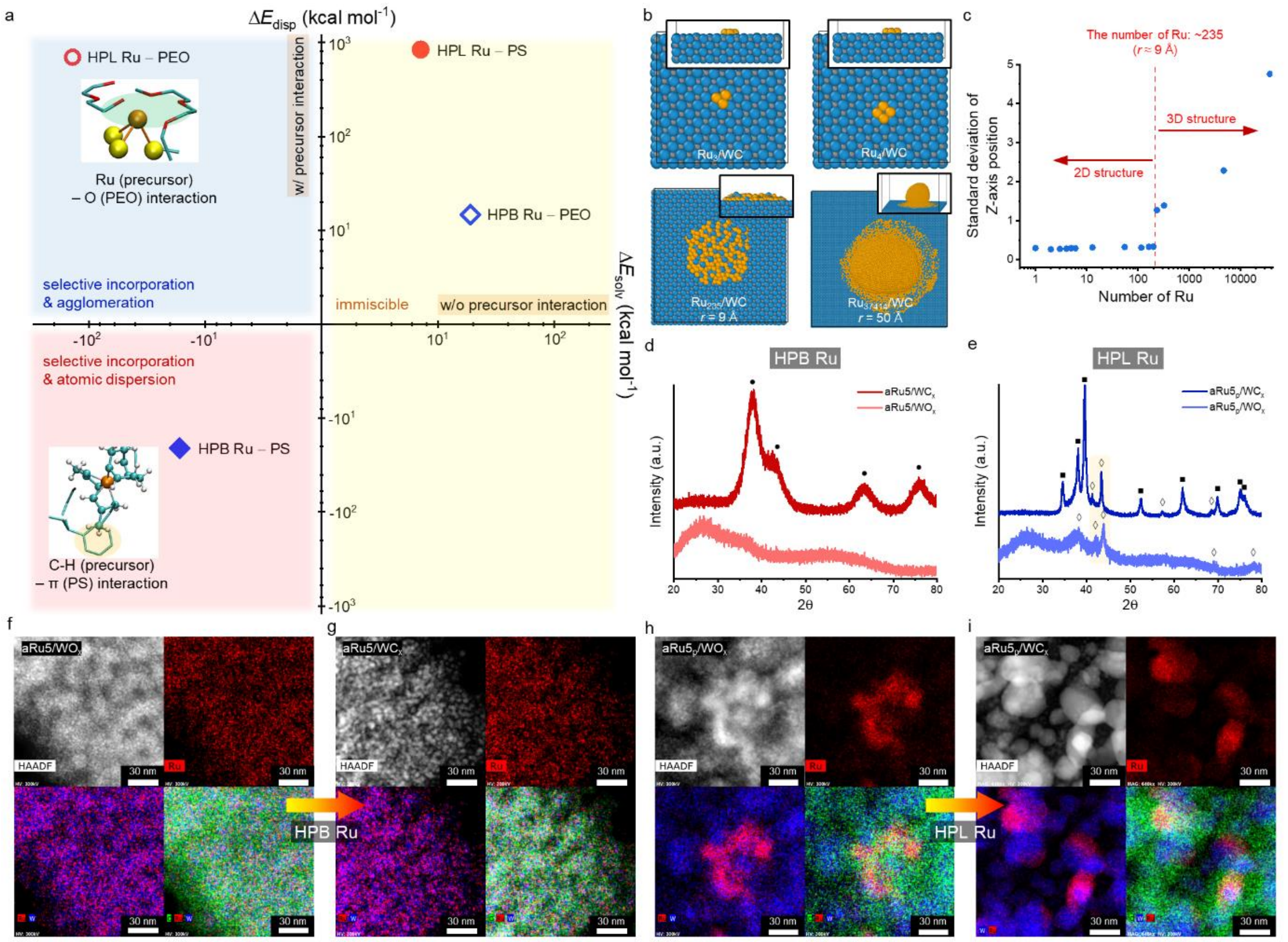

Fig. 2 | Thermodynamic stabilization strategy using molecular interactions (MIs) and strong metal-support interactions (SMSIs). **a,** Relationship between the polymer solvation energy (ΔE_{solv}) and dispersion propensity (ΔE_{disp}) of [Ru precursor], as calculated using MD simulations. **b,** Snapshots from MD simulations of the equilibrated Ru ensembles on WC (Ru_N/WC) structures at 1,200 K (orange: Ru, blue: W, gray: C). **c,** Standard deviation of *Z*-axis positional distribution of Ru species in Ru_N/WC structures as a function of the number of Ru atoms. **d,e,** XRD patterns of (**d**) samples prepared using HPB Ru (aRu5/WO_x and aRu5/WC_x) and (**e**) samples prepared using hydrophilic Ru precursor (HPL Ru) (aRu5$_p$/WO_x and aRu5$_p$/WC_x) (●: γ-WC [PDF 20-1316], ■: α-W_2C [PDF 35-0776], ◊: Ru [PDF 88-1734]). **f-i,** HAADF-STEM images and corresponding EDS mappings of (**f**) aRu5/WO_x, (**g**) aRu5/WC_x, (**h**) aRu5$_p$/WO_x, and (**i**) aRu5$_p$/WC_x (red: Ru, blue: W, green: C).

The original Figure 2 was reorganized as revised Figure 3 with improved panel arrangement and simplified WT-EXAFS visualizations to enhance readability and clarity of the XAS analyses..

(Page 9 in the revised Manuscript)

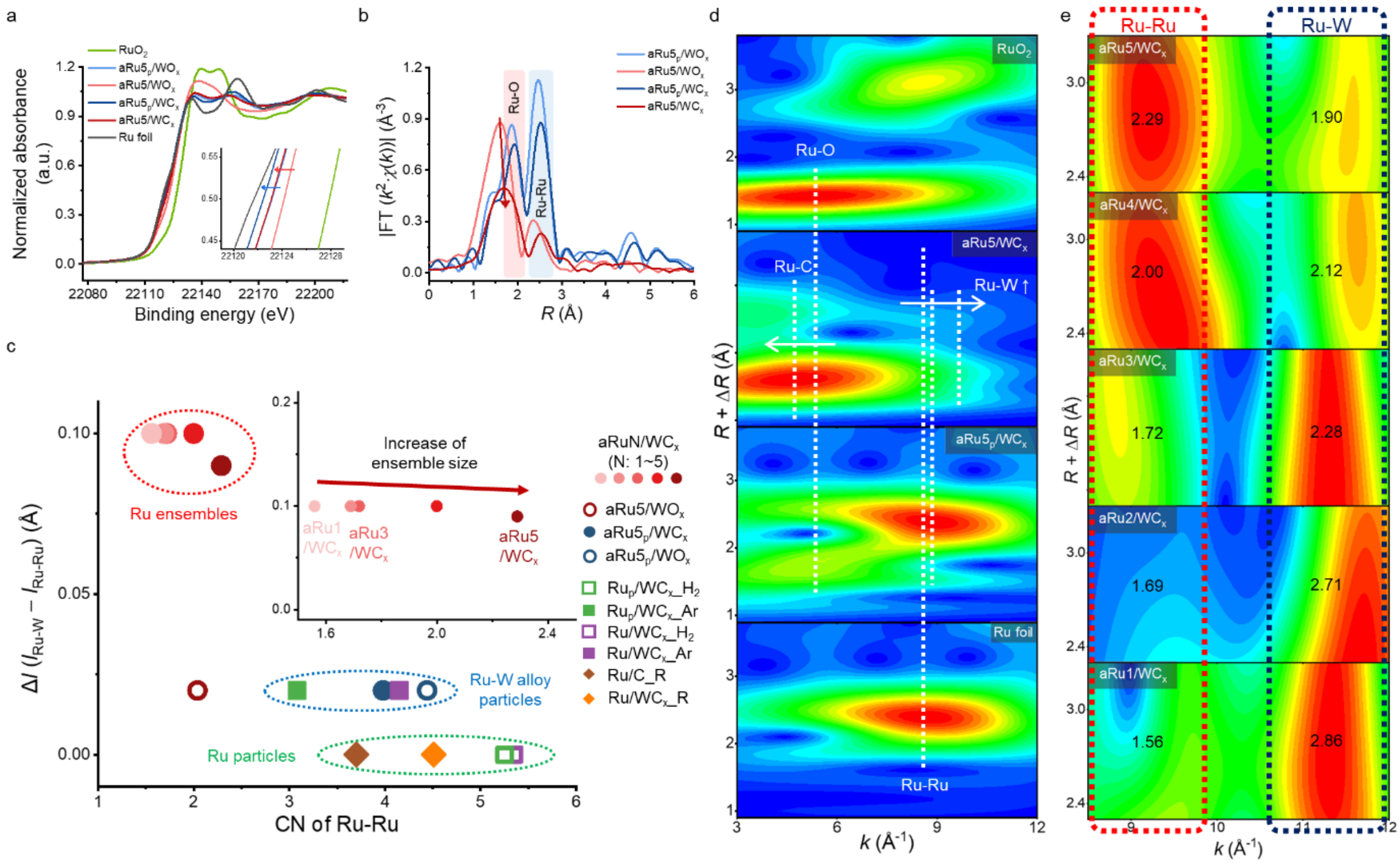

Fig. 3 | Alteration of the geometric and electronic structure of Ru ALNs. a, XANES spectra at the Ru K-edge. **b,** k^2-weighted EXAFS Fourier transform magnitude in R space (χ(R)) at the Ru K-edge of samples prepared using HPL Ru or HPB Ru with different annealing *T* (red shaded region: Ru-O bonds, blue shaded region: Ru-Ru bonds). **c,** Bond length differences between Ru-W and Ru-Ru bonds as a function of the CN of Ru-Ru bonds, based on EXAFS fitting results for the second shell at the Ru K-edge. **d,** Overview wavelet transforms of aRu5/WC_x and aRu5$_p$/WC_x at the Ru K-edge. **e,** Detailed wavelet transform analyses of aRuN/WC_x at the Ru K-edge second-shell region (red region: Ru-Ru bonds, blue region: Ru-W bonds). The inserted numbers indicate the coordination numbers (CNs) obtained from EXAFS fitting.

The *in-situ* spectroscopy figure was reorganized as revised Figure 5 with improved layout organization, consistent color presentation, and the addition of a difference contour map derived from the *in-situ* Raman spectra to improve visualization of the potential-dependent spectral evolution and dynamic interfacial changes during electrochemical operation.

(Page 13 in the revised Manuscript)

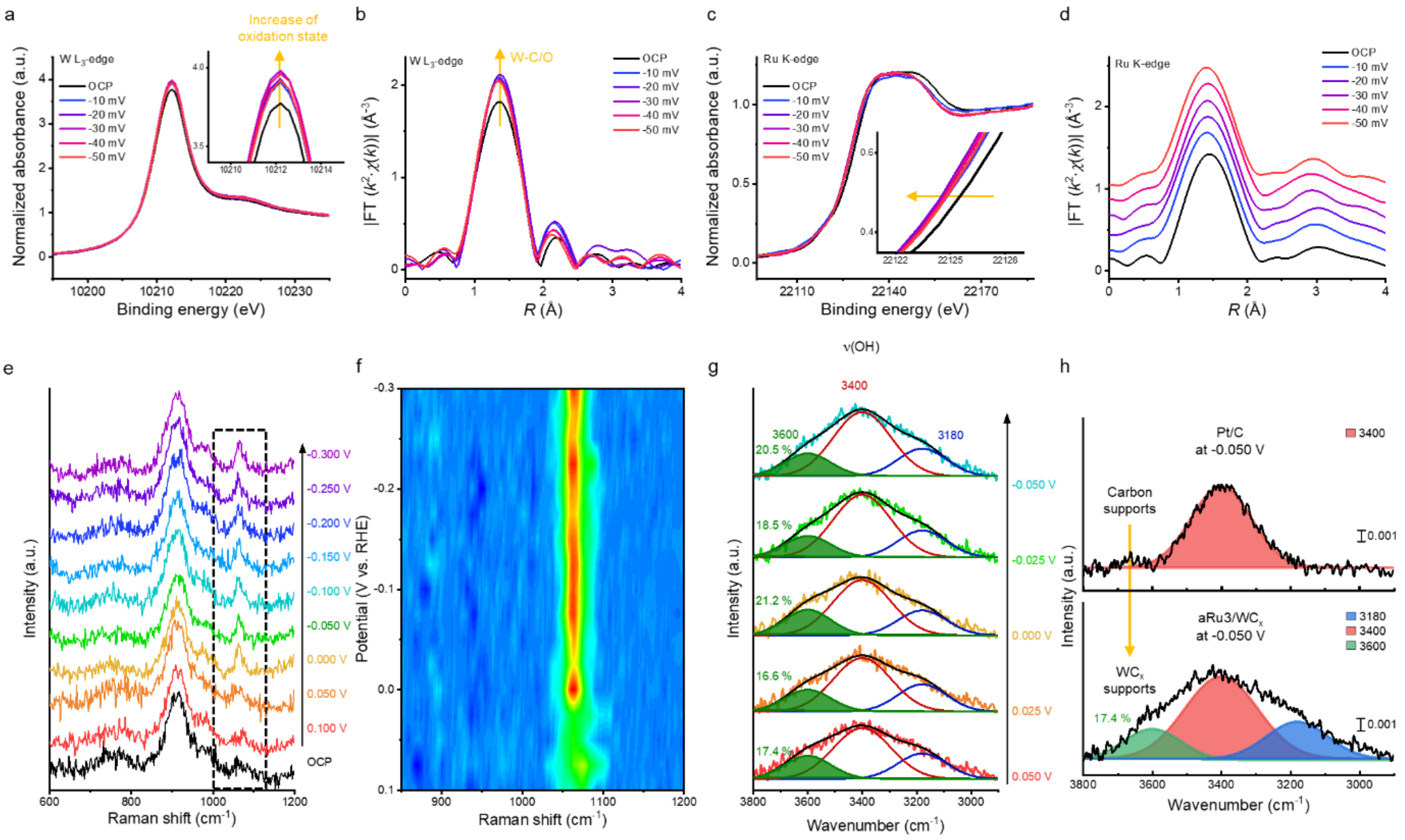

Fig. 5 | Dynamic modulation of the Ru ALNs-WC$_x$ interfacial environment revealed by *in-situ* spectroscopy. a,b, *In-situ* (**a**) XANES and (**b**) corresponding FT-EXAFS spectra at the W L_3-edge of aRu3/WC$_x$ at different potentials. **c,d,** *In-situ* (**c**) XANES and (**d**) corresponding FT-EXAFS spectra at the Ru K-edge of aRu3/WC$_x$ at different potentials. **e,** *In-situ* Raman spectra collected at sequential potentials (green shadow: the coupled O=W⋯W-OH mode (~1060 cm^{-1})). **f**, Difference contour map generated from the *in-situ* Raman spectra, showing the potential-dependent evolution of Raman intensity. **g,** *In-situ* ATR-SEIRAS spectra of aRu3/WC$_x$ at sequential potentials. h**,** Comparison of *in-situ* ATR-SEIRAS spectra at -0.050 V for Pt/C and aRu3/WC$_x$.

2. Please optimize Figure 2 e and f by deleting the contour lines. The spectrums in their current forms are too hard to read and identify.

Reply

We thank the reviewer for this helpful suggestion. To improve readability and visual clarity, the contour lines in the WT-EXAFS spectra have been removed in the revised Manuscript. The WT-EXAFS results in the revised Supplementary Information were also revised consistently. In addition, Figure 2 (now Figure 3) was reorganized to further improve the overall clarity and readability of the WT-EXAFS analyses.

Revision

The contour lines in the WT-EXAFS spectra have been removed in the revised Manuscript and Supplementary Information to improve readability and visual clarity. In addition, Figure 2 (now Figure 3) was reorganized to improve the overall presentation clarity of the WT-EXAFS analyses.

(Page 9 in the revised Manuscript)

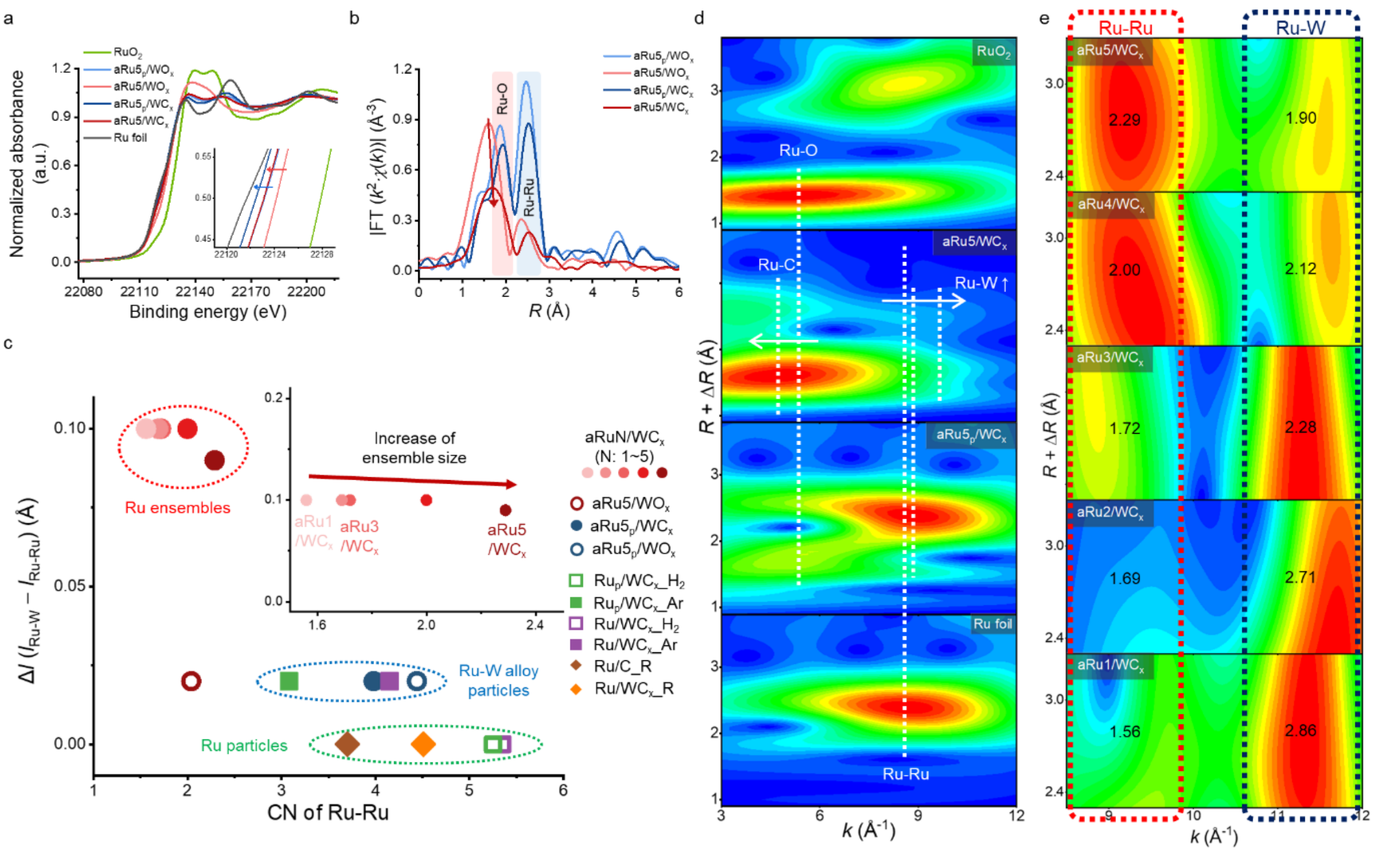

Fig. 3 | Alteration of the geometric and electronic structure of Ru ALNs. a, XANES spectra at the Ru K-edge. **b,** k^2-weighted

EXAFS Fourier transform magnitude in R space (χ(R)) at the Ru K-edge of samples prepared using HPL Ru or HPB Ru with different annealing *T* (red shaded region: Ru-O bonds, blue shaded region: Ru-Ru bonds). **c,** Bond length differences between Ru-W and Ru-Ru bonds as a function of the CN of Ru-Ru bonds, based on EXAFS fitting results for the second shell at the Ru K-edge. **d,** Overview wavelet transforms of aRu5/WC_x and aRu5$_p$/WC_x at the Ru K-edge. **e,** Detailed wavelet transform analyses of aRuN/WC_x at the Ru K-edge second-shell region (red region: Ru-Ru bonds, blue region: Ru-W bonds). The inserted numbers indicate the coordination numbers (CNs) obtained from EXAFS fitting.

(Page 102-103 in the revised Supplementary Information)

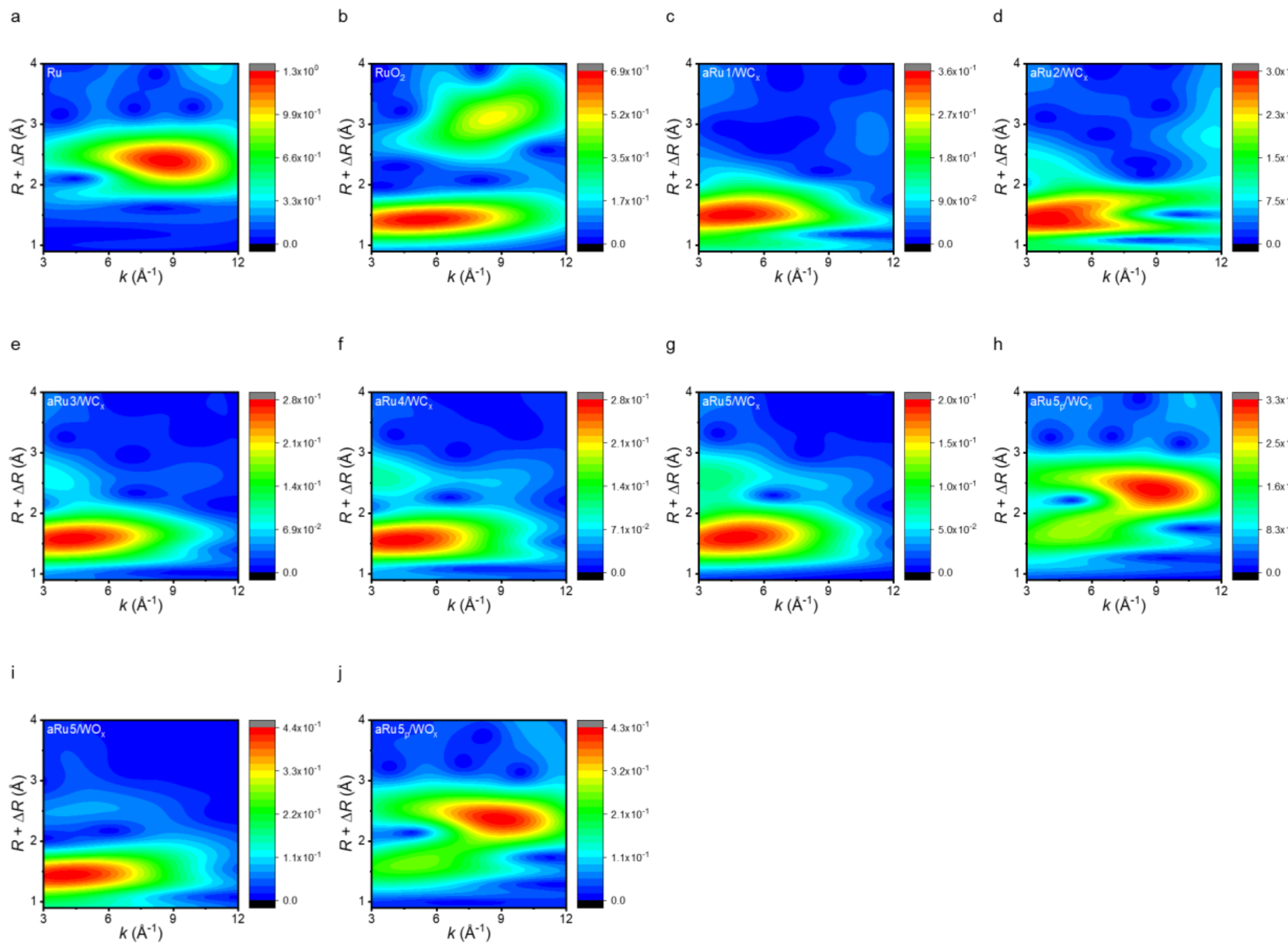

Supplementary Fig. 84 | WT-EXAFS. a-j, WT-EXAFS contour plots for (**a**) Ru foil, (**b**) RuO_2, (**c**) aRu1/WC_x, (**d**) aRu2/WC_x, (**e**) aRu3/WC_x, (**f**) aRu4/WC_x, (**g**) aRu5/WC_x, (**h**) aRu5$_p$/WC_x, (**i**) aRu5/WO_x, and (**j**) aRu5$_p$/WO_x.

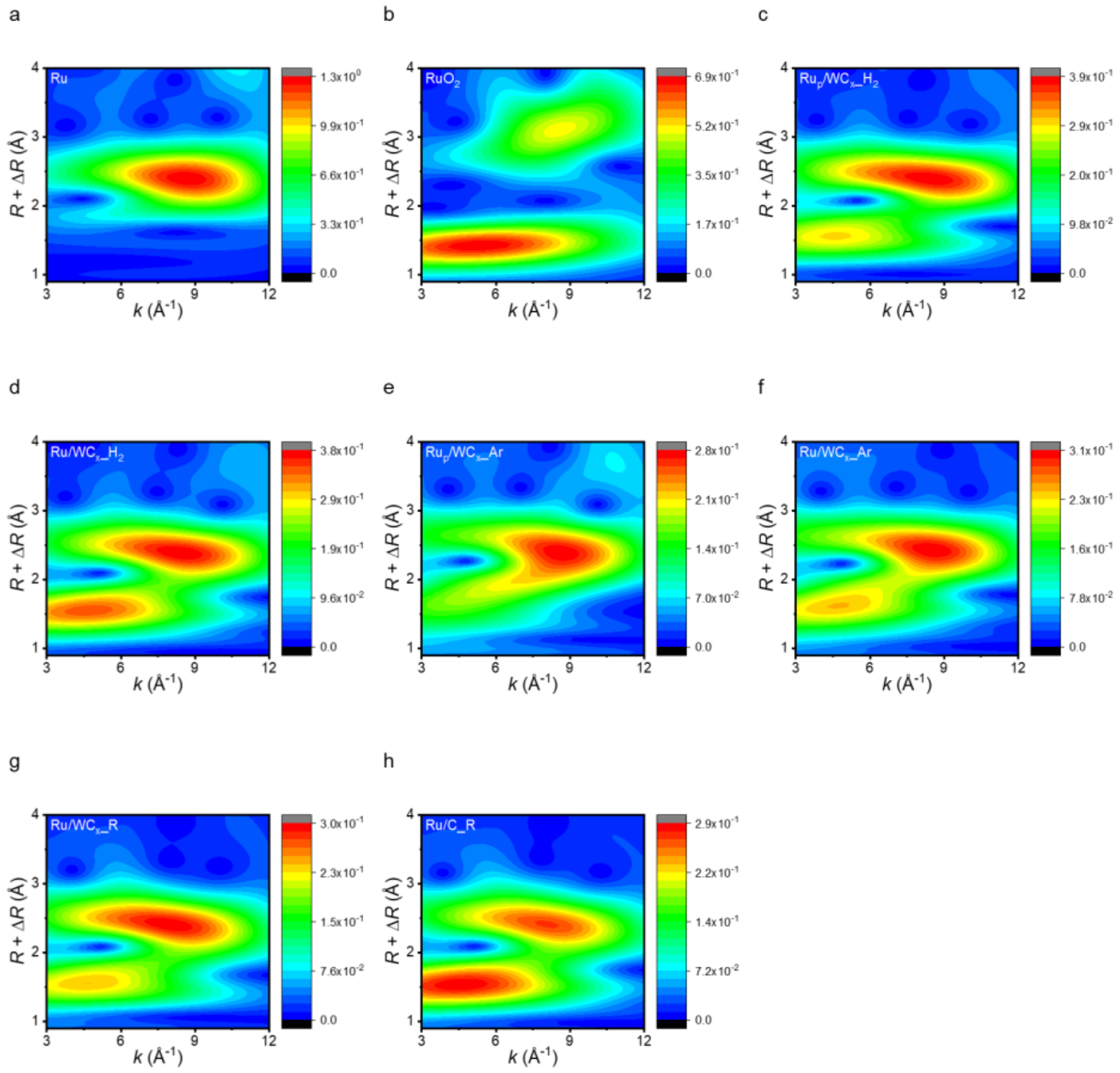

Supplementary Fig. 85 | WT-EXAFS. a-h, WT-EXAFS contour plots for (**a**) Ru foil, (**b**) RuO_2, (**c**) $Ru_p/WC_x\_H_2$, (**d**) $Ru/WC_x\_H_2$, (**e**) Ru_p/WC_x_Ar, (**f**) Ru/WC_x_Ar, (**g**) Ru/WC_x_R, and (**h**) Ru/C_R.

Reviewer #2 (Remarks to the Author):

The recommendation is to accept the manuscript with minor revisions. The paper extensively describes a novel approach for realizing HER catalyst materials for AEMWE by widening the concept of single-atom-catalysts to small ensembles of atoms forming Ru-based atom-layer-neighbours dispersed onto WC_x substrates. Authors in-depth combine computational methods, microscopy, and x-ray-based investigation techniques to completely characterise and optimise the fabrication process as well as to fully characterise the structural and chemical catalyst composition. Furthermore, catalyst activity and stability are investigated and the novel catalyst proposed is compared with state of art catalyst materials, showing remarkable performances in terms of both catalyst activity and stability. The proposed research is meaningful and it is in line with the editorial line of the journal. Nonetheless, before considering the paper for publication, the following points should be refined:

Reply

We Sincerely thank the reviewer for the highly positive and thoughtful evaluation of our work. We particularly appreciate the reviewer's recognition of the novelty and significance of our strategy for extending the concept of single-atom catalysts toward Ru-based atom-layer neighbors (ALNs), as well as the comprehensive combination of computational analysis, advanced microscopy, synchrotron-based X-ray spectroscopies, and electrochemical analyses to elucidate the catalyst structure, synthesis mechanism, and electrocatalytic performance. We are also grateful for the reviewer's positive assessment of the activity and durability of the proposed catalysts and for recognizing the relevance of this work to the scope and standards of *Nature Communications.*

Following the reviewer's valuable suggestions, we have carefully revised the Manuscript to further improve the scientific clarity, logical flow, readability, and overall presentation quality. In particular, we have:

(i) revised and clarified acronyms and terminology throughout the Manuscript,
(ii) improved the logical continuity of the Introduction by incorporating additional discussions on molecular interactions (MIs) and strong metal-support interactions (SMSIs),
(iii) substantially reorganized and redesigned the main figures to improve readability and visual clarity,
(iv) added detailed SAXS experimental descriptions and significantly expanded the SAXS discussion through a new Supplementary Note addressing mesostructural correlations, porosity evolution, and high-*q* scattering behavior,
(v) added the DOE reference and corrected formatting issues throughout the Manuscript, and
(vi) improved the clarity and organization of the Supplementary figures and captions.

We believe that these revisions have significantly strengthened the Manuscript and carefully addressed the reviewer's concerns. All changes are described in detail below.

1. Acronyms should be better defined, capitalizing the proper letters once the acronym is defined.

Reply

We thank the reviewer for this helpful comment. In the revised Manuscript, all acronyms have been carefully checked and revised to ensure that they are properly defined at their first appearance and consistently formatted with appropriate capitalization throughout the revised Manuscript. In addition, less common acronyms were clarified where appropriate, and infrequently used abbreviations were replaced with their full terms to improve readability.

Revision

All acronyms in the revised Manuscript have been carefully checked and revised to ensure proper definition at first appearance and consistent capitalization through the revised Manuscript. In addition, less common acronyms were clarified where appropriate, and infrequently used abbreviations were replaced with their full terms to improve readability.

2. While acronyms of techniques are commonly found and known by the readers, the acronym NM (Noble Metal) sounds odd; to improve manuscript readability, Authors are suggested to replace it with its textual version.

Reply

We thank the reviewer for this helpful suggestion. To improve readability and avoid unnecessary abbreviations, the acronym “NM” has been replaced with the full term “noble metal” throughout the revised Manuscript.

Revision

The acronym “NM” has been replaced with the full term “noble metal” throughout the revised Manuscript to improve readability.

3. At page 3 of the pdf file of the manuscript there is a remarkable logical jump in between the first and the second paragraph. Authors might consider improving this feature, but this advice can be considered optional.

Reply

We appreciate the reviewer's valuable suggestion regarding the logical flow of the Introduction. To improve the logical continuity between the discussion of block copolymer self-assembly and the subsequent introduction of AEMWE electrocatalysis, we revised the Introduction by adding bridging sentences describing the importance of stabilizing size-tuned noble metal ensembles through molecular interactions (MIs) and strong metal-support interactions (SMSIs) for practical electrocatalytic applications.

Revision

To improve the logical continuity between the first and second paragraphs of the introduction, additional sentences describing the stabilization of size-tuned noble metal ensembles through molecular interactions (MIs) and strong metal-support interactions (SMSIs) for practical electrocatalytic applications have been added to the revised Manuscript.

(Page 3 in the revised Manuscript)

Therefore, there is a pressing need for novel platforms that feature size-tuned noble metal ensembles for optimal adsorbate binding while incorporating additional active sites near the primary ones, thereby enabling enhanced catalytic activity and durability under practical operating conditions.

In this regard, strong metal-support interactions (SMSIs) can provide an additional means to stabilize atomic-scale metal species on porous supports. Recent studies, including our previous work on stabilization of noble metal SACs over MoC_x supports, have demonstrated that SMSI-stabilized atomic-scale noble metal catalysts can exhibit enhanced activity and stability even under harsh anion-exchange membrane water electrolyzer (AEMWE) single-cell operation conditions (*Energy Environ. Sci.*, **2025**, 18, 659-673). These findings suggest that integrating MIs with SMSIs may provide an effective strategy for constructing highly active and durable noble metal electrocatalysts through stabilization of size-tuned metal ensembles.

4. In Figure 1a, the first image on the left is hardly readable, as bonds between Ru nanoparticle surface and H atoms are barely distinguishable from the background, and “top”, “bridge”, and “hollow” configurations cannot be properly identified. Image outlook should be improved; moreover, legend arrangement should be improved, if possible.

Reply

We thank the reviewer for this helpful suggestion regarding the readability of Figure 1. To improve readability and visual clarity, the original Figure 1 has been reorganized into separate Figures 1 and 2 in the revised Manuscript. In particular, the schematic image illustrating the adsorption configurations (“top”, “bridge”, and “hollow”) was revised with improved contrast and visibility so that the Ru-H interactions and adsorption geometries can be more clearly distinguished from the background. In addition, the figure legends and overall layout were reorganized to improve readability and presentation clarity.

Revision

The original Figure 1 has been substantially reorganized and separated into revised Figures 1 and 2 to improve readability and presentation clarity. In particular, the schematic illustration of the adsorption configurations was redesigned with enhanced image contrast and visibility so that the “top”, “bridge”, and “hollow” configurations can be more clearly distinguished. Enlarged schematic views were also added to more clearly illustrate the individual adsorption configurations. In addition, the figure legends and overall layout were reorganized accordingly for improved visual clarity.

(Page 4 in the revised Manuscript)

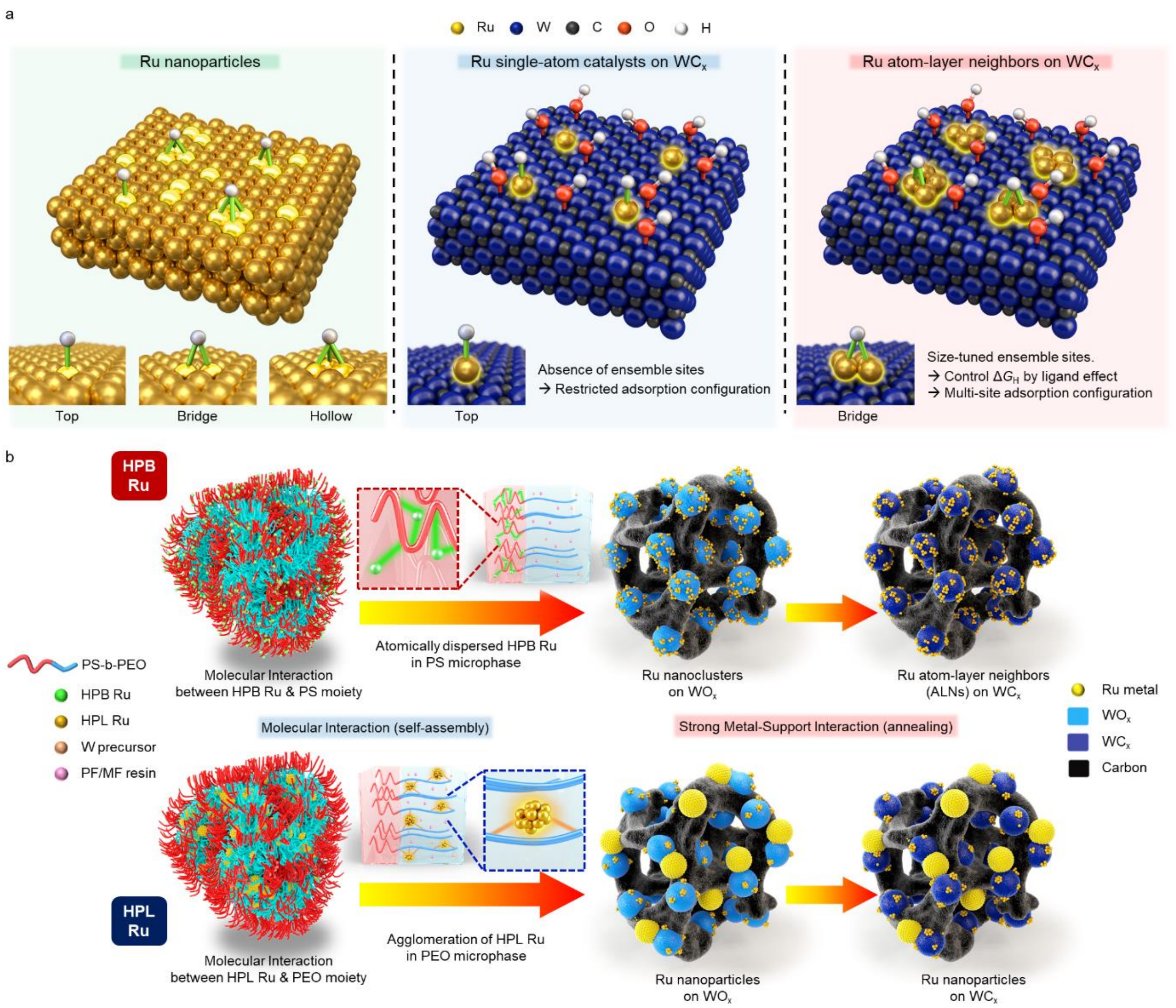

Fig. 1 | Concept of atom-layer neighbors and synthesis strategy via evaporation-induced self-assembly. a, Schematic comparison of Ru nanoparticle (NP) catalysts, Ru single-atom catalysts (SACs), and Ru atom-layer neighbors (ALNs). **b,** Schematic illustration of the thermodynamic stabilization strategy and the differences arising from varying BCP-precursor systems.

(Page 5 in the revised Manuscript)

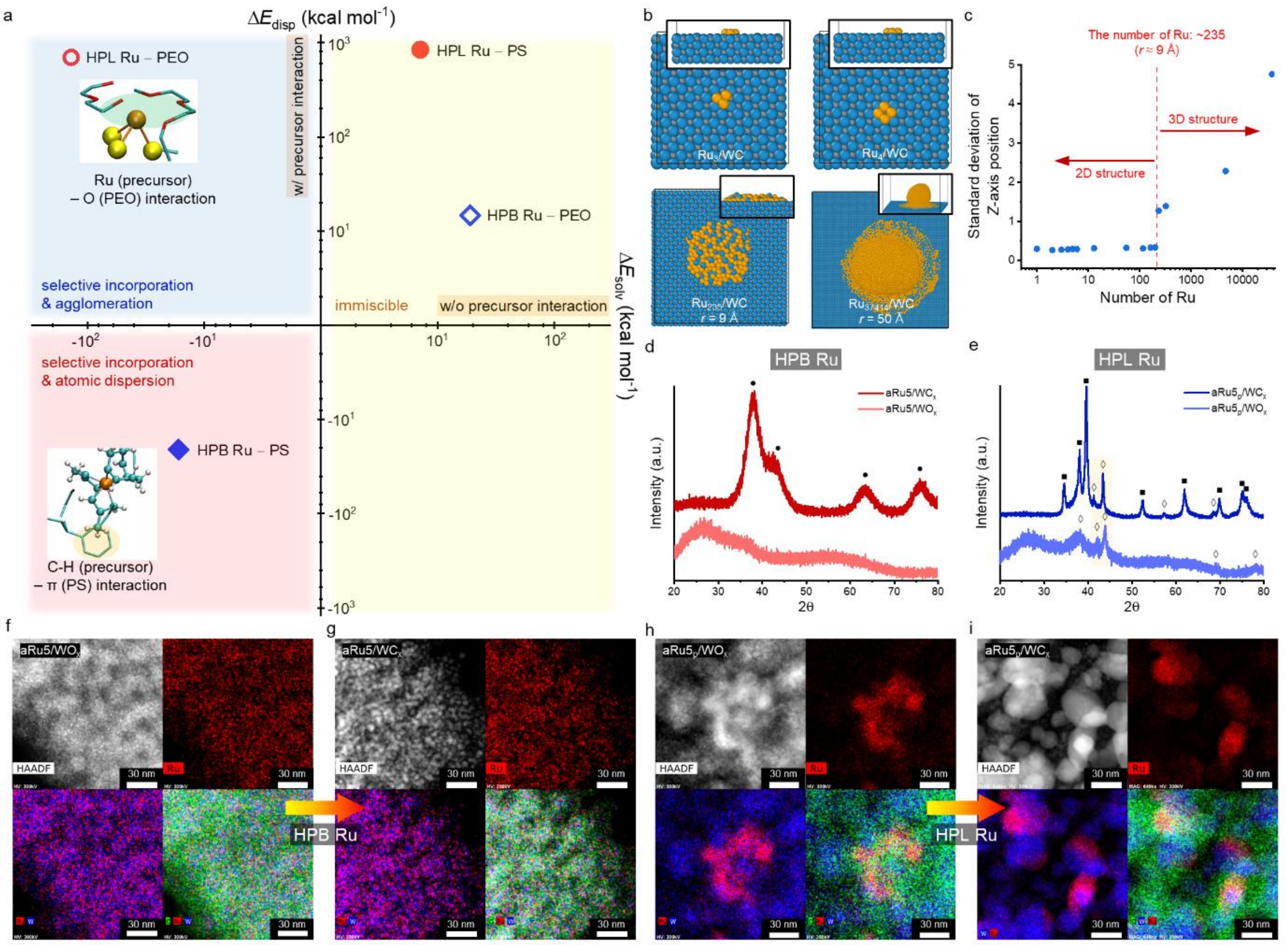

Fig. 2 | Thermodynamic stabilization strategy using molecular interactions (MIs) and strong metal-support interactions (SMSIs). **a,** Relationship between the polymer solvation energy (ΔE_{solv}) and dispersion propensity (ΔE_{disp}) of [Ru precursor], as calculated using MD simulations. **b,** Snapshots from MD simulations of the equilibrated Ru ensembles on WC (Ru_N/WC) structures at 1,200 K (orange: Ru, blue: W, gray: C). **c,** Standard deviation of *Z*-axis positional distribution of Ru species in Ru_N/WC structures as a function of the number of Ru atoms. **d,e,** XRD patterns of (**d**) samples prepared using HPB Ru (aRu5/WO_x and aRu5/WC_x) and (**e**) samples prepared using hydrophilic Ru precursor (HPL Ru) (aRu5$_p$/WO_x and aRu5$_p$/WC_x) (●: γ-WC [PDF 20-1316], ■: α-W_2C [PDF 35-0776], ◊: Ru [PDF 88-1734]). **f-i,** HAADF-STEM images and corresponding EDS mappings of (**f**) aRu5/WO_x, (**g**) aRu5/WC_x, (**h**) aRu5$_p$/WO_x, and (**i**) aRu5$_p$/WC_x (red: Ru, blue: W, green: C).

5. At page 15 of the manuscript, reference document for the DOE target must be provided.

Reply

We thank the reviewer for pointing this out. In the revised Manuscript, the appropriate reference for the U.S. Department of Energy (DOE) technical targets has been added to support the performance benchmark discussed.

Revision

The corresponding DOE reference has been added to the revised Manuscript.

(Page 18 in the revised Manuscript)

More importantly, at 1.8 V_{cell}, aRu3/WC_x reached 4.52 A cm^{-2} at 80 °C and 3.65 A cm^{-2} at milder operating *T* of 60 °C (Fig. 6a) despite its low noble metal loading of 0.10 mg_{Ru} cm^{-2}, exceeding US DOE's 2026 performance target (3.0 A cm^{-2} at 1.8 V_{cell}) and ultimate noble metal loading target (0.125 mg cm^{-2}) for PEMWEs[73].

(Page 36-37 in the *References* section of the revised Manuscript)

73 U.S. Department of Energy. Technical Targets for Proton Exchange Membrane Electrolysis, https://www.energy.gov/eere/fuelcells/technical-targets-proton-exchange-membrane-electrolysis (accessed 31 Mar, 2026)

6. At page 16, section “references” should be moved at the end of the manuscript.

Reply

We thank the reviewer for pointing this out. In the revised Manuscript, the *References* section has been relocated to the end of the revised Manuscript in accordance with the journal format, improving the overall structure and readability.

Revision

The *References* section has been moved to the end of the Manuscript.

7. At page 22, basic SAXS experimental setup details should be provided, such as: energy used, calibrant, and sample to detector distance.

Reply

We thank the reviewer's helpful suggestion regarding the SAXS experimental details. In the revised Manuscript, we have included detailed SAXS experimental parameters in the *Characterizations* section of the *Methods* to improve clarity and reproducibility.

Specifically, SAXS measurements were performed at the 4C SAXS beamline at the Pohang Accelerator Laboratory (PAL), Korea, with an X-ray energy of 10.0 keV. The sample-to-detector distance was set to 4 m. Calibration of the scattering vector (q) was carried out using silver behenate (Tokyo Chemical Industry, TCI) as a standard, while Ti-SBA-15 (Sigma-Aldrich) was used as a structural reference material. The scattered X-rays were collected using a two-dimensional CCD detector (Rayonix SX165, Evanston). The obtained 2D scattering patterns were circularly averaged with respect to the beam center and normalized by the transmitted X-ray beam intensity.

Based on this experimental setup, the SAXS intensity profiles and Kratky plots were replotted within the experimentally accessible q range of 0.67-1.4 nm^{-1} to ensure consistent and reliable comparison of the scattering features among samples.

These details have been added to the revised *Methods* section.

Revision

Detailed SAXS experimental parameters have been added to the *Characterization* section of the *Methods* in the revised Manuscript.

(Page 24 in the *Experimental* section of the revised Manuscript)

Small-angle X-ray scattering (SAXS) measurements were performed at the 4C SAXS beamline at the Pohang Accelerator Laboratory (PAL), Korea. The X-ray energy was set to 10.0 keV, and the sample-to-detector distance was 4 m. The scattering vector (q) was calibrated using silver behenate (Tokyo Chemical Industry, TCI), while Ti-SBA-15 (Sigma-Aldrich) was employed as a structural reference material. Scattered X-rays were recorded using a two-dimensional CCD detector (Rayonix SX165, Evanston). The 2D scattering images were circularly averaged with respect to the beam center to obtain one-dimensional profiles and subsequently normalized to the transmitted X-ray beam intensity. The SAXS intensity profiles and Kratky plots were presented within the experimentally accessible q range of 0.67-1.4 nm^{-1}.

Conventional SAXS intensity profiles ($I(q)$ vs. q) have been replotted within the experimentally accessible q range of 0.67-1.4 nm^{-1}.

(Page 32 in the revised Supplementary Information)

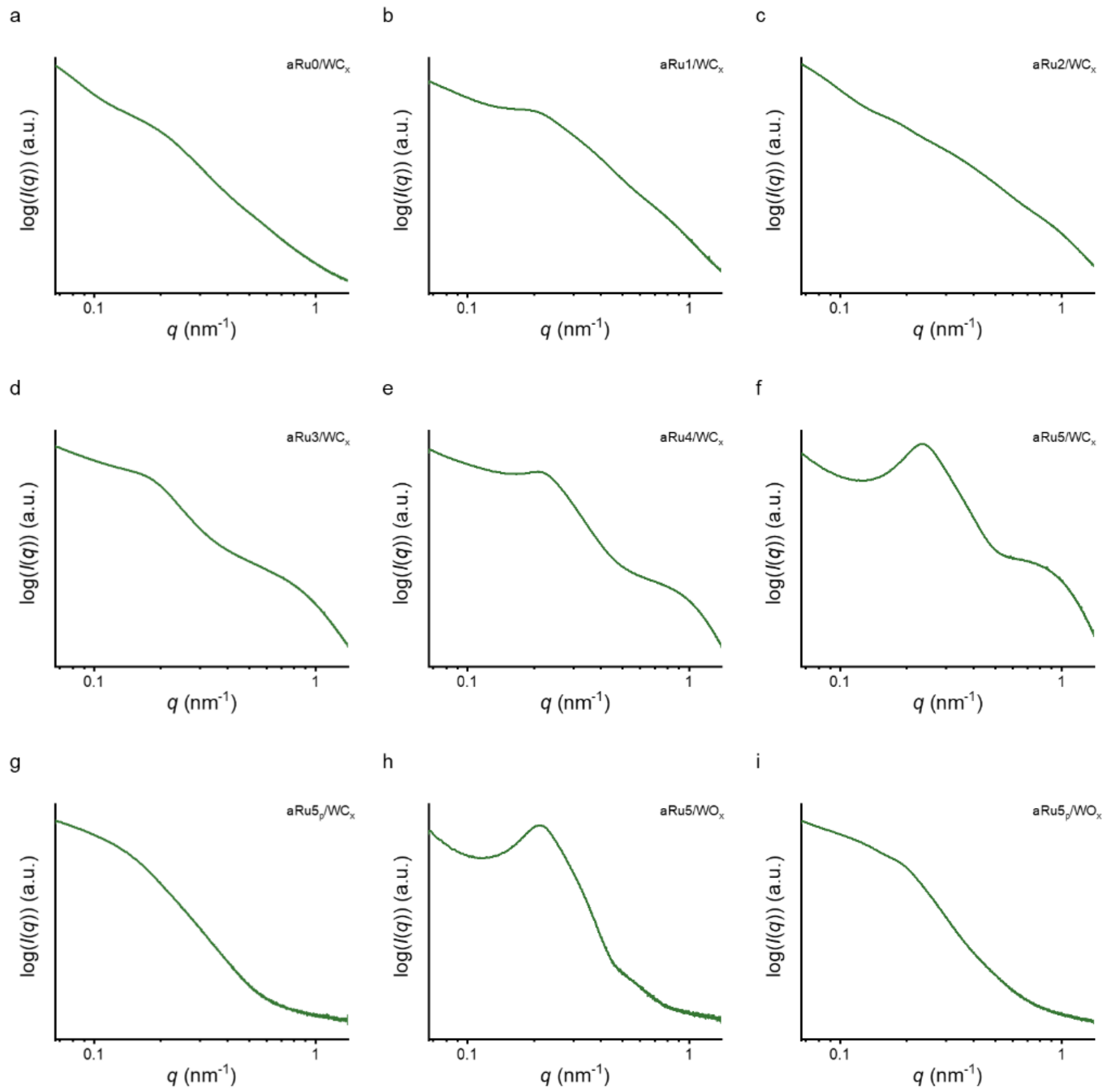

Supplementary Fig. 14 | SAXS profiles. a-i, SAXS intensity profiles (log $I(q)$ vs. q) of samples prepared via self-assembly: (**a**) aRu0/WC_x, (**b**) aRu1/WC_x, (**c**) aRu2/WC_x, (**d**) aRu3/WC_x, (**e**) aRu4/WC_x, (**f**) aRu5/WC_x, (**g**) aRu5_p/WC_x, (**h**) aRu5/WO_x, and (**i**) aRu5p/WO_x**.** The position of the broad scattering maximum (or shoulder) is indicated by line markers.

Kratky plots ($I(q)\cdot q^2$ vs. q) have been replotted within the experimentally accessible q range of 0.67-1.4 nm^{-1}.

(Page 33 in the revised Supplementary Information)

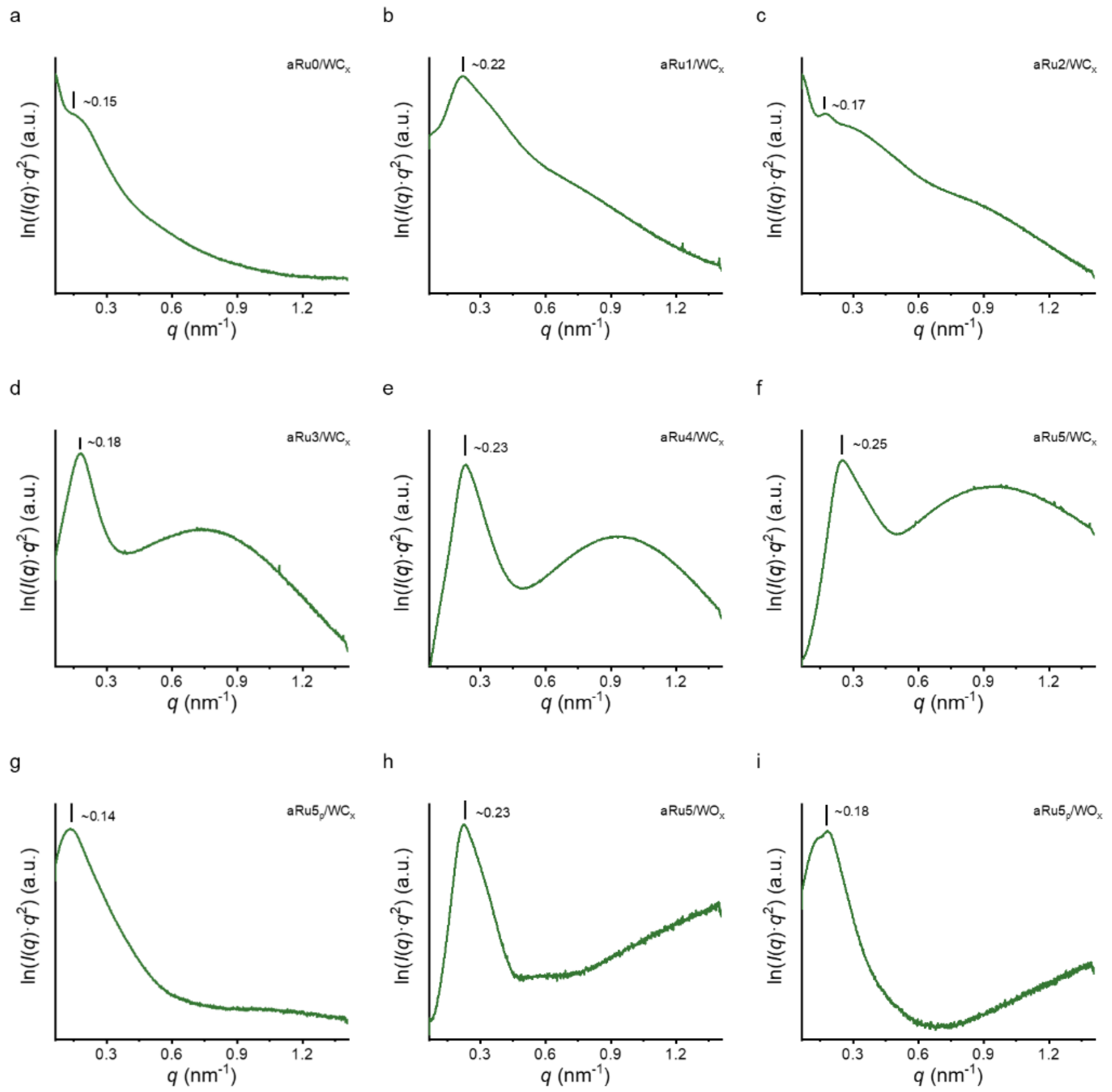

Supplementary Fig. 15 | SAXS analysis. a-i, Kratky plots ($I(q)\cdot q^2$ vs. q) of SAXS data for samples prepared via self-assembly: (**a**) aRu0/WC_x, (**b**) aRu1/WC_x, (**c**) aRu2/WC_x, (**d**) aRu3/WC_x, (**e**) aRu4/WC_x, (**f**) aRu5/WC_x, (**g**) aRu5_p/WC_x, (**h**) aRu5/WO_x, and (**i**) aRu5p/WO_x**.** The position of the broad scattering maximum (or shoulder) is indicated by line markers.

8. In SI, as did for XPS and XAS, SAXS data analysis should be detailed. In fact, SAXS patterns are represented as Kratky plots (I(q) * q^2 vs q) instead of standard scattering pattern (I(q) vs q), but this choice has not been explained. Moreover, Supplementary Figure 13 is show without being fully commented. Basic information, such as: (i) what is represented in the area highlighted by pale yellow, (ii) how different sample porosity reflects on scattering patterns, and (iii) why samples show different evolution at the higher q-values in Kratky plots and why the information in this part of the graph can be neglected, should be qualitatively introduced at least. In these regards, Authors should also apply the same considerations when commenting Supplementary Figure 18, where Kratky plots are directly compared with samples pore size distribution). Authors should comment why SAXS analyses were made via model-free approach instead of using an analytical model for retrieve pore size distribution.

Reply

We thank the reviewer for this insightful comment and apologize for the insufficient description of the SAXS data analysis in the original Manuscript. In the revised Manuscript, we have substantially expanded the description of SAXS analysis and clarified the rationale for employing Kratky plots together with a model-free approach. We particularly appreciate the reviewer's suggestion, as it allowed us to more clearly highlight how the choice of Ru precursor influence the mesostructural correlations, thereby strengthen the overall synthesis rationale.

Explanation for applying Kratky plot with a model-free approach

First, we emphasize that the primary purpose of the SAXS analysis in this study was not to derive exact pore structural parameters through model-dependent fitting, but rather to qualitatively identify mesostructural correlations and to compare relative differences in structural ordering and porosity arising from different Ru precursors and precursor amounts during the EISA process. The resulting WC-carbon nanocomposite framework exhibit predominantly short-range mesostructural ordering, accompanied by broad primary features and weak or no well-defined higher-order reflections (**Supplementary Figs. 14, 15**). Under such conditions, applying analytical models that assume specific pore geometries or long-range periodicity would require strong structural assumptions and could lead to overinterpretation or poorly constrained fitting parameters.

We therefore intentionally adopted a model-free approach to conservatively compare relative trends in mesostructural ordering and pore characteristics across the samples. Consistently, SEM images (**Supplementary Figs. 16, 17**) reveal worm-like interconnected mesoporous frameworks rather than a highly periodic pore lattice, which is in good agreement with the broad SAXS features lacking distinct higher-order reflections (**Supplementary Figs. 14, 15**). Quantitative pore size distributions were independently evaluated by N_2 physisorption measurements (**Supplementary Figs. 8-10**), while SAXS was primarily used to probe structural correlations associated with the self-assembled frameworks. The combined SAXS, SEM, and N_2 physisorption results indicate that the samples consist of worm-like mesoporous frameworks exhibiting short-range mesostructural ordering, rather than a highly periodic pore lattice.

Accordingly, the SAXS data were represented as Kratky plots ($I(q)\cdot q^2$ vs. q) (**Supplementary Fig. 15**), which enhance intermediate-length-scale structural correlations and allow clearer visualization of broad mesostructural features in weakly ordered porous materials (*J. Phys. Chem. B*, **2025**, 130, 171-178), compared with conventional scattering profiles ($I(q)$ vs. q) (**Supplementary Fig. 14**). Nevertheless, the conventional scattering profiles are also provided as complementary data, as they allow a more direct comparison of variations in peak width among samples in our system.

Substitution of pale-yellow area to line indicator

The pale-yellow region in the original figure was used to indicate the broad maximum associated with short-range mesostructural ordering. However, as correctly pointed out by the reviewer, such shading may introduce ambiguity in the interpretation of the SAXS data. To improve clarity, the revised figures instead mark the position of the broad scattering maximum (or shoulder) using line indicators. In addition, the figure captions have been revised to explicitly indicate that the line markers correspond to the positions of the broad scattering maximum (or shoulder) (**Supplementary Figs. 14, 15**).

Correlation between porosity and scattering patterns

In mesostructured systems with worm-like pore frameworks, the position of the SAXS feature (q^*) provides a measure of the characteristic structural spacing, which can be estimated as $d \approx 2\pi/q^*$. This length scale corresponds to the average distance between correlated mesostructural domains rather than an exact pore size. In contrast, the width of the SAXS feature reflects the structural correlation length within the mesoporous framework. Broader features indicate a shorter correlation length and increased structural heterogeneity, whereas narrower features suggest a higher degree of short-range ordering. In the present system, all samples share a similar WC-carbon nanocomposite framework, and thus differences in peak width are primarily attributed to variations in mesostructural ordering and pore uniformity arising from the precursor-dependent self-assembly process.

Samples with low or no contents of HPB Ru (aRu0-2/WC_x) exhibit broader or shoulder-like SAXS features, whereas samples with higher HPB Ru contents (aRu3-5/WC_x) show sharper and more well-defined peaks (**Supplementary Figs. 14, 15**). This indicates that aRu0-2/WC_x possess poorly defined mesostructural features, while aRu3-5/WC_x exhibit improved pore uniformity and enhanced short-range mesostructural ordering. Similarly, aRu3-5/WC_x display narrower pore size distributions in N_2 physisorption compared to aRu0-2/WC_x (**Supplementary Figs. 9, 10**), which is consistent with the more uniform mesostructures observed in SEM images (**Supplementary Figs. 16, 17**). In addition, for aRu3-5/WC_x, the SAXS peak position (q^*) shifts toward higher q-values, indicating a reduction in the characteristic structural spacing (**Supplementary Figs. 14, 15**).

Taken together, these results suggest that above a certain HPB Ru content, the short-range mesostructural ordering is significantly enhanced, accompanied by reduced structural spacing and improved pore uniformity. This evolution in porosity is likely associated with the interactions between HPB Ru and PS moieties, which influence the EISA process and thereby modulates the resulting mesostructural organization.

Samples prepared using HPB Ru (aRu5/WO_x and aRu5/WC_x) exhibit narrower pore size distributions in N_2 physisorption (**Supplementary Fig. 20a, b**) compared to those prepared using HPL Ru (aRu5$_p$/WO_x and aRu5$_p$/WC_x). In addition, HPB Ru-derived samples show narrower SAXS features (**Supplementary Fig. 20c**), indicating improved pore uniformity and enhanced short-range mesostructural ordering relative to the HPL Ru-derived counterparts. These observations suggest that the two different precursors interact preferentially with different blocks of the copolymer, thereby directing the EISA process through distinct block-specific interactions and leading to distinct mesostructural organizations.

During the transformation from WO_x to WC_x, the samples prepared using HPB Ru exhibit relatively minor changes in both pore size distribution and SAXS peak position (q^*), indicating that the mesostructural framework is largely preserved. In contrast, the samples prepared using HPL Ru show more pronounced changes, including substantial variation in pore size distribution and a shift in q^*, suggesting a greater degree of structural rearrangement during the carburization process (**Supplementary Fig. 20**).

Taken together, these results indicate that the chemical affinity of the Ru precursor toward different polymer blocks governs their interactions with the block copolymer, thereby influencing the EISA process and ultimately affecting the porosity evolution and structural integrity during the WO_x-to-WC_x transformation.

Origin of different evolution at high-q region

At higher q-values ($q > \sim 0.6\ nm^{-1}$), both the SAXS profiles and Kratky plots (**Supplementary Figs. 14, 15**) exhibit noticeable differences in their evolution, particularly for aRu3-5/WC_x. This high-q region probes shorter length-scale features within the framework and is distinct from the low-q region, which mainly reflects mesostructural correlations associated with characteristic structural spacing.

First, the high-q features can be distinguished from mesostructural periodicity. As confirmed by the SEM images and N_2 physisorption results (**Supplementary Figs. 8-10, 16, 17**), all samples consist of worm-like interconnected mesoporous frameworks rather than highly periodic pore lattices. Consistently, the SAXS profiles do not show well-defined higher-order reflections, indicating that long-range periodicity is limited. Therefore, the high-q evolution is unlikely to originate from higher-order mesostructural reflections, but instead reflects localized structural features within the WC-carbon framework.

Importantly, the composite nature of the WC-carbon framework provides a key origin for the distinct high-q behavior. In SAXS, scattering arises from electron-density contrast; however, the appearance of a distinct feature at high q is governed not only by the magnitude of this contrast but also by its spatial distribution at the nanoscale (*Macromol*, **2005**, 38, 3395-3405). XRD analysis (**Supplementary Fig. 21**) shows that increasing Ru content (*i.e.*, increasing N in aRuN/WC_x) promotes the formation of γ-WC via a Ru-induced auto-catalytic effect (*Chem. Mater.*, **2004**, 16, 307-314). As γ-WC exhibits lower crystallinity than α-W_2C and δ-WC, its formation suppresses the growth of large carbide crystallites and instead favors the formation of smaller and more uniformly dispersed WC domains, as supported by SEM and STEM analyses (**Supplementary Figs. 16, 17, 22-29**).

Such uniformly distributed nanoscale WC domains embedded within the carbon framework create a more homogeneous electron-density contrast at short length scales, which can give rise to a more clearly defined high-q feature. In contrast, larger and less uniformly distributed carbide agglomerates broaden the distribution of local length scales, resulting in weaker and more diffuse high-q scattering.

Consistently, samples with higher atomically dispersed Ru content (aRu3-5/WC_x) exhibit more pronounced high-q features (**Supplementary Figs. 14, 15**), which can be attributed to the combined effects of smaller WC domain size, improved dispersion, and more uniform local electron-density contrast within the WC-carbon framework. In contrast, aRu5_p/WC_x, despite having a similar total Ru content to aRu5/WC_x, shows less pronounced high-q evolution (**Supplementary Fig. 20**). This is attributed to a lower fraction of atomically dispersed Ru, leading to a weaker auto-catalytic effect, reduced formation of γ-WC, and the presence of larger, less uniformly distributed WC agglomerates. Consequently, the reduced uniformity in local electron-density contrast suppresses the high-q feature.

Taken together, the distinct high-q behavior is attributed to nanoscale structural uniformity within the WC-carbon framework, including WC domain size, dispersion, and the spatial uniformity of local electron-density contrast, rather than differences in long-range mesostructural ordering. These results indicate that the high-q region contains meaningful structural information related to local nanoscale heterogeneity and should not be neglected, but rather properly interpreted in terms of local structural features.

To address the reviewer's comments, we have added a new Supplementary Note (**Supplementary Note 5**) that provides a comprehensive description of the SAXS analysis, including the rationale for employing Kratky plots, clarification of peak representation, detailed correlation between porosity and scattering features, and the origin of the high-q behavior.

Revision

The SAXS analysis has been substantially revised and expanded, and a new section has been added to the revised Supplementary Information.

(Page 5-7 in the Supplementary Note 5 of the revised Supplementary Information)

Supplementary Note 5 | Small-angle X-ray scattering analysis of mesostructural correlations as a function of Ru precursor chemistry and content

Small-angle X-ray scattering (SAXS) analyses were performed to investigate mesostructural correlations and relative variations in structural ordering and porosity as a function of Ru precursor chemistry and content during the EISA process (*Biodesign*, **2017**, 5, 24-29). Conventional scattering profiles (intensity ($I(q)$) vs. q) for samples prepared via self-assembly are presented (Supplementary Fig. 14) to compare the evolution of scattering features among the samples. Scanning electron microscopy (SEM) images (Supplementary Figs. 16, 17) reveal interconnected worm-like

mesoporous frameworks rather than highly periodic pore lattices, which is consistent with the broad SAXS features and the absence of distinct higher-order reflections.

To further enhance intermediate-length-scale structural correlations and visualize broad mesostructural features in weakly ordered porous frameworks, the SAXS data were additionally represented as Kratky plots ($I(q)\cdot q^2$ vs. q) (Supplementary Fig. 15) (*J. Phys. Chem. B*, **2025**, 130, 171-178). The WC-carbon nanocomposite frameworks exhibit predominantly short-range mesostructural ordering with broad primary scattering features and weak or poorly resolved higher-order reflections. Under such conditions, analytical fitting models based on ideal pore geometries or long-range periodicity would require strong structural assumptions and may lead to poorly constrained fitting parameters. Therefore, a model-free approach was adopted to conservatively compare relative variations in mesostructural ordering and pore characteristics.

In worm-like mesostructured systems, the SAXS peak position (q^*) reflects the characteristic structural spacing according to $d \approx 2\pi/q^*$, whereas the peak width reflects the structural correlation length within the mesoporous framework. Narrower features generally indicate improved short-range ordering and more uniform mesostructural organization. Since all samples share similar WC-carbon composite frameworks, variations in peak width are mainly attributed to differences in mesostructural organization arising from precursor-dependent self-assembly.

Samples with low or no HPB Ru contents (aRu0-2/WC_x) exhibit broad or weakly defined SAXS features, whereas samples with higher HPB Ru contents (aRu3-5/WC_x) show sharper and more clearly resolved peaks (Supplementary Figs. 14, 15). Consistently, aRu3-5/WC_x exhibit narrower pore size distributions in N_2 physisorption measurements (Supplementary Figs. 9, 10) and more uniform mesostructures in SEM images (Supplementary Figs. 16,17). In addition, the SAXS peak position gradually shifts toward higher q-values from aRu3/WC_x to aRu5/WC_x, indicating a reduction in characteristic structural spacing. These results suggest that increasing HPB Ru content promotes improved short-range mesostructural ordering and pore uniformity during the EISA process. Such behavior is likely associated with preferential interactions between HPB Ru and the PS moieties of the block copolymer, which influence mesostructural organization during self-assembly.

Samples prepared using HPB Ru (aRu5/WO_x and aRu5/WC_x) also exhibit narrower SAXS features and pore size distributions than those prepared using HPL Ru (aRu5$_p$/WO_x and aRu5$_p$/WC_x) (Supplementary Fig. 20), indicating improved pore uniformity and structural ordering. During the WO_x-to-WC_x transformation, HPB Ru-derived samples show relatively minor changes in both SAXS peak position and pore size distribution, whereas HPL Ru-derived samples exhibit more pronounced variations, including decreases in q^*, suggesting greater structural rearrangement during carburization. These results indicate that precursor chemistry influences the EISA process through distinct block-specific interactions with the copolymer, thereby affecting porosity evolution and structural integrity during carburization.

At higher q-values (q > ~0.6 nm^{-1}), both the SAXS profiles and Kratky plots exhibit noticeable differences in scattering evolution, particularly for aRu3-5/WC_x (Supplementary Figs. 14, 15). This high-q region probes localized structural features at shorter length scales rather than mesoscopic periodicity. As confirmed by the SEM images (Supplementary Figs. 16, 17) and N_2 physisorption results (Supplementary Figs. 8-10), all samples consist of

interconnected worm-like mesoporous frameworks rather than highly periodic pore lattices. In addition, the SAXS profiles do not exhibit well-defined higher-order reflections, indicating limited long-range periodicity. Therefore, the high-q features are attributed to localized structural heterogeneity within the WC-carbon framework rather than long-range mesostructural ordering.

In SAXS, scattering originates from electron-density contrast, while the appearance of distinct high-q features is additionally governed by the spatial distribution of this contrast at the nanoscale (*Macromol*, **2005**, 38, 3395-3405). XRD analysis (Supplementary Fig. 21) shows that increasing Ru content promotes the formation of γ-WC via a Ru-induced auto-catalytic effect (*Chem. Mater.*, **2004**, 16, 307-314). Because γ-WC exhibits lower crystallinity than α-W_2C and δ-WC, its formation suppresses the growth of large carbide crystallites and instead favors smaller and more uniformly dispersed WC domains, as supported by SEM and high-angle annular dark-field scanning transmission electron microscopy (HAADF-STEM) analyses (Supplementary Figs. 16, 17, 22-29). Such uniformly distributed nanoscale WC domains embedded within the carbon framework generate more spatially uniform local electron-density contrast at short length scales, giving rise to more clearly defined high-q scattering features. Accordingly, samples with higher atomically dispersed Ru contents (aRu3-5/WC_x) exhibit more pronounced high-q features, which can be attributed to the combined effects of smaller WC domain size, improved dispersion, and more uniform local electron-density contrast within the WC-carbon framework (Supplementary Figs. 14, 15). In contrast, $aRu5_p/WC_x$ exhibits less pronounced high-q evolution despite having similar total Ru content to aRu5/WC_x (Supplementary Fig. 20c). This behavior is attributed to the lower fraction of atomically dispersed Ru, which weakens the auto-catalytic effect and results in larger and less uniformly distributed WC agglomerates. Consequently, the reduced uniformity of local electron-density contrast suppresses the high-q scattering feature. Overall, the distinct high-q behavior is attributed to nanoscale structural uniformity within the WC-carbon framework, including WC domain size, dispersion, and the spatial uniformity of local electron-density contrast, rather than differences in long-range mesostructural ordering.

A sentence describing the effects of Ru precursor chemistry and content on mesostructural correlations and porosity evolution has been added to the revised Manuscript.

(Page 7 in the revised Manuscript)

Consistent with previous reports, these results suggest that the chemical affinity of the Ru precursors govern their preferential interactions with specific BCP microphases during the EISA process, thereby influencing mesostructural correlations, structural ordering and porosity evolution (Supplementary Note 5) (*Biodesign*, **2017**, 5, 24-29, *J. Phys. Chem. B*, **2025**, 130, 171-178, *Macromol*, **2005**, 38, 3395-3405).

In the revised figures, the previously used pale-yellow shaded region has been replaced by line markers indicating the position of the broad scattering maximum (or shoulder) to improve clarity.

(Page 33 in the revised Supplementary Information)

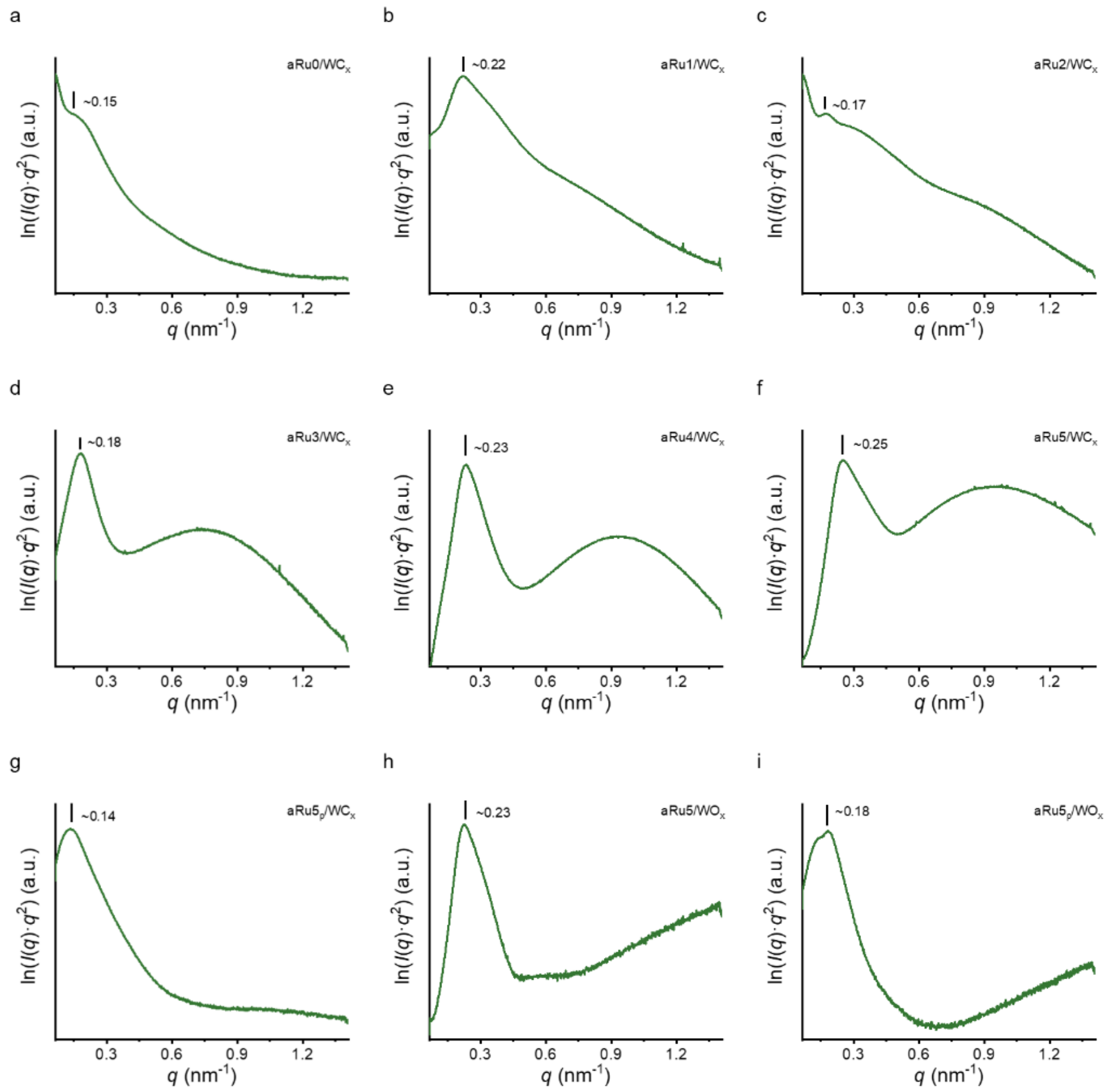

Supplementary Fig. 15 | SAXS analysis. a-i, Kratky plots ($I(q)\cdot q^2$ vs. q) of SAXS data for samples prepared via self-assembly: (**a**) aRu0/WC_x, (**b**) aRu1/WC_x, (**c**) aRu2/WC_x, (**d**) aRu3/WC_x, (**e**) aRu4/WC_x, (**f**) aRu5/WC_x, (**g**) $aRu5_p/WC_x$, (**h**) aRu5/WO_x, and (**i**) aRu5p/WO_x**.** The position of the broad scattering maximum (or shoulder) is indicated by line markers.

(Page 38 in the revised Supplementary Information)

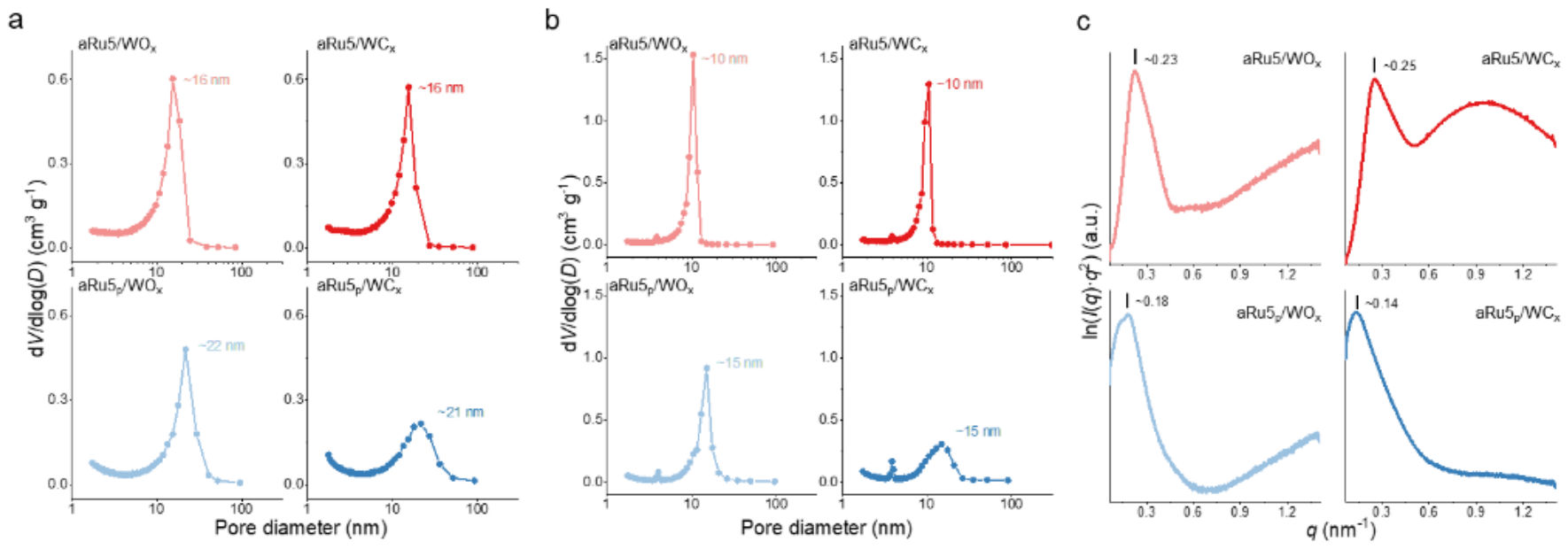

Supplementary Fig. 20 | Porosity alteration by different Ru precursors. a, Pore size distributions with the peak pore sizes labeled, **b,** window size distributions with the peak window sizes labeled, and **c,** Kratky plots of SAXS patterns (The position of the broad scattering maximum (or shoulder) is indicated by line markers). The samples were prepared using HPB Ru (aRu5/WO_x and aRu5/WC_x) and HPL Ru (aRu5_p/WO_x and aRu5_p/WC_x) at different annealing *T*.

Conventional SAXS intensity profiles (*I*(*q*) vs. *q*) have been added as complementary data to facilitate direct comparison of peak width variations.

(Page 32 in the revised Supplementary Information)

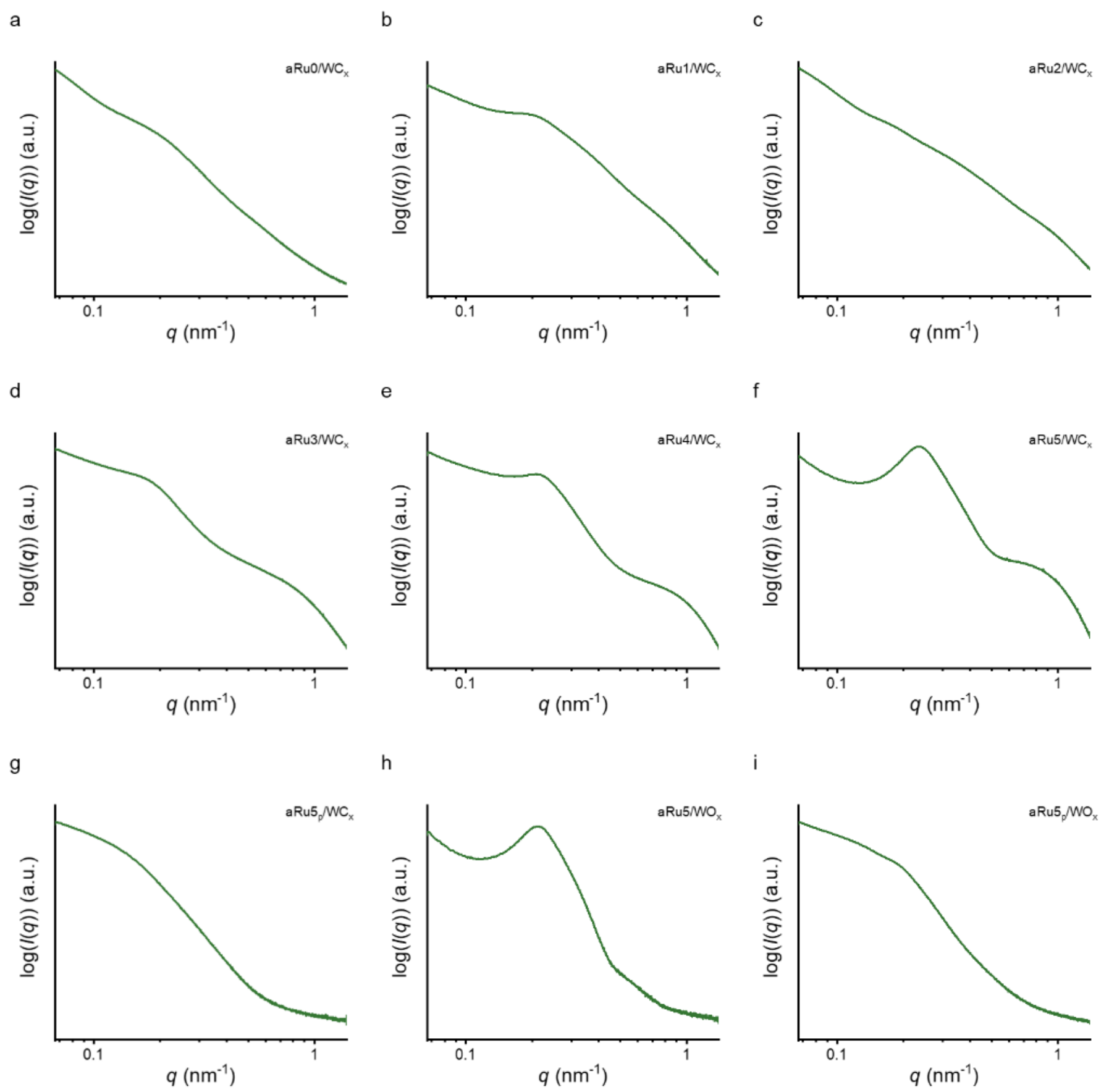

Supplementary Fig. 14 | SAXS profiles. a-i, SAXS intensity profiles (log *I*(*q*) vs. *q*) of samples prepared via self-assembly: (**a**) aRu0/WC_x, (**b**) aRu1/WC_x, (**c**) aRu2/WC_x, (**d**) aRu3/WC_x, (**e**) aRu4/WC_x, (**f**) aRu5/WC_x, (**g**) aRu5_p/WC_x, (**h**) aRu5/WO_x, and (**i**) aRu5p/WO_x**.** The position of the broad scattering maximum (or shoulder) is indicated by line markers.

Reviewer #3 (Remarks to the Author):

This manuscript reports a “Ru atomic-layered nanonetwork (ALN)” platform supported on WCx for alkaline HER and AEMWE cathodes. The authors claim that a thermodynamic stabilization strategy enables size-tunable Ru ensembles with high utilization, and that synergistic dual-site catalysis (Ru sites for *H adsorption and WC_x for *OH adsorption) improves interfacial water structure and kinetics. The topic is timely and relevant, and the electrochemical and device-level performances appear competitive. However, several key claims (especially regarding structural assignment of Ru ALNs and the interpretation of OH enrichment or gap water) rely on indirect evidence and potentially over-interpreted spectroscopy. Additional validation and more rigorous control experiments are needed before the conclusions can be fully supported. Addressing the following suggestions will render the manuscript suitable for publication in this journal.

Reply

We sincerely thank the reviewer for the careful evaluation of our manuscript and for the constructive comments and suggestions. We appreciate the reviewer’s recognition of the significance of the Ru atom-layer neighbor (ALN) platform and its promising electrochemical and AEMWE performance.

We also fully acknowledge the reviewer’s concerns regarding the structural assignment of the Ru ALNs, the interpretation of interfacial OH-related phenomena, the distinction between support and ensemble effects, and the need for additional validation under practical operating conditions. These comments have been extremely valuable in strengthening both the scientific rigor and the overall presentation of the manuscript.

In response, we substantially expanded the theoretical, structural, spectroscopic, electrochemical, and device-level investigations throughout the revised manuscript and Supplementary Information. To rigorously evaluate the structural assignment of the Ru ALNs, we performed additional DFT calculations, molecular dynamics simulations, EXAFS and WT-EXAFS analyses, STEM simulations, and experimentally verified control studies to systematically compare the Ru ALN model with alternative structural possibilities, including carbon-supported Ru ensembles, incompletely dispersed Ru clusters, Ru nanoparticles, Ru-W alloy structures, and compact three-dimensional Ru clusters. Importantly, the revised results demonstrate that the Ru ALN model is the only structure that consistently reproduces all experimentally observed structural fingerprints and their ensemble size-dependent evolution, providing strengthened agreement between theoretical predictions and experimental observations.

To further clarify the origin of the enhanced catalytic performance, we synthesized and characterized multiple control catalysts, including aRu/NC, RuNP/WC_x, and RuNP/C. Together with additional DFT analyses, these studies enabled us to decouple the contributions of neighboring Ru-Ru interactions, Ru-WC_x interactions, and support-mediated interfacial effects, providing a more rigorous mechanistic understanding of the catalytic behavior.

We further examined the generality of the thermodynamic stabilization strategy through the synthesis of aPt3/WC_x and aIr3/WC_x, demonstrating that the approach is not limited to Ru but can be extended to other noble metal atom-layer neighbor systems.

In addition, practical validation of the Ru ALN/WC_x system was significantly strengthened through Faradaic efficiency measurements, GC product analyses, intermittent start-stop durability tests, ICP-MS quantification of dissolved species, contact-angle measurements, bubble visualization studies, and EIS-based overpotential breakdown analyses. These additional investigations confirm both the catalytic robustness and practical applicability of the Ru ALN/WC_x catalyst under realistic AEMWE operating conditions.

Overall, the revised manuscript now provides substantially stronger evidence for the structural assignment of Ru ALNs, a more rigorous mechanistic understanding of their catalytic behavior, and expanded validation of their practical stability and performance. We believe that these extensive revisions comprehensively address the reviewer's concerns and significantly strengthen the conclusions of this work.

1. The manuscript claims that Ru ALNs represent an intermediate morphology between SACs and nanoparticles. However, the Ru–Ru coordination number (CN = 1.5–2.5) derived from EXAFS fitting alone is not sufficient to uniquely support a "layered adjacent" configuration. Such CN values could also arise from a statistical mixture of dimers/trimers and ultrasmall 3D Ru clusters. Can the authors provide direct and statistically meaningful structural evidence (e.g., interatomic distance histogram analysis from a large population of HAADF-STEM images, 4D-STEM, iDPC-STEM, or electron tomography) to demonstrate that Ru species indeed form a quasi-2D adjacent/layered ensemble rather than 3D stacked clusters?

Reply

We appreciate the reviewer's suggestions regarding advanced electron microscopy analyses, including interatomic-distance histogram analysis from a large population of HAADF-STEM images, 4D-STEM, iDPC-STEM, and electron tomography. These techniques can provide valuable structural information; however, their applicability to the present Ru ALN/WC_x system is intrinsically limited by the combination of a heavy WC_x support, the extremely small size of the Ru ensembles, and beam-induced structural evolution.

First, interatomic-distance histogram analysis requires reliable identification of individual Ru atomic positions from a statistically significant number of HAADF-STEM images. However, as discussed in the manuscript and Supplementary Note 8, the substantially higher atomic number of W ($Z = 74$) compared with Ru ($Z = 44$) results in dominant contrast from the WC_x support. Both simulated and experimental HAADF-STEM images demonstrate that individual Ru atoms cannot be unambiguously distinguished from local contrast variations within the WC_x lattice (**Supplementary Figs. 37, 38**). Consequently, reliable extraction of Ru atomic coordinates, which is a prerequisite for meaningful interatomic-distance histogram analysis, is not feasible for the present Ru ALN/WC_x system.

We also carefully considered the use of advanced atomic-resolution imaging approaches. Although a double Cs-corrected TEM was employed in this work, direct visualization of atomically thin Ru layers remains fundamentally challenging because the Ru ALNs are stabilized on a heavy W-containing support. Simulated STEM images and experimental observations consistently indicate that atomically thin Ru ALNs cannot be distinguished from the underlying WC_x lattice with sufficient confidence (**Supplementary Figs. 37, 38**). Similarly, interpretation of 4D-STEM and iDPC-STEM data is complicated by the fact that the scattering contribution from the WC_x support dominates both imaging and diffraction signals. Under these conditions, separating the contribution of atomically thin Ru ALNs from that of the support becomes highly challenging, making unambiguous structural determination difficult.

In addition, acquisition of statistically meaningful 4D-STEM datasets generally requires substantially higher cumulative electron doses than conventional HAADF-STEM imaging. In control experiments using aRu/NC (Ru atomic clusters on N-functionalized carbon) (**Supplementary Fig. 111**), prolonged electron-beam irradiation resulted in observable aggregation and structural evolution of Ru atomic clusters, indicating that the original atomic configuration can be altered during extended observation (**Supplementary Video 8**). Electron tomography faces an

even greater challenge because three-dimensional reconstruction requires acquisition of a large tilt series under prolonged electron-beam exposure. Furthermore, reliable atomic-resolution tomography generally requires electron-transparent specimens with minimal dynamical scattering. In contrast, the present catalyst consists of powder-type mesoporous carbon frameworks containing embedded WC_x nanodomains, where the heavy W atoms introduce strong multiple-scattering effects. Consequently, obtaining unambiguous structural information without perturbing the original Ru ensemble configuration is extremely difficult for the present system.

More importantly, even if such advanced microscopy measurements could be successfully performed, they would provide information only from a limited local region of the catalyst. In contrast, the central question in this work is whether Ru ALNs represent the predominant structural motif throughout the catalyst. Therefore, bulk-sensitive characterization techniques are essential. For this reason, we combined atomic-resolution microscopy with EXAFS and WT-EXAFS analyses, which directly probe the average coordination environment of all Ru species in the sample.

To rigorously verify the Ru ALN structure, we systematically compared theoretically predicted structural parameters with experimentally obtained coordination environments. DFT calculations showed that atomically thin Ru ensembles are thermodynamically more stable than three-dimensional Ru clusters on WC_x below a critical size (**Supplementary Figs. 68, 69**). Consistently, molecular dynamics simulations revealed spontaneous structural relaxation of three-dimensional Ru clusters into atom-layer configurations on WC_x (**Supplementary Fig. 6, Supplementary Videos 1–5**), indicating that the ALN geometry is the energetically preferred structure in the experimentally relevant size regime.

Most importantly, the experimentally observed Ru-Ru bond length elongation relative to bulk metallic Ru (~2.68 Å) was consistently reproduced by the Ru ALN models, whereas all alternative structural models predicted Ru-Ru bond length contraction. The average Ru-Ru distance of aRuN/WC_x (~2.74 Å) was significantly elongated compared with those of nanoparticle reference samples (Ru_p/WC_x_H_2, Ru/WC_x_H_2, Ru/WC_x_R, and Ru/C_R; ~2.67 Å) and bulk Ru metal (~2.68 Å) (**Supplementary Tables 8–11**). Importantly, DFT calculations showed that such Ru-Ru bond-length elongation is not a general consequence of small Ru ensembles or WC_x-supported Ru species. Instead, elongation was reproduced only for atomically thin Ru ALN configurations stabilized through strong Ru-W and Ru-C interactions at the WC_x interface (**Supplementary Figs. 68–70, Supplementary Note 8**).

To further examine alternative structural possibilities raised by the reviewers, we explicitly constructed compact three-dimensional Ru ensembles, including a three-dimensional Ru3 cluster on γ-WC and a tetrahedral Ru4 cluster on γ-WC (**Supplementary Fig. 68b, Supplementary Fig. 81**), which represent the smallest plausible three-dimensional Ru configurations that could account for the experimentally observed low Ru-Ru coordination numbers. In both cases, the calculated Ru-Ru bond lengths were substantially shorter than the experimental values, with three-dimensional Ru3 and Ru4 exhibiting average Ru-Ru distances of 2.45 and 2.58 Å, respectively. Notably, these values are even shorter than that of bulk metallic Ru (~2.68 Å), indicating bond-length contraction rather than the pronounced bond-length elongation observed experimentally for aRuN/WC_x (~2.74 Å). Similarly, Ru ensembles adsorbed on graphene surfaces or confined within graphitic shells, representing carbon-stabilized Ru clusters, also exhibited contracted Ru-Ru bond lengths that were inconsistent with the experimental observations (**Supplementary Figs. 75, 76**). These results

demonstrate that neither compact three-dimensional Ru clusters nor carbon-stabilized Ru ensembles can reproduce the experimentally observed Ru-Ru bond length elongation and are therefore fundamentally inconsistent with the structural fingerprints of aRuN/WC_x.

This conclusion is further supported by experimentally verified control samples. First, the aRu/NC catalyst, which contains three-dimensional Ru atomic clusters stabilized on N-functionalized carbon (**Supplementary Figs. 111, 112**), exhibited a Ru-Ru coordination number of 1.92, comparable to that of aRu3/WC_x (1.72) (**Supplementary Table 19**). However, despite the similar coordination numbers, aRu/NC exhibited a substantially shorter Ru-Ru bond length of 2.61 Å, which is even shorter than that of bulk metallic Ru (~2.68 Å). These results demonstrate that low Ru-Ru coordination numbers alone do not necessarily indicate an ALN structure and can also arise from incompletely dispersed three-dimensional Ru clusters. More importantly, such three-dimensional Ru clusters consistently exhibit contracted Ru-Ru bond lengths, in sharp contrast to the pronounced Ru-Ru bond length elongation observed for aRuN/WC_x.

In addition, the experimentally observed structure–activity relationship is difficult to reconcile with a statistical mixture of independent dimers and trimers. If the measured Ru-Ru coordination numbers merely reflected varying proportions of unrelated Ru species, the catalytic activity would be expected to evolve as a weighted average of the constituent ensembles. Instead, the HER activity exhibits a clear maximum at an intermediate Ru-Ru coordination number, indicating a well-defined ensemble-size effect rather than a simple averaging behavior (**Fig. 5d**). Although this observation does not by itself establish the ALN structure, it is inconsistent with the catalyst being dominated by a random statistical mixture of independent dimers and trimers.

Further evidence is provided by WC_x-supported Ru nanoparticle (RuNP/WC_x) and Ru nanocluster (Ru/WC_x_R) reference samples. RuNP/WC_x exhibited a Ru-Ru coordination number of 2.27, nearly identical to that of aRu5/WC_x (2.29), yet the two samples displayed fundamentally different structural characteristics (**Supplementary Tables 9, 19**). In RuNP/WC_x and Ru/WC_x_R, sub-nanometer Ru nanoparticles or nanoclusters could be directly identified from HAADF-STEM images through distinguishable nanoparticle-like contrast features (**Supplementary Fig. 39, 113**). In contrast, no analogous nanoparticle- or nanocluster-like features were observed for aRu5/WC_x, even in high-resolution HAADF-STEM images (**Fig. 22, Supplementary Fig. 83**). Moreover, the Ru nanoparticle reference samples exhibited Ru-Ru bond lengths of ~2.66 Å, close to but slightly shorter than that of bulk metallic Ru, whereas all aRuN/WC_x samples consistently exhibited elongated Ru-Ru bond lengths of ~2.74 Å (**Supplementary Tables 8–11, 19**). Taken together, these control experiments demonstrate that incompletely dispersed Ru clusters and Ru nanoparticles can exhibit Ru-Ru coordination numbers comparable to those of aRuN/WC_x, but they consistently display contracted Ru-Ru bond lengths and/or identifiable nanoparticle-like features in STEM images. In contrast, the combination of elongated Ru-Ru bond lengths, the absence of nanoparticle-like contrast, and the unique Ru-C/Ru-W coordination environment observed for aRuN/WC_x is uniquely consistent with the Ru ALN configuration.

Furthermore, the characteristic Ru-Ru bond length elongation was accompanied by distinct Ru-W and Ru-C coordination environments, and, importantly, all of these structural descriptors were reproduced only by the Ru ALN model. EXAFS fitting consistently revealed Ru-W coordination numbers greater than unity together with an unusually long Ru-W bond length of ~2.84 Å for aRuN/WC_x (**Supplementary Fig. 80e, Supplementary Tables 8–11**). DFT-derived coordination

analyses further showed that the experimentally observed ensemble size-dependent evolution of Ru-W coordination numbers could only be reproduced when Ru was stabilized as atomically thin ALNs on WC_x, whereas three-dimensional Ru clusters, Ru ensembles on W-terminated γ-WC and δ-WC surfaces, and alloy-type Ru-W structures failed to reproduce the experimentally observed ensemble size dependent evolution of Ru-W coordination numbers (**Supplementary Figs. 78-80, Supplementary Table 13**). Moreover, alloy-type Ru-W nanoparticle reference samples exhibited substantially shorter Ru-W bond lengths (~2.70–2.72 Å), reflecting fundamentally different local coordination environments from those observed experimentally (**Supplementary Fig. 80e, Supplementary Note 8**).

In addition, WT-EXAFS and EXAFS analyses consistently identified Ru-C coordination together with Ru-W coordination (**Fig. 3d, e, Supplementary Tables 8–11**), indicating that the Ru ensembles are directly anchored at the WC_x surface through simultaneous Ru-C and Ru-W interactions. Such a coordination environment is fundamentally different from those of carbon-supported Ru clusters, graphitic shell-confined Ru species, or Ru-W alloy nanoparticles. Therefore, the experimentally observed combination of (i) elongated Ru-Ru bond lengths (~2.74 Å), (ii) Ru-W coordination numbers exceeding unity with a bond length of ~2.84 Å, and (iii) direct Ru-C anchoring interactions can only be consistently explained by the Ru ALN structure.

Therefore, the structural assignment of Ru ALNs does not rely on a single characterization result. Rather, it emerges from the convergence of multiple independent theoretical and experimental observations. Importantly, the Ru ALN model is the only structure that simultaneously reproduces all experimentally observed structural fingerprints, including (i) thermodynamic stabilization of atomically thin Ru ensembles, (ii) the characteristic Ru-Ru bond length elongation (~2.74 Å), (iii) Ru-W coordination numbers exceeding unity together with a distinctive Ru-W bond length of ~2.84 Å, and (iv) direct Ru-C anchoring interactions identified by EXAFS and WT-EXAFS analyses. In contrast, three-dimensional Ru clusters, carbon-supported Ru ensembles, Ru nanoparticles, and Ru-W alloy structures consistently fail to reproduce one or more of these experimentally observed descriptors. Therefore, the agreement between theory and experiment extends across multiple independent structural parameters and provides strong bulk-level evidence that Ru ALNs constitute the predominant structural motif in aRuN/WC_x.

Revision

To further strengthen the structural assignment of Ru atom-layer neighbors (ALNs), we performed additional theoretical and experimental analyses to evaluate alternative structural models proposed by the reviewer, including carbon-supported Ru ensembles, simple Ru ensembles adsorbed on WC surfaces, Ru-W alloy structures, and compact three-dimensional Ru clusters. Additional DFT calculations, STEM simulations, EXAFS analyses, and experimentally verified control samples were incorporated to compare the structural fingerprints of these alternative models with those observed experimentally. Furthermore, the limitations of advanced electron microscopy techniques for the present Ru ALN/WC_x system were discussed. The revised manuscript and Supplementary Information now demonstrate that the Ru ALN model uniquely reproduces the experimentally observed Ru-Ru bond length elongation, distinctive Ru-W coordination environment, direct Ru-C

anchoring interactions, and their ensemble size-dependent evolution, providing comprehensive evidence for the predominance of Ru ALNs in aRuN/WC_x.

(Page 11 in the revised Manuscript)

Taken together, the Ru ALN model uniquely reproduces all experimentally observed structural fingerprints, including the characteristic Ru-Ru bond length elongation, the distinctive Ru-W coordination environment, direct Ru-C anchoring interactions, and their ensemble size-dependent evolution. The agreement between theory and experiment across these independent structural descriptors provides strong evidence that Ru species in aRuN/WC_x are predominantly stabilized as Ru atom-layer neighbors on WC_x supports (Supplementary Note 8).

(Page 9 in the Supplementary Note 8 of the revised Supplementary Information)

To further clarify whether the first-shell Ru-C coordination originated from carbide-derived carbon in WC_x or from carbonaceous environments such as polymer-derived carbon species, surface carbon contaminants, or graphitic carbon shells, additional EXAFS, STEM-EDS, DFT, and control-sample analyses were performed. In all aRuN/WC_x samples, substantial Ru-W coordination was observed together with Ru-C coordination, with Ru-W coordination numbers exceeding unity and Ru-W bond lengths of approximately 2.84 Å (Supplementary Tables 8, 9). If the Ru species were predominantly stabilized by carbonaceous environments rather than WCx domains, direct Ru-W coordination would be expected to be negligible. High-resolution STEM-EDS analyses further showed that Ru signals were selectively correlated with W-containing domains, whereas Ru intensity became negligible in carbon-rich regions, regardless of whether the analyses were performed on selected WC_x particles or carbon-rich areas (Supplementary Figs. 71-74). These results indicate that Ru species are preferentially associated with WC_x domains rather than carbonaceous regions.

Additional DFT calculations were performed for Ru3 and Ru4 ensembles adsorbed on graphene surfaces or confined between graphene layers to model possible carbon-derived coordination environments (Supplementary Fig. 75). Experimentally, the Ru ALN/WC_x samples exhibited elongated Ru-Ru bond lengths (~2.74 Å) compared with bulk metallic Ru (~2.68 Å), and this tendency was reproduced by Ru ensembles stabilized on γ-WC but not by carbon-supported or graphitic shell-confined Ru ensembles (Supplementary Fig. 76). Consistently, the aRu/NC control sample, which contains Ru atomic clusters stabilized on N-functionalized carbon with a Ru-Ru coordination number comparable to that of aRu3/WC_x, exhibited a contracted Ru-Ru bond length of 2.61 Å (Supplementary Figs. 111, 112, Supplementary Table 19). Taken together, these results indicate that carbonaceous environments do not reproduce the characteristic Ru-Ru bond length elongation of Ru

ALN/WC_x supporting that the first shell Ru-C coordination predominantly originates from carbide-derived carbon atoms participating in the Ru ALN/WC_x interfacial structure.

(Page 10-11 in the Supplementary Note 8 of the revised Supplementary Information)

To further examine the origin of the experimentally observed Ru-W bond length (~2.84 Å), additional DFT calculations were performed for Ru1-Ru6 ensembles adsorbed on W-terminated γ-WC and δ-WC surfaces using various adsorption geometries, including bridge- and hollow-site configurations (Supplementary Figs. 77, 78). For all ensemble sizes, the energetically most favorable structures exhibited average Ru-W bond lengths substantially shorter than the experimentally fitted value and instead closer to those expected for alloy-like Ru-W environments (Supplementary Fig. 79). These results suggest that simple surface-adsorbed Ru ensembles on ideal WC surfaces are unlikely to fully account for the experimentally observed long Ru-W bond length.

Because experimentally fitted EXAFS parameters represent averaged local environments in nanocrystalline WC_x containing defects, local disorder, nonstoichiometric carbide domains, and distributions of Ru ensemble configurations, direct quantitative agreement with idealized periodic DFT models is not necessarily expected. Therefore, rather than focusing solely on absolute bond-length agreement, we compared the experimental Ru-W coordination trends with those predicted by different structural models. Notably, only the Ru ALN/WC_x model reproduced the experimentally observed evolution of Ru-W coordination with increasing Ru ensemble size, whereas Ru ensembles adsorbed on γ-WC and δ-WC surfaces did not exhibit meaningful agreement with the experimental trend (Supplementary Fig. 80). These results further support that the experimentally observed ~2.84 Å Ru-W coordination environment is more consistent with the Ru ALN/WC_x configuration than with conventional alloy-like or simple surface-adsorbed Ru structures. The elongated Ru-W bond length may originate from the unique surface-confined coordination environment of Ru ALNs, where Ru species are simultaneously stabilized through Ru-W and Ru-C interactions on nanocrystalline WC_x rather than within a lattice-constrained metallic alloy framework.

To further evaluate whether the small Ru−Ru CNs of aRuN/WC_x could originate from compact three-dimensional Ru clusters rather than two-dimensional Ru ALNs, additional DFT and STEM-simulation analyses were performed. A tetrahedral Ru4 ensemble was constructed on γ-WC and optimized while maintaining the three-dimensional tetrahedral motif (Supplementary Fig. 81). HAADF-STEM image simulations using a 10-layer γ-WC slab indicated that such a small tetrahedral Ru4 cluster is difficult to distinguish unambiguously from small Ru ensembles based solely on projected HAADF-STEM contrast (Supplementary Fig. 82). However, the DFT-optimized tetrahedral Ru4 structure exhibited an average Ru-Ru bond length of 2.58 Å, substantially shorter than the experimentally observed Ru-Ru bond lengths of the Ru ALN/WC_x samples (~2.74 Å). Similarly, the three-dimensional Ru3 ensemble on γ-WC exhibited an average Ru-Ru bond length of 2.45 Å, shorter than both bulk metallic Ru (2.68 Å) and the experimentally observed Ru ALN/WC_x values (Supplementary Figs. 68, 70). These results indicate that the WC support alone does not induce Ru-Ru bond length

elongation; rather, elongation is reproduced only when Ru ensembles are stabilized in the ALN configuration through strong interactions with WC_x.

This structural assignment was further supported by experimental control samples. The aRu/NC control catalyst, containing Ru atomic clusters stabilized on N-functionalized carbon with a Ru-Ru CN comparable to that of aRu3/WC_x, exhibited a contracted Ru-Ru bond length of 2.61 Å (Supplementary Figs. 111, 112, Supplementary Table 19). In addition, experimentally verified Ru nanoparticle and nanocluster samples, including RuNP/WC_x and Ru/WC_x_R, showed identifiable nanoparticle- or nanocluster-like contrast features in HAADF-STEM images, whereas no analogous features were observed for aRu5/WC_x (Supplementary Figs. 23, 38, 83, 113). The Ru nanoparticles also exhibited a Ru-Ru bond length of ~2.66 Å, slightly shorter than that of bulk metallic Ru and substantially shorter than those of Ru ALN/WC_x samples. Therefore, although Ru-Ru CN alone cannot exclude very small three-dimensional Ru clusters, the combination of elongated Ru-Ru bond lengths, distinct Ru-W coordination, and the absence of nanoparticle-like contrast features supports the assignment of aRuN/WC_x as Ru ALN structures rather than compact three-dimensional Ru clusters or nanoparticles.

(Page 12 in the Supplementary Note 8 of the revised Supplementary Information)

It should also be noted that direct visualization of the Ru ALN configuration using more advanced electron microscopy techniques remains intrinsically challenging for the present catalyst system. Because atomically thin Ru ensembles are stabilized on a heavy W-containing support, the scattering contribution from the underlying WC_x lattice dominates both imaging and diffraction signals, making it difficult to unambiguously separate the contribution of Ru ALNs from that of the support. Furthermore, prolonged electron-beam irradiation can induce structural evolution of sub-nanometer Ru species, as demonstrated by the beam-induced aggregation of Ru atomic clusters in the aRu/NC control sample (Supplementary Video 8). Consequently, techniques requiring extensive data acquisition or prolonged beam exposure, such as interatomic-distance statistical analysis, 4D-STEM, iDPC-STEM, and electron tomography, are difficult to apply without potentially perturbing the original atomic configuration. Therefore, rather than relying on a single local microscopy measurement, the Ru ALN structure was evaluated through the agreement between multiple independent theoretical and experimental descriptors, including STEM-EELS analyses, EXAFS-derived coordination environments, characteristic Ru-Ru bond-length elongation, distinctive Ru-W coordination environments, and DFT/MD-predicted structural parameters.

Importantly, the Ru ALN model is the only structural model that simultaneously reproduces all experimentally observed structural fingerprints. Specifically, the Ru ALN configuration uniquely accounts for (i) the thermodynamic preference for atomically thin Ru ensembles predicted by DFT and MD simulations, (ii) the characteristic Ru-Ru bond length elongation relative to bulk Ru, (iii) Ru-W coordination numbers exceeding unity together with the distinctive Ru-W bond length of ~2.84 Å, (iv) direct Ru-C anchoring interactions identified by EXAFS and WT-EXAFS analyses, and (v) the ensemble-size-dependent evolution of structural parameters observed experimentally. In contrast, alternative structural models, including three-dimensional Ru clusters, carbon-supported Ru ensembles, Ru nanoparticles, and Ru-W alloy structures, consistently fail to reproduce one or

more of these experimentally observed descriptors. Therefore, the convergence of these independent theoretical and experimental observations provides strong evidence that Ru species in aRuN/WC_x are predominantly stabilized as Ru atom-layer neighbors (ALNs) on WC_x supports.

(Page 93-94 in the revised Supplementary Information)

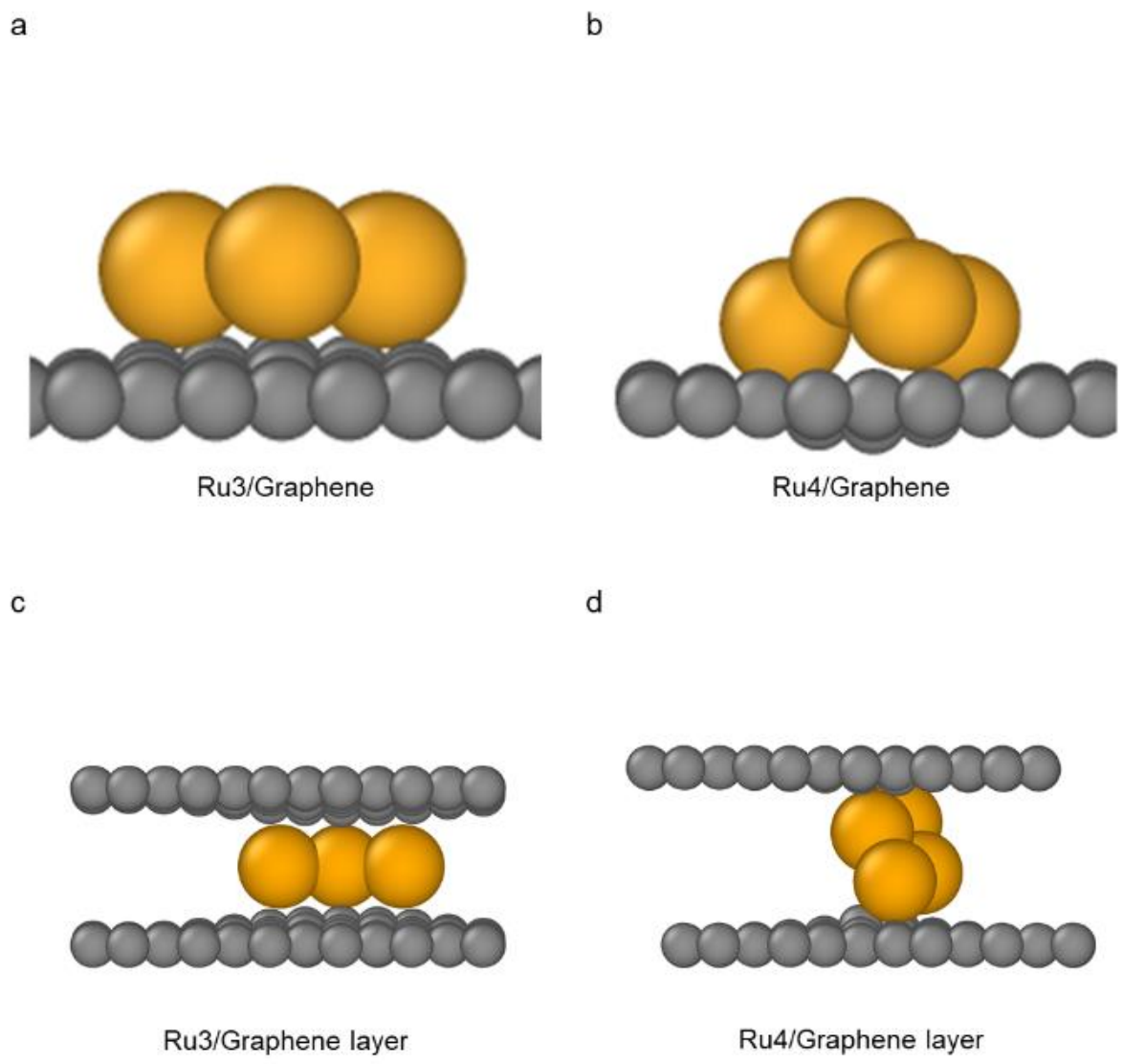

Supplementary Fig. 75 | DFT models representing possible carbon-derived coordination environments of Ru ensembles. a-b, DFT-optimized structures of Ru3 and Ru4 ensembles adsorbed on graphene surfaces. **c-d,** DFT-optimized structures of Ru3 and Ru4 ensembles confined between graphene layers.

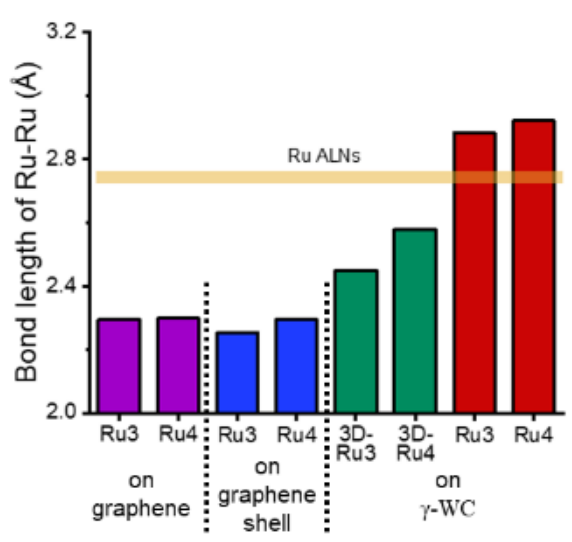

Supplementary Fig. 76 | Comparison of Ru-Ru bond lengths for Ru ensembles stabilized in different coordination environments. Average Ru-Ru bond lengths obtained from DFT-optimized Ru3 and Ru4 ensembles adsorbed on graphene surface (purple), Ru3 and Ru4 ensembles confined between graphene layers (blue), three-dimensional Ru3 and Ru4 ensembles on γ-WC supports (green), and Ru3 and Ru4 ALN stabilized on γ-WC supports (red). The orange shaded region represents the experimentally fitted Ru-Ru bond length range of the Ru ALN/WC_x samples (aRuN/WC_x).

(Page 96-97 in the revised Supplementary Information)

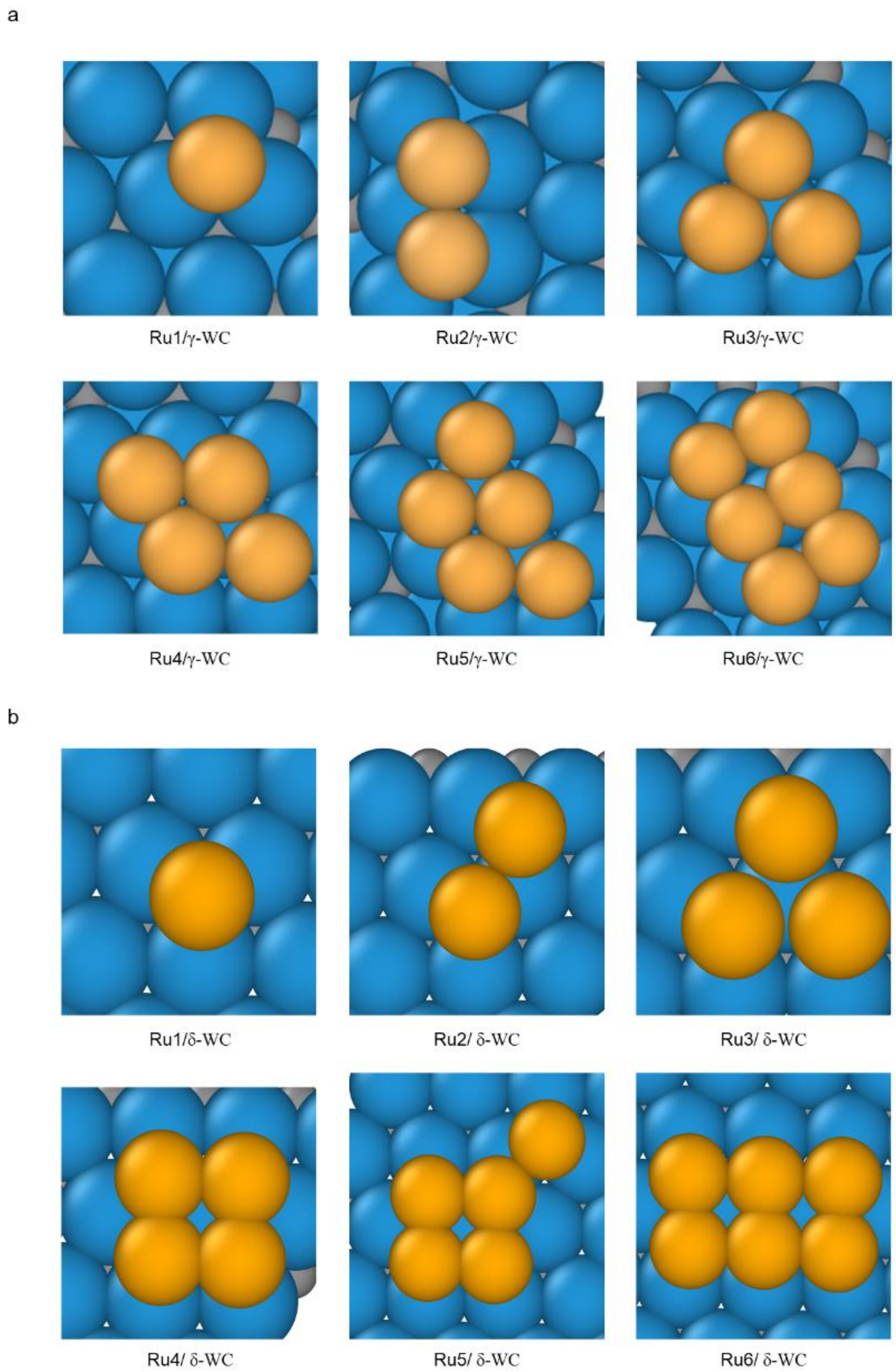

Supplementary Fig. 78 | DFT-optimized structures of Ru ensemble structures on γ-WC or δ-WC surfaces. a-b, DFT-optimized structures of Ru1, Ru2, Ru3, Ru4, Ru5, and Ru6 ensembles adsorbed on (**a**) γ-WC, and (**b**) δ-WC supports. Representative adsorption-site stability comparisons between bridge and hollow configurations are provided for Ru single atoms in Supplementary Fig. R1_1_1.

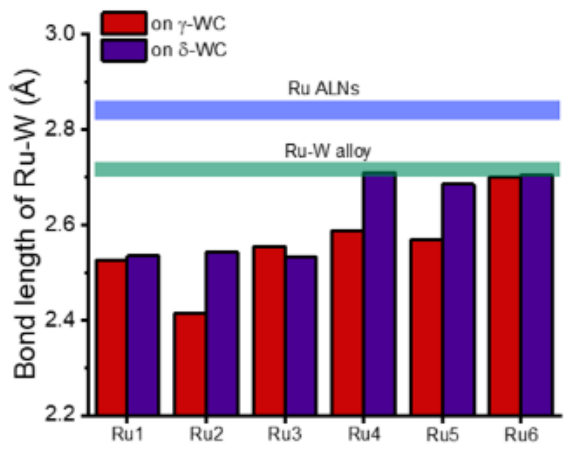

Supplementary Fig. 79 | Comparison of Ru-W coordination environments between DFT models and experimentally fitted structures. Average Ru-W bond lengths obtained from DFT-optimized Ru1, Ru2, Ru3, Ru4, Ru5, and Ru6 ensembles on γ-WC and δ-WC supports. The blue shaded region represents the experimentally fitted Ru-W bond length range for Ru ALN/WC_x samples (aRuN/WC_x), whereas the green shaded region corresponds to Ru-W alloy-like samples (aRu5$_p$/WO_x, aRu5$_p$/WC_x, Ru$_p$/WC_x_Ar, and Ru/WC_x_Ar).

(Page 99-100 in the revised Supplementary Information)

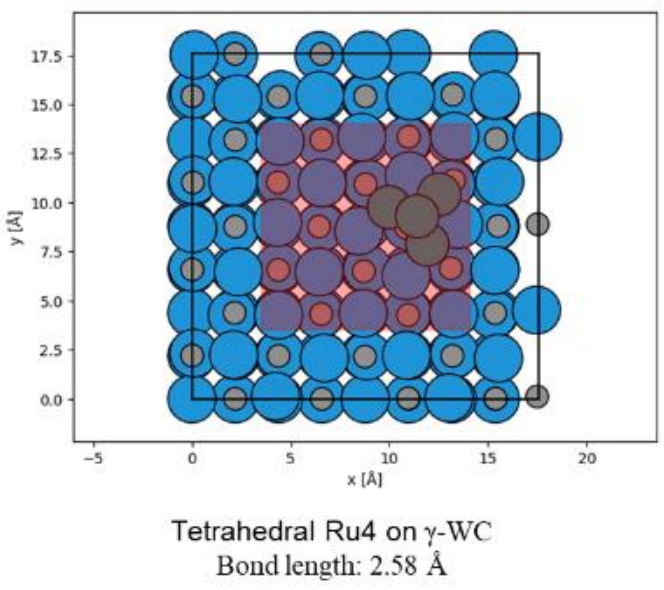

Supplementary Fig. 81 | DFT-optimized tetrahedral Ru4 ensemble on γ-WC. DFT-optimized structure of a three-dimensional tetrahedral Ru4 ensemble on γ-WC. The calculated average Ru-Ru bond length is 2.58 Å.

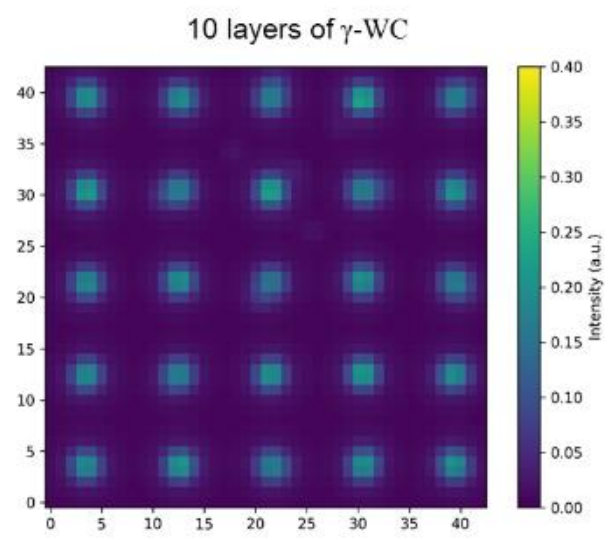

Supplementary Fig. 82 | Simulated HAADF-STEM images of a tetrahedral Ru4 ensembles on γ-WC. Simulated HAADF-STEM image of three-dimensional tetrahedral Ru4 ensembles on 10 layers of γ-WC slab.

(Page 101 in the revised Supplementary Information)

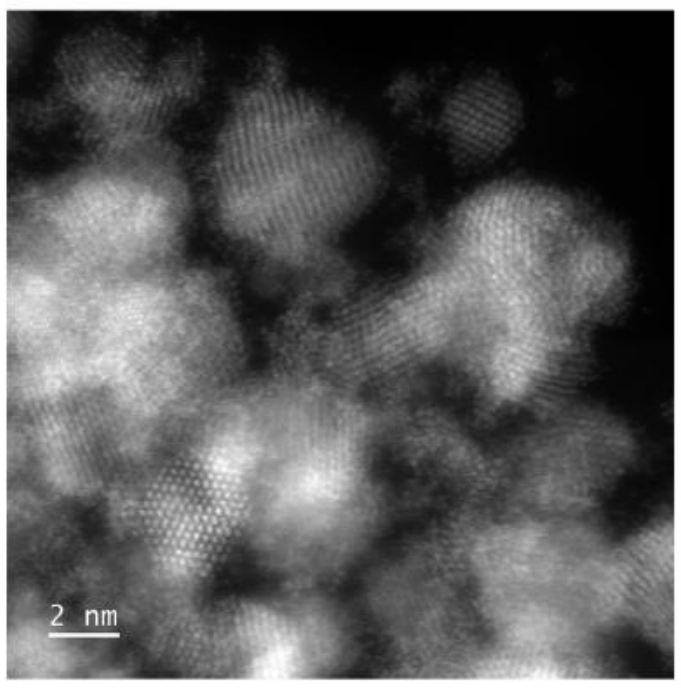

Supplementary Fig. 83 | High-resolution HAADF-STEM analysis of aRu5/WC_x. Representative high-resolution HAADF-STEM image of aRu5/WC_x.

(Page 129-133 in the revised Supplementary Information)

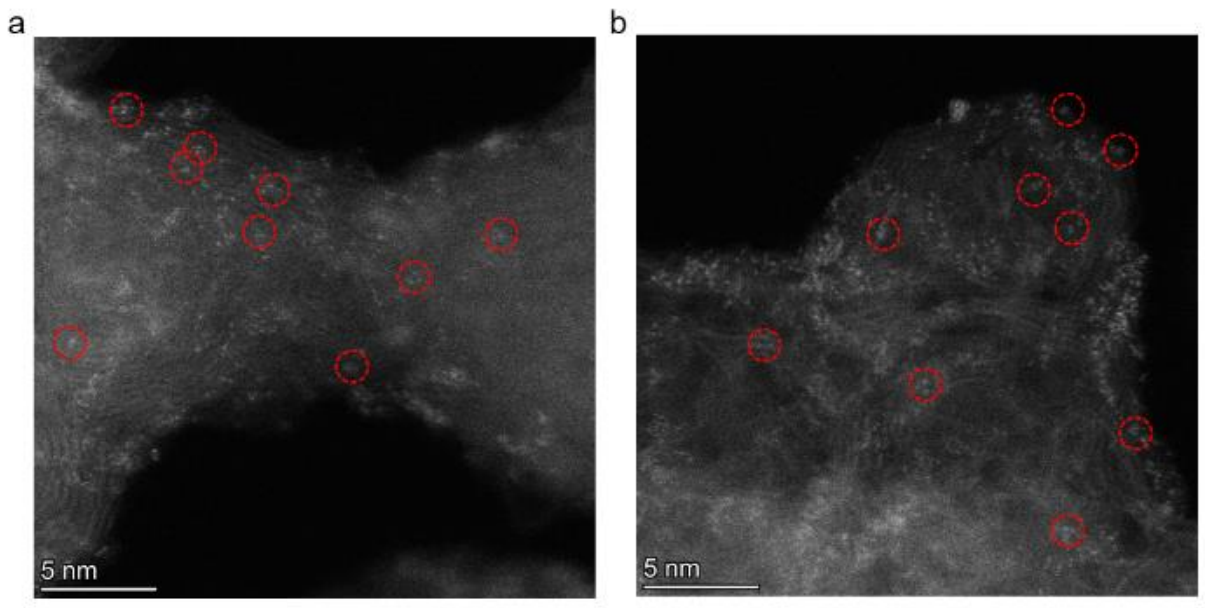

Supplementary Fig. 111 | Atomic-scale structure of aRu/NC. a-b, Representative HAADF-STEM images of aRu/NC (red dotted circles: Ru clusters composed of fewer than 10 Ru atoms)

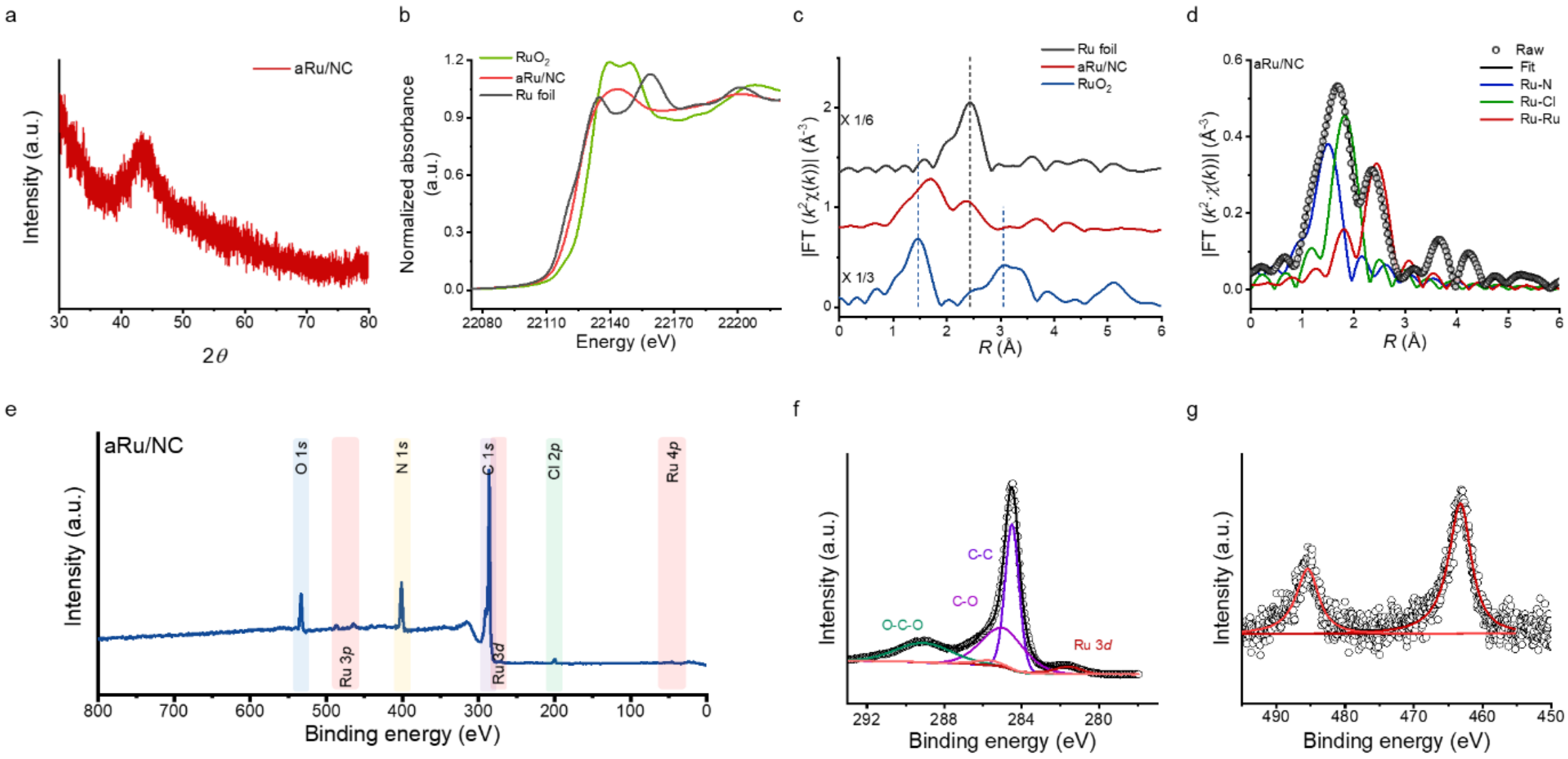

Supplementary Fig. 112 | Geometric and electronic structure of aRu/NC. a, XRD pattern of aRu/NC. **b-d,** XAS analyses of aRu/NC at the Ru K-edge, including (**b**) XANES spectra, (**c**) k^2-weighted FT-EXAFS spectra, and (**d**) corresponding FT-EXAFS fitting results. **e-g,** XPS analyses of aRu/NC, including (**e**) survey spectrum, (**f**) C 1s spectrum, and (**g**) Ru 3p spectrum.

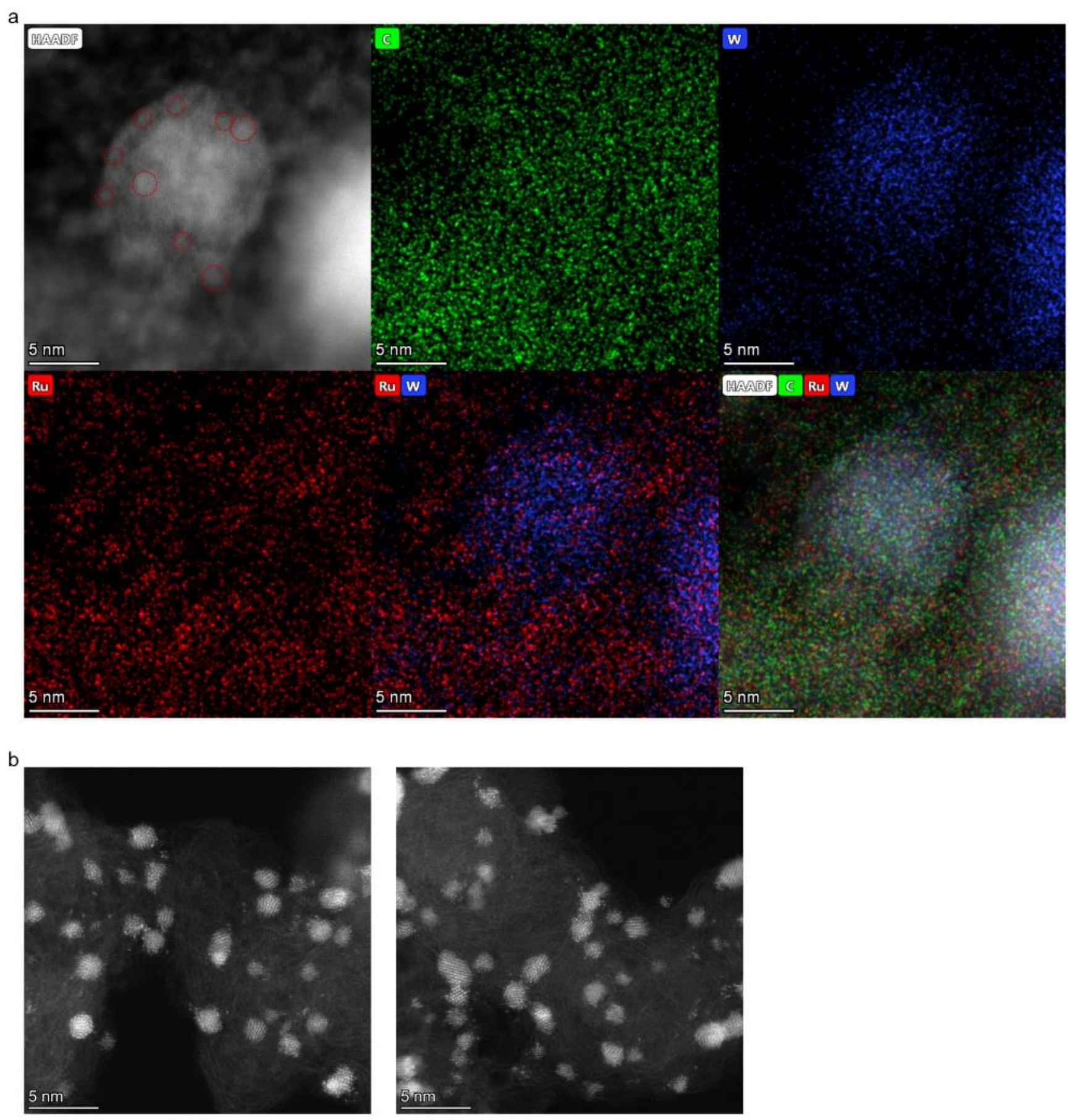

Supplementary Fig. 113 | STEM analysis of RuNP/WC$_x$ and RuNP/C. a, HAADF-STEM image and the corresponding EDS elemental mappings of RuNP/WC$_x$ for C (green), W (blue), Ru (red), along with the overlaid composite map (red dotted circle: Ru NPs). **b,** HAADF-STEM images of RuNP/C.

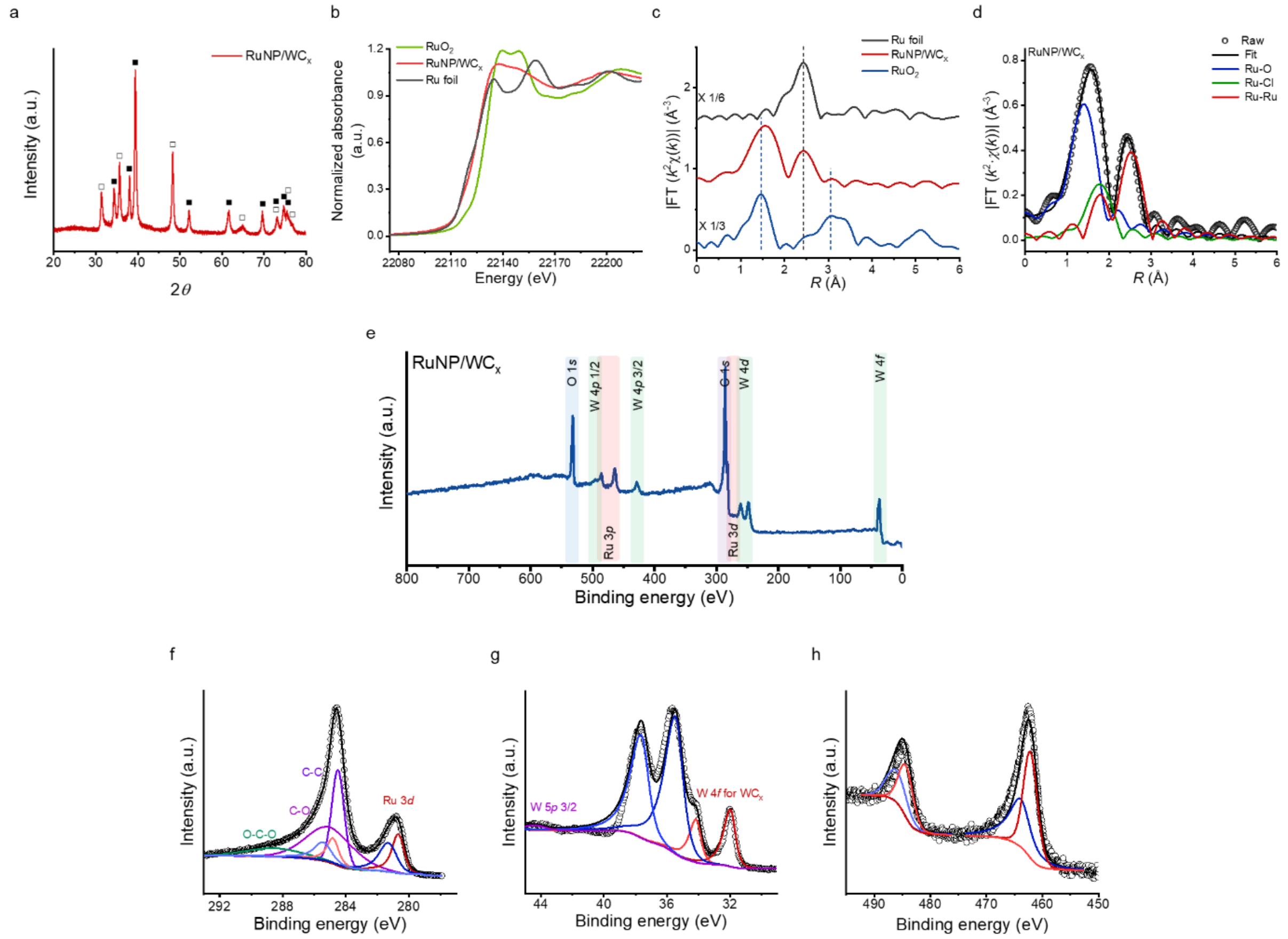

Supplementary Fig. 114 | Geometric and electronic structure of RuNP/WC$_x$. a, XRD pattern of RuNP/WC$_x$. **b-d,** XAS analyses of RuNP/WC$_x$ at the Ru K-edge, including (**b**) XANES spectra, (**c**) k^2-weighted FT-EXAFS spectra, and (**d**) corresponding FT-EXAFS fitting results. **e-h,** XPS analyses of RuNP/WC$_x$, including (**e**) survey spectrum, (**f**) C 1s spectrum, (**g**) W 4f spectrum, and (**h**) Ru 3p spectrum.

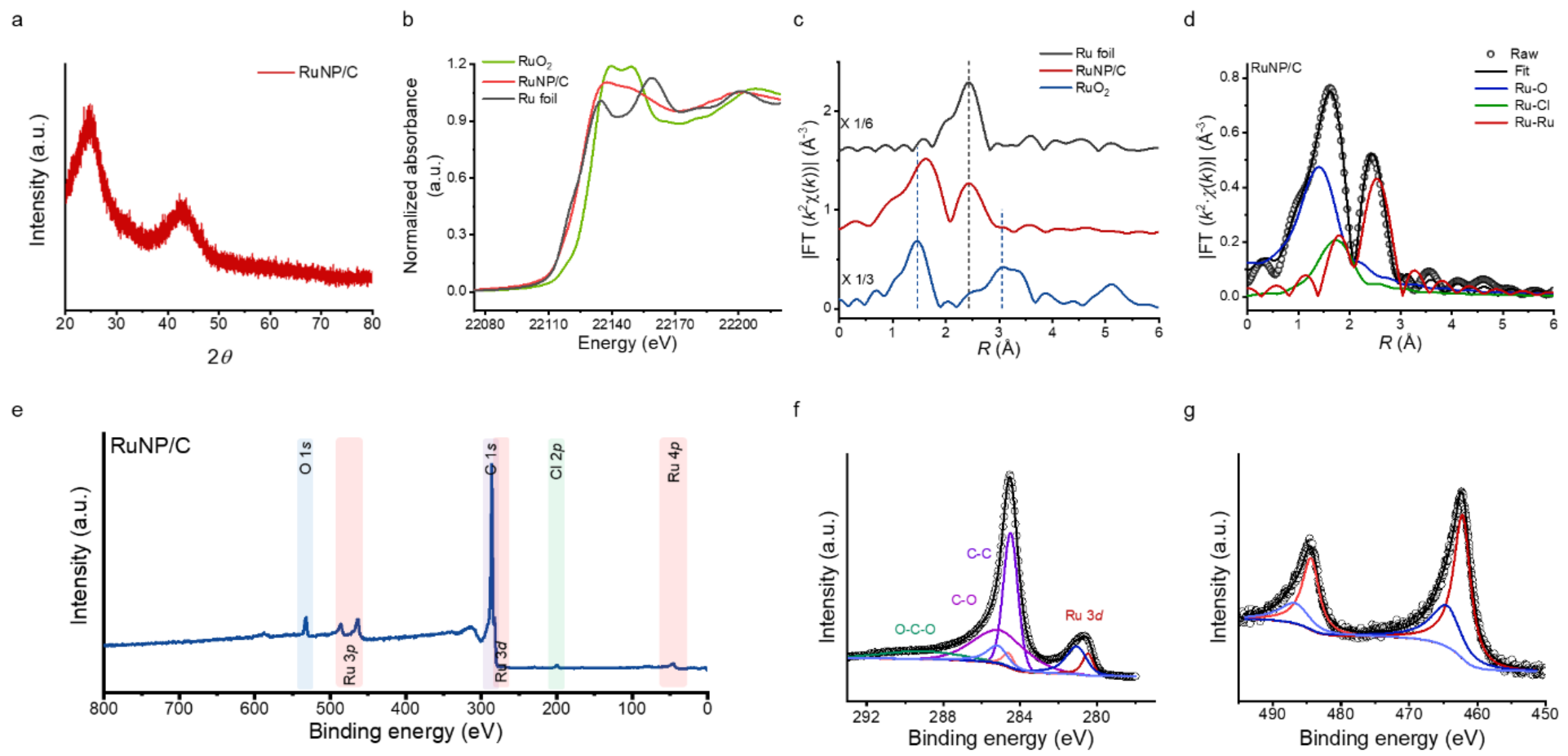

Supplementary Fig. 115 | Geometric and electronic structure of RuNP/C. a, XRD pattern of RuNP/C. **b-d,** XAS analyses of RuNP/C at the Ru K-edge, including (**b**) XANES spectra, (**c**) k^2-weighted FT-EXAFS spectra, and (**d**) corresponding FT-EXAFS fitting results. **e-h,** XPS analyses of RuNP/C, including (**e**) survey spectrum, (**f**) C 1s spectrum, and (**g**) Ru 3p spectrum.

(Page 22-23 in the *Experimental* section of the revised Manuscript)

Synthesis of aRu/NC. For control experiments, aRu/NC were prepared by IWI using soft-nitrided Vulcan XC 72R (*J. Am. Chem. Soc.*, **2016**, 138, 4718-4721). For the synthesis, 27.2 mg of HPL Ru was dissolved in 20 mL of ethanol, sonicated for 10 min, and stirred for 10 min to obtain a homogeneous solution. Next, 0.2 g of soft-nitrided carbon was added to the solution, dispersed by sonication for 10 min, then stirred at 323 K until complete solvent evaporation. The resulting powder was dried at 323 K under vacuum for 12 h. It was then annealed in a tube furnace under H_2 (20 sccm) and Ar (180 sccm) flow at 473 K for 2 h (1 K min^{-1}), then *RT* (5 K min^{-1}). After cooling to *RT*, the sample was passivated under O_2 (2 sccm) and Ar (200 sccm) flow for 12 h, and finely ground in an agate mortar for 10 min.

Synthesis of RuNP/WC_x and RuNP/C. 0.16 g of HPL Ru was dissolved in 20 mL of ethylene glycol in vial. Separately, 0.4 g of NaOH was dissolved in 20 mL of ethylene glycol in three-neck round-bottom flask. After complete dissolution, the two solutions were combined and vigorously stirred for 1 h under continuous Ar purging to remove dissolved oxygen. The resulting solution was then heated to 433 K for 3 hr (4 K min^{-1}), followed by naturally cooling to *RT*. A stable dispersion of Ru NPs in ethylene glycol was obtained. For ligand removal and support immobilization, 5 mL of Ru NP dispersion was mixed with 30 mL of 1.0 M HCL solution in a conical tube after ultrasonication of the dispersion. The mixture was centrifuged, and the supernatant was discarded. This washing process was repeated three times using a total of 15 mL of Ru NP dispersion. Subsequently, the precipitate was additionally washed with 30 mL and then 10 mL of 1.0 M HCl solution via centrifugation to collect the Ru NPs at the bottom of the tube. After acid washing, 117 mg of support materials (WCx or Vulcan XC 72R) and 5 mL of acetone were added to the conical tube. The resulting mixture was ultrasonicated for 1 h in an ice bath while preventing solvent evaporation. The obtained catalyst powder was collected by centrifugation and dried under vacuum at 323 K overnight. The dried samples were subsequently annealed in a tube furnace under H_2 (20 sccm) and Ar (180 sccm) flow at 473 K for 2 h (1 K min^{-1}), followed by an additional 2 h under pure Ar (200 sccm). The samples were then cooled to *RT* (5 K min^{-1}) and passivated under O_2 (2 sccm) and Ar (200 sccm) flow for 12 h.

(Supplementary Video 8)

Supplementary Video 8 Beam-induced aggregation of Ru atomic clusters in aRu/NC during prolonged HAADF-STEM observation. Sequential HAADF-STEM images acquired from the same region under continuous electron-beam irradiation.

2. Given the strong scattering contribution from the WC_x support and the high-temperature reduction process, how can the authors exclude that the Ru–Ru EXAFS contribution originates from residual RuO_x precursor or incompletely dispersed Ru species? It would be important to include reference fitting/LCF analysis against RuO_2 and Ru metal standards, and to explicitly discuss whether Ru–O contributions are present or suppressed (Fig.2f).

Reply

We thank the reviewer for this important comment. We agree that the structural assignment of the Ru species would be difficult to justify unless the possible contribution from residual RuO_x precursor species or incompletely dispersed Ru species are carefully examined.

To investigate whether the first-shell coordination could originate from Ru-O environments, we independently fitted the Ru K-edge EXAFS spectrum of aRu3/WC_x using either Ru-C or Ru-O first shell scattering paths (**Supplementary Fig. 86, Supplementary Table 12**). The Ru-C model provided excellent agreement with the experimental spectrum with an R-factor of 0.003, whereas the Ru-O model resulted in a substantially poorer fit with an R-factor of 0.045. Since R-factors exceeding ~0.020 are generally considered indicative of unreliable fitting results, the Ru-O model cannot satisfactorily describe the first shell coordination environment of aRu3/WC_x. These results demonstrate that the first shell coordination is more consistent with Ru-C coordination than with Ru-O coordination. This conclusion is further supported by WT-EXAFS analysis (**Fig. 3d, Supplementary Figs. 84, 85**). Compared with RuO_2, the maximum intensity in the first shell region of aRuN/WC_x is shifted toward lower *k* values, indicating coordination by a lighter scatterer than oxygen. Combined with the EXAFS fitting results, this observation is consistent with coordination by carbon atoms rather than oxygen atoms.

To further evaluate the possibility of residual RuO_x contributions, we compared the EXAFS features of aRu3/WC_x with those of the RuO_2 reference. The characteristic Ru-Ru paths in RuO_2 are located at approximately 3.17 and 3.57 Å, whereas the Ru-Ru bond length obtained for aRu3/WC_x is only 2.76 Å. Furthermore, the characteristic FT-EXAFS feature of RuO_2 near 3 Å (**Supplementary Fig. 63d**) is absent in aRu3/WC_x (**Supplementary Fig. 64j**), which instead exhibits only the peak near 2.5 Å. Therefore, the observed Ru-Ru contribution cannot be reasonably attributed to residual RuO_x species.

Following the reviewer's suggestion, we additionally performed linear combination fitting (LCF) using Ru metal and RuO_2 standards (**Supplementary Fig. 87**). Although the best fit was obtained using 62.4% Ru metal and 37.6% RuO_2 contributions, noticeable discrepancies remain between the fitted and experimental spectra, particularly in the XANES region. This result indicates that the local coordination environment of Ru in aRu3/WC_x cannot be fully described by a simple combination of metallic Ru and RuO_2 reference structures, suggesting the presence of a distinct Ru coordination environment.

We also evaluated the possibility that the observed Ru–Ru contribution originates from incompletely dispersed Ru species. Since small Ru ensembles can exhibit low Ru-Ru coordination numbers similar to those observed for the Ru ALNs, coordination number alone is insufficient to distinguish incompletely dispersed Ru clusters from Ru ALNs. Therefore, we focused on the Ru-Ru bond length, which provides an additional structural descriptor that can be directly compared between theory and experiment.

To understand the structural origin of the elongated Ru-Ru bond lengths observed for aRuN/WC_x, we systematically compared various theoretical models, including Ru3 and Ru4 ensembles adsorbed on graphene, Ru3 and Ru4 ensembles confined between graphene layers (**Supplementary Fig. 75**), three-dimensional Ru3 and tetrahedral Ru4 clusters on γ-WC (**Supplementary Figs. 68b, 81**) and two-dimensional Ru3 and Ru4 ALNs stabilized on γ-WC (**Supplementary Fig. 6c, d**). For the graphene-supported, graphene-confined, and three-dimensional Ru cluster models, the calculated Ru-Ru bond lengths were consistently shorter than that of bulk metallic Ru (~2.68 Å), indicating contraction of the Ru ensembles (**Supplementary Fig. 76**). In contrast, only the Ru ALN models stabilized through simultaneous Ru-C and Ru-W coordination on WC_x exhibited elongated Ru-Ru bond lengths, reproducing the experimentally observed trend (**Supplementary Table 9**). These results indicate that Ru-Ru bond length elongation is not a general feature of small Ru ensembles but instead emerges only when the Ru species are stabilized as Ru ALNs through strong interactions with the WC_x support.

This tendency was further verified experimentally using control samples representing incompletely dispersed Ru species. Specifically, Ru atomic clusters supported on N-functionalized carbon (aRu/NC) were synthesized, and HAADF-STEM analyses confirmed the formation of three-dimensional Ru atomic clusters composed of fewer than ten Ru atoms, representing a model system of incompletely dispersed Ru species (**Supplementary Figs. 111, 112**). EXAFS fitting revealed that aRu/NC exhibited a Ru-Ru coordination number of 1.92, comparable to that of aRu3/WC_x (1.72), indicating that coordination number alone cannot distinguish incompletely dispersed Ru species from Ru ALNs. However, the Ru-Ru bond length of aRu/NC was only 2.61 Å, which is even shorter than that of bulk metallic Ru (~2.68 Å) (**Supplementary Table 19**). Therefore, although incompletely dispersed Ru species can exhibit similarly low Ru-Ru coordination numbers, they consistently display contracted Ru-Ru bond lengths. In contrast, aRu3/WC_x exhibits a significantly elongated Ru-Ru bond length (~2.76 Å), which is opposite to the trend observed for incompletely dispersed Ru species and is consistent with the theoretical prediction for Ru ALNs stabilized through strong interactions with WC_x. Taken together, these results demonstrate that the experimentally observed Ru-Ru bond length elongation cannot be explained by incompletely dispersed Ru species and instead serves as a characteristic structural fingerprint of the Ru ALN configuration, thereby allowing the possibility of incompletely dispersed Ru species to be effectively excluded.

Taken together, the EXAFS fitting comparison, WT-EXAFS analysis, RuO_2 reference analysis, LCF results, DFT calculations, and experimentally verified control samples consistently demonstrate that the observed Ru-Ru contribution in aRu3/WC_x does not originate from residual RuO_x precursor species or incompletely dispersed Ru clusters. Notably, the experimentally observed Ru-Ru bond length elongation is reproduced only for Ru ALN models stabilized through simultaneous Ru-C and Ru-W coordination on WC_x and is absent for both RuO_x and incompletely

dispersed Ru species. Therefore, the Ru-Ru bond length elongation serves as a characteristic structural fingerprint of the Ru ALN configuration, strongly supporting the assignment of the Ru species in aRu3/WC_x as Ru ALNs stabilized on WC_x supports.

Revision

To address the reviewer's concern regarding the possible contributions of residual RuO_x precursor species and incompletely dispersed Ru species to the observed Ru-Ru EXAFS signal, additional structural analyses were performed. First, Ru-C and Ru-O first shell fitting models were directly compared to evaluate the possibility of residual RuO_x coordination. Additional WT-EXAFS analyses, RuO_2 reference fitting, and XANES linear combination fitting (LCF) using Ru metal and RuO_2 standards were also conducted to further examine the origin of the observed Ru-Ru contribution. To investigate whether the low Ru-Ru coordination numbers observed for aRuN/WC_x could originate from incompletely dispersed Ru clusters, additional DFT calculations were performed for various Ru ensemble configurations, including graphene-supported, graphene-confined, three-dimensional WC-supported, and Ru ALN structures. Furthermore, experimentally verified control samples representing incompletely dispersed Ru species, including Ru atomic clusters on N-functionalized carbon and Ru nanoparticle catalysts, were synthesized and analyzed.

The combined theoretical and experimental results consistently demonstrate that the observed Ru-Ru contribution in aRuN/WC_x cannot be reasonably attributed to residual RuO_x species or incompletely dispersed Ru clusters. Instead, the experimentally observed Ru-Ru bond length elongation is reproduced only for Ru ALN structures stabilized through simultaneous Ru-C and Ru-W coordination on WC_x, providing additional evidence for the structural assignment of Ru ALNs.

(Page 10 in the revised Manuscript)

Furthermore, the experimentally observed Ru-Ru bond length elongation is inconsistent with both residual RuO_x species and incompletely dispersed Ru clusters and is reproduced only for Ru ALN structures stabilized through simultaneous Ru-C and Ru-W coordination on WC_x (Supplementary Note 9).

(Page 12-14 in the Supplementary Note 9 of the revised Supplementary Information)

Supplementary Note 9 | Origin of the Ru-Ru EXAFS contribution in aRuN/WC_x

The origin of the Ru-Ru EXAFS contribution in aRuN/WC_x was investigated through EXAFS fitting analyses, XANES linear combination fitting (LCF), theoretical calculations, and experimentally verified control samples. Particular attention was paid to distinguishing the Ru

ALN structure from residual RuO_x species and incompletely dispersed Ru clusters, both of which could potentially contribute to the Ru-Ru scattering signal.

The possibility of residual RuO_x contributions was first examined by comparing EXAFS fitting results obtained using Ru-C and Ru-O first shell scattering models. For aRu3/WC_x, the Ru-C model provided excellent agreement with the experimental spectrum with an R-factor of 0.003, whereas the Ru-O model resulted in a substantially poorer fit with an R-factor of 0.045 (Supplementary Fig. 86, Supplementary Table 12). Since R-factors exceeding ~0.020 are generally considered indicative of unreliable fitting results, the Ru-O model cannot satisfactorily describe the first shell coordination environment of aRu3/WC_x. These results support the assignment of the first shell coordination to R--C rather than Ru-O species.

Additional evidence was obtained from comparison with the RuO_2 reference. The characteristic Ru-Ru paths of RuO_2 are located at approximately 3.17 and 3.57 Å, whereas the Ru-Ru bond length obtained for aRu3/WC_x is only ~2.76 Å. Furthermore, the characteristic FT-EXAFS feature of RuO_2 near 3 Å (Supplementary Fig. 63d) is absent in aRu3/WC_x (Supplementary Fig. 64j), which instead exhibits only the peak near 2.5 Å. Consistently, WT-EXAFS analysis revealed that the first shell maximum intensity of aRuN/WC_x is shifted toward lower *k* values compared with RuO_2, indicating coordination by lighter scatterers than oxygen (Fig. 3d, Supplementary Figs. 84, 85). Together, these results indicate that the observed Ru-Ru contribution cannot be reasonably attributed to residual RuO_x species.

XANES linear combination fitting (LCF) was performed using Ru metal and RuO_2 standards (Supplementary Fig. 87). Although the best fit was obtained using 62.4% Ru metal and 37.6% RuO_2 contributions, noticeable discrepancies remain between the fitted and experimental spectra, particularly in the XANES region. Therefore, the local coordination environment of Ru in aRu3/WC_x cannot be fully described by a simple combination of metallic Ru and RuO_2 reference structures, suggesting the presence of a distinct Ru coordination environment.

The possibility that the observed Ru-Ru contribution originates from incompletely dispersed Ru species was also examined. Since small Ru ensembles can exhibit low Ru-Ru coordination numbers similar to those observed for Ru ALNs, coordination number alone is insufficient to distinguish incompletely dispersed Ru clusters from Ru ALNs. Therefore, additional structural descriptors were examined through both theoretical and experimental approaches.

To understand the structural origin of the elongated Ru-Ru bond lengths observed for aRuN/WC_x, various theoretical models were systematically compared, including Ru3 and Ru4 ensembles adsorbed on graphene, Ru3 and Ru4 ensembles confined between graphene layers, three-dimensional Ru3 clusters and tetrahedral Ru4 clusters on γ-WC, and two-dimensional Ru3 and Ru4 ALNs stabilized on γ-WC (Supplementary Figs. 6c, d, 68b, 71, 81). For the graphene-supported, graphene-confined, and three-dimensional Ru cluster models, the calculated Ru-Ru bond lengths were consistently shorter than that of bulk metallic Ru (~2.68 Å), indicating contraction of the Ru ensembles (Supplementary Fig. 72). In contrast, only the Ru ALN models stabilized through simultaneous Ru-C and Ru-W coordination on WC_x exhibited elongated Ru-Ru bond lengths, reproducing the experimentally observed trend. These results indicate that Ru-Ru bond-length elongation is not a general feature of small Ru ensembles but instead emerges specifically when the Ru species are stabilized as Ru ALNs through strong interactions with the WC_x support.

This tendency was further verified experimentally using control samples representing incompletely dispersed Ru species. Ru atomic clusters supported on N-functionalized carbon (aRu/NC) were synthesized, and HAADF-STEM analyses confirmed the formation of three-dimensional atomic clusters composed of fewer than ten Ru atoms (Supplementary Figs. 111, 112). EXAFS fitting revealed that aRu/NC exhibited a Ru-Ru coordination number of 1.92, comparable to that of aRu3/WC_x (1.72), indicating that coordination number alone cannot distinguish incompletely dispersed Ru species from Ru ALNs. However, the Ru-Ru bond length of aRu/NC was only 2.61 Å, which is even shorter than that of bulk metallic Ru (~2.68 Å) (Supplementary Table 19). Similarly, Ru nanoparticles supported on WC_x and carbon supports exhibited Ru-Ru bond lengths of ~2.66 Å despite possessing Ru-Ru coordination numbers comparable to those of the Ru ALN samples. Therefore, although incompletely dispersed Ru species can exhibit similarly low Ru-Ru coordination numbers, they consistently display contracted Ru-Ru bond lengths. In contrast, aRu3/WC_x exhibits a significantly elongated Ru-Ru bond length (~2.76 Å), which is opposite to the trend observed for incompletely dispersed Ru species and is consistent with the theoretical prediction for Ru ALNs stabilized through strong interactions with WC_x.

Collectively, the EXAFS fitting comparison, WT-EXAFS analysis, RuO_2 reference analysis, LCF results, DFT calculations, and experimentally verified control samples consistently indicate that the Ru-Ru contribution observed in aRuN/WC_x does not originate from residual RuO_x species or incompletely dispersed Ru clusters. Instead, the experimentally observed Ru-Ru bond-length elongation is reproduced only for Ru ALN structures stabilized through simultaneous Ru-C and Ru-W coordination on WC_x. The Ru-Ru bond length elongation therefore represents a characteristic structural feature of the Ru ALN configuration and supports the assignment of the Ru species in aRuN/WC_x as Ru ALNs stabilized on WC_x supports.

(Page 104-105 in the revised Supplementary Information)

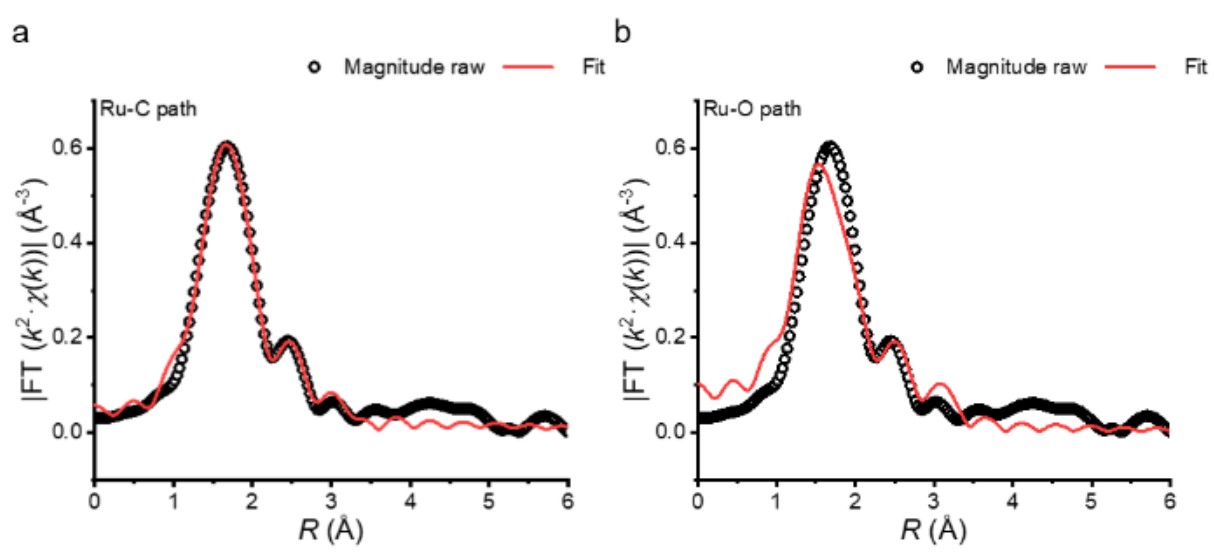

Supplementary Fig. 86 | Comparison of EXAFS Fitting results using Ru-C and Ru-O first shell scattering paths for aRu3/WC_x. a,b, Experimental and fitted Ru K-edge FT-EXAFS spectra of aRu3/WC_x obtained using (**a**) a Ru-C first shell model and (**b**) Ru-O first shell model.

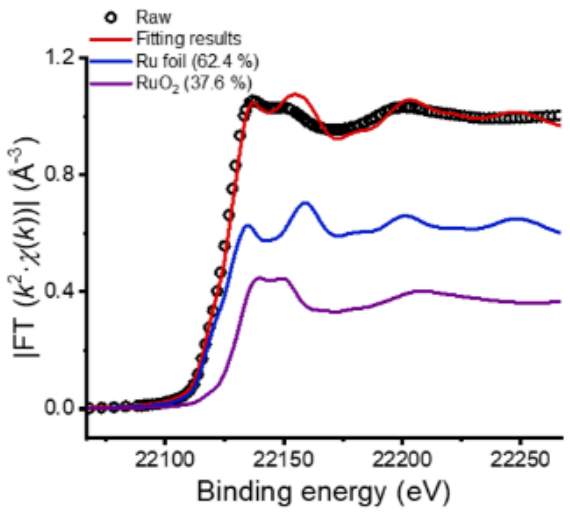

Supplementary Fig. 87 | Ru K-edge XANES linear combination fitting (LCF) of aRu3/WC_x. Experimental spectrum (black circles) and corresponding LCF result (red line) using Ru metal foil and RuO_2 standards.

(Page 165 in the revised Supplementary Information)

Supplementary Table 12 | Comparison of EXAFS fitting results using Ru-C and Ru-O first shell models for aRu3/WC_x. Structural parameters extracted from the Ru K-edge EXAFS fitting results. The experimentally observed first-shell coordination in aRu3/WC_x was fitted using either a Ru-C or Ru-O scattering path to evaluate the origin of the first shell contribution. CN: coordination number, R: interatomic distance, and σ^2: Debye-Waller factor. The goodness of fit is represented by the R-factor. (weighting: k^2)

Sample	Shell	CN	R (Å)	σ^2 (10^{-3} $Å^2$)	R-factor
Ru	Ru-Ru	6*	2.65 ± 0.01	2.56	0.007
	Ru-Ru	6*	2.70 ± 0.01		
RuO_2	Ru-O	2*	1.94 ± 0.01	0.18	0.019
	Ru-O	4*	1.99 ± 0.01		
	Ru-Ru	2*	3.17 ± 0.01	3.30	
	Ru-O	4*	3.31 ± 0.05	0.18	
	Ru-Ru	8*	3.57 ± 0.01	3.30	
aRu3/WC_x	Ru-C	4.01 ± 0.17	2.11 ± 0.00	6.18	0.003
	Ru-Ru	1.72 ± 0.14	2.76 ± 0.01		
	Ru-W	2.28 ± 0.28	2.86 ± 0.01	8.78	
aRu3/WC_x (Ru-O fitting)	Ru-O	2.41 ± 0.93	2.02 ± 0.02	9.28	0.045
	Ru-Ru	1.96 ± 1.03	2.75 ± 0.03		
	Ru-W	2.70 ± 1.38	2.85 ± 0.03	10.11	

Structural parameters extracted from the EXAFS fitting of reference metal foil. CN is the coordination number; R is the interatomic distance; σ^2 is the Debye-Waller factor (a measure of the static and thermal disorder in absorber-scatterer distance); ΔE_0 is edge energy shift (the zero kinetic energy difference between experiment and theoretical model); R-factor value is related with the goodness of the fitting.
* Value was assigned in curving fitting based on standard structure

R_{bkg} was set to 1.0 in background removal to remove the noise oscillation below 1 Å in R-space
S_0^2 value (0.744) was calculated from the fitting of metal foil with standard metal structure

(Page 129-130 in the revised Supplementary Information)

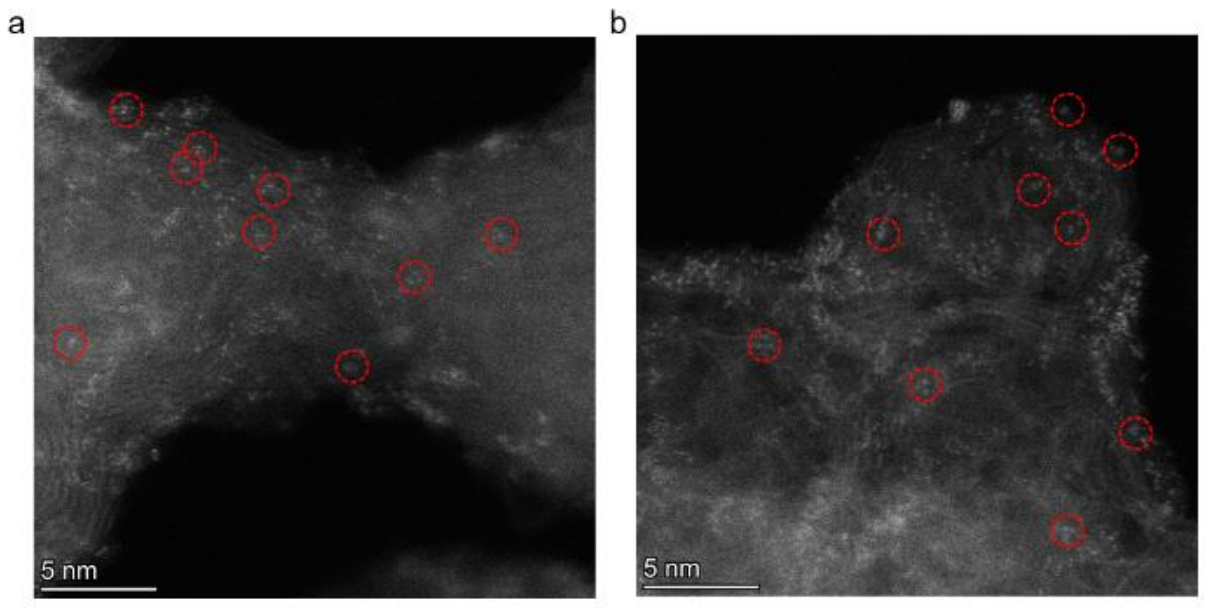

Supplementary Fig. 111 | Atomic-scale structure of aRu/NC. a-b, Representative HAADF-STEM images of aRu/NC (red dotted circles: Ru clusters composed of fewer than 10 Ru atoms)

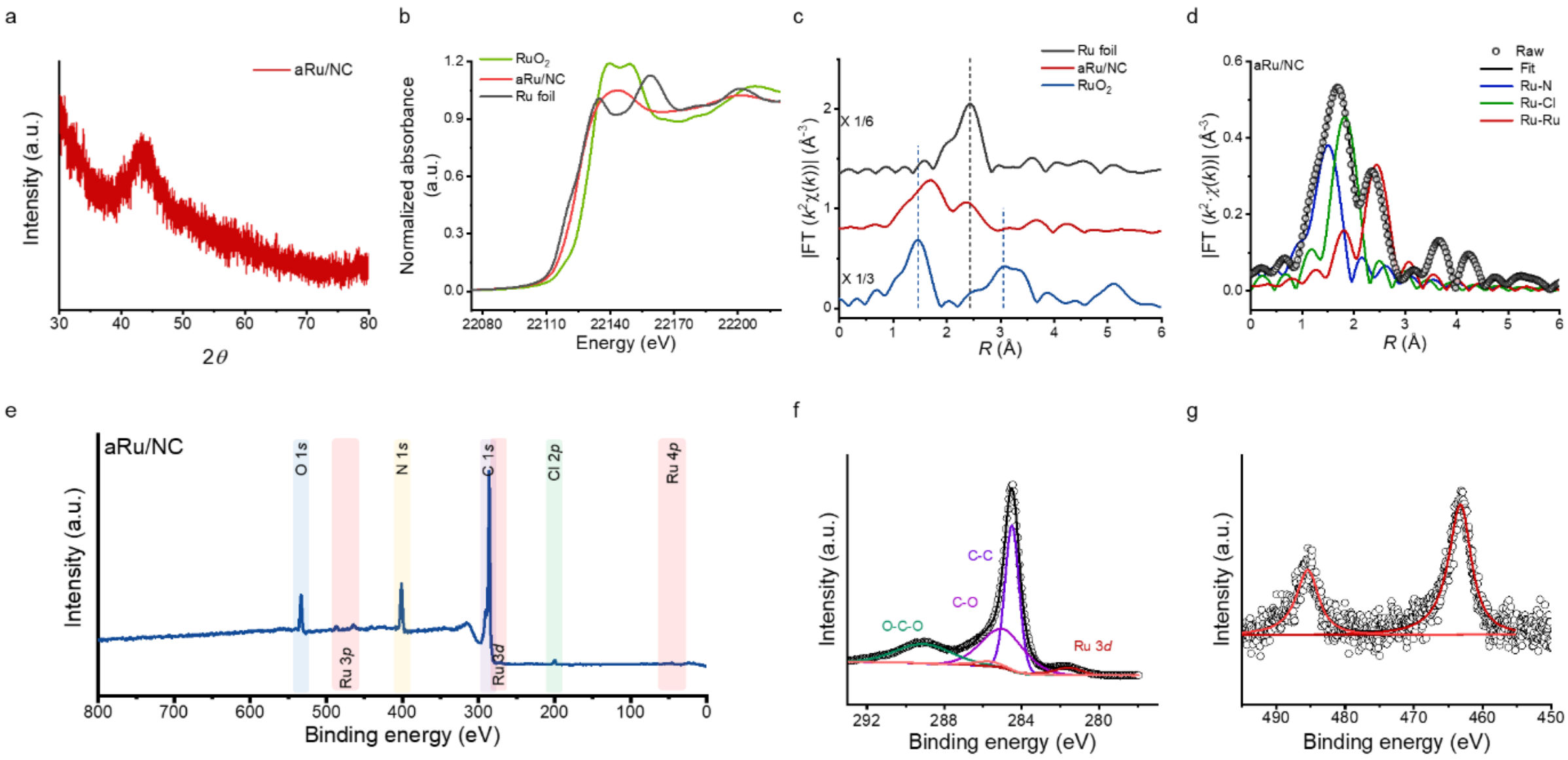

Supplementary Fig. 112 | Geometric and electronic structure of aRu/NC. a, XRD pattern of aRu/NC. **b-d,** XAS analyses of aRu/NC at the Ru K-edge, including (**b**) XANES spectra, (**c**) k^2-weighted FT-EXAFS spectra, and (**d**) corresponding FT-EXAFS fitting results. **e-g,** XPS analyses of aRu/NC, including (**e**) survey spectrum, (**f**) C 1s spectrum, and (**g**) Ru 3p spectrum.

(Page 172 in the revised Supplementary Information)

Supplementary Table 19 | Structural parameters extracted from EXAFS fitting analyses. Structural parameters extracted from the Ru K-edge, Pt L_2-edge, Ir L_2-edge EXAFS fitting results. CN: coordination number, R: interatomic distance, and σ^2: Debye-Waller factor. The goodness of fit is represented by the R-factor. (weighting: k^2)

Sample	Shell	CN	R (Å)	σ^2 (10^{-3} Å^2)	R-factor
Ru	Ru-Ru	6*	2.65 ± 0.01	2.56	0.007
	Ru-Ru	6*	2.70 ± 0.01		
RuO_2	Ru-O	2*	1.94 ± 0.01	0.73	0.014
	Ru-O	4*	1.98 ± 0.01		
Ru/WC_x_R	Ru-O	2.06 ± 0.33	1.97 ± 0.01	5.69	0.012
	Ru-Ru	4.51 ± 0.41	2.66 ± 0.01	6.93	
Ru/C_R	Ru-O	2.79 ± 0.37	1.97 ± 0.01	6.95	0.014
	Ru-Ru	3.70 ± 0.41	2.67 ± 0.01	6.45	
RuNP/WC_x	Ru-O	2.93 ± 0.19	1.94 ± 0.01	5.87	0.003
	Ru-Cl	0.86 ± 0.14	2.30 ± 0.01		
	Ru-Ru	2.27 ± 0.23	2.66 ± 0.01	5.74	
RuNP/C	Ru-O	4.63 ± 0.19	1.97 ± 0.00	17.23	0.001
	Ru-Cl	1.45 ± 0.14	2.27 ± 0.01		
	Ru-Ru	2.35 ± 0.11	2.66 ± 0.00	5.00	
aRu/NC	Ru-N	1.53 ± 0.06	2.01 ± 0.00	1.75	0.001
	Ru-Cl	1.06 ± 0.03	2.33 ± 0.00		
	Ru-Ru	1.92 ± 0.08	2.61 ± 0.00	8.14	
aPt3/WC_x**	Pt-C	1.59 ± 0.30	2.08 ± 0.02	2.16	0.009
	Pt-Pt	4.10 ± 0.34	2.75 ± 0.01	2.07	
	Pt-W	1.93 ± 0.37	2.88 ± 0.01		
aIr3/WC_x**	Ir-C	2.46 ± 0.08	2.11 ± 0.00	2.69	0.001
	Ir-Ir	1.87 ± 0.09	2.73 ± 0.00	1.49	
	Ir-W	1.06 ± 0.09	2.88 ± 0.01		

Structural parameters extracted from the EXAFS fitting of reference metal foil. CN is the coordination number; R is the interatomic distance; σ^2 is the Debye-Waller factor (a measure of the static and thermal disorder in absorber-scatterer distance); ΔE_0 is edge energy shift (the zero kinetic energy difference between experiment and theoretical model); R-factor value is related with the goodness of the fitting.
* Value was assigned in curving fitting based on standard structure
** For Pt and Ir, EXAFS fitting was conducted at the L_2-edge to avoid spectral overlap between the L_3-edge and the W L_2-edge. S_0^2 value was obtained from reference foil measurements at the corresponding L_2-edge
R_{bkg} was set to 1.0 in background removal to remove the noise oscillation below 1 Å in R-space
S_0^2 value (0.744) was calculated from the fitting of metal foil with standard metal structure

(Page 93-94 in the revised Supplementary Information)

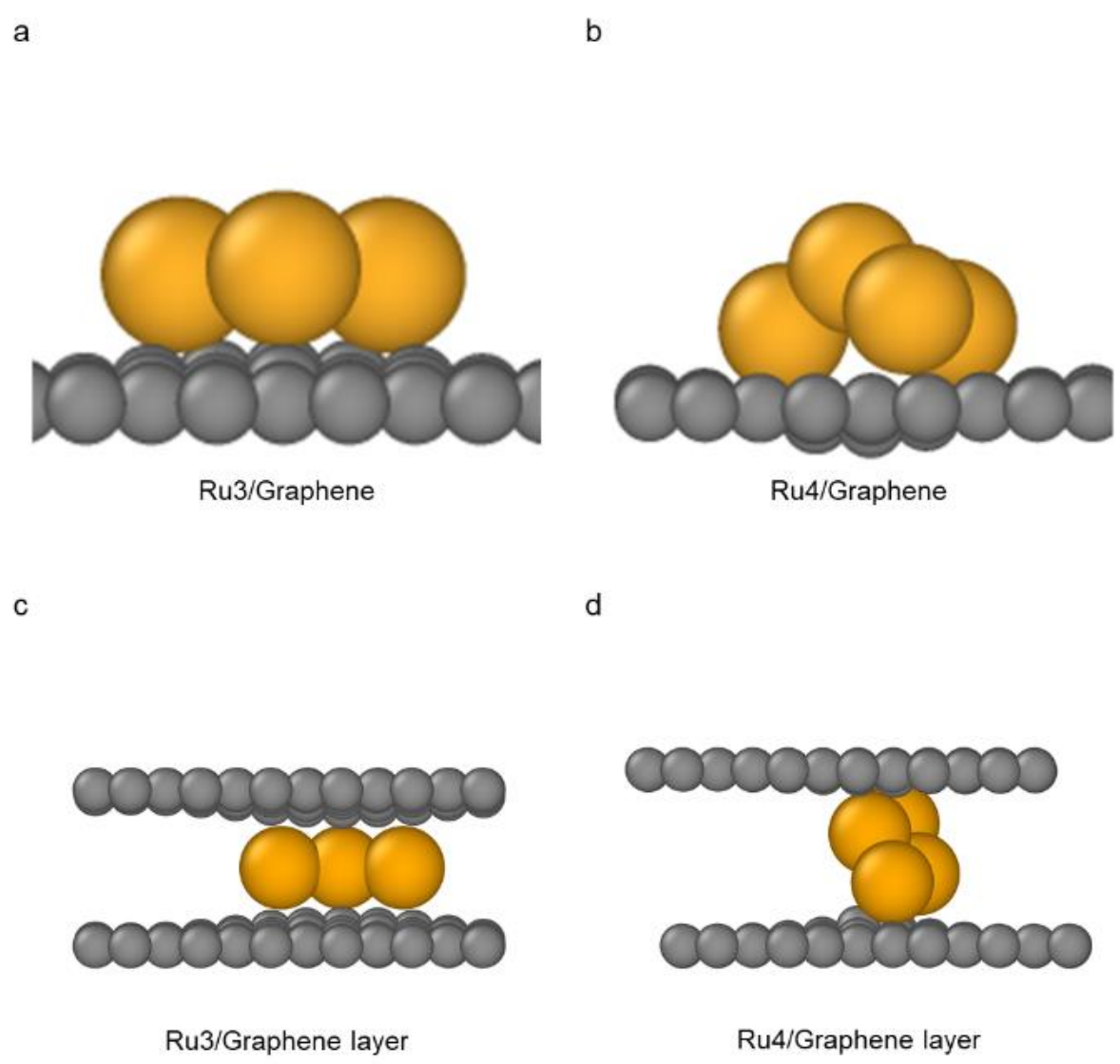

Supplementary Fig. 75 | DFT models representing possible carbon-derived coordination environments of Ru ensembles. a-b, DFT-optimized structures of Ru3 and Ru4 ensembles adsorbed on graphene surfaces. **c-d,** DFT-optimized structures of Ru3 and Ru4 ensembles confined between graphene layers.

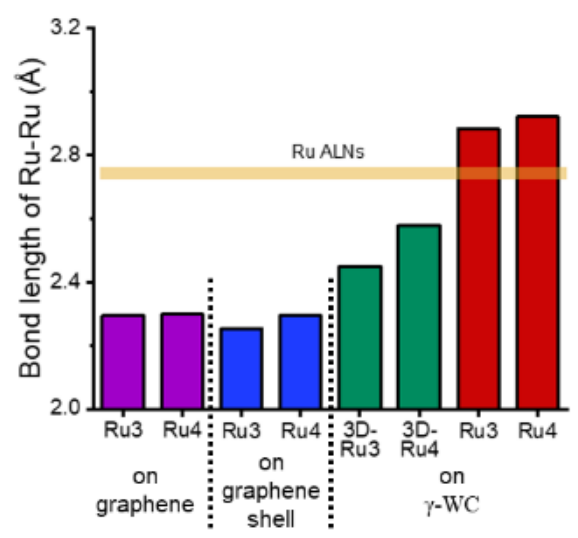

Supplementary Fig. 76 | Comparison of Ru-Ru bond lengths for Ru ensembles stabilized in different coordination environments. Average Ru-Ru bond lengths obtained from DFT-optimized Ru3 and Ru4 ensembles adsorbed on graphene surface (purple), Ru3 and Ru4 ensembles confined between graphene layers (blue), three-dimensional Ru3 and Ru4 ensembles on γ-WC supports (green), and Ru3 and Ru4 ALN stabilized on γ-WC supports (red). The orange shaded region represents the experimentally fitted Ru-Ru bond length range of the Ru ALN/WC_x samples (aRuN/WC_x).

(Page 22 in the *Experimental* section of the revised Manuscript)

Synthesis of aRu/NC. For control experiments, aRu/NC were prepared by IWI using soft-nitrided Vulcan XC 72R (*J. Am. Chem. Soc.*, **2016**, 138, 4718-4721). For the synthesis, 27.2 mg of HPL Ru was dissolved in 20 mL of ethanol, sonicated for 10 min, and stirred for 10 min to obtain a homogeneous solution. Next, 0.2 g of soft-nitrided carbon was added to the solution, dispersed by sonication for 10 min, then stirred at 323 K until complete solvent evaporation.

The resulting powder was dried at 323 K under vacuum for 12 h. It was then annealed in a tube furnace under H_2 (20 sccm) and Ar (180 sccm) flow at 473 K for 2 h (1 K min^{-1}), then *RT* (5 K min^{-1}). After cooling to *RT*, the sample was passivated under O_2 (2 sccm) and Ar (200 sccm) flow for 12 h, and finely ground in an agate mortar for 10 min.

3. Does this high activity primarily stem from the electronic neighbor effect between Ru-Ru atoms, or from the strong modulation of the Ru electronic structure by the WC_x support? We recommend that the authors supplement their work with a set of control experiments: synthesize Ru structures with similar coordination numbers on inert supports (e.g., carbon) or synthesize Ru nanoparticles on the same support. Employ DFT calculations to explicitly determine the relative contributions of the support effect and the spatial proximity effect to the hydrogen adsorption free energy change (ΔGH*).

Reply

We thank the reviewer for this insightful comment regarding the respective roles of neighboring Ru-Ru interactions and support-induced electronic modulation in determining the HER activity of Ru ALNs. We fully agree that distinguishing the contributions from the Ru ensemble effect and the WC_x support effect is important for understanding the origin of the enhanced catalytic activity.

To address this point, we additionally performed comparative DFT calculations for Ru1, Ru2, Ru3, and Ru4 ensembles supported on graphene and γ-WC models, together with Bader charge and hydrogen adsorption free energy (ΔG_H) analyses (**Supplementary Figs. 109, 110**). Bader charge analyses revealed that the electronic structures of Ru ensembles systematically varied with the ensemble size on both supports, indicating that neighboring Ru-Ru interactions themselves significantly influence the electronic structure of Ru active sites (**Supplementary Fig. 110a**). Furthermore, the Ru ensembles on γ-WC consistently exhibited lower Bader charge values than the corresponding Ru ensembles on graphene, demonstrating that additional electronic modulation is induced through strong Ru-WC_x interactions. Similarly, the calculated ΔG_H values also varied depending on both the Ru ensemble size and the support (**Supplementary Fig. 110b**). In particular, Ru ensembles on γ-WC exhibited ΔG_H values closer to the ideal hydrogen adsorption free energy ($\Delta G_H \approx 0$) than the corresponding Ru ensembles on graphene, suggesting that the WC_x support plays a critical role in optimizing the hydrogen adsorption energetics for alkaline HER.

Importantly, a substantial stabilization of ΔG_H was observed when the Ru single atom evolved into a Ru2 ensemble on γ-WC, despite the relatively similar Bader charge values between the two structures. This behavior can be understood from the change in hydrogen adsorption configuration from top-site adsorption on Ru1 to bridge-site adsorption on Ru2 (**Supplementary Fig. 88**), indicating that neighboring Ru sites provide additional adsorption geometries that cannot be achieved on isolated single-atom sites.

To experimentally validate these theoretical interpretations, we additionally synthesized control catalysts possessing comparable Ru coordination environments on different supports. First, atomically dispersed Ru clusters on N-functionalized carbon (aRu/NC) were synthesized as an inert-support analogue with Ru-Ru CNs similar to those of aRu3/WC_x (see *Catalyst synthesis* in *Methods*). HAADF-STEM analyses revealed highly dispersed atomic Ru clusters composed of fewer than 10 Ru atoms on the carbon support (**Supplementary Fig. 111**), while XAS analyses confirmed that the Ru clusters were stabilized through Ru-N coordination (CN = 1.53) and possessed a Ru-Ru CN of 1.92 comparable to that of aRu3/WC_x (CN = 1.72) (**Supplementary Fig. 112b-d, Supplementary**

Table 19). These results indicate that aRu/NC provides a suitable control system for examining the support effect while maintaining similar Ru ensemble coordination environments.

In addition, Ru nanoparticles (NPs) supported on WC_x and carbon (RuNP/WC_x and RuNP/C) were synthesized to directly examine the support effect for similar NP active-site structures (see *Catalyst synthesis* in *Methods*). HAADF-STEM analyses confirmed that both catalysts contained uniformly dispersed Ru NPs smaller than 2 nm (**Supplementary Fig. 113**). Furthermore, XRD, XAS, and XPS analyses revealed that the Ru NPs in both catalysts shared nearly identical structural and electronic characteristics except for the support-dependent interfacial interactions (**Supplementary Figs. 114, 115**). In particular, FT-EXAFS fitting showed nearly identical Ru-Ru CNs for RuNP/WC_x (CN = 2.27) and RuNP/C (CN = 2.35), whereas the Ru-O CN of RuNP/WC_x (CN = 2.93) was substantially smaller than that of RuNP/C (CN = 4.63), suggesting modified oxygen affinity induced by strong Ru-WC_x interactions (**Supplementary Table 19**).

The alkaline HER activities of these control catalysts were subsequently evaluated to experimentally distinguish the effects of neighboring Ru sites and support interactions (**Supplementary Fig. 116**). For catalysts possessing similar Ru ensemble coordination environments, aRu3/WC_x exhibited substantially superior HER activity (η_{10}: 22 mV, Tafel slope: 41.9 mV dec^{-1}) compared with aRu/NC (η_{10}: 128 mV, Tafel slope: 105.5 mV dec^{-1}), demonstrating the critical role of the WC_x support. Likewise, for catalysts possessing similar Ru NP structures, RuNP/WC_x exhibited better HER activity (η_{10}: 20 mV, Tafel slope: 30.8 mV dec^{-1}) than RuNP/C (η_{10}: 36 mV, Tafel slope: 52.1 mV dec^{-1}), further confirming the beneficial support effect of WC_x.

These results collectively indicate that the superior HER activity of aRu3/WC_x originates from the cooperative effects of (i) optimized hydrogen adsorption energetics induced by both the Ru ensemble size and strong Ru-WC_x interactions and (ii) enhanced proton transfer kinetics provided by the WC_x support compared with inert carbon supports (**Fig. 5g, h**, **Supplementary Note 12**). At the same time, comparison between aRu/NC and RuNP/C further suggests that neighboring Ru-Ru interactions themselves also strongly influence HER activity under inert-carbon supported conditions, as Ru NPs exhibited substantially higher activity than small Ru clusters supported on carbon. This trend is also consistent with the DFT results, which predict relatively non-ideal ΔG_H values for low-coordination Ru ensembles on inert carbon supports compared with Ru ensembles stabilized on γ-WC (**Fig. 4a, b and Supplementary Figs. 109, 110**).

Although RuNP/WC_x and aRu3/WC_x possess different active site geometries, both catalysts exhibited excellent HER activity owing to the beneficial Ru-WC_x interactions, together with the favorable interfacial water structure provided by the WC_x support. However, unlike Ru NPs, all Ru atoms in the Ru ALN structures are directly stabilized through chemical interactions with WC_x, enabling substantially stronger SMSI stabilization and consequently superior durability during long-term HER operation (**Supplementary Fig. 116d**).

Therefore, both the DFT calculations and experimental control studies consistently demonstrate that the superior alkaline HER activity of Ru ALNs originates from the cooperative contributions of (i) electronic structure modulation induced by both neighboring Ru-Ru interactions and strong Ru-WC_x interactions, (ii) adsorption-configuration changes enabled by neighboring Ru sites, and (iii) interfacial kinetic enhancement provided the WC_x support.

Revision

To address the reviewer's comment regarding the respective contributions of neighboring Ru-Ru interactions and WC_x support effects to the enhanced HER activity of Ru ALNs, additional DFT calculations and experimental control studies were performed. Comparative theoretical analyses using Ru ensembles supported on graphene and γ-WC were conducted to distinguish the effects of Ru ensemble size and support-induced electronic modulation on hydrogen adsorption energetics. In addition, control catalysts possessing similar Ru-Ru coordination environments on inert carbon supports or similar Ru nanoparticle structures on different supports were synthesized to experimentally distinguish the active-site structure effect and the WC_x support effect. Corresponding discussions and experimental results have been added to the revised Manuscript and Supplementary Information.

Additional discussions regarding the respective roles of Ru ensemble structures and WC_x support effects on hydrogen adsorption energetics, interfacial proton-transfer kinetics, and alkaline HER activity have been added to the revised Manuscript.

(Page 15-16 in the revised Manuscript)

To distinguish the effects of the active-site structure and WC_x support, additional DFT calculations were performed for Ru ensembles supported on graphene and γ-WC models, together with Bader charge and ΔG_H analyses (Supplementary Figs. 109, 110). Both the Bader charge and ΔG_H values varied with the Ru ensemble size on both supports, indicating that neighboring Ru-Ru interactions significantly influence the electronic structure and hydrogen adsorption energetics of Ru active sites. Furthermore, Ru ensembles on γ-WC consistently exhibited lower Bader charge values and ΔG_H values closer to the ideal hydrogen adsorption free energy than those on graphene, demonstrating that additional electronic modulation is induced through strong Ru-WC_x interactions. These results indicate that both the Ru ensemble structure and the WC_x support play critical roles in optimizing the hydrogen adsorption energetics for alkaline HER.

To experimentally distinguish the active-site structure effect and the WC_x support effect, atomically dispersed Ru clusters on N-functionalized carbon (aRu/NC) with a Ru-Ru CN of 1.92 comparable to that of aRu3/WC_x (CN = 1.72) were synthesized (Supplementary Fig. 111, 112). In addition, Ru NPs supported on WC_x and carbon (RuNP/WC_x and RuNP/C) were prepared to directly examine the support effect for similar NP active-site structures (Supplementary Fig. 113-115). During alkaline HER (Supplementary Fig. 116), aRu3/WC_x exhibited substantially superior HER activity (η_{10}: 22 mV, Tafel slope: 41.9 mV dec^{-1}) compared with aRu/NC (η_{10}: 128 mV, Tafel slope: 105.5 mV dec^{-1}), despite their similar Ru coordination environments. Likewise, RuNP/WC_x exhibited better HER activity (η_{10}: 20 mV,

Tafel slope: 30.8 mV dec^{-1}) than RuNP/C (η_{10}: 36 mV, Tafel slope: 52.1 mV dec^{-1}), further confirming the beneficial role of the WC_x support. These results collectively indicate that superior HER activity of aRu3/WC_x originates from the cooperative effects of (i) optimized hydrogen adsorption energetics induced by both the Ru ensemble size and strong Ru-WC_x interactions and (ii) enhanced proton-transfer kinetics arising from the favorable interfacial water structure provided by the WC_x support compared with inert carbon supports (Fig. 5g, h, Supplementary Note 12).

At the same time, comparison between aRu/NC and RuNP/C further suggests that neighboring Ru-Ru interactions themselves also strongly influence HER activity under inert carbon supported conditions, as Ru NPs exhibited substantially higher activity than small Ru clusters supported on carbon. This trend is also consistent with the DFT results, which predict relatively non-ideal ΔG_H values for low-coordination Ru ensembles on inert carbon supports compared with Ru ensembles stabilized on γ-WC (Fig. 4a, b, Supplementary Fig. 109, 110). Although RuNP/WC_x and aRu3/WC_x possess different active-site geometries, both catalysts exhibited excellent HER activity owing to the beneficial Ru-WC_x interactions, together with the favorable interfacial water structure provided by the WC_x support. However, unlike Ru NPs, all Ru atoms in the Ru ALN structures are directly stabilized through chemical interactions with WC_x, enabling substantially stronger SMSI stabilization and consequently superior durability during long-term HER operation (Supplementary Fig. 116d).

Therefore, both the DFT calculations and experimental control studies consistently demonstrate that the superior alkaline HER activity of Ru ALNs originates from the cooperative contributions of (i) electronic structure modulation induced by both neighboring Ru-Ru interactions and strong Ru-WC_x interactions, (ii) adsorption-configuration changes enabled by neighboring Ru sites, and (iii) interfacial kinetic enhancement provided the WC_x support.

Additional supplementary figures related to DFT calculations, structural analyses, and alkaline HER activities of control catalysts including aRu/NC, RuNP/WCx, and RuNP/C have been added to the revised Supplementary Information.

(Page 127-134 in the revised Supplementary Information)

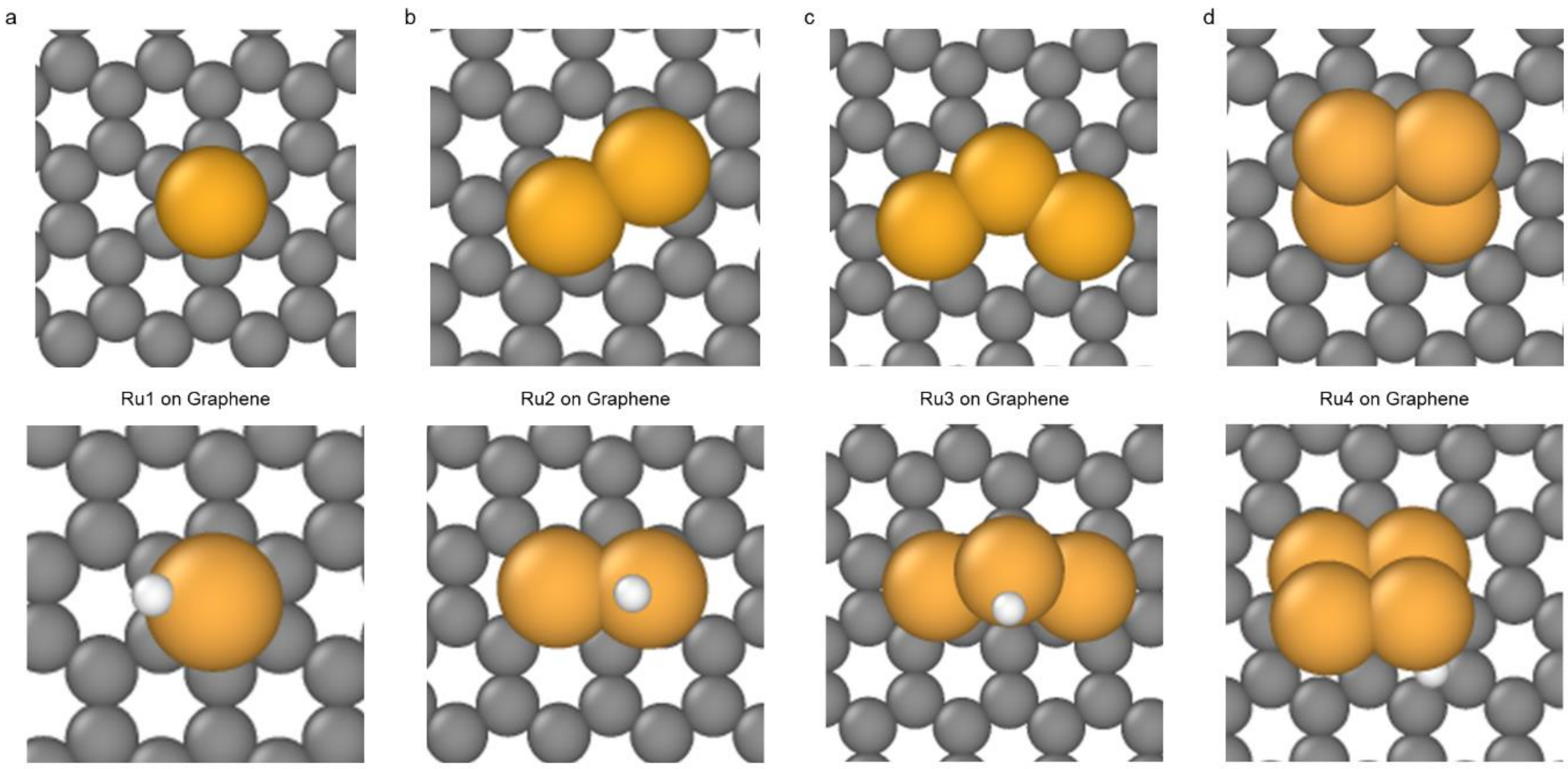

Supplementary Fig. 109 | DFT-optimized structures of Ru ensembles on graphene. a-d, Optimized atomic configurations of (**a**) Ru1, (**b**) Ru2, (**c**) Ru3, and (**d**) Ru4 ensembles supported on graphene together with the corresponding *H adsorbed configurations, which were used for the Bader charge analyses and ΔG_H calculations.

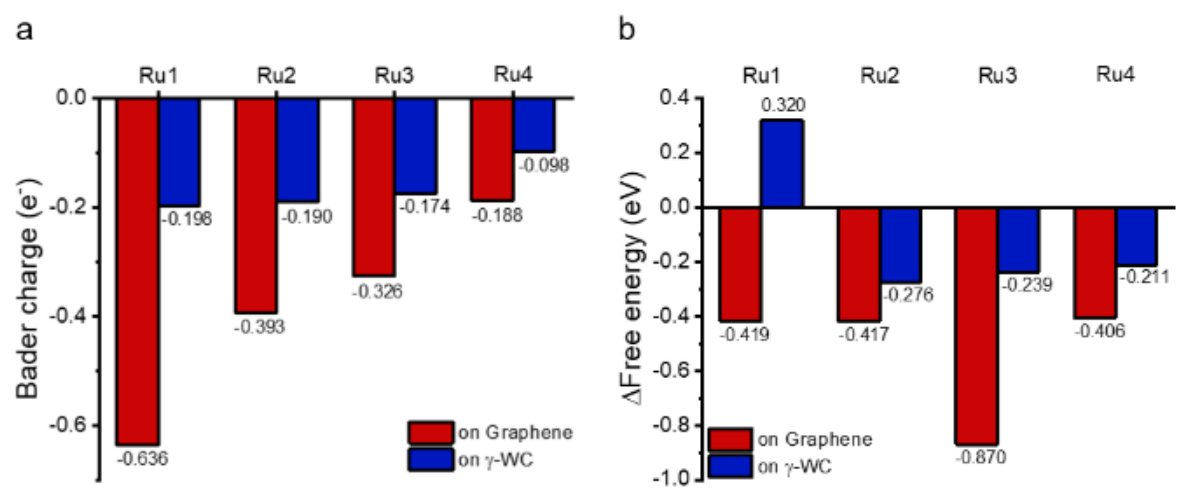

Supplementary Fig. 110 | Electronic structure and hydrogen adsorption properties of Ru ensembles on different supports. a, Bader charge analyses, and **b,** calculated ΔG_H for Ru ensembles supported on graphene and γ-WC.

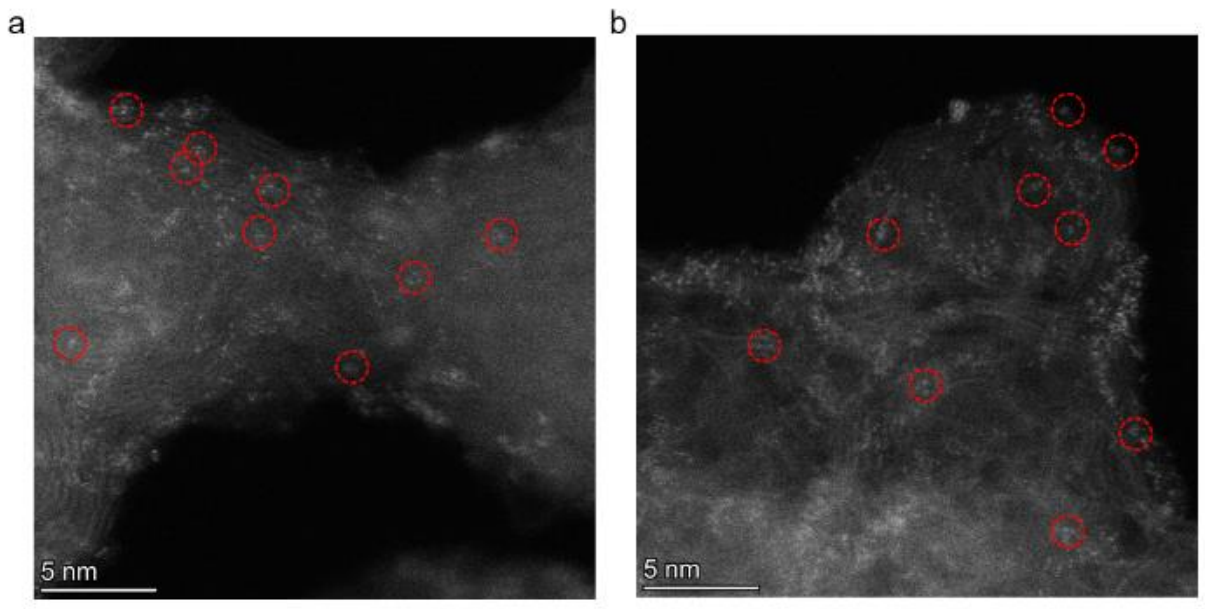

Supplementary Fig. 111 | Atomic-scale structure of aRu/NC. a-b, Representative HAADF-STEM images of aRu/NC (red dotted circles: Ru clusters composed of fewer than 10 Ru atoms)

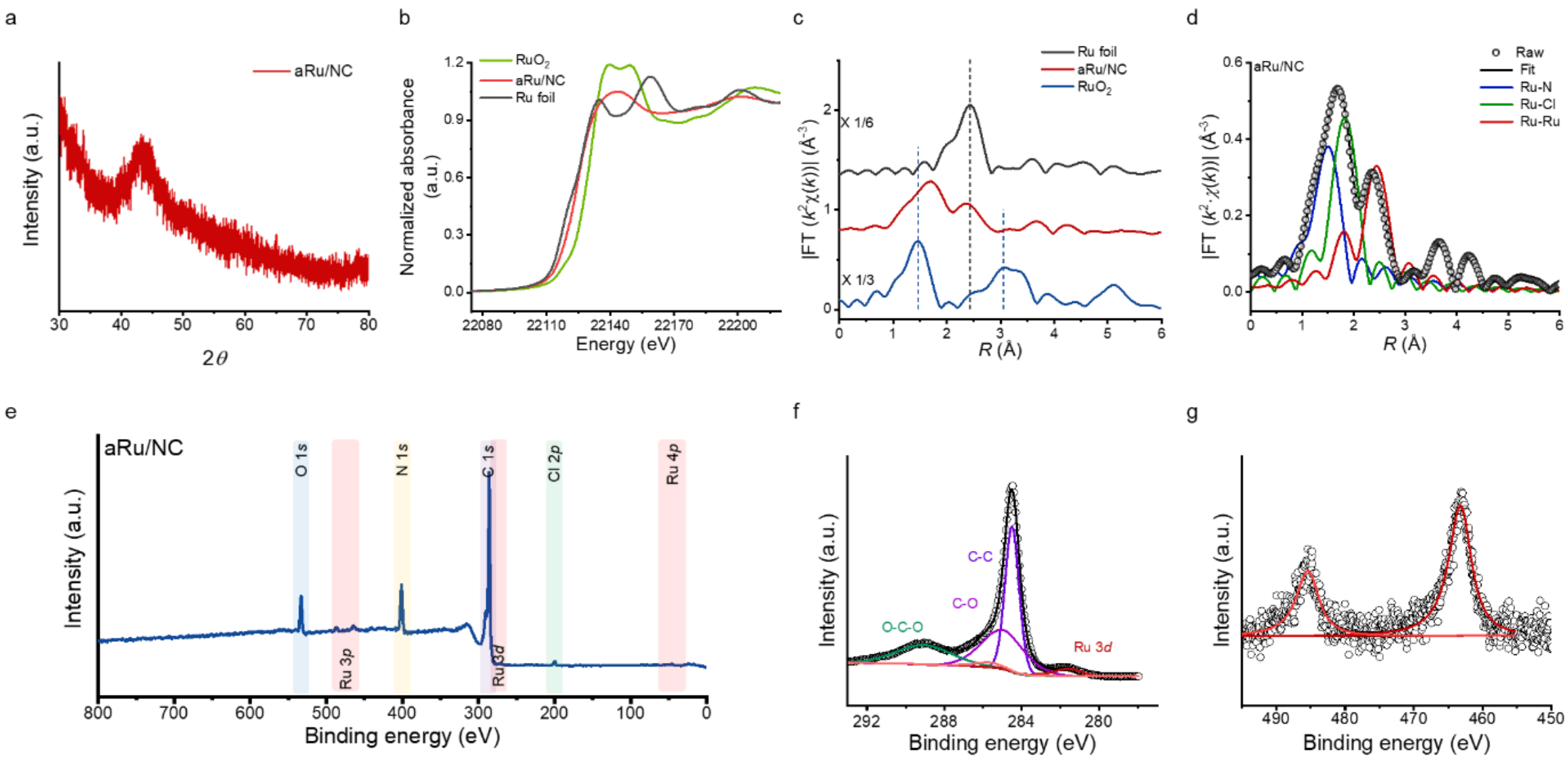

Supplementary Fig. 112 | Geometric and electronic structure of aRu/NC. a, XRD pattern of aRu/NC. **b-d,** XAS analyses of aRu/NC at the Ru K-edge, including (**b**) XANES spectra, (**c**) k^2-weighted FT-EXAFS spectra, and (**d**) corresponding FT-EXAFS fitting results. **e-g,** XPS analyses of aRu/NC, including (**e**) survey spectrum, (**f**) C 1s spectrum, and (**g**) Ru 3p spectrum.

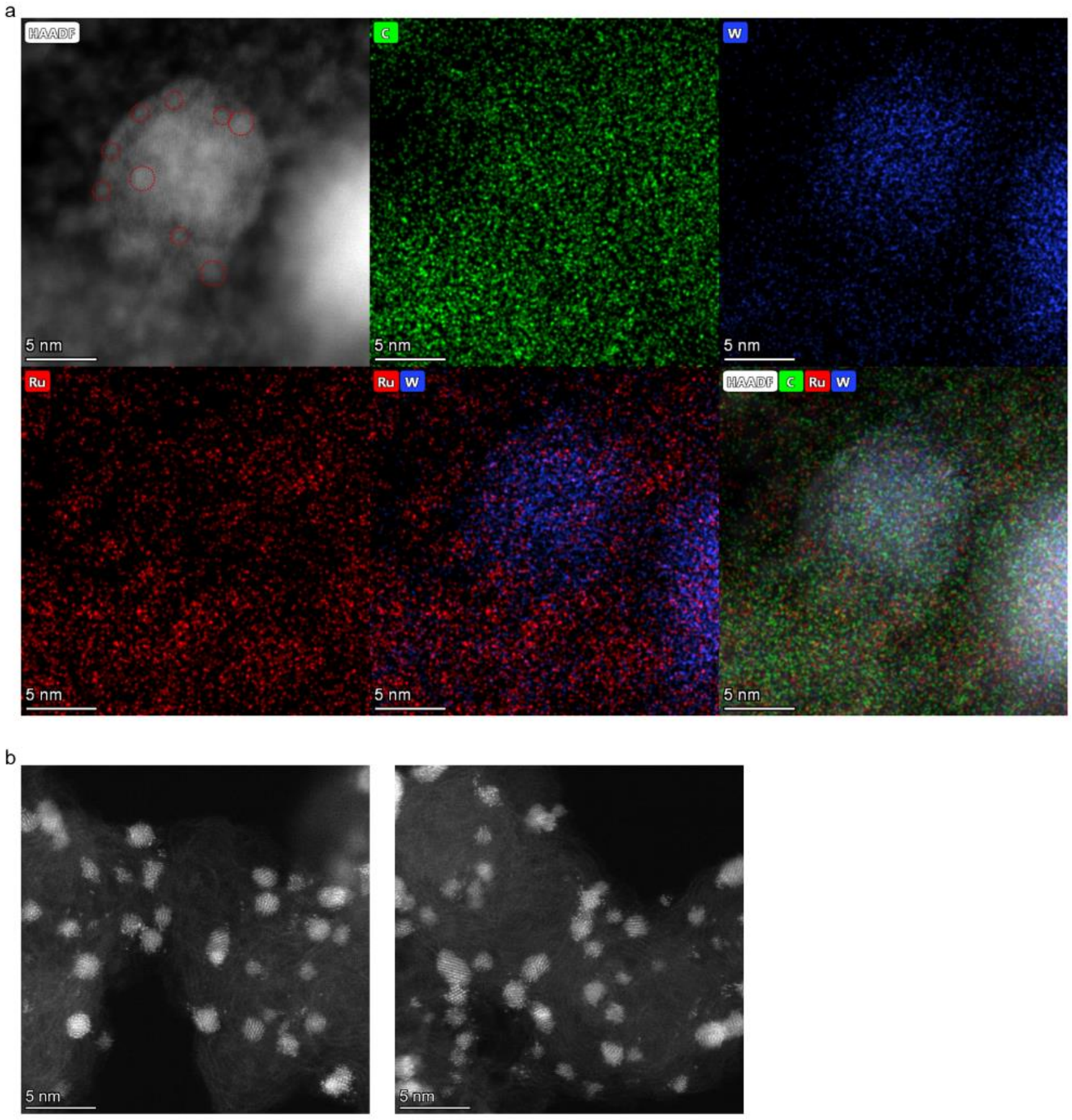

Supplementary Fig. 113 | STEM analysis of RuNP/WC_x and RuNP/C. a, HAADF-STEM image and the corresponding EDS elemental mappings of RuNP/WC_x for C (green), W (blue), Ru (red), along with the overlaid composite map (red dotted circle: Ru NPs). **b,** HAADF-STEM images of RuNP/C.

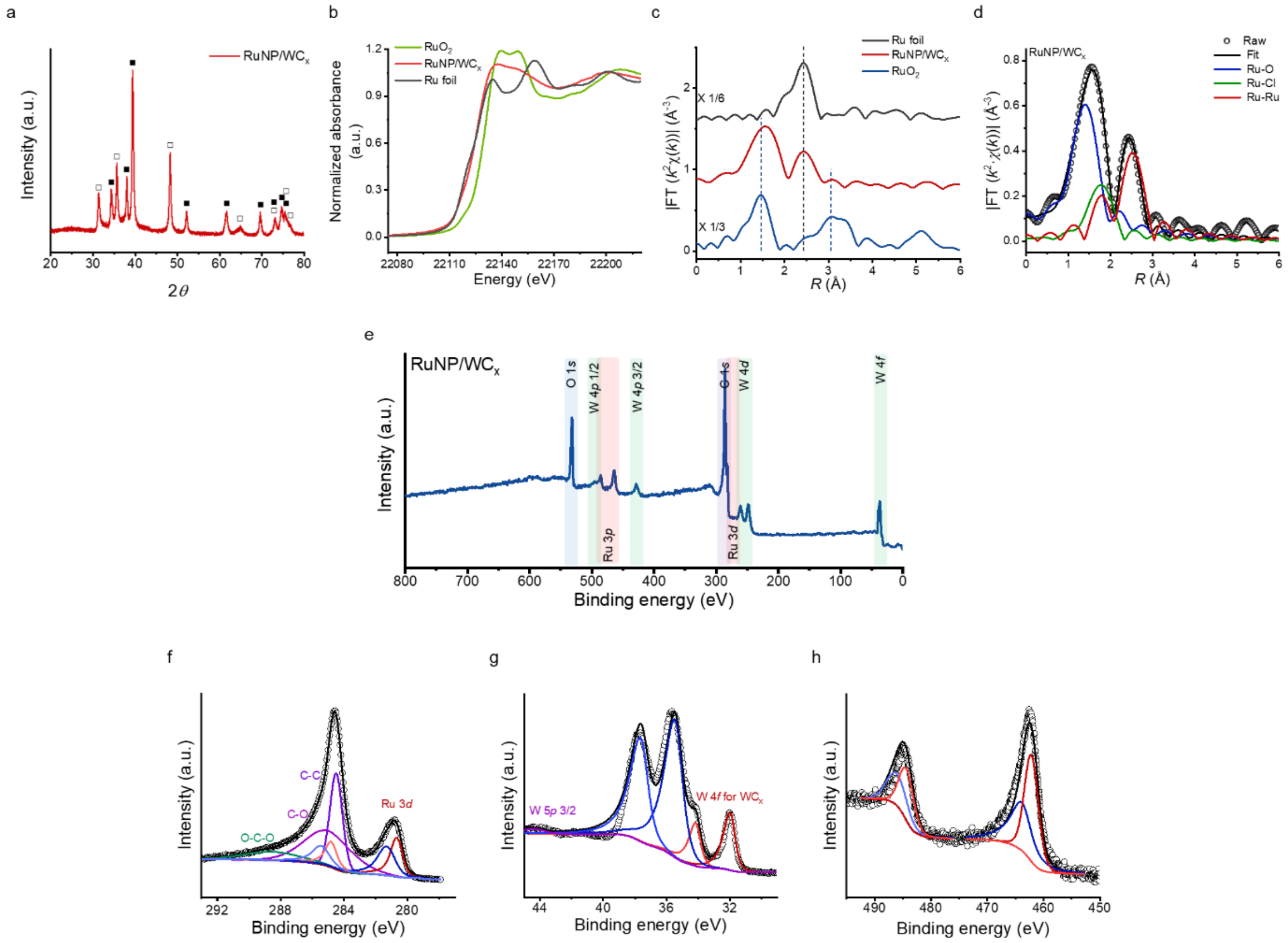

Supplementary Fig. 114 | Geometric and electronic structure of RuNP/WC$_x$. a, XRD pattern of RuNP/WC$_x$. **b-d,** XAS analyses of RuNP/WC$_x$ at the Ru K-edge, including (**b**) XANES spectra, (**c**) k^2-weighted FT-EXAFS spectra, and (**d**) corresponding FT-EXAFS fitting results. **e-h,** XPS analyses of RuNP/WC$_x$, including (**e**) survey spectrum, (**f**) C 1s spectrum, (**g**) W 4f spectrum, and (**h**) Ru 3p spectrum.

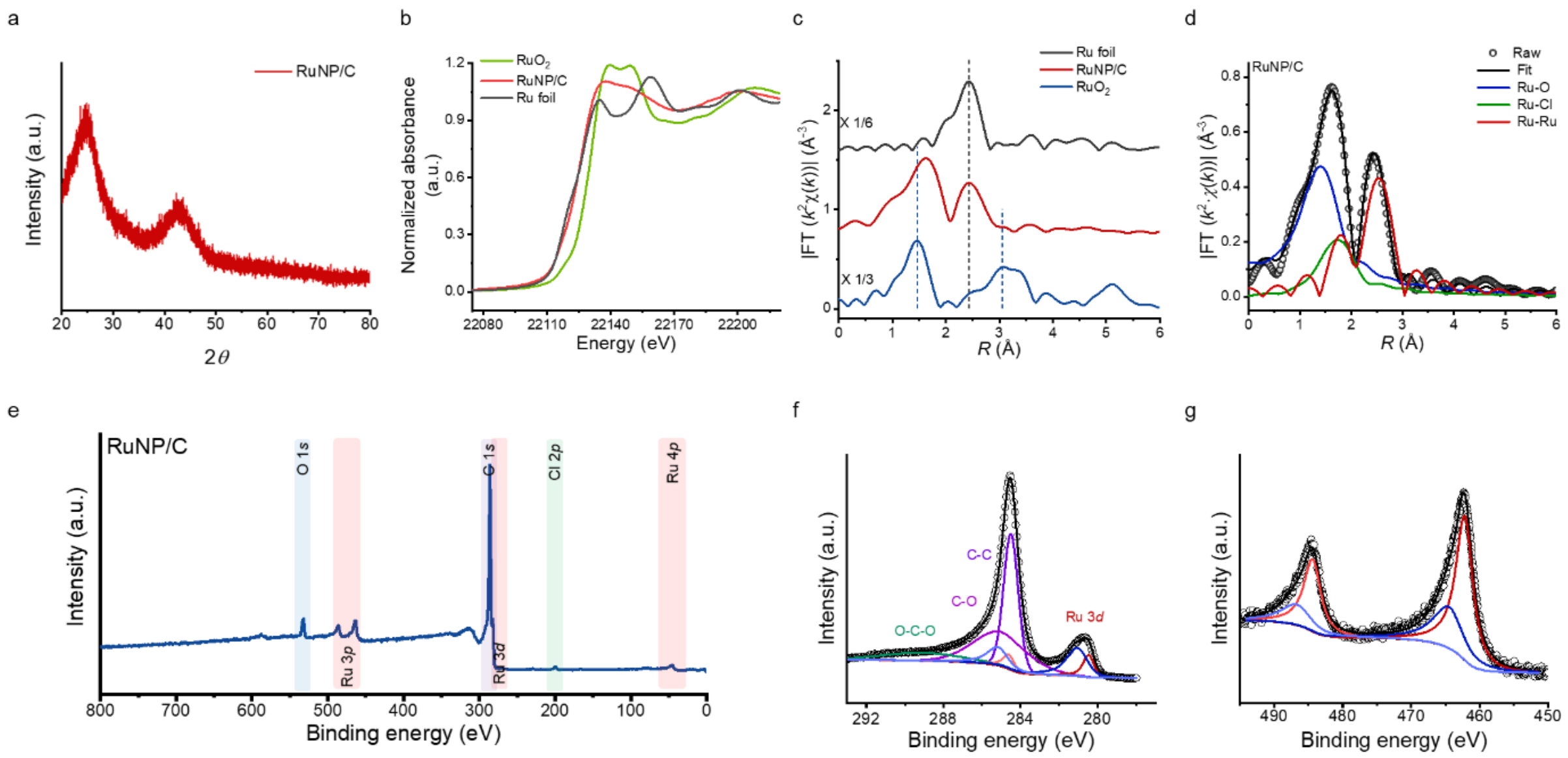

Supplementary Fig. 115 | Geometric and electronic structure of RuNP/C. a, XRD pattern of RuNP/C. **b-d,** XAS analyses of RuNP/C at the Ru K-edge, including (**b**) XANES spectra, (**c**) k^2-weighted FT-EXAFS spectra, and (**d**) corresponding FT-EXAFS fitting results. **e-h,** XPS analyses of RuNP/C, including (**e**) survey spectrum, (**f**) C 1s spectrum, and (**g**) Ru 3p spectrum.

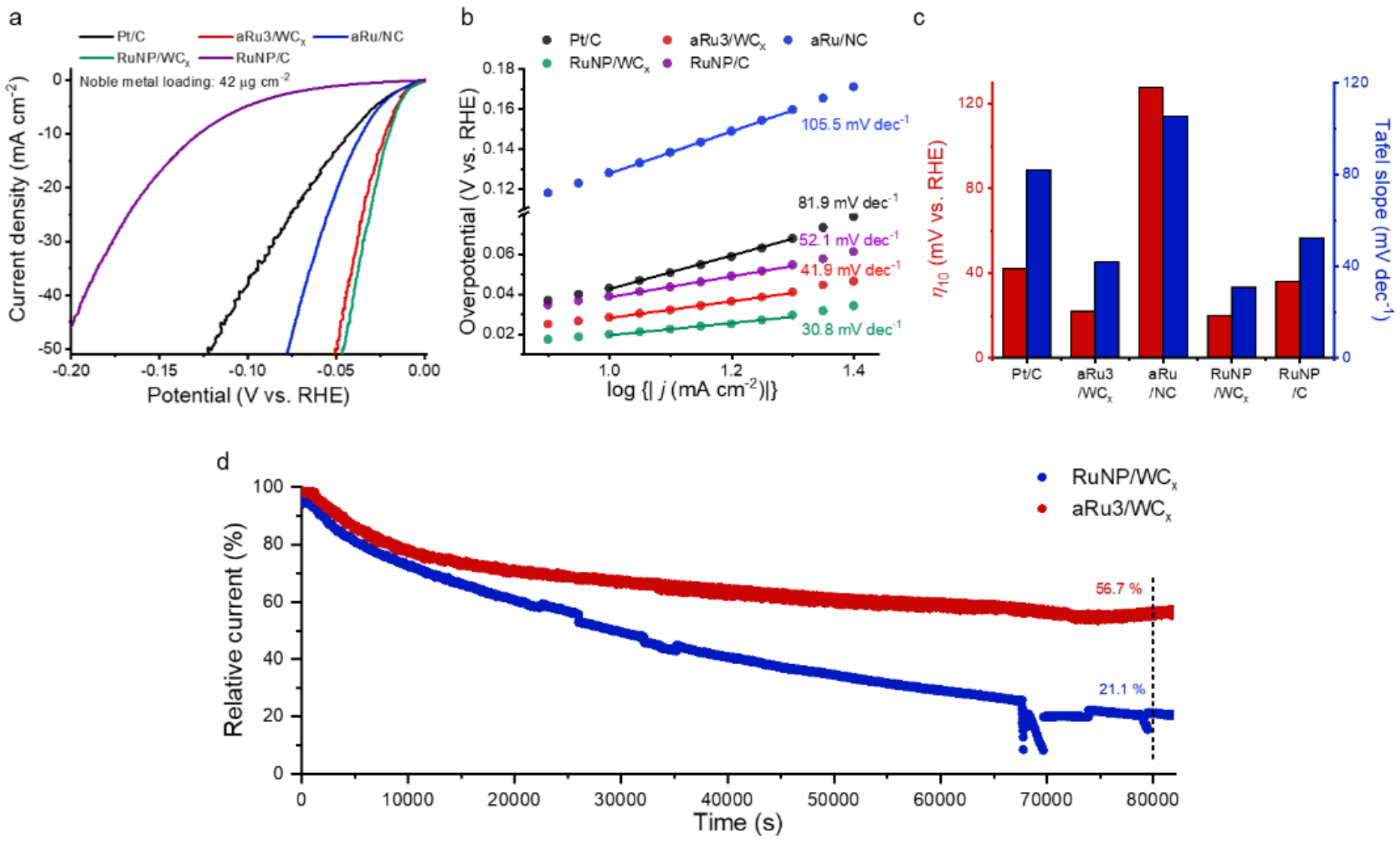

Supplementary Fig. 116 | Alkaline HER activity for aRu/NC, RuNP/WC$_x$, and RuNP/C. a, LSV curves, **b,** corresponding Tafel plot, **c,** corresponding η_{10} and Tafel slope, and **d,** relative current retention (%) during chronoamperometry at an overpoential of 50 mV over 80,000 s.

Additional EXAFS fitting results for the control catalysts including aRu/NC, RuNP/WC_x, and RuNP/C have been added to the revised Supplementary Information.

(Page 172 in the revised Supplementary Information)

Supplementary Table 19 | Structural parameters extracted from EXAFS fitting analyses. Structural parameters extracted from the Ru K-edge, Pt L_2-edge, Ir L_2-edge EXAFS fitting results. CN: coordination number, R: interatomic distance, and σ^2: Debye-Waller factor. The goodness of fit is represented by the R-factor. (weighting: k^2)

Sample	Shell	CN	R (Å)	σ^2 (10^{-3} Å^2)	R-factor
Ru	Ru-Ru	6*	2.65 ± 0.01	2.56	0.007
	Ru-Ru	6*	2.70 ± 0.01		
RuO_2	Ru-O	2*	1.94 ± 0.01	0.73	0.014
	Ru-O	4*	1.98 ± 0.01		
Ru/WC_x_R	Ru-O	2.06 ± 0.33	1.97 ± 0.01	5.69	0.012
	Ru-Ru	4.51 ± 0.41	2.66 ± 0.01	6.93	
Ru/C_R	Ru-O	2.79 ± 0.37	1.97 ± 0.01	6.95	0.014
	Ru-Ru	3.70 ± 0.41	2.67 ± 0.01	6.45	
RuNP/WC_x	Ru-O	2.93 ± 0.19	1.94 ± 0.01	5.87	0.003
	Ru-Cl	0.86 ± 0.14	2.30 ± 0.01		
	Ru-Ru	2.27 ± 0.23	2.66 ± 0.01	5.74	
RuNP/C	Ru-O	4.63 ± 0.19	1.97 ± 0.00	17.23	0.001
	Ru-Cl	1.45 ± 0.14	2.27 ± 0.01		
	Ru-Ru	2.35 ± 0.11	2.66 ± 0.00	5.00	
aRu/NC	Ru-N	1.53 ± 0.06	2.01 ± 0.00	1.75	0.001
	Ru-Cl	1.06 ± 0.03	2.33 ± 0.00		
	Ru-Ru	1.92 ± 0.08	2.61 ± 0.00	8.14	
aPt3/WC_x**	Pt-C	1.59 ± 0.30	2.08 ± 0.02	2.16	0.009
	Pt-Pt	4.10 ± 0.34	2.75 ± 0.01	2.07	
	Pt-W	1.93 ± 0.37	2.88 ± 0.01		
aIr3/WC_x**	Ir-C	2.46 ± 0.08	2.11 ± 0.00	2.69	0.001
	Ir-Ir	1.87 ± 0.09	2.73 ± 0.00	1.49	
	Ir-W	1.06 ± 0.09	2.88 ± 0.01		

Structural parameters extracted from the EXAFS fitting of reference metal foil. CN is the coordination number; R is the interatomic distance; σ^2 is the Debye-Waller factor (a measure of the static and thermal disorder in absorber-scatterer distance); ΔE_0 is edge energy shift (the zero kinetic energy difference between experiment and theoretical model); R-factor value is related with the goodness of the fitting.
* Value was assigned in curving fitting based on standard structure
** For Pt and Ir, EXAFS fitting was conducted at the L_2-edge to avoid spectral overlap between the L_3-edge and the W L_2-edge. S_0^2 value was obtained from reference foil measurements at the corresponding L_2-edge
R_{bkg} was set to 1.0 in background removal to remove the noise oscillation below 1 Å in R-space
S_0^2 value (0.744) was calculated from the fitting of metal foil with standard metal structure

Experimental procedures for the synthesis of aRu/NC, RuNP/WC_x, and RuNP/C have been added to the revised *Experimental* section.

(Page 22-23 in the *Experimental* section of the revised Manuscript)

Synthesis of aRu/NC. For control experiments, aRu/NC were prepared by IWI using soft-nitrided Vulcan XC 72R (*J. Am. Chem. Soc.*, **2016**, 138, 4718-4721). For the synthesis, 27.2 mg of HPL Ru was dissolved in 20 mL of ethanol, sonicated for 10 min, and stirred for 10 min to obtain a homogeneous solution. Next, 0.2 g of soft-nitrided carbon was added to the solution, dispersed by sonication for 10 min, then stirred at 323 K until complete solvent evaporation. The resulting powder was dried at 323 K under vacuum for 12 h. It was then annealed in a tube furnace under H_2 (20 sccm) and Ar (180 sccm) flow at 473 K for 2 h (1 K min^{-1}), then *RT* (5 K min^{-1}). After cooling to *RT*, the sample was passivated under O_2 (2 sccm) and Ar (200 sccm) flow for 12 h, and finely ground in an agate mortar for 10 min.

Synthesis of RuNP/WC_x and RuNP/C. 0.16 g of HPL Ru was dissolved in 20 mL of ethylene glycol in vial. Separately, 0.4 g of NaOH was dissolved in 20 mL of ethylene glycol in three-neck round-bottom flask. After complete dissolution, the two solutions were combined and vigorously stirred for 1 h under continuous Ar purging to remove dissolved oxygen. The resulting solution was then heated to 433 K for 3 hr (4 K min^{-1}), followed by naturally cooling to *RT*. A stable dispersion of Ru NPs in ethylene glycol was obtained. For ligand removal and support immobilization, 5 mL of Ru NP dispersion was mixed with 30 mL of 1.0 M HCL solution in a conical tube after ultrasonication of the dispersion. The mixture was centrifuged, and the supernatant was discarded. This washing process was repeated three times using a total of 15 mL of Ru NP dispersion. Subsequently, the precipitate was additionally washed with 30 mL and then 10 mL of 1.0 M HCl solution via centrifugation to collect the Ru NPs at the bottom of the tube. After acid washing, 117 mg of support materials (WCx or Vulcan XC 72R) and 5 mL of acetone were added to the conical tube. The resulting mixture was ultrasonicated for 1 h in an ice bath while preventing solvent evaporation. The obtained catalyst powder was collected by centrifugation and dried under vacuum at 323 K overnight. The dried samples were subsequently annealed in a tube furnace under H_2 (20 sccm) and Ar (180 sccm) flow at 473 K for 2 h (1 K min^{-1}), followed by an additional 2 h under pure Ar (200 sccm). The samples were then cooled to *RT* (5 K min^{-1}) and passivated under O_2 (2 sccm) and Ar (200 sccm) flow for 12 h.

4. Under high current density operation, evaluating the Faraday efficiency of hydrogen production is crucial. It is recommended that the author employ gas chromatography (GC) or the displacement gas collection method to monitor hydrogen production at the AEMWE outlet in real time and calculate Faraday efficiency.

Reply

We thank the reviewer for this valuable suggestion and apologize for the lack of clarification in the original Manuscript.

In response, the generated H_2 was quantified using a mass flow meter (MFM; calibrated for H_2 at 0 °C and 1 atm) installed at the cathode outlet of the electrolyzer, enabling real-time monitoring of the hydrogen evolution rate (**Supplementary Fig. 123**). Under multistep chronopotentiometric operation from 1.0 to 5.0 A cm^{-2}), the experimentally measured hydrogen flow rates closely match the theoretical values calculated from Faraday's law across the entire current density range, corresponding to high Faradaic efficiency of 96.0–97.8 % (**Supplementary Fig. 124**). We attribute the minor deviation from the theoretical values to experimental factors such as minor gas loss and dead volume in the gas-handling line rather than parasitic electrochemical reactions.

In addition, gas chromatography (GC) analysis of the cathode outlet stream confirmed that hydrogen was the dominant product with no detectable impurity peaks (**Supplementary Fig. 125**). A minor oxygen peak was observed, which is attributed to oxygen crossover through the membrane.

These results confirm that the current is predominantly utilized for hydrogen evolution even under high-current-density AEMWE operation.

Revision

Real-time hydrogen quantification and Faradaic efficiency analyses were added for high-current-density AEMWE operation using a mass flow meter and gas chromatography (GC) analysis to validate hydrogen production under practical operating conditions.

Additional descriptions regarding real-time hydrogen quantification, Faradaic efficiency validation, and product analysis under high-current-density AEMWE operation have been added to the revised Manuscript and Supplementary Information.

(Page 18 in the revised Manuscript)

All samples exhibited nearly 100 % Faradaic efficiency for H_2 based on real-time hydrogen quantification using a mass flow meter and gas chromatography (GC) analysis of the cathode outlet stream (Fig. 5e, Supplementary Figs. 123-126), indicating that the measured current predominantly originated from hydrogen evolution.

The experimental setup for real-time hydrogen quantification during high-current-density AEMWE operation was added in the revised Supplementary Information.

(Page 141 in the revised Supplementary Information)

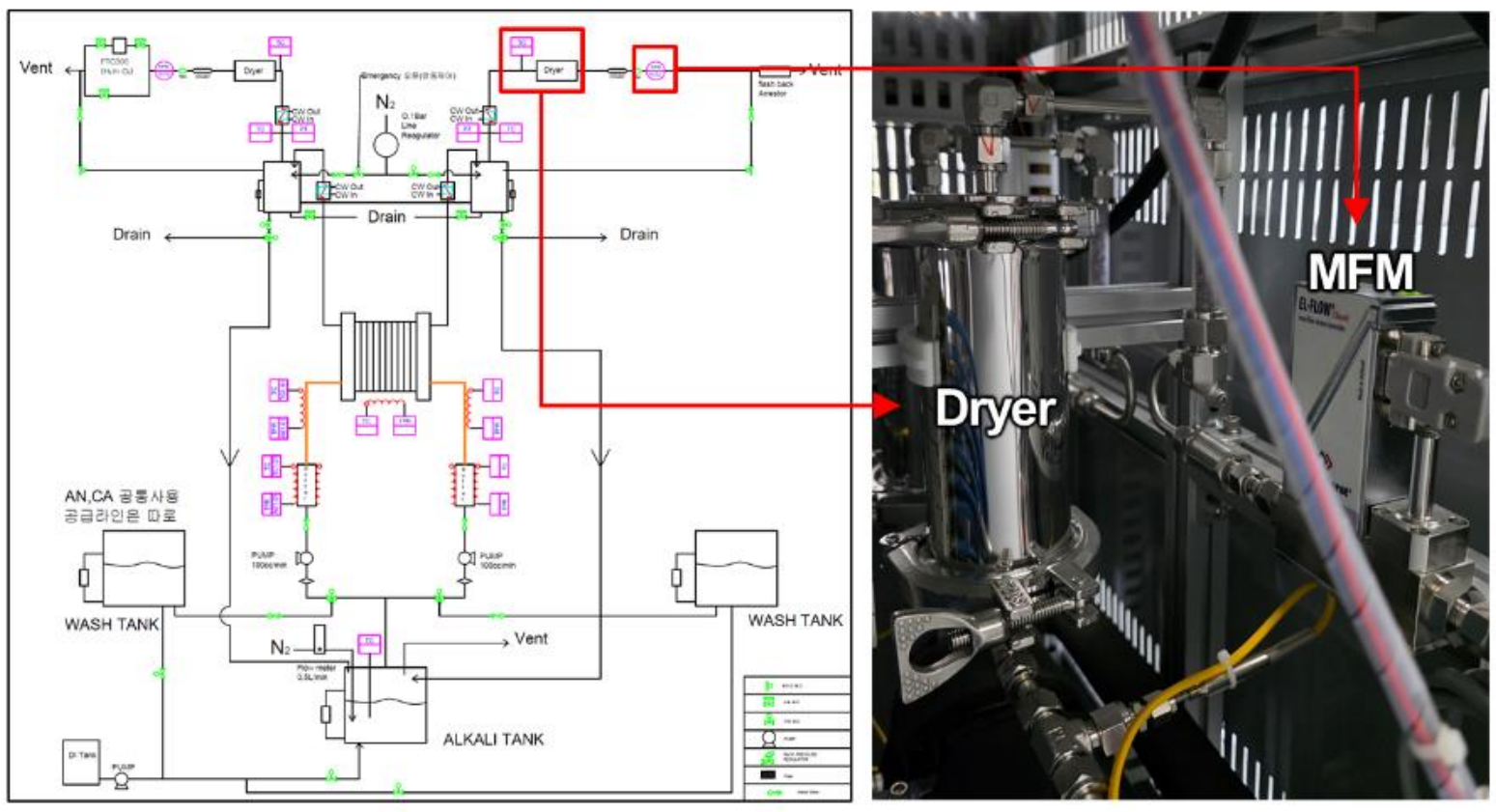

Supplementary Fig. 123 | Experimental setup for hydrogen quantification under high-current-density AEMWE operation. (left) Schematic flow diagram of the electrolyzer system. (right) Photograph of the experimental setup showing the dryer and mass flow meter (MFM) installed at the cathode outlet for real-time hydrogen flow measurement.

Validation of Faradaic efficiency by comparing experimentally measured and theoretically calculated hydrogen evolution rates was added in the revised Supplementary Information.

(Page 142 in the revised Supplementary Information)

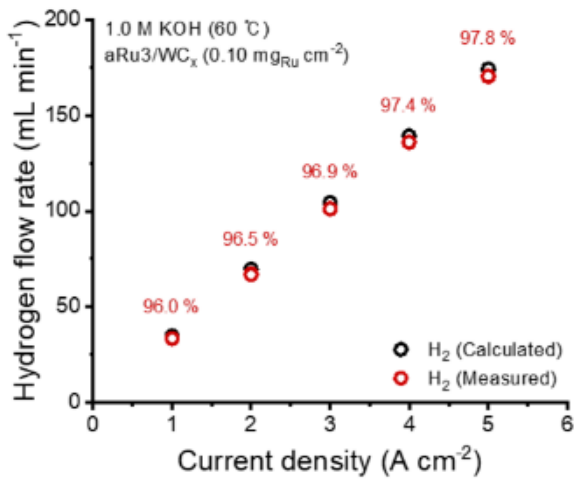

Supplementary Fig. 124 | Validation of Faradaic efficiency under high-current-density AEMWE operation. Comparison between the experimentally measured hydrogen evolution rate and the theoretical values calculated from Faraday's law as a function of current density during chronopotentiometric operation using aRu3/WC_x (0.10 mg_{Ru} cm^{-2}) in 1.0 M KOH at 60 ℃.

Gas chromatography (GC) analysis of the cathode outlet stream was added to confirm the selective generation of H_2 during AEMWE operation.

(Page 143 in the revised Supplementary Information)

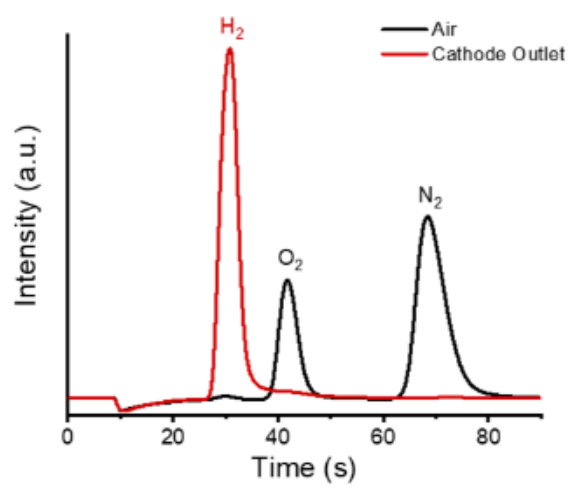

Supplementary Fig. 125 | Gas chromatography (GC) analysis. GC analysis of the cathode outlet stream under AEMWE operation. The cathode outlet stream is dominated by H_2 with no detectable impurity peaks, while a minor O_2 peak is attributed to oxygen crossover through the membrane.

5. Under ultra-high current densities, bubble coverage and gas transport limitations often dominate the apparent cell voltage and can obscure intrinsic catalytic activity. Can the authors clarify whether the Ru ALN morphology leads to any measurable difference in gas bubble behavior compared to conventional Ru nanoparticles (e.g., bubble detachment frequency, bubble size distribution, or gas coverage)? If possible, please provide high-speed visualization or contact angle measurements, as well as iR-compensated polarization curves or EIS analysis to support that the improved performance is not primarily governed by bubble shielding or mass transport effects.

Reply

We thank the reviewer for this insightful comment regarding the possible influence of catalyst-dependent bubble behavior and mass transport limitations under high-current-density AEMWE operation.

As suggested by the reviewer, we systematically investigated whether catalyst structural features, including Ru ALN morphology, Ru aggregation, and support properties, influence wettability, bubble evolution behavior, and mass transport characteristics using aRu3/WC_x, aRu5/WC_x, aRu5$_p$/WC_x, commercial Ru/C, and commercial Pt/C cathodes under identical noble metal loadings (0.10 mg_{mass} cm^{-2}). This comparison allows us to evaluate whether the superior performance of aRu3/WC_x originates from intrinsically enhanced catalytic activity rather than differences in bubble shielding or gas transport behavior.

Contact angle analysis

First, static water contact angle measurements were performed using catalyst layers deposited on non-porous Grafoil substrates to evaluate the intrinsic wettability of the catalyst layers (**Supplementary Fig. 128**). Most catalysts exhibited comparable contact angles (105.3–121.4°), except for aRu5$_p$/WC_x (83.7°), indicating that differences in surface wettability among Pt/C, Ru/C, aRu3/WC_x, and aRu5/WC_x are relatively small. In particular, aRu3/WC_x and aRu5/WC_x showed similar contact angles despite differences in Ru ALN ensemble size, suggesting that the Ru ALN morphology does not substantially alter the macroscopic wettability of the catalyst layer. In addition, the similar wettability of aRu3/WC_x to those of commercial Pt/C and Ru/C suggests that the enhanced AEMWE performance of aRu3/WC_x cannot be explained primarily by differences in surface wettability.

Dynamic bubble behavior

To further investigate gas evolution behavior, real-time optical visualization of hydrogen bubble formation was conducted under alkaline HER conditions using a custom-built transparent electrochemical cell (**Supplementary Fig. 129**). Although this setup does not operate under ultra-high-current-density conditions, it enables direct comparison of bubble nucleation, growth, detachment, and surface coverage across different catalyst layers. As a validation of the sensitivity of this setup, we additionally provide reviewer-only reference data from an independent study, where

pronounced morphology-dependent bubble behavior was successfully resolved using the same visualization system (**Fig. R2**).

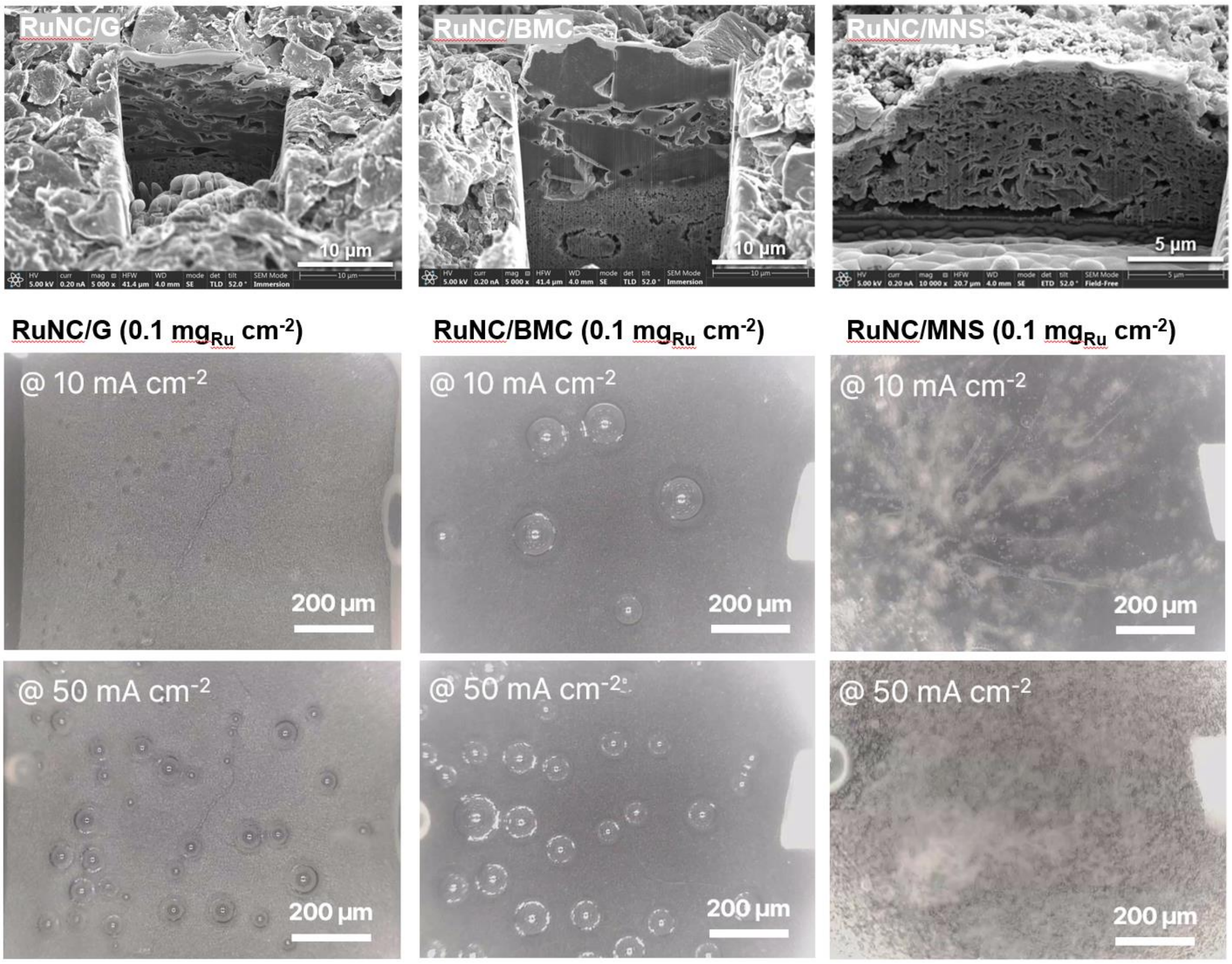

Fig. R2 | SEM images (top) and corresponding optical images of gas bubbles generated during alkaline HER (bottom) on RuNC/G, RuNC/BMC, and RuNC/MNS electrodes.

Note. These data are part of an independent study (accepted in *Joule*) and are therefore not included in the present manuscript. They are provided here as reviewer-only information to demonstrate that the experimental setup is capable of resolving differences in dynamic bubble behavior.

At 10 mA cm^{-2}, Pt/C and WC_x-supported catalysts, including aRu3/WC_x, aRu5/WC_x, and aRu5$_p$/WC_x, exhibited similarly rapid microbubble detachment behavior with no noticeable differences in bubble size or surface coverage (**Supplementary Fig. 129**). Notably, aRu3/WC_x and aRu5/WC_x showed comparable bubble evolution behavior, indicating that the Ru ALN morphology does not lead to noticeable differences in gas bubble behavior under the present conditions. Moreover, aRu5$_p$/WC_x, which contains larger Ru agglomerates, also showed bubble release behavior similar to that of aRu3/WC_x and aRu5/WC_x, suggesting that Ru aggregation within the WC_x-supported catalyst layer is not the dominant factor governing bubble detachment or gas coverage. In contrast, Ru/C showed localized bubble accumulation (red dotted circles in **Supplementary Fig. 129b**), which may be associated with differences in catalyst layer morphology and local gas transport characteristics.

Overpotential breakdown analysis

To further determine whether mass transport limitations dominate the observed AEMWE performance, we additionally performed multistep chronopotentiometry combined with electrochemical impedance spectroscopy (EIS) to deconvolute the overpotential contributions under pseudo-steady-state conditions (**Supplementary Fig. 130**). The ohmic contribution was removed using EIS-derived resistance values, and the residual overpotential was analyzed after subtraction of the kinetic contribution extracted from the Tafel region. Notably, Pt/C, aRu3/WC_x, aRu5/WC_x, and aRu5_p/WC_x exhibited comparable residual overpotentials, whereas Ru/C showed a substantially larger residual contribution. This trend is consistent with the bubble visualization results.

Importantly, the comparable residual overpotentials of aRu3/WC_x and aRu5/WC_x indicate that differences in Ru ALN morphology do not substantially affect the mass-transport-related overpotential. In addition, the similar residual overpotential of aRu5_p/WC_x suggests that the presence of larger Ru agglomerates in the WC_x-supported catalyst layer does not significantly increase mass-transport-related losses. Therefore, the superior performance of aRu3/WC_x over Pt/C and the other comparison catalysts cannot be attributed primarily to reduced bubble shielding or lower mass transport resistance.

Taken together, these results demonstrate that the improved AEMWE performance of aRu3/WC_x does not primarily originate from differences in bubble coverage, bubble detachment behavior, or gas transport limitations. Instead, the performance enhancement is mainly attributed to the intrinsically enhanced catalytic activity and favorable electrode kinetics of the Ru ALNs on WC_x.

Revision

Additional analyses regarding catalyst-dependent bubble behavior and mass transport effects during high-current-density AEMWE operation were added to the revised Manuscript and Supplementary Information. Specifically, contact angle measurements, *in-situ* optical visualization of hydrogen bubble evolution, and EIS-based overpotential breakdown analyses were performed to clarify that the superior performance of aRu3/WC_x does not primarily governed by bubble shielding or mass transport limitations.

Additional discussions on the effects of catalyst structural characteristics, including Ru ALN morphology, Ru aggregation, and support properties, on bubble coverage and mass transport limitations during high-current-density AEMWE operation were added to the revised Manuscript.

(Page 19 in the revised Manuscript)

To examine whether the superior high-current-density AEMWE performance originates from catalyst-dependent bubble behavior or mass transport effects, we systematically investigated the influence of catalyst structural features, including Ru ALN morphology, Ru aggregation, and support properties, on wettability, bubble evolution, and mass transport characteristics using aRu3/WC_x, aRu5/WC_x, aRu5_p/WC_x, commercial Ru/C and Pt/C cathodes. Most catalysts exhibited comparable contact angles (105.3−121.4°), except for aRu5_p/WC_x (83.7°) (Supplementary Fig. 128). In addition, Pt/C and WC_x-supported catalysts exhibited similarly rapid microbubble detachment behavior with no noticeable differences in bubble size or surface coverage during real-time optical visualization of hydrogen bubble evolution, whereas Ru/C showed localized bubble accumulation (Supplementary Fig. 129). Furthermore, multistep chronopotentiometry combined with EIS revealed comparable residual overpotentials for Pt/C and WC_x-supported catalysts, while Ru/C exhibited a substantially larger residual contribution (Supplementary Fig. 130). Notably, aRu3/WC_x and aRu5/WC_x showed similar residual overpotentials, indicating that differences in Ru ALN morphology do not substantially affect mass-transport-related losses. These results demonstrate that the superior AEMWE performance of aRu3/WC_x does not primarily originate from bubble shielding or gas transport effects, but rather from the intrinsically enhanced catalytic activity and favorable interfacial kinetics of the Ru ALNs on WC_x.

Water contact angle analyses were added in the revised Supplementary Information to evaluate the wettability of the catalyst layers.

(Page 146 in the revised Supplementary Information)

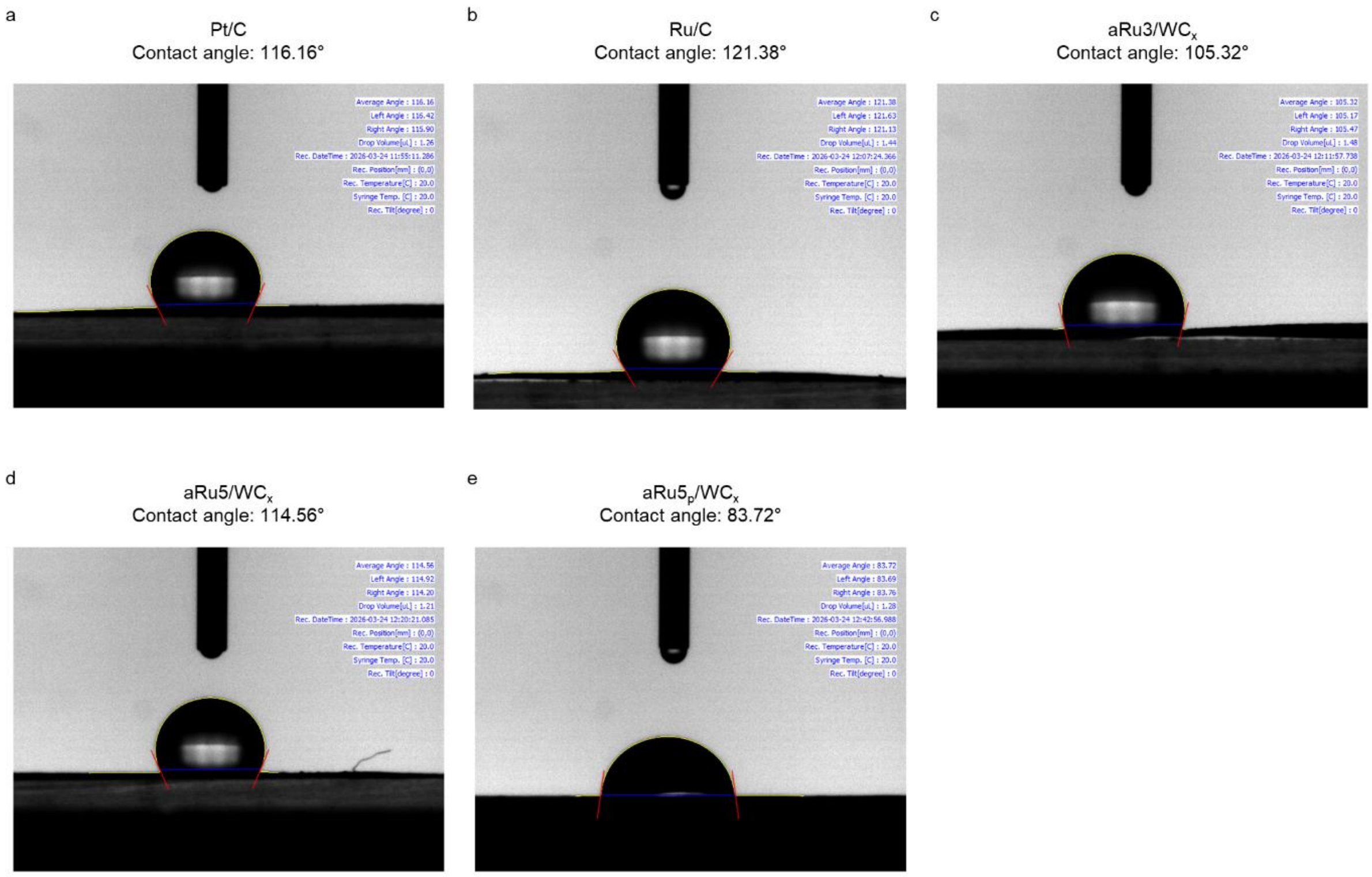

Supplementary Fig. 128 | Water contact angle analysis of catalyst layers. a-e, Water contact angle measurements for catalyst layers prepared with (**a**) commercial Pt/C, (**b**) commercial Ru/C, (**c**) aRu3/WC$_x$, (**d**) aRu5/WC$_x$, and (e) aRu5$_p$/WC$_x$. All catalyst layers were prepared with an identical noble metal loading of 0.10 mg$_{mass}$ cm^{-2} on non-porous Grafoil substrates.

In-situ optical visualization of hydrogen bubble evolution was added in the revised Supplementary Information to compare dynamic bubble behavior across different catalyst layers under alkaline HER conditions.

(Page 147 in the revised Supplementary Information)

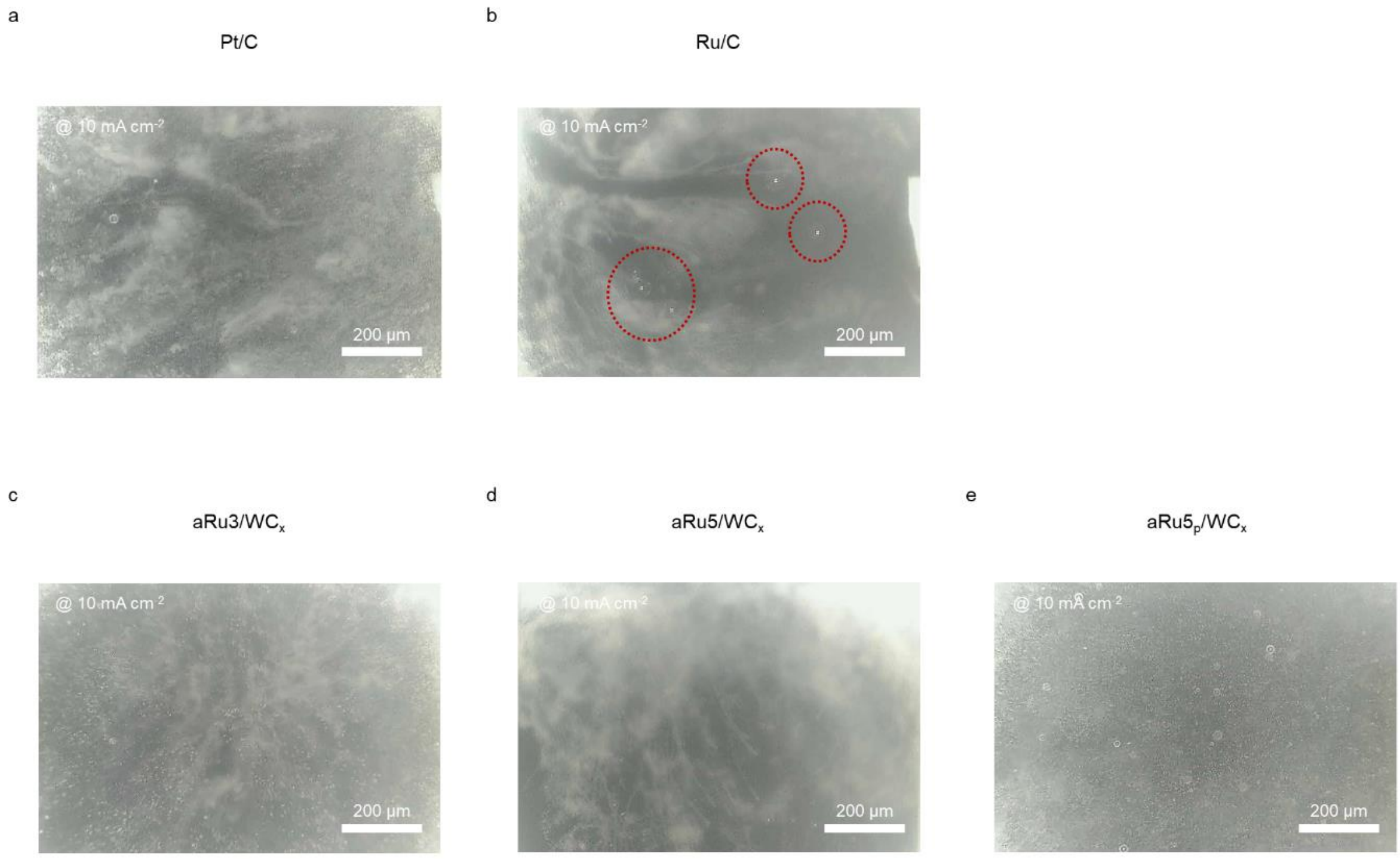

Supplementary Fig. 129 | *In-situ* optical visualization of hydrogen bubble evolution on catalyst layers under alkaline HER conditions. a-e, Optical images of gas bubbles generated on catalyst layers prepared with (**a**) commercial Pt/C, (**b**) commercial Ru/C, (**c**) aRu3/WC_x, (**d**) aRu5/WC_x, and (**e**) $aRu5_p/WC_x$ at a current density of 10 mA cm^{-2}. Red dotted circles indicate localized bubble accumulation observed on the Ru/C catalyst layer.

EIS-based overpotential breakdown analyses were added in the revised Supplementary Information to evaluate the contribution of mass transport limitations during high-current-density AEMWE operation.

(Page 148 in the revised Supplementary Information)

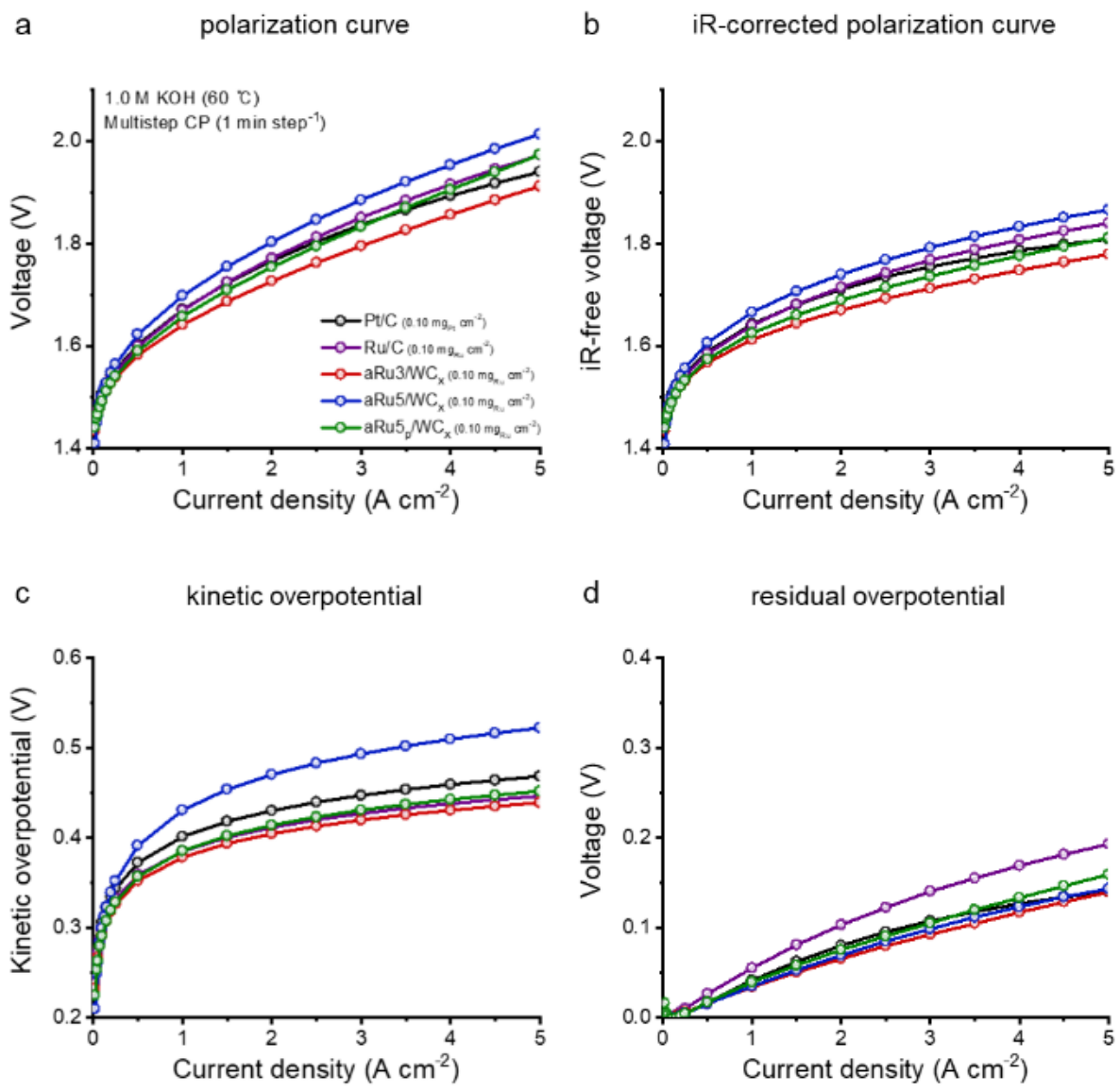

Supplementary Fig. 130 | Overpotential breakdown analysis of AEMWE performance. a, Single-cell polarization curves of AEMWEs using commercial Pt/C, commercial Ru/C, aRu3/WC_x, aRu5/WC_x, and aRu5_p/WC_x cathodes in 1.0 M KOH at 60 ℃ under multistep chronopotentiometric (CP) operation. Corresponding **b,** iR-corrected polarization curves, **c,** kinetic overpotentials, and **d,** residual overpotentials derived from electrochemical impedance spectroscopy (EIS)-based analysis.

Experimental details for the overpotential breakdown analysis and *in-situ* optical visualization of hydrogen bubble evolution were added in the *Experimental* section of the revised Manuscript.

(Page 32 in the *Experimental* section of the revised Manuscript)

Overpotential breakdown

Polarization curves for overpotential breakdown analysis were obtained using multistep chronopotentiometry (CP) with current densities ranging from 0.02 to 5.0 A cm^{-2}. The applied current density was sequentially increased from 0.02 to 0.25 A cm^{-2} (0.02, 0.04, 0.05, 0.075, 0.10, 0.15, 0.20, and 0.25 A cm^{-2}), followed by increments of 0.5 A cm^{-2} up to 5.0 A cm^{-2}. Each current step was maintained for 1 min.

The ohmic resistance at each current density was determined by electrochemical impedance spectroscopy (EIS), and the corresponding iR-corrected polarization curves were obtained by subtracting the ohmic contribution. The kinetic overpotential was extracted from the Tafel region (0.02−0.2 A cm^{-2}), where mass transport effects are negligible. The residual overpotential was calculated by subtracting the ohmic and kinetic contributions from the total cell voltage. The residual overpotential is mainly associated with mass transport limitations, including gas bubble accumulation and reactant/product transport within the catalyst layer.

***In-situ* dynamic bubble behavior of catalyst layers**

Real-time imaging of hydrogen bubble evolution was conducted under alkaline HER conditions using a custom-built transparent electrochemical cell with a three-electrode configuration. The working electrodes were prepared by spray coating catalyst inks onto non-porous Grafoil substrates with a noble metal loading of 0.10 mg_{mass} cm^{-2}. Bubble evolution behavior was monitored under galvanostatic operation at 10 mA cm^{-2} using a digital optical microscope positioned perpendicular to the electrode surface. Time-resolved optical images were recorded to analyze bubble nucleation, growth, detachment, and surface coverage.

6. Is this synthetic strategy universally applicable to other platinum group metals (e.g., Ir, Pt) or non-precious metals (e.g., Ni, Co, Mo)? Furthermore, given the high molecular weight of BCP, how can the uniformity of the Ru ALN structure be ensured during carbonization without local collapse?

Reply

We thank the reviewer for this insightful comment regarding the general applicability of our synthetic strategy and the structural uniformity of Ru ALNs during high-temperature carbonization.

General applicability of the atom-layer neighbor stabilization strategy on WC_x supports

First, we would like to clarify that stabilization of atom-layer neighbor (ALN) structures on WC_x supports requires that the metal species remain surface-confined during high-temperature annealing rather than diffusing into the carbide lattice or forming thermodynamically stable bulk mixed-carbide phases. In the case of many transition metals such as Fe, Co, Ni, and Mo, strong carbide-forming tendencies and relatively high solid solubility in tungsten carbide lattices often promote lattice diffusion, alloying, or mixed-carbide formation during high-temperature treatment (*ACS Appl. Mater. Interfaces*, **2018**, 10, 22094-22101; *Nano Energy*, **2020**, 74, 104850). Therefore, maintaining surface-confined ALN structures under the present annealing conditions is expected to be substantially more challenging for these systems.

In contrast, noble metals such as Ru, Ir, and Pt exhibit substantially lower solubility in tungsten carbide lattices and weaker tendencies toward bulk carbide formation under reductive annealing environments (*Science*, **2016**, 352, 974-978; *Energy Environ. Sci.*, **2025**, 18, 659-673; *Solid State Commun.*, **2005**, 133, 55-59). As a result, noble metal species can remain preferentially confined at the carbide surface, thereby enabling the stabilization of two-dimensional ALN structures through strong metal-support interactions with WC_x.

To examine whether the same stabilization mechanism can be extended to other noble metals, we additionally performed DFT calculations for Pt3, Ir3, Ru3, Pt4, Ir4, and Ru4 clusters on γ-WC supports ($Metal_N$/WC, N: the number of metal atoms). In all cases, the noble metal clusters were preferentially stabilized as two-dimensional surface-confined structures through strong interactions with γ-WC (**Supplementary Fig. 98**). Furthermore, comparison between the cohesive energy of the noble metals (E_{coh}) and the binding energy of the noble metal ALNs on γ-WC (E_{bind}) revealed positive $E_{coh} - E_{bind}$ values for all investigated systems (**Supplementary Fig. 99**), indicating that formation of surface-confined noble metal ALNs on γ-WC is thermodynamically more favorable than metal aggregation.

Nevertheless, extending the present strategy to other noble metals requires identification of suitable precursor-block copolymer systems capable of inducing molecular-level stabilization of the noble metal precursors during the EISA process through favorable molecular interactions. Because such interactions strongly depend on the precursor ligand chemistry, polarity, and compatibility with the block copolymer domains, systematic optimization is required and remains beyond the scope of the present study. As an initial proof-of-concept, we experimentally investigated hydrophobic noble-

metal precursors, including dimethyl(1,5-cyclooctadiene)platinum(II) (HPB Pt) and ((1,5-cyclooctadiene)-η^5-indenyl)iridium(I) (HPB Ir), which possess hydrophobic ligand environments analogous to those of the Ru precursor employed in this work.

The synthetic procedure for aPt3/WC_x and aIr3/WC_x was identical to that used for aRu3/WC_x, except that hydrophobic Pt and Ir precursors, dimethyl(1,5-cyclooctadiene)platinum(II) (HPB Pt) and ((1,5-cyclooctadiene)-η^5-indenyl)iridium(I) (HPB Ir), were employed with the same molar loading as the Ru precursor (see *Catalyst synthesis* in *Methods*).

SEM, N_2 physisorption, and SAXS analyses (**Supplementary Figs. 100a-f, 101a-f**) revealed that both aPt3/WC_x and aIr3/WC_x exhibit structural characteristics similar to those of aRu3/WC_x, including worm-like mesoporous frameworks with short-range mesostructural ordering. These results indicate that the overall mesostructural framework generated through the EISA process can be extended to other noble metal precursor systems. However, distinct differences in noble metal stabilization behavior were observed between Pt and Ir. XRD analysis of aPt3/WC_x (**Supplementary Fig. 100g**) showed discernible crystalline Pt diffraction peaks, whereas no crystalline Ir peaks were observed for aIr3/WC_x (**Supplementary Fig. 101g**). Consistently, HAADF-STEM analyses revealed that both Pt and Ir species were preferentially localized on the WC_x supports (**Supplementary Figs. 102, 103**), indicating strong metal-support interactions (SMSIs) with WC_x. Nevertheless, aPt3/WC_x exhibited partially aggregated bright nanoclusters in the Z-contrast images (red dotted circles), whereas aIr3/WC_x showed highly dispersed Ir species without observable nanocluster formation.

XPS analyses (**Supplementary Figs. 104a-d, 105a-d**) further confirmed that both aPt3/WC_x and aIr3/WC_x possess electronic structures analogous to those of aRu3/WC_x (**Supplementary Fig. 46**), including metallic noble metal species and electronically similar WC_x supports. Because the Pt and Ir L_3-edges overlap with the W L_2-edge, XAS analyses were conducted at the Pt and Ir L_2-edges instead. Both aPt3/WC_x and aIr3/WC_x exhibited white-line intensities (**Supplementary Figs. 104e, 105e**) similar to those of the corresponding metal foils, indicating that the noble metal species retain predominantly metallic character. Notably, FT-EXAFS revealed substantially different local coordination environments for Pt and Ir. In the case of aPt3/WC_x, the FT-EXAFS spectra closely resembled those of Pt foil. Quantitative fitting analysis showed a Pt-Pt coordination number (CN) of 4.10 with a bond length of 2.75 Å, similar to that of Pt foil (2.76 Å). Nevertheless, additional Pt-C and Pt-W coordination components were identified, indicating that Pt nanoclusters remain anchored on WC_x through SMSIs.

In contrast, aIr3/WC_x exhibited a local structure substantially different from that of Ir foil. EXAFS fitting analysis revealed a low Ir-Ir CN of 1.87 with an elongated Ir-Ir bond length of 2.73 Å compared with Ir foil (2.70 Å), together with significant Ir-C coordination (CN = 2.46) and an Ir-W bond length of 2.88 Å. These features are consistent with the structural characteristics observed for Ru ALNs, including low metal-metal CN (< 2.5), substantial metal-C coordination, and enlarged metal-W bond distances relative to metal-metal bonds (**Supplementary Note 8**). These results strongly suggest that Ir can also be stabilized as ALN-like structures on WC_x through the same stabilization mechanism.

The different stabilization behaviors observed for Pt and Ir likely originated from differences in precursor-block copolymer interactions during the EISA process. Although DFT calculations indicate that Pt clusters can also exhibit thermodynamically favorable interactions with WC_x, insufficient molecular-level stabilization of the HPB Pt precursor within the PEO-*b*-PS framework during solvent evaporation and carbonization may promote partial Pt aggregation prior to ALN stabilization. These results suggest that thermodynamically favorable stabilization on WC_x alone is insufficient for ALN formation, and that precursor-directed molecular interactions during EISA play a critical role in suppressing premature aggregation and enabling the formation of surface confined ALN structures.

The alkaline HER activities of the resulting catalysts were additionally evaluated at identical noble metal loadings (42 μg cm^{-2}). aPt3/WC_x exhibited an η_{10} of 26 mV and a Tafel slope of 25.8 mV dec^{-1}, comparable to those of aRu3/WC_x, whereas aIr3/WC_x showed relatively lower HER activity with an η_{10} of 51 mV and a Tafel slope of 97.2 mV dec^{-1}. Collectively, these results demonstrate that the present synthetic strategy can, in principle, be extended to other noble metals, while the successful formation of well-defined ALN structures strongly depends on appropriate precursor-block copolymer interactions during the EISA process.

Structural uniformity of Ru ALNs during carbonization

Regarding the reviewer's concern on the structural uniformity of Ru ALNs during carbonization, we would like to clarify that the present EISA-derived mesostructure provides spatial confinement that suppresses local collapse and premature aggregation during high-temperature annealing.

First, our DFT calculations and molecular interaction analyses indicate that the hydrophobic HPB Ru precursor can be molecularly stabilized within the PS domains through favorable molecular interactions between HPB Ru and the PS moieties (**Fig. 2a**). As a result, the Ru precursors remain highly dispersed within the PS microphases during solvent evaporation and the early stage of thermal treatment. Importantly, during annealing, the PS domains are not simply removed abruptly, but are gradually converted into carbonaceous frameworks that subsequently evolve into mesoporous carbon walls (*Nat. Mater.*, **2008**, 7, 222-228). This progressive carbonization process enables the initially dispersed Ru species to remain spatially confined within the evolving carbon framework, thereby suppressing large-scale migration and aggregation during thermal treatment. Consequently, atomically dispersed Ru species can reorganize into size-confined Ru nanoclusters depending on the Ru loading amount, eventually forming ALN structures after carbide formation.

In addition, strong interactions between Ru species and tungsten oxide/carbide domains further stabilize the Ru clusters during high-temperature annealing and suppress sintering. Previous studies have similarly reported that carbon matrices derived from polymeric templates can effectively inhibit nanoparticle aggregation even under high-temperature intermetallic-forming conditions (*ACS Nano*, **2012**, 6, 6870-6881), while additional interactions with oxide supports further enhance structural stabilization (*RSC Adv.*, **2016**, 6, 88255-88264).

Experimental evidence supporting this stabilization mechanism was obtained from the intermediate sample aRu5/WO_x synthesized at 600 °C, where PS-derived carbonization had progressed but carbide formation was not yet completed. HAADF-STEM analyses revealed that Ru nanoclusters remained selectively localized on WO_x domains without formation of large aggregates

(**Supplementary Fig. 22**), indicating that the combined effects of carbon confinement and Ru-WO_x interactions effectively suppress large-scale structural collapse during annealing. Furthermore, EXAFS fitting analysis showed that the Ru-Ru coordination number remained as low as 2.04 together with substantial Ru-W coordination (CN = 1.03) even after partial carbonization (**Supplementary Table 8**), confirming that the small Ru-cluster structure is preserved throughout the carbonization and carbide-formation process. Moreover, the preservation of the worm-like mesoporous morphology in SEM and TEM analyses after carbonization further supports that large-scale local collapse does not occur during the thermal evolution process (**Supplementary Figs. 16, 17, 22, 23**).

These results collectively indicate that the uniformity of the Ru ALN structures originates from the cooperative stabilization effects of (i) precursor-level molecular dispersion within the PS domains, (ii) spatial confinement provided by the gradually carbonized mesostructure, and (iii) strong Ru-WO_x/WC_x interactions that suppress sintering and large-scale aggregation during carbonization.

Revision

To address the reviewer's comment regarding the general applicability of the present ALN stabilization strategy and the structural uniformity of Ru ALNs during carbonization, additional DFT calculations, experimental validations using Pt- and Ir-based systems, and mechanistic analyses of the carbonization process were performed. Corresponding discussions regarding the thermodynamic stabilization mechanism of noble metal ALNs on WC_x supports, the precursor dependent formation behavior during EISA, and the structural evolution of Ru ALNs during carbonization have been added to the revised Manuscript and Supplementary Information.

Additional discussions regarding the general applicability of the present ALN stabilization strategy to other noble metals, including Pt and Ir systems, together with their corresponding structural analyses and alkaline HER activities, have been added to the revised Manuscript.

(Page 12-13 in the revised Manuscript)

To examine the general applicability of the present ALN stabilization strategy, aPt3/WC_x and aIr3/WC_x were synthesized using hydrophobic Pt and Ir precursors, dimethyl(1,5-cyclooctadiene)platinum(II) (HPB Pt) and ((1,5-cyclooctadiene)-η^5-indenyl)iridium(I) (HPB Ir), respectively, under identical noble metal molar loading conditions to those used for aRu3/WC_x (Supplementary Note 11). Structural analyses revealed that Ir could be stabilized as ALN-like structures on WC_x through the same stabilization mechanism, whereas Pt formed partially aggregated nanoclusters anchored on WC_x (Supplementary Figs. 98-105, Supplementary Table 19). These different stabilization behaviors suggest that thermodynamically favorable noble metal-WC_x interactions alone are insufficient for well-

defined ALN formation, and that precursor-directed MIs during EISA are critical for suppressing premature aggregation and enabling surface confined ALN structures. The alkaline HER activities of the resulting catalysts were additionally evaluated at identical noble-metal loadings (42 μg cm^{-2}). aPt3/WC_x exhibited an η_{10} of 26 mV and a Tafel slope of 25.8 mV dec^{-1}, comparable to those of aRu3/WC_x, whereas aIr3/WC_x showed relatively lower HER activity with η_{10} of 51 mV and a Tafel slope of 97.2 mV dec^{-1} (Supplementary Fig. 106).

The synthetic procedures for aPt3/WC_x and aIr3/WC_x have been added to the revised *Experimental* section.

(Page 23 in the *Experimental* section of the revised Manuscript)

Synthesis of aPt3/WC_x and aIr3/WC_x. The synthetic procedure was identical to that used for aRu3/WC_x, except that dimethyl(1,5-cyclooctadiene)platinum(II) (HPB Pt, 69.4 mg) and ((1,5-cyclooctadiene)-η^5-indenyl)iridium(I) (HPB Ir, 86.4 mg) were employed as noble metal precursors for aPt3/WC_x and aIr3/WC_x, respectively. The noble metal-to-W molar ratio was maintained identical to that used for aRu3/WC_x.

Supplementary Note 11 entitled "General applicability of the ALN stabilization strategy on WC_x supports" has been added to the revised Supplementary Information. This section includes additional DFT calculations for Pt-, Ir-, and Ru-based ALN structures on γ-WC supports, together with structural analyses, XPS/XAS characterizations, and alkaline HER activities of experimentally synthesized aPt3/WC_x and aIr3/WC_x catalysts.

(Page 15-17 in the Supplementary Note 11 of the revised Supplementary Information)

Supplementary Note 11 | General applicability of the ALN stabilization strategy on WC_x supports

The general applicability of the present ALN stabilization strategy was additionally investigated using Pt and Ir precursors on WC_x supports. Stabilization of ALN structures on carbide supports requires that the metal species remain preferentially confined at the carbide surface during high-temperature annealing, rather than diffusing into the carbide lattice or forming thermodynamically stable mixed-carbide phases. In the case of many transition metals such as Fe, Co, Ni, and Mo, strong carbide-forming tendencies and relatively high solid solubility in tungsten carbide lattices can promote lattice diffusion, alloying, or mixed carbide formation during high-temperature treatment, thereby making the stabilization of surface confined ALN structures substantially more challenging under the present synthetic conditions[15,16].

In contrast, noble metals such as Ru, Ir, and Pt exhibit substantially lower solubility in tungsten carbide lattices and weaker tendencies toward bulk carbide formation under reductive annealing environments[1,17,18]. As a result, noble metal species can remain preferentially stabilized at the WC_x surface through SMSIs, thereby enabling the formation of two-dimensional ALN structures. To examine the thermodynamic feasibility of extending the stabilization mechanism to other noble metals, DFT calculations were additionally performed for Pt3, Ir3, Ru3, Pt4, Ir4, and Ru4 clusters on γ-WC supports ($Metal_N$/WC, N: the number of metal atoms) (Supplementary Fig. 98). In all cases, the noble metal clusters were preferentially stabilized as two-dimensional surface confined structures through strong interactions with γ-WC. Furthermore, comparison between the cohesive energies of the noble metals (E_{coh}) and the binding energies of the noble metal ALNs on γ-WC (E_{bind}) revealed positive $E_{coh} - E_{bind}$ values for all investigated systems (Supplementary Fig. 99), indicating that stabilization of surface confined noble metal ALNs on γ-WC is thermodynamically more favorable than metal aggregation.

To experimentally examine the generalizability of the present synthetic strategy, Pt- and Ir-based catalysts were synthesized using the same procedure employed for aRu3/WC_x, except that dimethyl(1,5-cyclooctadiene)platinum(II) (HPB Pt) and ((1,5-cyclooctadiene)-η^5-indenyl)iridium(I) (HPB Ir) were used as hydrophobic noble metal precursors with identical molar loading conditions. SEM, N_2 physisorption, and SAXS analyses revealed that both aPt3/WC_x and aIr3/WC_x exhibit structural characteristics analogous to those of aRu3/WC_x, including worm-like mesoporous frameworks with short-range mesostructural ordering (Supplementary Figs. 100a-f, 101a-f). These results indicate that the mesostructural framework generated through EISA process can be extended to other noble metal precursor systems.

However, distinct differences in noble metal stabilization behavior were observed between Pt and Ir. XRD analysis of aPt3/WC_x showed discernible crystalline Pt diffraction peaks, whereas no crystalline Ir peaks were observed for aIr3/WC_x (Supplementary Figs. 100g, 101g). Consistently, HAADF-STEM analyses revealed that both Pt and Ir species were preferentially localized on the WC_x supports, indicating strong SMSIs with WC_x (Supplementary Figs. 102, 103). Nevertheless, aPt3/WC_x exhibited partially aggregated bright nanoclusters in the Z-contrast images, whereas aIr3/WC_x showed highly dispersed Ir species without observable nanocluster formation.

XPS analyses further confirmed that both aPt3/WC_x and aIr3/WC_x possess electronic structures analogous to those of aRu3/WC_x, including metallic noble metal species and electronically similar WC_x supports (Supplementary Figs. 104, 105). Because the Pt and Ir L_3-edges overlap with the W L_2-edge, XAS analyses were conducted at the Pt and Ir L_2-edges instead. Both aPt3/WC_x and aIr3/WC_x exhibited white-line intensities similar to those of the corresponding metal foils, indicating that the noble-metal species retain predominantly metallic character.

Notably, FT-EXAFS analyses revealed substantially different local coordination environments for Pt and Ir. In the case of aPt3/WC_x, the FT-EXAFS spectra closely resembled those of Pt foil. Quantitative fitting analysis revealed a Pt-Pt CN of 4.10 with a bond length of 2.75 Å, similar to that of Pt foil (2.76 Å). Nevertheless, additional Pt-C and Pt-W coordination components were identified, indicating that Pt nanoclusters remain anchored on WC_x through SMSIs. In contrast, aIr3/WC_x exhibited a local structure substantially different from that of Ir foil. EXAFS fitting analysis revealed a low Ir-Ir CN of 1.87 with an elongated Ir-Ir bond length of 2.73 Å compared with Ir foil (2.70 Å), together with substantial Ir-C coordination (CN = 2.46) and an Ir-W bond length of 2.88 Å. These

structural characteristics are highly consistent with those observed for Ru ALNs, including low metal-metal CNs (< 2.5), substantial metal-C coordination, and enlarged metal-W bond distances relative to metal-metal bonds (Supplementary Note 8). These results strongly suggest that Ir can also be stabilized as ALN-like structures on WC_x through the same stabilization mechanism.

The different stabilization behaviors observed for Pt and Ir likely originate from differences in precursor-block copolymer interactions during the EISA process. Although DFT calculations indicate that Pt clusters can also exhibit thermodynamically favorable interactions with WC_x, insufficient molecular-level stabilization of the HPB Pt precursor within the PEO-*b*-PS framework during solvent evaporation and annealing may promote partial Pt aggregation prior to ALN stabilization. These results suggest that thermodynamically favorable stabilization on WC_x alone is insufficient for ALN formation, and that precursor-directed molecular interactions during EISA play a critical role in suppressing premature aggregation and enabling the formation of surface confined ALN structures.

The alkaline HER activities of the resulting catalysts were additionally evaluated at identical noble-metal loadings (42 μg cm^{-2}). aPt3/WC_x exhibited an η_{10} of 26 mV and a Tafel slope of 25.8 mV dec^{-1}, comparable to those of aRu3/WC_x, whereas aIr3/WC_x showed relatively lower HER activity with η_{10} of 51 mV and a Tafel slope of 97.2 mV dec^{-1}. Collectively, these results demonstrate that the present synthetic strategy can, in principle, be extended to other noble metals, while the successful formation of well-defined ALN structures strongly depends on molecular-level precursor stabilization within the block copolymer framework during the EISA process.

Additional supplementary figures and tables related to the general applicability of the ALN stabilization strategy, including DFT-optimized structures, stabilization energy analyses, structural characterizations, STEM/XAS analyses, and alkaline HER performances of aPt3/WC_x and aIr3/WC_x have been added to the revised Supplementary Information.

(Page 116-124, 172 in the revised Supplementary Information)

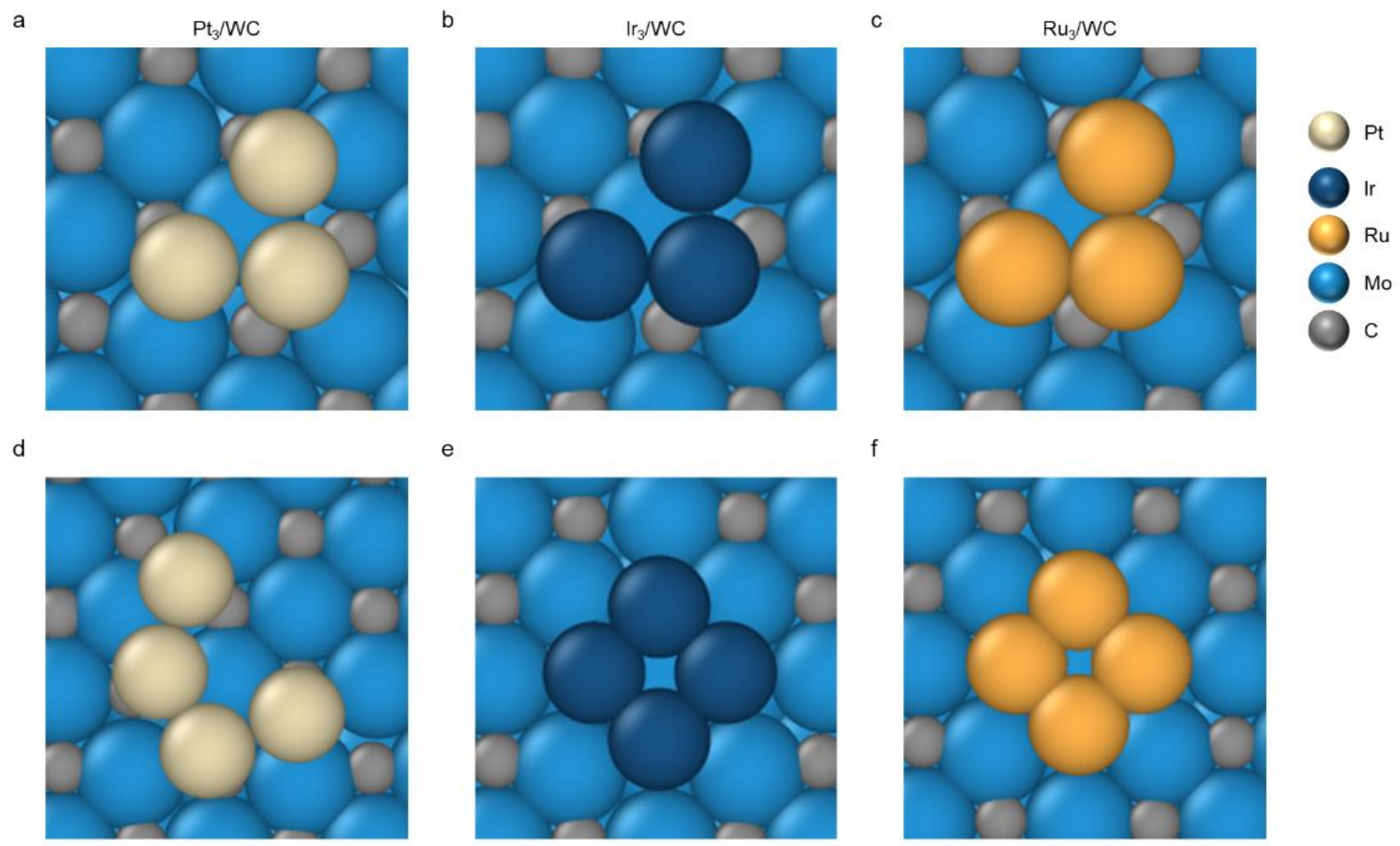

Supplementary Fig. 98 | DFT-optimized structures of noble metal ALNs on γ-WC support. a-f, Optimized atomic configurations of noble metal ALNs supported on γ-WC, including (a) Pt_3/WC, (**b**) Ir_3/WC, (**c**) Ru_3/WC, (**d**) Pt_4/WC, (**e**) Ir_4/WC, and (**f**) Ru_4/WC, which were used to calculate the cohesive energy (E_{coh}) and binding energy (E_{bind}) of the noble metal ALNs on γ-WC

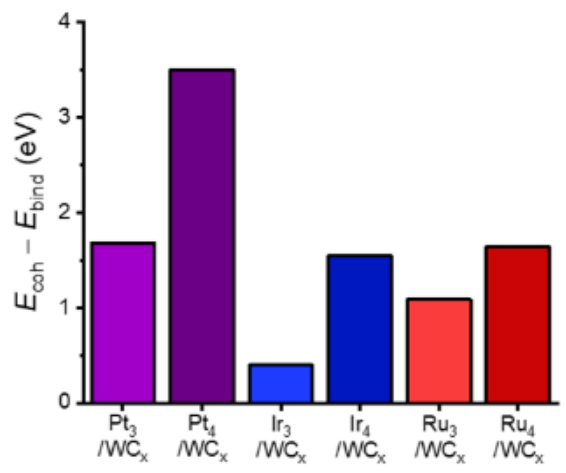

Supplementary Fig. 99 | Relative stabilization energies of noble metal ALNs on γ-WC support. Comparison between the cohesive energy (E_{coh}) of noble metals and the binding energy (E_{bind}) of noble metal ALNs on γ-WC support.

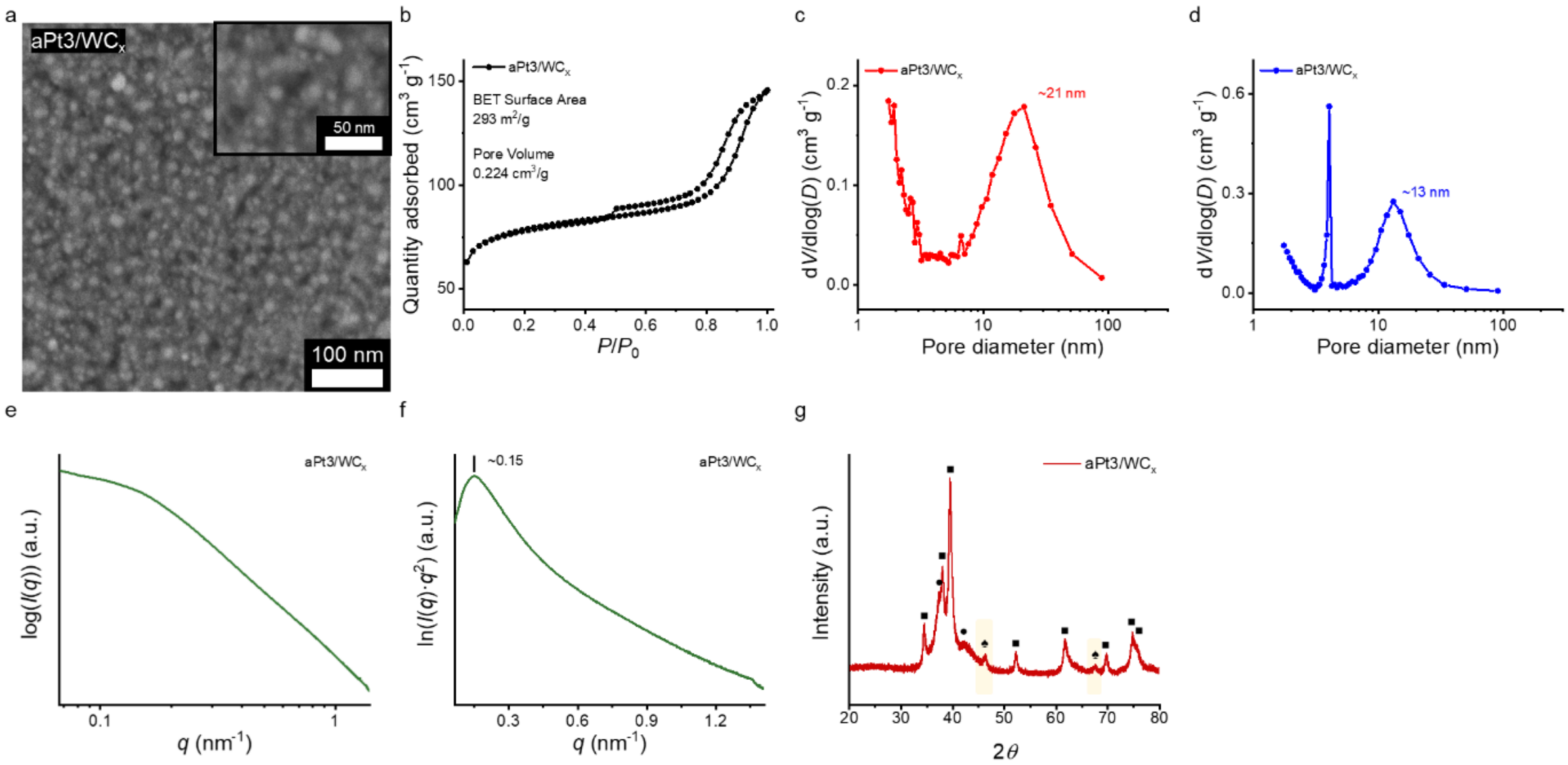

Supplementary Fig. 100 | Structural characterization of aPt3/WC$_x$. a, SEM image of aPt3/WC$_x$. Insets show the corresponding higher-magnification image. **b,** N_2 adsorption-desorption isotherms, **c,** pore size distribution, and **d**, window size distribution derived from N_2 physisorption analysis. **e,** SAXS scattering profile and **f,** corresponding Kratky plot. **g,** XRD pattern of aPt3/WC$_x$.

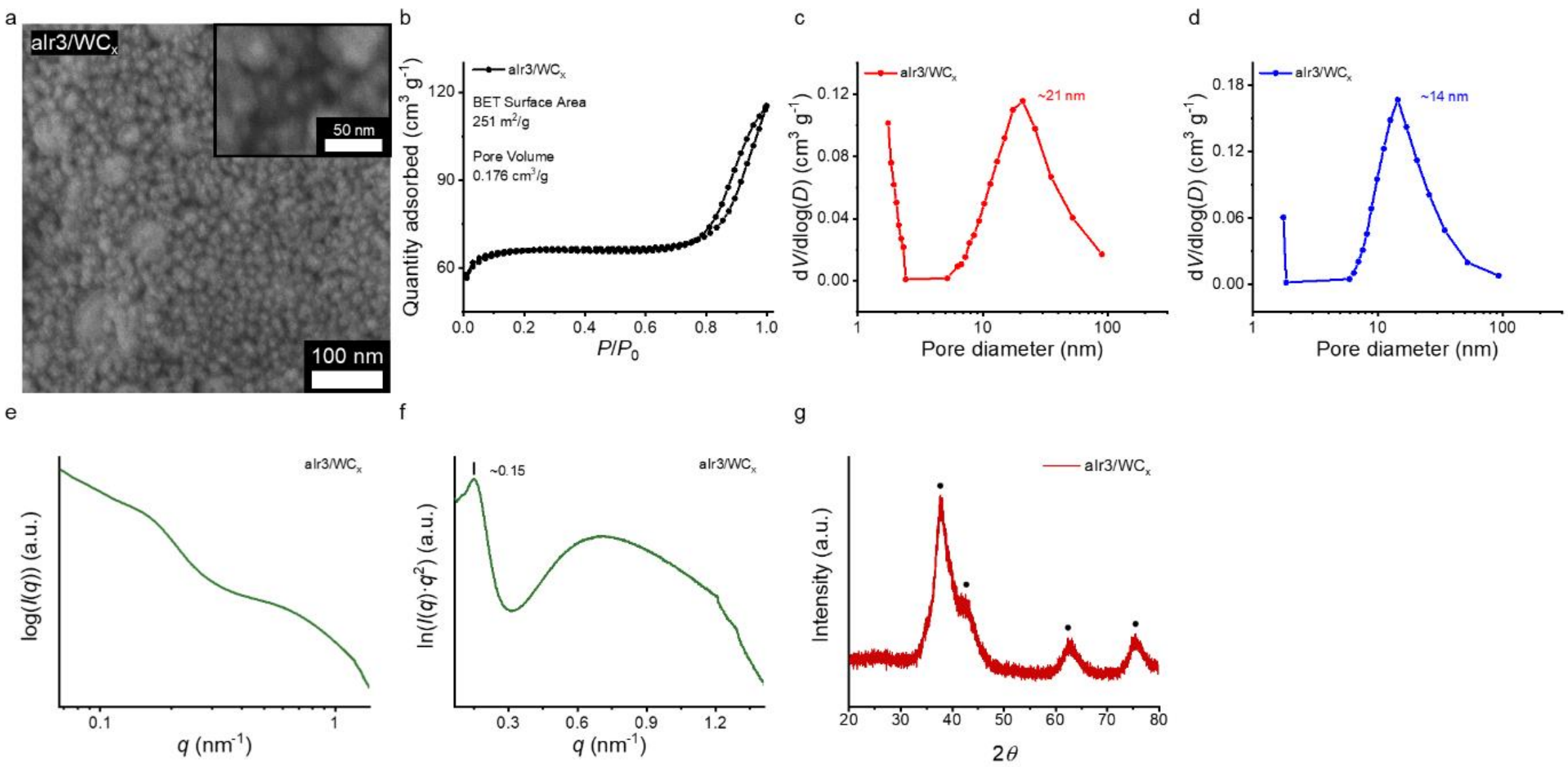

Supplementary Fig. 101 | Structural characterization of aIr3/WC$_x$. a, SEM image of aIr3/WC$_x$. Insets show the corresponding higher-magnification image. **b,** N_2 adsorption-desorption isotherms, **c,** pore size distribution, and **d**, window size distribution derived from N_2 physisorption analysis. **e,** SAXS scattering profile and **f,** corresponding Kratky plot. **g,** XRD pattern of aIr3/WC$_x$.

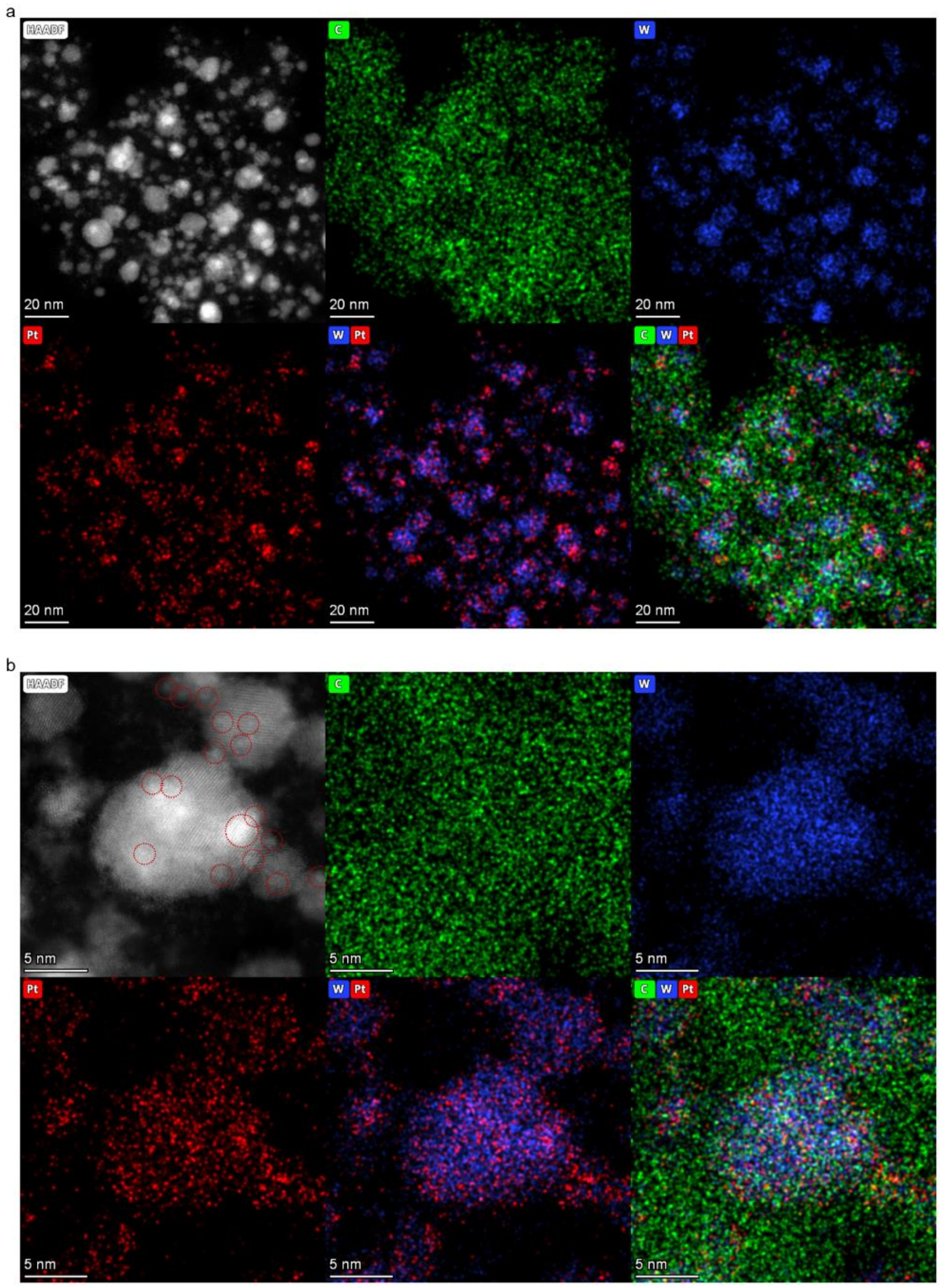

Supplementary Fig. 102 | STEM analysis of aPt3/WC$_x$. a, HAADF-STEM image and the corresponding EDS elemental mappings for C (green), W (blue), Pt (red), along with the overlaid composite map. **b,** Higher-magnification HAADF-STEM image and corresponding EDS mappings for the same elements, their composite overlay.

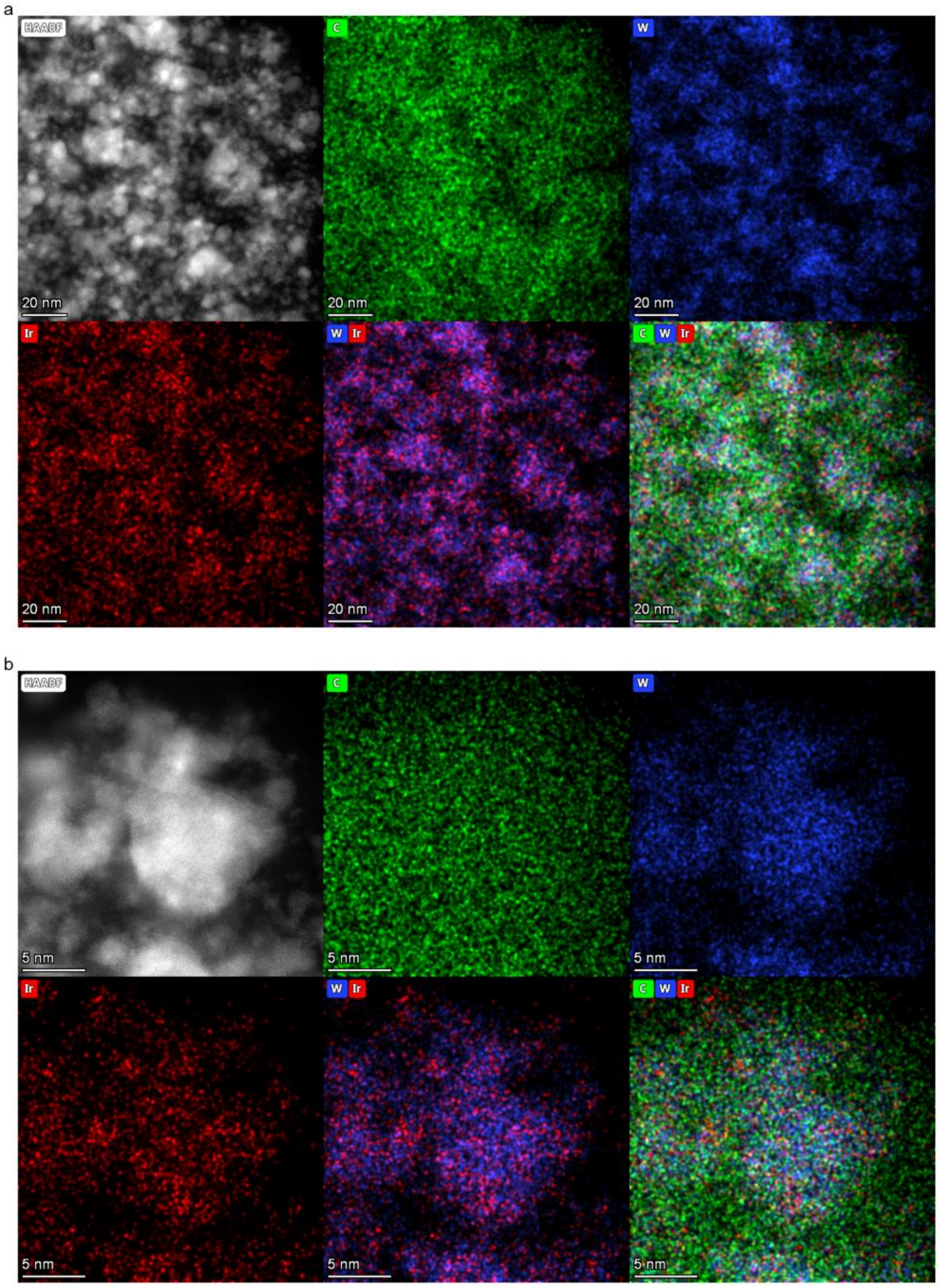

Supplementary Fig. 103 | STEM analysis of aIr3/WC$_x$. a, HAADF-STEM image and the corresponding EDS elemental mappings for C (green), W (blue), Ir (red), along with the overlaid composite map. **b,** Higher-magnification HAADF-STEM image and corresponding EDS mappings for the same elements, their composite overlay.

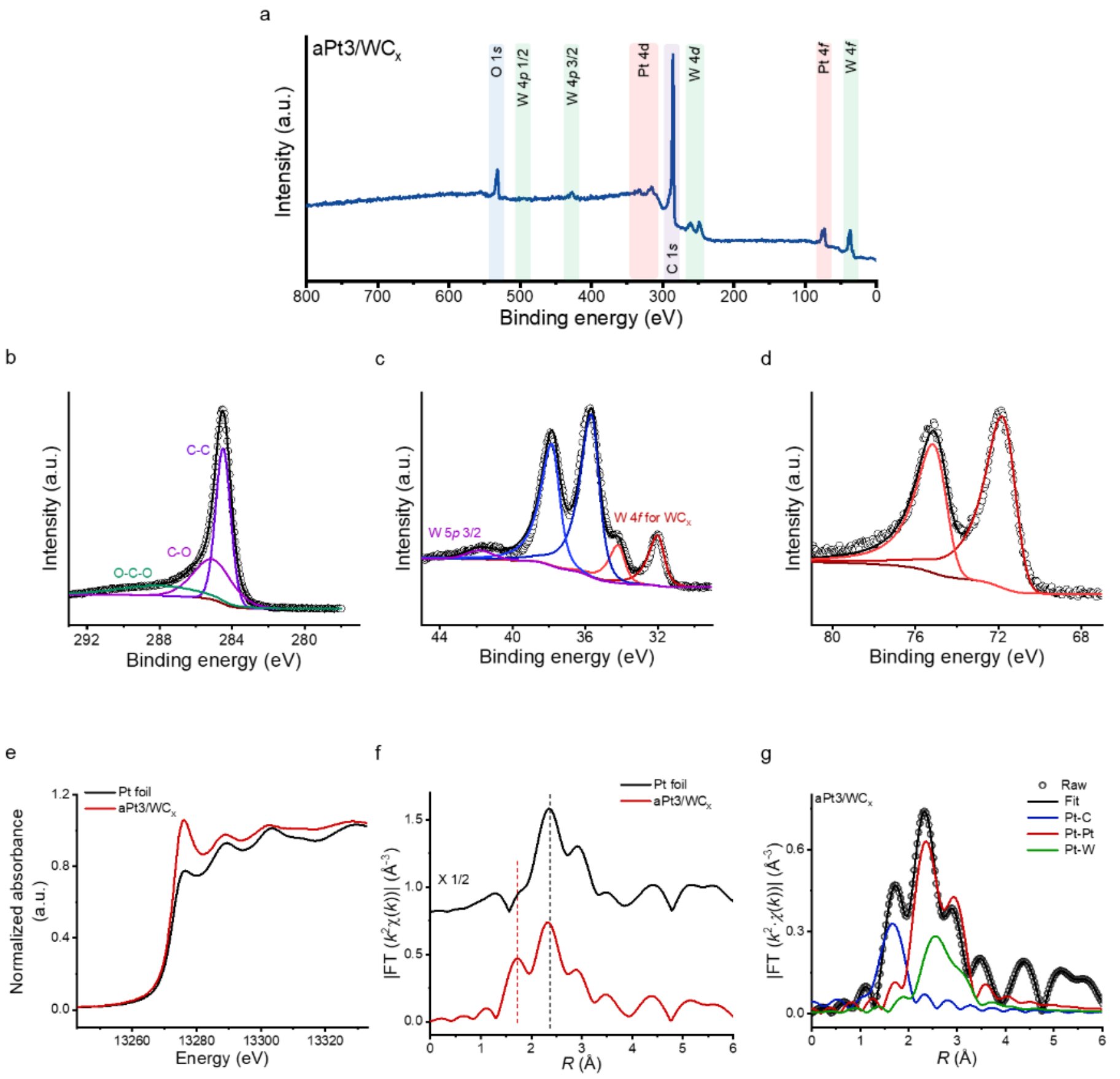

Supplementary Fig. 104 | Electronic and geometric structure analyses of aPt3/WC_x. a-d, XPS analyses of aPt3/WC_x. (**a**) Survey XPS spectrum. (**b**) C 1s spectrum (violet: C-C, purple: C-O, green: O-C-O), (**c**) W 4f spectrum (red: WC_x, blue: surface WO_x attributed to naturally formed oxides, purple: W 5p 3/2), and (**d**) Pt 4f spectrum. **e-g,** XAS analyses of aPt3/WC_x at the Pt L_2-edge. (**e**) XANES spectra, (**f**) k^2-weighted FT-EXAFS spectra, and (**g**) corresponding FT-EXAFS fitting results.

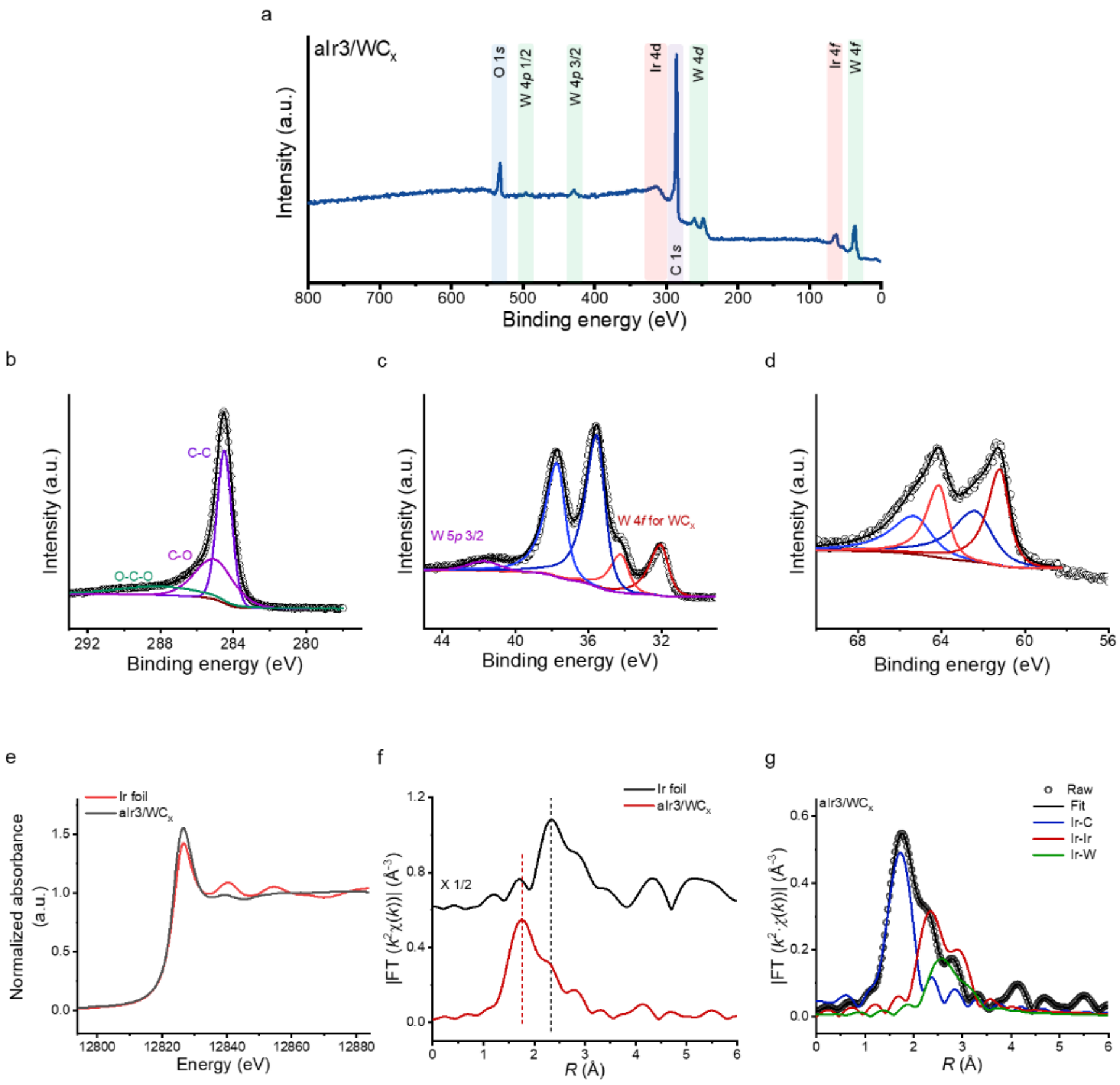

Supplementary Fig. 105 | Electronic and geometric structure analyses of aIr3/WC$_x$. a-d, XPS analyses of aIr3/WC$_x$. (**a**) Survey XPS spectrum. (**b**) C 1s spectrum (violet: C-C, purple: C-O, green: O-C-O), (**c**) W 4f spectrum (red: WC$_x$, blue: surface WO$_x$ attributed to naturally formed oxides, purple: W 5p 3/2), and (**d**) Ir 4f spectrum. **e-g,** XAS analyses of aIr3/WC$_x$ at the Ir L$_2$-edge. (**e**) XANES spectra, (**f**) k^2-weighted FT-EXAFS spectra, and (**g**) corresponding FT-EXAFS fitting results.

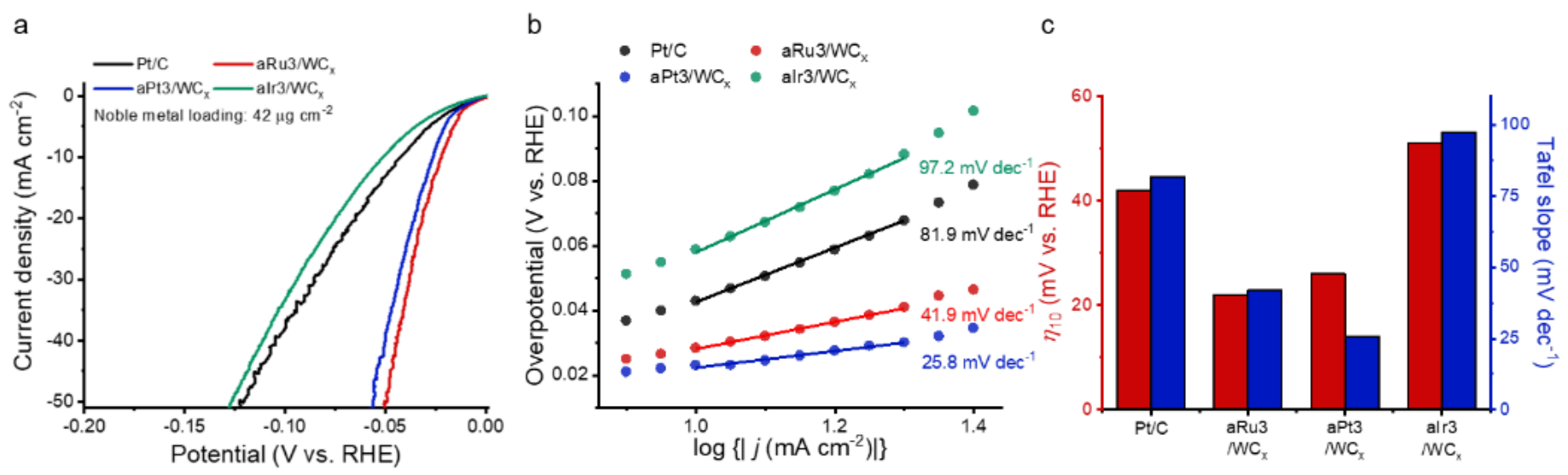

Supplementary Fig. 106 | Alkaline HER activity for aPt3/WC$_x$ and aIr3/WC$_x$. a, LSV curves, **b,** corresponding Tafel plot, and **c,** corresponding η_{10} and Tafel slope.

Supplementary Table 19 | Structural parameters extracted from EXAFS fitting analyses. Structural parameters extracted from the Ru K-edge, Pt L_2-edge, Ir L_2-edge EXAFS fitting results. CN: coordination number, R: interatomic distance, and σ^2: Debye-Waller factor. The goodness of fit is represented by the R-factor. (weighting: k^2)

Sample	Shell	CN	R (Å)	σ^2 (10^{-3} Å^2)	R-factor
Ru	Ru-Ru	6*	2.65 ± 0.01	2.56	0.007
	Ru-Ru	6*	2.70 ± 0.01		
RuO_2	Ru-O	2*	1.94 ± 0.01	0.73	0.014
	Ru-O	4*	1.98 ± 0.01		
Ru/WC_x_R	Ru-O	2.06 ± 0.33	1.97 ± 0.01	5.69	0.012
	Ru-Ru	4.51 ± 0.41	2.66 ± 0.01	6.93	
Ru/C_R	Ru-O	2.79 ± 0.37	1.97 ± 0.01	6.95	0.014
	Ru-Ru	3.70 ± 0.41	2.67 ± 0.01	6.45	
RuNP/WC_x	Ru-O	2.93 ± 0.19	1.94 ± 0.01	5.87	0.003
	Ru-Cl	0.86 ± 0.14	2.30 ± 0.01		
	Ru-Ru	2.27 ± 0.23	2.66 ± 0.01	5.74	
RuNP/C	Ru-O	4.63 ± 0.19	1.97 ± 0.00	17.23	0.001
	Ru-Cl	1.45 ± 0.14	2.27 ± 0.01		
	Ru-Ru	2.35 ± 0.11	2.66 ± 0.00	5.00	
aRu/NC	Ru-N	1.53 ± 0.06	2.01 ± 0.00	1.75	0.001
	Ru-Cl	1.06 ± 0.03	2.33 ± 0.00		
	Ru-Ru	1.92 ± 0.08	2.61 ± 0.00	8.14	
aPt3/WC_x**	Pt-C	1.59 ± 0.30	2.08 ± 0.02	2.16	0.009
	Pt-Pt	4.10 ± 0.34	2.75 ± 0.01	2.07	
	Pt-W	1.93 ± 0.37	2.88 ± 0.01		
aIr3/WC_x**	Ir-C	2.46 ± 0.08	2.11 ± 0.00	2.69	0.001
	Ir-Ir	1.87 ± 0.09	2.73 ± 0.00	1.49	
	Ir-W	1.06 ± 0.09	2.88 ± 0.01		

Structural parameters extracted from the EXAFS fitting of reference metal foil. CN is the coordination number; R is the interatomic distance; σ^2 is the Debye-Waller factor (a measure of the static and thermal disorder in absorber-scatterer distance); ΔE_0 is edge energy shift (the zero kinetic energy difference between experiment and theoretical model); R-factor value is related with the goodness of the fitting.
* Value was assigned in curving fitting based on standard structure
** For Pt and Ir, EXAFS fitting was conducted at the L_2-edge to avoid spectral overlap between the L_3-edge and the W L_2-edge. S_0^2 value was obtained from reference foil measurements at the corresponding L_2-edge
R_{bkg} was set to 1.0 in background removal to remove the noise oscillation below 1 Å in R-space
S_0^2 value (0.744) was calculated from the fitting of metal foil with standard metal structure

Supplementary Note 3 entitled "Structural evolution and stabilization mechanism of Ru ALNs during carbonization" has been added to clarify how the EISA-derived mesostructure suppresses local collapse and premature aggregation during high-temperature annealing through precursor-level molecular stabilization, carbon-confinement effects and Ru-WO_x/WC_x interactions. Additional discussions regarding the structural evolution mechanism of Ru ALNs during carbonization, including intermediate state analyses of aRu5/WO_x and EXAFS evidence for preservation of small Ru-cluster structures during thermal evolution, have been added to the revised Supplementary Information.

(Page 3-4 in the Supplementary Note 3 of the revised Supplementary Information)

Supplementary Note 3 | Structural evolution and stabilization mechanism of Ru ALNs during carbonization

The structural evolution and stabilization mechanism of Ru ALNs during carbonization were additionally investigated to clarify how the present EISA-derived mesostructure suppresses local collapse and premature aggregation during high-temperature annealing. DFT calculations and molecular interaction analyses indicate that the hydrophobic HPB Ru precursor can be molecularly stabilized within the PS domains through favorable molecular interactions between HPB Ru and the PS moieties (Fig. 2a). As a result, the Ru precursors remain highly dispersed within the PS microphases during solvent evaporation and the early stage of thermal treatment.

Importantly, during annealing, the PS domains are not removed abruptly but are gradually converted into carbonaceous frameworks that subsequently evolve into mesoporous carbon walls (*Nat. Mater.*, **2008**, 7, 222-228). This progressive carbonization process enables the initially dispersed Ru species to remain spatially confined within the evolving carbon framework, thereby suppressing large-scale migration and aggregation during thermal treatment. Consequently, atomically dispersed Ru species can reorganize into size-confined Ru nanoclusters depending on the Ru loading amount, eventually forming ALN structures after carbide formation.

In addition, strong interactions between Ru species and tungsten oxide/carbide domains further stabilize the Ru clusters during high-temperature annealing and suppress sintering. Previous studies have similarly reported that carbon matrices derived from polymeric templates can effectively inhibit nanoparticle aggregation even under high-temperature intermetallic-forming conditions (*ACS Nano*, **2012**, 6, 6870-6881), while additional interactions with oxide supports further enhance structural stabilization (*RSC Adv.*, **2016**, 6, 88255-88264).

Experimental evidence supporting this stabilization mechanism was obtained from the intermediate sample aRu5/WO_x synthesized at 600 °C, where PS-derived carbonization had progressed but carbide formation was not yet completed. High-angle annular dark-field scanning transmission electron microscopy (HAADF-STEM) analyses revealed that Ru nanoclusters remained selectively localized on WO_x domains without formation of large aggregates (Supplementary Fig. 22), indicating that the combined effects of carbon confinement and Ru-WO_x interactions effectively suppress large-scale structural collapse during annealing. Furthermore, Fourier transforms of extended X-ray absorption fine structure (FT-EXAFS) fitting analysis showed

that the Ru−Ru coordination number (CN) remained as low as 2.04 together with substantial Ru−W coordination (CN = 1.03) even after partial carbonization (Supplementary Table 8), confirming that the small Ru-cluster structure is preserved throughout the carbonization and carbide-formation process. Moreover, the preservation of the worm-like mesoporous morphology in scanning electron microscopy (SEM) and STEM analyses after carbonization further supports that large-scale local collapse does not occur during the thermal evolution process (Supplementary Figs. 16, 17, 22, 23).

These results collectively indicate that the uniformity of the Ru ALN structures originates from the cooperative stabilization effects of (i) precursor-level molecular dispersion within the PS domains, (ii) spatial confinement provided by the gradually carbonized mesostructure, and (iii) strong Ru−WO_x/WC_x interactions that suppress sintering and large-scale aggregation during carbonization.

7. How does the carrier tolerate high potentials in an alkaline environment? Could oxidation occur, leading to Ru ALN detachment? Please supplement with ICP-MS analysis data of the electrolyte following accelerated degradation testing (ADT) to confirm the dissolution loss rates of Ru and W elements under simulated real-world conditions (including start-stop cycles).

Reply

We thank the reviewer for this insightful comment regarding the electrochemical stability of the WC_x support and the possible detachment of Ru ALNs under alkaline AEMWE operation.

First, we would like to clarify that aRu3/WC_x functions as a HER cathode catalyst under AEMWE operating conditions. In contrast to AEMFC systems, where local fuel starvation during start−stop events can induce reverse-voltage conditions and highly oxidative potentials that accelerate carbon corrosion and catalyst degradation (*J. Power Sources*, **2021**, 488, 229434), AEMWE cathodes generally operate under HER-relevant cathodic or near-equilibrium potentials even during intermittent operation. Therefore, severe anodic potential excursions capable of strongly oxidizing the WC_x support are significantly less likely to occur during practical AEMWE operation.

In addition, previous studies on transition-metal carbides have reported that WC exhibits relatively high electrochemical stability under HER-relevant reductive potentials, particularly in alkaline environments, where WC is reported to maintain substantial stability within the HER-relevant cathodic potential region. In particular, the stability-map and electrochemical analyses reported by Jingguang G. Chen group (*J. Power Sources*, **2012**, 202, 11-17) demonstrated that WC can remain substantially stable under alkaline HER conditions and serve as a robust catalyst support over a broad cathodic potential range.

Nevertheless, to directly address the reviewer's concern, we additionally performed intermittent start-stop cycling tests by alternating between 1.0 A cm^{-2} and open-circuit voltage (OCV) every 1 h for a total operation time of 100 h to simulate practical intermittent AEMWE operation (**Supplementary Fig. 127**). aRu3/WC_x exhibited substantially smaller voltage degradation than commercial Pt/C even under repeated start-stop operation, demonstrating the excellent operational robustness of the Ru ALN/WC_x system.

Following the intermittent operation test, distilled water in the cathode-side trap bottle (1 L) was recirculated through the cathode compartment for 1 h to collect dissolved metal species remaining in the system. The collected solution was subsequently analyzed by ICP-MS to quantify dissolved Ru, W, and Pt species (**Supplementary Table 22**). No detectable Ru dissolution was observed from aRu3/WC_x, whereas measurable Pt dissolution was detected from the commercial Pt/C cathode. Although trace W dissolution was detected from aRu3/WC_x, the dissolved amount was extremely small, indicating that the WC_x support remained largely stable during intermittent AEMWE operation. Importantly, the absence of detectable Ru dissolution strongly suggests that the Ru ALNs remain stably anchored on the WC_x support without significant detachment or electrochemical

corrosion during intermittent operation. This result is also fully consistent with the exceptionally high S-number of aRu3/WC_x obtained during continuous 100 h AEMWE operation in the Manuscript (**Fig. 6h**), where aRu3/WC_x exhibited an S-number approximately five and nine times higher than those of Pt/C and Ru/C respectively, indicating substantially suppressed noble metal dissolution under practical operating conditions.

We additionally note that the total dissolved metal concentration measured during the intermittent start-stop test was lower than that observed during the 100 h constant-current durability operation. For example, the dissolved Pt amount under intermittent operation (9.6 μg kg^{-1}) was substantially lower than that measured during continuous chronopotentiometric operation (72.8 μg kg^{-1}). Although the exact origin of this behavior remains unclear, this result further suggests that intermittent AEMWE operation does not induce unusually severe cathode degradation associated with reverse-voltage-type conditions, in contrast to fuel-cell systems. Furthermore, these results suggest that the Ru ALN/WC_x system is substantially less susceptible to intermittent-operation-induced degradation mechanisms commonly observed in non-noble-metal HER catalysts, such as oxidation/reconstruction and metal dissolution during repeated start-stop cycling.

Taken together, these results indicate that the WC_x support maintains sufficient electrochemical stability under practical alkaline HER conditions and that the Ru ALNs remain strongly stabilized on the WC_x support even during intermittent AEMWE operation.

Revision

Additional analyses regarding the electrochemical stability of the WC_x support and the operational robustness of the Ru ALN/WC_x system under practical intermittent AEMWE conditions were added in the revised Manuscript and Supplementary Information. To directly address the reviewer's concern regarding possible support oxidation, Ru ALN detachment, and metal dissolution during start-stop operation, intermittent AEMWE durability tests and ICP-MS analyses of dissolved metal species were additionally performed.

Descriptions regarding the intermittent operational stability of the Ru ALN/WC_x system and dissolved metal analysis after start-stop operation were added in the revised Manuscript.

(Page 18-19 in the revised Manuscript)

To further evaluate the operational stability of the Ru ALNs under practical intermittent AEMWE conditions, intermittent start-stop cycling tests were additionally performed by alternating between 1.0 A cm^{-2} and open-circuit voltage (OCV) every 1 h for a total operation time of 100 h (Supplementary Fig. 127). aRu3/WC_x exhibited substantially smaller voltage degradation than commercial Pt/C even under repeated start-stop operation, indicating the high operational stability of the Ru ALN/WC_x system. In addition, inductively coupled plasma mass spectrometry (ICP-MS) analysis of dissolved metal species after the intermittent operation revealed no detectable Ru dissolution from aRu3/WC_x, whereas measurable Pt dissolution was observed from commercial Pt/C (Supplementary Table 22). Although trace W dissolution was detected from aRu3/WC_x, the dissolved amount was extremely small, indicating that the WC_x support remained largely stable during intermittent operation. These results further suggest that the Ru ALNs remain stably anchored on the WC_x support without significant detachment under practical alkaline AEMWE operating conditions.

Voltage profiles during intermittent start-stop AEMWE operation were added in the revised Supplementary Information.

(Page 145 in the revised Supplementary Information)

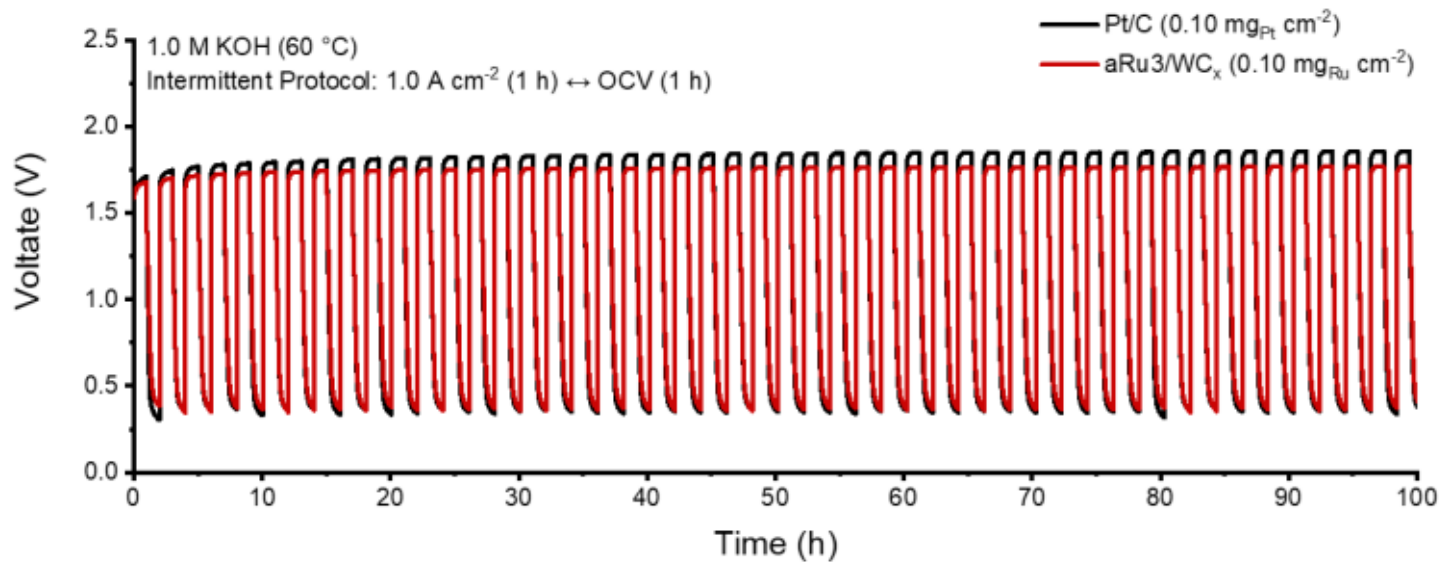

Supplementary Fig. 127 | Intermittent AEMWE operation stability test. Voltage profiles of AEMWEs employing commercial Pt/C and aRu3/WC_x cathodes during intermittent operation by alternating between galvanostatic operation at 1.0 A cm^{-2} and OCV every 1 h for a total operation time of 100 h in 1.0 M KOH at 60 °C.

ICP-MS analysis of dissolved metal species after intermittent operation was added in the revised Supplementary Information.

(Page 177 in the revised Supplementary Information)

Supplementary Table 22 | Dissolved metal concentrations after intermittent AEMWE operation. ICP-MS analysis of dissolved metal species in the catholyte collected after intermittent start-stop cycling tests. N.D indicates concentrations below the detection limit (5 μg kg^{-1})

Element	aRu3/WC_x	commercial Pt/C
W	31.1 μg kg^{-1}	N/D
Pt	N/D	9.6 μg kg^{-1}
Ru	N/D	N/D

Experimental details regarding intermittent start-stop cycling tests and ICP-MS analysis were added in the *Experimental* section of the revised Manuscript.

(Page 31 in the *Experimental* section of the revised Manuscript)

To evaluate operational stability under practical intermittent AEMWE conditions, start-stop cycling tests were additionally performed by alternating between galvanostatic operation at 1.0 A cm^{-2} and OCV every 1h for a total operation time of 100 h. After the intermittent operation test, distilled water in the cathode-side trap bottle (1 L) was recirculated through the cathode compartment for 1 h to collect dissolved metal species remaining in the system. The collected solution was subsequently analyzed using ICP-MS to quantify dissolved Ru, W, and Pt species.

8. Some figures (e.g., Fig.2) lack sufficient "white space" between sub-panels, leading to a cluttered appearance. I recommend re-aligning these panels to a standard grid to improve the reader's navigation through the structural characterization data. In Fig.4, the in-situ Raman and ATR-SEIRAS waterfall plots are currently overcrowded. Using a consistent color gradient for potential increments and providing difference spectra would significantly enhance the clarity of the mechanistic insights. The figures appear cluttered and lack the professional polish required for clear communication of complex data.

Reply

We appreciate the reviewer's constructive comments regarding the figure organization and readability. To improve visual clarity and reduce the cluttered appearance, the figures were substantially reorganized throughout the revised Manuscript. In particular, the original Figure 1 was divided into revised Figures 1 and 2 to improve figure navigation and presentation clarity. The panel layouts were re-aligned with increased spacing and clearer visual hierarchy to facilitate interpretation of the structural characterization data.

In addition, the original Figure 2 was reorganized as revised Figure 3 with improved panel arrangement and readability of the XAS analyses. The WT-EXAFS plots were simplified by removing contour lines and reorganizing the panel layout to provide clearer visualization of the spectral features.

The *in-situ* Raman and ATR-SEIRAS figures were also revised using improved layout organization and color presentation to enhance the clarity of the mechanistic analyses. Following the reviewer's suggestion, consistent color gradients corresponding to the applied potentials were adopted within the *in-situ* XAS panels (**Fig. 5a-d**) and within the *in-situ* Raman and ATR-SEIRAS panels (**Fig. 5e-h**) to improve visual consistency and readability. In addition, a difference contour map derived from the *in-situ* Raman spectra was added to more clearly visualize the potential-dependent spectral evolution. These revisions further improved the visualization of the dynamic interfacial changes during electrochemical operation.

Revision

The figures throughout the revised Manuscript were substantially reorganized to improve readability, white space, panel alignment, and overall presentation clarity.

The original Figure 1 was divided into revised Figures 1 and 2 with reorganized layouts and clearer visual hierarchy for improved navigation of the conceptual and structural characterization data.

(Page 4 in the revised Manuscript)

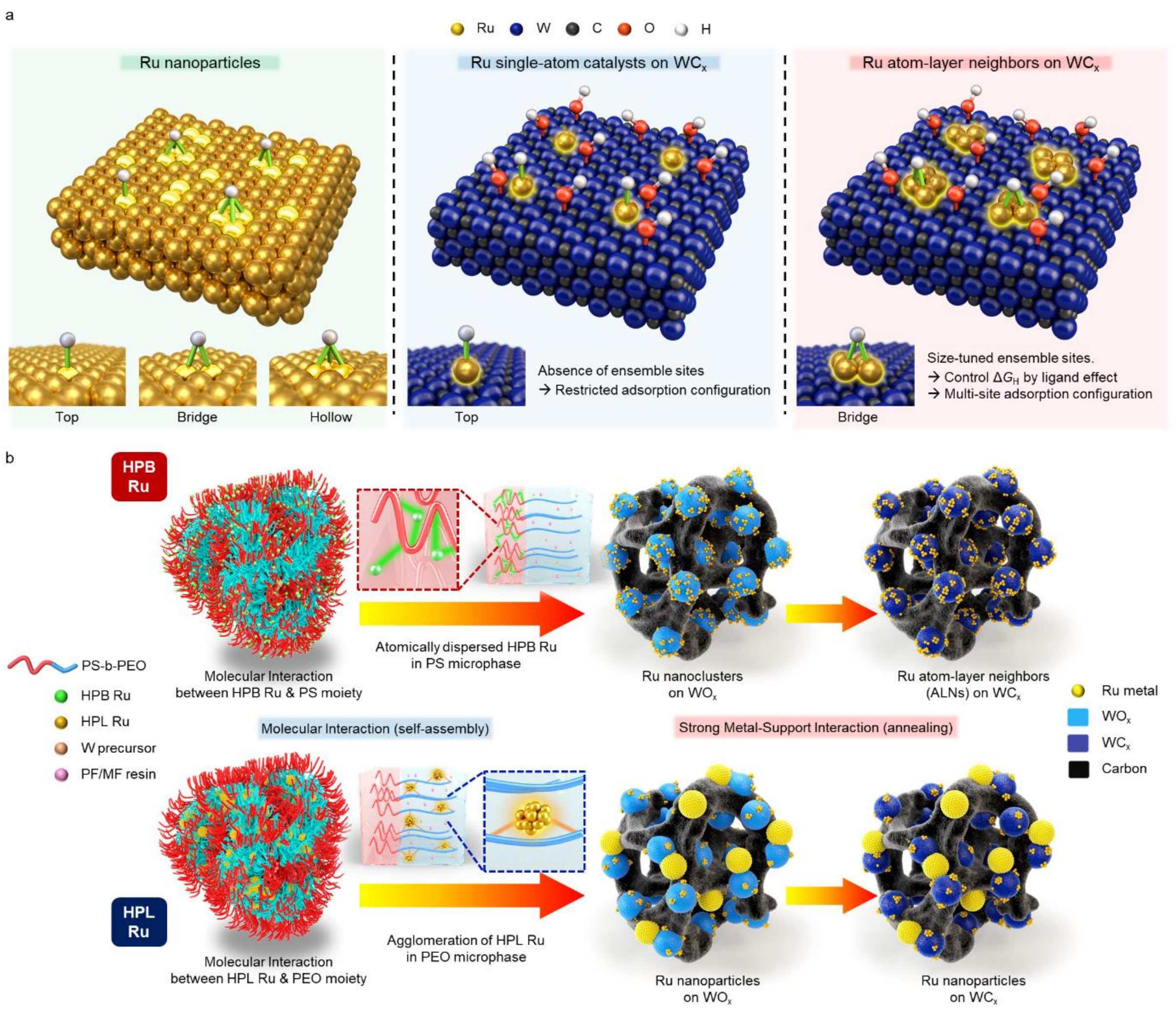

Fig. 1 | Concept of atom-layer neighbors and synthesis strategy via evaporation-induced self-assembly. a, Schematic comparison of Ru nanoparticle (NP) catalysts, Ru single-atom catalysts (SACs), and Ru atom-layer neighbors (ALNs). **b,** Schematic illustration of the thermodynamic stabilization strategy and the differences arising from varying BCP-precursor systems.

(Page 5 in the revised Manuscript)

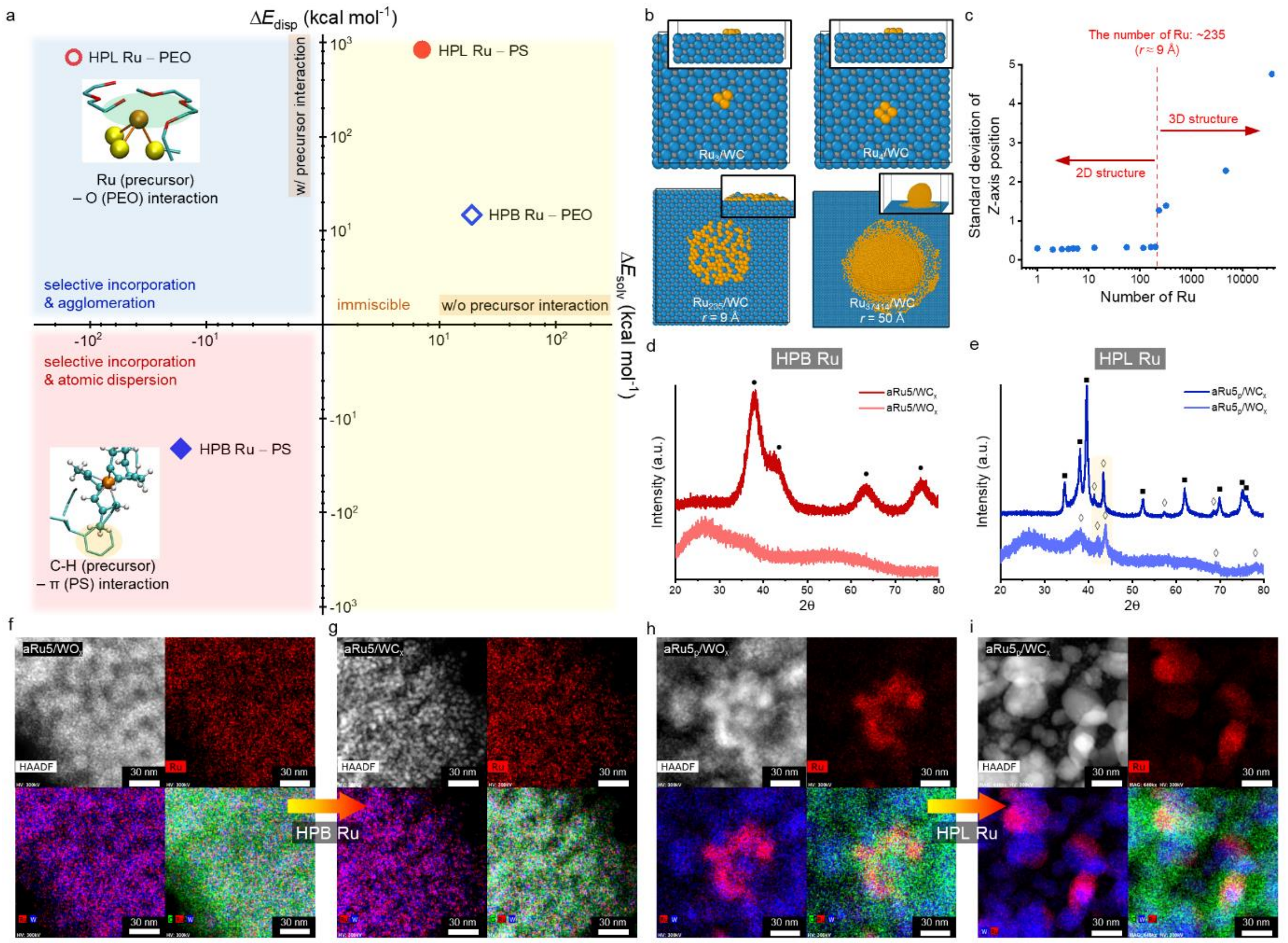

Fig. 2 | Thermodynamic stabilization strategy using molecular interactions (MIs) and strong metal-support interactions (SMSIs). **a,** Relationship between the polymer solvation energy (ΔE_{solv}) and dispersion propensity (ΔE_{disp}) of [Ru precursor], as calculated using MD simulations. **b,** Snapshots from MD simulations of the equilibrated Ru ensembles on WC (Ru_N/WC) structures at 1,200 K (orange: Ru, blue: W, gray: C). **c,** Standard deviation of *Z*-axis positional distribution of Ru species in Ru_N/WC structures as a function of the number of Ru atoms. **d,e,** XRD patterns of (**d**) samples prepared using HPB Ru (aRu5/WO_x and aRu5/WC_x) and (**e**) samples prepared using hydrophilic Ru precursor (HPL Ru) (aRu5$_p$/WO_x and aRu5$_p$/WC_x) (●: γ-WC [PDF 20-1316], ■: α-W_2C [PDF 35-0776], ◊: Ru [PDF 88-1734]). **f-i,** HAADF-STEM images and corresponding EDS mappings of (**f**) aRu5/WO_x, (**g**) aRu5/WC_x, (**h**) aRu5$_p$/WO_x, and (**i**) aRu5$_p$/WC_x (red: Ru, blue: W, green: C).

The original Figure 2 was reorganized as revised Figure 3 with improved panel arrangement and simplified WT-EXAFS visualizations to enhance readability and clarity of the XAS analyses.

(Page 9 in the revised Manuscript)

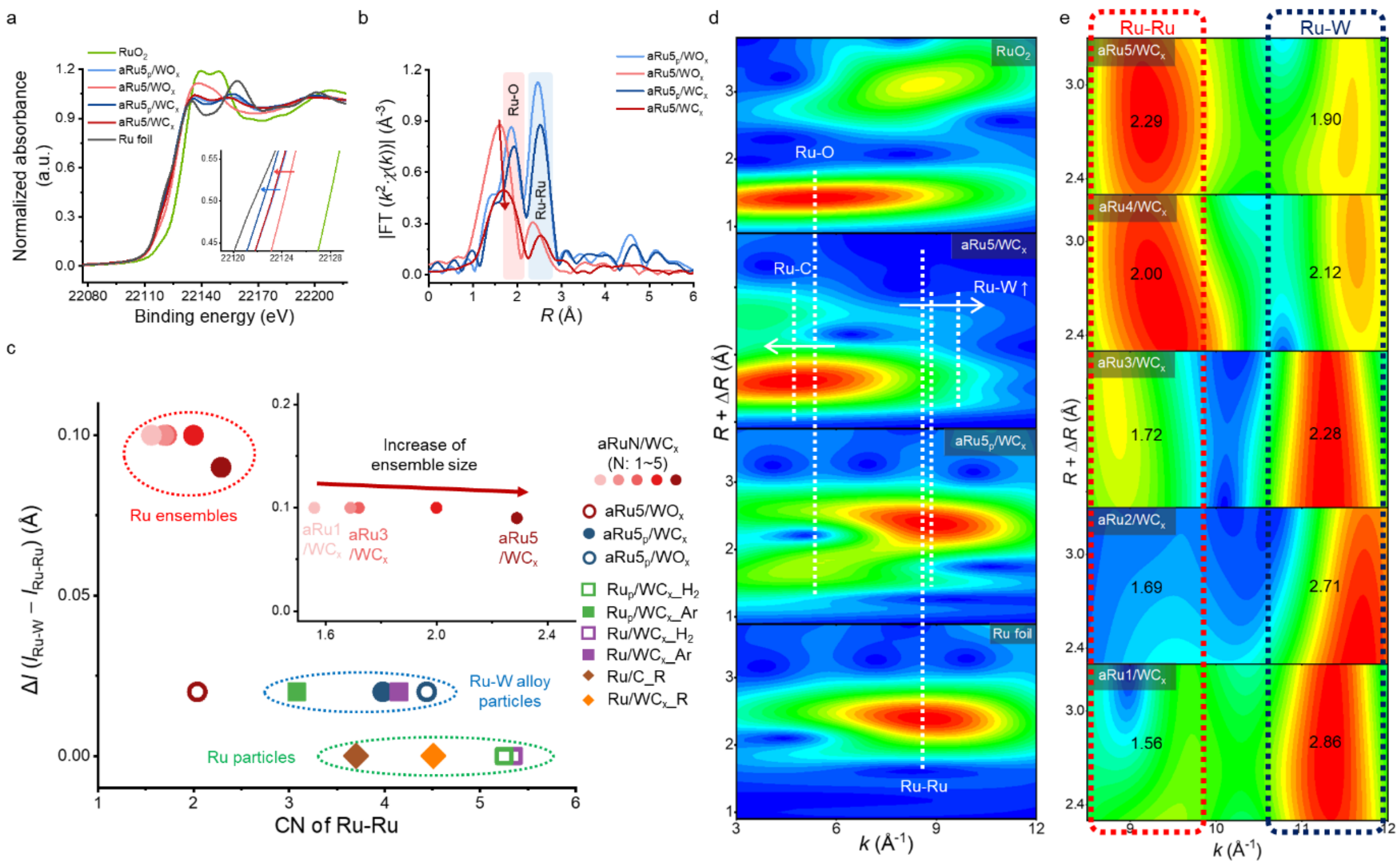

Fig. 3 | Alteration of the geometric and electronic structure of Ru ALNs. a, XANES spectra at the Ru K-edge. **b,** k^2-weighted EXAFS Fourier transform magnitude in R space (χ(R)) at the Ru K-edge of samples prepared using HPL Ru or HPB Ru with different annealing T (red shaded region: Ru-O bonds, blue shaded region: Ru-Ru bonds). **c,** Bond length differences between Ru-W and Ru-Ru bonds as a function of the CN of Ru-Ru bonds, based on EXAFS fitting results for the second shell at the Ru K-edge. **d,** Overview wavelet transforms of aRu5/WC_x and aRu5$_p$/WC_x at the Ru K-edge. **e,** Detailed wavelet transform analyses of aRuN/WC_x at the Ru K-edge second-shell region (red region: Ru-Ru bonds, blue region: Ru-W bonds). The inserted numbers indicate the coordination numbers (CNs) obtained from EXAFS fitting.

The *in-situ* Raman and ATR-SEIRAS figures were revised with improved layout organization and consistent color presentation to facilitate interpretation of the mechanistic data. Spectra collected at different potentials were plotted using consistent color gradients within the *in-situ* XAS panels and within the *in-situ* Raman and ATR-SEIRAS panels to improve visual consistency and readability. In addition, a difference contour map derived from the *in-situ* Raman spectra was added to clearly show the potential-dependent spectral evolution. The ATR-SEIRAS spectra in the revised Supplementary Information were also updated consistently.

(Page 13 in the revised Manuscript)

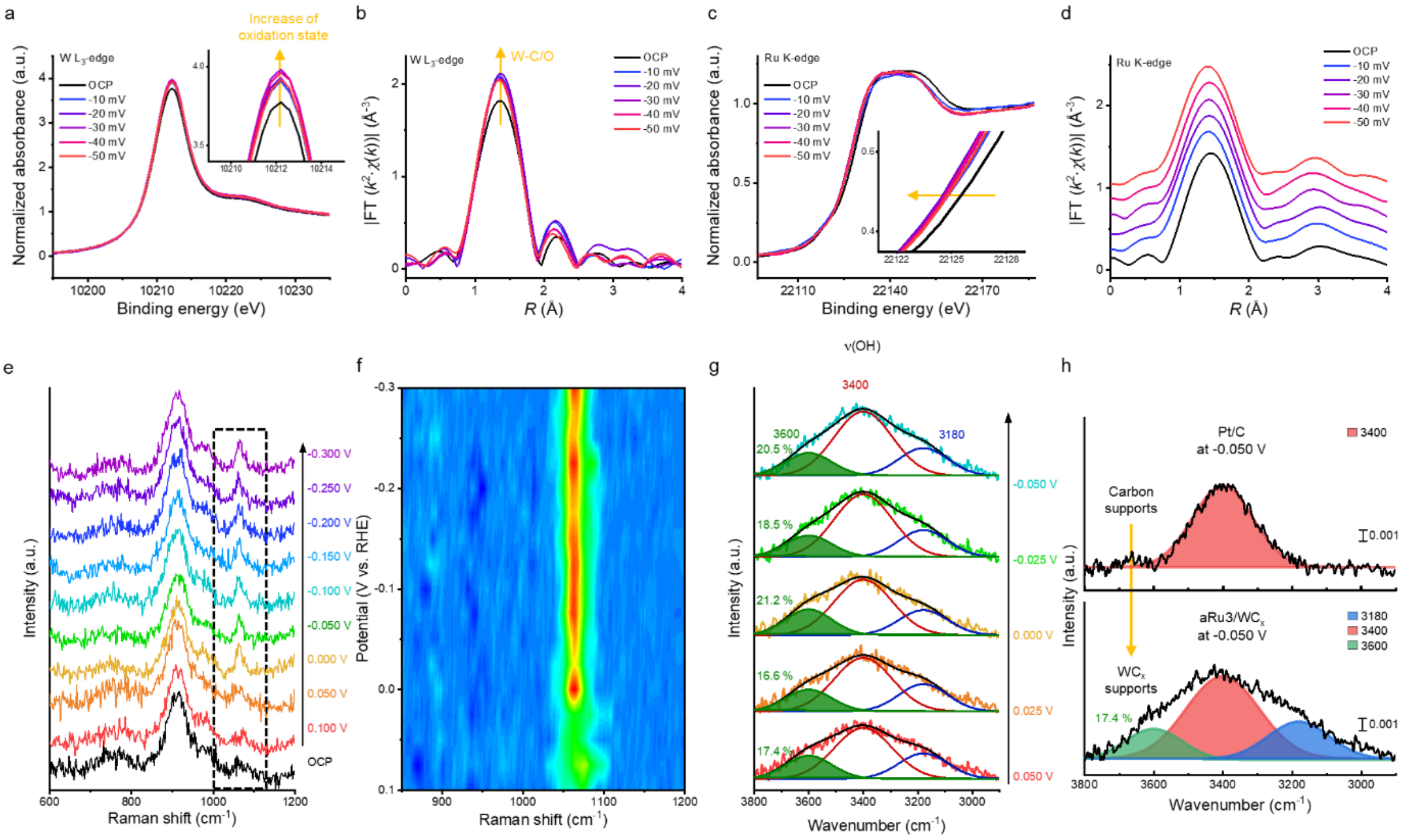

Fig. 5 | Dynamic modulation of the Ru ALNs-WC_x interfacial environment revealed by *in-situ* spectroscopy. a,b, *In-situ* (**a**) XANES and (**b**) corresponding FT-EXAFS spectra at the W L_3-edge of aRu3/WC_x at different potentials. **c,d,** *In-situ* (**c**) XANES and (**d**) corresponding FT-EXAFS spectra at the Ru K-edge of aRu3/WC_x at different potentials. **e,** *In-situ* Raman spectra collected at sequential potentials (green shadow: the coupled O=W⋯W-OH mode (~1060 cm^{-1})). **f**, Difference contour map generated from the *in-situ* Raman spectra, showing the potential-dependent evolution of Raman intensity. **g,** *In-situ* ATR-SEIRAS spectra of aRu3/WC_x at sequential potentials. h**,** Comparison of *in-situ* ATR-SEIRAS spectra at -0.050 V for Pt/C and aRu3/WC_x.

(Page 125 in the revised Supplementary Information)

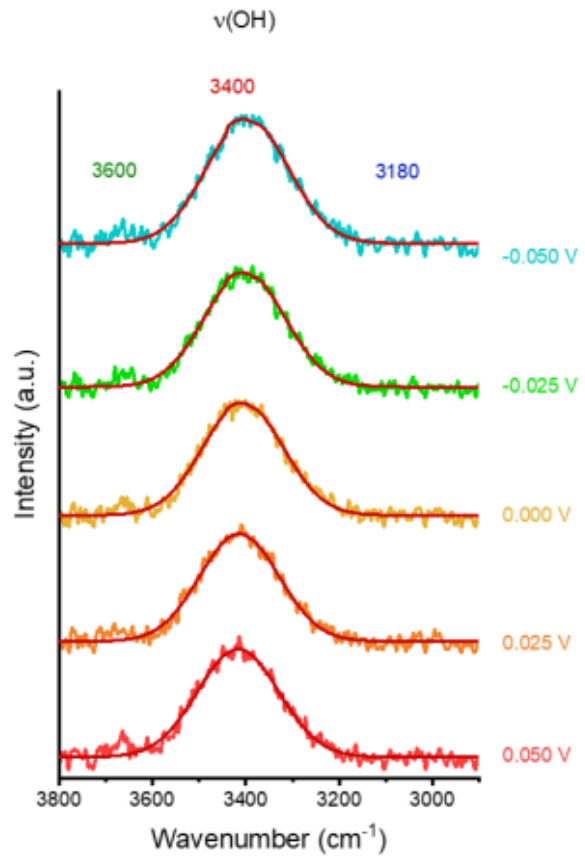

Supplementary Fig. 107 | *In-situ* ATR-SEIRAS results coupled with LSV. *In-situ* ATR-SEIRAS spectra during LSV from 0.050 to -0.050 V (vs. RHE) for commercial Pt/C used for alkaline HER in H_2-saturated 1 M KOH solution.
