# Supplementary material for: Stabilizing ruthenium atom layer neighbors through molecular and metal support interactions for water electrolyzers
Source: Nat Commun. 2026 Sep 22;17:11021. doi: 10.1038/s41467-026-78037-3 (PMC13687079; doi:10.1038/s41467-026-78037-3)
Supplement: Supplementary file 2 [file 41467_2026_78037_MOESM1_ESM.pdf]

<u>**Supplementary Information**</u>

Stabilizing Ruthenium Atom Layer Neighbors through Molecular and Metal Support Interactions for Water Electrolyzers

Seongbeen Kim[1]†, Hyunwoo Jun[1]†, Jihye Park[2,3]†, Sungjun Kim[4]†, Sunghyun Noh[5], Seung-Jae Shin[6], Bupmo Kim[5], Kug-Seung Lee[7], Jiwon Kim[1], Jaeho Byeon[1], Minkyeong Ban[1], Daeeun Choi[1], Hoijun Kim[1], Jong Hyun Jang[8], Wooyul Kim[5], Joonhee Moon[9]*, Jang Yong Lee[10]*, Hyungjun Kim[2]*, and Jinwoo Lee[1]*

Affiliations:

[1]Department of Chemical and Biomolecular Engineering, Korea Advanced Institute of Science and Technology (KAIST); Daejeon 34141, Korea.

[2]Department of Chemistry, Korea Advanced Institute of Science and Technology (KAIST); Daejeon 34141, Korea

[3]Materials Science Division, Lawrence Berkeley National Laboratory; California 94720, United States

[4]Hydrogen Energy Research Center, Korea Research Institute of Chemical Technology (KRICT); Daejeon 34114, Korea

[5]Department of Energy Engineering, Korea Institute of Energy Technology (KENTECH); Naju 58330, Korea

[6]School of Energy and Chemical Engineering, Ulsan National Institute of Science and Technology (UNIST); Ulsan 44919, Korea

[7]Pohang Accelerator Laboratory, Pohang University of Science and Technology (POSTECH); Pohang 37673, Korea

[8]Center for Hydrogen and Fuel Cells, Korea Institute of Science and Technology (KIST); Seoul 02792, Korea.

[9]Research Center for Materials Analysis, Korea Basic Science Institute (KBSI); Daejeon 34133, Korea

[10]Division of Chemical, Biomolecular and Energy Engineering, Konkuk University; Seoul 05029, Korea

†These authors contributed equally to this work.

Correspondence to: junnymoon@kbsi.re.kr (J.M.); ljyljy78@konkuk.ac.kr (J.Y.L.); linus16@kaist.ac.kr (H.K.); jwlee1@kaist.ac.kr (J.L.)

This PDF file includes:

Supplementary Notes 1-13

Supplementary Figures 1-135

Supplementary Tables 1-22

Supplementary References

Other Supplementary Materials for this manuscript include the following:

Supplementary Movies 1-8

Supplementary Notes

Supplementary Note 1 | Size-tuning of Ru ensembles by adjusting the Ru precursor amount

Molecular dynamics (MD) simulations revealed two critical insights: single-atom Ru exhibits high mobility on WC_x supports (Supplementary Fig. 6a, Supplementary Movie 1a), while Ru atom-layer neighbors (ALNs) showed restricted movement due to the proximity of neighboring Ru (Supplementary Fig. 6b-j, Supplementary Movies 1-4, 5a). When considering the poly(ethylene oxide)-*block*-poly(styrene) (PEO-*b*-PS), hydrophobic Ru precursor (HPB Ru), and hydrophilic Ru precursor (HPL Ru) systems, the molecular interaction (MI) between PS moiety and HPB Ru (MIs of [PS-HPB Ru]) supports the assumption of a thermodynamically uniform atomic distribution of HPB Ru within PS microphase after evaporation-induced self-assembly (EISA). During the thermal treatment prior to reaching the carburization temperature (T) of ~923 K, WO_x nanoparticles were generated via the decomposition of tungsten precursors. Because this T is lower than the Tammann T of Ru, the Ru species lacked sufficient mobility to sinter into larger agglomerates[1]. Owing to the weak interaction between Ru and WO_x, Ru nanoclusters were formed, with their size governed by the amount of Ru precursor owing to MIs of [PS-HPB Ru]. Upon reaching a sufficiently high T of 1,173 K, WC_x nanoparticles were formed through a reaction between the carbon scaffold and WO_x nanoparticles. Strong metal-support interactions (SMSIs) between Ru and WC_x facilitated the transformation of Ru nanoclusters into Ru ALNs. Even at the elevated T of 1,173 K, the close proximity of neighboring Ru atoms restricted the migration of ALNs or atomic detachment (Supplementary Fig. 6, Supplementary Movies 1-5), thereby maintaining the ensemble size of Ru ALNs.

Supplementary Note 2 | TG-DSC analysis of precursor-polymer interactions

To experimentally investigate the precursor-polymer interactions underlying the molecular interaction analysis and MD simulations, thermogravimetry analysis with differential scanning calorimetry (TGA-DSC) was performed using solvent-cast precursor/polymer composites. For the measurements, PEO and HPL Ru were completely dissolved in ethanol, while PS and HPB Ru were completely dissolved in THF, followed by solvent evaporation at 50 °C. The precursor-to-polymer ratios were identical to those used during the synthesis of the block copolymer-directed Ru ALN/WC_x catalysts. TGA-DSC measurements were conducted under Ar atmosphere from room temperature to 900 °C at a heating rate of 10 °C min^{-1} (Supplementary Fig. 4).

For the PEO/HPL Ru system, the TG profile of the composite exhibited thermal decomposition behavior distinct from those of pure PEO and pure HPL Ru. The composite could not be described as a simple superposition of the individual components and displayed characteristic mass-loss events at temperatures different from those of either constituent. Consistent changes were also observed in the DSC profile. In particular, the thermal event associated with HPL Ru near 200 °C shifted to higher temperature in the composite, whereas the thermal event associated with PEO near 400 °C shifted to lower temperature. These simultaneous changes indicate the presence of appreciable molecular interactions between HPL Ru and PEO.

A similar tendency was observed for the PS/HPB Ru system. Compared with pure PS and pure HPB Ru, the PS/HPB Ru composite exhibited markedly different TG and DSC behaviors. The sharp thermal event observed for HPB Ru near 100 °C disappeared upon composite formation, while the thermal transition associated with PS shifted to lower temperature. These pronounced changes in thermal behavior support the presence of appreciable molecular interactions between HPB Ru and PS.

The observed thermal behavior demonstrates that the precursor molecules do not behave independently within the polymer matrix but instead interact with the corresponding polymer domains. These results provide experimental support for the molecular interaction calculations and MD simulations presented in Supplementary Note 1 and are consistent with the proposed precursor-partitioning mechanism during the block copolymer self-assembly process.

The observed precursor-polymer interactions are also consistent with previous reports employing block copolymer-directed self-assembly. Ruthenium chloride hydrate (HPL Ru) has been widely utilized in block copolymer systems because of its favorable interactions with PEO domains, enabling the synthesis of ordered mesoporous materials[2-4]. Likewise, hydrophobic organometallic precursors have been reported to preferentially associate with hydrophobic PS domains during block copolymer self-assembly[5,6]. In particular, HPB Ru has been successfully incorporated into mesostructured materials through interactions with hydrophobic polymer domains during one-pot synthesis[7]. Therefore, both the TG-DSC analyses and the established literature precedents support the precursor-partitioning mechanism proposed in this work.

Supplementary Note 3 | Structural evolution and stabilization mechanism of Ru ALNs during carbonization

The structural evolution and stabilization mechanism of Ru ALNs during carbonization were additionally investigated to clarify how the present EISA-derived mesostructure suppresses local collapse and premature aggregation during high-temperature annealing. DFT calculations and molecular interaction analyses indicate that the hydrophobic HPB Ru precursor can be molecularly stabilized within the PS domains through favorable molecular interactions between HPB Ru and the PS moieties (Fig. 2a). As a result, the Ru precursors remain highly dispersed within the PS microphases during solvent evaporation and the early stage of thermal treatment.

Importantly, during annealing, the PS domains are not removed abruptly but are gradually converted into carbonaceous frameworks that subsequently evolve into mesoporous carbon walls[8]. This progressive carbonization process enables the initially dispersed Ru species to remain spatially confined within the evolving carbon framework, thereby suppressing large-scale migration and aggregation during thermal treatment. Consequently, atomically dispersed Ru species can reorganize into size-confined Ru nanoclusters depending on the Ru loading amount, eventually forming ALN structures after carbide formation.

In addition, strong interactions between Ru species and tungsten oxide/carbide domains further stabilize the Ru clusters during high-temperature annealing and suppress sintering. Previous studies have similarly reported that carbon matrices derived from polymeric templates can effectively inhibit nanoparticle aggregation even under high-temperature intermetallic-forming conditions[5], while additional interactions with oxide supports further enhance structural stabilization[9].

Experimental evidence supporting this stabilization mechanism was obtained from the intermediate sample aRu5/WO_x synthesized at 600 °C, where PS-derived carbonization had progressed but carbide formation was not yet completed. High-angle annular dark-field scanning transmission electron microscopy (HAADF-STEM) analyses revealed that Ru nanoclusters remained selectively localized on WO_x domains without formation of large aggregates (Supplementary Fig. 22), indicating that the combined effects of carbon confinement and Ru-WO_x interactions effectively suppress large-scale structural collapse during annealing. Furthermore, Fourier transforms of extended X-ray absorption fine structure (FT-EXAFS) fitting analysis showed that the Ru−Ru coordination number (CN) remained as low as 2.04 together with substantial Ru−W coordination (CN = 1.03) even after partial carbonization (Supplementary Table 8), confirming that the small Ru-cluster structure is preserved throughout the carbonization and carbide-formation process. Moreover, the preservation of the worm-like mesoporous morphology in scanning electron microscopy (SEM) and STEM analyses after carbonization further supports that large-scale local collapse does not occur during the thermal evolution process (Supplementary Figs. 16, 17, 22, 23).

These results collectively indicate that the uniformity of the Ru ALN structures originates from the cooperative stabilization effects of (i) precursor-level molecular dispersion within the PS domains, (ii) spatial confinement provided by the gradually carbonized mesostructure, and (iii) strong Ru−WO_x/WC_x interactions that suppress sintering and large-scale aggregation during carbonization.

Supplementary Note 4 | Interpretation of X-ray diffraction patterns

To examine SMSI with WC_x, low-T annealing at 873 K under Ar was performed on samples prepared via self-assembly. This T was chosen to prevent the carburization of tungsten species. In X-ray diffraction (XRD) patterns, for aRu5/WO_x, no discernible crystalline peaks were observed, suggesting either the absence of crystalline W and Ru phases or the atomic dispersion of Ru (Supplementary Fig. 21a). In contrast, aRu5_p/WO_x showed clear crystalline Ru peaks, indicating that the MI of [PS-HPB Ru] effectively reduced the MI of [HPB Ru], facilitating the atomic dispersion of Ru. On the other hand, the MI of [PEO-HPL Ru] failed to reduce the MI of [HPL Ru], resulting in the formation of large Ru agglomerates.

High-T annealing at 1,173 K converted WO_x to WC_x. Theoretical models revealed thermal migrations of Ru induced by the high temperature, which stabilize Ru ensembles as Ru ALNs on WC_x (Supplementary Fig. 6b-j, Supplementary Movies 1-5). It was also predicted that Ru clusters exceeding a critical size threshold could not stabilize as Ru ALNs (Fig. 2b, c, Supplementary Fig. 6k-m, Supplementary Movie 5). Samples synthesized via self-assembly using HPB Ru (aRuN/WC_x) displayed nanocrystalline γ-WC_x peaks, while samples synthesized with HPL Ru (aRu5_p/WC_x) showed both crystalline α-W_2C and metallic Ru peaks (Supplementary Fig. 21a, b). This suggests that the SMSI with WC_x in aRuN/WC_x effectively prevented thermal sintering of Ru ALNs into nanoparticles (NPs) during high-T annealing, across a wide range of Ru contents (~ 10 wt%). In contrast, large Ru agglomerates exceeding the critical size threshold failed to stabilize as Ru ALNs.

The crystalline phase of WC_x varied with annealing conditions and Ru content, which is influenced by the auto-catalytic effect of noble metal species[10]. Incipient wetness impregnation (IWI)-prepared samples and Ru/WC_x_R displayed crystalline α-W_2C and δ-WC peaks

(Supplementary Fig. 21c). High-T annealing promoted the thermal sintering of Ru, resulting in the appearance of crystalline Ru peaks in Ru/WC_x_Ar and Ru_p/WC_x_Ar. Conversely, low-T reduction limited the thermal sintering of Ru kinetically. However, the strong MI of [HPL Ru] caused the agglomeration of HPL Ru during the IWI process, leading to the crystalline Ru peaks for Ru_p/WC_x_H_2. In contrast, the weaker MI of [HPB Ru] suppressed the agglomeration of HPB Ru to some extent, preventing the appearance of crystalline Ru peaks in Ru/WC_x_H_2. Samples prepared by $NaBH_4$ reduction showed no crystalline Ru peaks, likely due to their low crystallinity (Supplementary Fig. 21d, e).

Supplementary Note 5 | Small-angle X-ray scattering analysis of mesostructural correlations as a function of Ru precursor chemistry and content

Small-angle X-ray scattering (SAXS) analyses were performed to investigate mesostructural correlations and relative variations in structural ordering and porosity as a function of Ru precursor chemistry and content during the EISA process[11]. Conventional scattering profiles (intensity ($I(q)$) vs. q) for samples prepared via self-assembly are presented (Supplementary Fig. 14) to compare the evolution of scattering features among the samples. SEM images (Supplementary Figs. 16, 17) reveal interconnected worm-like mesoporous frameworks rather than highly periodic pore lattices, which is consistent with the broad SAXS features and the absence of distinct higher-order reflections.

To further enhance intermediate-length-scale structural correlations and visualize broad mesostructural features in weakly ordered porous frameworks, the SAXS data were additionally represented as Kratky plots ($I(q)\cdot q^2$ vs. q) (Supplementary Fig. 15) [12]. The WC-carbon nanocomposite frameworks exhibit predominantly short-range mesostructural ordering with broad primary scattering features and weak or poorly resolved higher-order reflections. Under such conditions, analytical fitting models based on ideal pore geometries or long-range periodicity would require strong structural assumptions and may lead to poorly constrained fitting parameters. Therefore, a model-free approach was adopted to conservatively compare relative variations in mesostructural ordering and pore characteristics.

In worm-like mesostructured systems, the SAXS peak position (q^*) reflects the characteristic structural spacing according to $d \approx 2\pi/q^*$, whereas the peak width reflects the structural correlation length within the mesoporous framework. Narrower features generally indicate improved short-range ordering and more uniform mesostructural organization. Since all samples share similar WC-carbon composite frameworks, variations in peak width are mainly attributed to differences in mesostructural organization arising from precursor-dependent self-assembly.

Samples with low or no HPB Ru contents (aRu0-2/WC_x) exhibit broad or weakly defined SAXS features, whereas samples with higher HPB Ru contents (aRu3-5/WC_x) show sharper and more clearly resolved peaks (Supplementary Figs. 14, 15). Consistently, aRu3-5/WC_x exhibit narrower pore size distributions in N_2 physisorption measurements (Supplementary Figs. 9, 10) and more uniform mesostructures in SEM images (Supplementary Figs. 16, 17). In addition, the SAXS peak position gradually shifts toward higher q-values from aRu3/WC_x to aRu5/WC_x, indicating a reduction in characteristic structural spacing. These results suggest that increasing HPB Ru content promotes improved short-range mesostructural ordering and

pore uniformity during the EISA process. Such behavior is likely associated with preferential interactions between HPB Ru and the PS moieties of the block copolymer, which influence mesostructural organization during self-assembly.

Samples prepared using HPB Ru (aRu5/WO_x and aRu5/WC_x) also exhibit narrower SAXS features and pore size distributions than those prepared using HPL Ru (aRu5$_p$/WO_x and aRu5$_p$/WC_x) (Supplementary Fig. 20), indicating improved pore uniformity and structural ordering. During the WO_x-to-WC_x transformation, HPB Ru-derived samples show relatively minor changes in both SAXS peak position and pore size distribution, whereas HPL Ru-derived samples exhibit more pronounced variations, including decreases in q^*, suggesting greater structural rearrangement during carburization. These results indicate that precursor chemistry influences the EISA process through distinct block-specific interactions with the copolymer, thereby affecting porosity evolution and structural integrity during carburization.

At higher q-values (q > ~0.6 nm^{-1}), both the SAXS profiles and Kratky plots exhibit noticeable differences in scattering evolution, particularly for aRu3-5/WC_x (Supplementary Figs. 14, 15). This high-q region probes localized structural features at shorter length scales rather than mesoscopic periodicity. As confirmed by the SEM images (Supplementary Figs. 16, 17) and N_2 physisorption results (Supplementary Figs. 8-10), all samples consist of interconnected worm-like mesoporous frameworks rather than highly periodic pore lattices. In addition, the SAXS profiles do not exhibit well-defined higher-order reflections, indicating limited long-range periodicity. Therefore, the high-q features are attributed to localized structural heterogeneity within the WC-carbon framework rather than long-range mesostructural ordering.

In SAXS, scattering originates from electron-density contrast, while the appearance of distinct high-q features is additionally governed by the spatial distribution of this contrast at the nanoscale[13]. XRD analysis (Supplementary Fig. 21) shows that increasing Ru content promotes the formation of γ-WC via a Ru-induced auto-catalytic effect[10]. Because γ-WC exhibits lower crystallinity than α-W_2C and δ-WC, its formation suppresses the growth of large carbide crystallites and instead favors smaller and more uniformly dispersed WC domains, as supported by SEM and HAADF-STEM analyses (Supplementary Figs. 16, 17, 22-29). Such uniformly distributed nanoscale WC domains embedded within the carbon framework generate more spatially uniform local electron-density contrast at short length scales, giving rise to more clearly defined high-q scattering features. Accordingly, samples with higher atomically dispersed Ru contents (aRu3-5/WC_x) exhibit more pronounced high-q features, which can be attributed to the combined effects of smaller WC domain size, improved dispersion, and more uniform local electron-density contrast within the WC-carbon framework (Supplementary Figs. 14, 15). In contrast, aRu5$_p$/WC_x exhibits less pronounced high-q evolution despite having similar total Ru content to aRu5/WC_x (Supplementary Fig. 20c). This behavior is attributed to the lower fraction of atomically dispersed Ru, which weakens the auto-catalytic effect and results in larger and less uniformly distributed WC agglomerates. Consequently, the reduced uniformity of local electron-density contrast suppresses the high-q scattering feature. Overall, the distinct high-q behavior is attributed to nanoscale structural uniformity within the WC-carbon framework, including WC domain size, dispersion, and the spatial uniformity of local electron-density contrast, rather than differences in long-range mesostructural ordering.

Supplementary Note 6 | Morphology and dispersity of Ru metals

Several previous studies have employed Z-contrast imaging HAADF-STEM to elucidate the atomic structure of noble metal on supports. Typically, the atomic structure of noble metal appears brighter in contrast images due to their higher atomic number (*Z*) compared to support materials[14]. However, for Ru ALNs on WC_x, W has a higher *Z* value than Ru, making W brighter and complicating the differentiation of Ru on thick WC_x support[15,16]. To address this issue, STEM with energy dispersive spectroscopy (EDS) mapping was used to assess the dispersity of Ru and detect its potential agglomerates on WC_x supports.

STEM-EDS images indicated that WC_x supports in aRu*N*/WC_x were uniformly distributed on mesoporous carbon scaffolds with small particle sizes of less than 5 nm (Supplementary Figs. 22-29). This high dispersity significantly increased the specific surface area of WC_x supports available for stabilizing Ru ALNs. Similar to the Ru precursor, the MI of [BCP-W precursor] improved the dispersity of W species within the PEO microphase, enhancing the specific surface area of WC_x supports. STEM-EDS analysis of aRu5/WO_x and aRu5_p/WO_x revealed an amorphous phase of tungsten (Supplementary Figs. 22, 24), consistent with the XRD results (Supplementary Fig. 21). Notably, aRu5_p/WO_x displayed both large Ru agglomerates (orange circles) and atomically dispersed Ru (Supplementary Fig. 24). Similarly, aRu5_p/WC_x showed large Ru agglomerates (orange circles) segregated on WC_x supports (Supplementary Fig. 25). These large agglomerates significantly reduced noble metal utilization efficiency, negatively impacting the catalytic activity per unit mass of noble metal. Nevertheless, the SMSI of WC_x enabled some degree of atomic dispersion of Ru on WC_x supports. For aRu*N*/WC_x, as Ru contents increased, the signal of Ru increased without evidence of the formation of Ru agglomerates (Supplementary Figs. 23, 25-29). Importantly, the Ru signal overlapped with the W signal, indicating selective positioning of Ru on WC_x supports, likely driven by SMSI. This selective positioning facilitated electrocatalysis by enriching *OH species near neighboring Ru ALNs[1].

IWI is a commonly used method for preparing noble metal NP catalysts on support materials. To compare thermodynamic stabilization with the conventional IWI strategy, Ru_p/WC_x_H_2, Ru_p/WC_x_Ar, Ru/WC_x_H_2, and Ru/WC_x_Ar were synthesized via IWI followed by thermal annealing (see Catalyst synthesis in Methods). Low-T reduction (473 K) under 10 v/v% H_2/Ar was typically employed to minimize the thermal sintering of noble metal species. STEM-EDS of Ru/WC_x_H_2 exhibited improved dispersity compared to Ru_p/WC_x_H_2, likely due to the weaker MI of [Ru precursor] (Supplementary Figs. 31, 32). However, without a mechanism to mitigate the MI of [Ru precursor], large Ru agglomerates inevitably formed, reducing noble metal utilization efficiency. These findings highlighted the importance of selecting appropriate precursors for the IWI system based on the MI of [noble metal precursor] to achieve better noble metal dispersion. Both Ru_p/WC_x_Ar and Ru/WC_x_Ar exhibited large Ru agglomerates, suggesting that such agglomerates formed during IWI could not be stabilized as Ru ALNs because their size exceeded a critical threshold (Supplementary Figs. 33, 34). Nevertheless, Ru/WC_x_Ar showed a higher degree of atomic dispersion of Ru on WC_x supports, owing to the more uniform loading of Ru precursor facilitated by the weaker MI of [HPB Ru].

Supplementary Note 7 | Electronic structure variation of Ru catalysts

X-ray photoelectron spectroscopy (XPS) revealed a negative shift in Ru 3*d* and Ru 3*p* peaks during the transition from both aRu5/WO_x to aRu5/WC_x and aRu5_p/WO_x to aRu5_p/WC_x (Supplementary Fig. 41a, c). This shift in the aRu5/WO_x to aRu5/WC_x transition is attributed to the SMSI with metallic WC_x supports, which imparted metallic properties to Ru ALNs by modifying their electronic structures. At low-*T* annealing, Ru agglomerates in aRu5_p/WO_x showed lower crystallinity than those in aRu5_p/WC_x (Supplementary Figs. 21a, 24,25), making them more susceptible to oxidation under ambient conditions[17-19]. High-*T* annealing improved the crystallinity of Ru agglomerates, resulting in the observed negative shift in the XPS Ru peaks.

IWI-prepared samples subjected to low-*T* reduction (Ru/WC_x_H_2 and Ru_p/WC_x_H_2) displayed more positive Ru peaks than high-*T* annealed samples (Ru/WC_x_Ar and Ru_p/WC_x_Ar) (Supplementary Fig. 41b, d). Additionally, samples of HPB Ru (Ru/WC_x_H_2 and Ru/WC_x_Ar) exhibited more positive Ru peaks than HPL Ru samples (Ru_p/WC_x_H_2 and Ru_p/WC_x_Ar). The low-*T* reduction kinetically restricted the thermal sintering of Ru species, while the weaker MI of [HPB Ru] reduced the Ru precursor agglomeration during the EISA process. These led to higher dispersity and lower crystallinity of Ru species, resulting in more cationic properties. According to the surface-sensitive information provided by XPS, several Ru NPs showed highly cationic properties, likely due to their increased susceptibility to oxidation as particle size decreases. However, despite the atomic structure of Ru ALNs, aRu5/WC_x exhibited metallic properties owing to SMSI with WC_x.

In the W 4*f* region, no WC_x peaks were detected for both aRu5/WO_x and aRu5_p/WO_x, indicating that annealing at 873 K effectively prevented the carburization of W species, thereby facilitating the analysis of the SMSI effects with WC_x supports (Supplementary Fig. 42a). After high-*T* annealing, WC_x peaks were observed in both aRu5/WC_x and aRu5_p/WC_x, with no significant peak shifts noticed among the comparison group and main samples. These observations indicate that the WC_x supports maintained a similar electronic state across these samples. (Supplementary Figs. 42-57).

Unlike XPS, X-ray absorption spectroscopy (XAS) analysis provides information averaged across all atoms with the same *Z* value. X-ray absorption near-edge structure (XANES) analysis at the Ru K-edge exhibited a negative shift in the edge position during both the aRu5/WO_x to aRu5/WC_x and the aRu5_p/WO_x to aRu5_p/WC_x transitions (Fig. 3a). The shift during the aRu5_p/WO_x to aRu5_p/WC_x transition suggested increased crystallinity of Ru agglomerates in aRu5_p/WC_x, which would result in more pronounced metallic properties. Despite the atomic structure of aRu*N*/WC_x, the edge positions of aRu*N*/WC_x exhibited a negative shift similar to that of aRu5_p/WO_x. This indicated that the SMSI with WC_x imparted metallic properties to aRu*N*/WC_x similar to those of large Ru agglomerates in aRu5_p/WO_x. Consistent with the XPS results, the XANES analysis of IWI-prepared samples displayed a similar trend in their electronic states (Supplementary Fig. 58).

Supplementary Note 8 | Geometric structure variation of Ru catalysts

The geometric structure of Ru catalysts was characterized using Fourier transforms of extended X-ray absorption fine structure (FT-EXAFS) analysis with corresponding fittings, as

well as wavelet transforms of EXAFS (WT-EXAFS) (Supplementary Figs. 59-67, 84, 85 and Supplementary Tables 8-11). FT-EXAFS with fitting indicated that aRu*N*/WC_x were stabilized by ~4 Ru-C bonds with a bond length of ~2.10 Å, while Ru agglomerates in aRu5_p/WC_x and IWI-prepared samples were coordinated by ~2 Ru-O bonds with a bond length of ~1.98 Å (Supplementary Figs. 59, 63-66, Supplementary Tables 8-10). The longer bond length observed for aRu*N*/WC_x suggests a distinct coordination environment compared to the oxygen-coordinated Ru agglomerates in comparison samples. The fitting of the imaginary part of FT-EXAFS, which is highly sensitive to the coordination environment including the nature of the neighboring atoms[20,21], provides atomic differentiation with a resolution of $Z \approx \pm 2$[22]. The wavelet transform further distinguishes the relative Z by k resolution[22]. Fitting the imaginary part of FT-EXAFS and analysis of the overall WT-EXAFS revealed that the coordinating atoms in the first shell of aRu*N*/WC_x were primarily lighter carbon atoms rather than heavier oxygen atoms (Supplementary Figs. 63-67, 84, 85). Due to the susceptibility of the surface of Ru agglomerates to oxidation in comparison samples, Ru-O bonds were observed for all Ru agglomerates (Supplementary Tables 8-11).

To further clarify whether the first-shell Ru-C coordination originated from carbide-derived carbon in WC_x or from carbonaceous environments such as polymer-derived carbon species, surface carbon contaminants, or graphitic carbon shells, additional EXAFS, STEM-EDS, DFT, and control-sample analyses were performed. In all aRu*N*/WC_x samples, substantial Ru-W coordination was observed together with Ru-C coordination, with Ru-W coordination numbers exceeding unity and Ru-W bond lengths of approximately 2.84 Å (Supplementary Tables 8, 9). If the Ru species were predominantly stabilized by carbonaceous environments rather than WCx domains, direct Ru-W coordination would be expected to be negligible. High-resolution STEM-EDS analyses further showed that Ru signals were selectively correlated with W-containing domains, whereas Ru intensity became negligible in carbon-rich regions, regardless of whether the analyses were performed on selected WC_x particles or carbon-rich areas (Supplementary Figs. 71-74). These results indicate that Ru species are preferentially associated with WC_x domains rather than carbonaceous regions.

Additional DFT calculations were performed for Ru3 and Ru4 ensembles adsorbed on graphene surfaces or confined between graphene layers to model possible carbon-derived coordination environments (Supplementary Fig. 75). Because these DFT models represent idealized local structures, they do not fully capture the structural heterogeneity, defect distribution, solvation environment, dynamic surface reconstruction, or applied-potential effects present under catalytic operating conditions. Therefore, the calculated results are used mainly to compare structural and energetic trends and to guide the interpretation of the experimental observations. Experimentally, the Ru ALN/WC_x samples exhibited elongated Ru-Ru bond lengths (~2.74 Å) compared with bulk metallic Ru (~2.68 Å), and this tendency was reproduced by Ru ensembles stabilized on γ-WC but not by carbon-supported or graphitic shell-confined Ru ensembles (Supplementary Fig. 76). Consistently, the aRu/NC control sample, which contains Ru atomic clusters stabilized on N-functionalized carbon with a Ru-Ru coordination number comparable to that of aRu3/WC_x, exhibited a contracted Ru-Ru bond length of 2.61 Å (Supplementary Figs. 111, 112, Supplementary Table 19). Taken together, these results indicate that carbonaceous environments do not reproduce the characteristic Ru-Ru bond length elongation of Ru ALN/WC_x, supporting the conclusion that the first shell Ru-

C coordination predominantly originates from carbide-derived carbon atoms participating in the Ru ALN/WC_x interfacial structure.

The results from FT-EXAFS with fitting demonstrated that aRu*N*/WC_x were coordinated by Ru-W bonds with a length of ~2.84 Å, suggesting selective positioning of Ru ALNs on WC_x supports owing to the SMSI (Supplementary Tables 8, 9). This selective positioning would facilitate the alkaline hydrogen evolution reaction (HER) kinetics of Ru ALNs by optimizing the interfacial water structure via *OH species on WC_x supports. Notably, the Ru-Ru coordination number (CN) for aRu*N*/WC_x ranged from 1.5 to 2.5, increasing systematically with Ru contents. Theoretical results indicated that each Ru ALN consisted of six or fewer Ru atoms, with the increasing Ru-Ru CN reflecting systematic alterations in ensemble size (Supplementary Table 6).

To further examine the origin of the experimentally observed Ru-W bond length (~2.84 Å), additional DFT calculations were performed for Ru1-Ru6 ensembles adsorbed on W-terminated γ-WC and δ-WC surfaces using various adsorption geometries, including bridge- and hollow-site configurations (Supplementary Figs. 77, 78). For all ensemble sizes, the energetically most favorable structures exhibited average Ru-W bond lengths substantially shorter than the experimentally fitted value and instead closer to those expected for alloy-like Ru-W environments (Supplementary Fig. 79). These results suggest that simple surface-adsorbed Ru ensembles on ideal WC surfaces are unlikely to fully account for the experimentally observed long Ru-W bond length.

Because experimentally fitted EXAFS parameters represent averaged local environments in nanocrystalline WC_x containing defects, local disorder, nonstoichiometric carbide domains, and distributions of Ru ensemble configurations, direct quantitative agreement with idealized periodic DFT models is not necessarily expected. Therefore, rather than focusing solely on absolute bond-length agreement, we compared the experimental Ru-W coordination trends with those predicted by different structural models. Notably, the Ru ALN/WC_x model more consistently reproduced the experimentally observed evolution of Ru-W coordination with increasing Ru ensemble size, whereas Ru ensembles adsorbed on γ-WC and δ-WC surfaces showed less agreement with the experimental trend (Supplementary Fig. 80). These results further support that the experimentally observed ~2.84 Å Ru-W coordination environment is more consistent with the Ru ALN/WC_x configuration than with conventional alloy-like or simple surface-adsorbed Ru structures. The elongated Ru-W bond length may originate from the distinct surface-confined coordination environment of Ru ALNs, where Ru species are simultaneously stabilized through Ru-W and Ru-C interactions on nanocrystalline WC_x rather than within a lattice-constrained metallic alloy framework.

To further evaluate whether the small Ru−Ru CNs of aRu*N*/WC_x could originate from compact three-dimensional Ru clusters rather than two-dimensional Ru ALNs, additional DFT and STEM-simulation analyses were performed. A tetrahedral Ru4 ensemble was constructed on γ-WC and optimized while maintaining the three-dimensional tetrahedral motif (Supplementary Fig. 81). HAADF-STEM image simulations using a 10-layer γ-WC slab indicated that such a small tetrahedral Ru4 cluster is difficult to distinguish unambiguously from small Ru ensembles based solely on projected HAADF-STEM contrast (Supplementary Fig. 82). However, the DFT-optimized tetrahedral Ru4 structure exhibited an average Ru-Ru

bond length of 2.58 Å, substantially shorter than the experimentally observed Ru-Ru bond lengths of the Ru ALN/WC_x samples (~2.74 Å). Similarly, the three-dimensional Ru3 ensemble on γ-WC exhibited an average Ru-Ru bond length of 2.45 Å, shorter than both bulk metallic Ru (2.68 Å) and the experimentally observed Ru ALN/WC_x values (Supplementary Figs. 68, 70). These results suggest that the experimentally observed Ru-Ru bond length elongation is more consistent with Ru ensembles stabilized in the ALN configuration through strong interactions with WC_x than with compact three-dimensional Ru clusters.

This structural assignment was further supported by experimental control samples. The aRu/NC control catalyst, containing Ru atomic clusters stabilized on N-functionalized carbon with a Ru-Ru CN comparable to that of aRu3/WC_x, exhibited a contracted Ru-Ru bond length of 2.61 Å (Supplementary Figs. 111, 112, Supplementary Table 19). In addition, experimentally verified Ru nanoparticle and nanocluster samples, including RuNP/WC_x and Ru/WC_x_R, showed identifiable nanoparticle- or nanocluster-like contrast features in HAADF-STEM images, whereas no analogous features were observed for aRu5/WC_x (Supplementary Figs. 23, 38, 83, 113). The Ru nanoparticles also exhibited a Ru-Ru bond length of ~2.66 Å, slightly shorter than that of bulk metallic Ru and substantially shorter than those of Ru ALN/WC_x samples. Therefore, although Ru-Ru CN alone cannot exclude very small three-dimensional Ru clusters, the combination of elongated Ru-Ru bond lengths, distinct Ru-W coordination, and the absence of nanoparticle-like contrast features supports the assignment of aRu*N*/WC_x as Ru ALN structures rather than compact three-dimensional Ru clusters or nanoparticles.

Bond lengths of Ru-Ru and Ru-W in Ru-W (with dilute W content) alloy agglomerates are expected to be similar, due to shared lattice structures[1]. Indeed, the differences in the bond lengths between Ru-W and Ru-Ru (Δl_{Ru-W}) in samples such as aRu5$_p$/WO_x, aRu5/WO_x, aRu5$_p$/WC_x, Ru/WC_x_Ar, and Ru$_p$/WC_x_Ar were found to be ~0.02 Å, consistent with the formation of Ru-W alloy agglomerates. In contrast, aRu*N*/WC_x displayed a significantly larger Δl_{Ru-W} of ~0.10 Å (Supplementary Tables 8-10), suggesting that Ru species in these samples did not form Ru-W alloys. According to theoretical results, Ru clusters containing fewer than 235 atoms spontaneously stabilized as Ru ALNs at T of 1,173 K due to SMSI with WC_x supports (Fig. 1c, Supplementary Fig. 6). This stabilization is reflected in the large Δl_{Ru-W}, which could be proposed as a structural characteristic of Ru ALN formation. In contrast, aRu5/WO_x exhibited a small Ru-Ru CN of ~2.04 and a Δl_{Ru-W} of ~0.02 Å, indicating that Ru species in aRu5/WO_x formed three-dimensional Ru-W alloy nanoclusters on WO_x supports (Supplementary Table 10). However, after high-T annealing, aRu5/WC_x displayed a significantly larger Δl_{Ru-W} of ~0.10 Å, demonstrating that SMSI with WC_x effectively stabilized Ru ALNs on WC_x supports. In summary, the distinct atomic structure of Ru ALNs was characterized by a large Ru-C/O CN (~4), a large Δl_{Ru-W} (~0.10 Å), and a small Ru-Ru CN (< 2.5), which together provided clear evidence of the unique stabilization of Ru ALNs on WC_x supports.

For Ru$_p$/WC_x_H_2, the results from FT-EXAFS with fitting revealed the presence of Ru-Cl coordination (Supplementary Table 10), indicating that low-T reduction at 473 K under 10 v/v% H_2/Ar was insufficient to fully reduce the precursor, leaving Cl ligands. Similarly, Ru-C coordination was observed for Ru/WC_x_H_2, suggesting the retention of C-containing ligands from HPB Ru (Supplementary Table 10). The reduction at 473 K did not overcome the thermodynamic barrier for Ru-W alloy formation, resulting in large Ru agglomerates. In

contrast, high-T annealing at 1,173 K facilitated the formation of Ru-W alloys, leading to large Ru-W alloy agglomerates in both Ru_p/WC_x_Ar and Ru/WC_x_Ar samples (Supplementary Table 10).

It should also be noted that direct visualization of the Ru ALN configuration using more advanced electron microscopy techniques remains intrinsically challenging for the present catalyst system. Because atomically thin Ru ensembles are stabilized on a heavy W-containing support, the scattering contribution from the underlying WC_x lattice dominates both imaging and diffraction signals, making it difficult to unambiguously separate the contribution of Ru ALNs from that of the support. Furthermore, prolonged electron-beam irradiation can induce structural evolution of sub-nanometer Ru species, as demonstrated by the beam-induced aggregation of Ru atomic clusters in the aRu/NC control sample (Supplementary Movie 8). Consequently, techniques requiring extensive data acquisition or prolonged beam exposure, such as interatomic-distance statistical analysis, 4D-STEM, iDPC-STEM, and electron tomography, are difficult to apply without potentially perturbing the original atomic configuration. Therefore, rather than relying on a single local microscopy measurement, the Ru ALN structure was evaluated through the agreement between multiple independent theoretical and experimental descriptors, including STEM-EELS analyses, EXAFS-derived coordination environments, characteristic Ru-Ru bond-length elongation, distinctive Ru-W coordination environments, and DFT/MD-predicted structural parameters.

Among the examined structural models, the Ru ALN model most consistently reproduces the experimentally observed structural fingerprints. Specifically, the Ru ALN configuration accounts for (i) the thermodynamic preference for atomically thin Ru ensembles predicted by DFT and MD simulations, (ii) the characteristic Ru-Ru bond length elongation relative to bulk Ru, (iii) Ru-W coordination numbers exceeding unity together with the distinctive Ru-W bond length of ~2.84 Å, (iv) direct Ru-C anchoring interactions identified by EXAFS and WT-EXAFS analyses, and (v) the ensemble-size-dependent evolution of structural parameters observed experimentally. In contrast, alternative structural models, including three-dimensional Ru clusters, carbon-supported Ru ensembles, Ru nanoparticles, and Ru-W alloy structures, do not reproduce one or more of these experimentally observed descriptors. Therefore, the convergence of these independent theoretical and experimental observations supports the assignment of Ru species in aRuN/WC_x as predominantly stabilized as Ru atom-layer neighbors (ALNs) on WC_x supports.

Supplementary Note 9 | Origin of the Ru-Ru EXAFS contribution in aRuN/WC_x

The origin of the Ru-Ru EXAFS contribution in aRuN/WC_x was investigated through EXAFS fitting analyses, XANES linear combination fitting (LCF), theoretical calculations, and experimentally verified control samples. Particular attention was paid to distinguishing the Ru ALN structure from residual RuO_x species and incompletely dispersed Ru clusters, both of which could potentially contribute to the Ru-Ru scattering signal.

The possibility of residual RuO_x contributions was first examined by comparing EXAFS fitting results obtained using Ru-C and Ru-O first shell scattering models. For $aRu3/WC_x$, the Ru-C model provided good agreement with the experimental spectrum with an R-factor of 0.003, whereas the Ru-O model resulted in a substantially poorer fit with an R-factor of 0.045 (Supplementary Fig. 86, Supplementary Table 12). Since R-factors exceeding ~0.020 are

generally considered indicative of unreliable fitting results, the Ru-O model cannot satisfactorily describe the first shell coordination environment of aRu3/WC_x. These results support the assignment of the first shell coordination to Ru-C rather than Ru-O species.

Additional evidence was obtained from comparison with the RuO_2 reference. The characteristic Ru-Ru paths of RuO_2 are located at approximately 3.17 and 3.57 Å, whereas the Ru-Ru bond length obtained for aRu3/WC_x is only ~2.76 Å. Furthermore, the characteristic FT-EXAFS feature of RuO_2 near 3 Å (Supplementary Fig. 63d) is absent in aRu3/WC_x (Supplementary Fig. 64j), which instead exhibits only the peak near 2.5 Å. Consistently, WT-EXAFS analysis revealed that the first shell maximum intensity of aRu*N*/WC_x is shifted toward lower *k* values compared with RuO_2, indicating coordination by lighter scatterers than oxygen (Fig. 3d, Supplementary Figs. 84, 85). Together, these results indicate that the observed Ru-Ru contribution cannot be reasonably attributed to residual RuO_x species.

XANES linear combination fitting (LCF) was performed using Ru metal and RuO_2 standards (Supplementary Fig. 87). Although the lowest0residual fit was obtained using 62.4% Ru metal and 37.6% RuO_2 contributions, noticeable discrepancies remain between the fitted and experimental spectra, particularly in the XANES region. Therefore, the local coordination environment of Ru in aRu3/WC_x cannot be fully described by a simple combination of metallic Ru and RuO_2 reference structures, suggesting the presence of a distinct Ru coordination environment.

The possibility that the observed Ru-Ru contribution originates from incompletely dispersed Ru species was also examined. Since small Ru ensembles can exhibit low Ru-Ru coordination numbers similar to those observed for Ru ALNs, coordination number alone is insufficient to distinguish incompletely dispersed Ru clusters from Ru ALNs. Therefore, additional structural descriptors were examined through both theoretical and experimental approaches.

To understand the structural origin of the elongated Ru-Ru bond lengths observed for aRu*N*/WC_x, various theoretical models were systematically compared, including Ru3 and Ru4 ensembles adsorbed on graphene, Ru3 and Ru4 ensembles confined between graphene layers, three-dimensional Ru3 clusters and tetrahedral Ru4 clusters on γ-WC, and two-dimensional Ru3 and Ru4 ALNs stabilized on γ-WC (Supplementary Figs. 6c, d, 68b, 71, 81). For the graphene-supported, graphene-confined, and three-dimensional Ru cluster models, the calculated Ru-Ru bond lengths were consistently shorter than that of bulk metallic Ru (~2.68 Å), indicating contraction of the Ru ensembles (Supplementary Fig. 72). In contrast, the Ru ALN models stabilized through simultaneous Ru-C and Ru-W coordination on WC_x exhibited elongated Ru-Ru bond lengths that were more consistent with the experimentally observed trend. These results indicate that Ru-Ru bond-length elongation is not a general feature of small Ru ensembles but instead emerges specifically when the Ru species are stabilized as Ru ALNs through strong interactions with the WC_x support.

This tendency was further verified experimentally using control samples representing incompletely dispersed Ru species. Ru atomic clusters supported on N-functionalized carbon (aRu/NC) were synthesized, and HAADF-STEM analyses confirmed the formation of three-dimensional atomic clusters composed of fewer than ten Ru atoms (Supplementary Figs. 111, 112). EXAFS fitting revealed that aRu/NC exhibited a Ru-Ru coordination number of 1.92, comparable to that of aRu3/WC_x (1.72), indicating that coordination number alone cannot

distinguish incompletely dispersed Ru species from Ru ALNs. However, the Ru-Ru bond length of aRu/NC was only 2.61 Å, which is even shorter than that of bulk metallic Ru (~2.68 Å) (Supplementary Table 19). Similarly, Ru nanoparticles supported on WC_x and carbon supports exhibited Ru-Ru bond lengths of ~2.66 Å despite possessing Ru-Ru coordination numbers comparable to those of the Ru ALN samples. Therefore, although incompletely dispersed Ru species can exhibit similarly low Ru-Ru coordination numbers, they consistently display contracted Ru-Ru bond lengths. In contrast, aRu3/WC_x exhibits a significantly elongated Ru-Ru bond length (~2.76 Å), which is opposite to the trend observed for incompletely dispersed Ru species and is consistent with the theoretical prediction for Ru ALNs stabilized through strong interactions with WC_x.

Collectively, the EXAFS fitting comparison, WT-EXAFS analysis, RuO_2 reference analysis, LCF results, DFT calculations, and experimentally verified control samples support that the Ru-Ru contribution observed in aRu*N*/WC_x is unlikely to originated primarily from residual RuO_x species or incompletely dispersed Ru clusters. Instead, the experimentally observed Ru-Ru bond-length elongation is more consistent with Ru ALN structures stabilized through simultaneous Ru-C and Ru-W coordination on WC_x. The Ru-Ru bond length elongation therefore represents a characteristic structural feature of the Ru ALN configuration and supports the assignment of the Ru species in aRu*N*/WC_x as Ru ALNs stabilized on WC_x supports.

Supplementary Note 10 | Alkaline hydrogen evolution reaction activity of Ru catalysts

To prepare comparison samples featuring nanoparticle structures of Ru, Ru NPs were synthesized on WC_x-C nanocomposite supports (Ru/WC_x_R) and commercial carbon supports (Ru/C_R) via chemical reduction of HPL Ru. The noble metal loadings of these samples were ~20 wt%, similar to that of commercial Pt/C catalysts (Supplementary Table 7, see Catalyst synthesis in Methods). The Ru NPs were uniformly dispersed on the support surfaces (Supplementary Figs. 21, 35, 36) and exhibited low Ru-Ru CN of 4.51 for Ru/WC_x_R and 3.70 for Ru/C_R, along with surface Ru-O bonding, indicating sufficiently small Ru NP sizes (Supplementary Table 11).

Under alkaline conditions (H_2-saturated 1 M KOH), the HER activity of aRu*N*/WC_x displayed a volcano-type dependence on the ensemble size of Ru ALNs. Among the series, aRu3/WC_x exhibited the lowest overpotential for -10 mA cm^{-2} (η_{10}) at 22 mV (potentials referenced to the reversible hydrogen electrode, RHE) and a Tafel slope of 41.9 mV dec^{-1} (Supplementary Fig. 91a, Supplementary Table 17). Additionally, aRu3/WC_x showed a current density of -1202.0 mA mg_{mass}^{-1} at an overpotential of 50 mV per noble metal mass ($j_{50/mass}$). The systematic variation in Ru-Ru CN across the aRu*N*/WC_x series with increasing Ru contents revealed changes in the predominant Ru ensemble structures on WC supports (Ru_N/WC) (Supplementary Tables 6, 9). Among the aRu*N*/WC_x and Ru NP samples (Ru/C_R and Ru/WC_x_R), aRu3/WC_x showed high kinetic activity for alkaline HER. DFT calculations suggested that Ru_3/WC and Ru_4/WC structures have Gibbs free energies of hydrogen adsorption (ΔG_H) closer to thermoneutral values than other Ru_N/WC structures and Ru (0001) (Supplementary Fig. 90a). These findings are consistent with Ru_3/WC- or Ru_4/WC-like local structures being major contributors to the observed activity of aRu3/WC_x (Supplementary Fig.

88, Supplementary Table 15). The size-dependent modulation of *H binding, together with high noble metal utilization efficiency, may contribute to the enhanced kinetic activity (Supplementary Figs. 91-94).

Despite using the same relative ratio of Ru precursor to W precursor, aRu5/WC_x (obtained with HPB Ru) showed higher performance than aRu5$_p$/WC_x (obtained with HPL Ru) under the tested conditions. The Ru-Ru CN of aRu5/WC_x was 2.29 (Supplementary Table 8), indicating that the dominant structures in this sample were likely Ru_5/WC_x and Ru_6/WC_x (Supplementary Table 6). Although Ru_5/WC_x and Ru_6/WC_x exhibited less favorable ΔG_H compared to Ru (0001), the higher kinetic activity of aRu5/WC_x can be attributed to the higher noble metal utilization efficiency of Ru ALNs compared to Ru agglomerates.

Supplementary Note 11 | General applicability of the ALN stabilization strategy on WC_x supports

The general applicability of the present ALN stabilization strategy was additionally investigated using Pt and Ir precursors on WC_x supports. Stabilization of ALN structures on carbide supports requires that the metal species remain preferentially confined at the carbide surface during high-temperature annealing, rather than diffusing into the carbide lattice or forming thermodynamically stable mixed-carbide phases. In the case of many transition metals such as Fe, Co, Ni, and Mo, strong carbide-forming tendencies and relatively high solid solubility in tungsten carbide lattices can promote lattice diffusion, alloying, or mixed carbide formation during high-temperature treatment, thereby making the stabilization of surface confined ALN structures substantially more challenging under the present synthetic conditions[23,24].

In contrast, noble metals such as Ru, Ir, and Pt exhibit substantially lower solubility in tungsten carbide lattices and weaker tendencies toward bulk carbide formation under reductive annealing environments[1,25,26]. As a result, noble metal species can remain preferentially stabilized at the WC_x surface through SMSIs, thereby enabling the formation of two-dimensional ALN structures. To examine the thermodynamic feasibility of extending the stabilization mechanism to other noble metals, DFT calculations were additionally performed for Pt3, Ir3, Ru3, Pt4, Ir4, and Ru4 clusters on γ-WC supports (Metal$_N$/WC, N: the number of metal atoms) (Supplementary Fig. 98). In all cases, the noble metal clusters were preferentially stabilized as two-dimensional surface confined structures through strong interactions with γ-WC. Furthermore, comparison between the cohesive energies of the noble metals (E_{coh}) and the binding energies of the noble metal ALNs on γ-WC (E_{bind}) revealed positive $E_{coh} - E_{bind}$ values for all investigated systems (Supplementary Fig. 99), indicating that stabilization of surface confined noble metal ALNs on γ-WC is thermodynamically more favorable than metal aggregation.

To experimentally examine the generalizability of the present synthetic strategy, Pt- and Ir-based catalysts were synthesized using the same procedure employed for aRu3/WC_x, except that dimethyl(1,5-cyclooctadiene)platinum(II) (HPB Pt) and ((1,5-cyclooctadiene)-η^5-indenyl)iridium(I) (HPB Ir) were used as hydrophobic noble metal precursors with identical molar loading conditions. SEM, N_2 physisorption, and SAXS analyses revealed that both aPt3/WC_x and aIr3/WC_x exhibit structural characteristics analogous to those of aRu3/WC_x, including worm-like mesoporous frameworks with short-range mesostructural ordering (Supplementary Figs. 100a-f, 101a-f). These

results indicate that the mesostructural framework generated through EISA process can be extended to other noble metal precursor systems.

However, distinct differences in noble metal stabilization behavior were observed between Pt and Ir. XRD analysis of aPt3/WC_x showed discernible crystalline Pt diffraction peaks, whereas no crystalline Ir peaks were observed for aIr3/WC_x (Supplementary Figs. 100g, 101g). Consistently, HAADF-STEM analyses revealed that both Pt and Ir species were preferentially localized on the WC_x supports, indicating strong SMSIs with WC_x (Supplementary Figs. 102, 103). Nevertheless, aPt3/WC_x exhibited partially aggregated bright nanoclusters in the Z-contrast images, whereas aIr3/WC_x showed highly dispersed Ir species without observable nanocluster formation.

XPS analyses further confirmed that both aPt3/WC_x and aIr3/WC_x possess electronic structures analogous to those of aRu3/WC_x, including metallic noble metal species and electronically similar WC_x supports (Supplementary Figs. 104, 105). Because the Pt and Ir L_3-edges overlap with the W L_2-edge, XAS analyses were conducted at the Pt and Ir L_2-edges instead. Both aPt3/WC_x and aIr3/WC_x exhibited white-line intensities similar to those of the corresponding metal foils, indicating that the noble-metal species retain predominantly metallic character.

Notably, FT-EXAFS analyses revealed substantially different local coordination environments for Pt and Ir. In the case of aPt3/WC_x, the FT-EXAFS spectra closely resembled those of Pt foil. Quantitative fitting analysis revealed a Pt-Pt CN of 4.10 with a bond length of 2.75 Å, similar to that of Pt foil (2.76 Å). Nevertheless, additional Pt-C and Pt-W coordination components were identified, indicating that Pt nanoclusters remain anchored on WC_x through SMSIs. In contrast, aIr3/WC_x exhibited a local structure substantially different from that of Ir foil. EXAFS fitting analysis revealed a low Ir-Ir CN of 1.87 with an elongated Ir-Ir bond length of 2.73 Å compared with Ir foil (2.70 Å), together with substantial Ir-C coordination (CN = 2.46) and an Ir-W bond length of 2.88 Å. These structural characteristics are highly consistent with those observed for Ru ALNs, including low metal-metal CNs (< 2.5), substantial metal-C coordination, and enlarged metal-W bond distances relative to metal-metal bonds (Supplementary Note 8). These results suggest that Ir can also be stabilized as ALN-like structures on WC_x through the same stabilization mechanism.

The different stabilization behaviors observed for Pt and Ir likely originate from differences in precursor-block copolymer interactions during the EISA process. Although DFT calculations suggest that Pt clusters can exhibit thermodynamically favorable interactions with WC_x, insufficient molecular-level stabilization of the HPB Pt precursor within the PEO-*b*-PS framework during solvent evaporation and annealing may promote partial Pt aggregation prior to ALN stabilization. These results suggest that thermodynamically favorable stabilization on WC_x alone is insufficient for ALN formation, and that precursor-directed molecular interactions during EISA likely play an important role in suppressing premature aggregation and enabling the formation of surface confined ALN structures.

The alkaline HER activities (Supplementary Fig. 106) of the resulting catalysts were additionally evaluated at identical noble-metal loadings (42 μg cm^{-2}). aPt3/WC_x exhibited η_{10} of 26 mV and a Tafel slope of 25.8 mV dec^{-1}, comparable to those of aRu3/WC_x, whereas aIr3/WC_x showed relatively lower HER activity with an η_{10} of 51 mV and a Tafel slope of 97.2 mV dec^{-1}. Collectively, these results suggest that the present synthetic strategy can, in principle, be extended to other noble metals, while the successful formation of well-defined ALN structures strongly depends on

molecular-level precursor stabilization within the block copolymer framework during the EISA process.

Supplementary Note 12 | Functions of *OH on WC_x supports for efficient alkaline HER: hydrogen bond connectivity

Theoretical calculations suggest that Pt (111) has a ΔG_H closer to the thermoneutral value than Ru_2/WC or Ru_3/WC structures. However, the commercial Pt/C exhibited lower kinetic activity than aRu3/WC_x (Supplementary Fig. 91). Previous studies have attributed the sluggish kinetics of Pt catalysts to reduced hydrogen bond (H-bond) network connectivity in interfacial water. Furthermore, the presence of *OH species adjacent to the active sites for *H adsorption has been shown to accelerate the kinetics of alkaline HER by enhancing the H-bond network. DFT calculations revealed that WC supports have a more favorable Gibbs free energy of hydroxyl group adsorption (ΔG_{OH}) compared to Pt (111) and carbon supports. This suggests that *OH species spontaneously adsorb onto WC surfaces, enriching *OH coverage on WC surfaces (Supplementary Fig. 90b, Supplementary Table 16). This theoretical prediction is consistent with experimental data: during the alkaline HER, the white line intensity of aRu3/WC_x in in situ XANES analysis at the W L_3-edge progressively increased with increasing current density, indicating *OH enrichment on WC_x supports (Fig. 5a, b)[27,28].

To analyze the H-bond network connectivity in interfacial water, in situ attenuated total reflection surface-enhanced infrared absorption spectroscopy (ATR-SEIRAS) was performed. The O-H stretching mode ($\nu_{O\text{-}H}$) of water exhibits peak shifts depending on the strength of H-bonding. Previous studies assigned peaks at 3600, 3400, and 3180 cm^{-1} to weakly H-bonded water (free water), trihedrally coordinated H-bonded water (liquid-like water), and tetrahedrally coordinated H-bonded water (ice-like water), respectively. A reduced population of gap water weakens the H-bond network, thereby slowing the kinetics of alkaline HER. In the gap region, water forms relatively weak H-bonds, which manifest as a detectable peak at 3600 cm^{-1}. Within a potential range of 0.050 to -0.050 V, commercial Pt/C showed a negligible peak at 3600 cm^{-1}, indicating a scarcity of gap water species (Supplementary Fig. 107). In contrast, aRu3/WC_x exhibited a significantly enhanced peak at 3600 cm^{-1} across the entire potential window, signifying an increased population of gap water species (Fig. 5g). This increased presence of gap water species strengthens the H-bond network connectivity, effectively accelerating the alkaline HER kinetics for aRu3/WC_x. Taken together, these results indicate that the WC_x support primarily enhances proton-transfer kinetics from the bulk electrolyte to the electrode-electrolyte interface by improving interfacial H-bond network connectivity, while the Ru ALNs remain the principal HER-active sites for hydrogen adsorption and H_2 evolution.

Supplementary Note 13 | Post-durability analyses of single-cell operation for Pt/C, Ru/C, and aRu3/WC_x

The HAADF-STEM images of the pristine commercial Pt/C and Ru/C cathodes showed that numerous nanoparticles (< 3 nm) were uniformly distributed on the carbon supports (Supplementary Figs. 132a, 133a). However, after the anion-exchange membrane water

electrolyzer (AEMWE) single-cell durability test, both catalysts exhibited severe nanoparticle agglomeration, forming large aggregates with locally concentrated distributions on the supports (Supplementary Figs. 132b, c, 133b, c). The corresponding EDS elemental mappings further revealed pronounced Fe/Ni deposition, originating from the migration and reduction of dissolved Fe/Ni ions from the anode (Supplementary Figs. 132, 133). As previously reported by Kwon and co-workers, both impurity redeposition and cathodic nanoparticle instability contribute to the initial performance degradation commonly observed in AEMWE systems[29]. In particular, their operando three-electrode analyses identified Pt nanoparticle agglomeration as the dominant contributor to the rapid increase in cathodic kinetic and residual overpotentials during the early operation stage, whereas impurity deposition alone did not fully account for the degradation behavior.

In our case, the aRu3/WC_x cathode also experienced similar Fe/Ni deposition (Supplementary Fig. 134); however, it largely preserved the atomic structure of Ru species on the WC_x supports, showing only minor and localized Ru agglomeration even after prolonged AEMWE operation. The SEM and high-resolution HAADF-STEM images confirmed that the mesoporous morphology and atomic-scale Ru dispersion of aRu3/WC_x were retained without formation of large Ru aggregates (Supplementary Fig. 135a-c). In addition, both the XANES and FT-EXAFS spectra exhibited nearly identical features before and after the durability test (Supplementary Fig. 135c, d). A slight increase in the second-shell intensity in the FT-EXAFS spectra (Supplementary Fig. 135d) suggests minor Ru-transition metal or Ru-Ru coordination, likely caused by Fe/Ni adsorption or limited Ru aggregation, whereas the preserved first-shell Ru-C coordination confirms that the atomic structure of Ru remained largely intact.

These results collectively indicate that, although Fe/Ni redeposition may partially contribute to active site blocking and initial performance degradation in AEMWEs, the substantially different durability behaviors among the investigated cathodes correlate more strongly with the degree of noble metal migration, aggregation, and dissolution. In this regard, the SMSI between Ru ALNs and WC_x supports effectively suppresses Ru migration and aggregation, thereby contributing to the lower degradation of aRu3/WC_x compared with conventional nanoparticle catalysts.

It should be noted that Fe/Ni redeposition remains a fundamental challenge for practical AEMWE operation, as it originated from the oxidative dissolution of transition-metal species at the anode and their subsequent transport through the membrane-electrolyte interface. Mitigating this process requires not only stable cathode materials but also advances in anode catalyst design, membrane engineering, and integrated cell architecture, which collectively govern cross-contamination phenomena in AEMWE systems. Therefore, improving the overall durability of AEMWEs demands coordinated progress in these complementary areas of research beyond cathode development alone.

Supplementary Figures

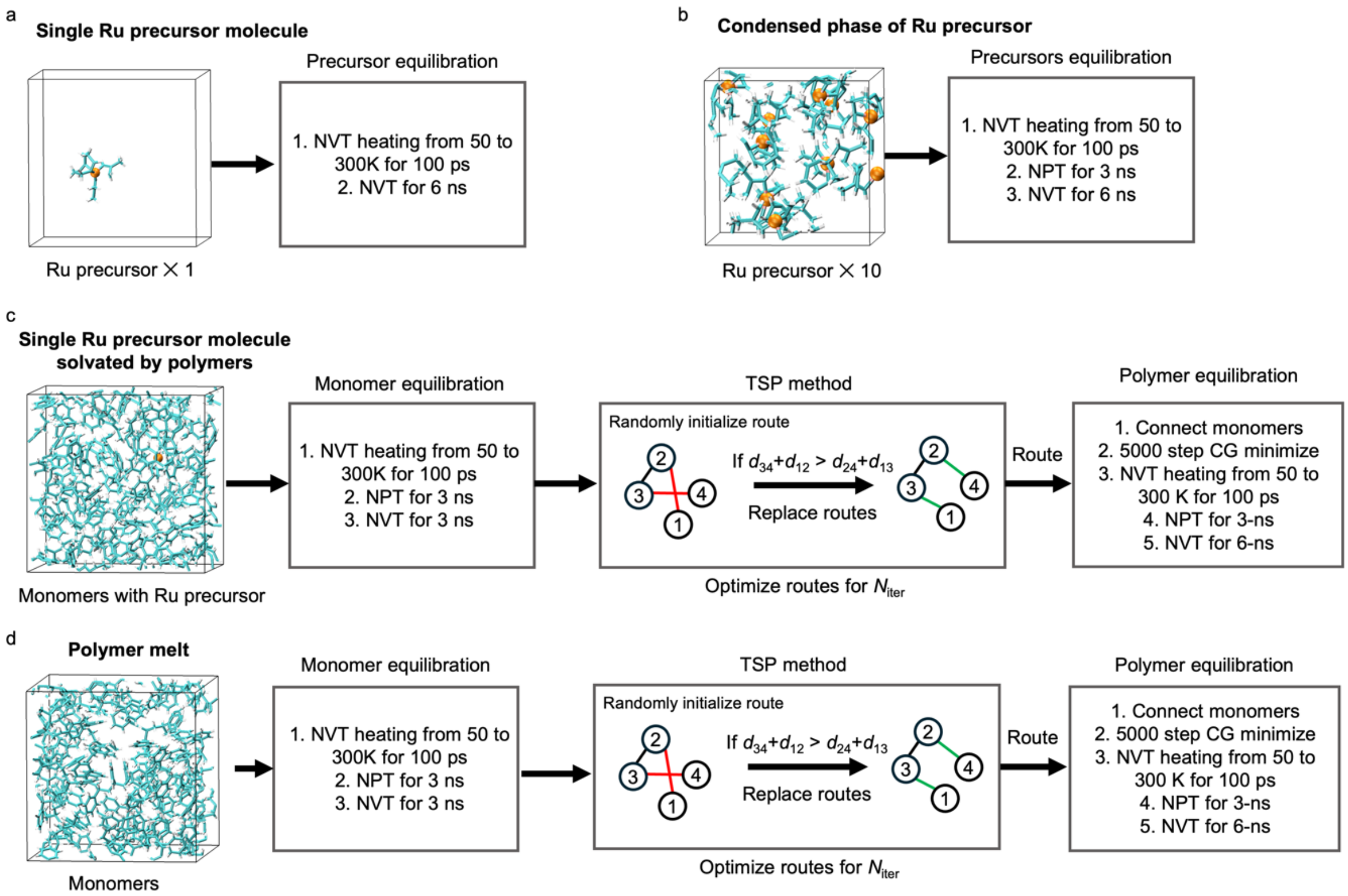

Supplementary Fig. 1 | Overview of the simulation procedures. a-d, Simulation procedures for preparing systems involving (**a**) a single Ru precursor molecule, (**b**) a condensed phase of Ru precursor, (**c**) a single Ru precursor molecule solvated by polymers, and (d) a polymer melt.

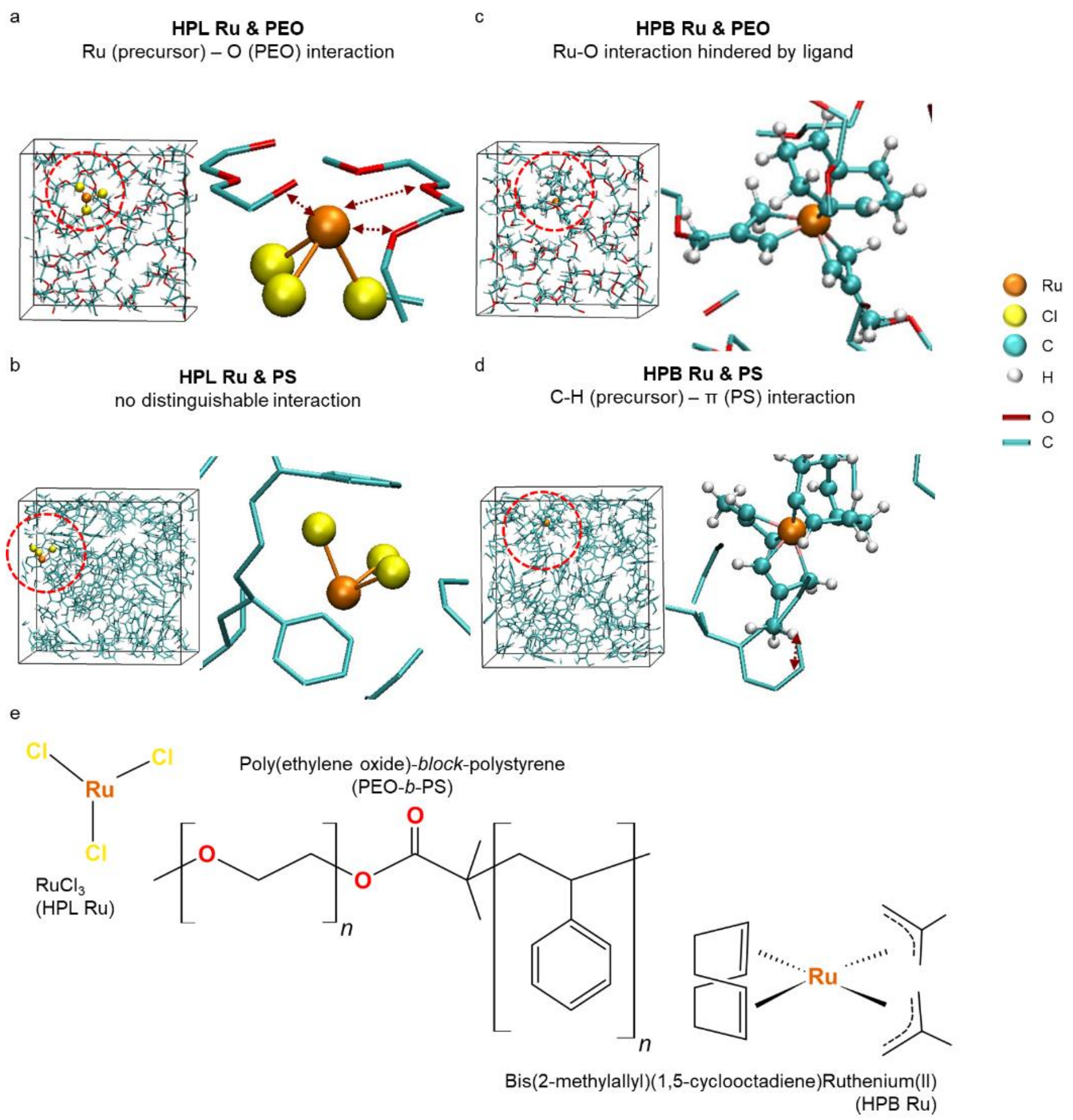

Supplementary Fig. 2 | MIs in different BCP-Ru precursor systems. a-d, Snapshots of equilibrated (**a**) PEO-HPL Ru, (**b**) PS-HPL Ru, (**c**) PEO-HPB Ru, and (**d**) PS-HPB Ru systems from molecular dynamic (MD) simulations (cyan sticks: CH_x-CH_x bond, red sticks: C-O bond, orange balls: Ru, yellow balls: Cl, cyan balls: carbon, gray balls: H, red arrows: distinguishable MIs). **e,** Molecular structures of HPL Ru, PEO-*b*-PS, and HPB Ru.

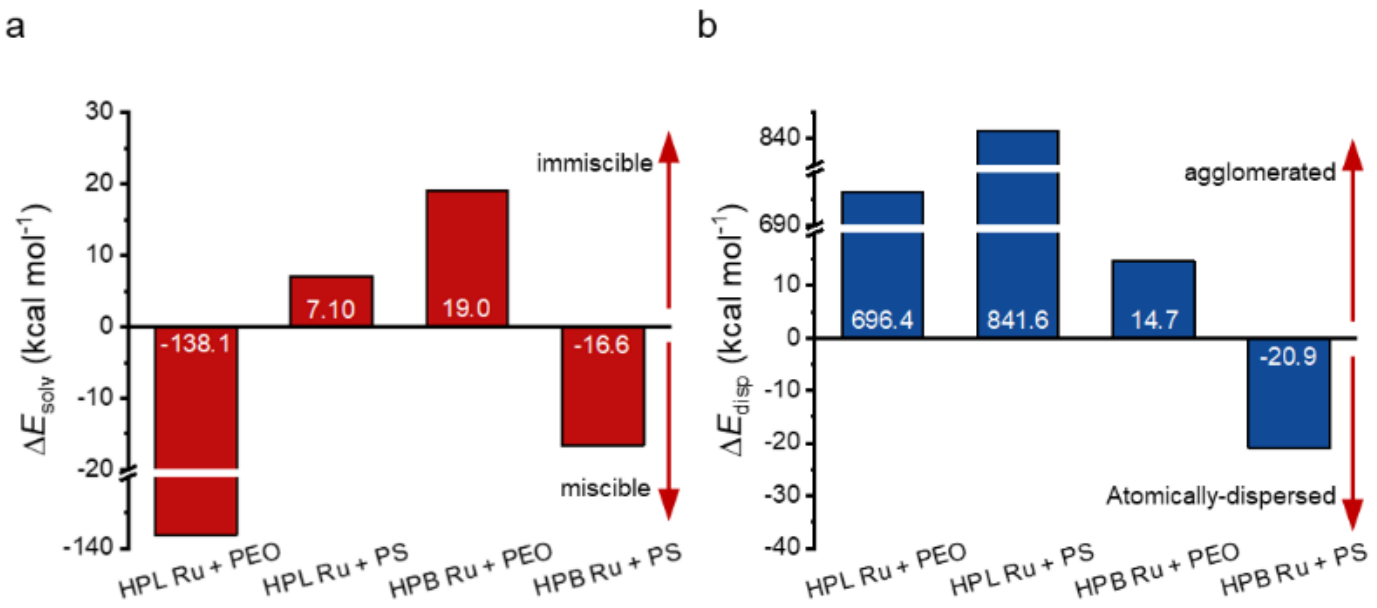

Supplementary Fig. 3 | Ru precursor positioning and dispersity in specific BCP microphases. a, The polymer solvation energy (ΔE_{solv}) and **b,** the dispersion propensity (ΔE_{disp}) for PEO-HPL Ru, PS-HPL Ru, PEO-HPB Ru, and PS-HPB Ru systems as obtained from MD simulations.

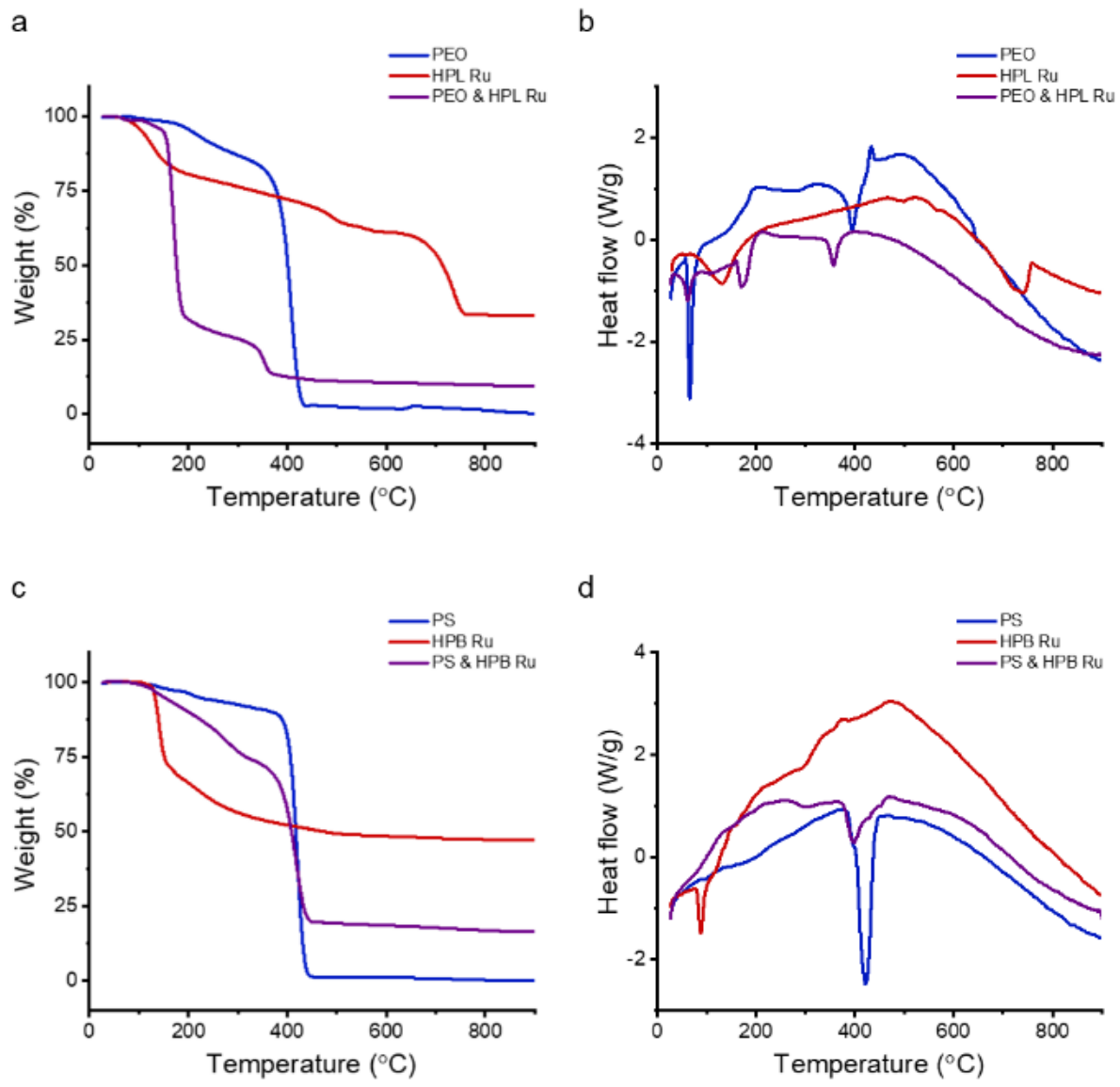

Supplementary Fig. 4 | TG-DSC analyses of polymer-precursor interactions. a,b, Thermogravimetric (TG) and differential scanning calorimetry (DSC) profiles of PEO, HPL Ru precursor, and solvent-cast PEO/HPL Ru composite. **c,d,** TG and DSC profiles of PS, HPB Ru precursor, and solvent-cast PS/HPB Ru composite.

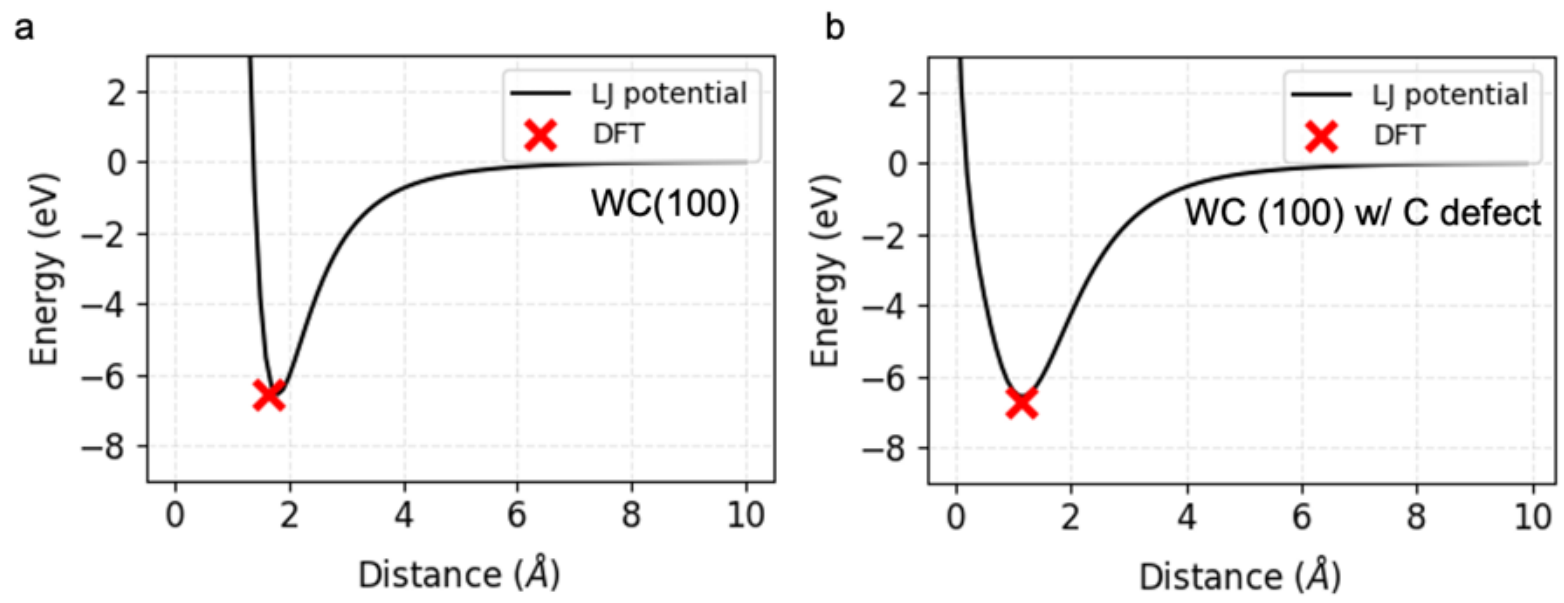

Supplementary Fig. 5 | Binding energy profiles. a,b, Fitted binding energy profile compared with the density functional theory (DFT)-calculated energy minima for (**a**) WC (100) and (**b**) WC (100) with C defect. The x-axis represents the distance of the Ru atom from the top layer of the slab in the z direction.

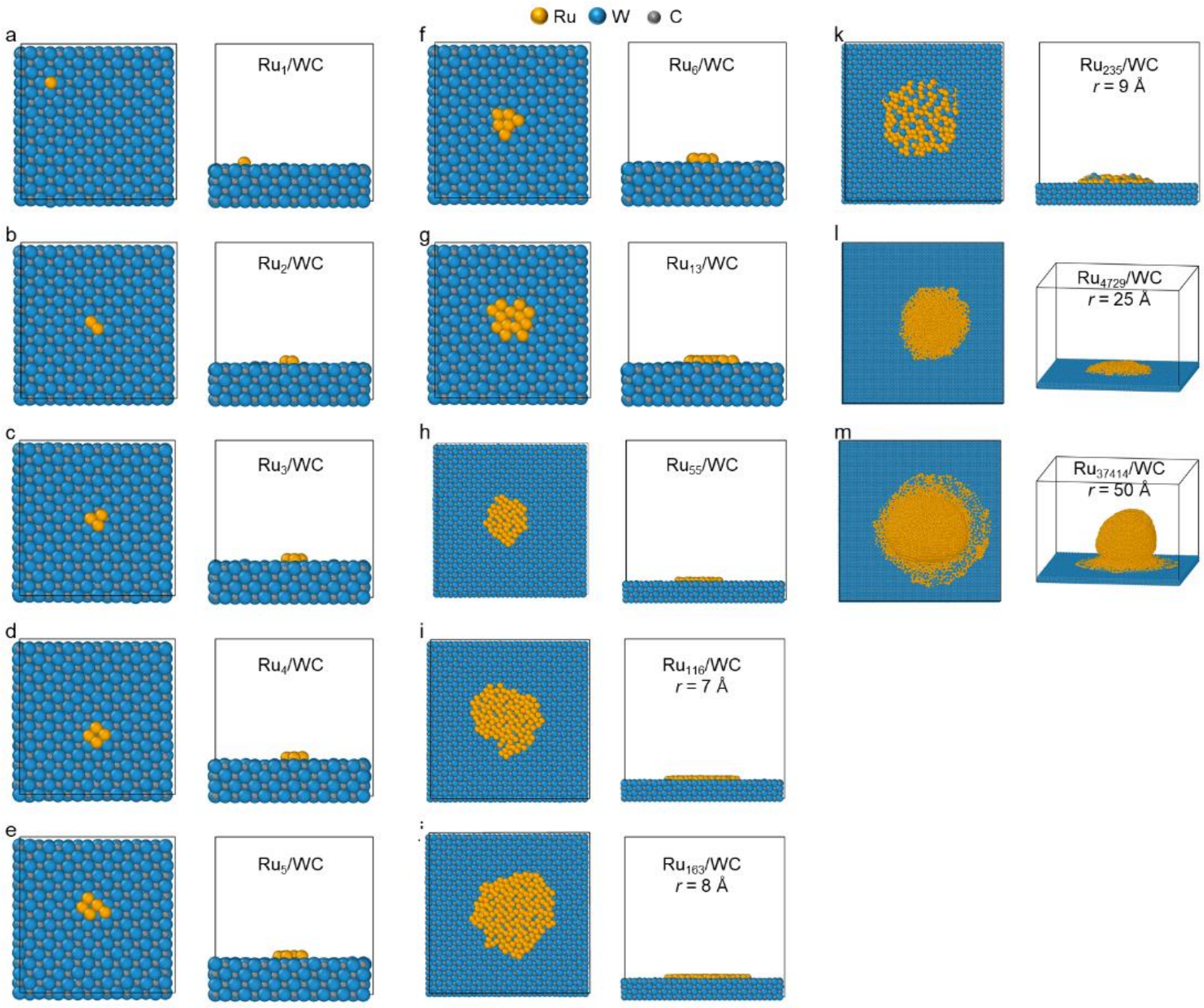

Supplementary Fig. 6 | Structural variation of Ru species on WC supports. a-m, Snapshots of the MD simulations at 1,200 K showing both the top and side views for (**a**) Ru_1/WC, (**b**) Ru_2/WC, (**c**) Ru_3/WC, (**d**) Ru_4/WC, (**e**) Ru_5/WC, (**f**) Ru_6/WC, (**g**) Ru_{13}/WC, (**h**) Ru_{55}/WC, (**i**) Ru_{116}/WC (particle radius r = 7 Å), (**j**) Ru_{163}/WC (r = 8 Å), (**k**) Ru_{235}/WC (r = 9 Å), (**l**) Ru_{4729}/WC (r = 25 Å), and (**m**) Ru_{37414}/WC (r = 50 Å) (orange: Ru, blue: W, gray: C).

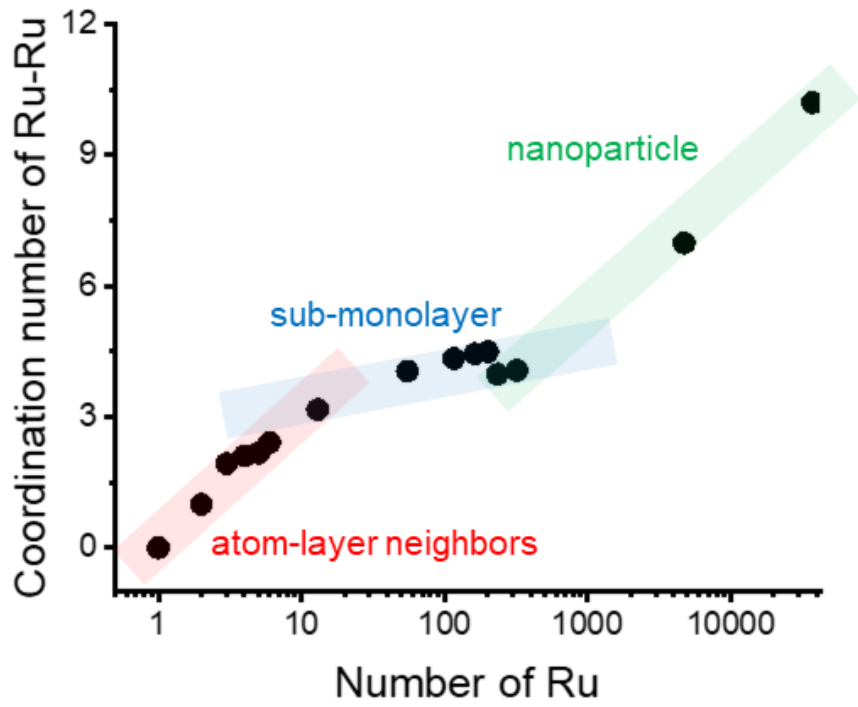

Supplementary Fig. 7 | Alteration of geometric structure. Coordination number (CN) of Ru-Ru bonds as a function of the number of Ru atoms.

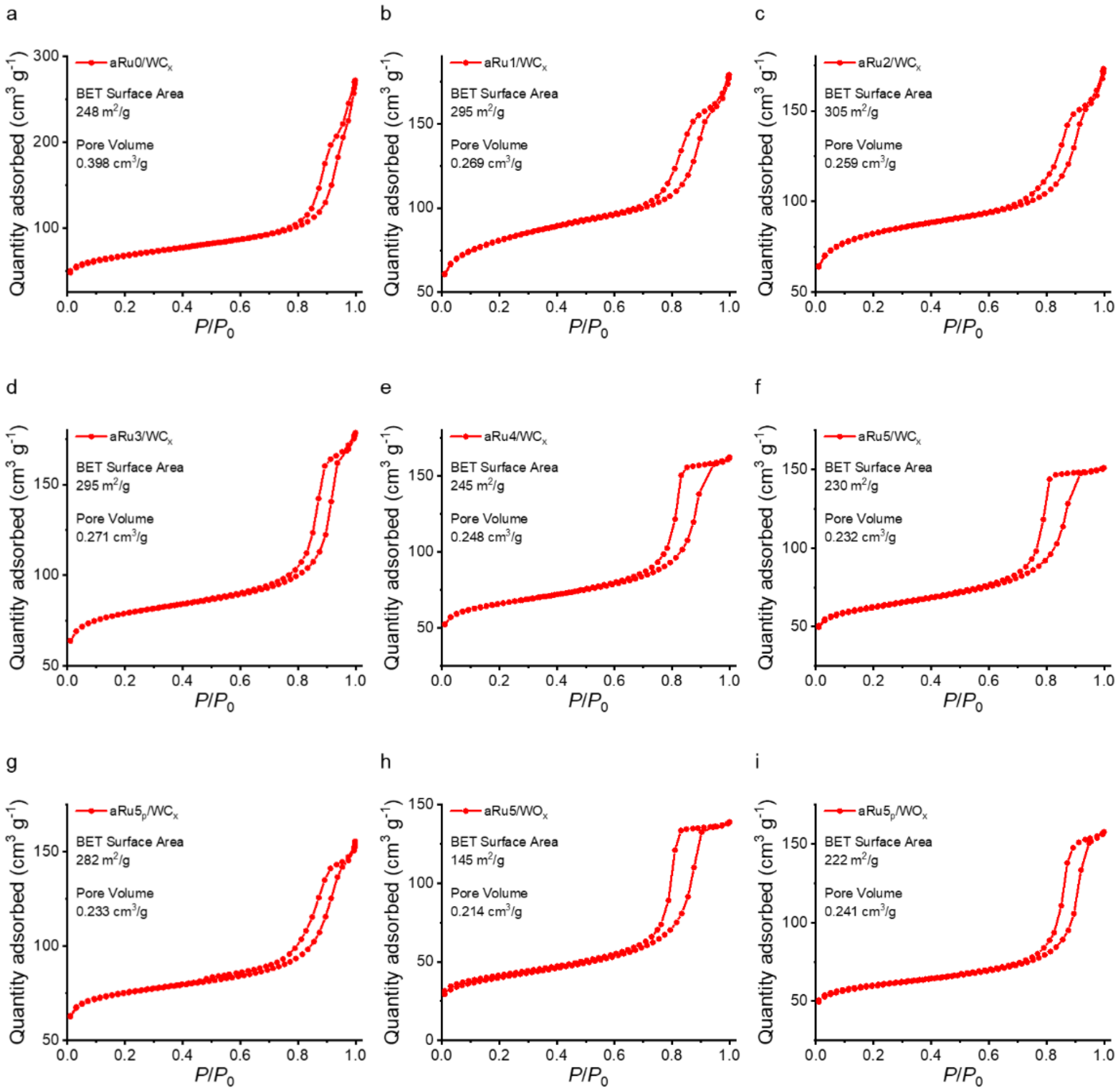

Supplementary Fig. 8 | N_2 physisorption. a-i, N_2 adsorption-desorption isotherms for samples prepared by self-assembly: (**a**) aRu0/WC_x, (**b**) aRu1/WC_x, (**c**) aRu2/WC_x, (**d**) aRu3/WC_x, (**e**) aRu4/WC_x, (**f**) aRu5/WC_x, (**g**) aRu5_p/WC_x, (**h**) aRu5/WO_x, and (**i**) aRu5_p/WO_x. The corresponding BET surface area and pore volume are provided.

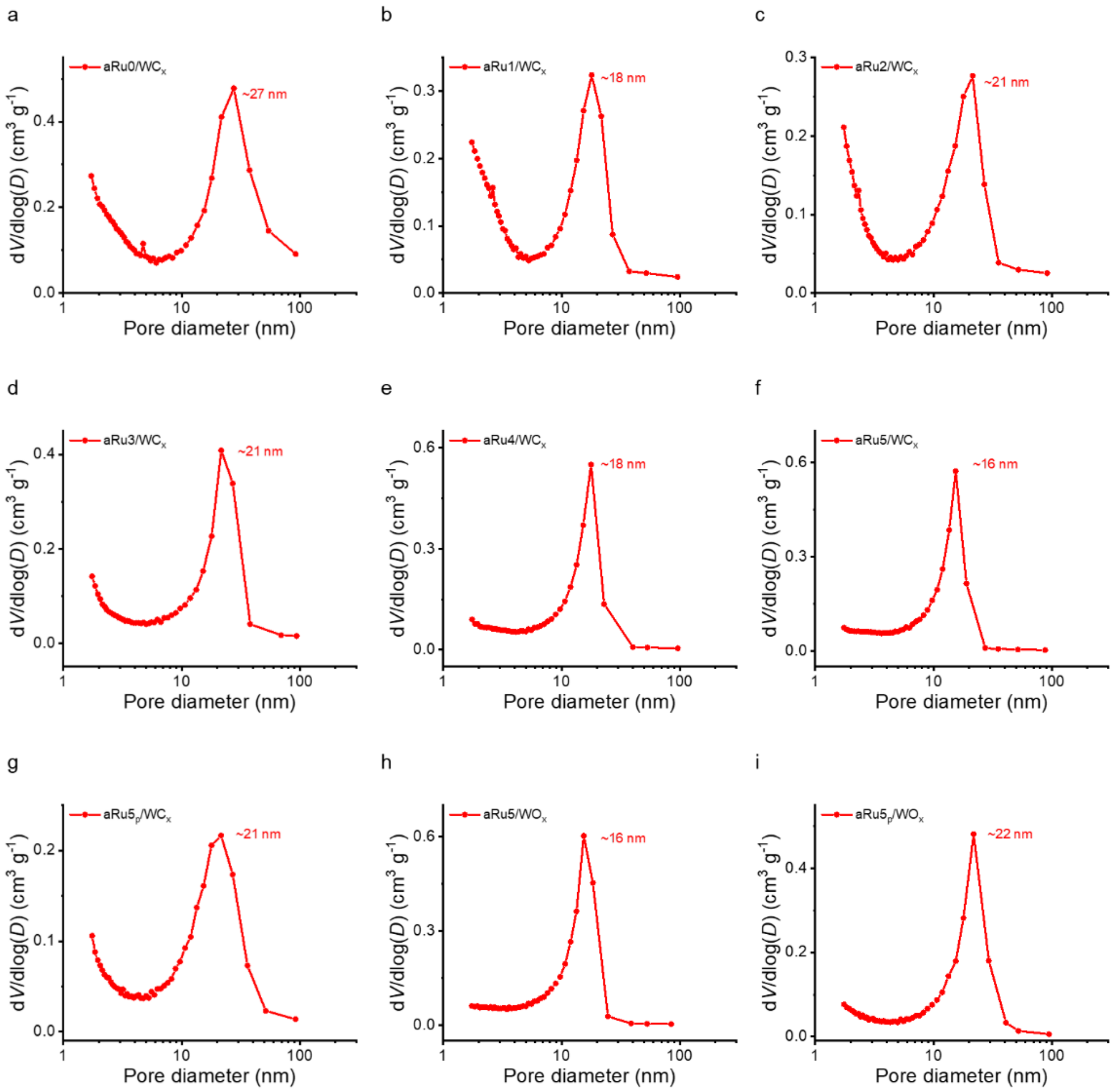

Supplementary Fig. 9 | Pore size distribution. a-i, Pore size distribution of samples prepared by self-assembly, as determined from N_2 physisorption: (**a**) aRu0/WC_x, (**b**) aRu1/WC_x, (**c**) aRu2/WC_x, (**d**) aRu3/WC_x, (**e**) aRu4/WC_x, (**f**) aRu5/WC_x, (**g**) $aRu5_p$/WC_x, (**h**) aRu5/WO_x, and (**i**) $aRu5_p$/WO_x.

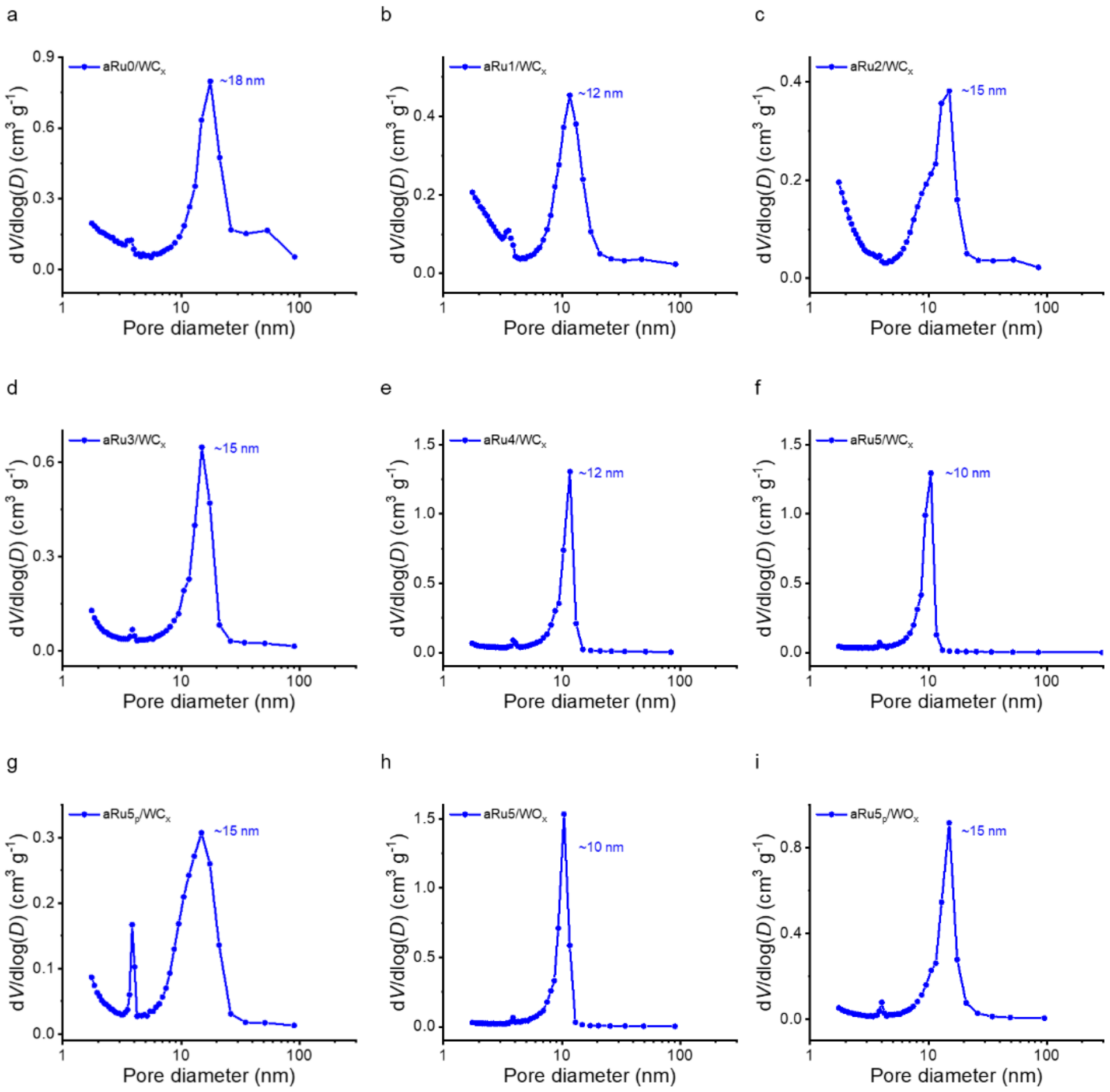

Supplementary Fig. 10 | Window size distribution. a-i, Window size distributions of samples prepared by self-assembly, as determined from N_2 physisorption: (**a**) aRu0/WC_x, (**b**) aRu1/WC_x, (**c**) aRu2/WC_x, (**d**) aRu3/WC_x, (**e**) aRu4/WC_x, (**f**) aRu5/WC_x, (**g**) aRu5_p/WC_x, (**h**) aRu5/WO_x, and (**i**) aRu5p/WO_x.

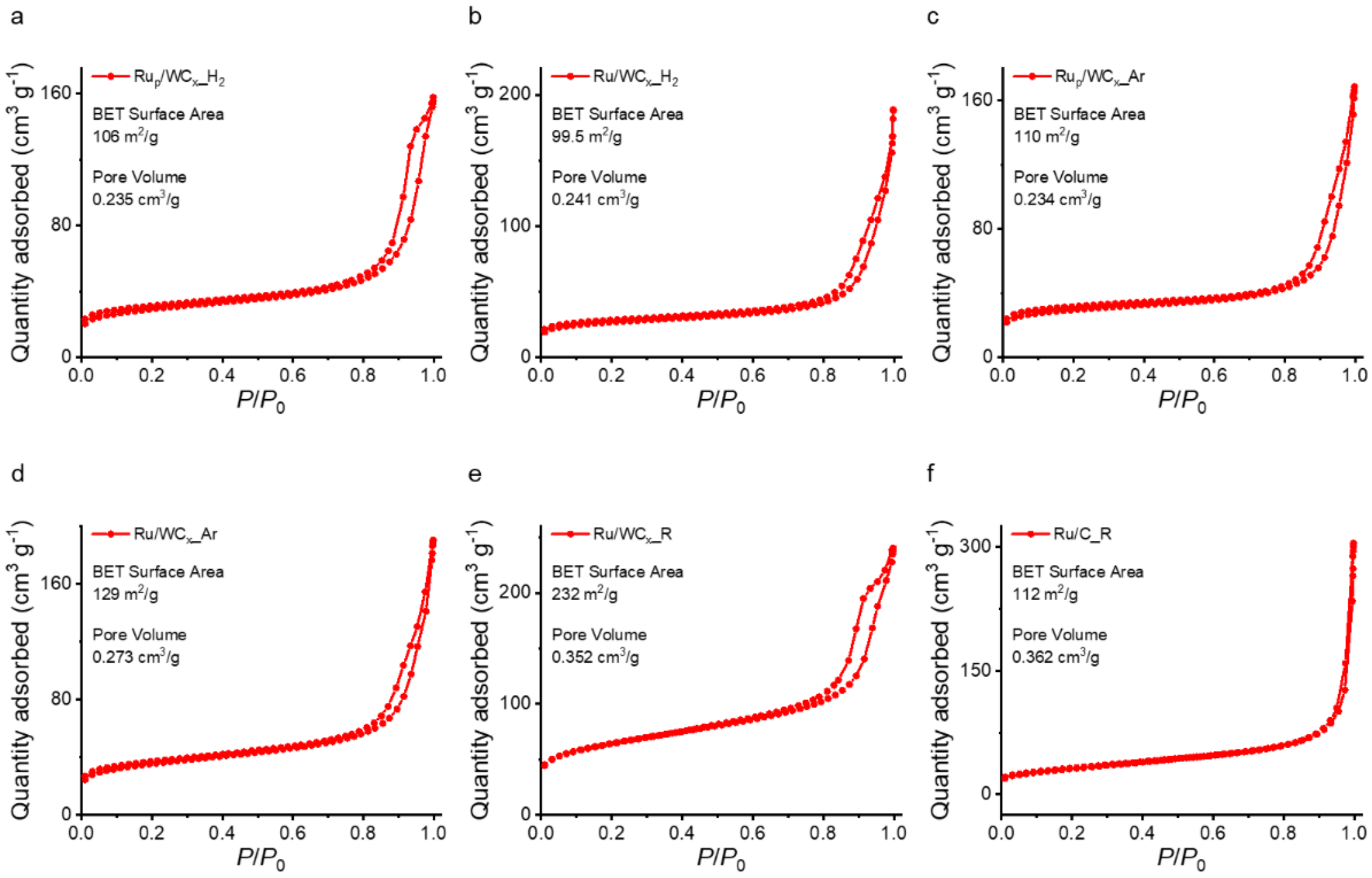

Supplementary Fig. 11 | N_2 physisorption analysis of comparison samples. a-f, N_2 adsorption-desorption isotherms of comparison samples: (**a**) Ru_p/WC_x_H_2, (**b**) Ru/WC_x_H_2, (**c**) Ru_p/WC_x_Ar, (**d**) Ru/WC_x_Ar, (**e**) Ru/WC_x_R, and (**f**) Ru/C_R. The corresponding BET surface area and pore volume are provided.

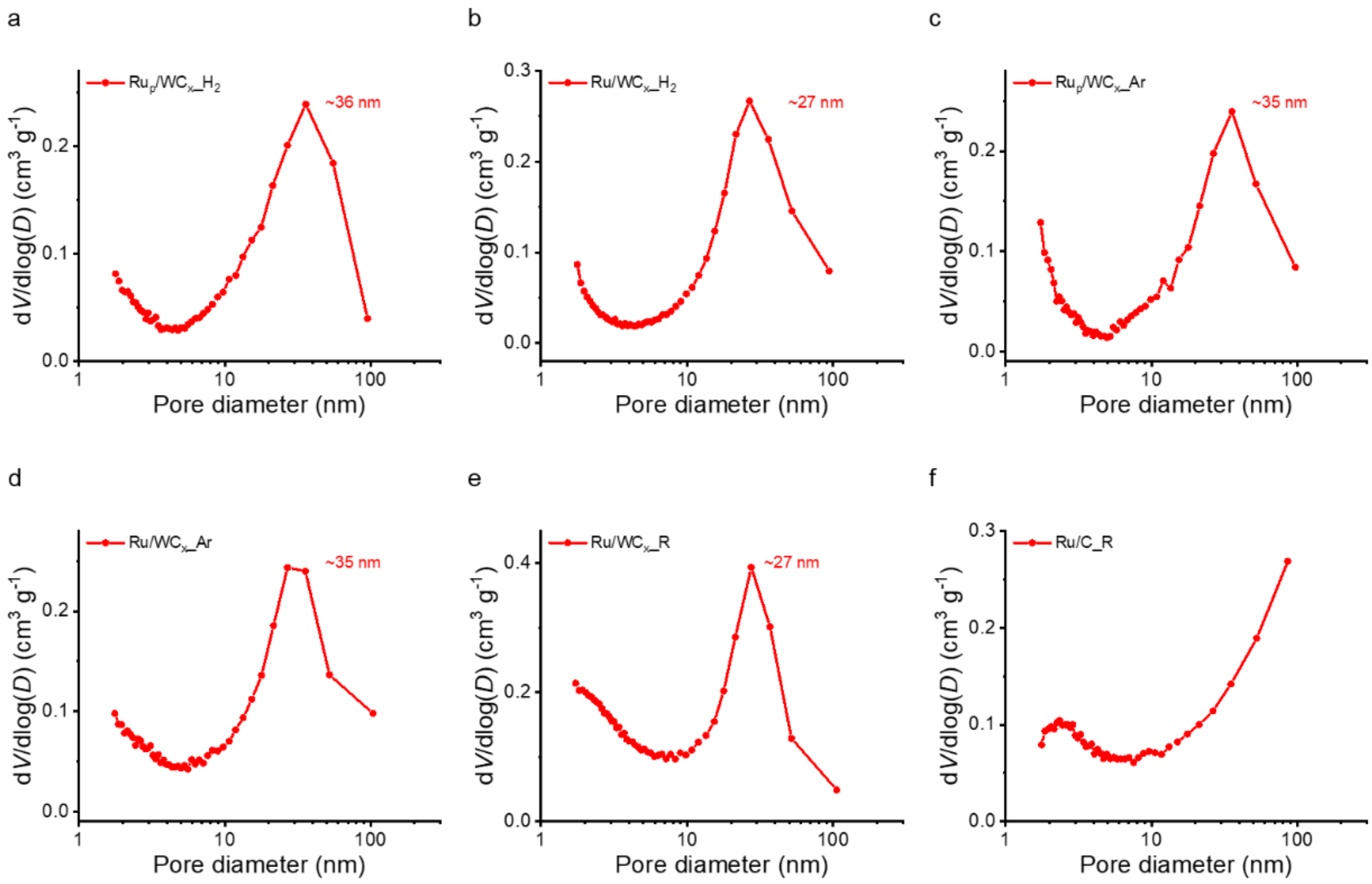

Supplementary Fig. 12 | Pore size distribution analysis of comparison samples. a-f, Pore size distributions of comparison samples, as determined from N_2 physisorption: (**a**) Ru_p/WC_x_H_2, (**b**) Ru/WC_x_H_2, (**c**) Ru_p/WC_x_Ar, (**d**) Ru/WC_x_Ar, (**e**) Ru/WC_x_R, and (**f**) Ru/C_R**.**

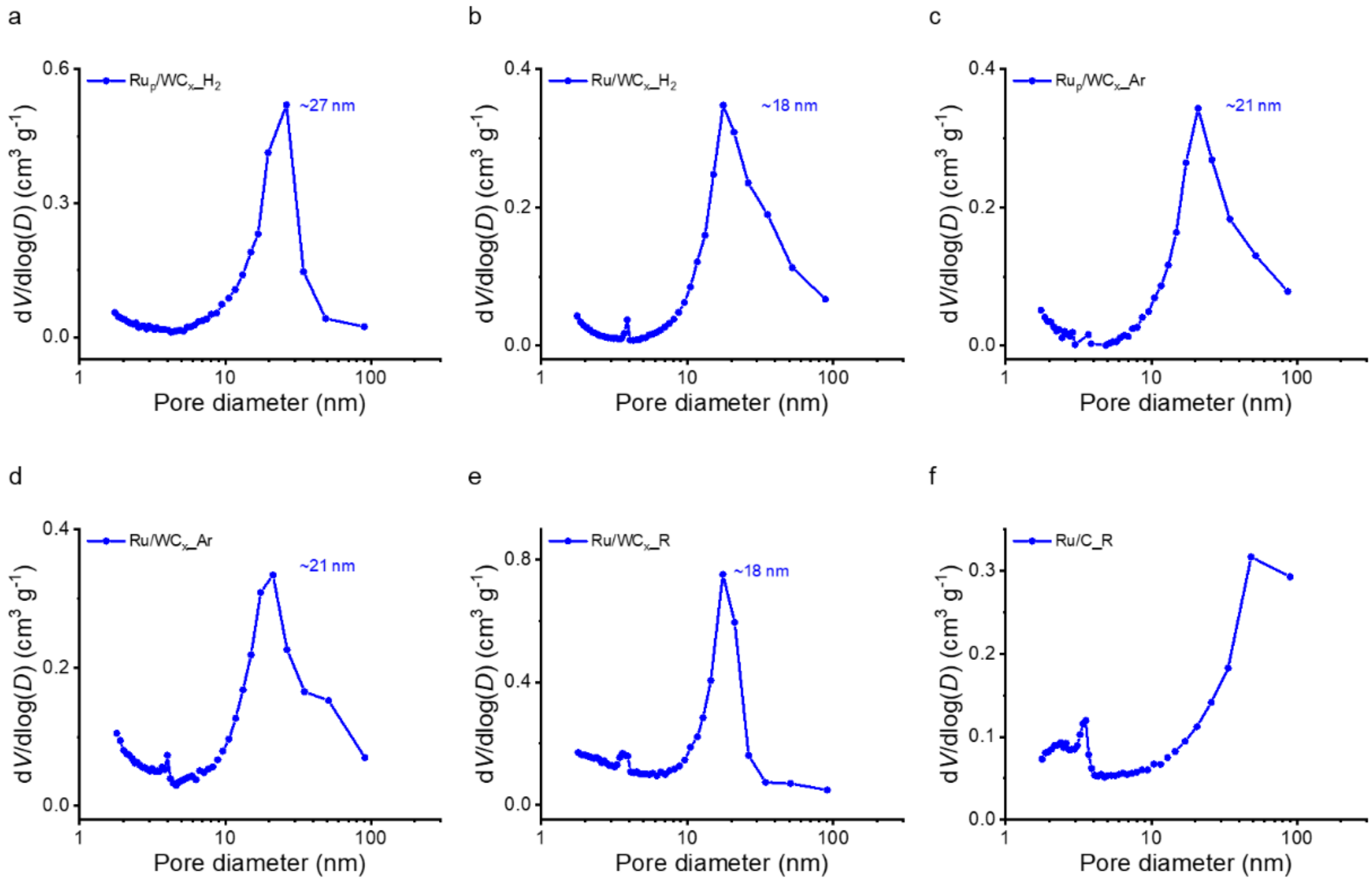

Supplementary Fig. 13 | Window size distribution analysis of comparison samples. a-f, Window size distributions of comparison samples, as determined from N_2 physisorption: (**a**) Ru$_p$/WC$_x$_H$_2$, (**b**) Ru/WC$_x$_H$_2$, (**c**) Ru$_p$/WC$_x$_Ar, (**d**) Ru/WC$_x$_Ar, (**e**) Ru/WC$_x$_R, and (**f**) Ru/C_R.

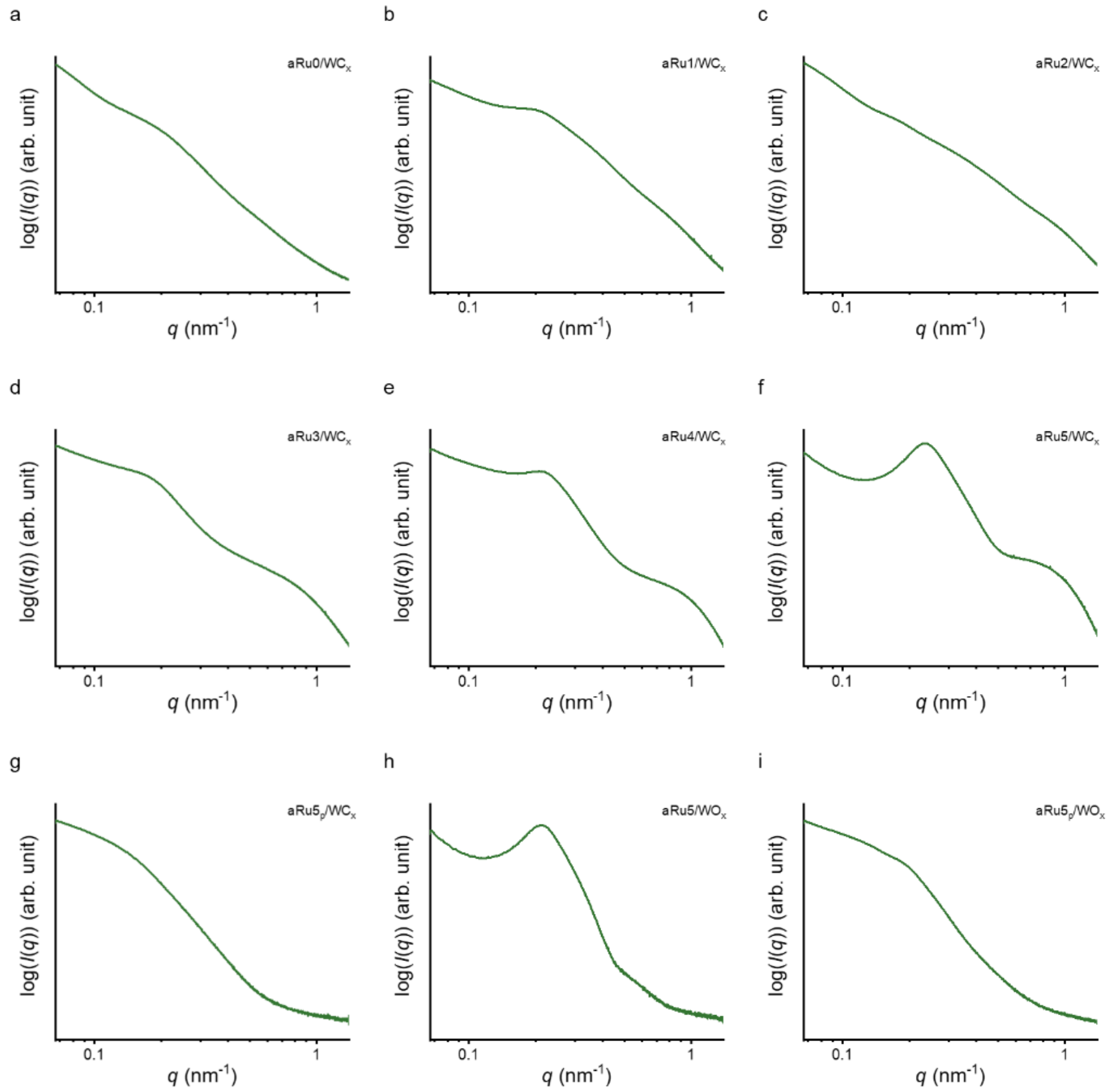

Supplementary Fig. 14 | SAXS profiles. a-i, SAXS intensity profiles (log $I(q)$ vs. q) of samples prepared via self-assembly: (**a**) aRu0/WC_x, (**b**) aRu1/WC_x, (**c**) aRu2/WC_x, (**d**) aRu3/WC_x, (**e**) aRu4/WC_x, (**f**) aRu5/WC_x, (**g**) $aRu5_p/WC_x$, (**h**) aRu5/WO_x, and (**i**) aRu5p/WO_x**.** The position of the broad scattering maximum (or shoulder) is indicated by line markers.

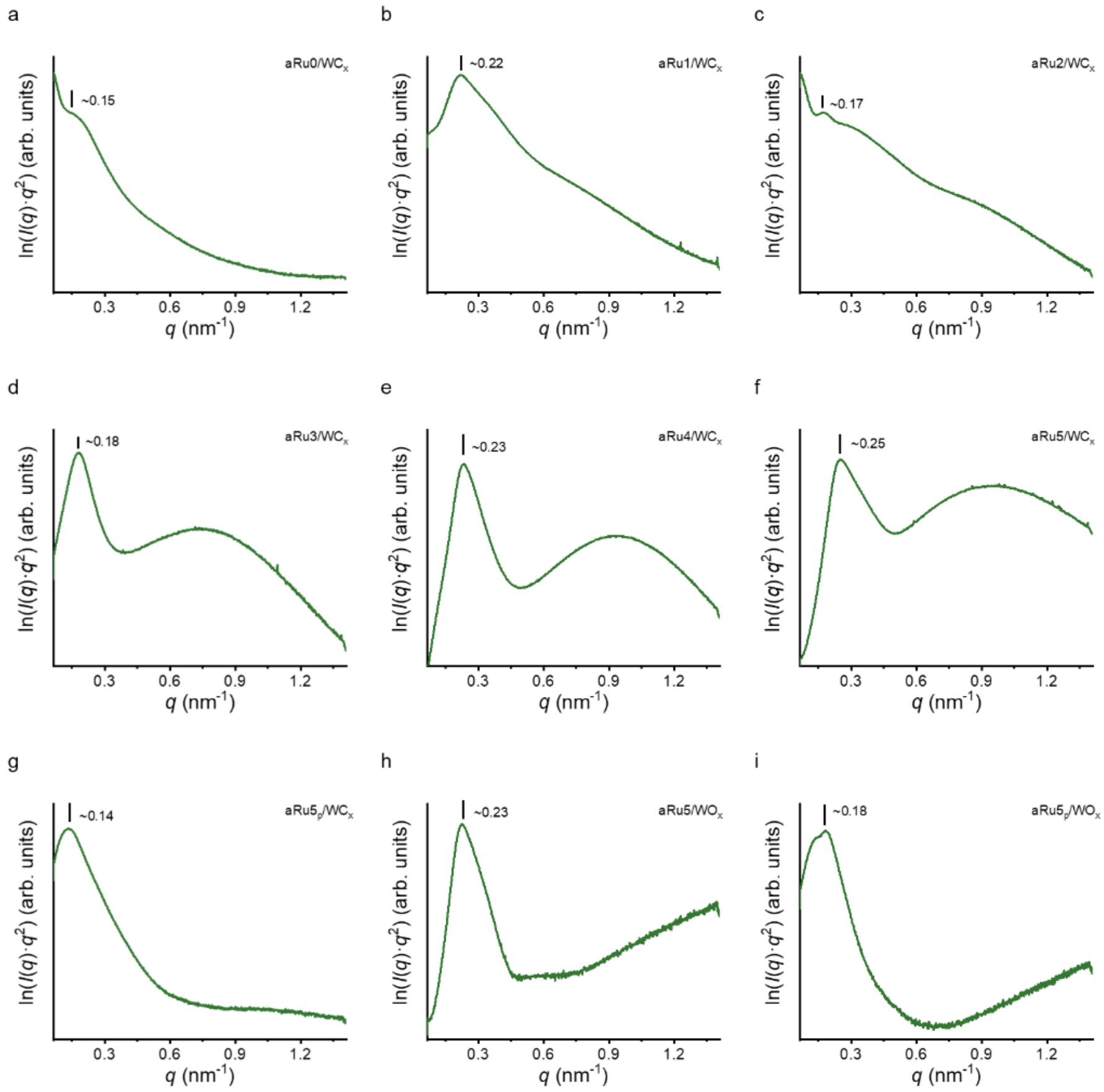

Supplementary Fig. 15 | SAXS analysis. a-i, Kratky plots ($I(q) \cdot q^2$ vs. q) of SAXS data for samples prepared via self-assembly: (**a**) aRu0/WC_x, (**b**) aRu1/WC_x, (**c**) aRu2/WC_x, (**d**) aRu3/WC_x, (**e**) aRu4/WC_x, (**f**) aRu5/WC_x, (**g**) $aRu5_p/WC_x$, (**h**) aRu5/WO_x, and (**i**) aRu5p/WO_x**.** The position of the broad scattering maximum (or shoulder) is indicated by line markers. *q* denotes the magnitude of scattering vector.

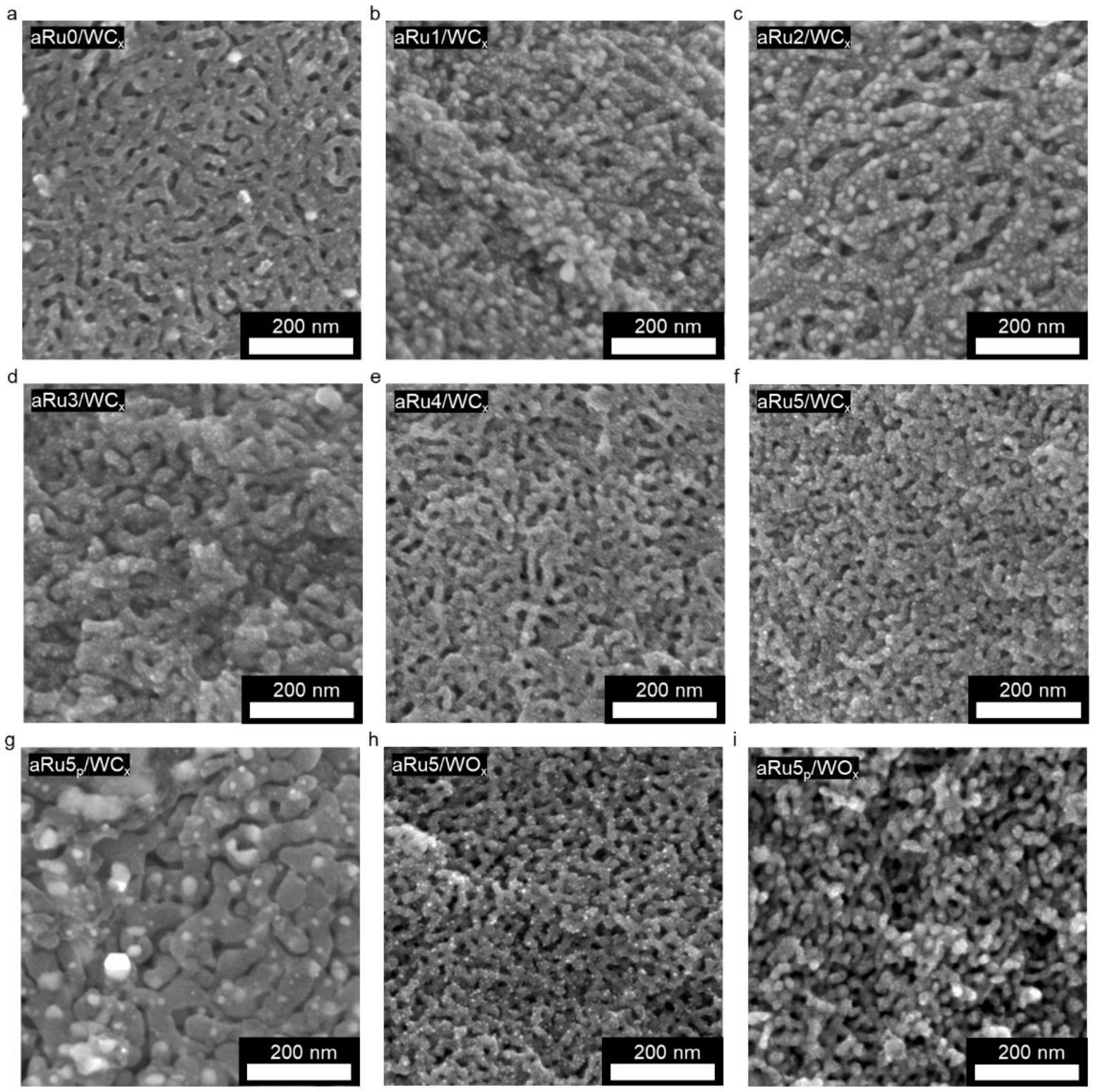

Supplementary Fig. 16 | Scanning electron microscopy (SEM) images. a-i, SEM images of samples prepared by self-assembly: (**a**) aRu0/WC$_x$, (**b**) aRu1/WC$_x$, (**c**) aRu2/WC$_x$, (**d**) aRu3/WC$_x$, (**e**) aRu4/WC$_x$, (**f**) aRu5/WC$_x$, (**g**) aRu5$_p$/WC$_x$, (**h**) aRu5/WO$_x$, and (**i**) aRu5p/WO$_x$.

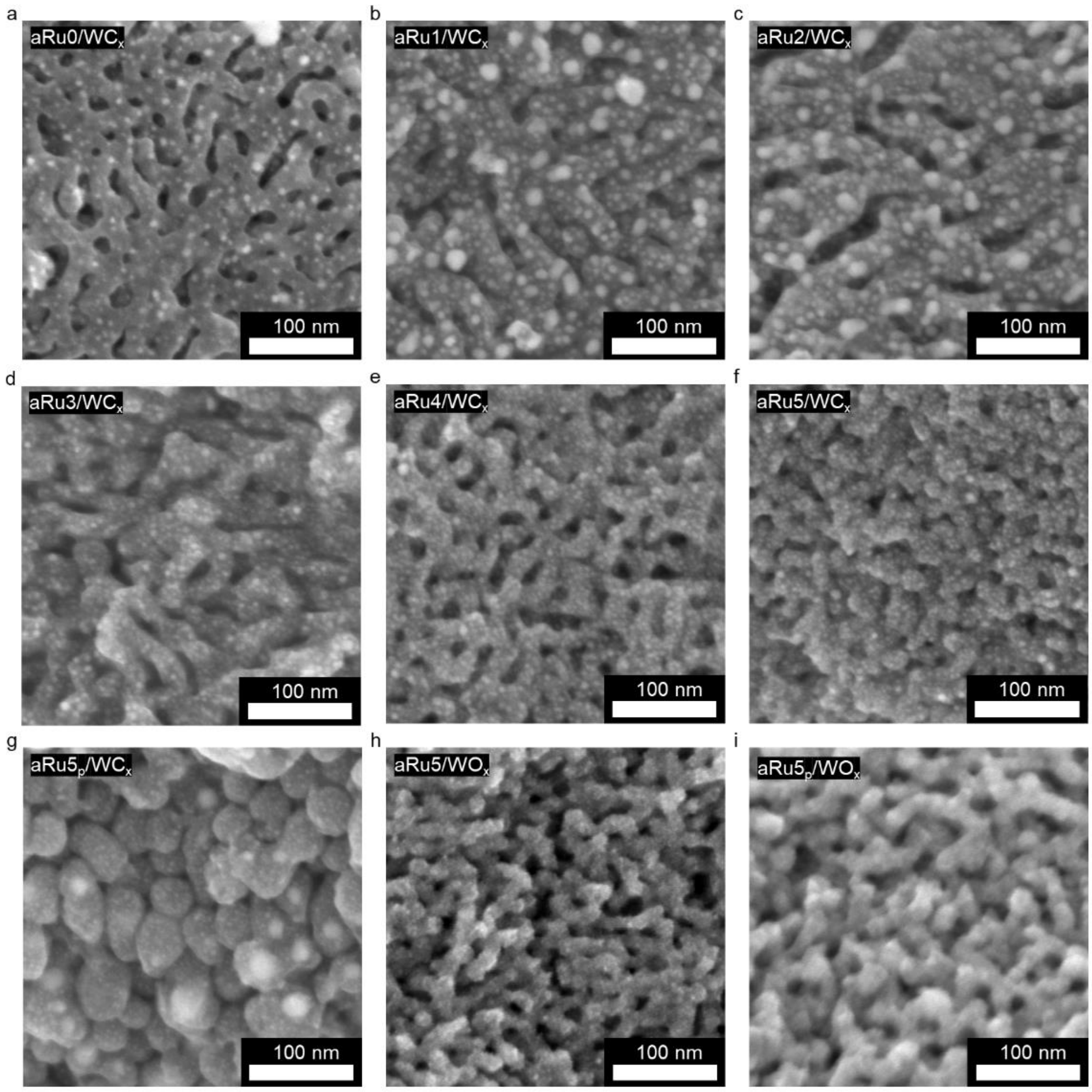

Supplementary Fig. 17 | Scanning electron microscopy (SEM) images. a-i, Enlarged SEM images of samples prepared by self-assembly: (**a**) aRu0/WC_x, (**b**) aRu1/WC_x, (**c**) aRu2/WC_x, (**d**) aRu3/WC_x, (**e**) aRu4/WC_x, (**f**) aRu5/WC_x, (**g**) $aRu5_p$/WC_x, (**h**) aRu5/WO_x, and (**i**) aRu5p/WO_x.

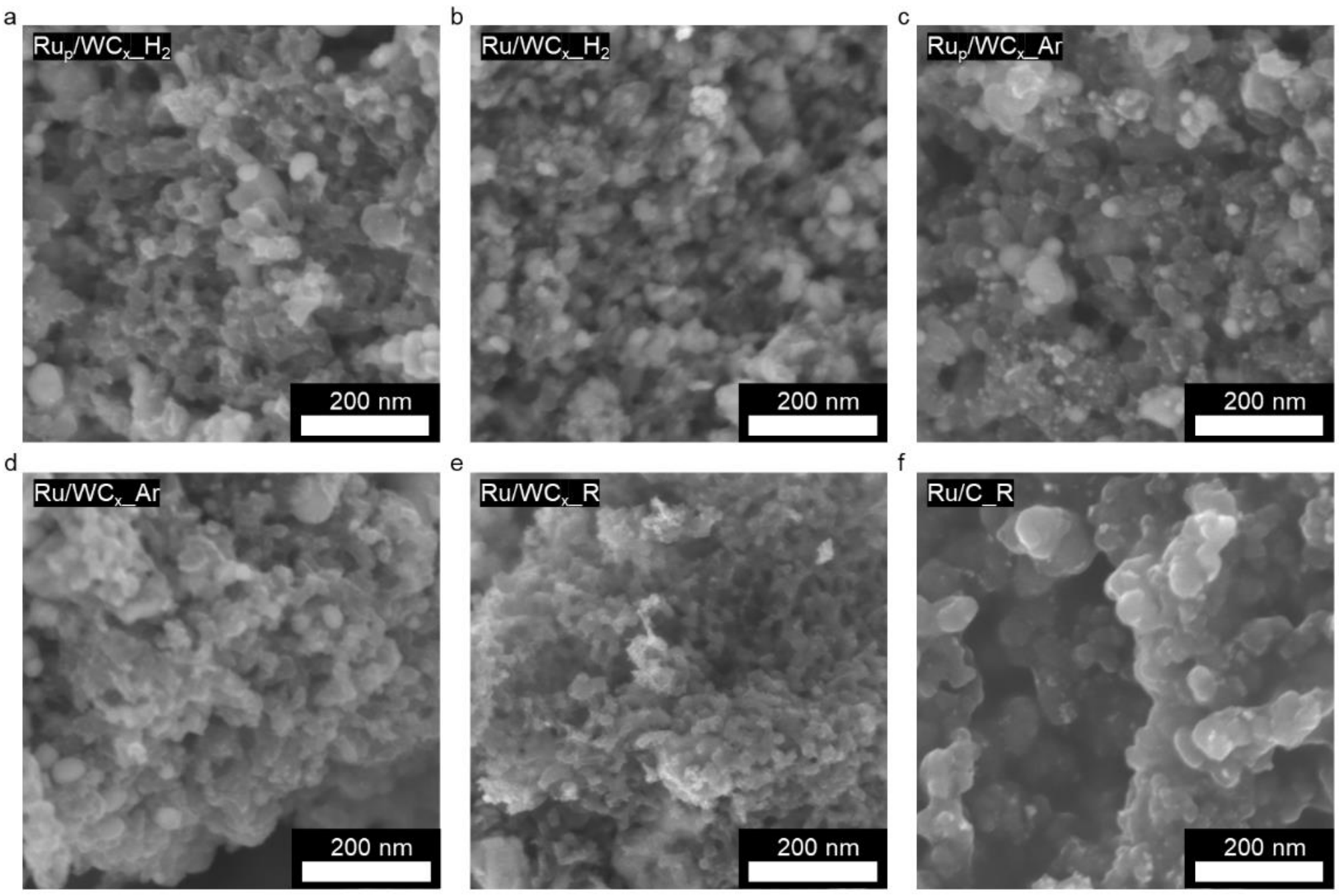

Supplementary Fig. 18 | Scanning electron microscopy (SEM) images. a-f, SEM images of comparison samples: (**a**) $Ru_p/WC_x\_H_2$, (**b**) $Ru/WC_x\_H_2$, (**c**) Ru_p/WC_x_Ar, (**d**) Ru/WC_x_Ar, (**e**) Ru/WC_x_R, and (**f**) Ru/C_R**.**

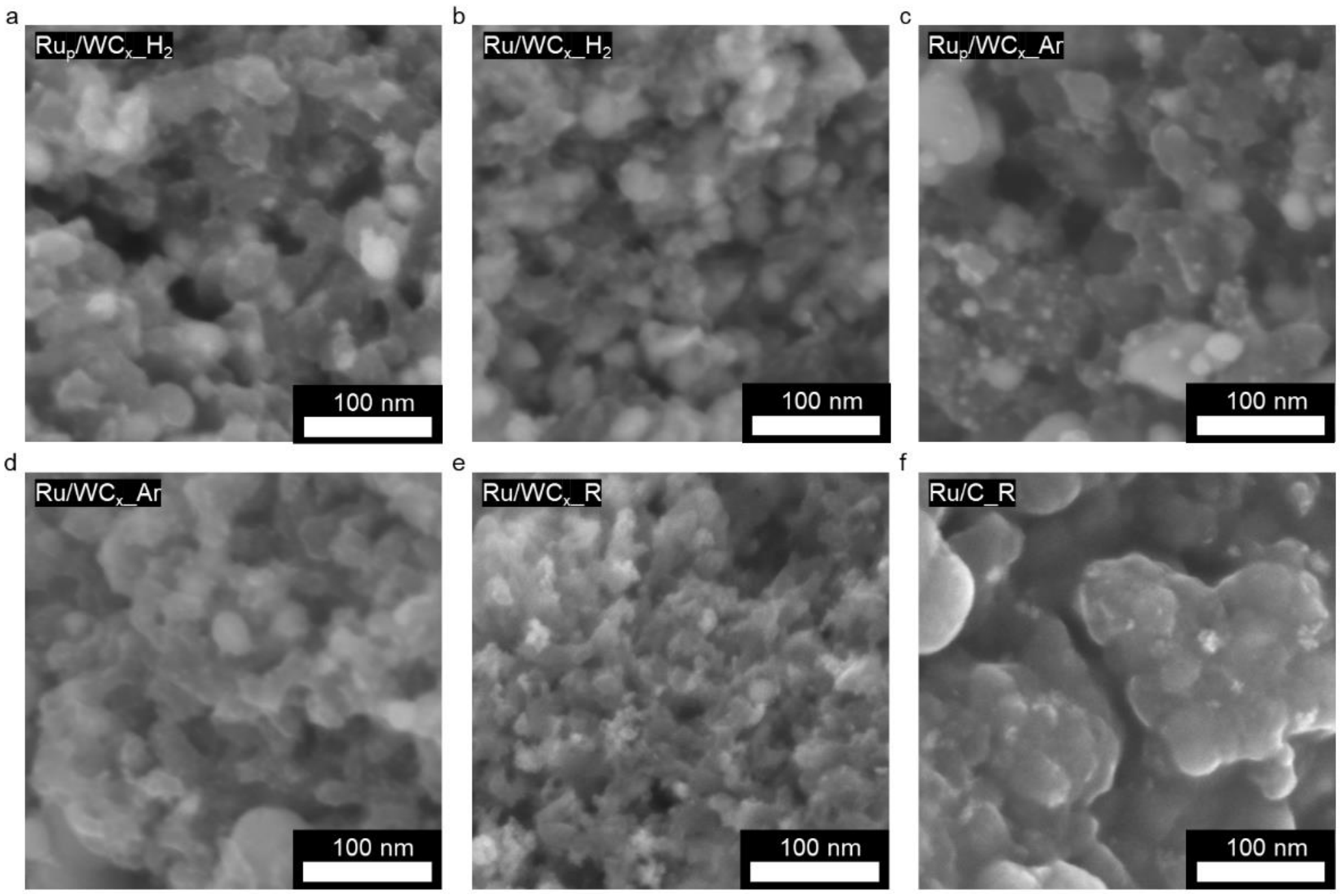

Supplementary Fig. 19 | Scanning electron microscopy (SEM) images. a-f, Enlarged SEM images of comparison samples: (**a**) $Ru_p/WC_x\_H_2$, (**b**) $Ru/WC_x\_H_2$, (**c**) Ru_p/WC_x_Ar, (**d**) Ru/WC_x_Ar, (**e**) Ru/WC_x_R, and (**f**) Ru/C_R**.**

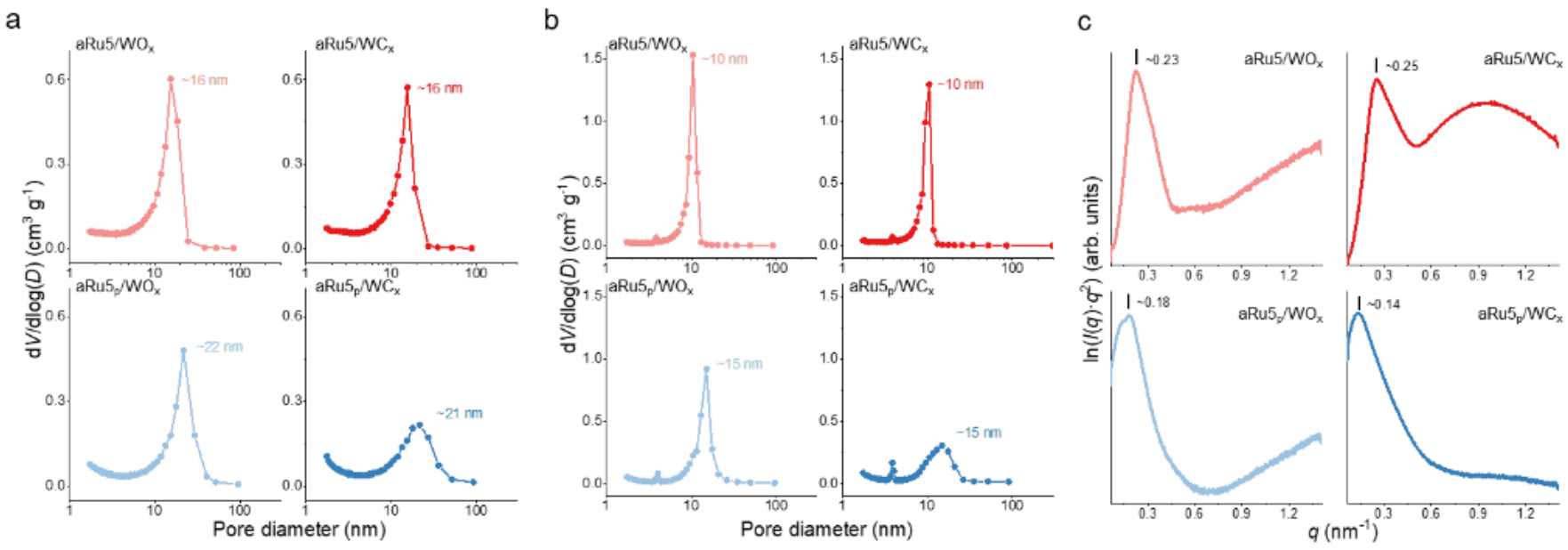

Supplementary Fig. 20 | Porosity alteration by different Ru precursors. a, Pore size distributions with the peak pore sizes labeled, **b,** window size distributions with the peak window sizes labeled, and **c,** Kratky plots of SAXS patterns (The position of the broad scattering maximum (or shoulder) is indicated by line markers). The samples were prepared using HPB Ru (aRu5/WO_x and aRu5/WC_x) and HPL Ru (aRu5_p/WO_x and aRu5_p/WC_x) at different annealing *T*. *q* denotes the magnitude of scattering vector.

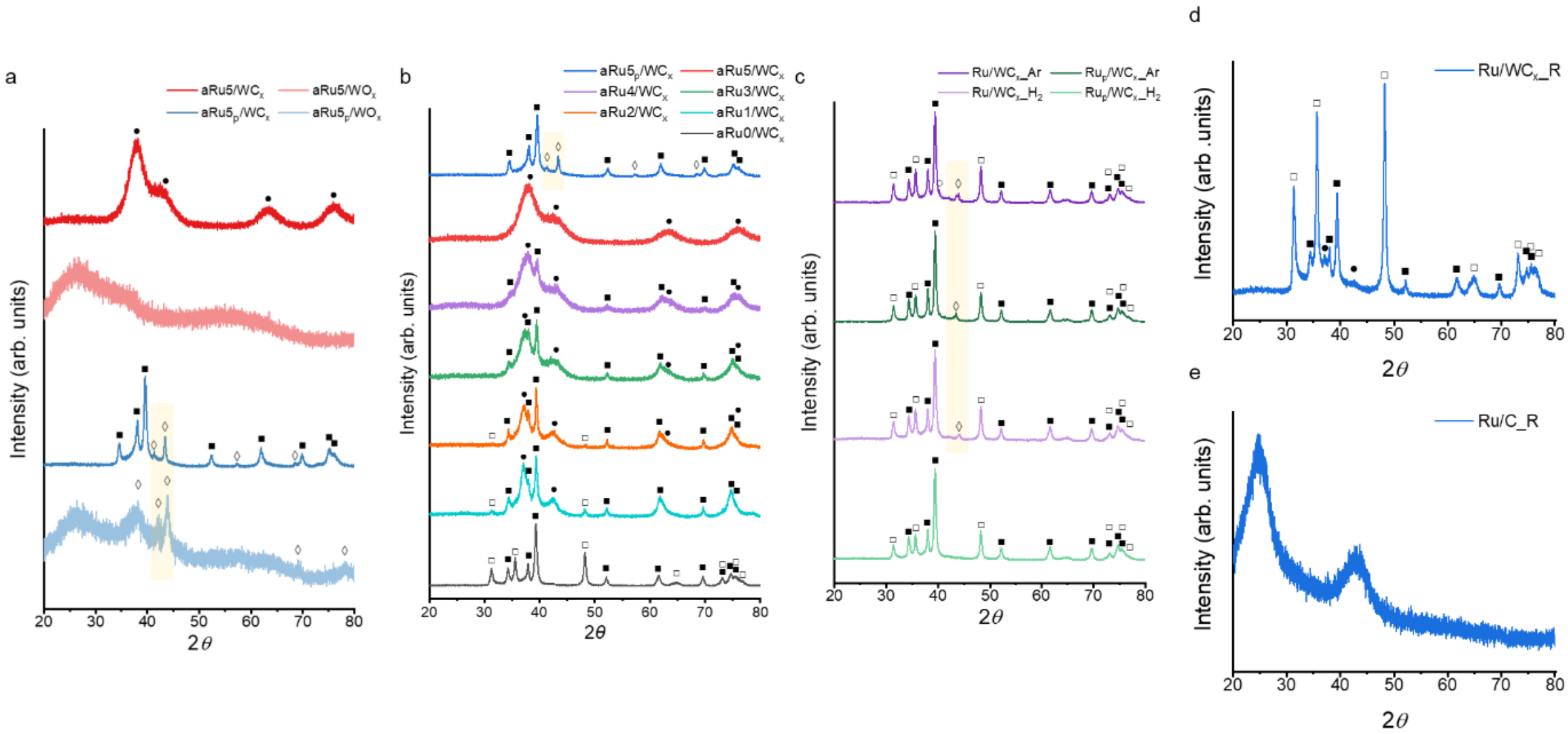

Supplementary Fig. 21 | XRD analyses of Ru samples. a-e, XRD patterns of (**a**) samples prepared using HPB Ru (aRu5/WO$_x$ and aRu5/WC$_x$) and HPL Ru (aRu5$_p$/WO$_x$ and aRu5$_p$/WC$_x$) at different annealing *T*, (**b**) aRu*N*/WC$_x$, aRu5$_p$/WC$_x$, (**c**) samples prepared by IWI using HPB Ru (Ru/WC$_x$_H$_2$ and Ru/WC$_x$_Ar) and HPL Ru (Rup/WC$_x$_H$_2$ and Ru$_p$/WC$_x$_Ar), and Ru samples prepared by chemical reduction on (**d**) WC$_x$-C nanocomposite and (**e**) carbon supports (●: γ-WC [PDF 20-1316], ■: α-W_2C [PDF 35-0776], □: δ-WC [PDF 25-1047], ◊: Ru [PDF 88-1734]). The pale-yellow shaded regions highlight the crystalline Ru peaks.

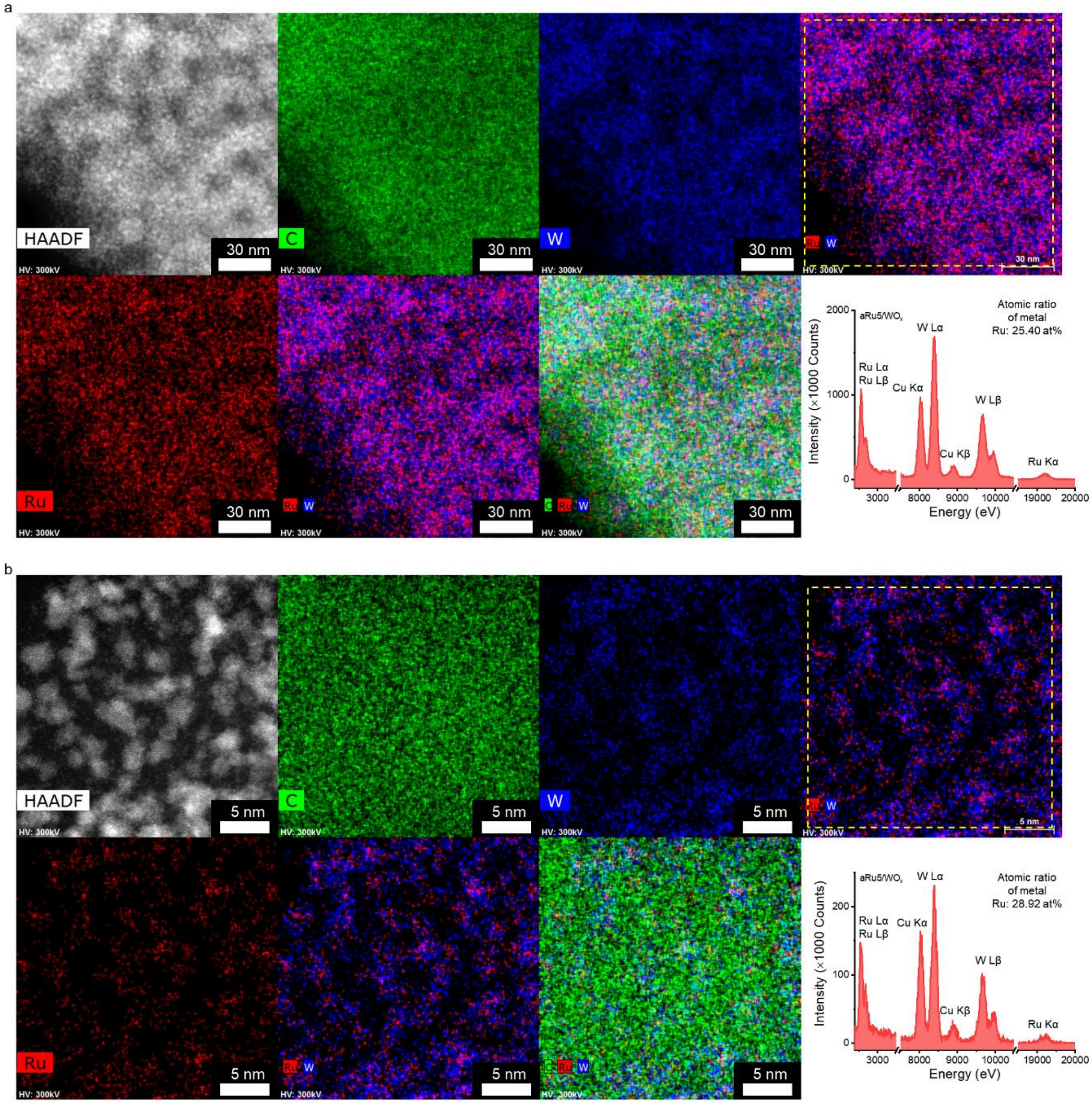

Supplementary Fig. 22 | STEM analysis of aRu5/WO$_x$. a, High-angle annular dark-field scanning transmission electron microscopy (HAADF-STEM) image and the corresponding energy-dispersive spectroscopy (EDS) elemental mappings for C (green), W (blue), Ru (red), along with the overlaid composite map and the EDS spectrum showing the atomic ratio of metals. **b,** Higher-magnification HAADF-STEM image and the corresponding EDS mappings for the same elements, their composite overlay, and the EDS spectrum showing the atomic ratio of metals.

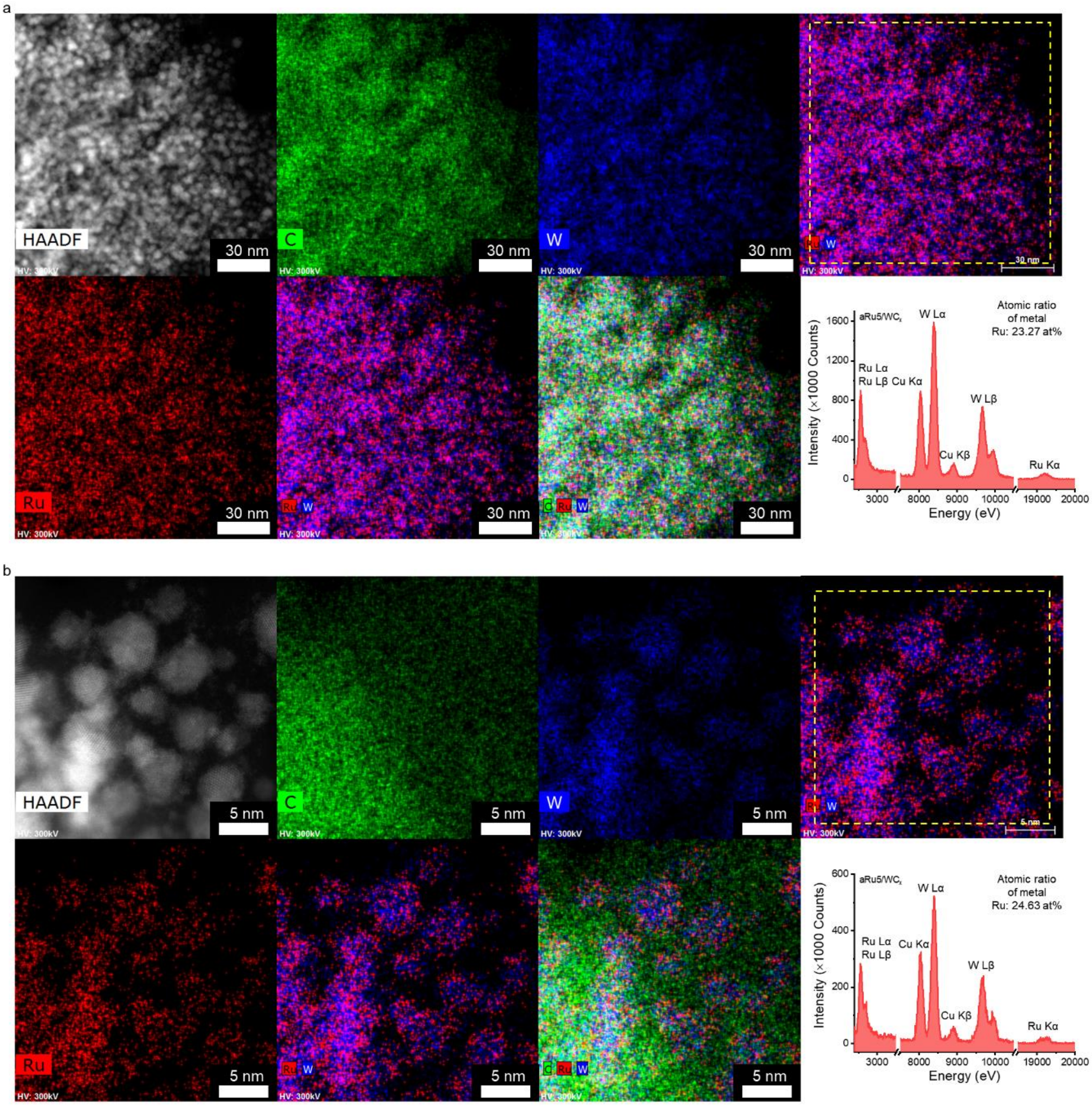

Supplementary Fig. 23 | STEM analysis of aRu5/WC$_x$. a, HAADF-STEM image and the corresponding EDS elemental mappings for C (green), W (blue), Ru (red), along with the overlaid composite map and the EDS spectrum showing the atomic ratio of metals. **b,** Higher-magnification HAADF-STEM image and the corresponding EDS mappings for the same elements, their composite overlay, and the EDS spectrum showing the atomic ratio of metals.

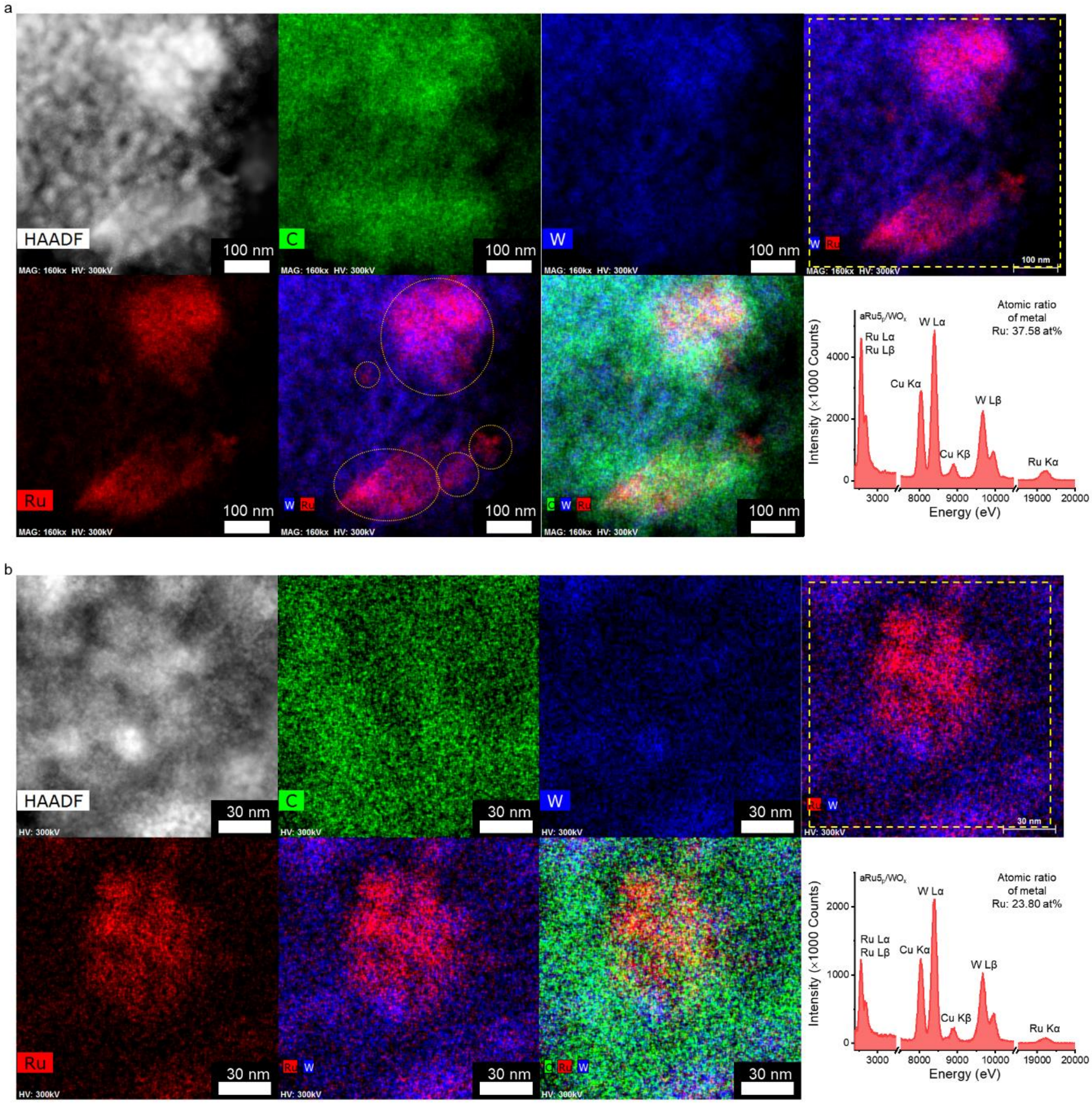

Supplementary Fig. 24 | STEM analysis of aRu5$_p$/WO$_x$. a, HAADF-STEM image and the corresponding EDS elemental mappings for C (green), W (blue), Ru (red), along with the overlaid composite map and the EDS spectrum showing the atomic ratio of metals (orange circles: Ru agglomerates). **b,** Higher-magnification HAADF-STEM image and the corresponding EDS mappings for the same elements, their composite overlay, and the EDS spectrum showing the atomic ratio of metals.

a

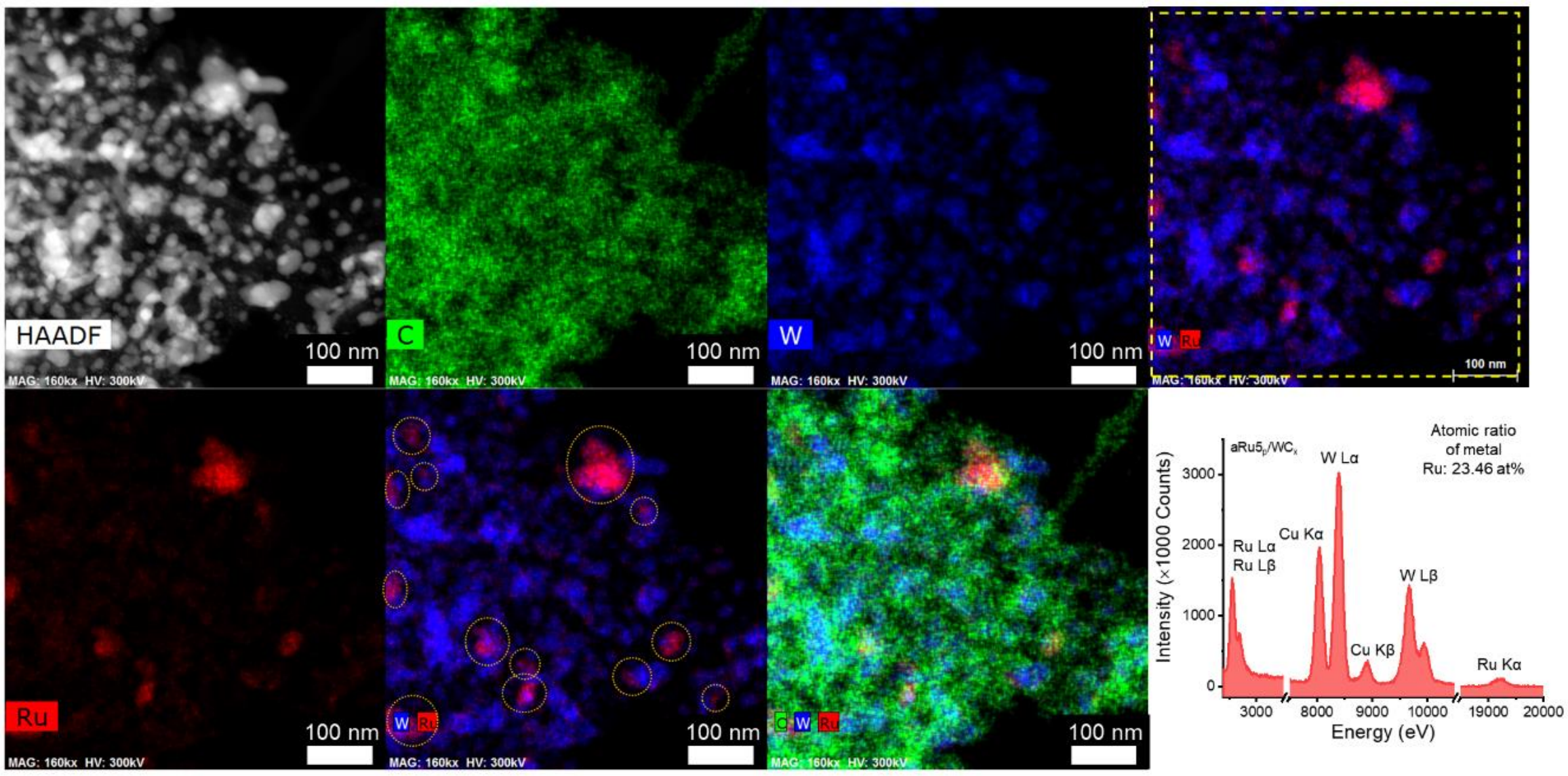

b

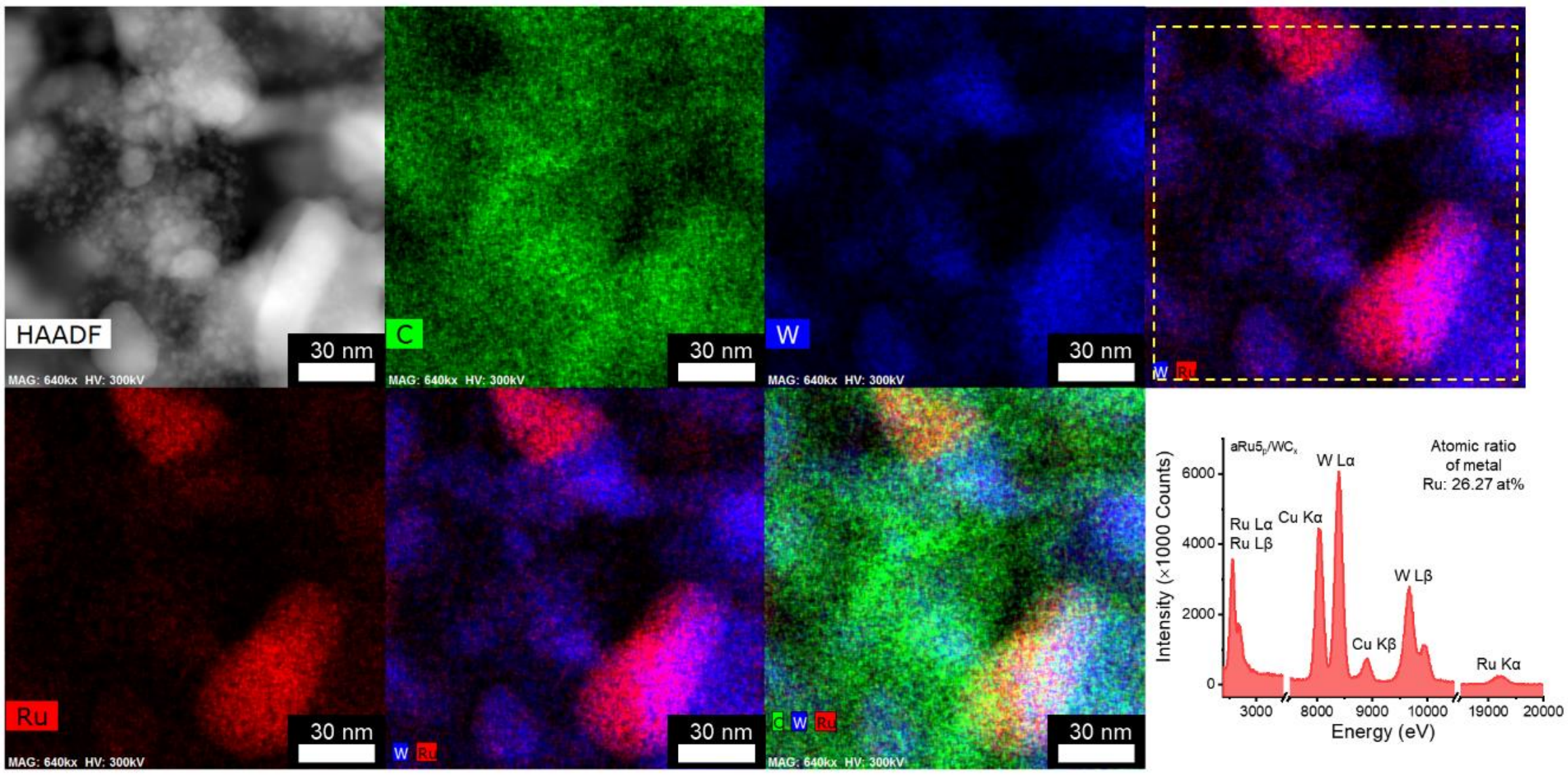

Supplementary Fig. 25 | STEM analysis of aRu5$_p$/WC$_x$. a, HAADF-STEM image and the corresponding EDS elemental mappings for C (green), W (blue), Ru (red), along with the overlaid composite map and the EDS spectrum showing the atomic ratio of metals (orange circles: Ru agglomerates). **b,** Higher-magnification HAADF-STEM image and the corresponding EDS mappings for the same elements, their composite overlay, and the EDS spectrum showing the atomic ratio of metals.

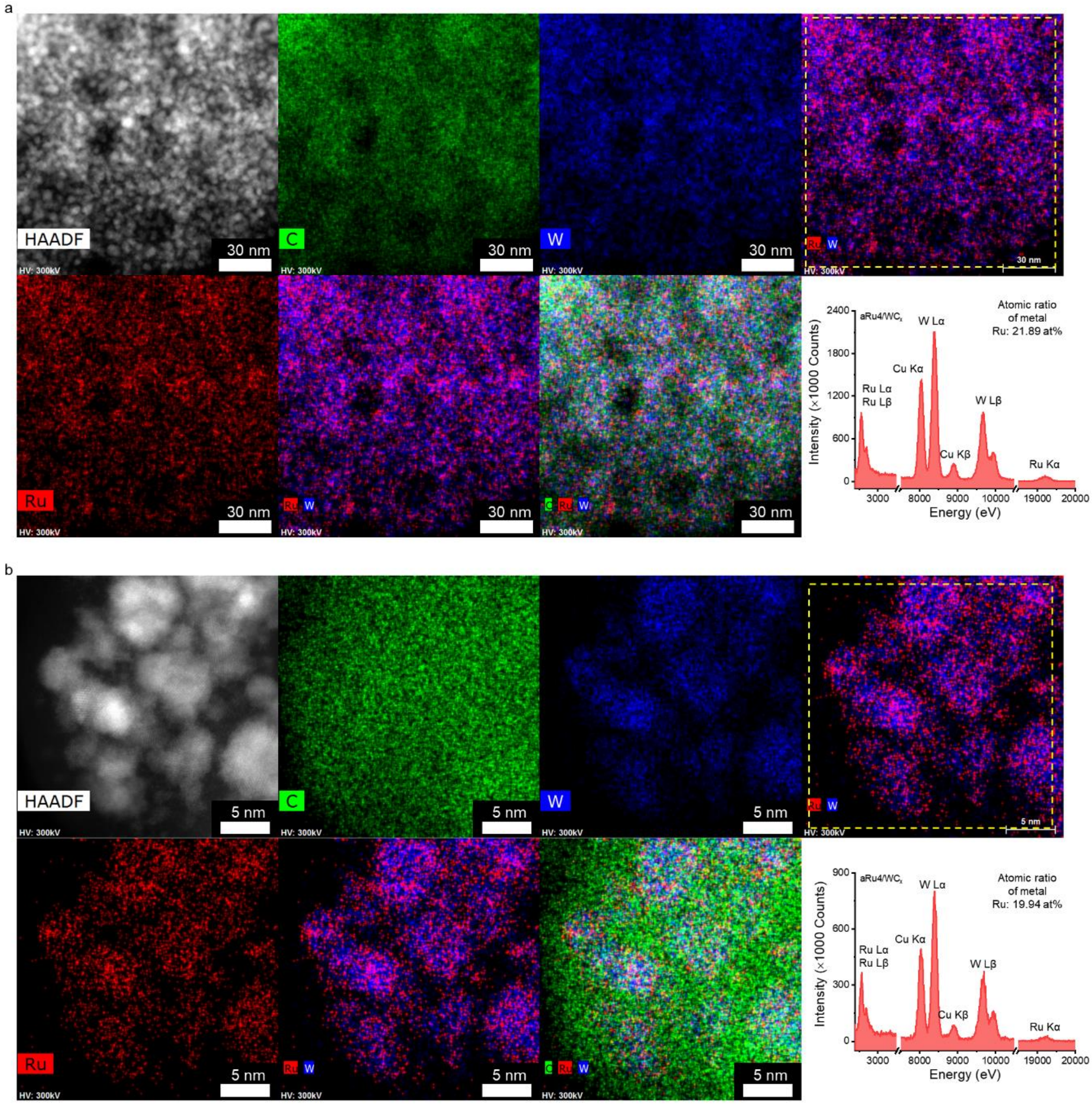

Supplementary Fig. 26 | STEM analysis of aRu4/WC$_x$. a, HAADF-STEM image and the corresponding EDS elemental mappings for C (green), W (blue), Ru (red), along with the overlaid composite map and the EDS spectrum showing the atomic ratio of metals. **b,** Higher-magnification HAADF-STEM image and the corresponding EDS mappings for the same elements, their composite overlay, and the EDS spectrum showing the atomic ratio of metals.

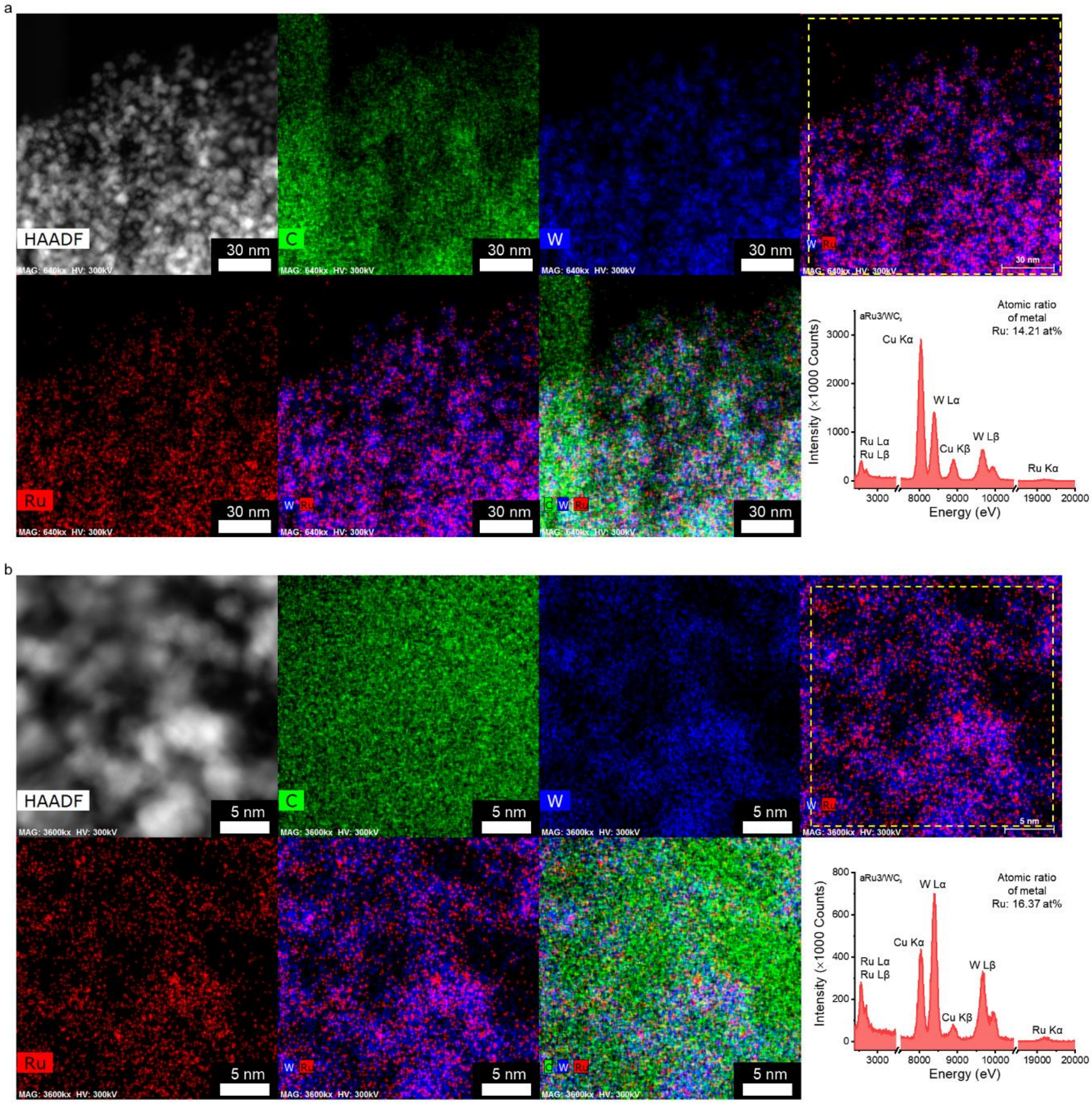

Supplementary Fig. 27 | STEM analysis of aRu3/WC$_x$. a, HAADF-STEM image and the corresponding EDS elemental mappings for C (green), W (blue), Ru (red), along with the overlaid composite map and the EDS spectrum showing the atomic ratio of metals. **b,** Higher-magnification HAADF-STEM image and the corresponding EDS mappings for the same elements, their composite overlay, and the EDS spectrum showing the atomic ratio of metals.

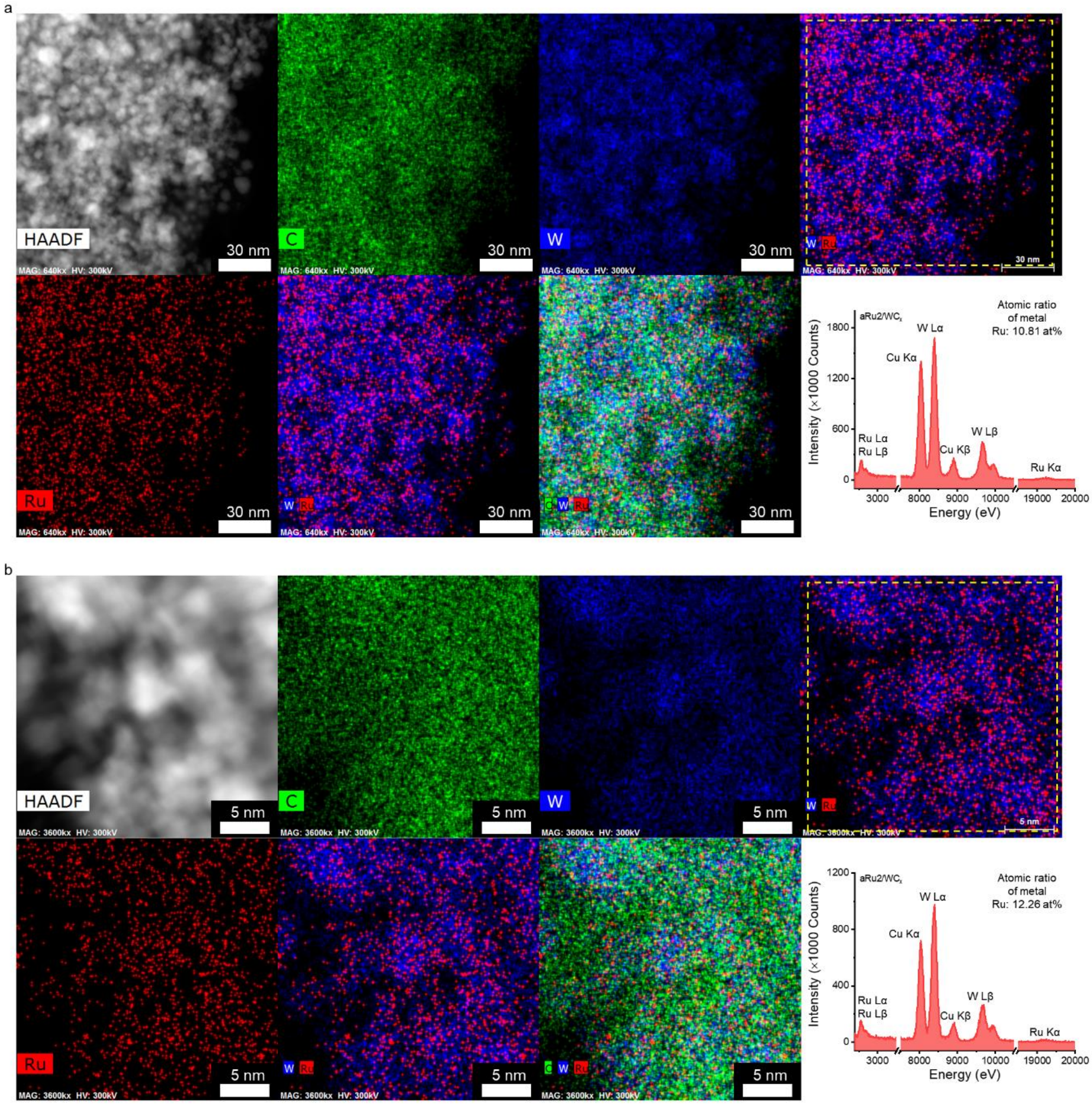

Supplementary Fig. 28 | STEM analysis of aRu2/WC$_x$. a, HAADF-STEM image and the corresponding EDS elemental mappings for C (green), W (blue), Ru (red), along with the overlaid composite map and the EDS spectrum showing the atomic ratio of metals. **b,** Higher-magnification HAADF-STEM image and the corresponding EDS mappings for the same elements, their composite overlay, and the EDS spectrum showing the atomic ratio of metals.

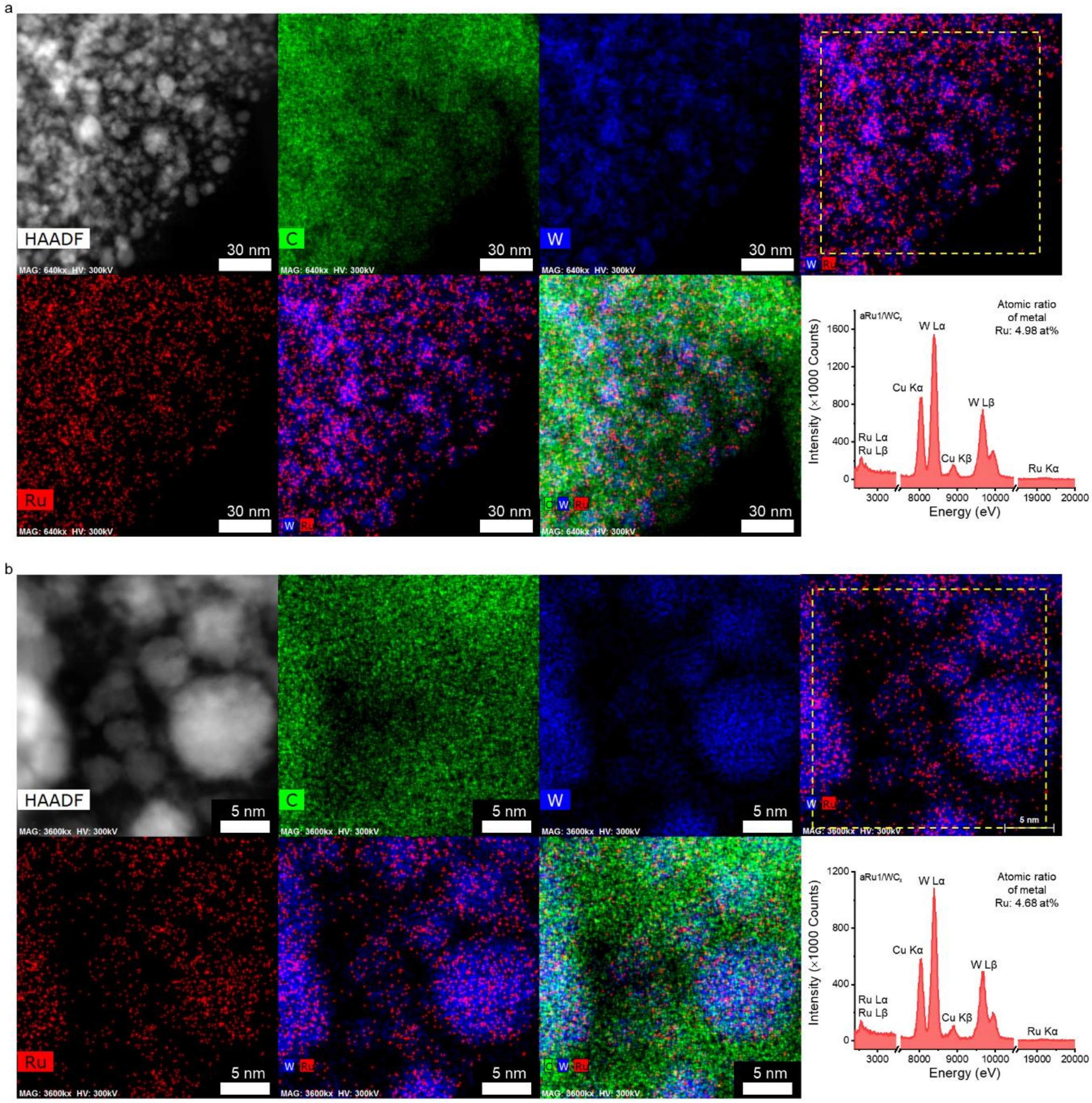

Supplementary Fig. 29 | STEM analysis of aRu1/WC$_x$. a, HAADF-STEM image and the corresponding EDS elemental mappings for C (green), W (blue), Ru (red), along with the overlaid composite map and the EDS spectrum showing the atomic ratio of metals. **b,** Higher-magnification HAADF-STEM image and the corresponding EDS mappings for the same elements, their composite overlay, and the EDS spectrum showing the atomic ratio of metals.

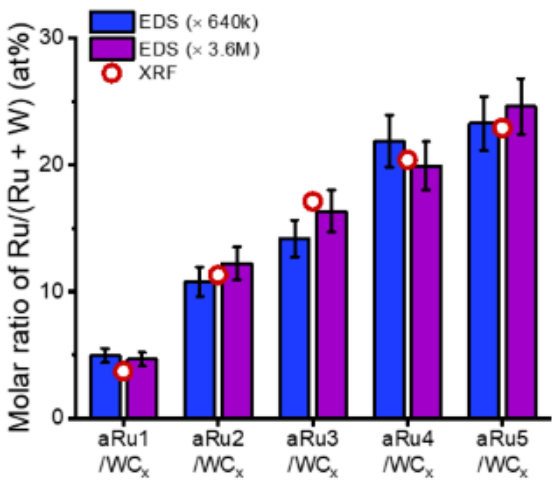

Supplementary Fig. 30 | Comparison of Ru atomic ratios obtained from EDS and XRF analyses. Molar ratios of Ru/(Ru + W) for aRu*N*/WC_x determined by EDS and X-ray fluorescence (XRF). Error bars represent the standard deviation of repeated EDS measurements at different regions.

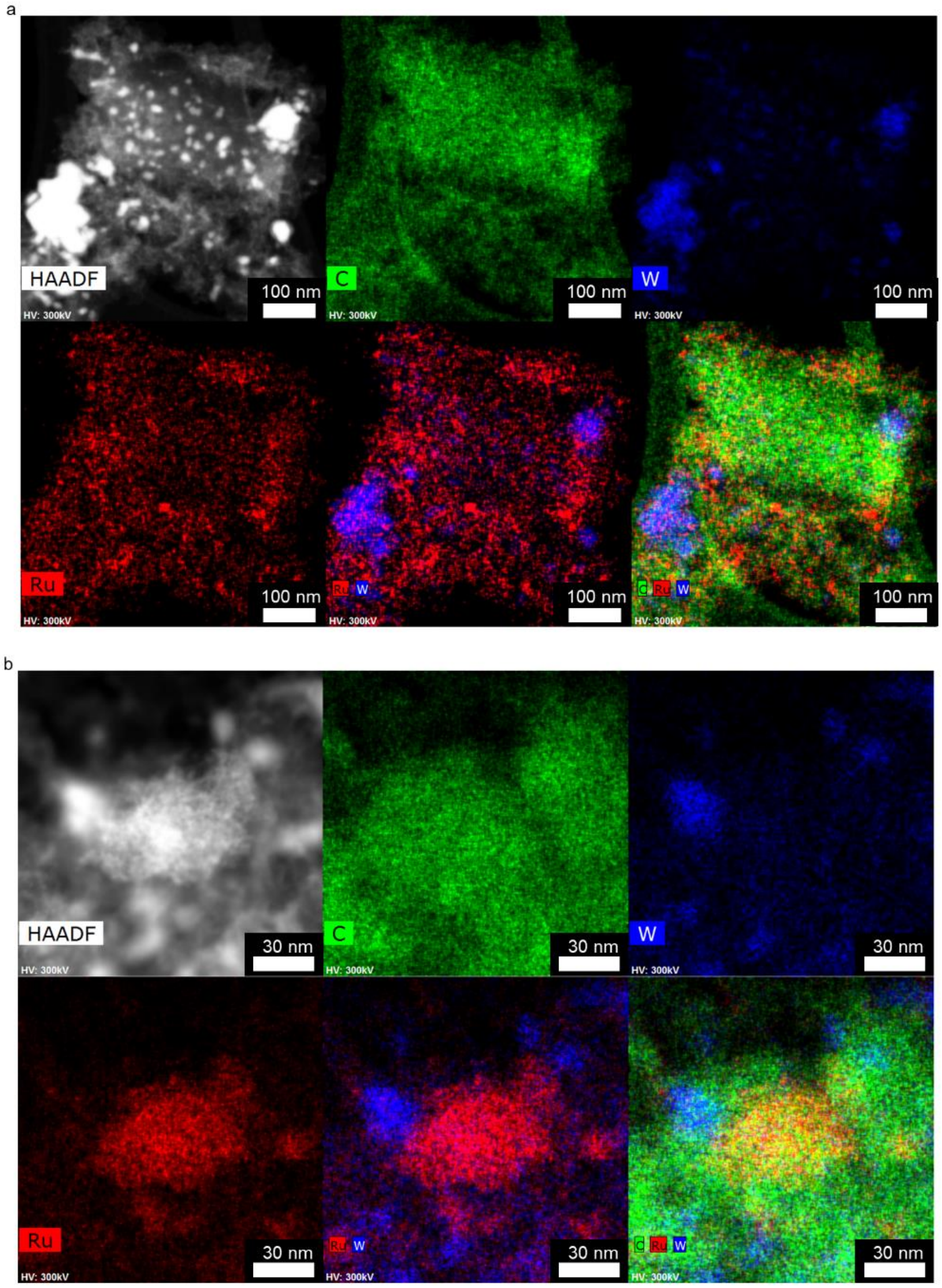

Supplementary Fig. 31 | STEM analysis of Ru_p/WC_x_H_2. a, HAADF-STEM image and the corresponding EDS elemental mappings for C (green), W (blue), and Ru (red), along with the composite overlay showing partial Ru agglomeration. **b,** Higher-magnification HAADF-STEM image and the corresponding EDS mappings for the same elements and their composite overlay.

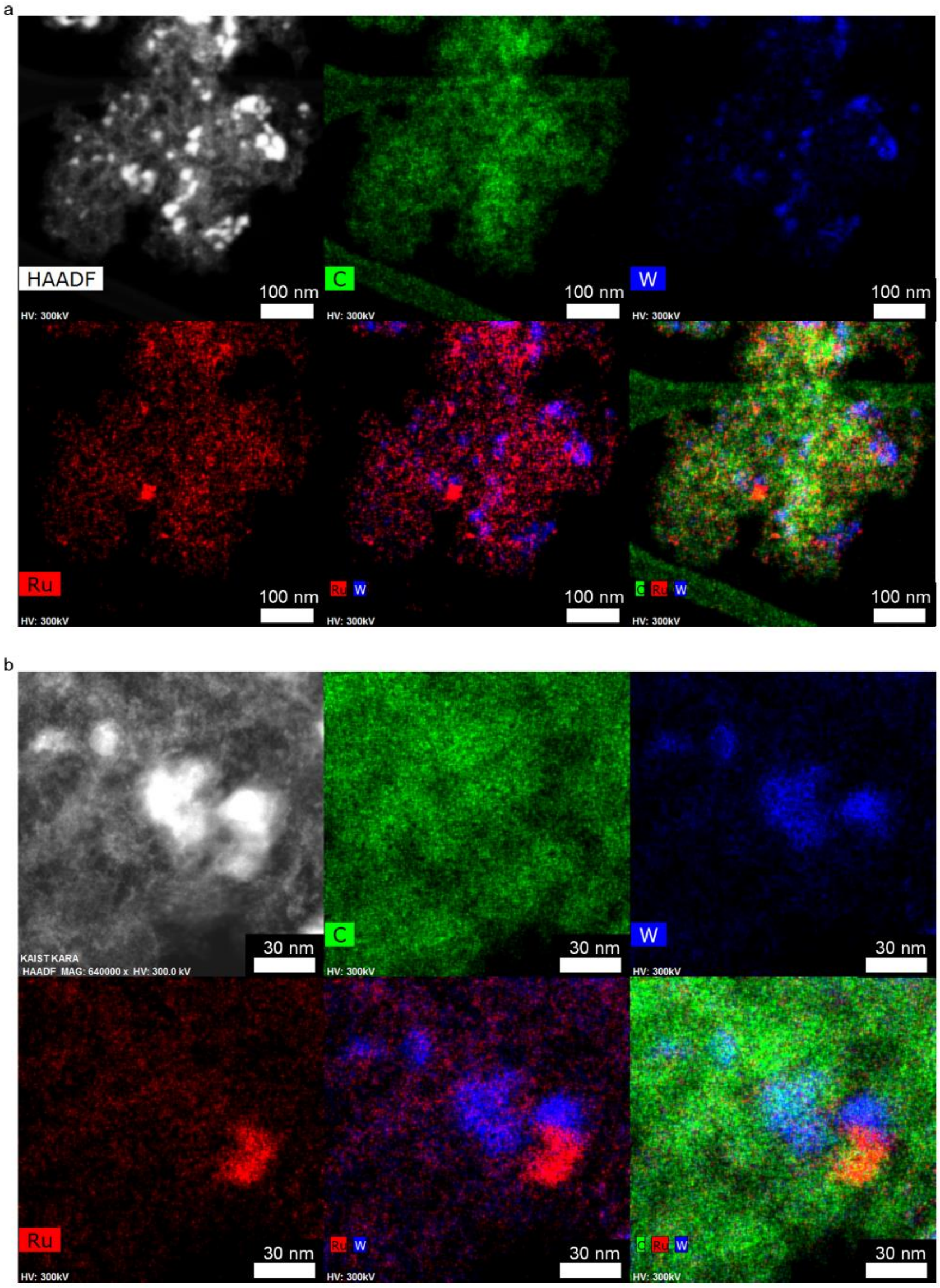

Supplementary Fig. 32 | STEM analysis of Ru/WC$_x$_H$_2$. a, HAADF-STEM image and the corresponding EDS elemental mappings for C (green), W (blue), and Ru (red), along with the composite overlay showing partial Ru agglomeration. **b,** Higher-magnification HAADF-STEM image and the corresponding EDS mappings for the same elements and their composite overlay.

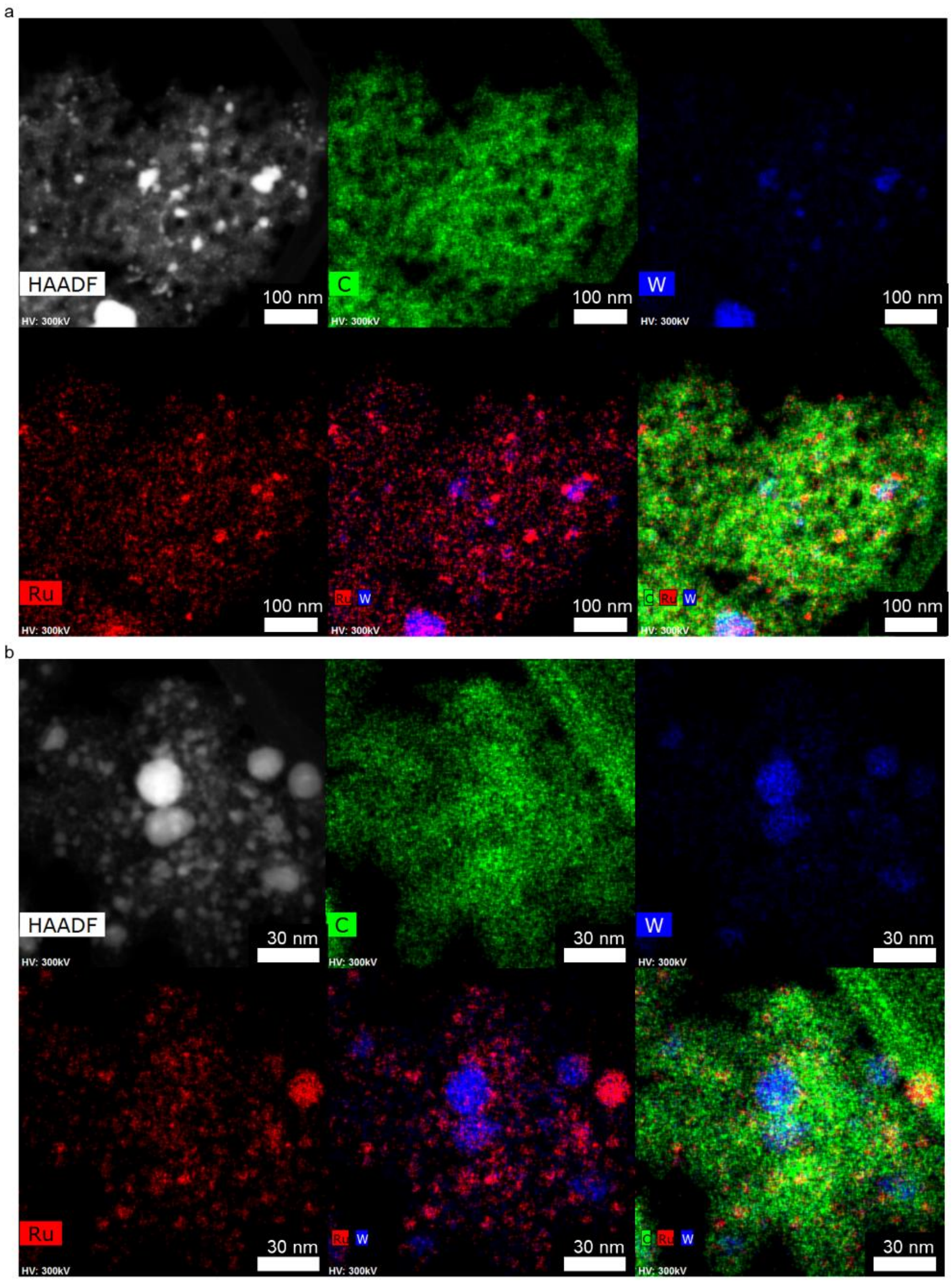

Supplementary Fig. 33 | STEM analysis of Ru_p/WC_x_Ar. a, HAADF-STEM image and the corresponding EDS elemental mappings for C (green), W (blue), and Ru (red), along with the composite overlay showing partial Ru agglomeration. **b,** Higher-magnification HAADF-STEM image and the corresponding EDS mappings for the same elements and their composite overlay.

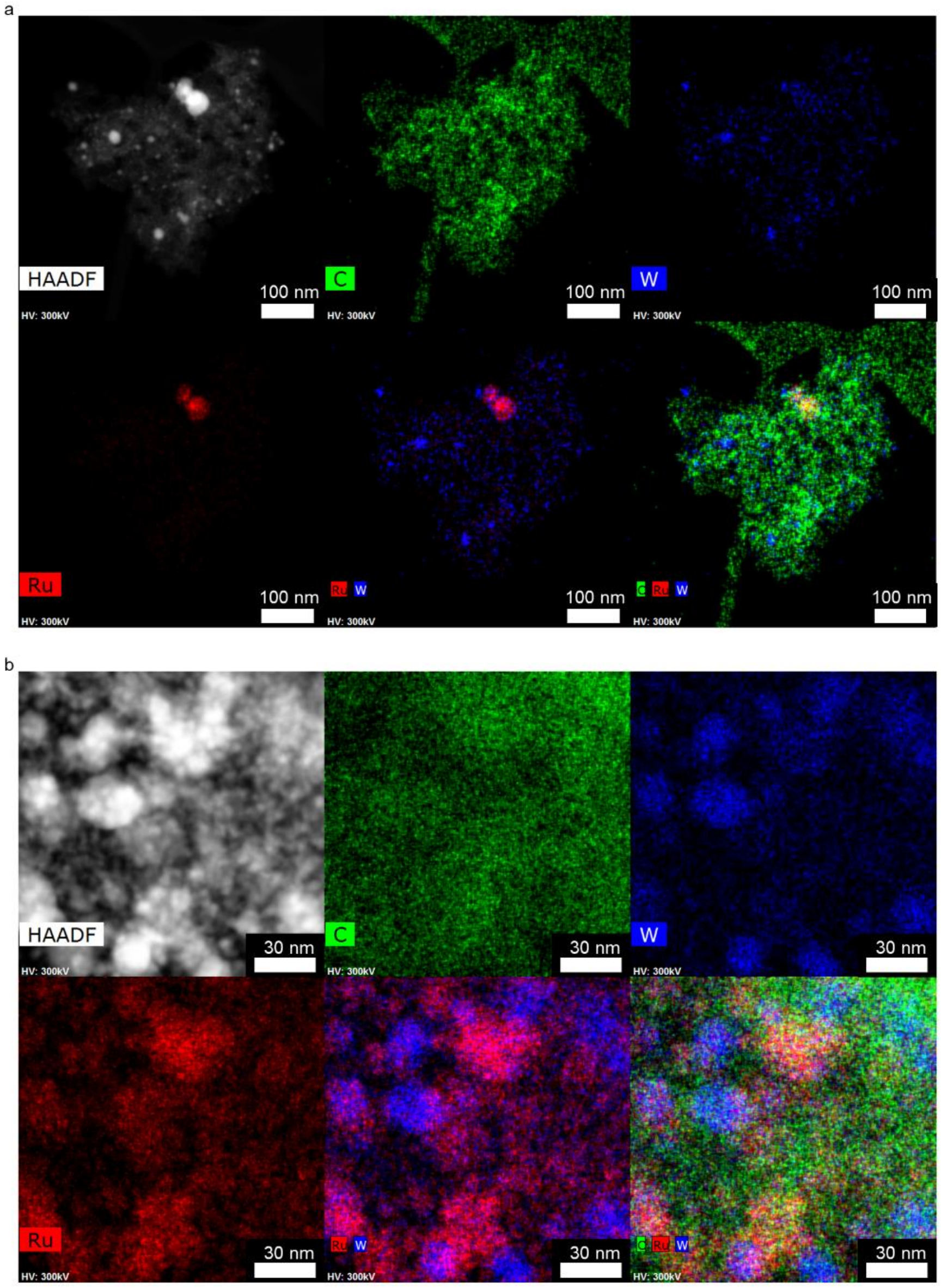

Supplementary Fig. 34 | STEM analysis of Ru/WC$_x$_Ar. a, HAADF-STEM image and the corresponding EDS elemental mappings for C (green), W (blue), and Ru (red), along with the composite overlay showing partial Ru agglomeration. **b,** Higher-magnification HAADF-STEM image and the corresponding EDS mappings for the same elements and their composite overlay.

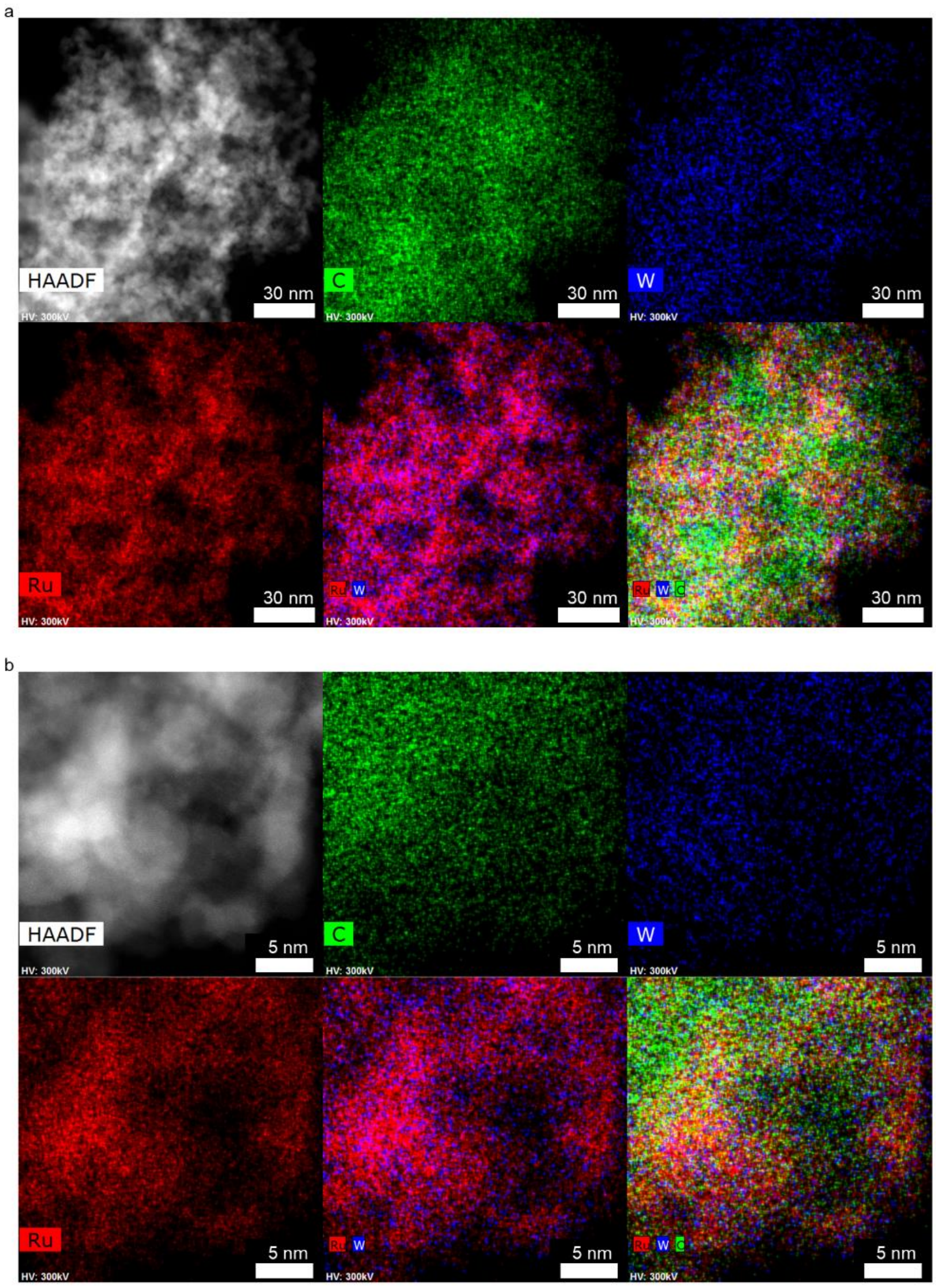

Supplementary Fig. 35 | STEM analysis of Ru/WC$_x$_R. a, HAADF-STEM image and the corresponding EDS elemental mappings for C (green), W (blue), and Ru (red), along with the composite overlay. **b,** Higher-magnification HAADF-STEM image and the corresponding EDS mappings for the same elements and their composite overlay.

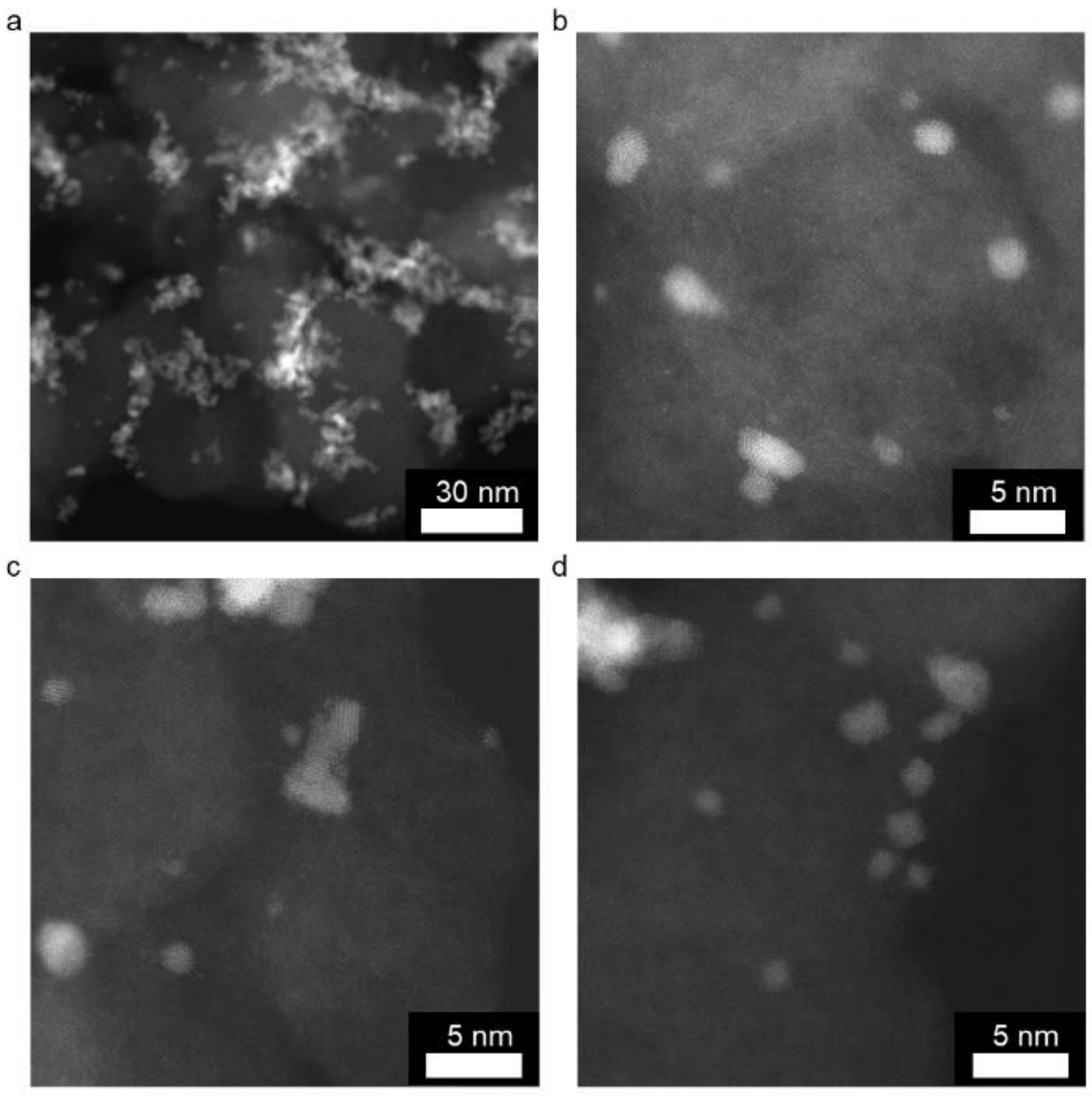

Supplementary Fig. 36 | STEM analysis of Ru/C_R. a, HAADF-STEM images and **b-d**, higher magnification HAADF-STEM images of Ru/C_R.

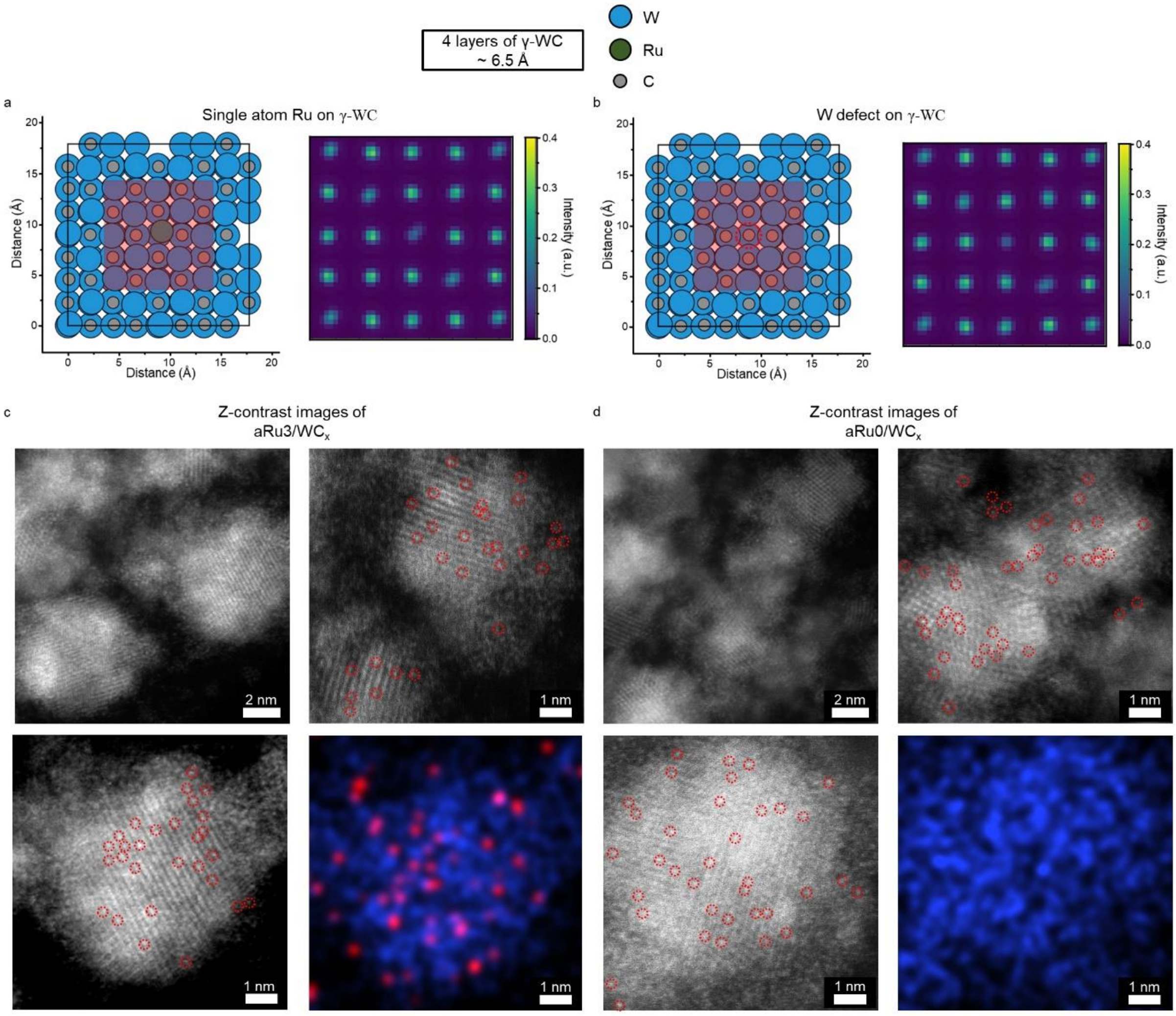

Supplementary Fig. 37 | Comparison of simulated and experimental HAADF-STEM images to evaluate Ru identification on WC_x supports. a,b, Density functional theory (DFT) models and corresponding simulated HAADF-STEM images for (**a**) a single Ru atom on γ-WC and (**b**) a single W defect on γ-WC. **c,d,** High-resolution HAADF-STEM images and the corresponding EDS elemental mappings of Ru (red) and W (blue) for (**c**) aRu3/WC_x and (**d**) Ru-free aRu0/WC_x. Bright contrast spots highlighted by red circles correspond to W atoms adjacent to Ru or intact WC_x lattice sites without Ru, confirming that HAADF-STEM Z-contrast image alone cannot be used to identify Ru atoms unambiguously.

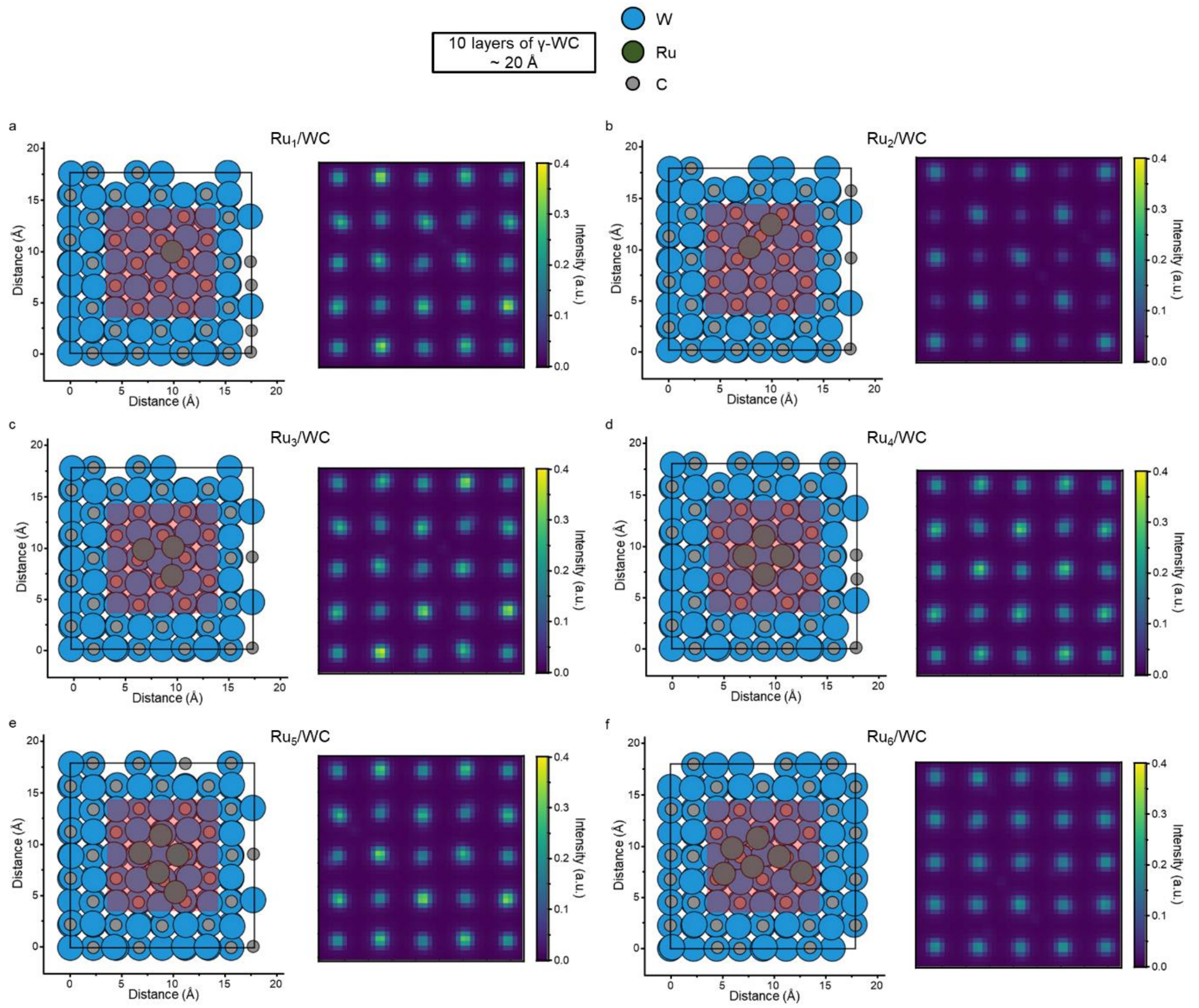

Supplementary Fig. 38 | Simulated HAADF-STEM images for Ru_N/WC models. a-f, DFT models and corresponding simulated HAADF-STEM images for (**a**) Ru_1/WC, (**b**) Ru_2/WC, (**c**) Ru_3/WC, (**d**) Ru_4/WC, (**e**) Ru_5/WC, and (**f**) Ru_6/WC.

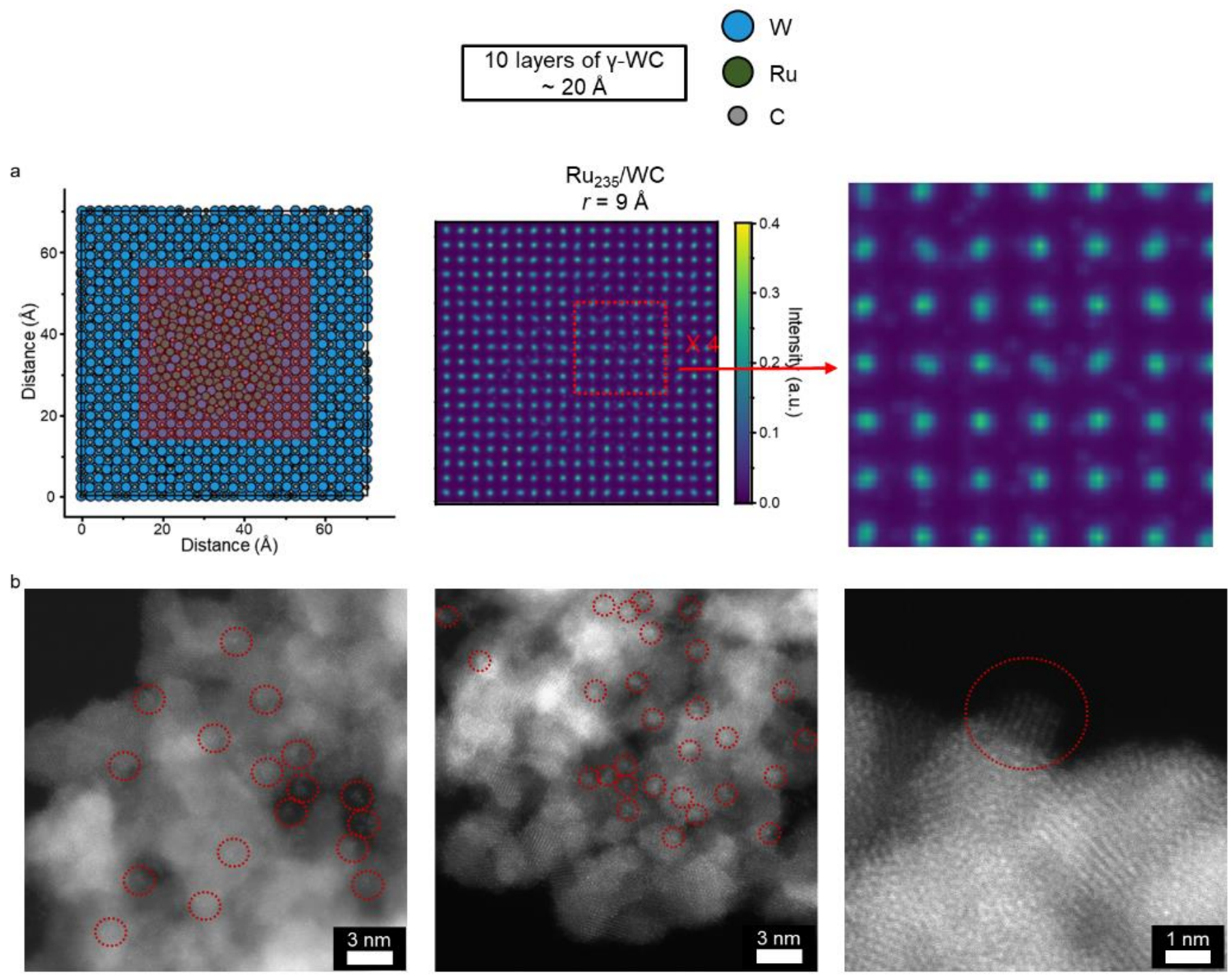

Supplementary Fig. 39 | Comparison of simulated and experimental HAADF-STEM images showing the formation of three-dimensional Ru nanoclusters on WC_x supports. a, DFT-optimized atomic model and the corresponding simulated HAADF-STEM image (with a 4× magnified view) for Ru_{235}/WC, representing the structural evolution from two-dimensional Ru ALNs to three-dimensional configurations as the number of Ru atoms increases. **b,** Experimental HAADF-STEM images of Ru/WCx_R, an intentionally prepared sample containing three-dimensional Ru nanoclusters on WC_x supports. The bright contrast regions highlighted by red circles correspond to lattice sites deviating from the regular WC_x periodicity, indicating the presence of sub-nanometer (<1 nm) three-dimensional Ru clusters.

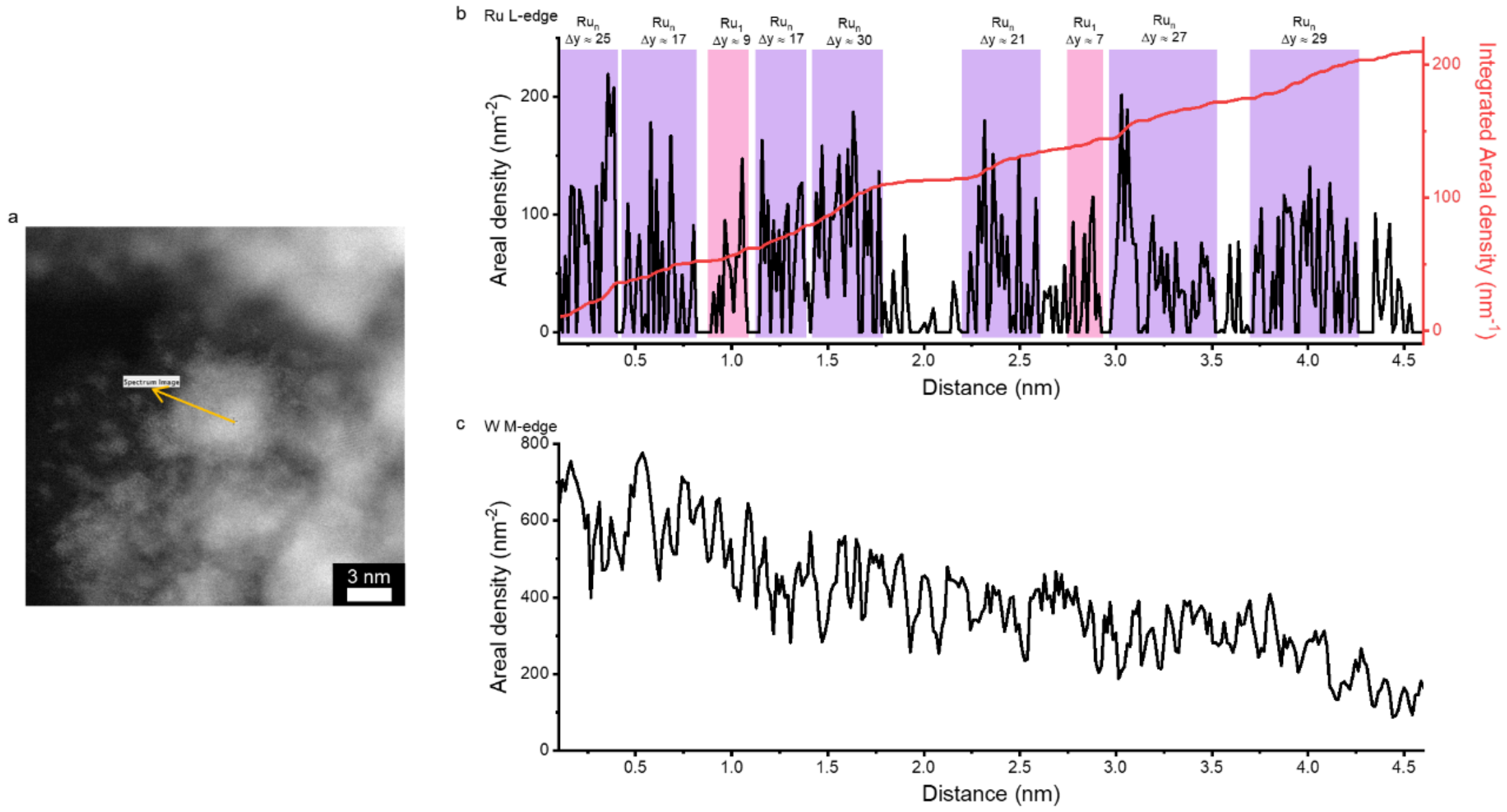

Supplementary Fig. 40 | Atomic-resolution electron energy-loss spectroscopy (EELS) analysis for aRu3/WC$_x$. a, High-resolution HAADF-STEM image showing EELS line-scanning path. **b, c,** Areal density profiles (black) and integrated areal density (red) obtained from EELS line scanning for (**b**) Ru (Ru L-edge) and (**c**) W (W M-edge). The purple-shaded regions in (**b**) correspond to Ru ensembles, indicating the presence of adjacent Ru atoms along the scanning direction.

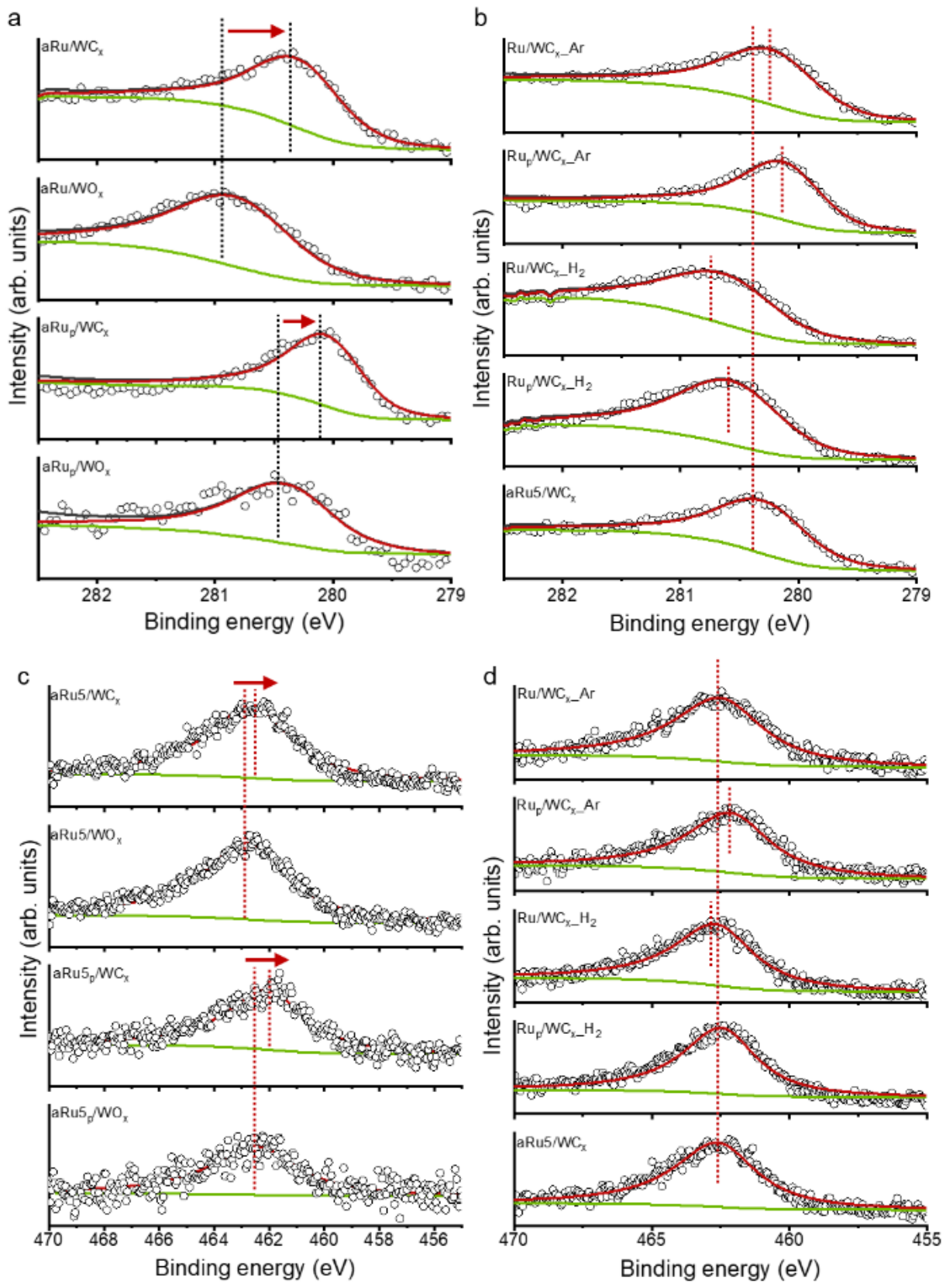

Supplementary Fig. 41 | Alteration of Ru electronic structure. a,b, Ru 3*d* region and **c,d,** Ru 3*p* region of X-ray photoelectron spectroscopy (XPS) for samples obtained by self-assembly or incipient wetness impregnation (IWI).

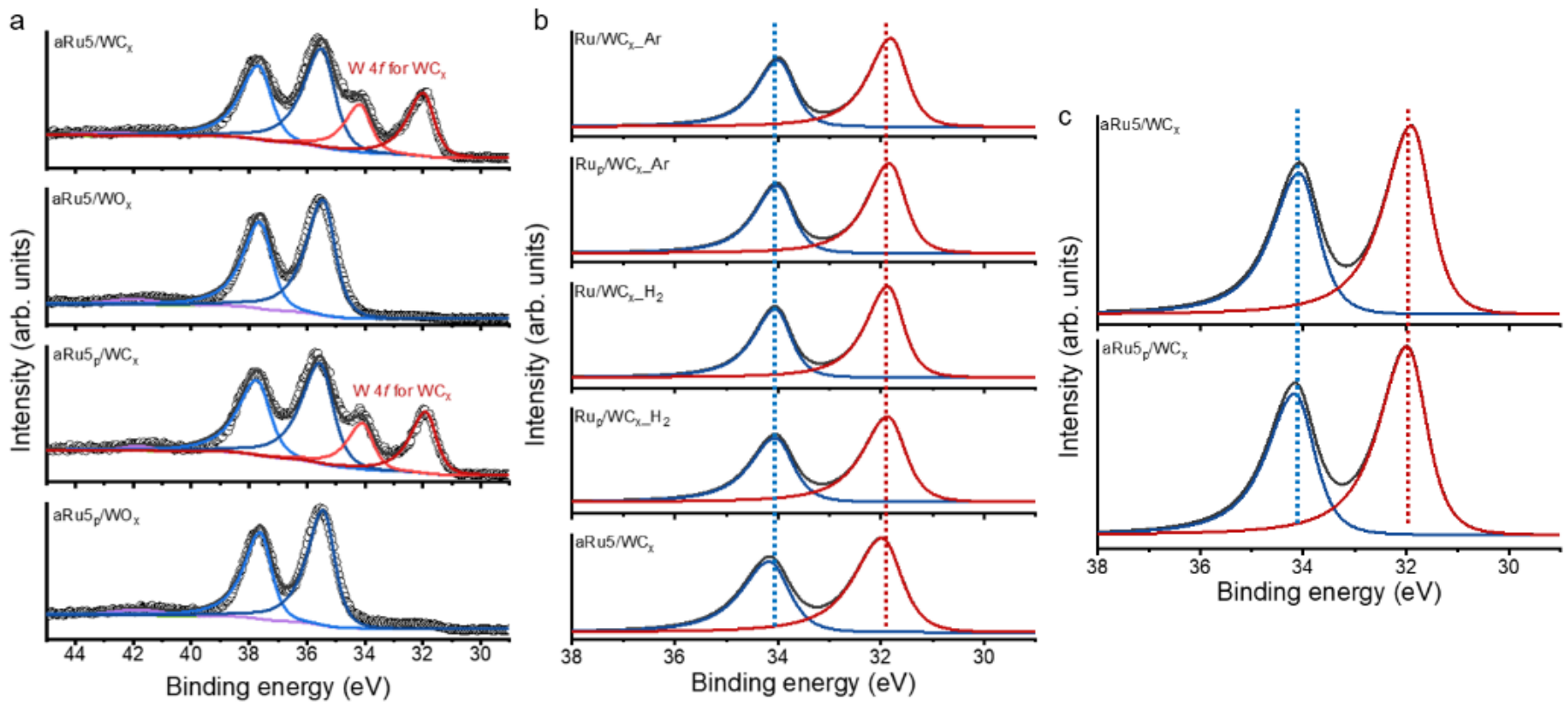

Supplementary Fig. 42 | Alteration of W electronic structure. a-c, XPS analysis of the W 4*f* region for (**a**) samples prepared by self-assembly at different annealing temperature, (**b**) samples prepared by IWI and aRu5/WC_x, (**c**) aRu5/WC_x, and aRu5$_p$/WC_x.

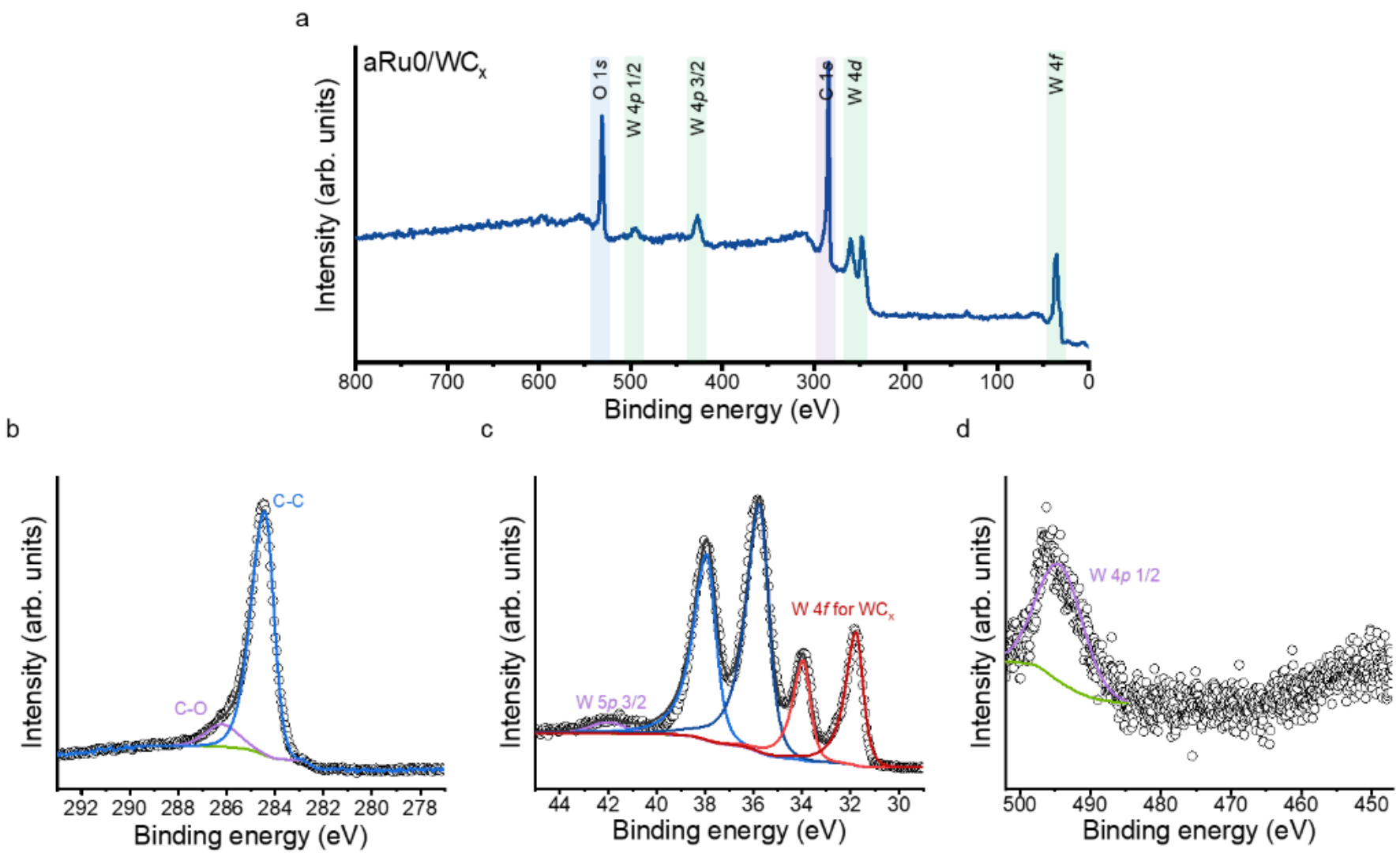

Supplementary Fig. 43 | Electronic structure of aRu0/WC$_x$. XPS analysis of aRu0/WC$_x$. **a,** XPS survey spectrum, **b,** C 1*s* region (blue: C=C, purple: C-O), **c,** W 4*f* region (red: WC$_x$, blue: surface WO$_x$ attributed to naturally formed oxides, purple: W 5*p* 3/2), and **d,** Ru 3*p* region (purple: W 4*p* 1/2).

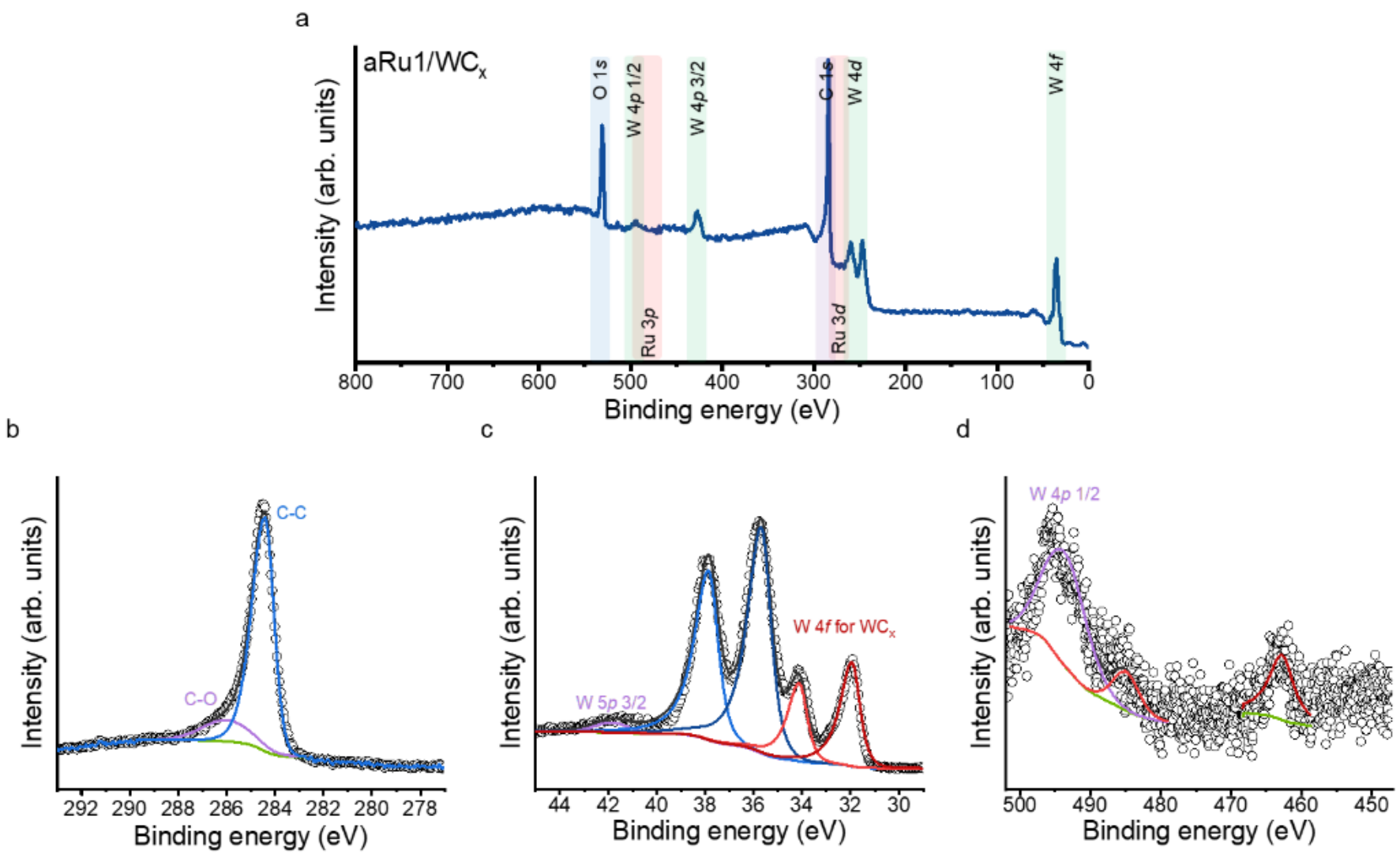

Supplementary Fig. 44 | Electronic structure of aRu1/WC$_x$. XPS analysis of aRu1/WC$_x$. **a,** XPS survey spectrum, **b,** C 1*s* region (blue: C=C, purple: C-O), **c,** W 4*f* region (red: WC$_x$, blue: surface WO$_x$ attributed to naturally formed oxides, purple: W 5*p* 3/2), and **d,** Ru 3*p* region (purple: W 4*p* 1/2).

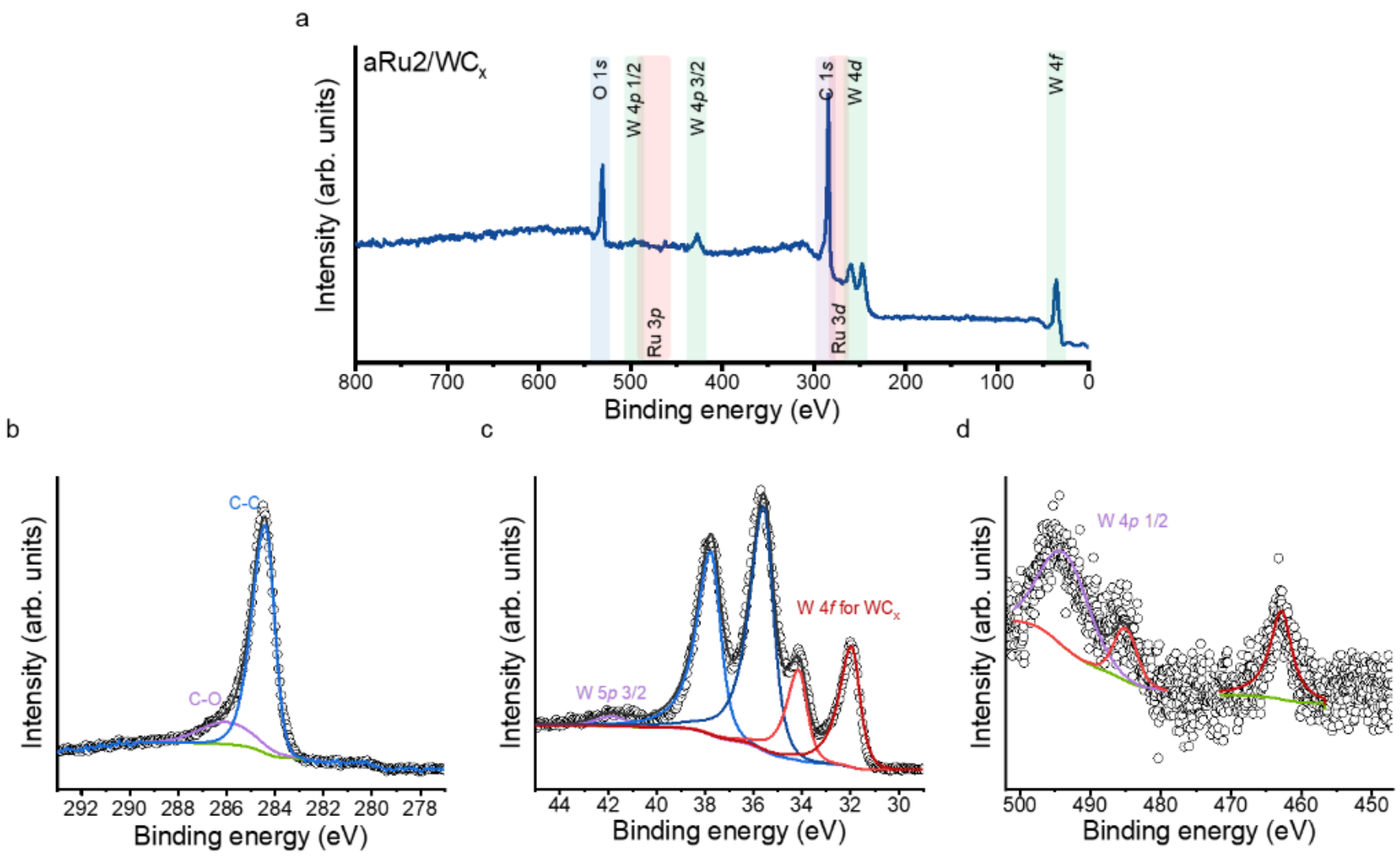

Supplementary Fig. 45 | Electronic structure of aRu2/WC$_x$. XPS analysis of aRu2/WC$_x$. **a,** XPS survey spectrum, **b,** C 1*s* region (blue: C=C, purple: C-O), **c,** W 4*f* region (red: WC$_x$, blue: surface WO$_x$ attributed to naturally formed oxides, purple: W 5*p* 3/2), and **d,** Ru 3*p* region (purple: W 4*p* 1/2).

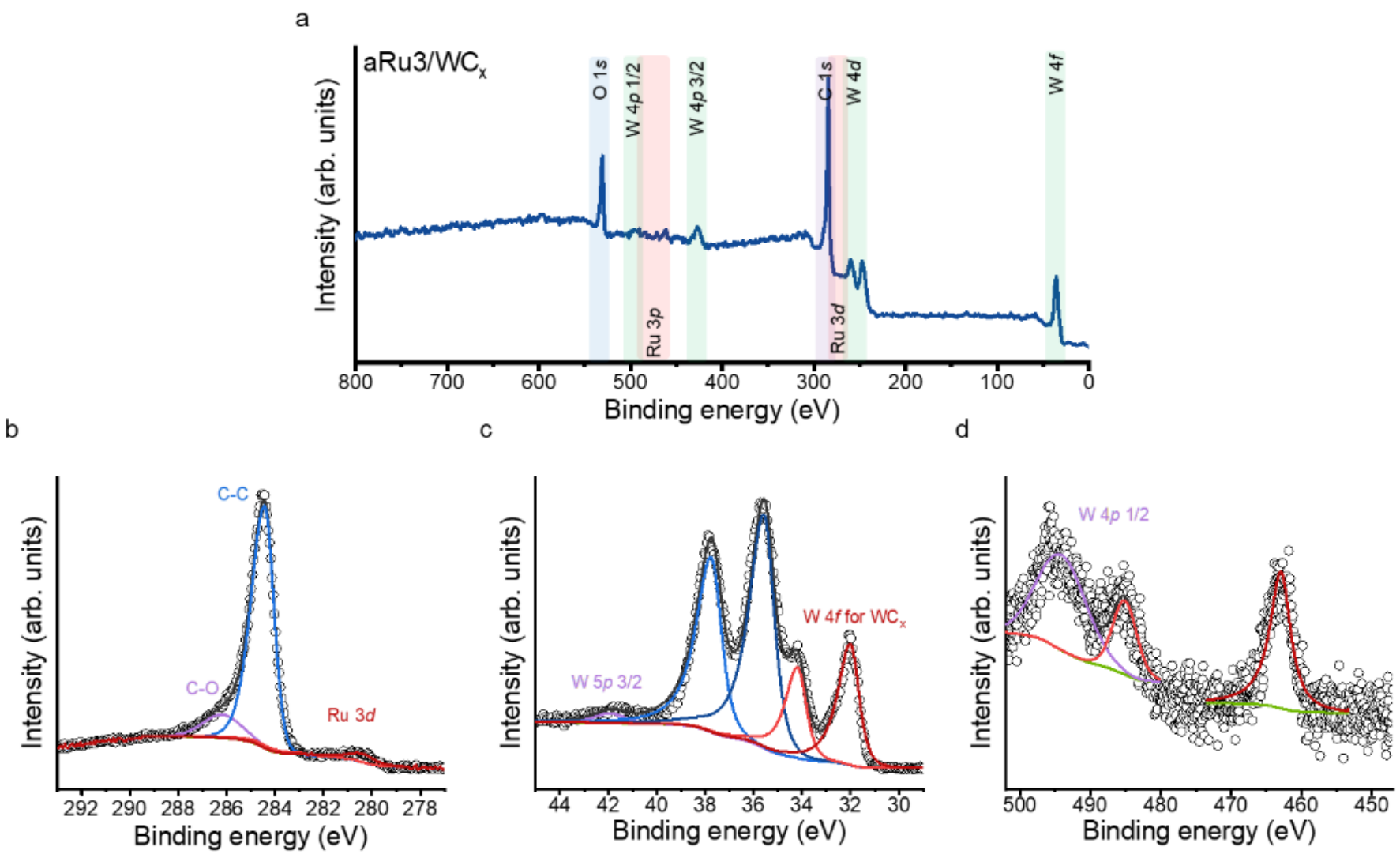

Supplementary Fig. 46 | Electronic structure of aRu3/WC$_x$. XPS analysis of aRu3/WC$_x$. **a,** XPS survey spectrum, **b,** C 1*s* region (red: Ru 3*d*, blue: C=C, purple: C-O), **c,** W 4*f* region (red: WC$_x$, blue: surface WO$_x$ attributed to naturally formed oxides, purple: W 5*p* 3/2), and **d,** Ru 3*p* region (purple: W 4*p* 1/2).

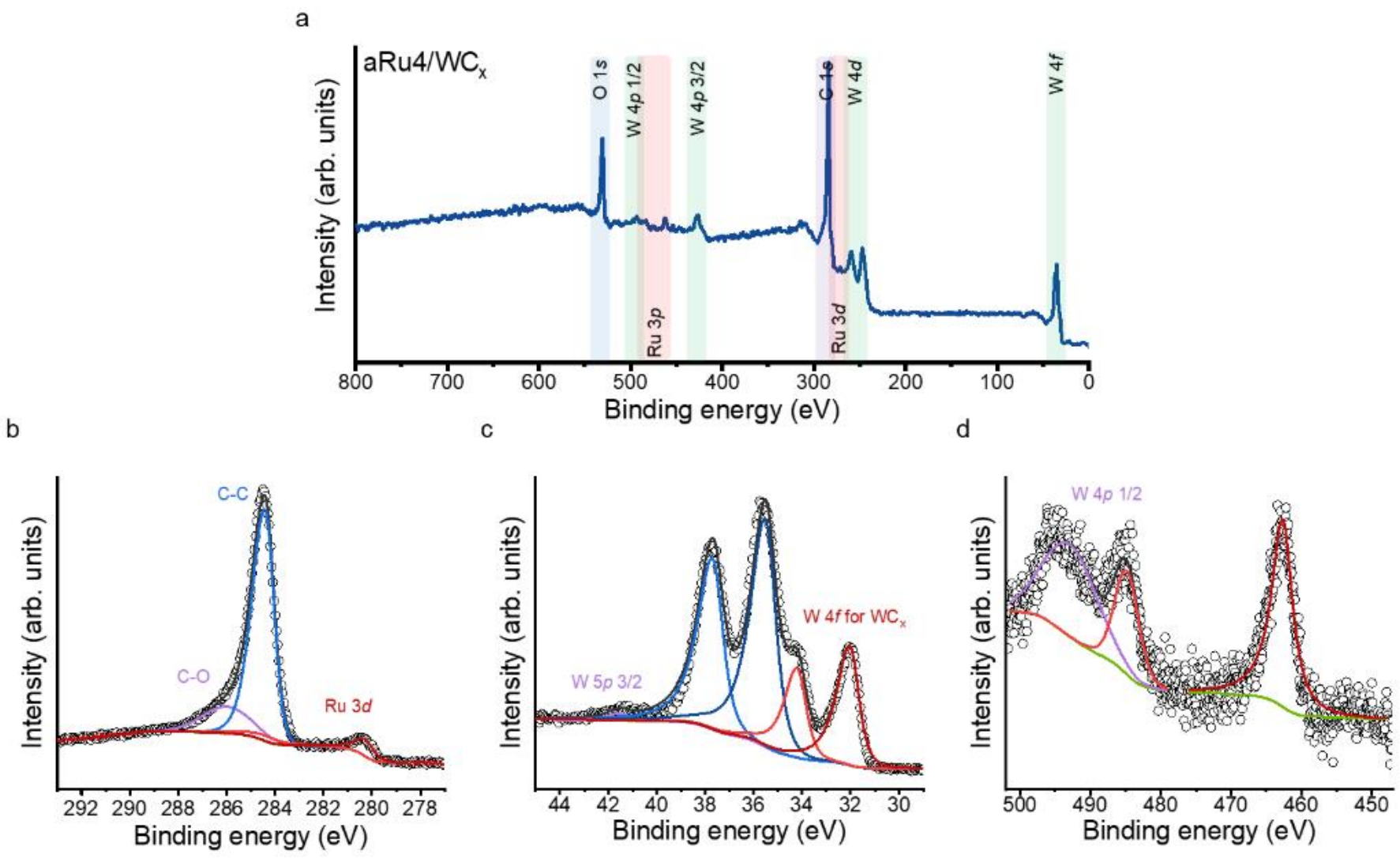

Supplementary Fig. 47 | Electronic structure of aRu4/WC_x. XPS analysis of aRu4/WC_x. **a,** XPS survey spectrum, **b,** C 1*s* region (red: Ru 3*d*, blue: C=C, purple: C-O), **c,** W 4*f* region (red: WC_x, blue: surface WO_x attributed to naturally formed oxides, purple: W 5*p* 3/2), and **d,** Ru 3*p* region (purple: W 4*p* 1/2).

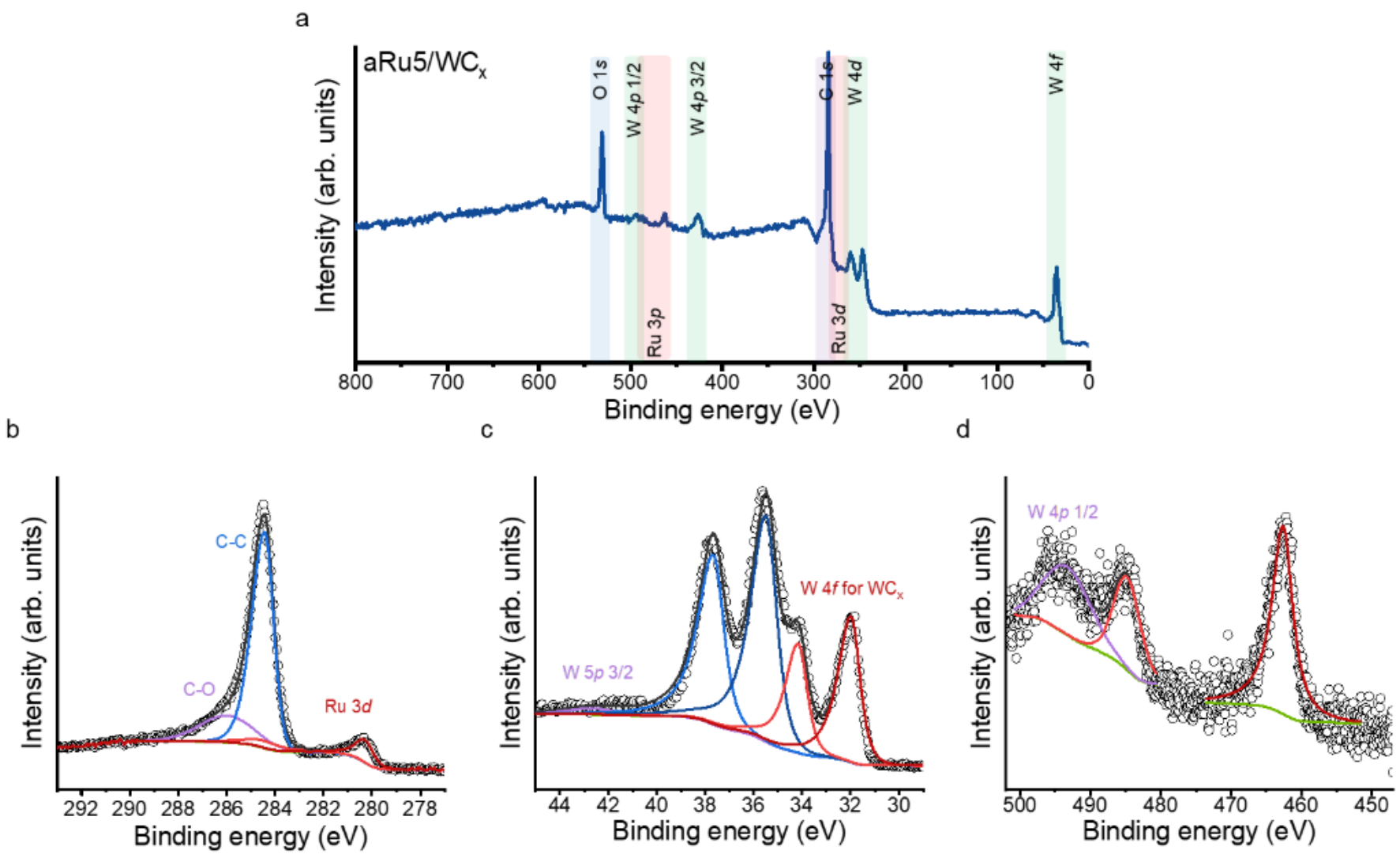

Supplementary Fig. 48 | Electronic structure of aRu5/WC_x. XPS analysis of aRu5/WC_x. **a,** XPS survey spectrum, **b,** C 1*s* region (red: Ru 3*d*, blue: C=C, purple: C-O), **c,** W 4*f* region (red: WC_x, blue: surface WO_x attributed to naturally formed oxides, purple: W 5*p* 3/2), and **d,** Ru 3*p* region (purple: W 4*p* 1/2).

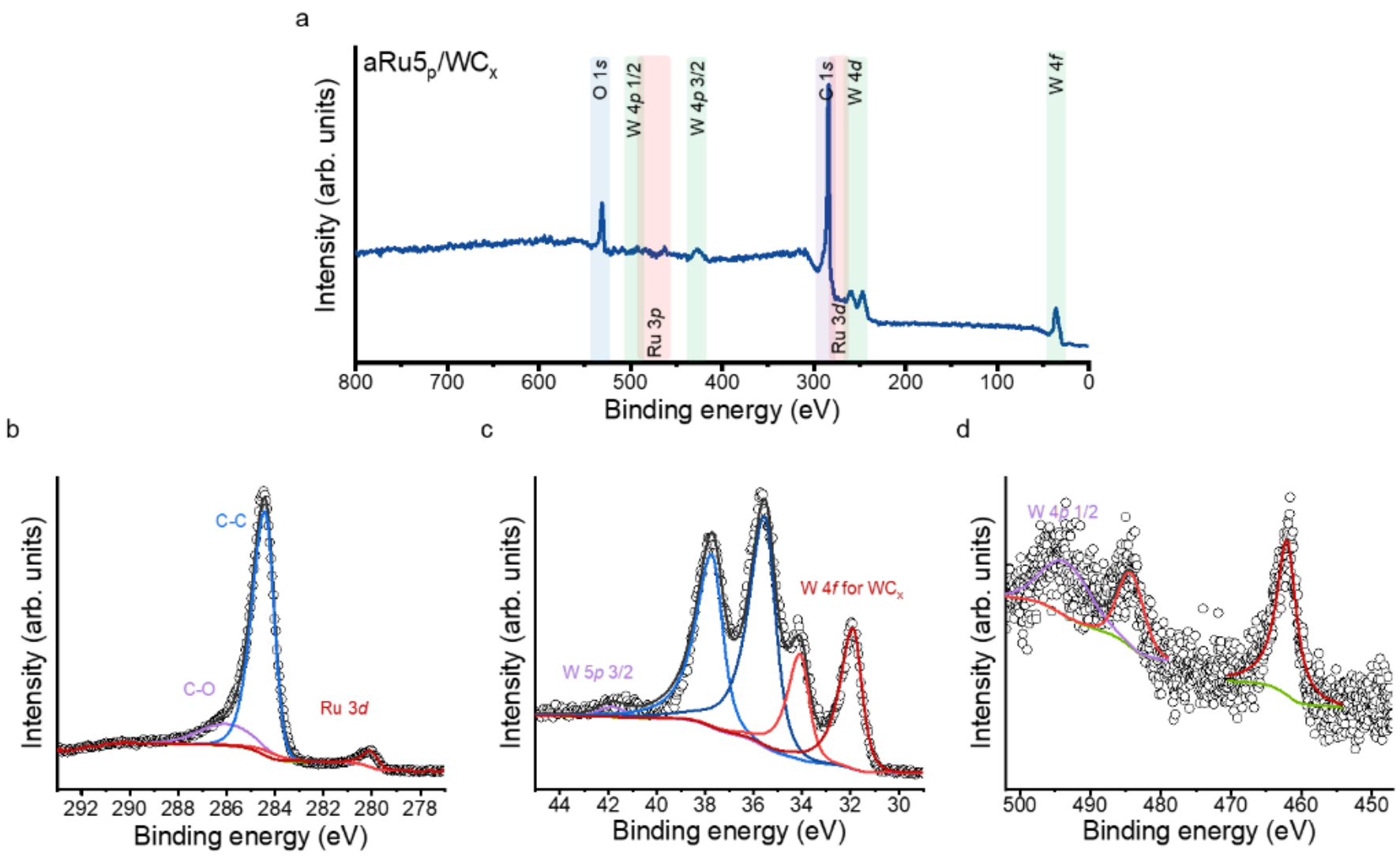

Supplementary Fig. 49 | Electronic structure of aRu5$_p$/WC$_x$. XPS analysis of aRu5$_p$/WC$_x$. **a,** XPS survey spectrum, **b,** C 1*s* region (red: Ru 3*d*, blue: C=C, purple: C-O), **c,** W 4*f* region (red: WC$_x$, blue: surface WO$_x$ attributed to naturally formed oxides, purple: W 5*p* 3/2), and **d,** Ru 3*p* region (purple: W 4*p* 1/2).

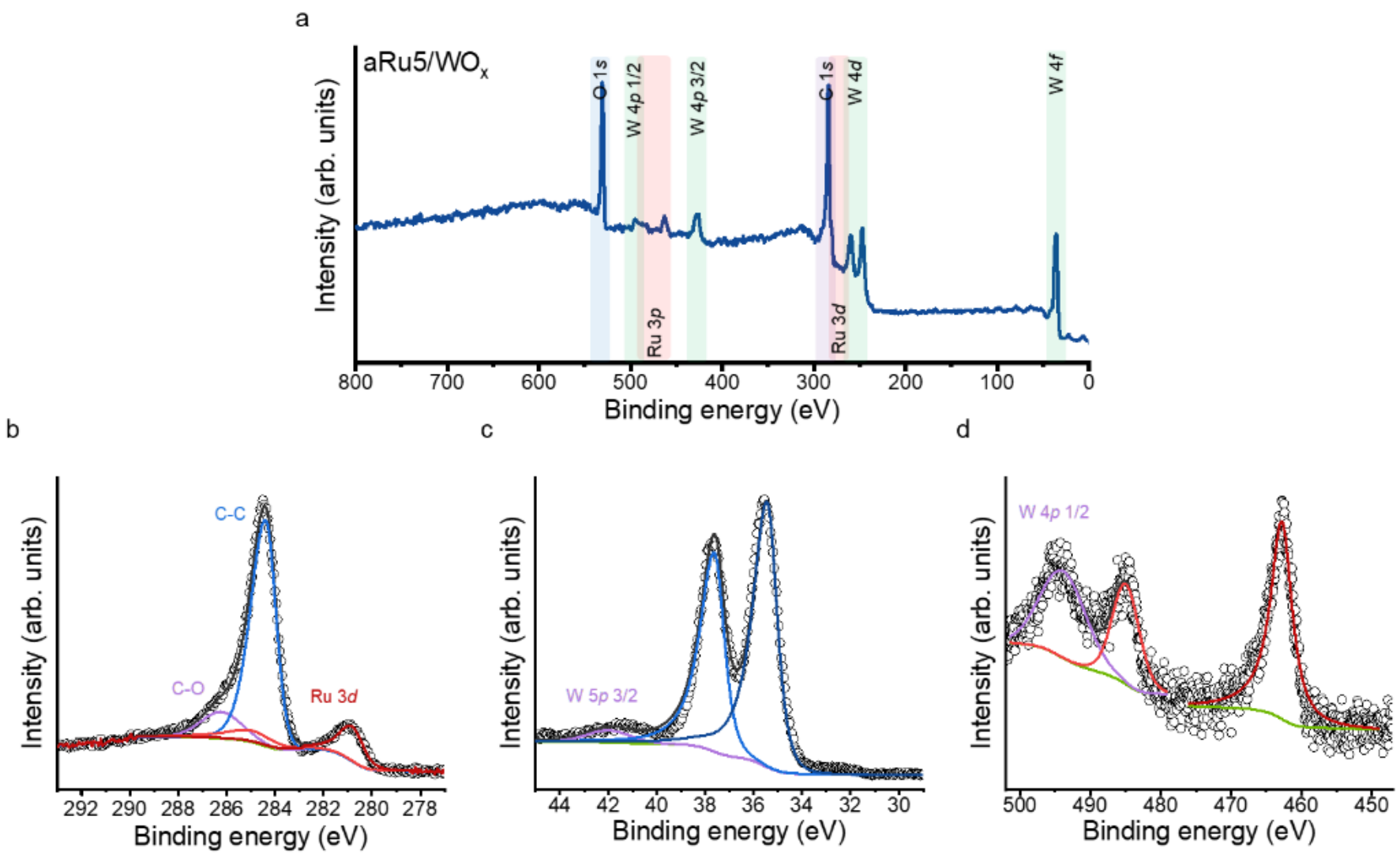

Supplementary Fig. 50 | Electronic structure of aRu5/WO$_x$. XPS analysis of aRu5/WO$_x$. **a,** XPS survey spectrum, **b,** C 1*s* region (red: Ru 3*d*, blue: C=C, purple: C-O), **c,** W 4*f* region (purple: W 5*p* 3/2), and **d,** Ru 3*p* region (purple: W 4*p* 1/2).

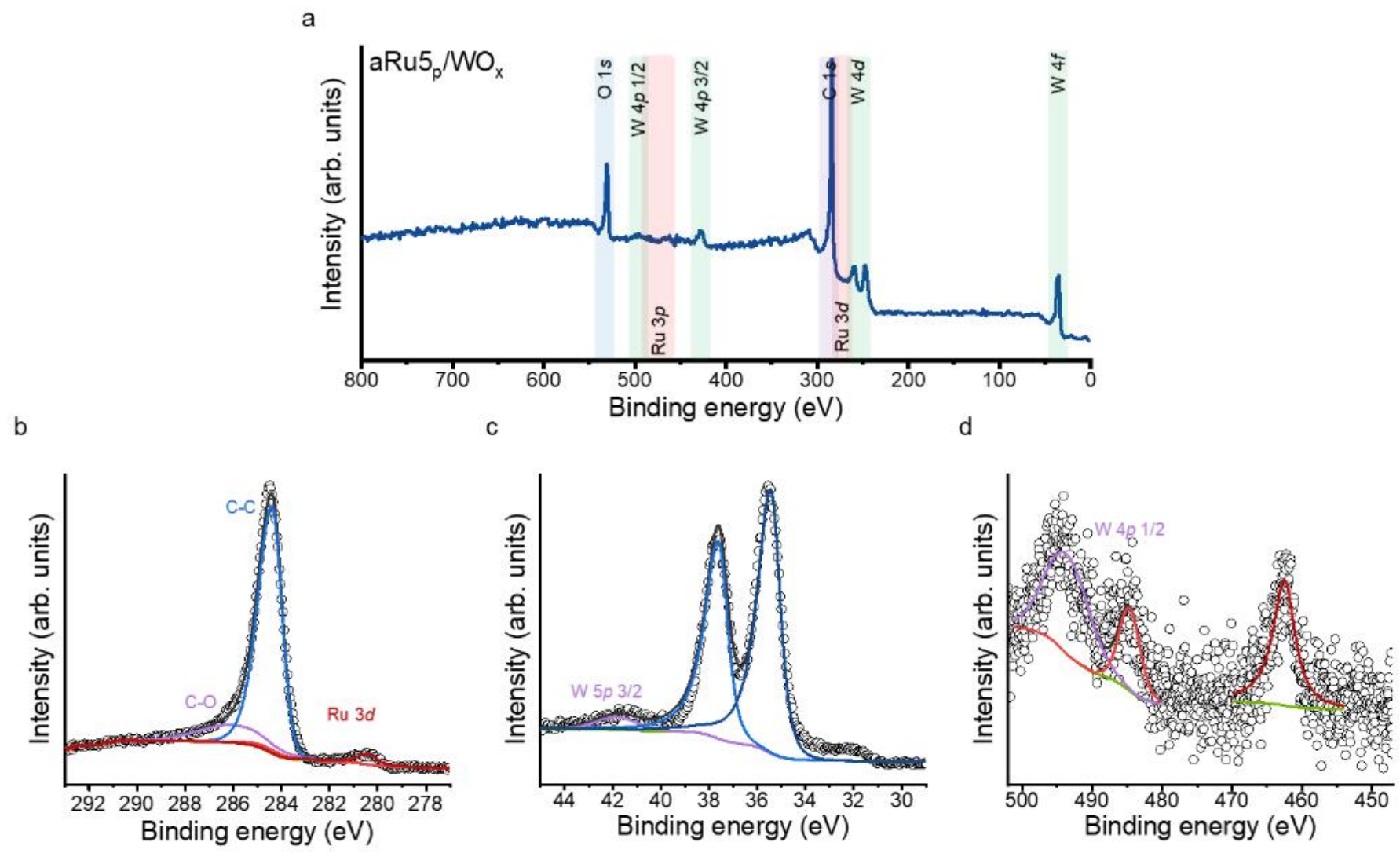

Supplementary Fig. 51 | Electronic structure of aRu5$_p$/WO$_x$. XPS analysis of aRu5$_p$/WO$_x$. **a,** XPS survey spectrum, **b,** C 1*s* region (red: Ru 3*d*, blue: C=C, purple: C-O), **c,** W 4*f* region (purple: W 5*p* 3/2), and **d,** Ru 3*p* region (purple: W 4*p* 1/2).

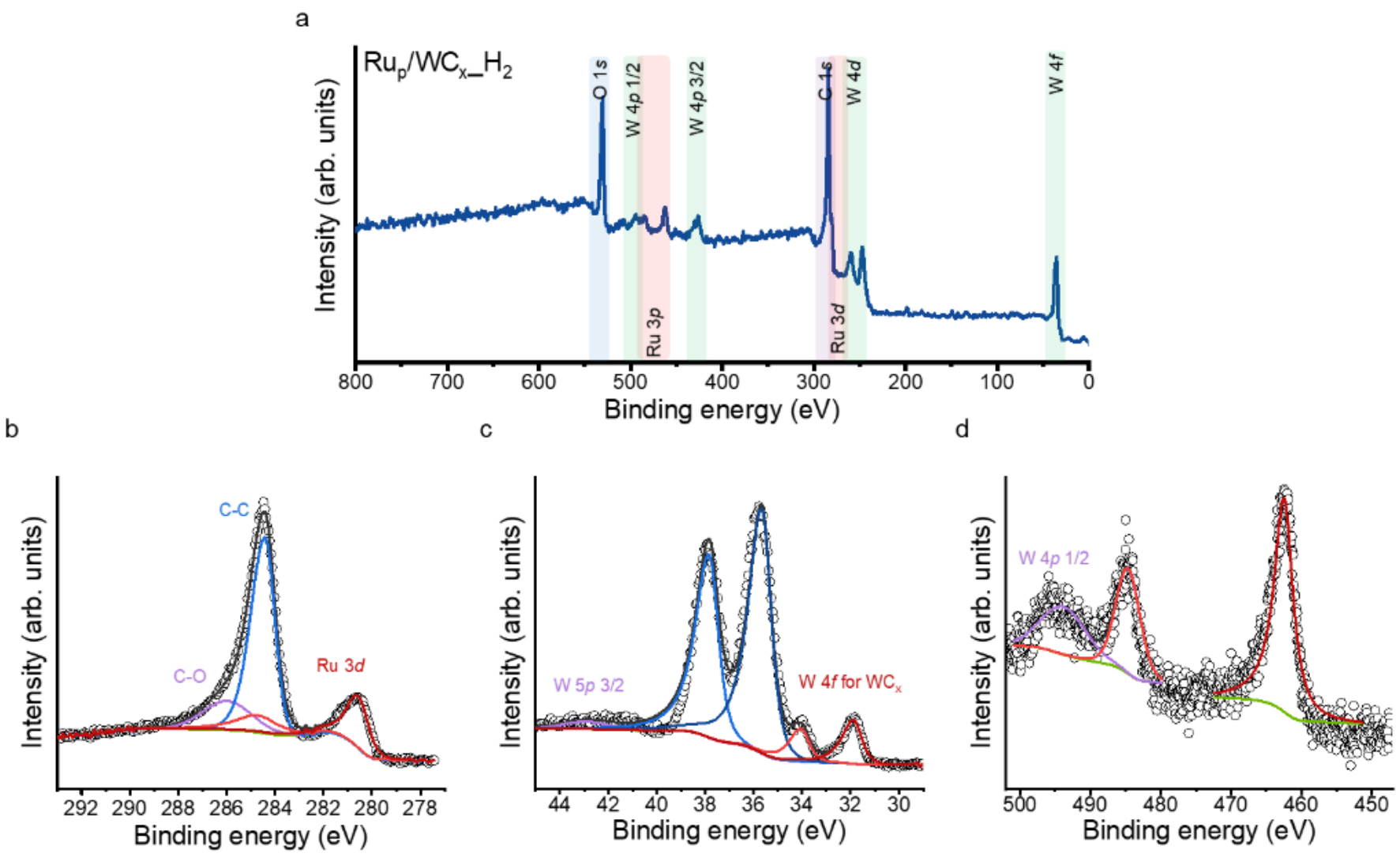

Supplementary Fig. 52 | Electronic structure of $Ru_p/WC_x\_H_2$. XPS analysis of $Ru_p/WC_x\_H_2$. **a,** XPS survey spectrum, **b,** C 1*s* region (red: Ru 3*d*, blue: C=C, purple: C-O), **c,** W 4*f* region (red: WC_x, blue: surface WO_x attributed to naturally formed oxides, purple: W 5*p* 3/2), and **d,** Ru 3*p* region (purple: W 4*p* 1/2).

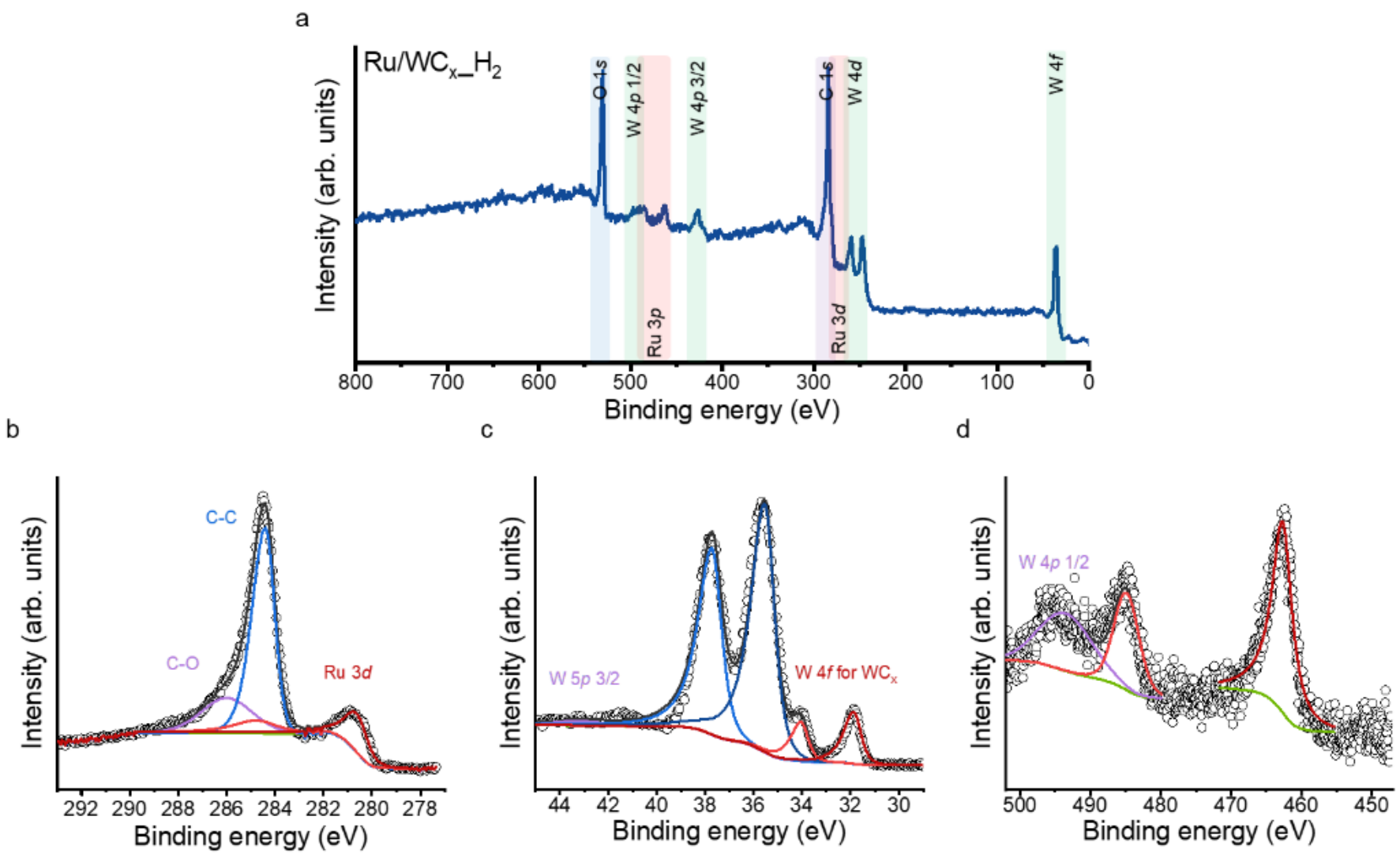

Supplementary Fig. 53 | Electronic structure of Ru/WC_x_H_2. XPS analysis of Ru/WC_x_H_2. **a,** XPS survey spectrum, **b,** C 1*s* region (red: Ru 3*d*, blue: C=C, purple: C-O), **c,** W 4*f* region (red: WC_x, blue: surface WO_x attributed to naturally formed oxides, purple: W 5*p* 3/2), and **d,** Ru 3*p* region (purple: W 4*p* 1/2).

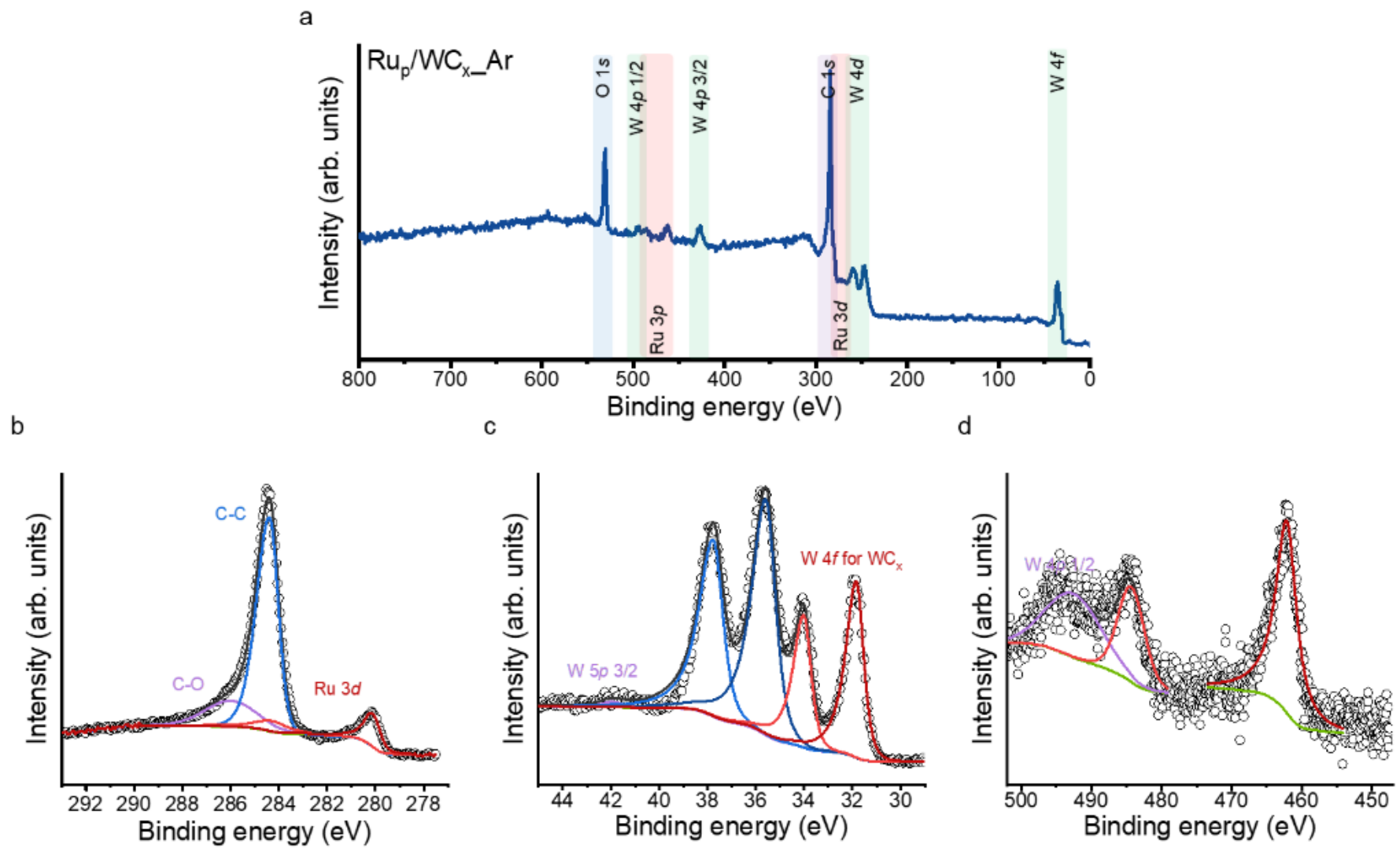

Supplementary Fig. 54 | Electronic structure of Ru_p/WC_x_Ar. XPS analysis of Ru_p/WC_x_Ar. **a,** XPS survey spectrum, **b,** C 1*s* region (red: Ru 3*d*, blue: C=C, purple: C-O), **c,** W 4*f* region (red: WC_x, blue: surface WO_x attributed to naturally formed oxides, purple: W 5*p* 3/2), and **d,** Ru 3*p* region (purple: W 4*p* 1/2).

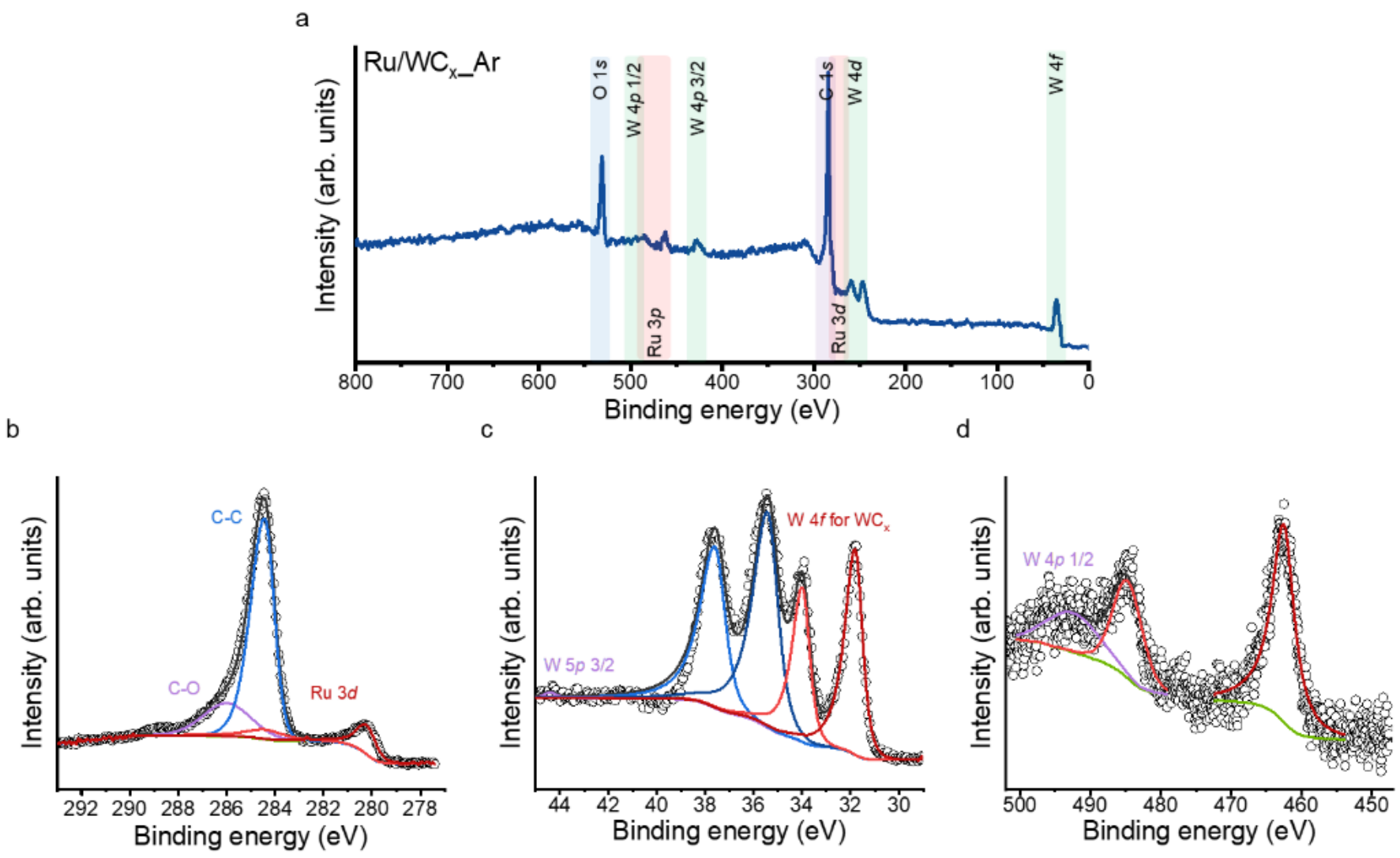

Supplementary Fig. 55 | Electronic structure of Ru/WC$_x$_Ar. XPS analysis of Ru/WC$_x$_Ar. **a,** XPS survey spectrum, **b,** C 1*s* region (red: Ru 3*d*, blue: C=C, purple: C-O), **c,** W 4*f* region (red: WC$_x$, blue: surface WO$_x$ attributed to naturally formed oxides, purple: W 5*p* 3/2), and **d,** Ru 3*p* region (purple: W 4*p* 1/2).

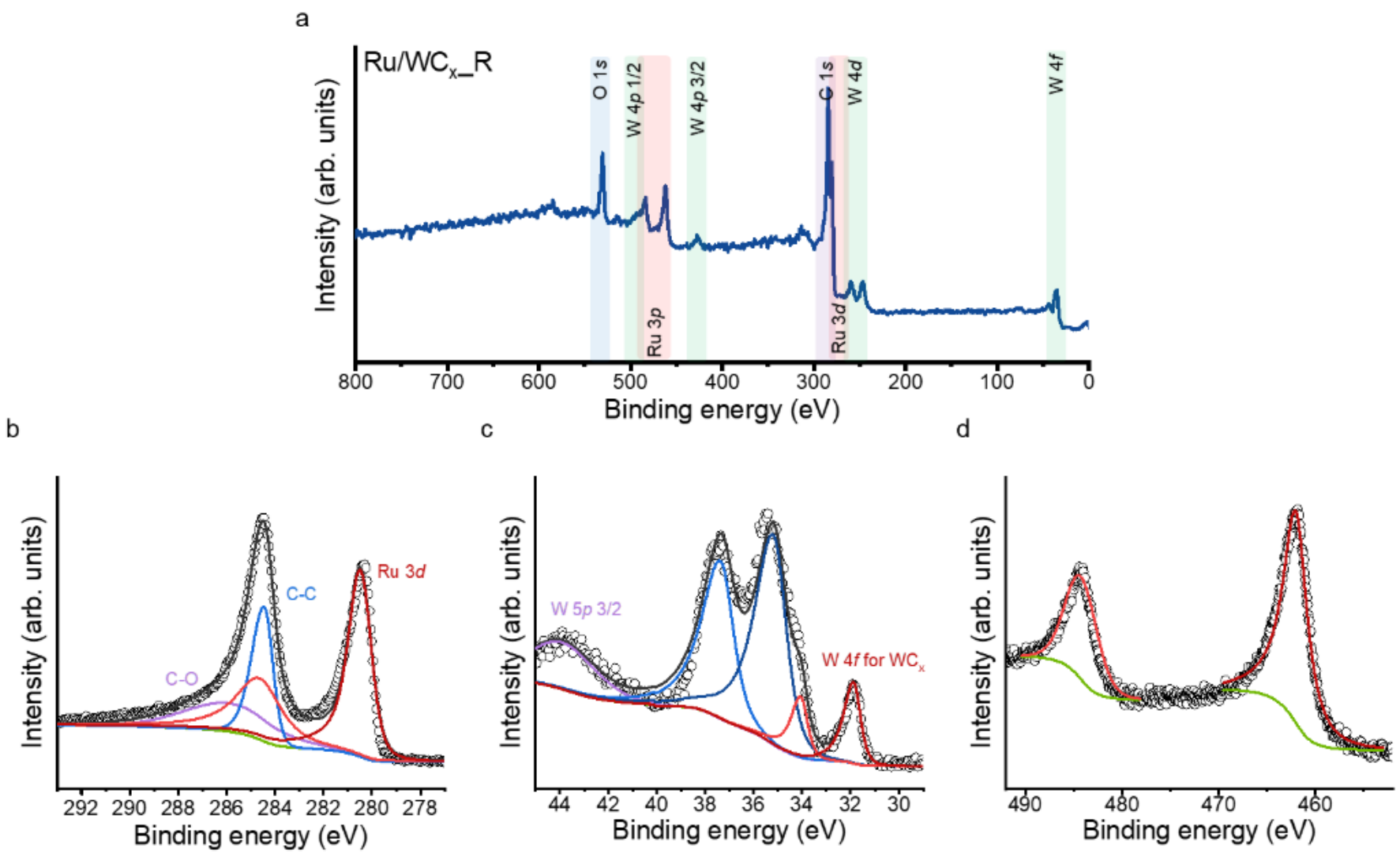

Supplementary Fig. 56 | Electronic structure of Ru/WC$_x$_R. XPS analysis of Ru/WC$_x$_R. **a,** XPS survey spectrum, **b,** C 1*s* region (red: Ru 3*d*, blue: C=C, purple: C-O), **c,** W 4*f* region (red: WC$_x$, blue: surface WO$_x$ attributed to naturally formed oxides, purple: W 5*p* 3/2), and **d,** Ru 3*p* region (purple: W 4*p* 1/2).

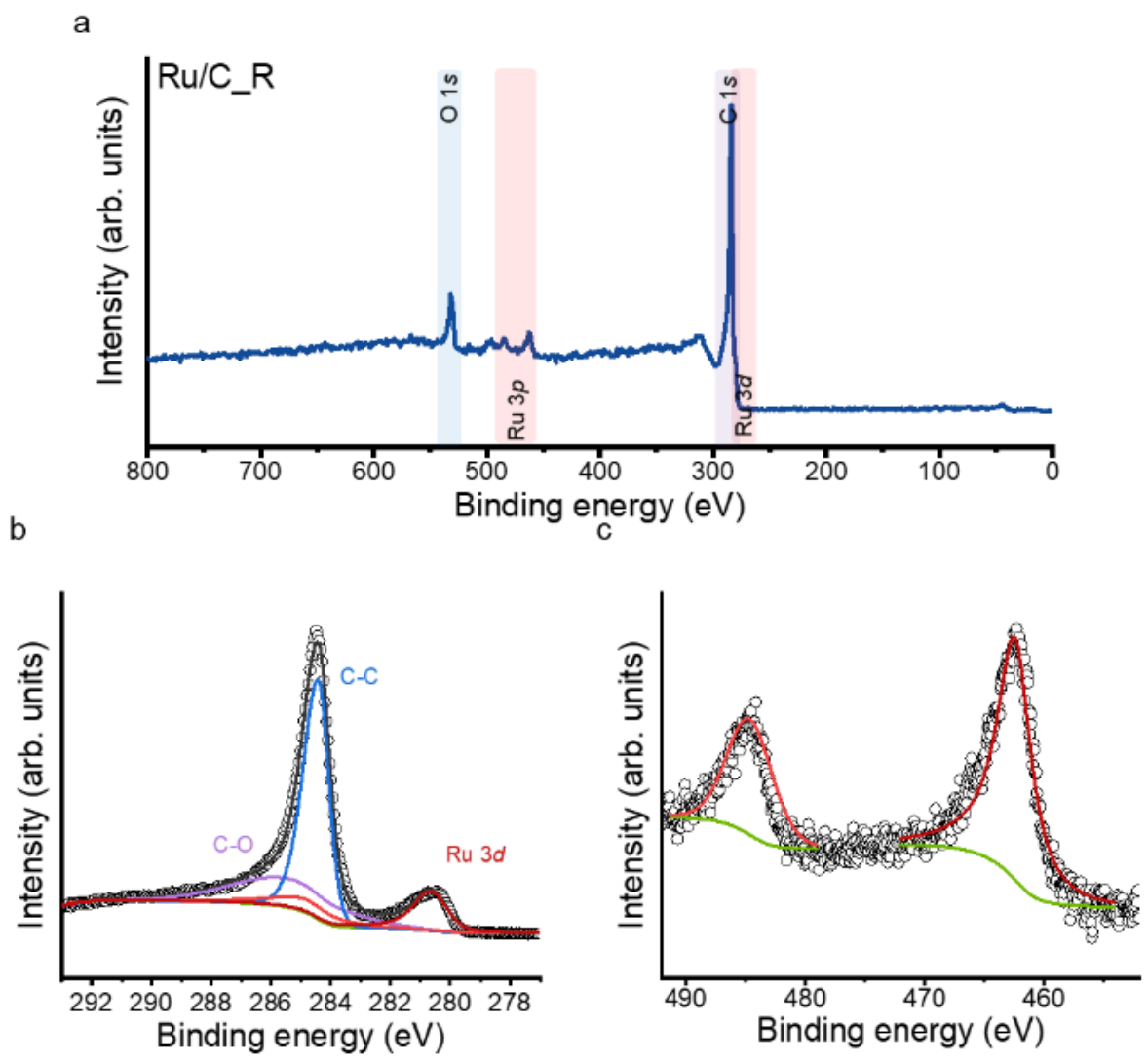

Supplementary Fig. 57 | Electronic structure of Ru/C_R. XPS analysis of Ru/C_R. **a,** XPS survey spectrum, **b,** C 1*s* region (red: Ru 3*d*, blue: C=C, purple: C-O), and **c,** Ru 3*p* region.

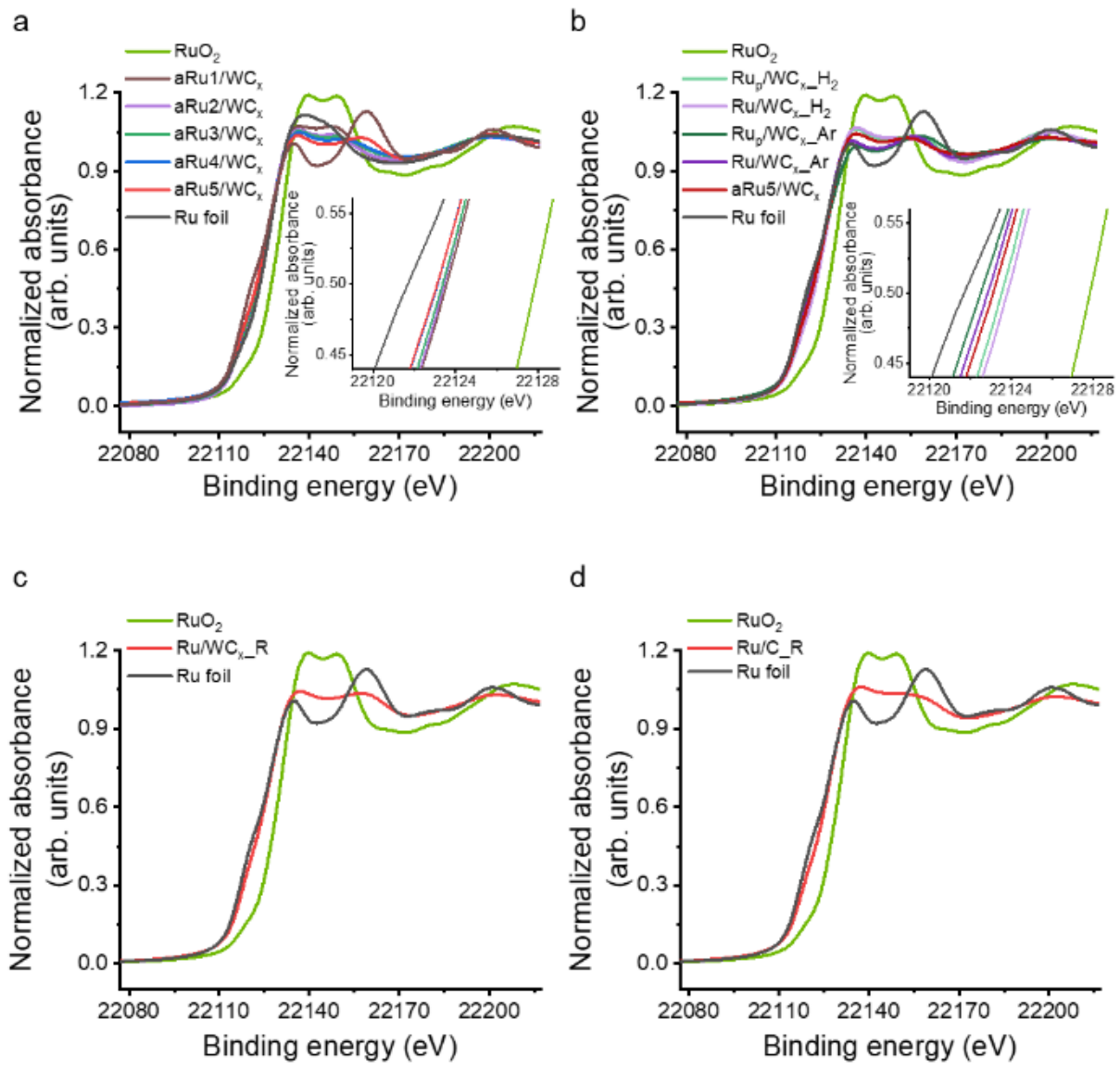

Supplementary Fig. 58 | XANES spectra. a-d, X-ray absorption near-edge structure (XANES) spectra at the Ru K-edge: (**a**) samples prepared by self-assembly using HPB Ru, (**b**) aRu5/WC_x, samples prepared by IWI using HPL Ru and HPB Ru, (**c**) Ru/WC_x_R, and (**d**) Ru/C_R. Spectra are compared with Ru foil (black) and RuO_2 (yellow-green) as references. Insets in a and b show enlarged views of the selected regions of the corresponding main panels.

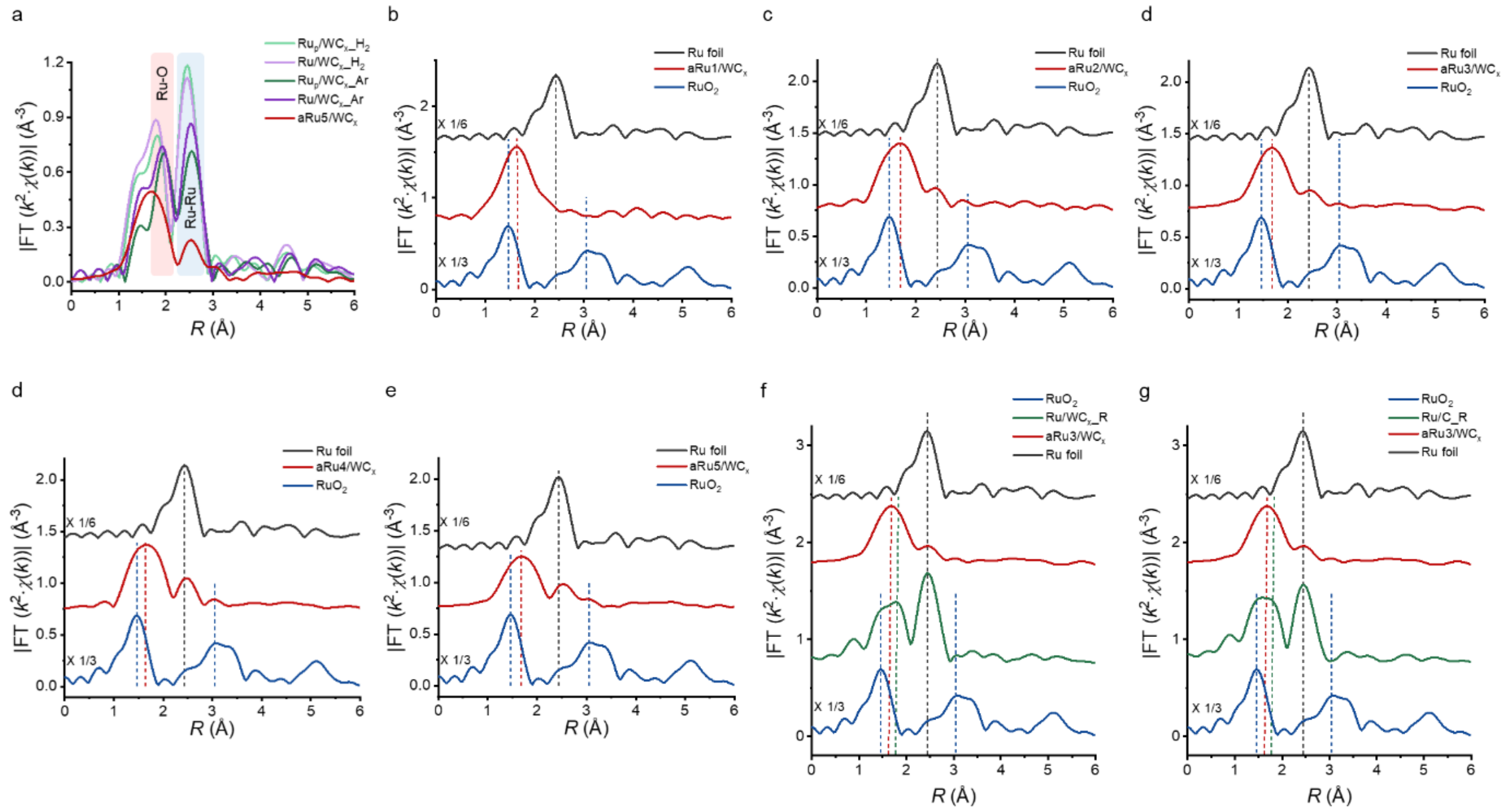

Supplementary Fig. 59 | k^2-weighted FT-EXAFS magnitude in R space ($\chi(R)$). a-h, k^2-weighted Fourier-transformed extended X-ray absorption fine structure (FT-EXAFS) magnitude ($\chi(R)$) at the Ru K-edge for (**a**) aRu5/WC$_x$, samples prepared by IWI using HPL Ru and HPB Ru, (**b**) aRu1/WC$_x$, (**c**) aRu2/WC$_x$, (**d**) aRu3/WC$_x$, (**e**) aRu4/WC$_x$, (**f**) aRu5/WC$_x$, (**g**) Ru/WC$_x$_R, and (**h**) Ru/C_R. (*k*: the photoelectron wavenumber, *R*: the radial distance in Fourier-transformed EXAFS *R* space)

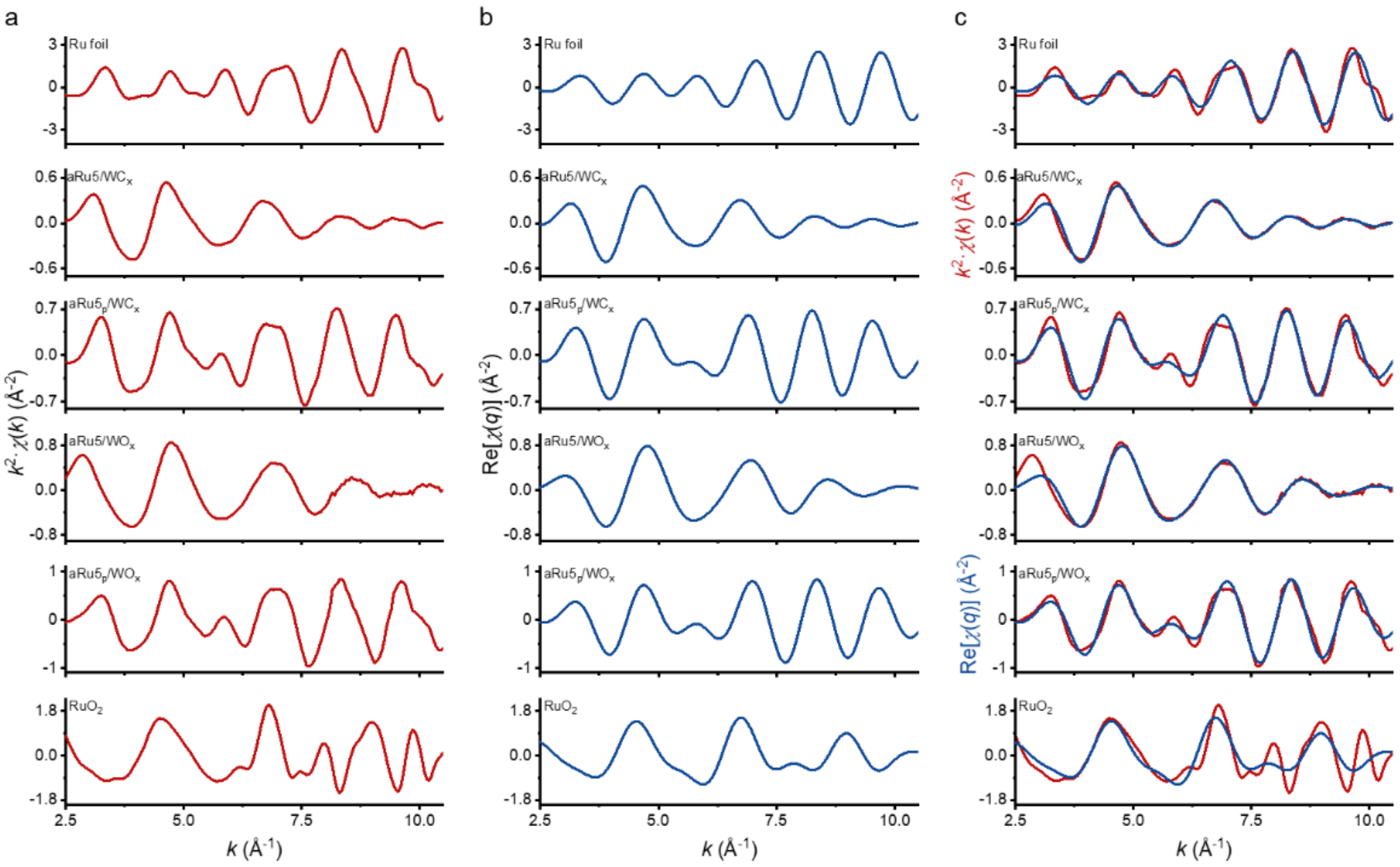

Supplementary Fig. 60 | EXAFS analysis. a, k^2-weighted oscillations ($k^2\chi(k)$) as a function of the photoelectron wavenumber, *k*. **b,** The real part of the back Fourier transform magnitude in *q* space (Re($\chi(q)$)), and **c,** their overlay for samples prepared by self-assembly using HPB Ru (aRu5/WO_x and aRu5/WC_x) and HPL Ru (aRu5$_p$/WO_x and aRu5$_p$/WC_x) with different annealing *T* at the Ru K-edge. (*q*: the photoelectron wavenumber used for the back Fourier-transformed EXAFS oscillations)

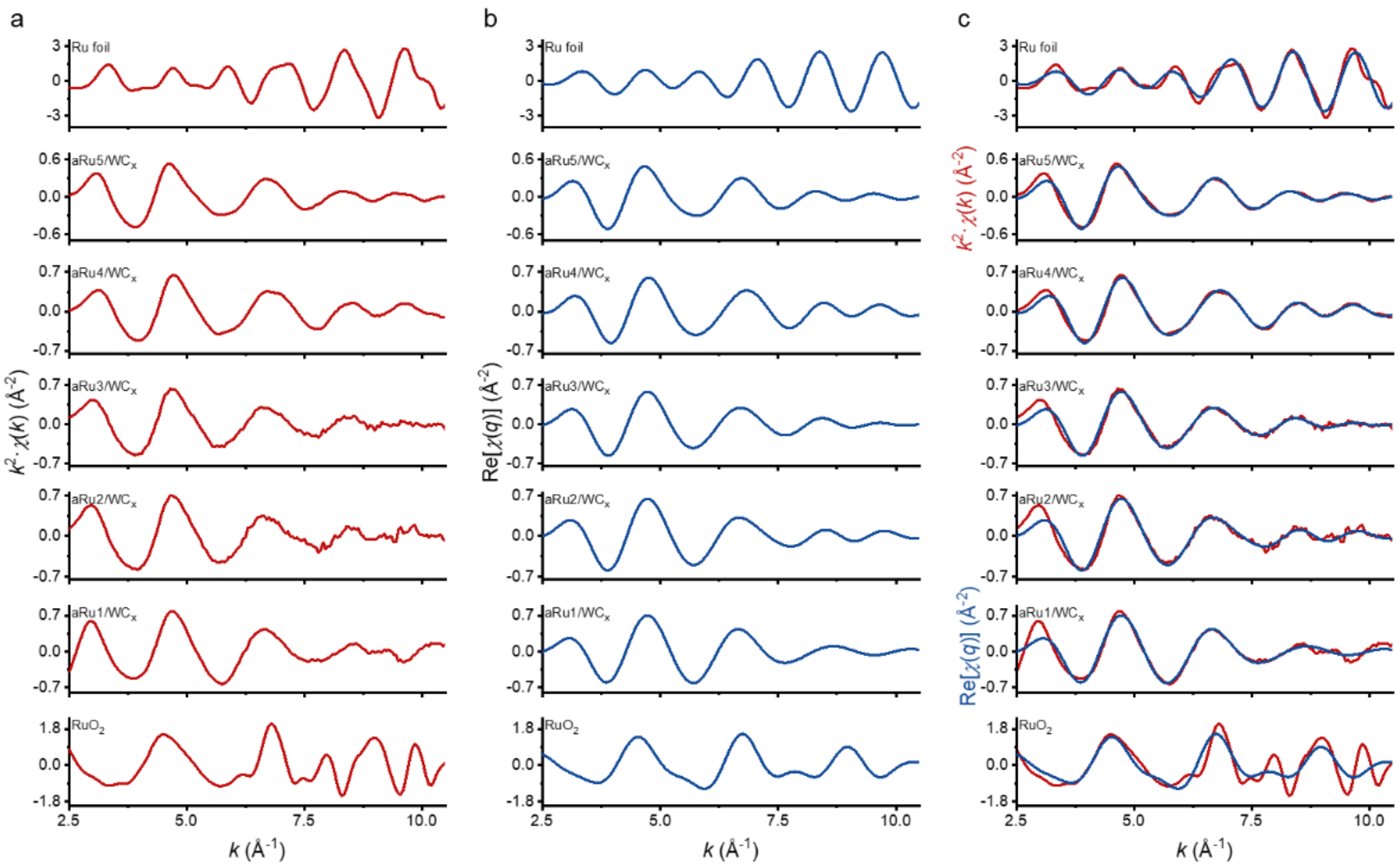

Supplementary Fig. 61 | EXAFS analysis of aRu*N*/WC$_x$ at the Ru K-edge. a, $k^2\chi(k)$ oscillations as a function of the photoelectron wavenumber, *k*. **b,** real part of back Fourier transform magnitude in *q* space (Re($\chi(q)$)), and **c,** their overlay. (*q*: the photoelectron wavenumber used for the back Fourier-transformed EXAFS oscillations)

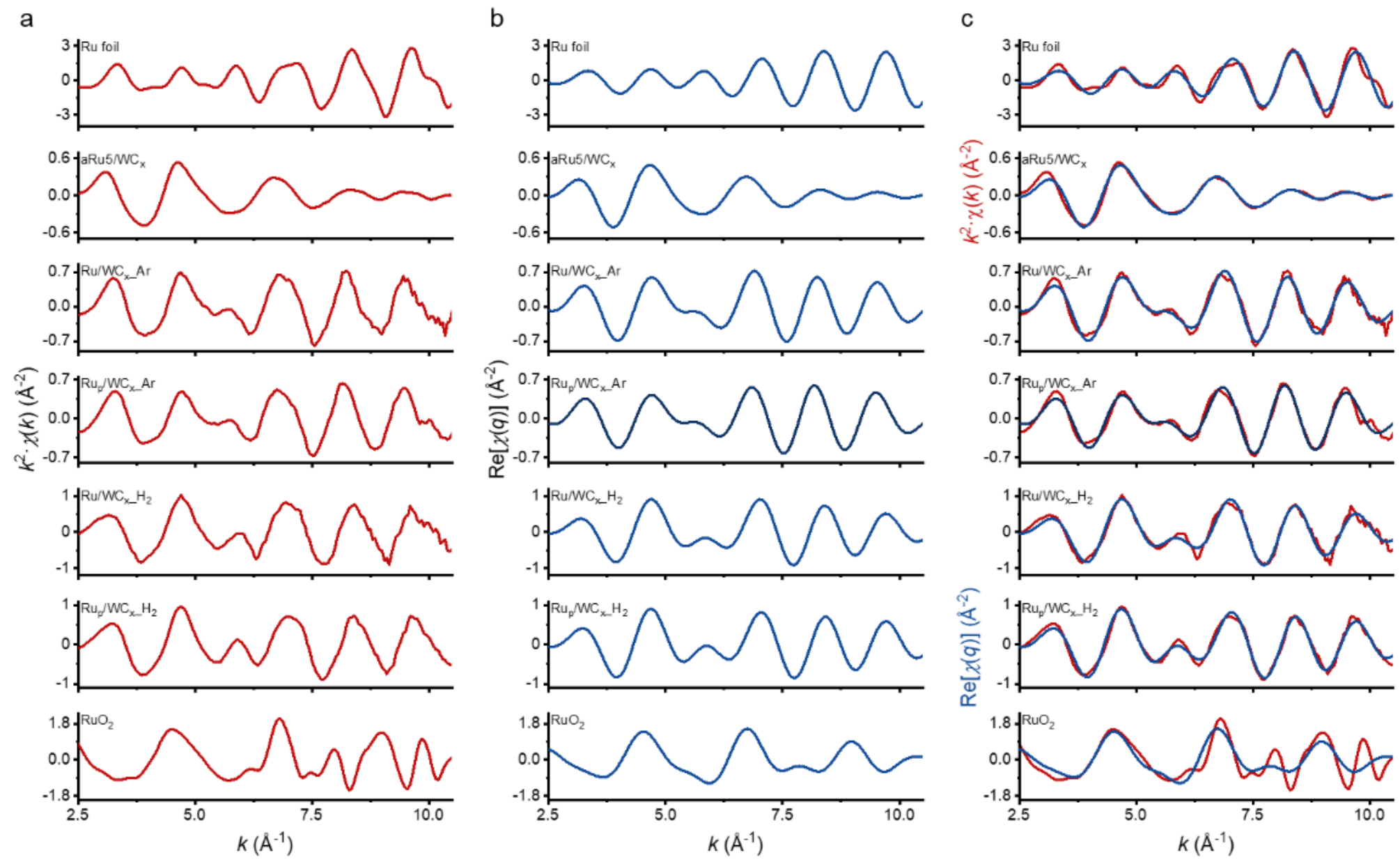

Supplementary Fig. 62 | EXAFS analysis of samples prepared by IWI at the Ru K-edge. a, $k^2\chi(k)$ oscillations as a function of the photoelectron wavenumber, k. **b,** real part of back Fourier transform magnitude in q space (Re($\chi(q)$)), and **c,** their overlay for samples prepared by IWI using HPB Ru (Ru/WC$_x$_H$_2$ and Ru/WC$_x$_Ar) and HPL Ru (Ru$_p$/WC$_x$_H$_2$ and Ru$_p$/WC$_x$_Ar). (q: the photoelectron wavenumber used for the back Fourier-transformed EXAFS oscillations)

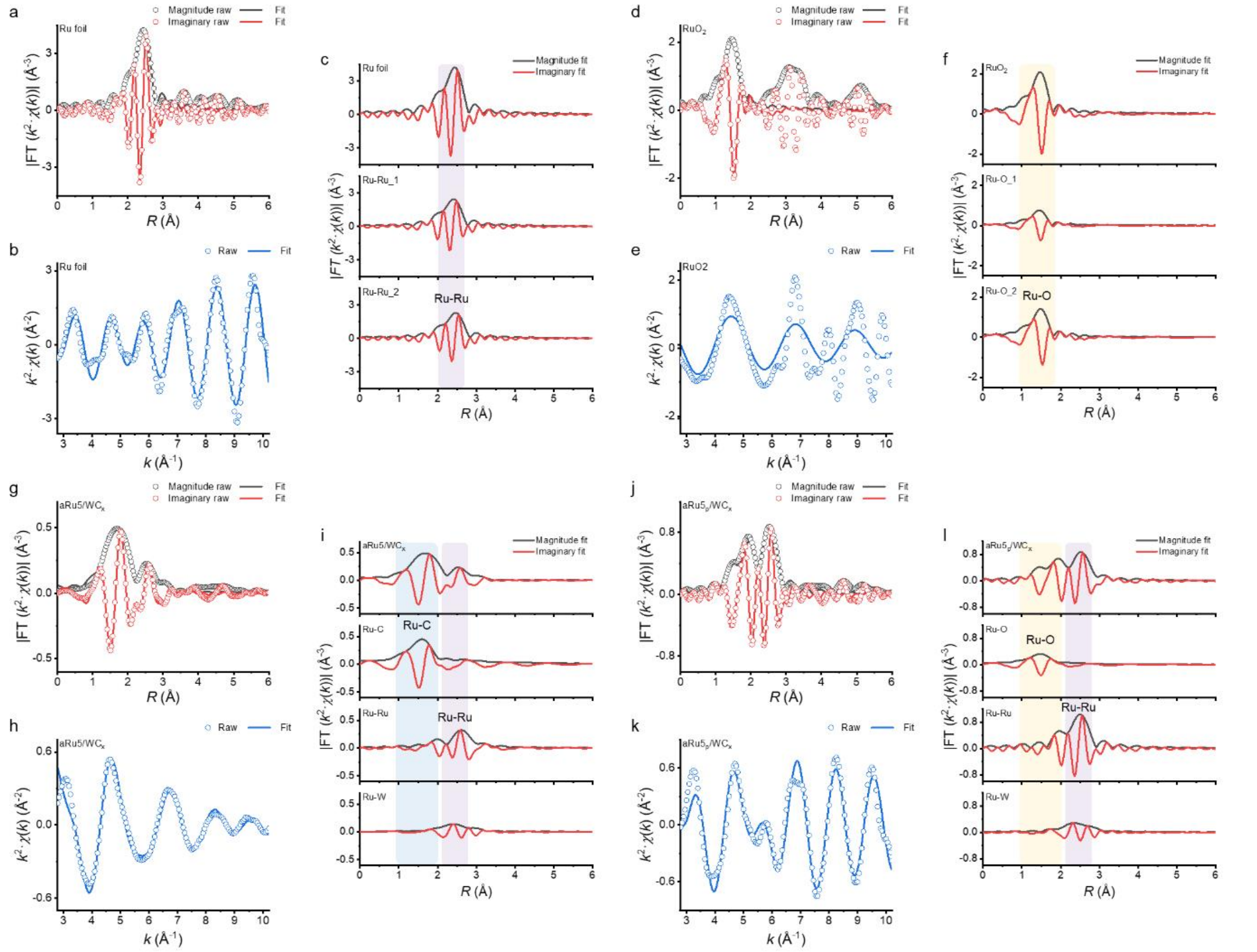

Supplementary Fig. 63 | EXAFS experimental and fitted data. a-l, EXAFS experimental (dotted line) and fitted data (solid line) at the Ru K-edge for (**a-c**) Ru foil, (**d-f**) RuO_2, (**g-i**) aRu5/WC_x, and (**j-l**) $aRu5_p/WC_x$ in (**a,d,g,j**) *R* space, and (**b,e,h,k**) *k* space. (**c,f,i,l**) Deconvoluted path for fitted data. (*k*: the photoelectron wavenumber, *R*: the radial distance in Fourier-transformed EXAFS *R* space)

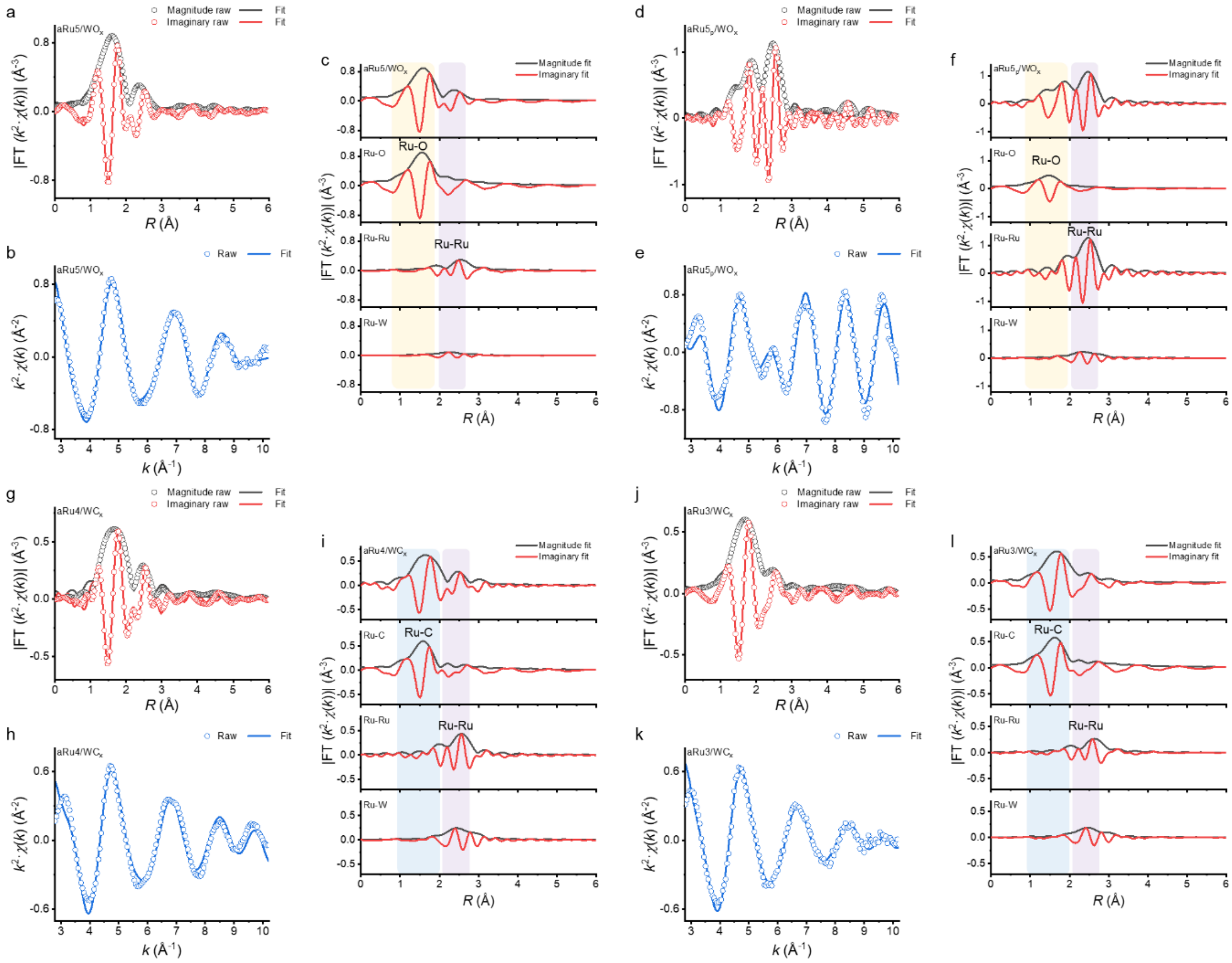

Supplementary Fig. 64 | EXAFS experimental and fitted data. a-l, EXAFS experimental (dotted line) and fitted data (solid line) at the Ru K-edge for (**a-c**) aRu5/WO_x, (**d-f**) aRu5$_p$/WO_x, (**g-i**) aRu4/WC_x, and (**j-l**) aRu3/WC_x in (**a,d,g,j**) *R* space, and (**b,e,h,k**) *k* space. (**c,f,i,l**) Deconvoluted path for fitted data. (*k*: the photoelectron wavenumber, *R*: the radial distance in Fourier-transformed EXAFS *R* space)

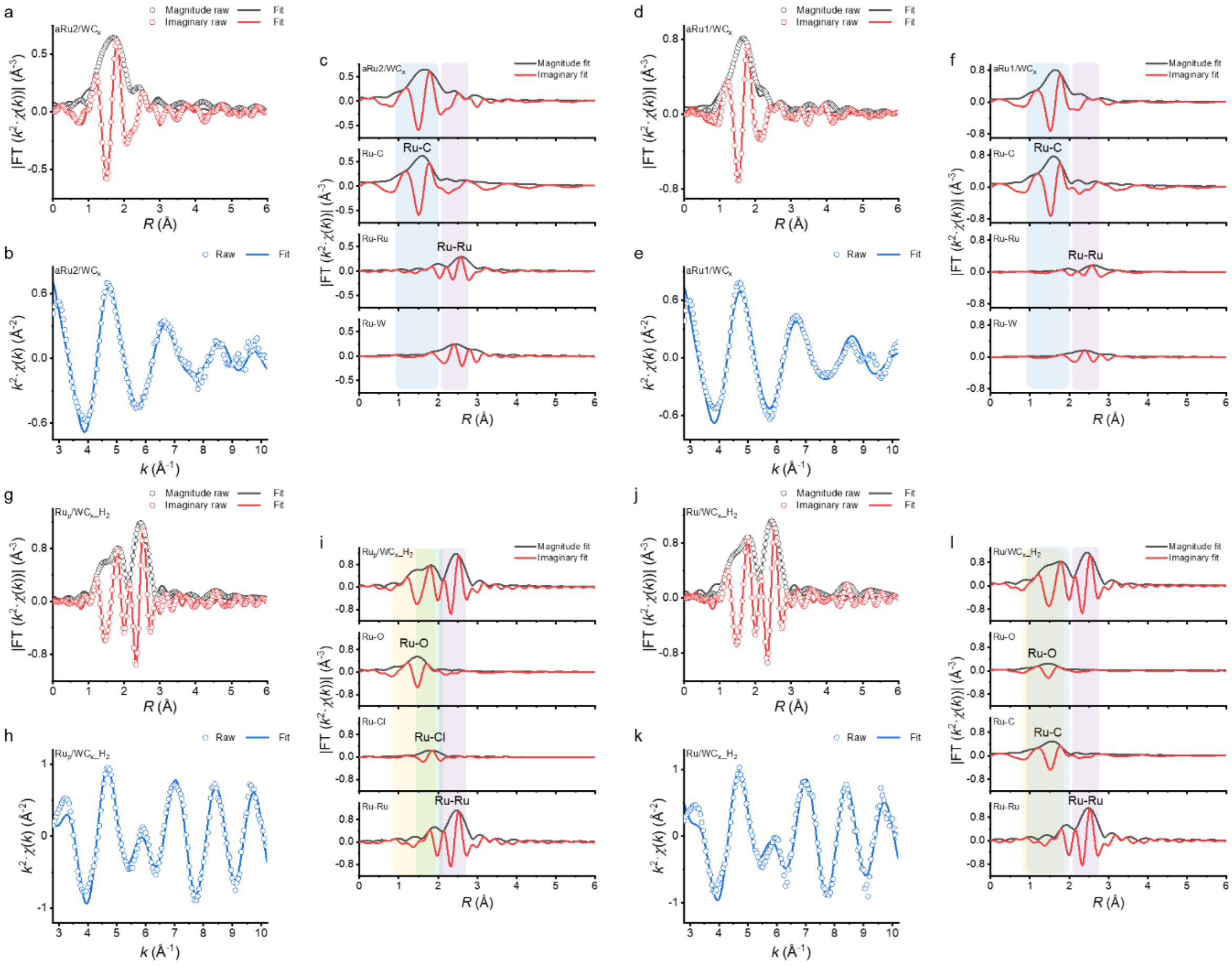

Supplementary Fig. 65 | EXAFS experimental and fitted data. a-l, EXAFS experimental (dotted line) and fitted data (solid line) at the Ru K-edge for (**a-c**) aRu2/WC_x, (**d-f**) aRu1/WC_x, (**g-i**) Ru_p/WC_x_H_2, and (**j-l**) Ru/WC_x_H_2 in (**a,d,g,j**) *R* space, and (**b,e,h,k**) *k* space. (**c,f,i,l**) Deconvoluted path for fitted data. (*k*: the photoelectron wavenumber, *R*: the radial distance in Fourier-transformed EXAFS *R* space)

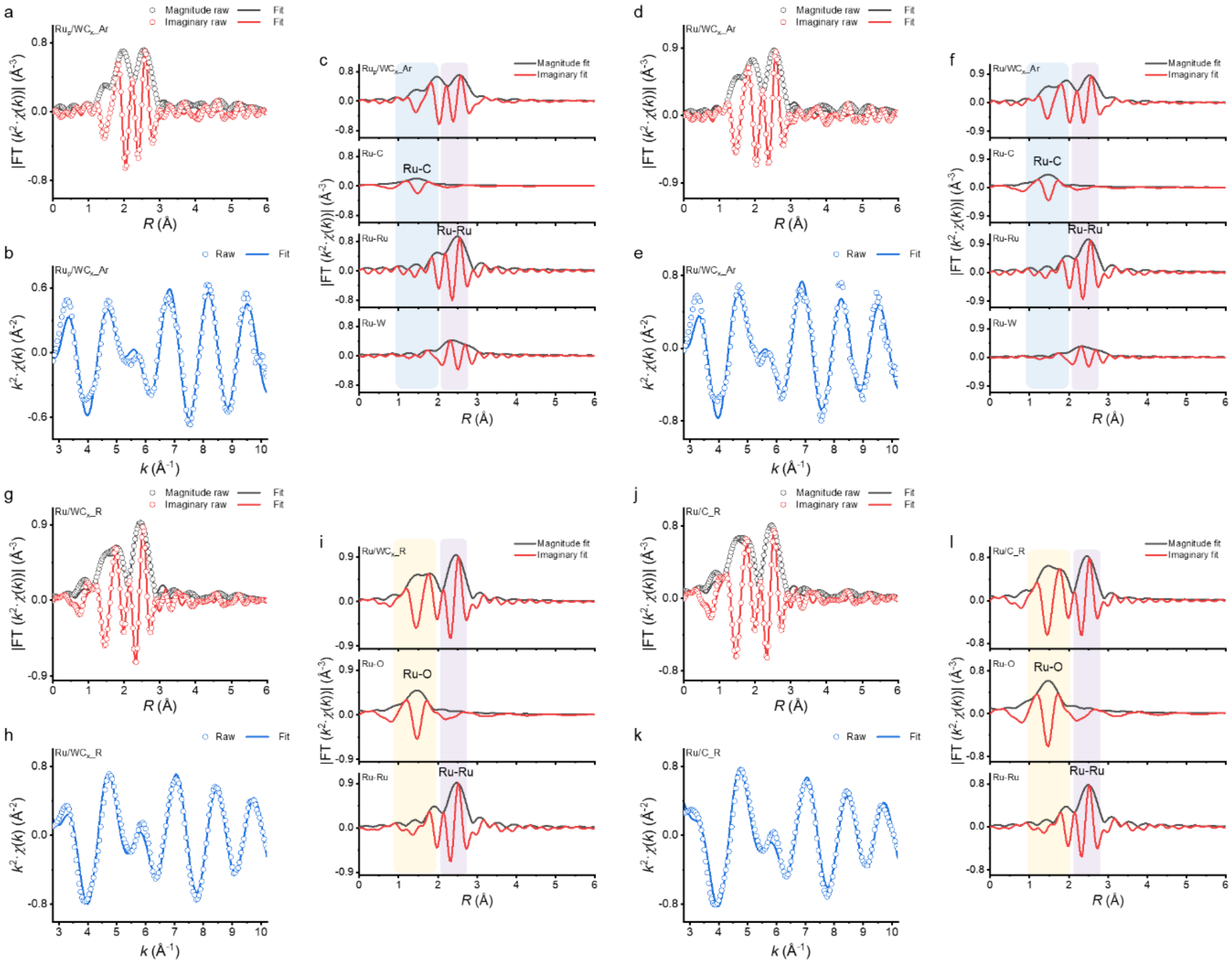

Supplementary Fig. 66 | EXAFS experimental and fitted data. a-l, EXAFS experimental (dotted line) and fitted data (solid line) at the Ru K-edge for (**a-c**) Ru_p/WC_x_Ar, (**d-f**) Ru/WC_x_Ar, (**g-i**) Ru/WC_x_R, and (**j-l**) Ru/C_R in (**a,d,g,j**) *R* space, and (**b,e,h,k**) *k* space. (**c,f,i,l**) Deconvoluted path for fitted data. (*k*: the photoelectron wavenumber, *R*: the radial distance in Fourier-transformed EXAFS *R* space)

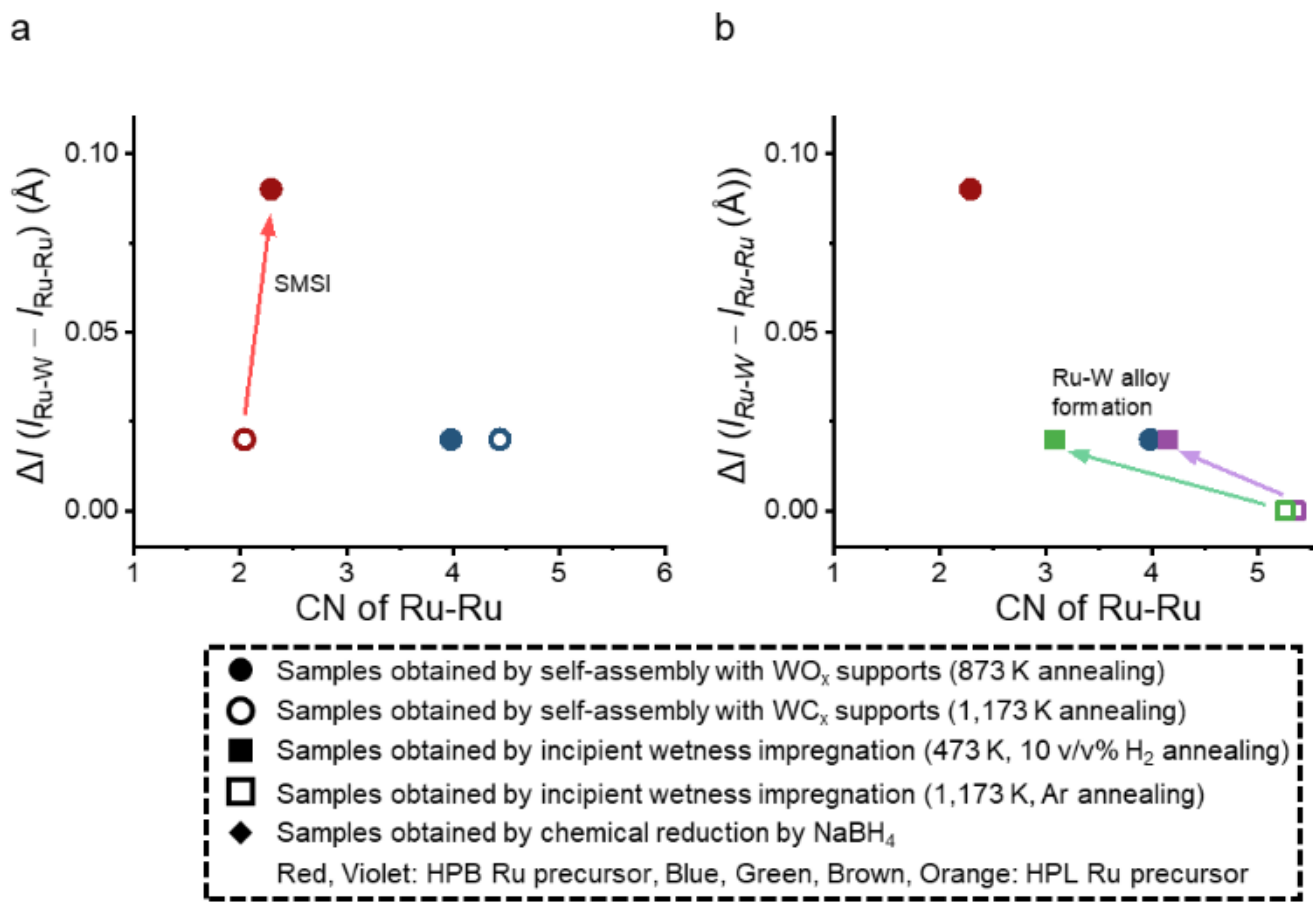

Supplementary Fig. 67 | FT-EXAFS structural parameters. a,b, Bond length difference between Ru-W and Ru-Ru bonds as a function of Ru-Ru bond CN, based on EXAFS fitting results for the second shell at the Ru K-edge for (**a**) samples prepared by self-assembly, and (**b**) samples prepared by IWI, highlighting the effects of annealing conditions. (CN: coordination number, Δ*l*: $l_{Ru\text{-}W} - l_{Ru\text{-}Ru}$, where $l_{Ru\text{-}W}$ and $l_{Ru\text{-}Ru}$ are Ru-W and Ru-Ru bond lengths)

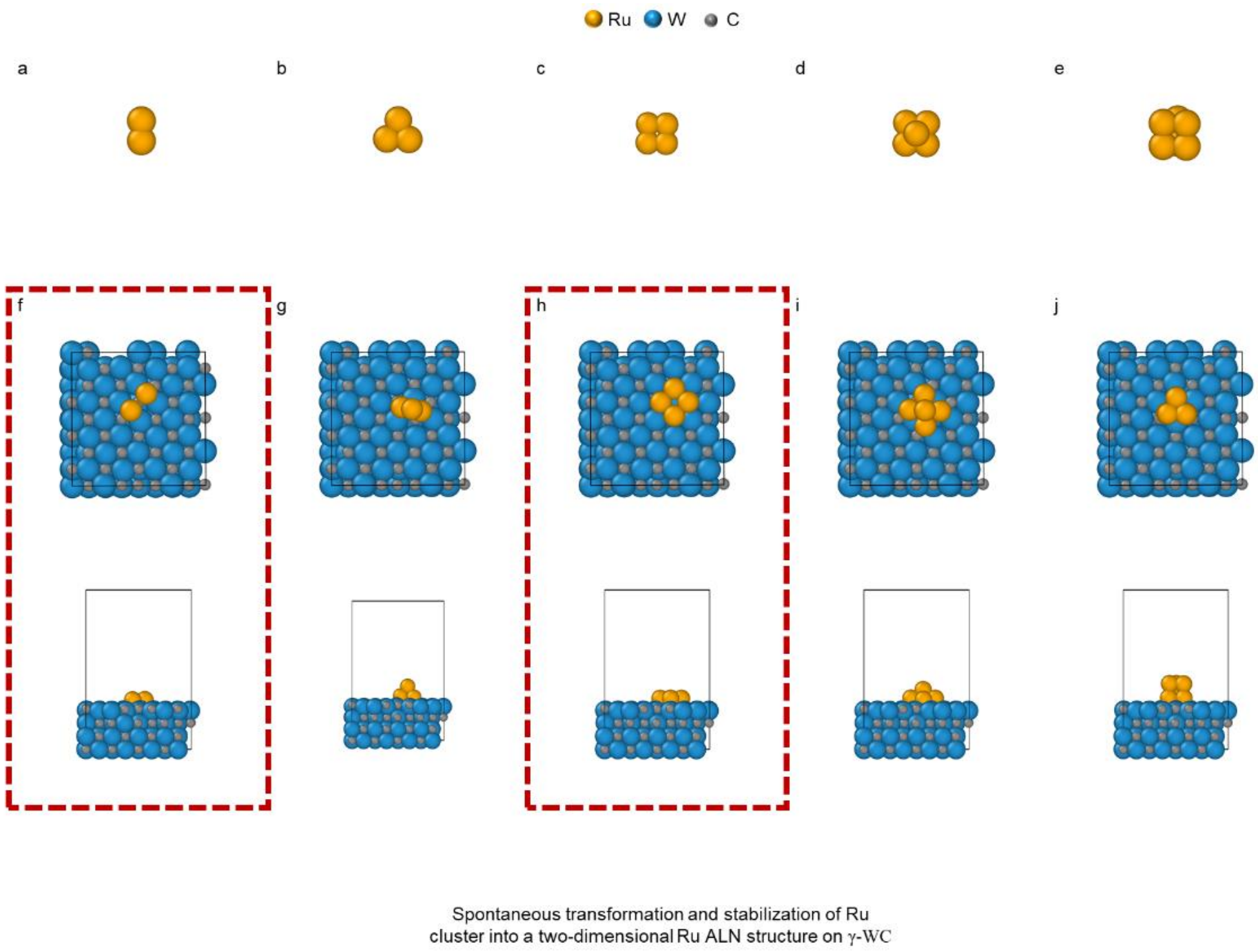

Supplementary Fig. 68 | DFT-optimized structures of three-dimensional Ru_N clusters (N = 2-6) in vacuum and on γ-WC support. a-e, Optimized structures of three-dimensional Ru_N clusters in vacuum. **f-j,** Optimized structures of Ru_N clusters supported on γ-WC support. Red-dashed boxes indicate models that spontaneously evolve two-dimensional Ru ALN configurations during structural optimization, as illustrated in Supplementary Movies 6 and 7.

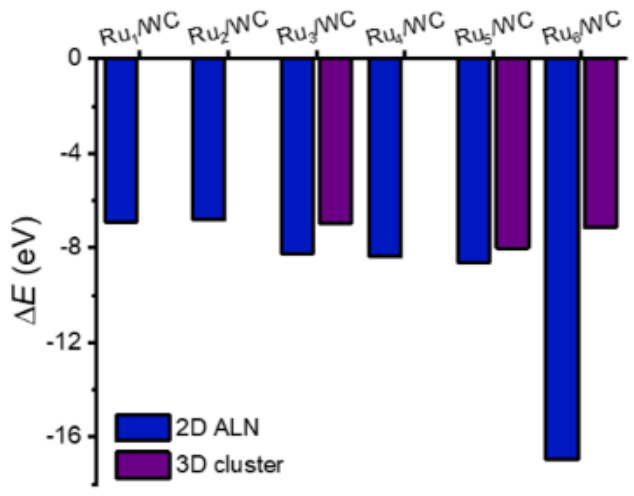

Supplementary Fig. 69 | DFT-calculated energy differences (Δ*E*) of two-dimensional Ru ALN (blue) and three-dimensional Ru clusters (purples) on γ-WC, relative to the corresponding Ru_N clusters in vacuum (N = 1-6).

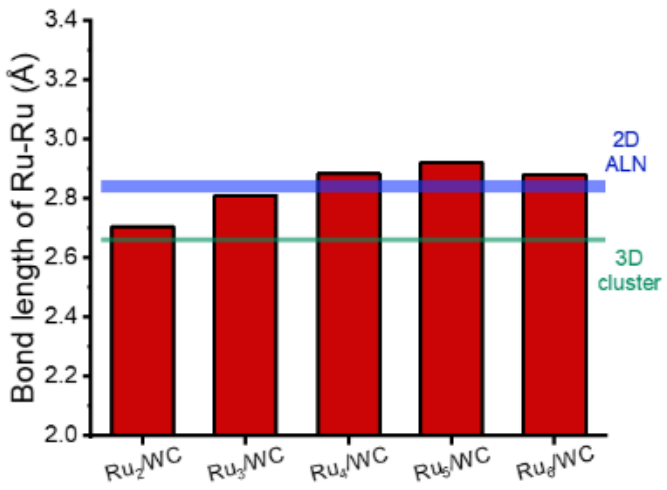

Supplementary Fig. 70 | Average Ru-Ru bond lengths for DFT-optimized RuN/WC (Ru ALNs) models compared with experimentally fitted values. The green shaded region represents the Ru-Ru bond length obtained from EXAFS fitting of Ru nanoparticle samples (Ru_p/WC_x_H_2, Ru/WCx_H_2, Ru/WC_x_R, and Ru/C_R), while the blue shaded region corresponds to that of Ru ALN samples (aRu*N*/WC_x, *N* = 1-5).

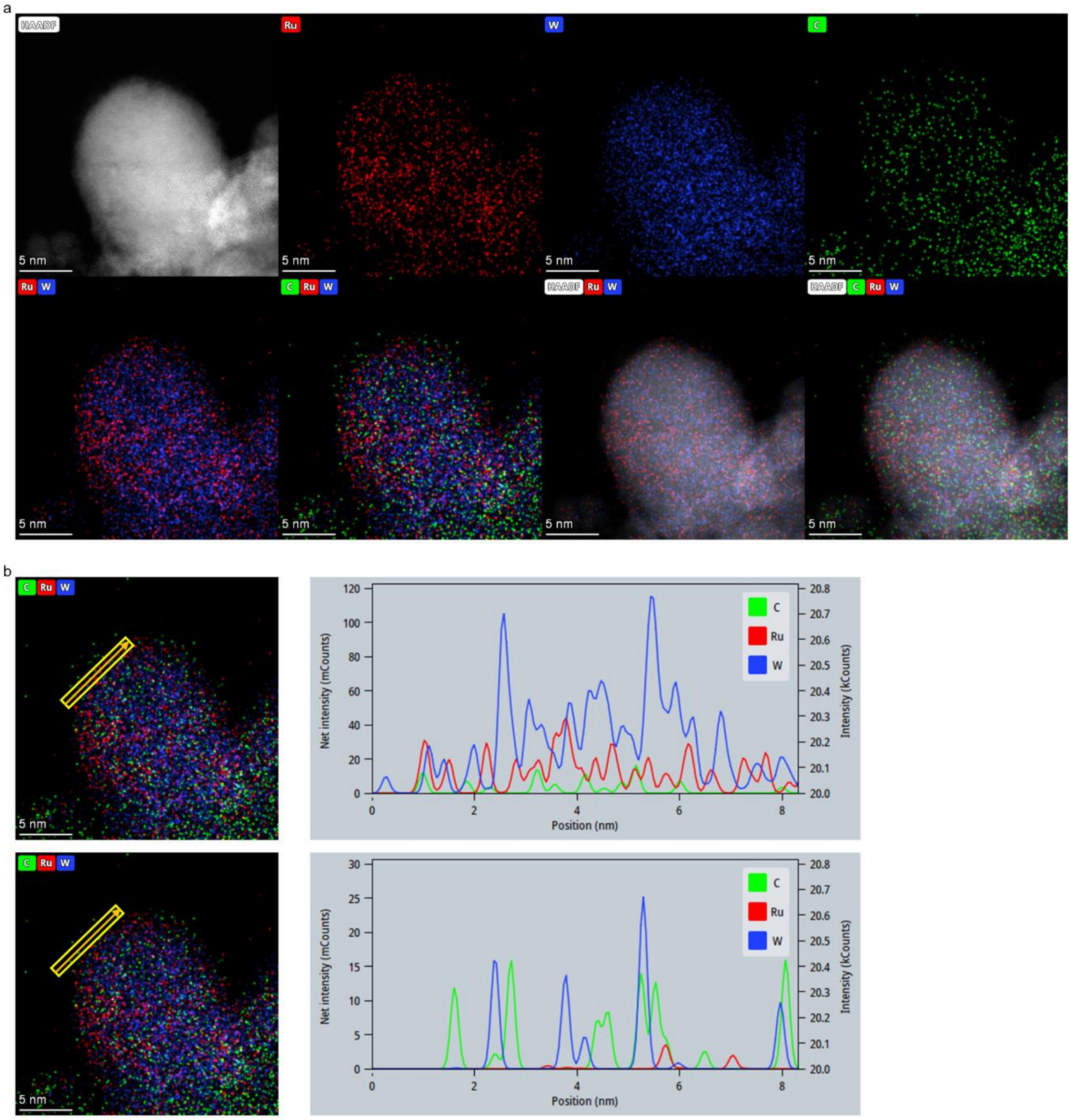

Supplementary Fig. 71 | High-resolution STEM-EDS analysis of selected WC_x particles in aRu3/WC_x. a, HAADF-STEM image and the corresponding EDS elemental mappings of C (green), W (blue), Ru (red) for a representative WC_x particle in aRu3/WC_x, together with the overlaid elemental distributions. **b,** Representative EDS line-scan profiles acquired along the directions indicated by the yellow rectangles

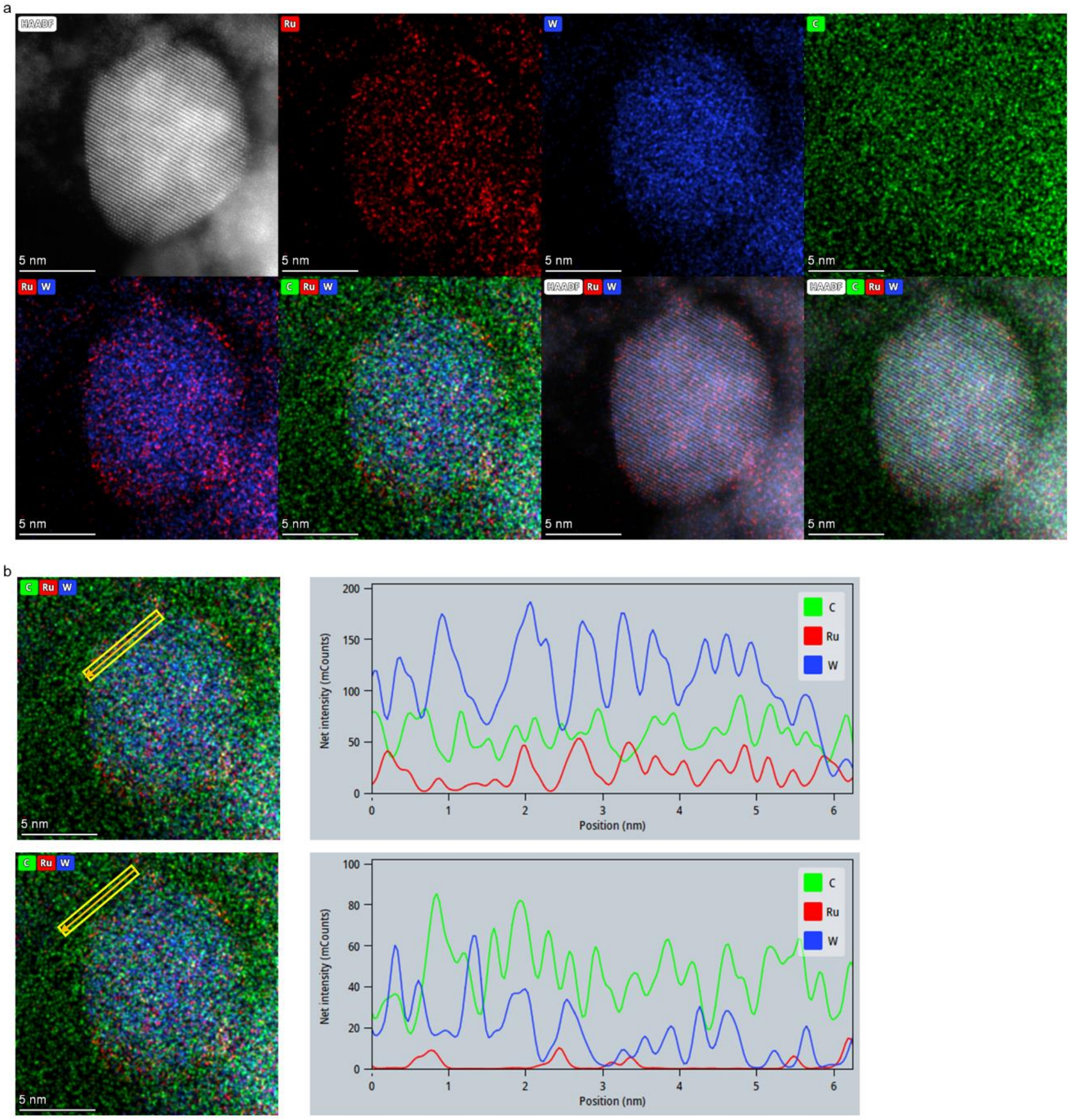

Supplementary Fig. 72 | High-resolution STEM-EDS analysis of selected WC_x particles in aRu3/WC_x. a, HAADF-STEM image and the corresponding EDS elemental mappings of C (green), W (blue), Ru (red) for a representative WC_x particle in aRu3/WC_x, together with the overlaid elemental distributions. **b,** Representative EDS line-scan profiles acquired along the directions indicated by the yellow rectangles

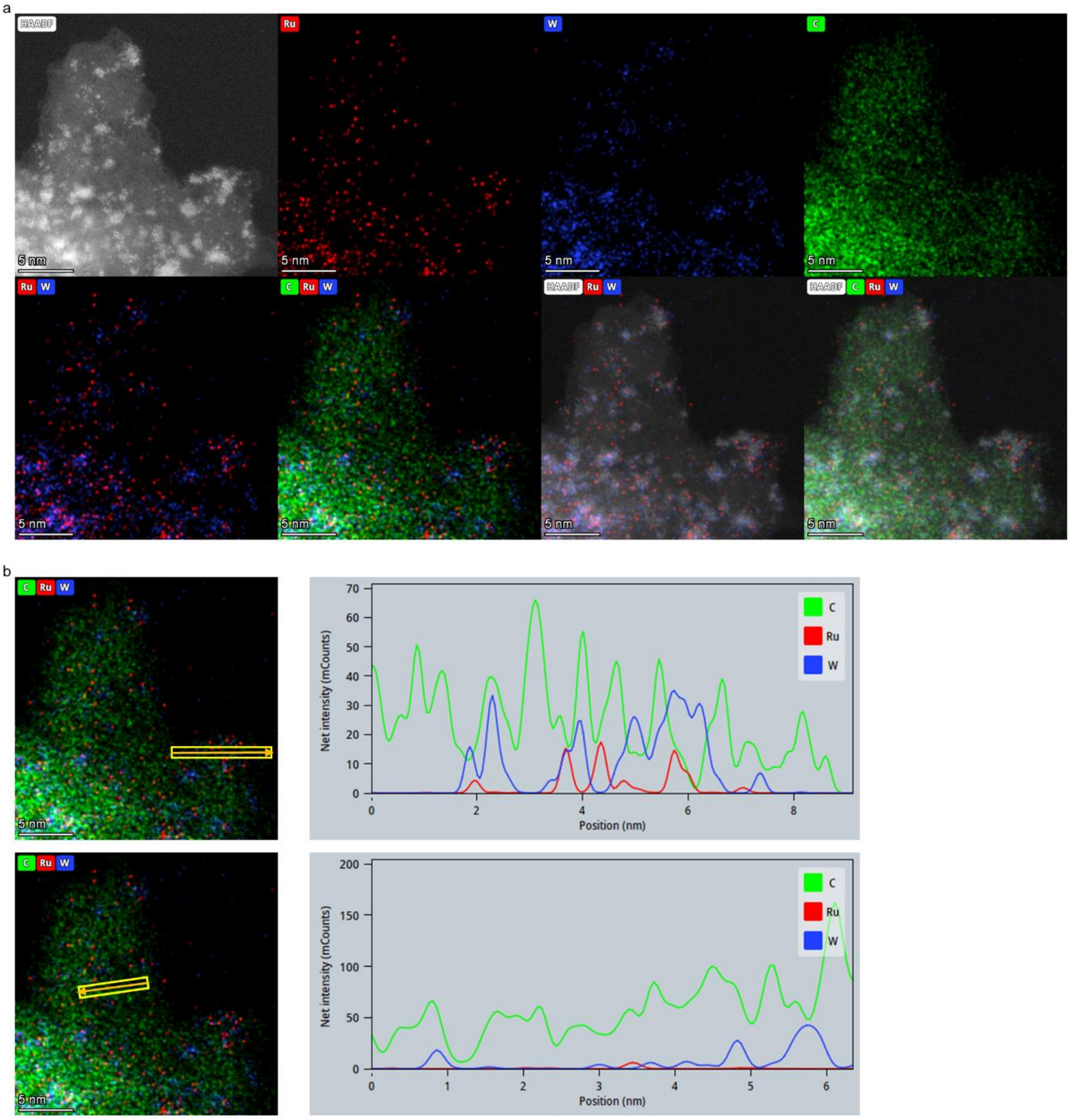

Supplementary Fig. 73 | High-resolution STEM-EDS analysis of selected carbon-rich regions in aRu3/WC$_x$. a, HAADF-STEM image and the corresponding EDS elemental mappings of C (green), W (blue), Ru (red) for a representative carbon-rich region in aRu3/WC$_x$, together with the overlaid elemental distributions. **b,** Representative EDS line-scan profiles acquired along the directions indicated by the yellow rectangles

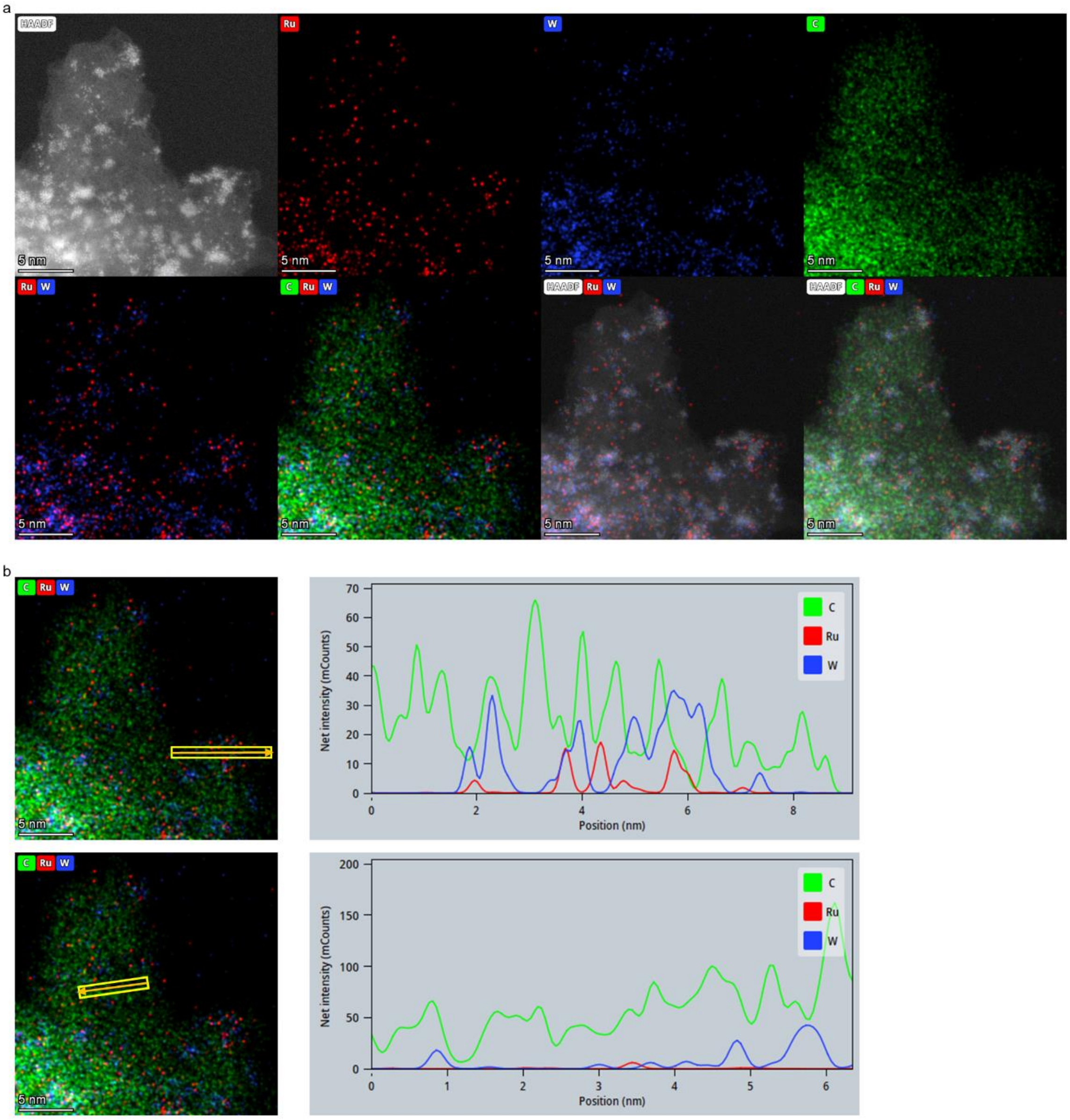

Supplementary Fig. 74 | High-resolution STEM-EDS analysis of selected carbon-rich regions in aRu3/WC_x. a, HAADF-STEM image and the corresponding EDS elemental mappings of C (green), W (blue), Ru (red) for a representative carbon-rich region in aRu3/WC_x, together with the overlaid elemental distributions. **b,** Representative EDS line-scan profiles acquired along the directions indicated by the yellow rectangles

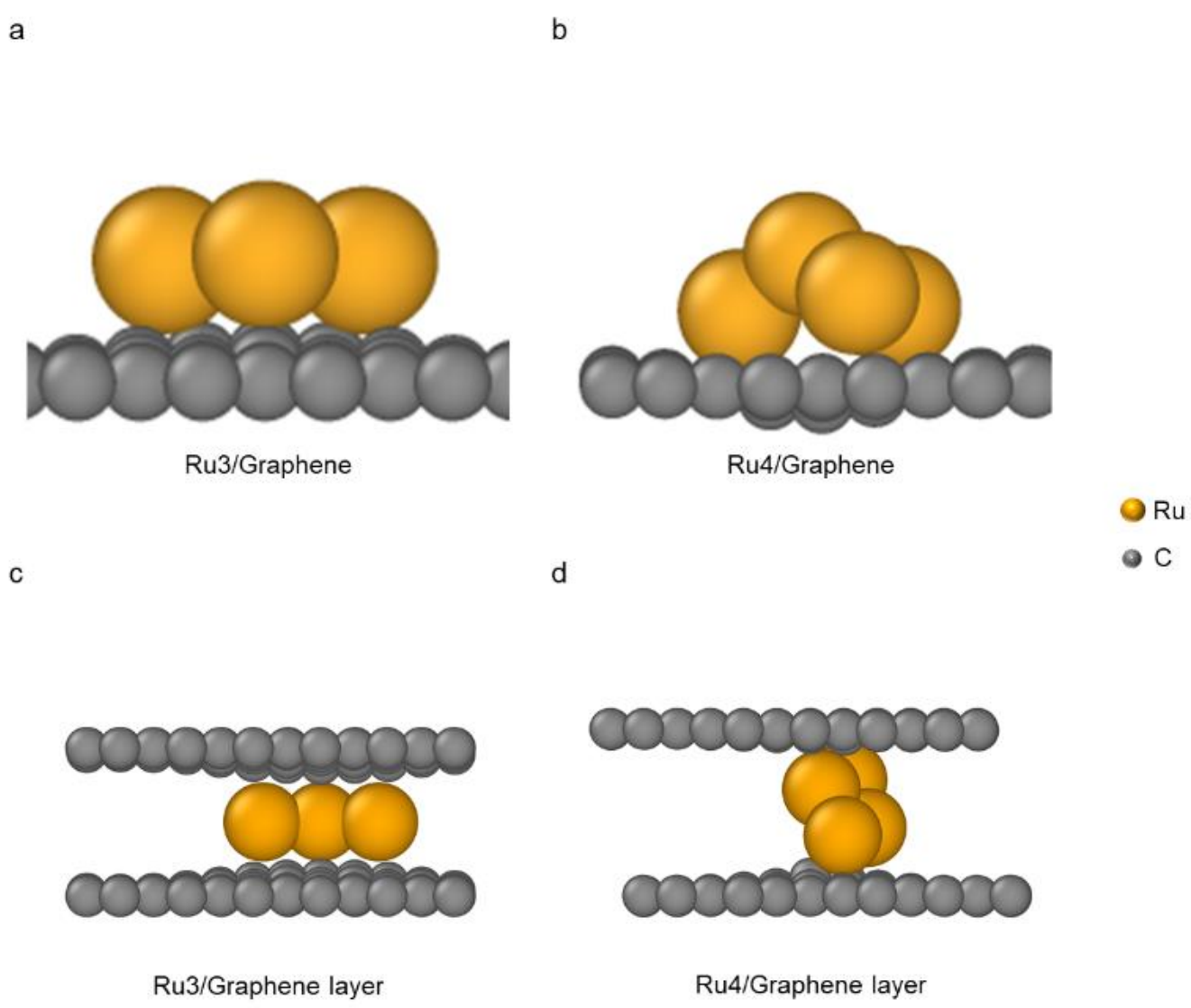

Supplementary Fig. 75 | DFT models representing possible carbon-derived coordination environments of Ru ensembles. **a-b,** DFT-optimized structures of Ru3 and Ru4 ensembles adsorbed on graphene surfaces. **c-d,** DFT-optimized structures of Ru3 and Ru4 ensembles confined between graphene layers.

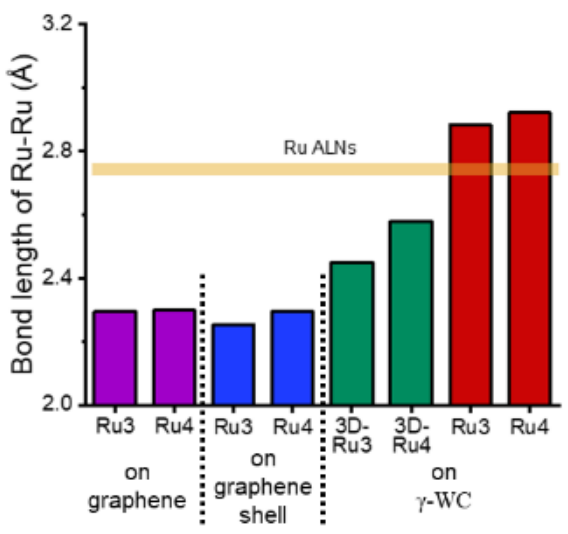

Supplementary Fig. 76 | Comparison of Ru-Ru bond lengths for Ru ensembles stabilized in different coordination environments. Average Ru-Ru bond lengths obtained from DFT-optimized Ru3 and Ru4 ensembles adsorbed on graphene surface (purple), Ru3 and Ru4 ensembles confined between graphene layers (blue), three-dimensional Ru3 and Ru4 ensembles on γ-WC supports (green), and Ru3 and Ru4 ALN stabilized on γ-WC supports (red). The orange shaded region represents the experimentally fitted Ru-Ru bond length range of the Ru ALN/WC_x samples (aRu*N*/WC_x).

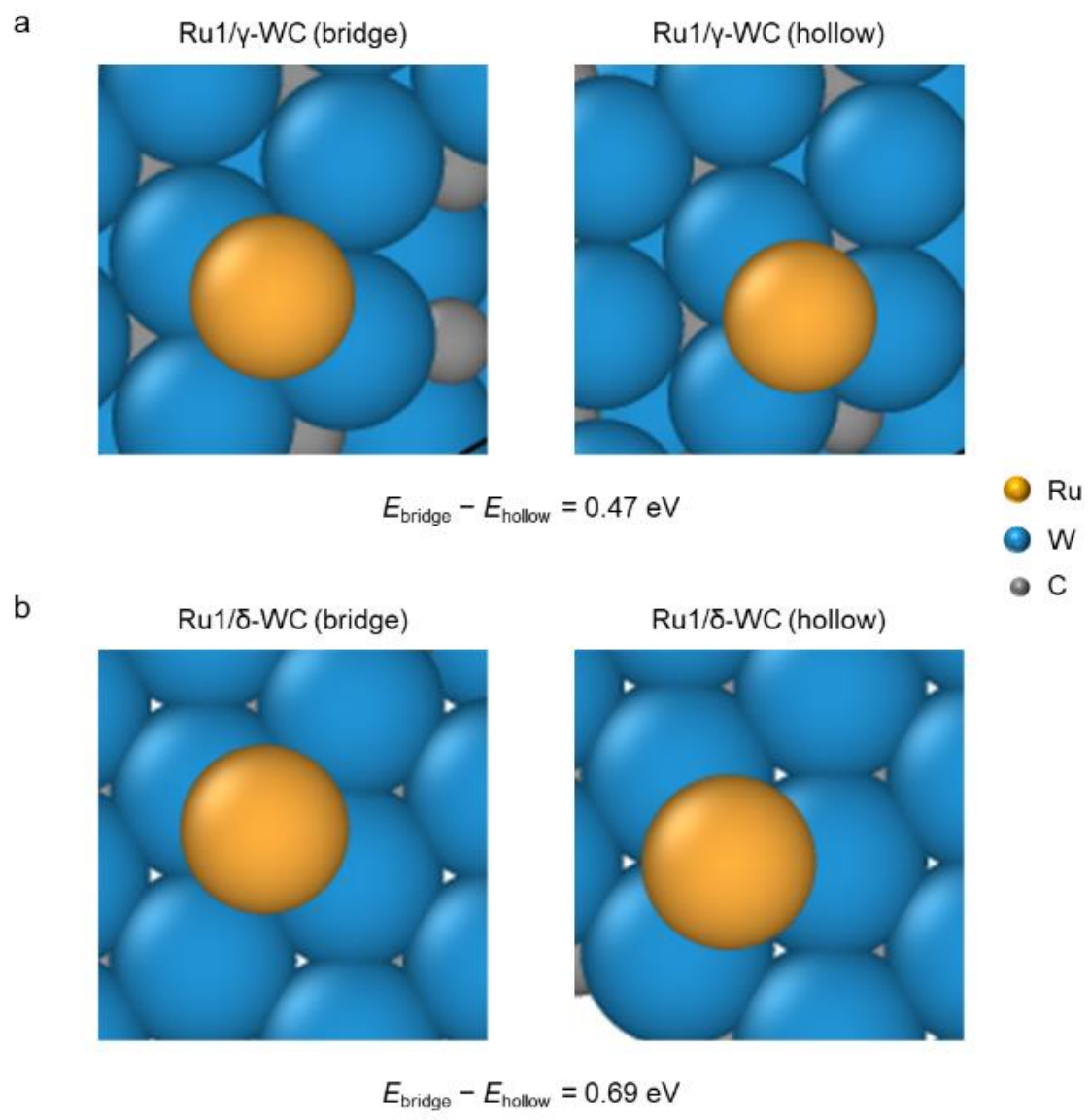

Supplementary Fig. 77 | Energetic preference of Ru single atoms on γ-WC or δ-WC surfaces. a-b, DFT-optimized structures of Ru single atoms adsorbed at bridge and hollow sites on (**a**) γ-WC, and (**b**) δ-WC supports, together with the corresponding free energy differences ($E_{bridge} - E_{hollow}$).

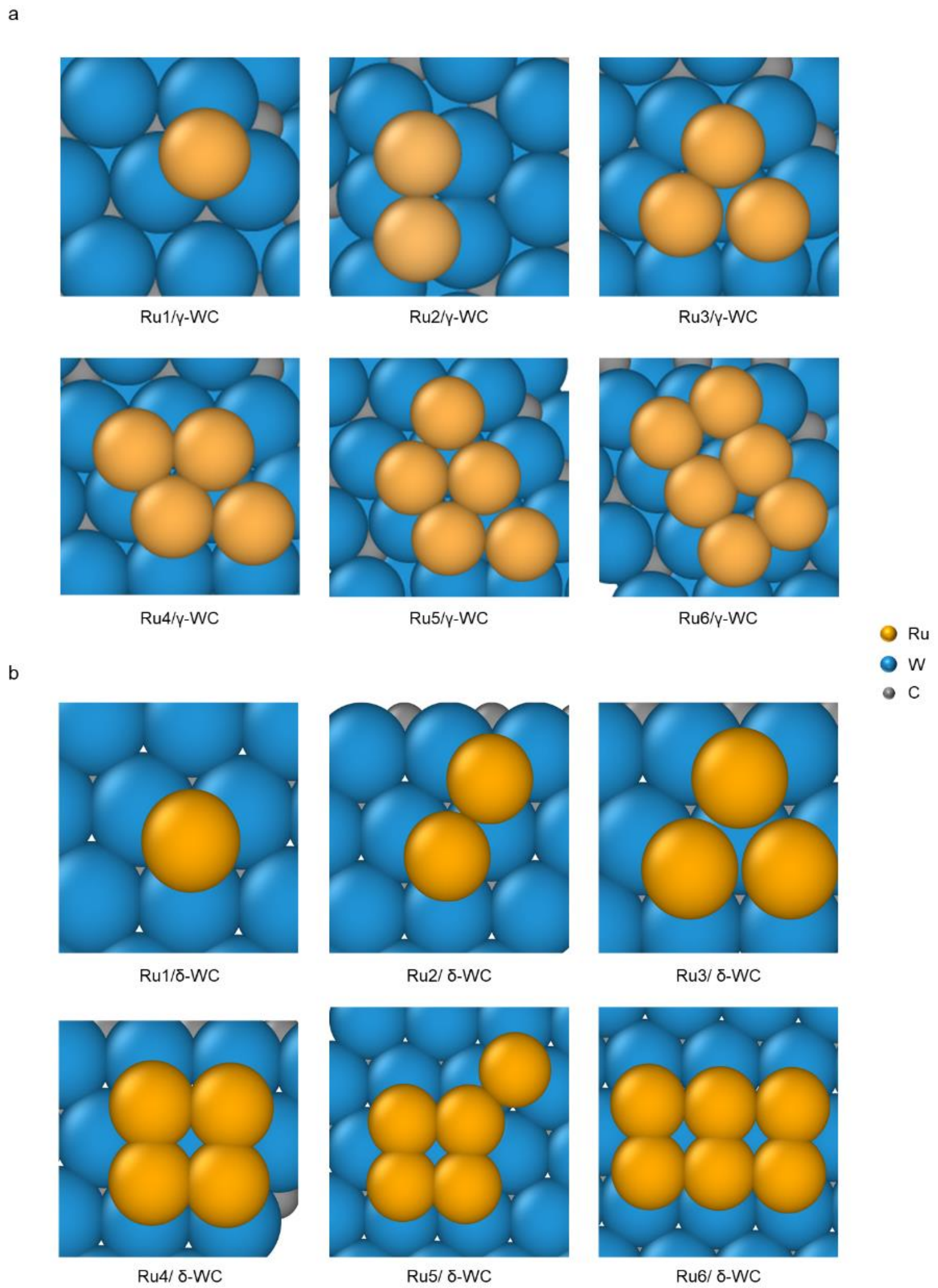

Supplementary Fig. 78 | DFT-optimized structures of Ru ensemble structures on γ-WC or δ-WC surfaces. a-b, DFT-optimized structures of Ru1, Ru2, Ru3, Ru4, Ru5, and Ru6 ensembles adsorbed on (**a**) γ-WC, and (**b**) δ-WC supports. Representative adsorption-site stability comparisons between bridge and hollow configurations are provided for Ru single atoms in Supplementary Fig. 77.

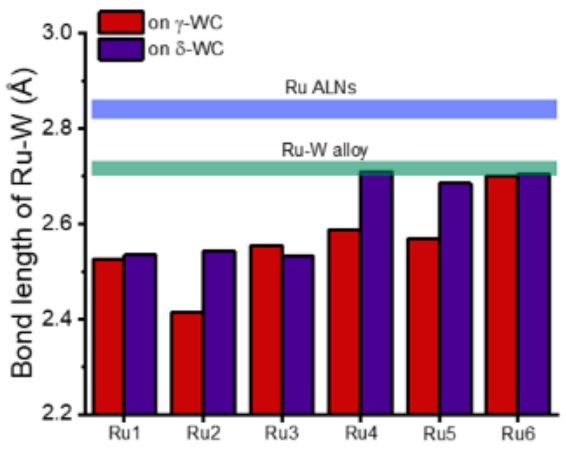

Supplementary Fig. 79 | Comparison of Ru-W coordination environments between DFT models and experimentally fitted structures. Average Ru-W bond lengths obtained from DFT-optimized Ru1, Ru2, Ru3, Ru4, Ru5, and Ru6 ensembles on γ-WC and δ-WC supports. The blue shaded region represents the experimentally fitted Ru-W bond length range for Ru ALN/WC_x samples (aRu*N*/WC_x), whereas the green shaded region corresponds to Ru-W alloy-like samples (aRu5_p/WO_x, aRu5_p/WC_x, Ru_p/WC_x_Ar, and Ru/WC_x_Ar).

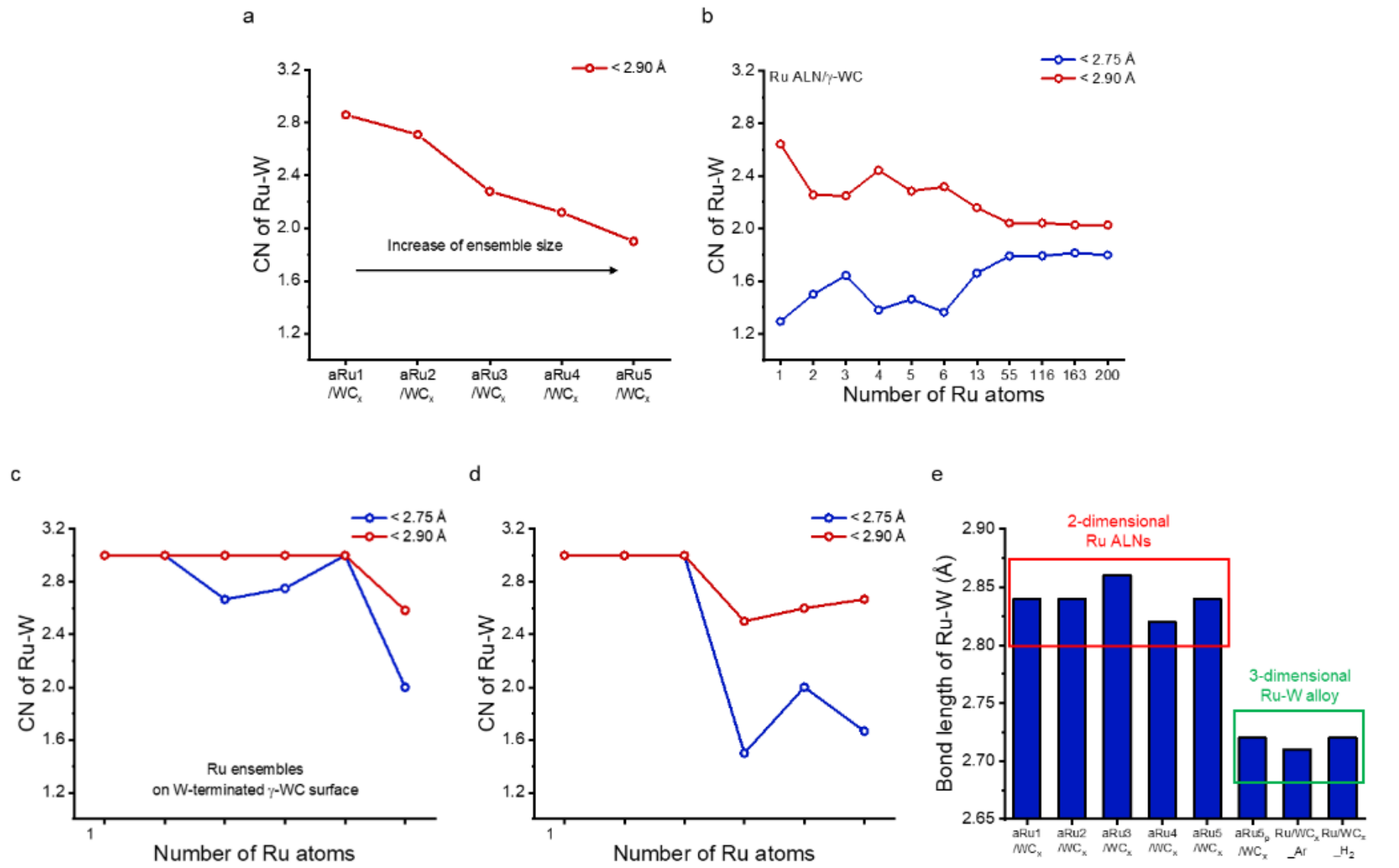

Supplementary Fig. 80 | Comparison between experimental and theoretical Ru-W coordination environments supporting the Ru ALN structure. a, Experimental Ru-W CNs obtained from EXAFS fitting for aRu*N*/WC_x (*N* = 1-5). **b-d,** DFT-calculated Ru-W CNs for (**b**) Ru ALN/WC_x models, (**c**) Ru ensembles on W-terminated γ-WC, and (d) Ru ensembles on W-terminated δ-WC, determined using cutoff distances of <2.75 Å (blue) and <2.90 Å (red). **e,** Comparison of experimentally fitted average Ru-W bond lengths. Samples assigned to Ru ALN/WC_x structures (red box) exhibited longer Ru-W bond lengths (~2.84 Å), whereas Ru-W alloy-like samples (green box) showed shorter Ru-W bond lengths (~2.70 Å). (CN: coordination number)

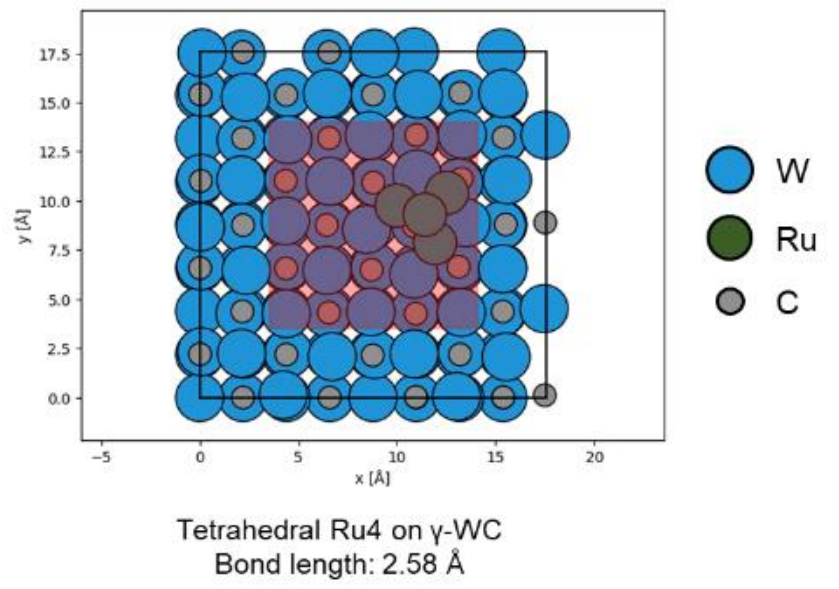

Supplementary Fig. 81 | DFT-optimized tetrahedral Ru4 ensemble on γ-WC. DFT-optimized structure of a three-dimensional tetrahedral Ru4 ensemble on γ-WC. The calculated average Ru-Ru bond length is 2.58 Å.

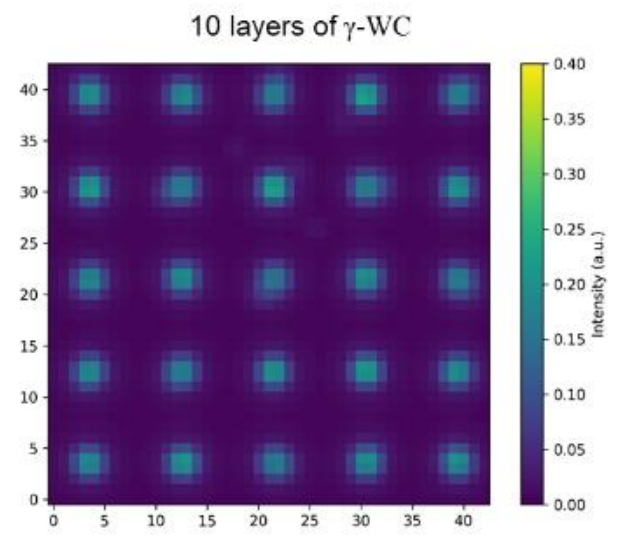

Supplementary Fig. 82 | Simulated HAADF-STEM images of a tetrahedral Ru4 ensemble on γ-WC. Simulated HAADF-STEM image of three-dimensional tetrahedral Ru4 ensemble on a 10-layer γ-WC slab.

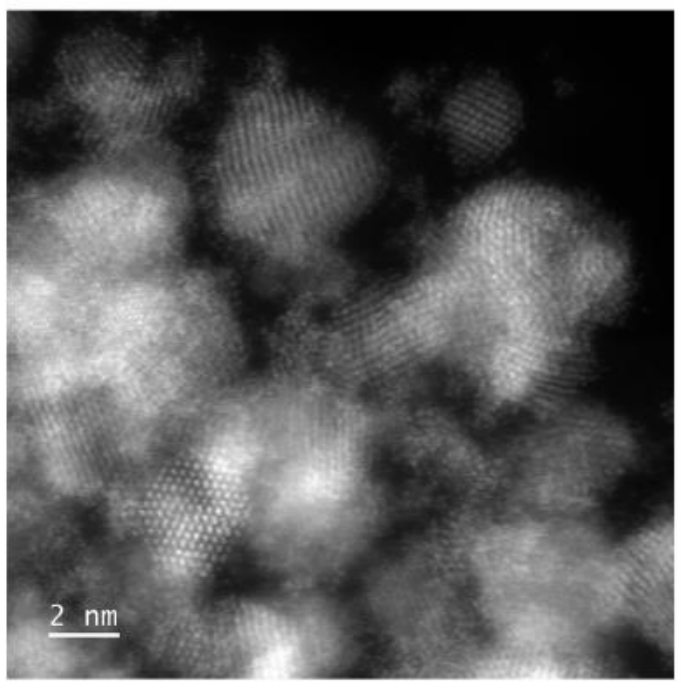

Supplementary Fig. 83 | High-resolution HAADF-STEM analysis of aRu5/WC_x. Representative high-resolution HAADF-STEM image of aRu5/WC_x.

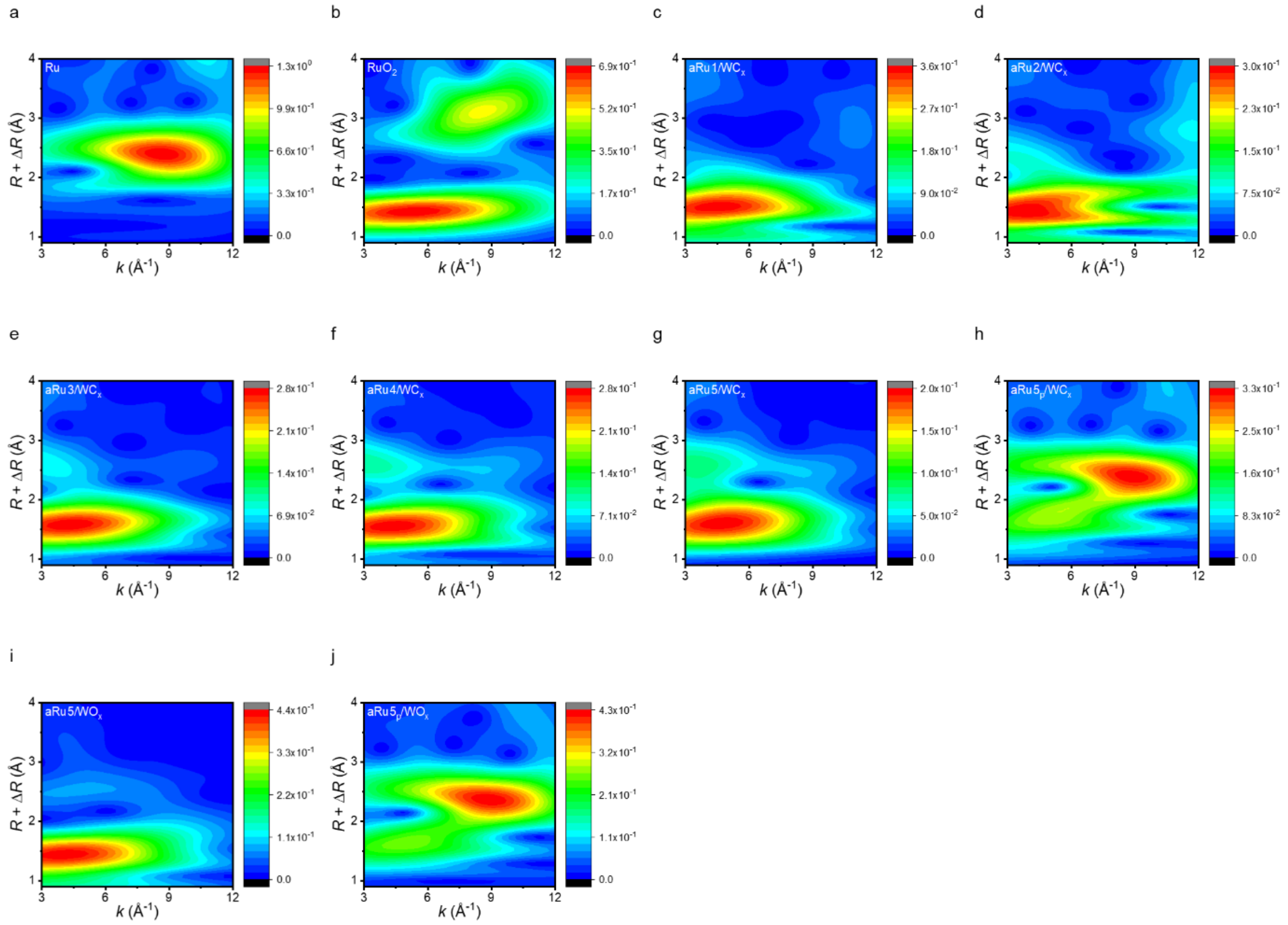

Supplementary Fig. 84 | WT-EXAFS. a-j, WT-EXAFS contour plots for (**a**) Ru foil, (**b**) RuO_2, (**c**) aRu1/WC_x, (**d**) aRu2/WC_x, (**e**) aRu3/WC_x, (**f**) aRu4/WC_x, (**g**) aRu5/WC_x, (**h**) $aRu5_p/WC_x$, (**i**) aRu5/WO_x, and (**j**) $aRu5_p/WO_x$. (*k*: the photoelectron wavenumber, $R + \Delta R$: the radial coordinate used in the wavelet-transform maps)

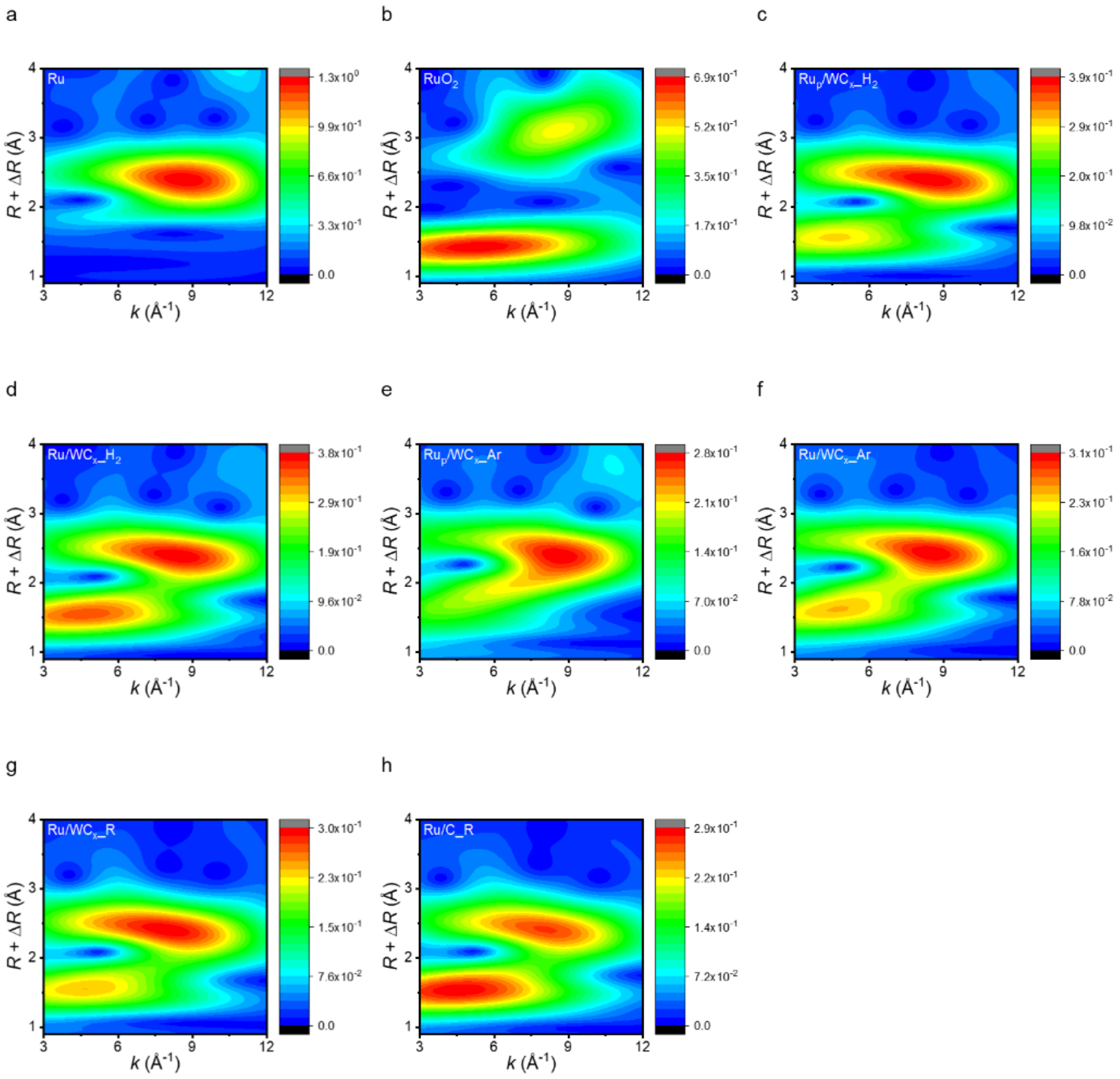

Supplementary Fig. 85 | WT-EXAFS. a-h, WT-EXAFS contour plots for (**a**) Ru foil, (**b**) RuO_2, (**c**) Ru_p/WC_x_H_2, (**d**) Ru/WC_x_H_2, (**e**) Ru_p/WC_x_Ar, (**f**) Ru/WC_x_Ar, (**g**) Ru/WC_x_R, and (**h**) Ru/C_R. (*k*: the photoelectron wavenumber, $R + \Delta R$: the radial coordinate used in the wavelet-transform maps)

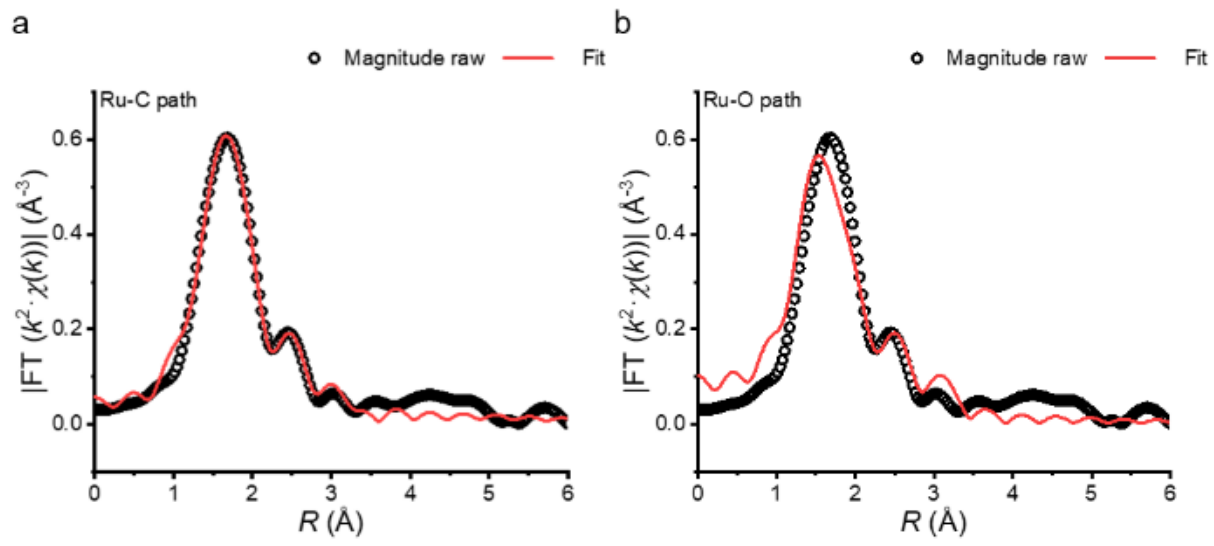

Supplementary Fig. 86 | Comparison of EXAFS Fitting results using Ru-C and Ru-O first shell scattering paths for aRu3/WC$_x$. a,b, Experimental and fitted Ru K-edge FT-EXAFS spectra of aRu3/WC$_x$ obtained using (**a**) a Ru-C first shell model and (**b**) Ru-O first shell model. (*R*: the radial distance in Fourier-transformed EXAFS *R* space)

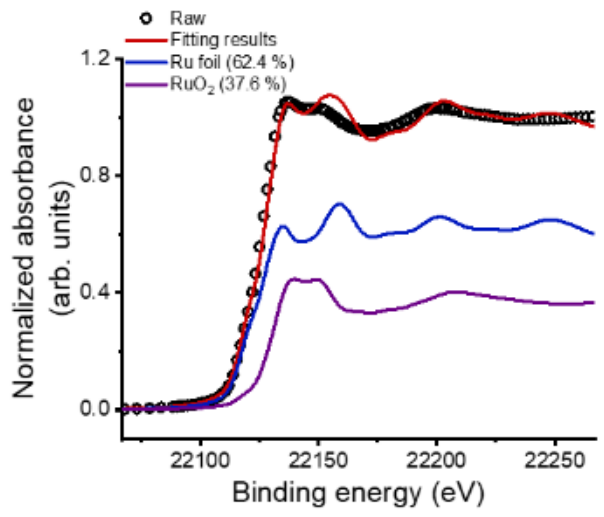

Supplementary Fig. 87 | Ru K-edge XANES linear combination fitting (LCF) of aRu3/WC$_x$. Experimental spectrum (black circles) and corresponding LCF result (red line) using Ru metal foil and RuO_2 standards.

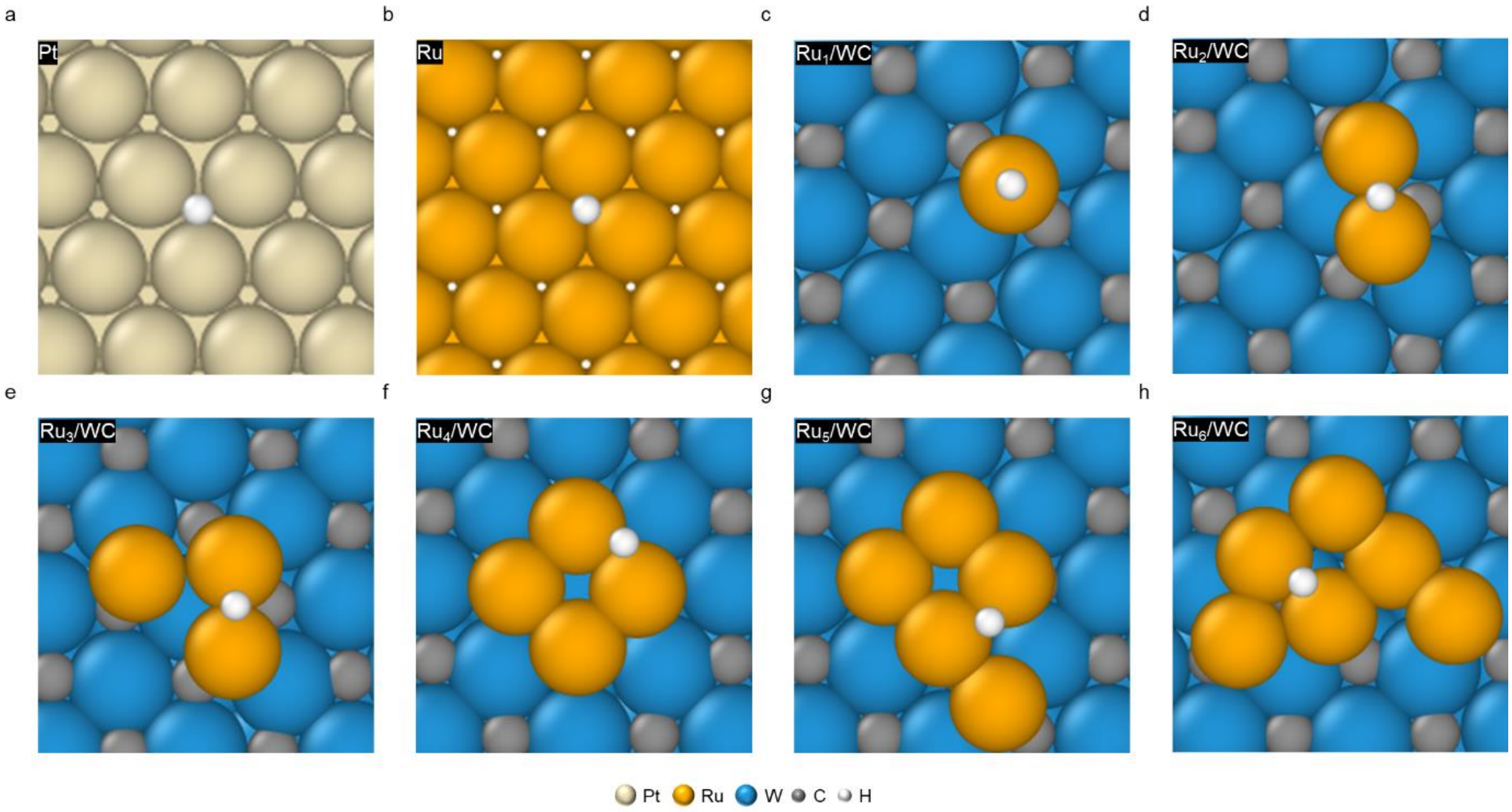

Supplementary Fig. 88 | The dependence of *H adsorption configurations on geometric structures of noble metal. a-h, DFT models used to calculate the ΔG_H for (**a**) Pt (111), (**b**) Ru (0001), (**c**) single-atom Ru on γ-WC (Ru_1/WC), (**d**) Ru dimer on γ-WC (Ru_2/WC), (**e**) Ru trimer on γ-WC (Ru_3/WC), (**f**) Ru tetramer on γ-WC (Ru_4/WC), (**g**) Ru pentamer on γ-WC (Ru_5/WC), and (**h**) Ru hexamer on γ-WC (Ru_6/WC).

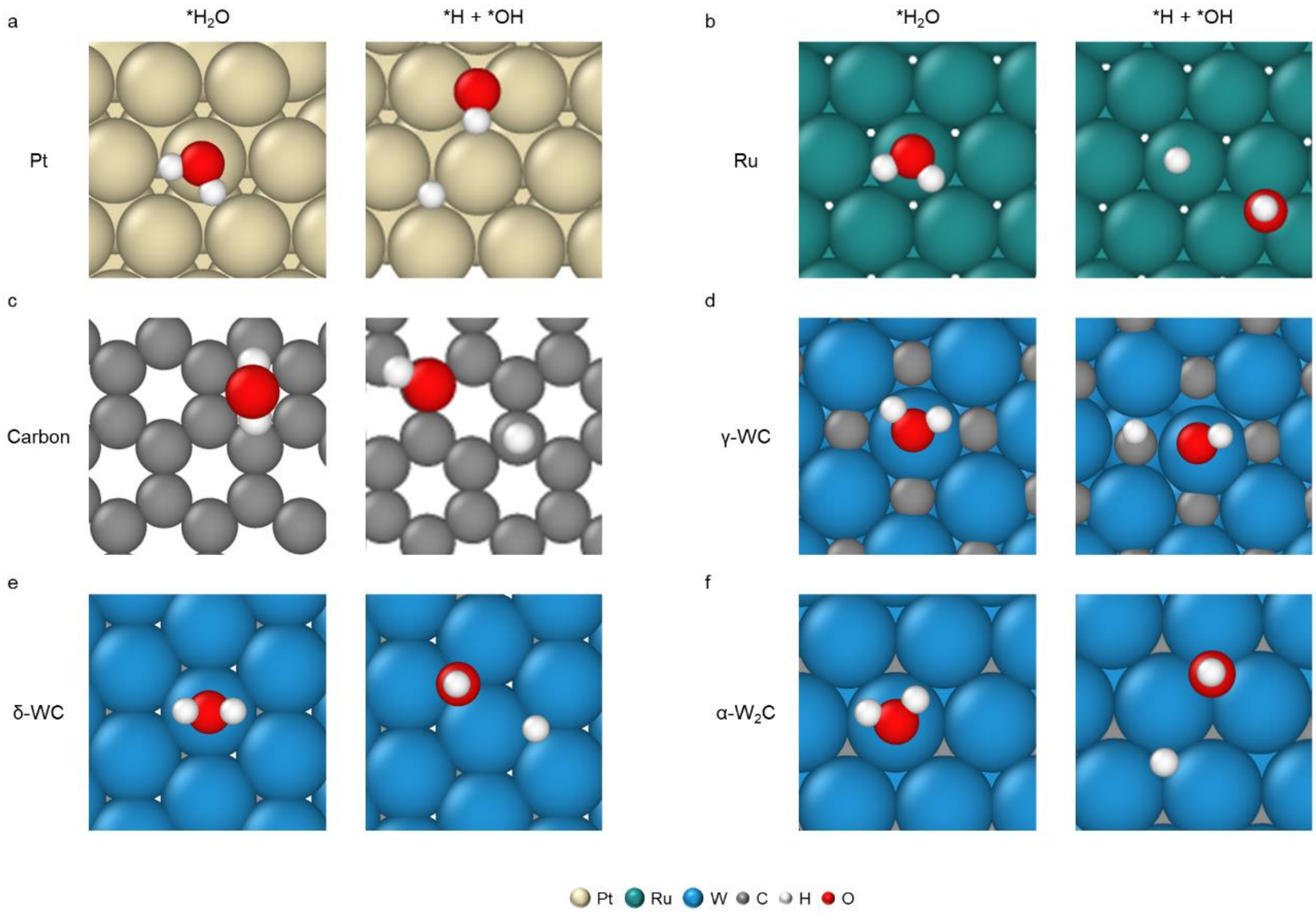

Supplementary Fig. 89 | The Adsorption configuration of oxygenated species depending on support types. a-f, DFT models for calculating the Gibbs free energy of adsorption for *H_2O and (*H + *OH) on (**a**) Pt (111), (**b**) Ru (0001), (**c**) graphene, (**d**) γ-WC, (**e**) δ-WC, and (**f**) α-W_2C.

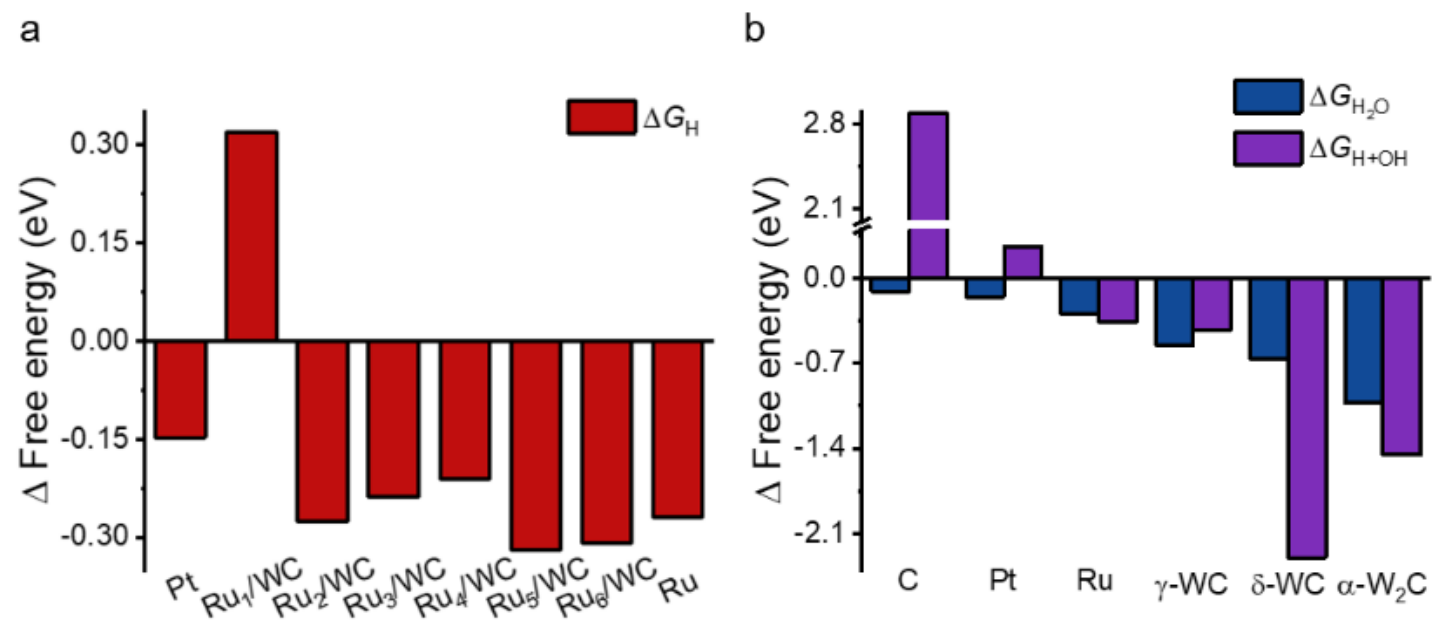

Supplementary Fig. 90 | Adsorbate binding energies. a,b, The Gibbs free energy for (**a**) *H, (**b**) *H_2O, and (*H + *OH) adsorption on catalysts.

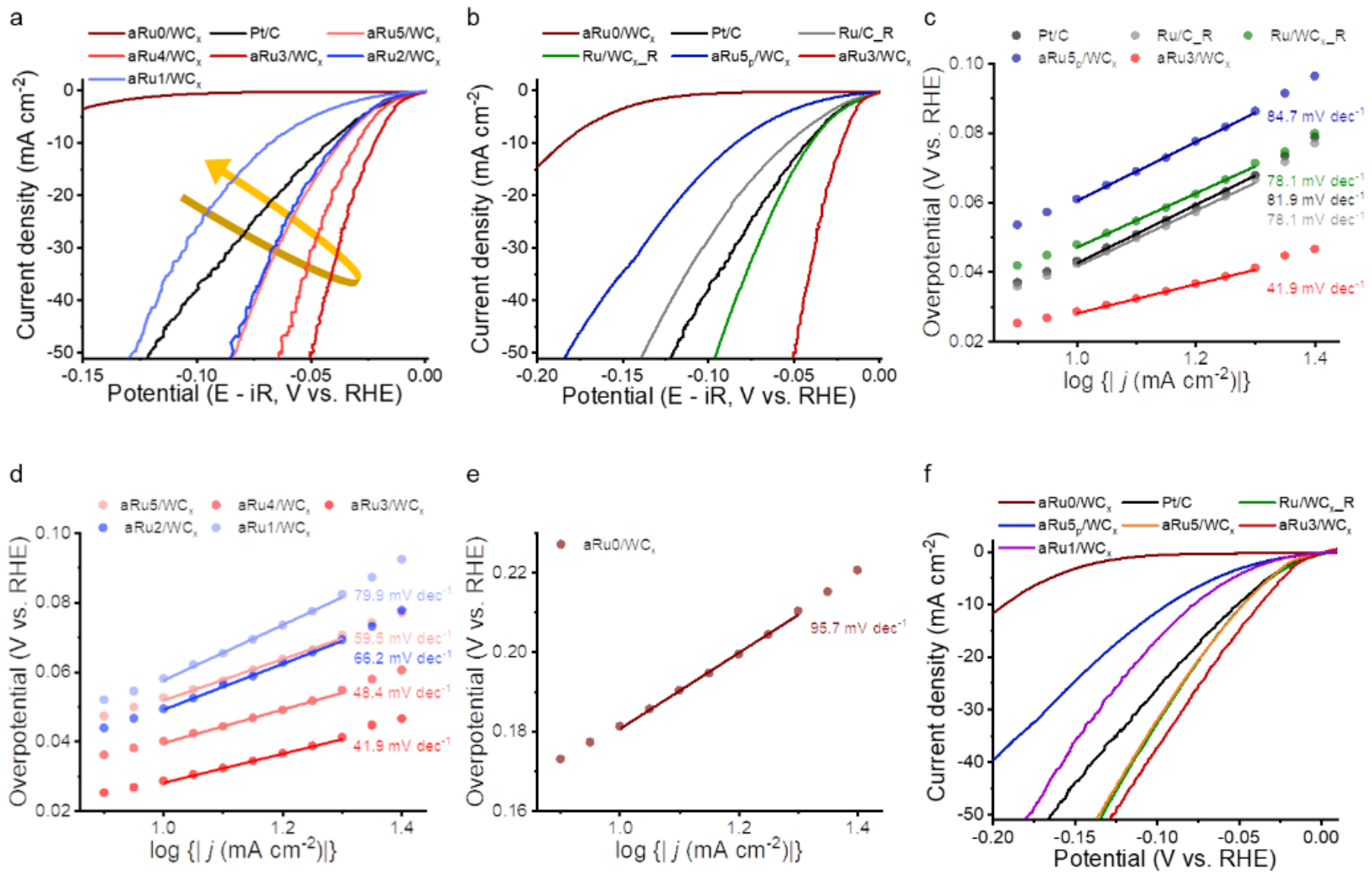

Supplementary Fig. 91 | Kinetic activity of alkaline HER. a,b, Linear sweep voltammetry (LSV) curves in H_2-saturated 1 M KOH solution showing HER performance of (**a**) samples obtained by thermodynamic stabilization (aRu*N*/WC_x), (**b**) aRu3/WC_x, comparison samples (aRu5$_p$/WC_x, Ru/WC_x_R, Ru/C_R), and aRu0/WC_x. **c-e,** Corresponding Tafel plots for (**c**) comparison samples (aRu5$_p$/WC_x, Ru/WC_x_R, Ru/C_R), aRu3/WC_x, (**d**) samples prepared by thermodynamic stabilization (aRu*N*/WC_x), and (**e**) aRu0/WC_x. All potentials are referenced to the reversible hydrogen electrode (RHE). The RDE polarization curves were iR-corrected using the EIS-derived uncompensated solution resistance measured before each experiment. **f,** Non-iR-corrected HER polarization curves of aRu*N*/WC_x and control catalysts. The uncompensated solution resistance value was 5.5 ± 1.5 Ω. The corresponding iR-corrected polarization curves are shown in Fig. 4c.

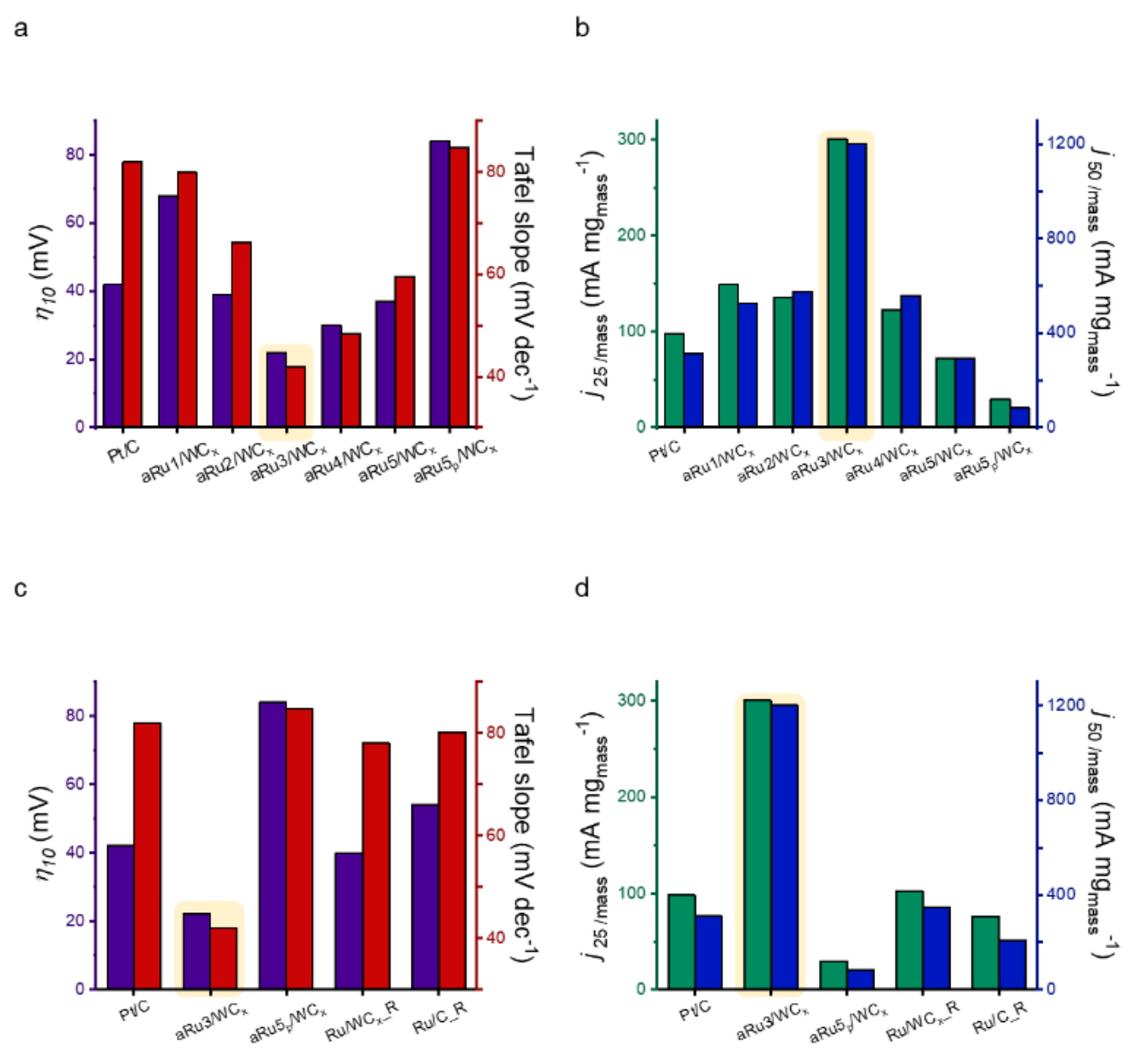

Supplementary Fig. 92 | Comparison of alkaline HER kinetic activity. a,c, Overpotential at -10 mA cm^{-2} (η_{10}, vs. RHE) and Tafel slopes for samples prepared by (**a**) thermodynamic stabilization (aRu*N*/WC$_x$), (**c**) aRu3/WC$_x$, and comparison samples (aRu5$_p$/WC$_x$, Ru/WC$_x$_R, Ru/C_R). **b,d,** current density per unit mass of noble metal (mass activity) at overpotentials of 25 mV ($j_{25\,/mass}$) and 50 mV ($j_{50\,/mass}$) for samples prepared by (**b**) thermodynamic stabilization (aRu*N*/WC$_x$), (**d**) aRu3/WC$_x$, and comparison samples (aRu5$_p$/WC$_x$, Ru/WC$_x$_R, Ru/C_R).

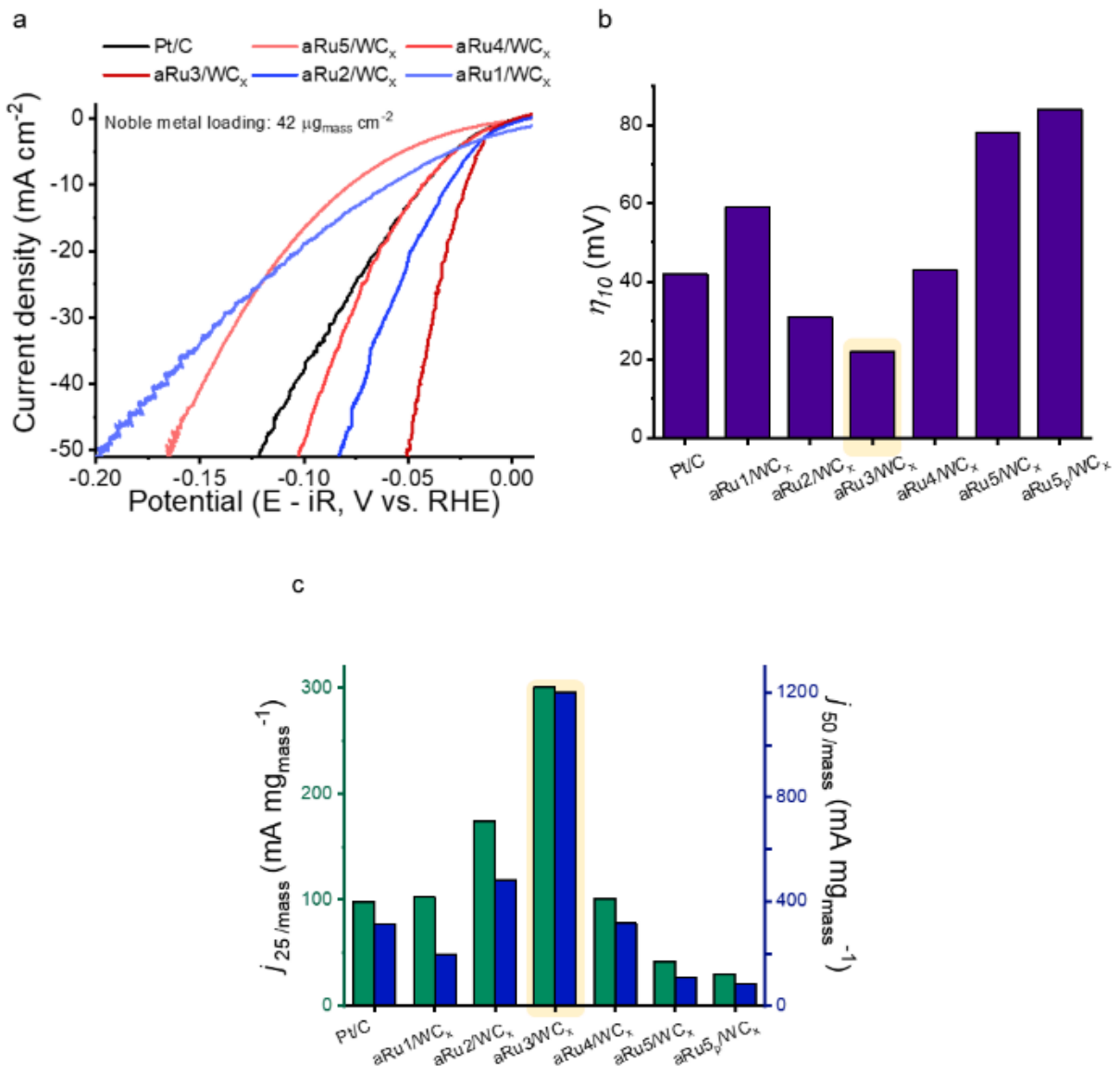

Supplementary Fig. 93 | Kinetic activity of aRu*N*/WC$_x$ (*N* = 1-5) for alkaline HER under identical noble metal loading (42 μg$_{mass}$ cm^{-2}). a, LSV curves in H_2-saturated 1 M KOH solution. **b,** Corresponding overpotentials (η_{10}) at −10 mA cm^{-2}, **c,** mass activity ($j_{25\,/mass}$ (green), and $j_{50\,/mass}$ (blue)). The RDE polarization curves were iR-corrected using the EIS-derived uncompensated solution resistance measured before each experiment.

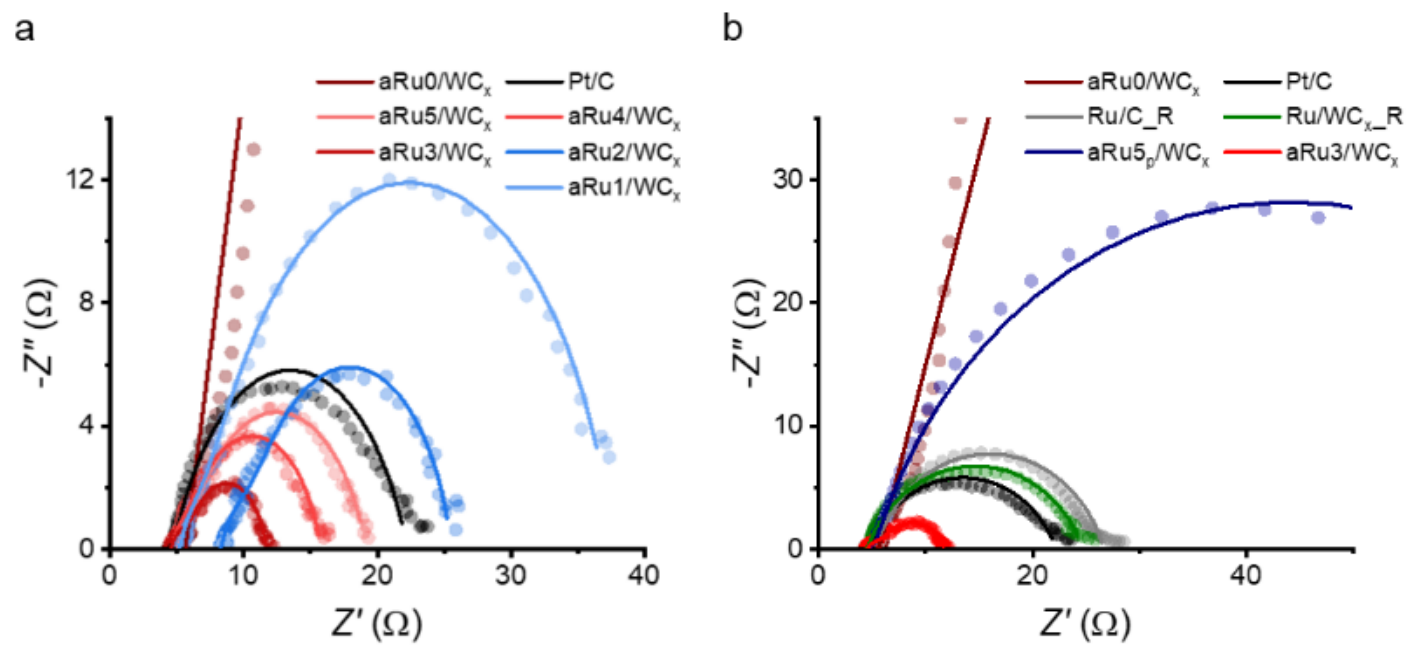

Supplementary Fig. 94 | Electrochemical impedance spectroscopy (EIS) for HER. a,b, EIS Nyquist plots at -40 mV in H_2-saturated 1 M KOH solution for (**a**) samples obtained via thermodynamic stabilization (aRu*N*/WC_x), (**b**) aRu3/WC_x, and comparison samples (aRu5_p/WC_x, Ru/WC_x_R, Ru/C_R).

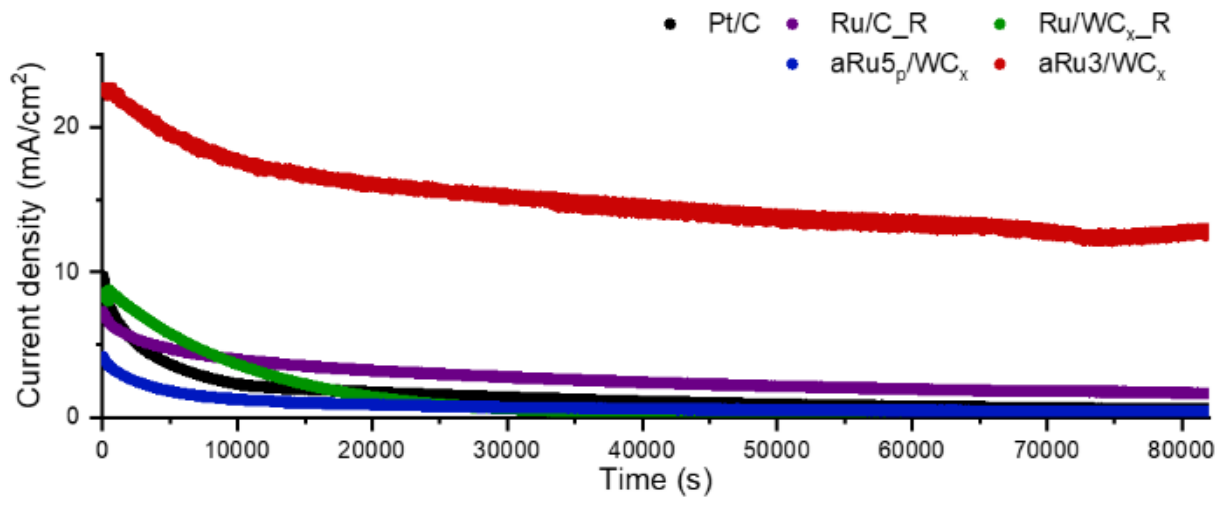

Supplementary Fig. 95 | Electrochemical durability of aRu3/WC$_x$ for alkaline HER. *j* of aRu3/WC$_x$, and comparison samples (aRu5$_p$/WC$_x$, Ru/WC$_x$_R, Ru/C_R) in chronopotentiometry at an overpotential of 50 mV over 80,000 s, in H_2-saturated 1 M KOH solution.

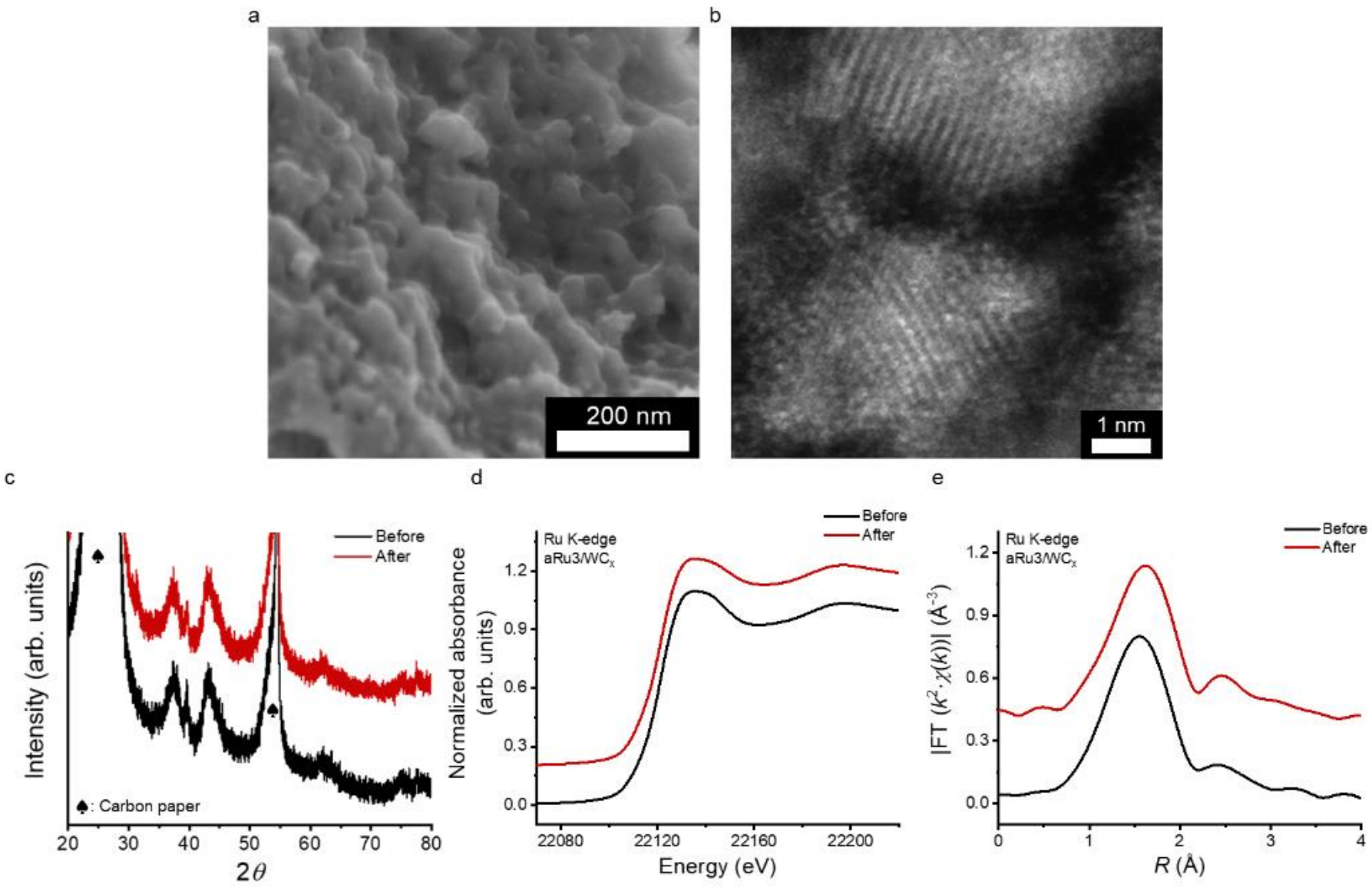

Supplementary Fig. 96 | Material characterization of aRu3/WC_x after the long-term durability test. a, SEM image and **b,** high-resolution HAADF-STEM image of the used aRu3/WC_x catalyst. **c-e,** Comparison of aRu3/WC_x before and after durability test via (**c**) XRD patterns (*: carbon paper), (**d**) Ru K-edge XANES spectra, and (**e**) Ru K-edge FT-EXAFS spectra. (*R*: the radial distance in Fourier-transformed EXAFS *R* space)

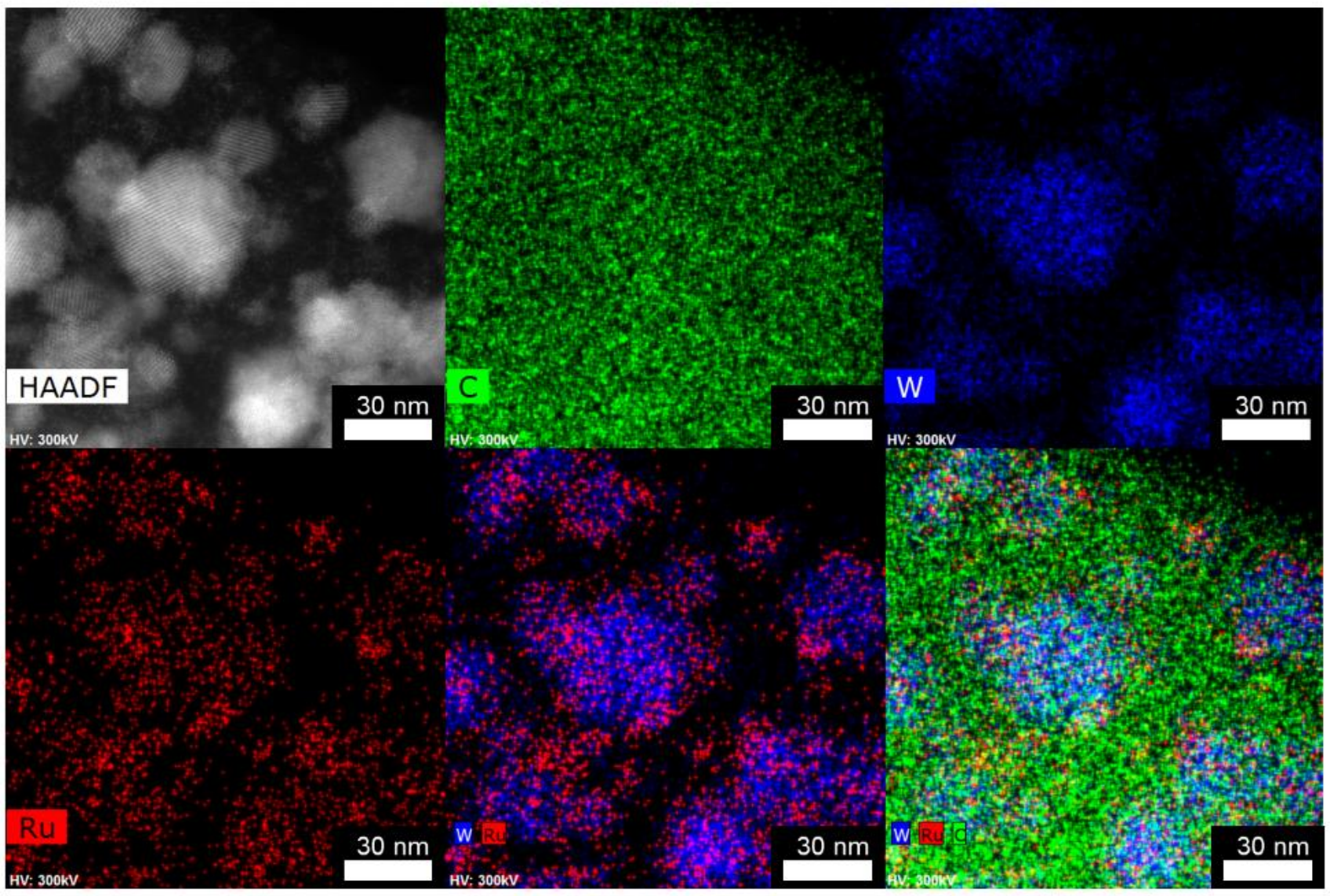

Supplementary Fig. 97 | STEM analysis of aRu3/WC_x after the long-term durability test. High-resolution HAADF-STEM image and the corresponding EDS elemental mappings for carbon (green), tungsten (blue), and ruthenium (red), along with their overlay images.

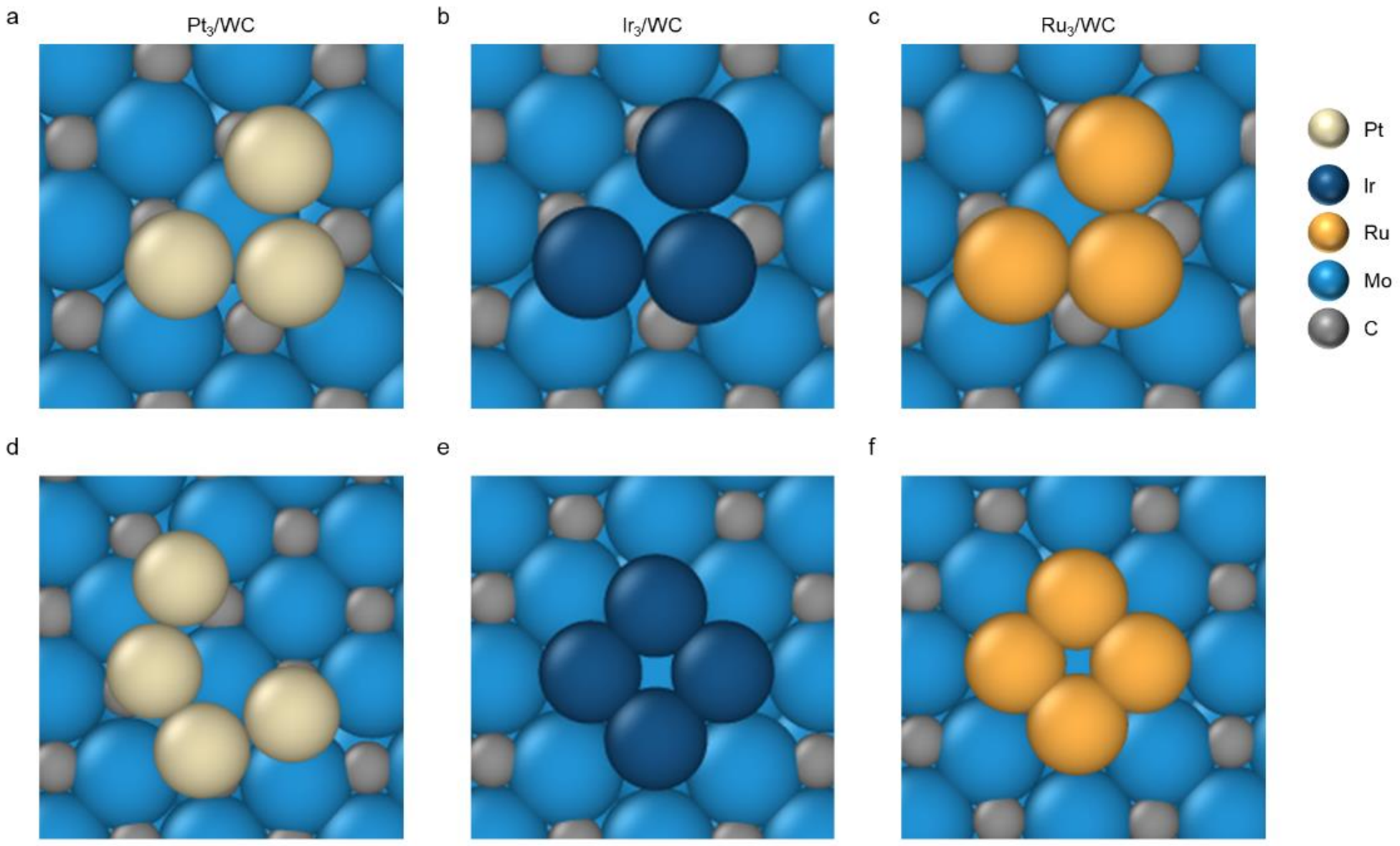

Supplementary Fig. 98 | DFT-optimized structures of noble metal ALNs on γ-WC support. a-f, Optimized atomic configurations of noble metal ALNs supported on γ-WC, including (a) Pt_3/WC, (**b**) Ir_3/WC, (**c**) Ru_3/WC, (**d**) Pt_4/WC, (**e**) Ir_4/WC, and (**f**) Ru_4/WC, which were used to calculate the cohesive energy (E_{coh}) and binding energy (E_{bind}) of the noble metal ALNs on γ-WC.

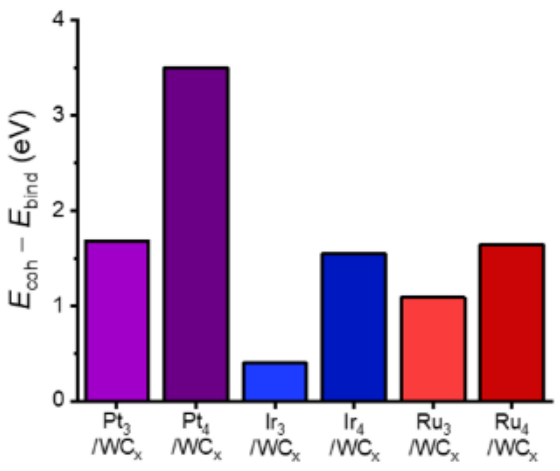

Supplementary Fig. 99 | Relative stabilization energies of noble metal ALNs on γ-WC support. Comparison between the cohesive energy (E_{coh}) of noble metals and the binding energy (E_{bind}) of noble metal ALNs on γ-WC support.

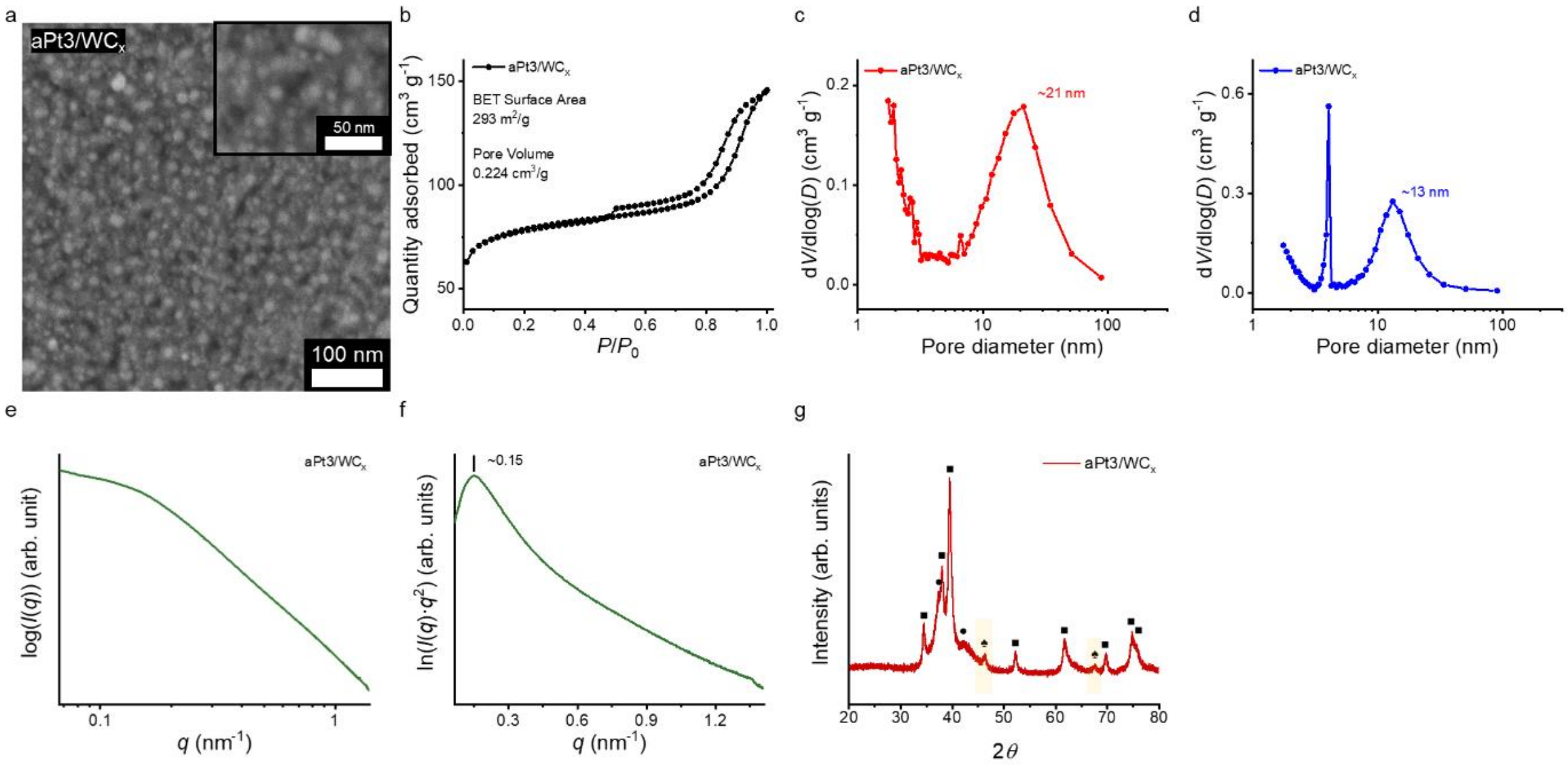

Supplementary Fig. 100 | Structural characterization of aPt3/WC_x. a, SEM image of aPt3/WC_x. Insets show the corresponding higher-magnification image. **b,** N_2 adsorption-desorption isotherms, **c,** pore size distribution, and **d**, window size distribution derived from N_2 physisorption analysis. **e,** SAXS scattering profile and **f,** corresponding Kratky plot (*q*: the magnitude of the scattering vector). **g,** XRD pattern of aPt3/WC_x. The pale-yellow shaded regions highlight the crystalline Pt peaks.

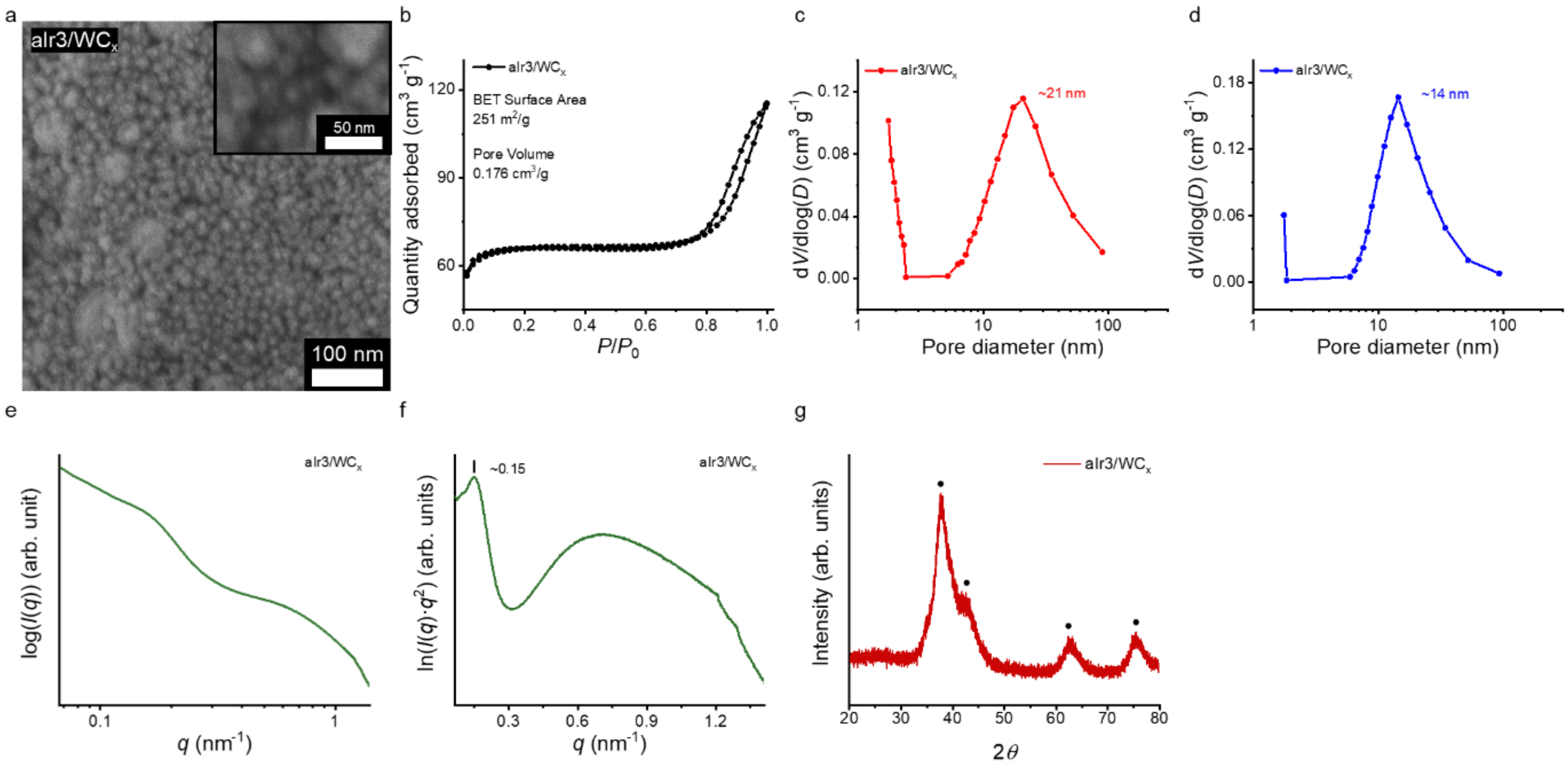

Supplementary Fig. 101 | Structural characterization of aIr3/WC$_x$. a, SEM image of aIr3/WC$_x$. Insets show the corresponding higher-magnification image. **b,** N_2 adsorption-desorption isotherms, **c,** pore size distribution, and **d**, window size distribution derived from N_2 physisorption analysis. **e,** SAXS scattering profile and **f,** corresponding Kratky plot (*q*: the magnitude of the scattering vector). **g,** XRD pattern of aIr3/WC$_x$.

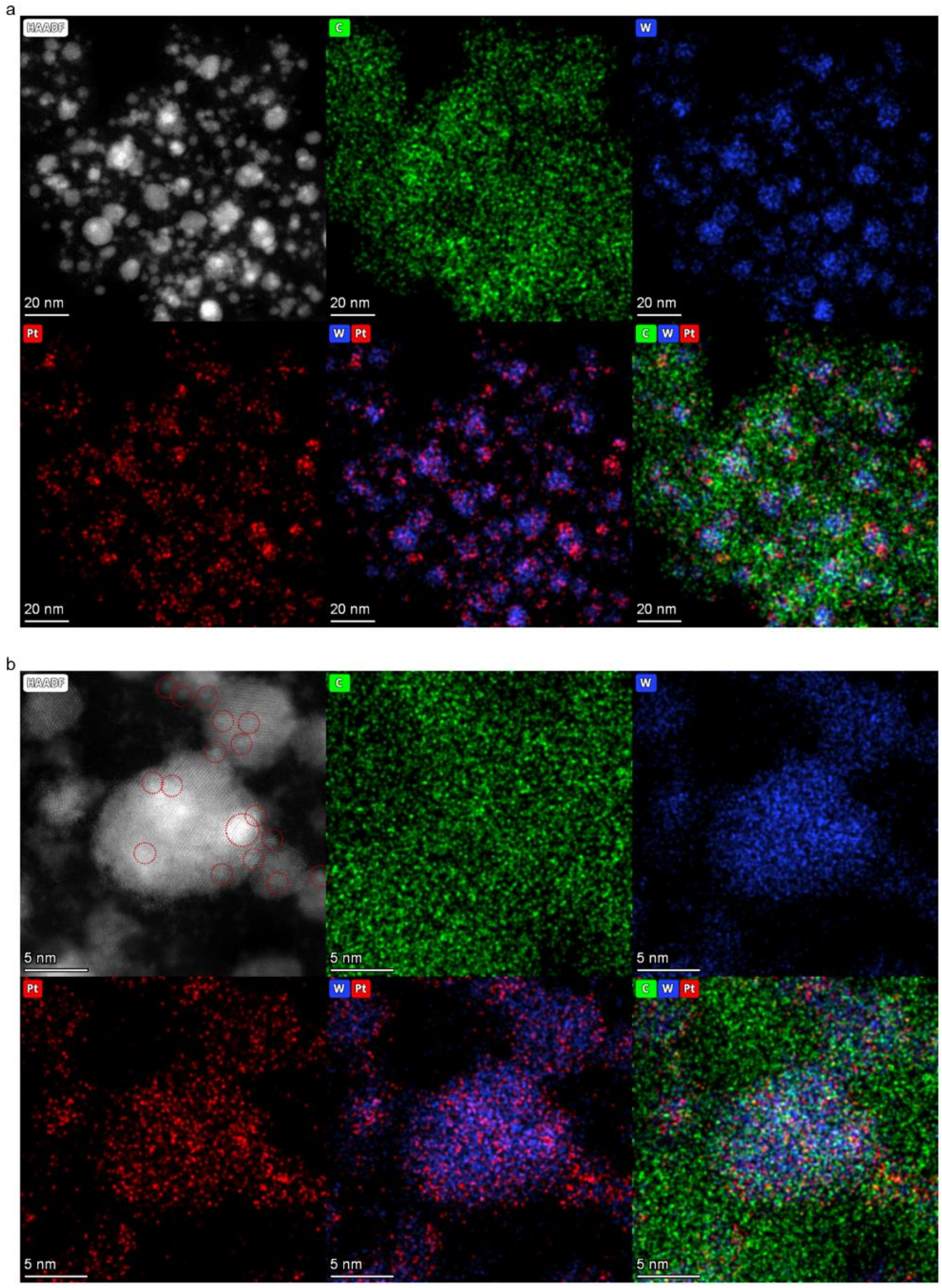

Supplementary Fig. 102 | STEM analysis of aPt3/WC$_x$. a, HAADF-STEM image and the corresponding EDS elemental mappings for C (green), W (blue), Pt (red), along with the overlaid composite map. **b,** Higher-magnification HAADF-STEM image and corresponding EDS mappings for the same elements, their composite overlay.

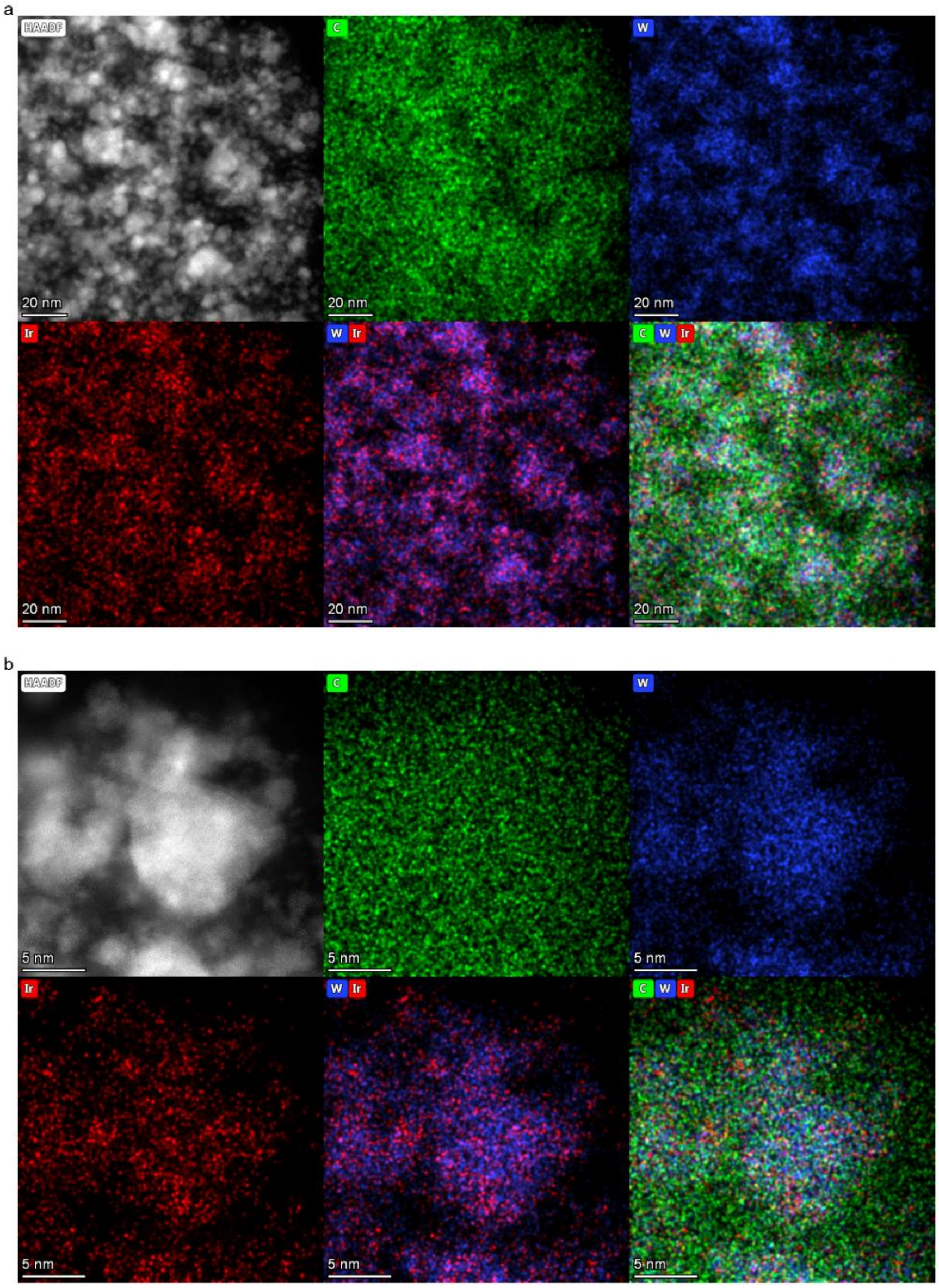

Supplementary Fig. 103 | STEM analysis of aIr3/WC$_x$. a, HAADF-STEM image and the corresponding EDS elemental mappings for C (green), W (blue), Ir (red), along with the overlaid composite map. **b,** Higher-magnification HAADF-STEM image and corresponding EDS mappings for the same elements, their composite overlay.

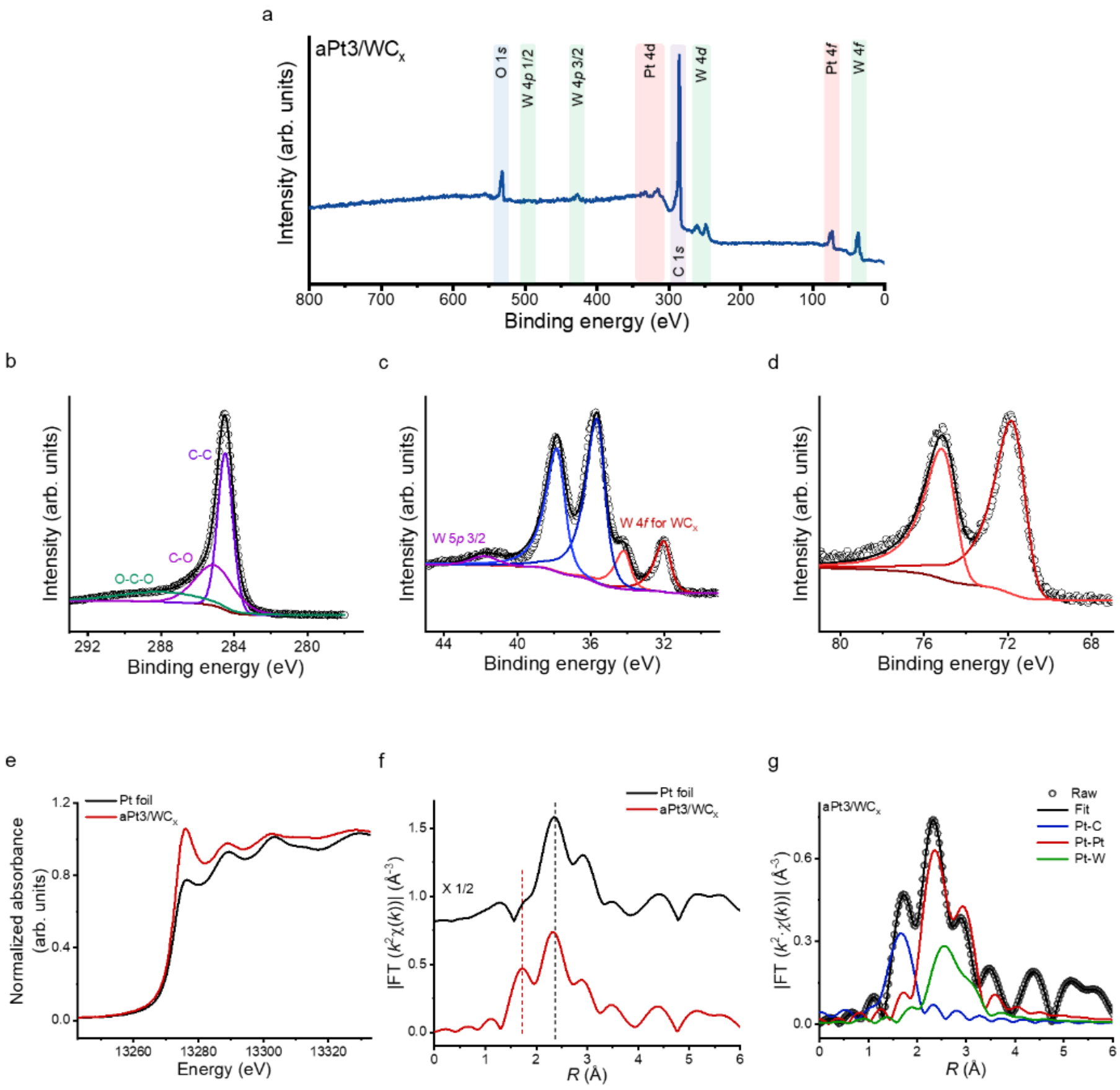

Supplementary Fig. 104 | Electronic and geometric structure analyses of aPt3/WC$_x$. a-d, XPS analyses of aPt3/WC$_x$. (**a**) Survey XPS spectrum. (**b**) C 1*s* spectrum (violet: C-C, purple: C-O, green: O-C-O), (**c**) W 4*f* spectrum (red: WC$_x$, blue: surface WO$_x$ attributed to naturally formed oxides, purple: W 5*p* 3/2), and (**d**) Pt 4*f* spectrum. **e-g,** XAS analyses of aPt3/WC$_x$ at the Pt L$_2$-edge. (**e**) XANES spectra, (**f**) k^2-weighted FT-EXAFS spectra, and (**g**) corresponding FT-EXAFS fitting results. (*R*: the radial distance in Fourier-transformed EXAFS *R* space)

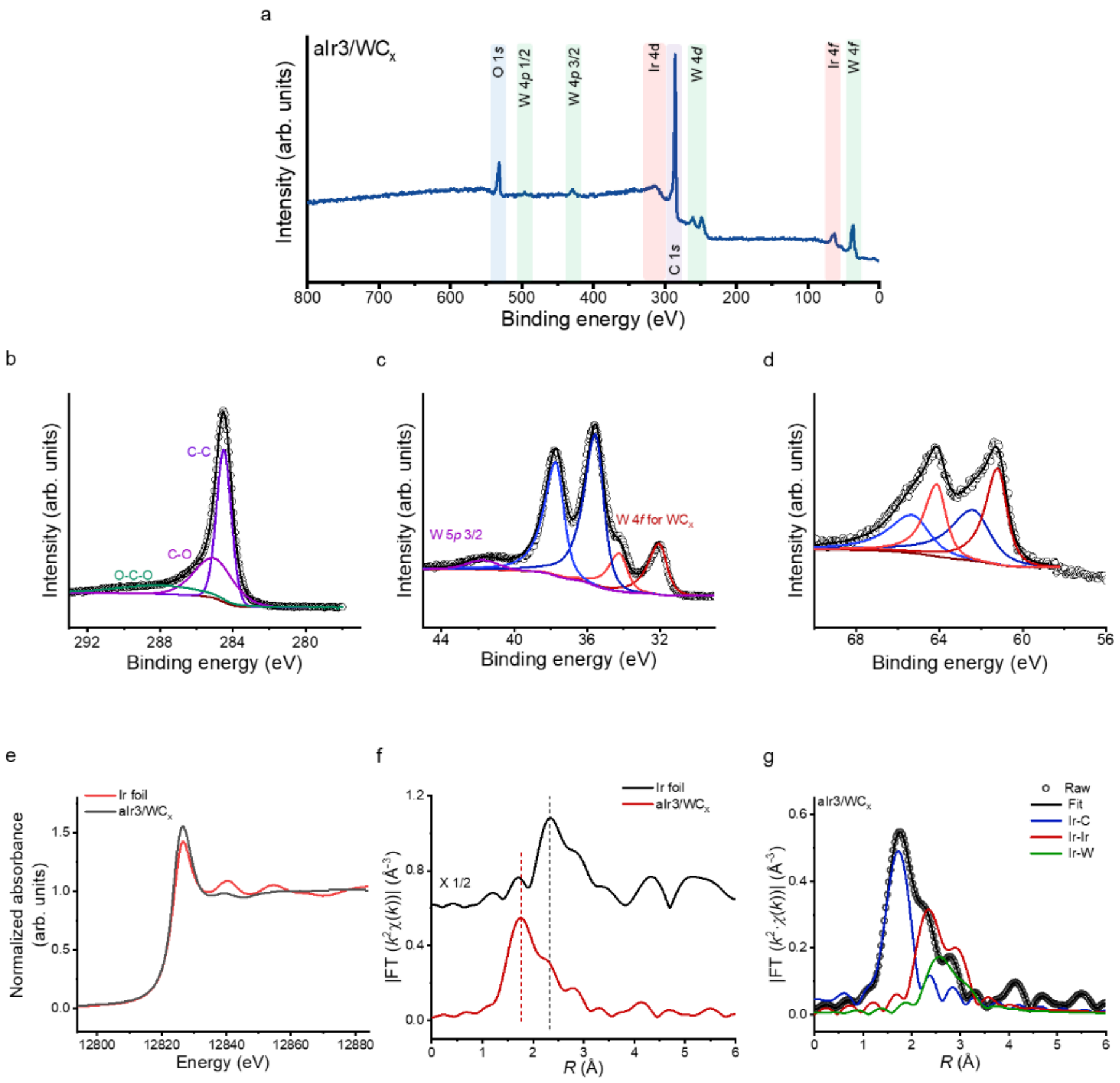

Supplementary Fig. 105 | Electronic and geometric structure analyses of aIr3/WC$_x$. a-d, XPS analyses of aIr3/WC$_x$. (**a**) Survey XPS spectrum. (**b**) C 1*s* spectrum (violet: C-C, purple: C-O, green: O-C-O), (**c**) W 4*f* spectrum (red: WC$_x$, blue: surface WO$_x$ attributed to naturally formed oxides, purple: W 5*p* 3/2), and (**d**) Ir 4*f* spectrum. **e-g,** XAS analyses of aIr3/WC$_x$ at the Ir L$_2$-edge. (**e**) XANES spectra, (**f**) k^2-weighted FT-EXAFS spectra, and (**g**) corresponding FT-EXAFS fitting results. (*R*: the radial distance in Fourier-transformed EXAFS *R* space)

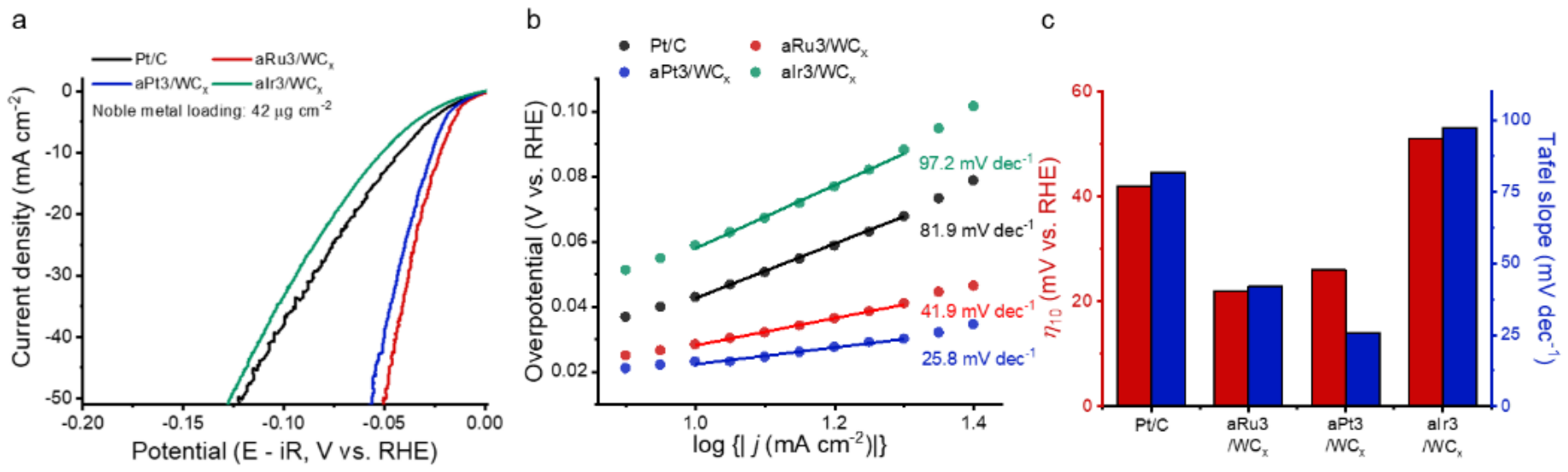

Supplementary Fig. 106 | Alkaline HER activity for aPt3/WC$_x$ and aIr3/WC$_x$. a, LSV curves, **b,** corresponding Tafel plot, and **c,** corresponding η_{10} and Tafel slope. All potentials are referenced to the reversible hydrogen electrode (RHE). The RDE polarization curves were iR-corrected using the EIS-derived uncompensated solution resistance measured before each experiment.

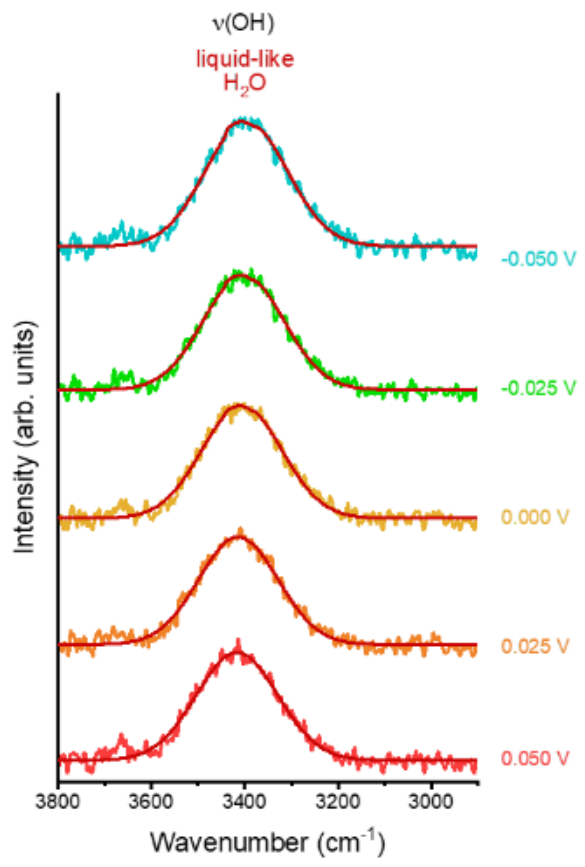

Supplementary Fig. 107 | In situ ATR-SEIRAS results coupled with LSV. In situ attenuated total reflection surface-enhanced infrared absorption spectroscopy (ATR-SEIRAS) spectra during LSV from 0.050 to -0.050 V (vs. RHE) for commercial Pt/C used for alkaline HER in H_2-saturated 1 M KOH solution. All potentials are referenced to the reversible hydrogen electrode (RHE).

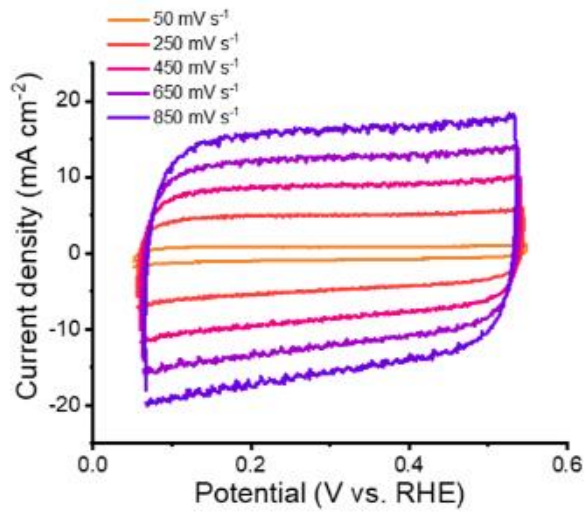

Supplementary Fig. 108 | Electrochemical *H desorption behavior of aRu3/WC_x. Cyclic voltammetry (CV) profiles of aRu3/WC_x catalyst with the scan rate from 50 to 850 mV s^{-1} in Ar-saturated 1.0 M KOH. All potentials are referenced to the reversible hydrogen electrode (RHE).

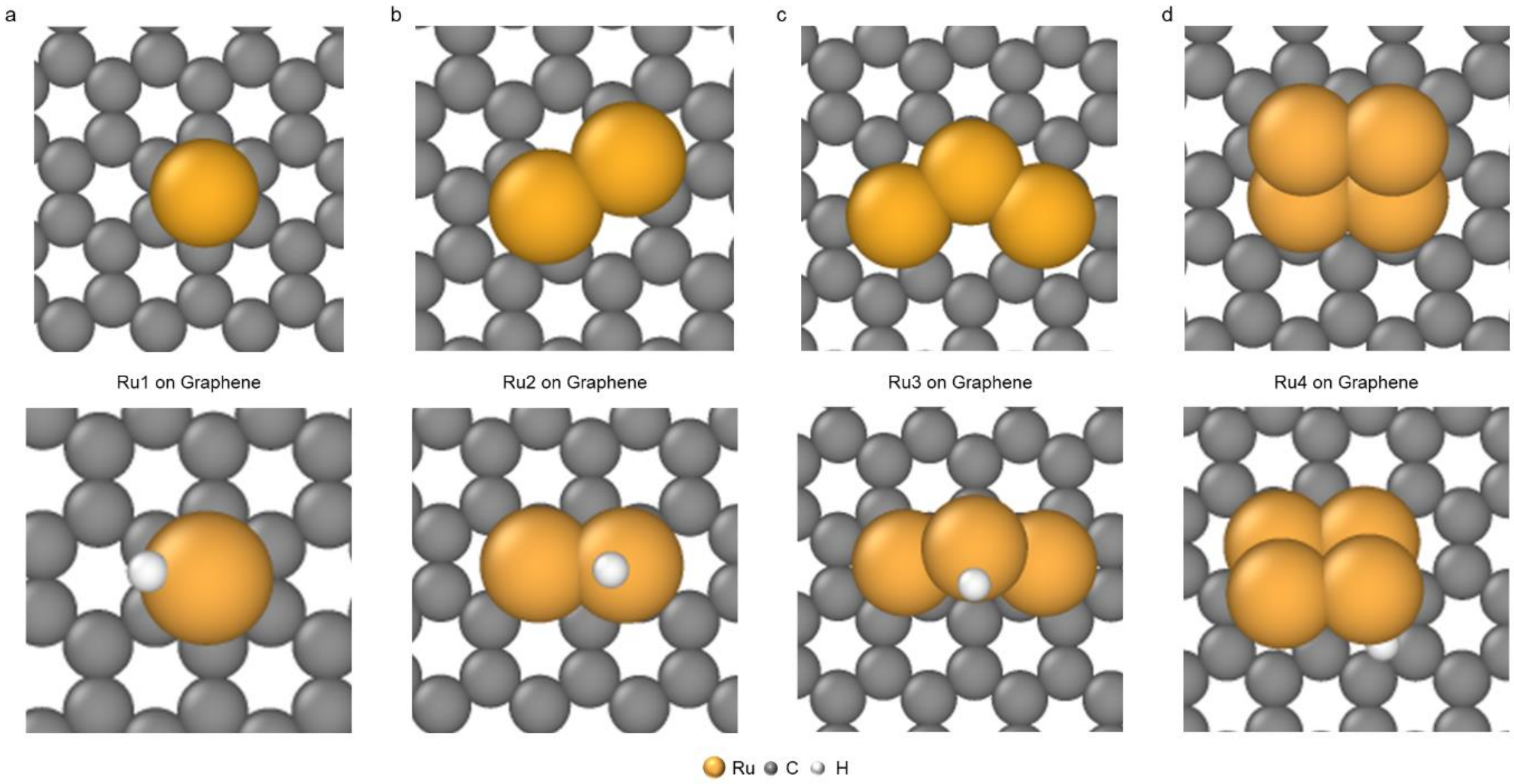

Supplementary Fig. 109 | DFT-optimized structures of Ru ensembles on graphene. a-d, Optimized atomic configurations of (**a**) Ru1, (**b**) Ru2, (**c**) Ru3, and (**d**) Ru4 ensembles supported on graphene together with the corresponding *H adsorbed configurations, which were used for the Bader charge analyses and ΔG_H calculations.

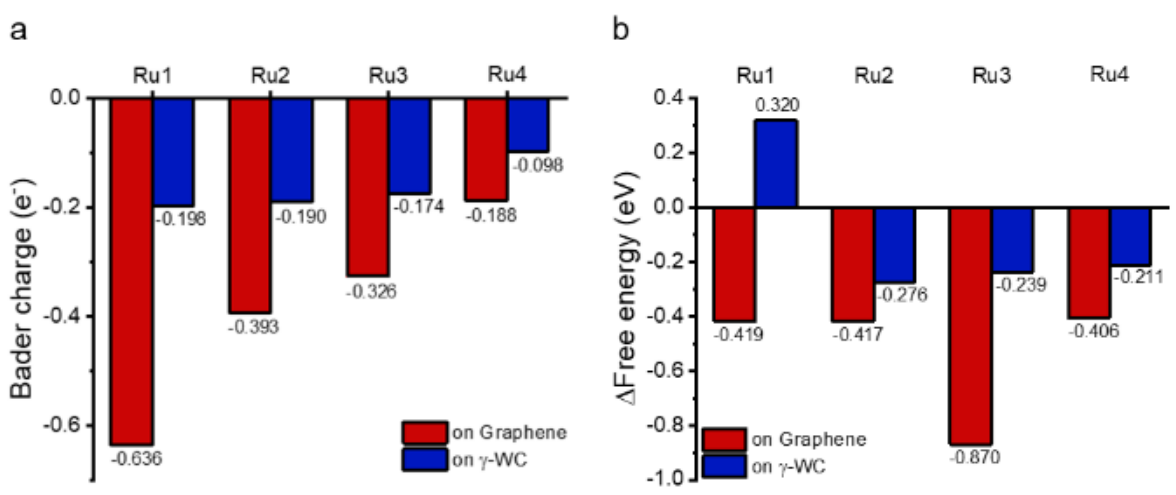

Supplementary Fig. 110 | Electronic structure and hydrogen adsorption properties of Ru ensembles on different supports. a, Bader charge analyses, and **b,** calculated ΔG_H for Ru ensembles supported on graphene and γ-WC.

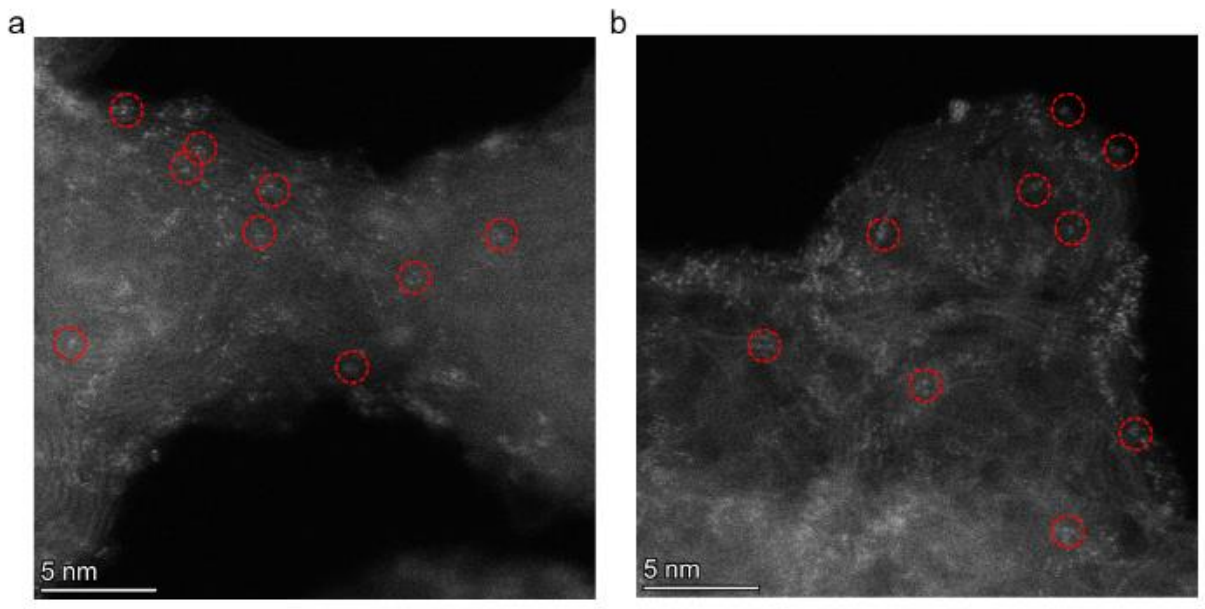

Supplementary Fig. 111 | Atomic-scale structure of aRu/NC. a-b, Representative HAADF-STEM images of aRu/NC (red dotted circles: Ru clusters composed of fewer than 10 Ru atoms)

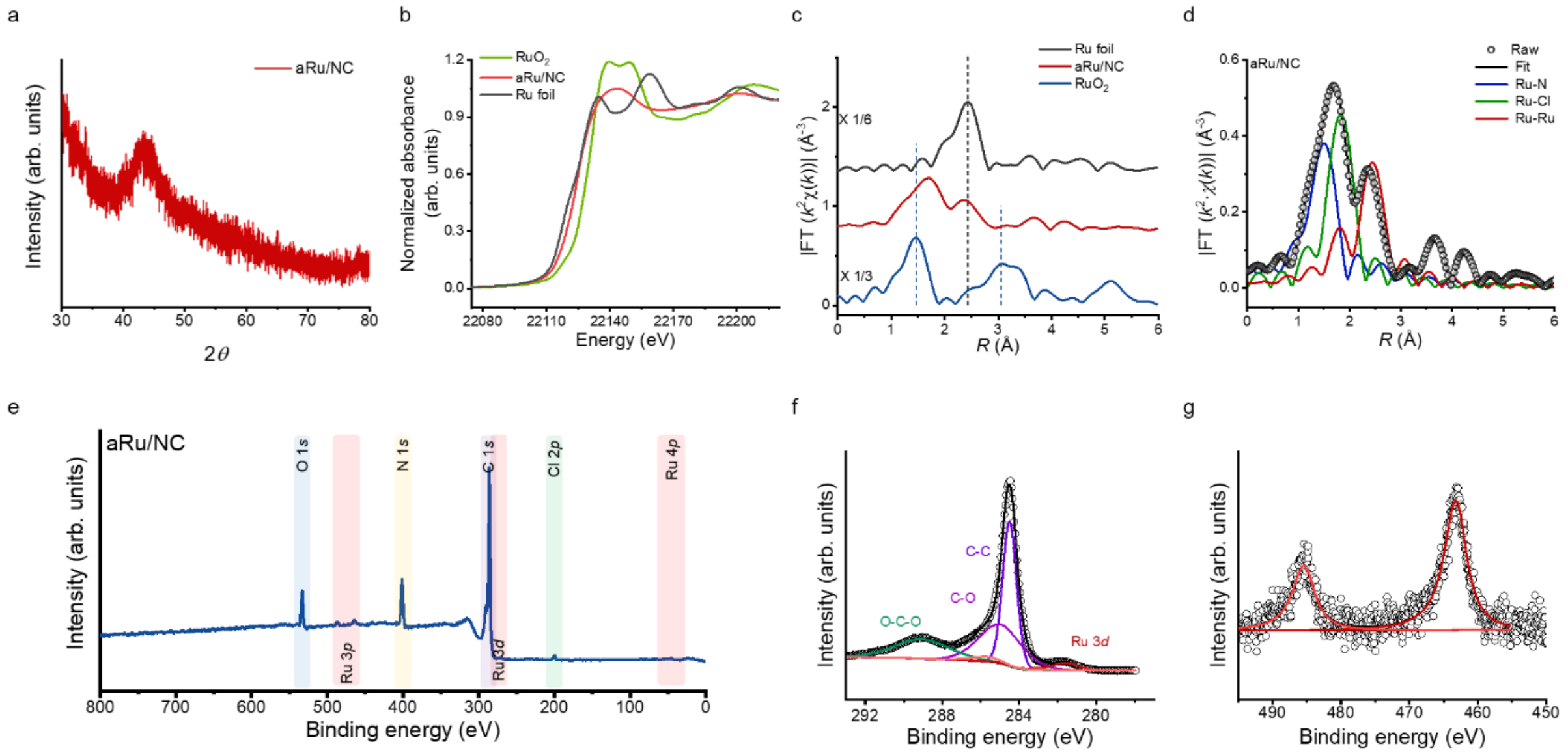

Supplementary Fig. 112 | Geometric and electronic structure of aRu/NC. a, XRD pattern of aRu/NC. **b-d,** XAS analyses of aRu/NC at the Ru K-edge, including (**b**) XANES spectra, (**c**) k^2-weighted FT-EXAFS spectra, and (**d**) corresponding FT-EXAFS fitting results (*R*: the radial distance in Fourier-transformed EXAFS *R* space). **e-g,** XPS analyses of aRu/NC, including (**e**) survey spectrum, (**f**) C 1*s* spectrum, and (**g**) Ru 3*p* spectrum.

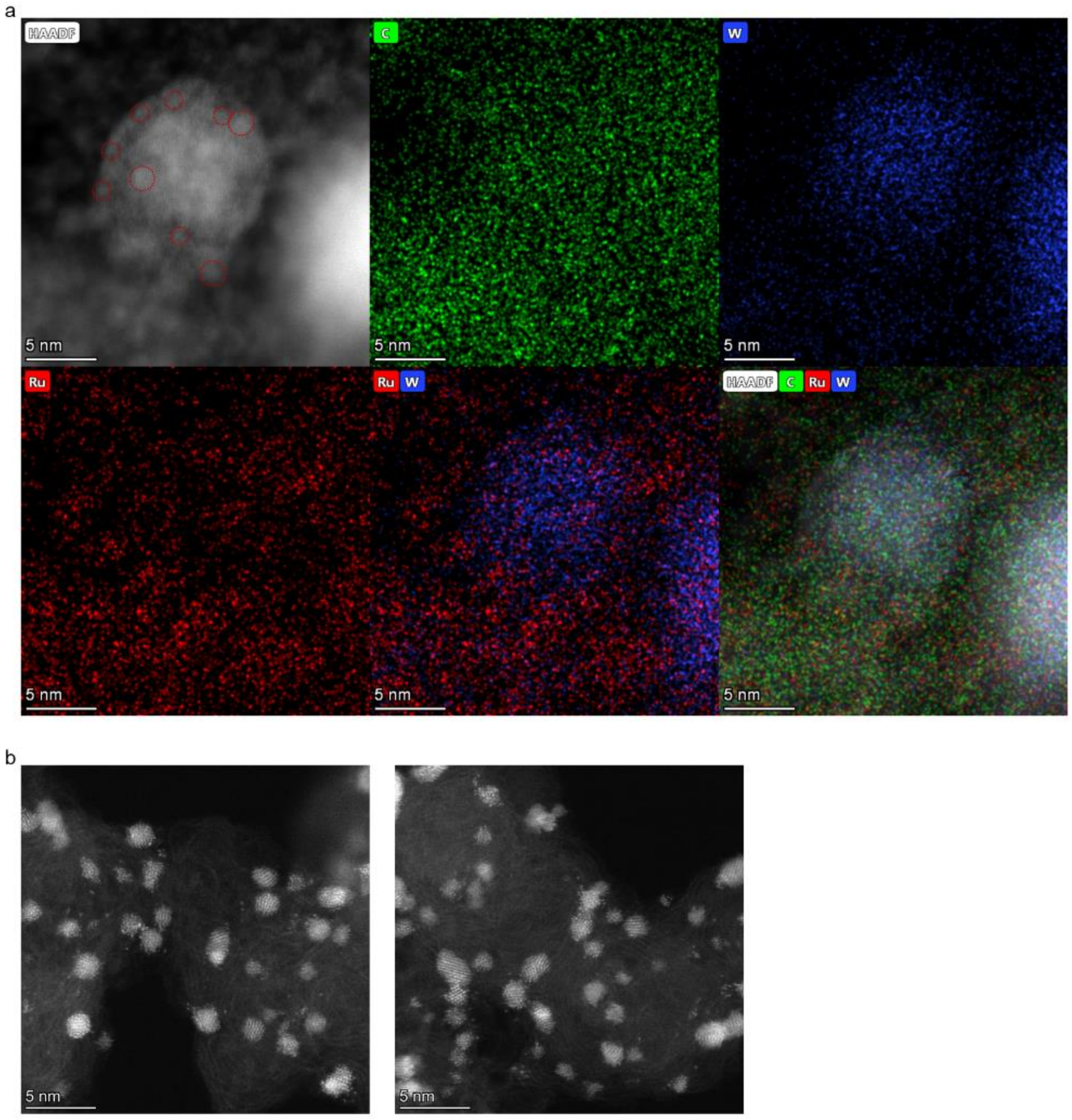

Supplementary Fig. 113 | STEM analysis of RuNP/WC$_x$ and RuNP/C. a, HAADF-STEM image and the corresponding EDS elemental mappings of RuNP/WC$_x$ for C (green), W (blue), Ru (red), along with the overlaid composite map (red dotted circle: Ru NPs). **b,** HAADF-STEM images of RuNP/C.

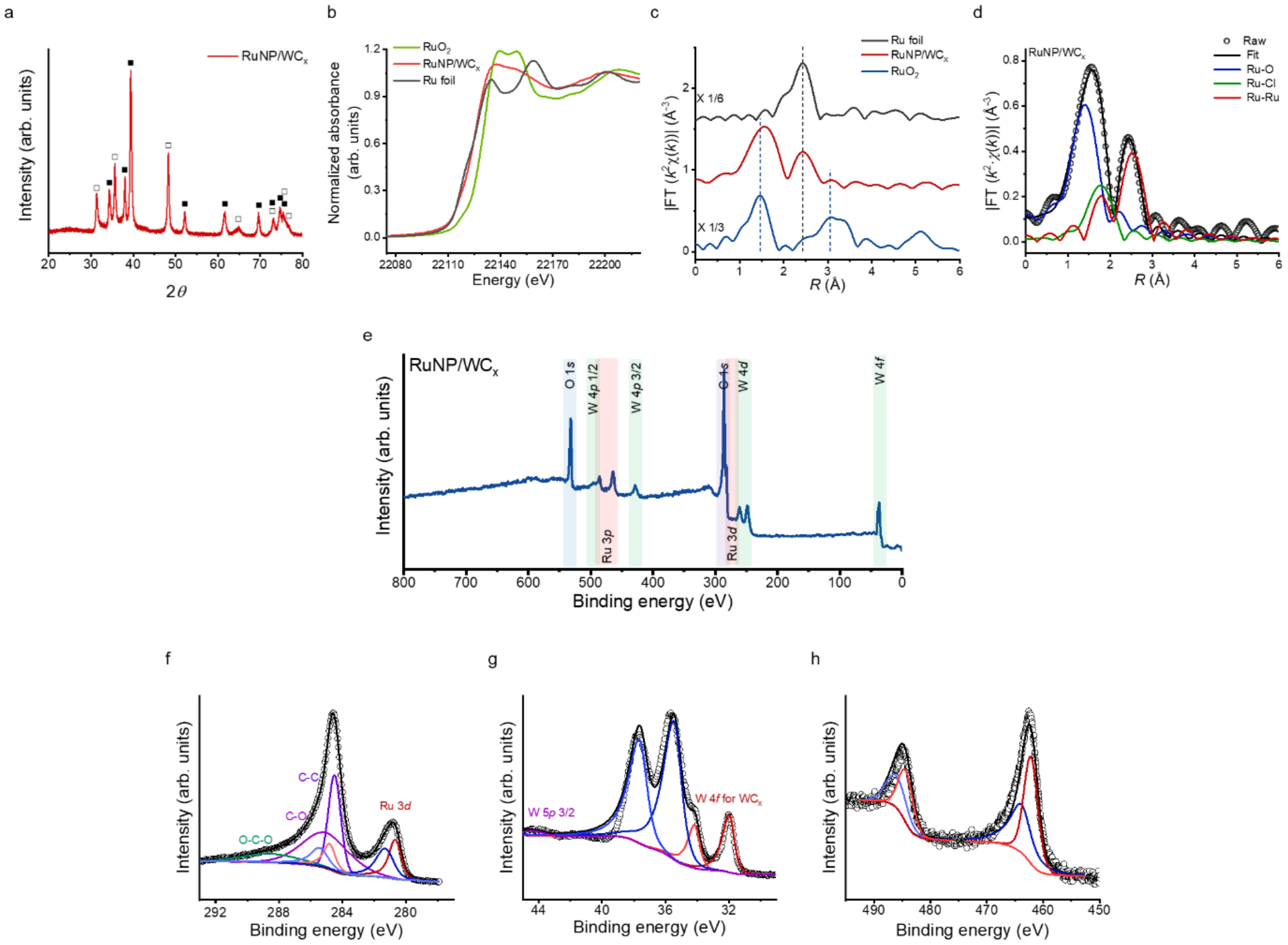

Supplementary Fig. 114 | Geometric and electronic structure of RuNP/WC$_x$. a, XRD pattern of RuNP/WC$_x$. **b-d,** XAS analyses of RuNP/WC$_x$ at the Ru K-edge, including (**b**) XANES spectra, (**c**) k^2-weighted FT-EXAFS spectra, and (**d**) corresponding FT-EXAFS fitting results (*R*: the radial distance in Fourier-transformed EXAFS *R* space). **e-h,** XPS analyses of RuNP/WC$_x$, including (**e**) survey spectrum, (**f**) C 1*s* spectrum, (**g**) W 4*f* spectrum, and (**h**) Ru 3*p* spectrum.

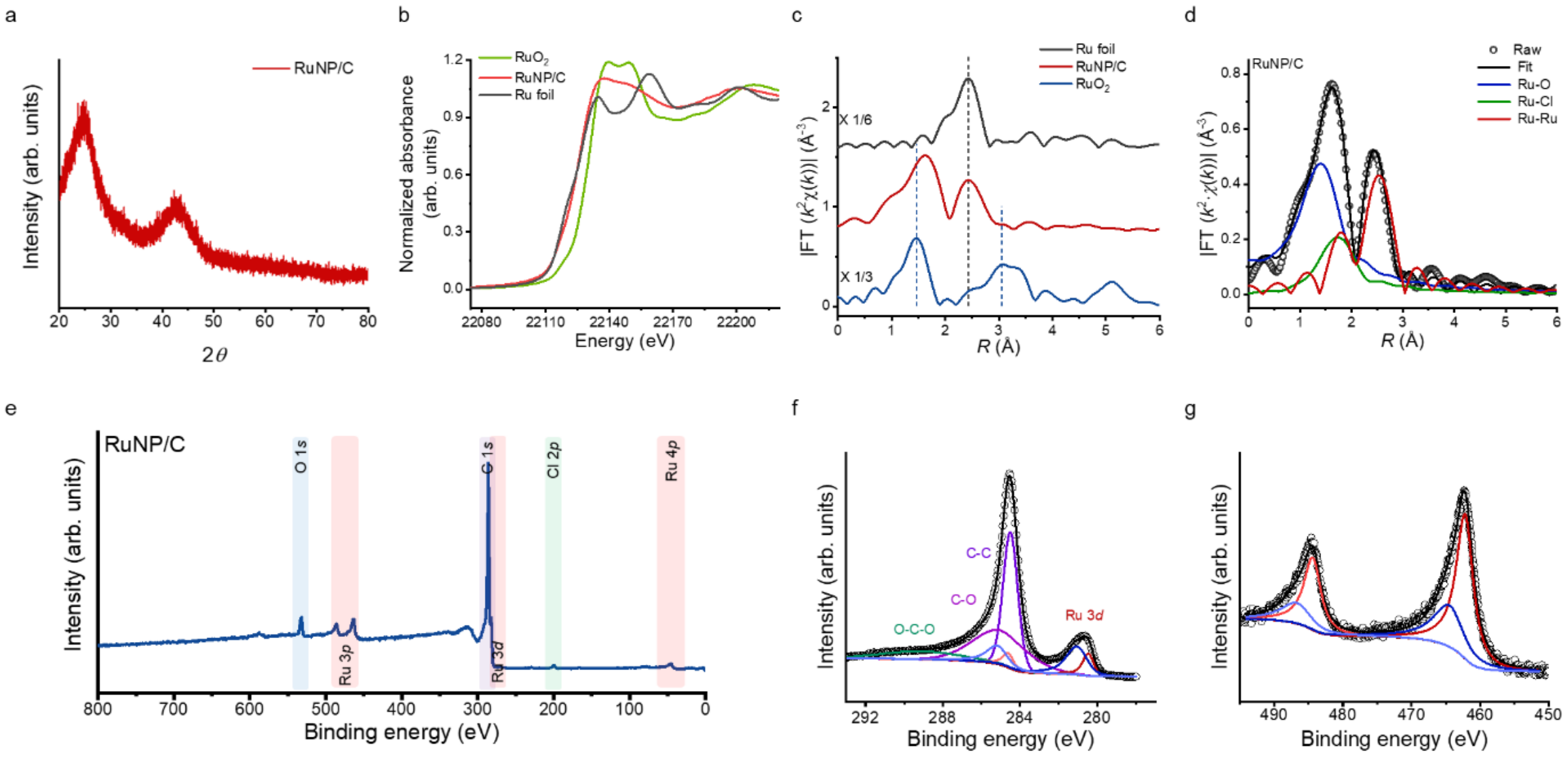

Supplementary Fig. 115 | Geometric and electronic structure of RuNP/C. a, XRD pattern of RuNP/C. **b-d,** XAS analyses of RuNP/C at the Ru K-edge, including (**b**) XANES spectra, (**c**) k^2-weighted FT-EXAFS spectra, and (**d**) corresponding FT-EXAFS fitting results (*R*: the radial distance in Fourier-transformed EXAFS *R* space). **e-g,** XPS analyses of RuNP/C, including (**e**) survey spectrum, (**f**) C 1*s* spectrum, and (**g**) Ru 3*p* spectrum.

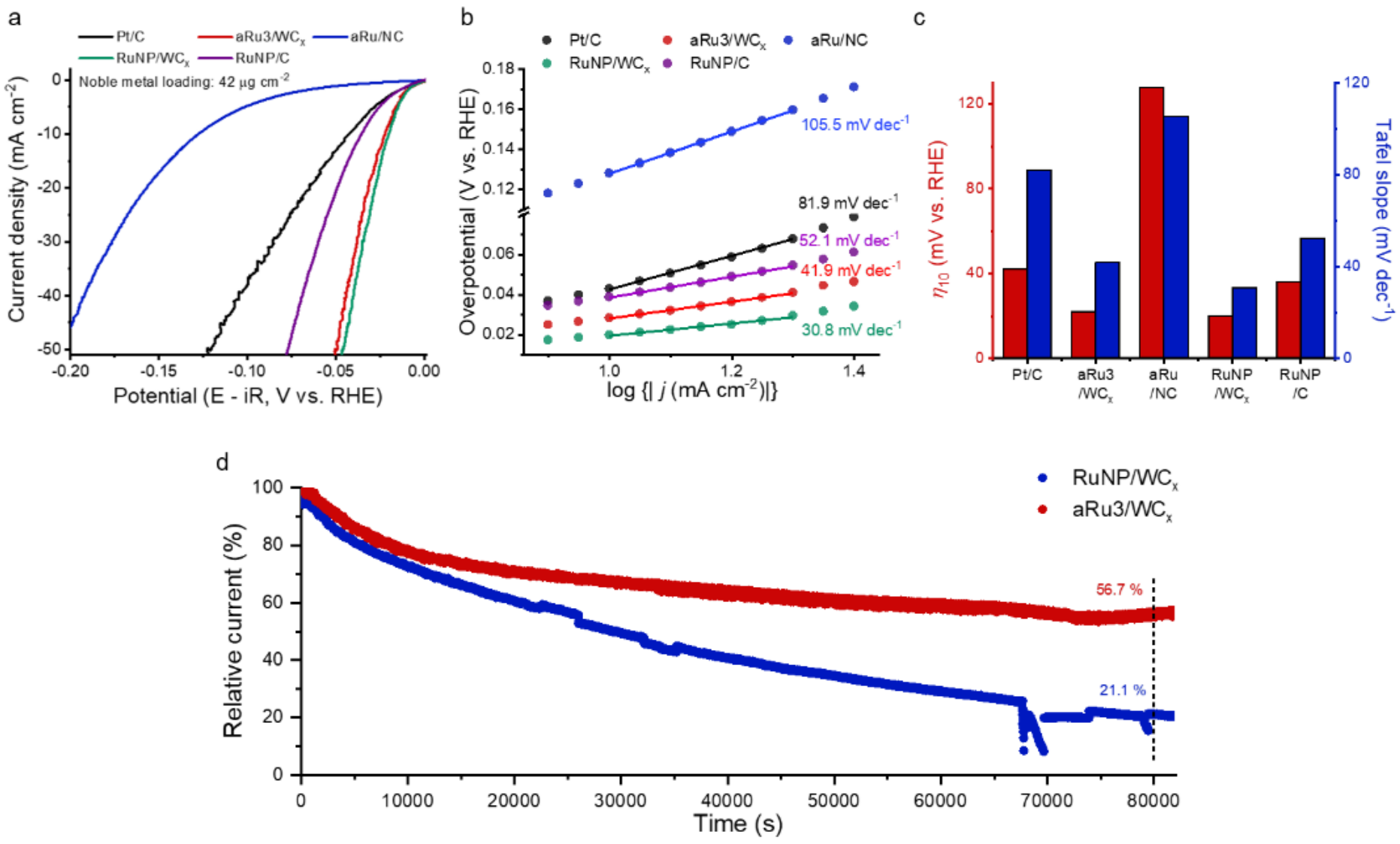

Supplementary Fig. 116 | Alkaline HER activity for aRu/NC, RuNP/WC$_x$, and RuNP/C. a, LSV curves, **b,** corresponding Tafel plot, **c,** corresponding η_{10} and Tafel slope, and **d,** relative current retention (%) during chronoamperometry at an overpotential of 50 mV over 80,000 s, H_2-saturated 1 M KOH solution. All potential referenced to the reversible hydrogen electrode (RHE). The RDE polarization curves were iR-corrected using the EIS-derived uncompensated solution resistance measured before each experiment.

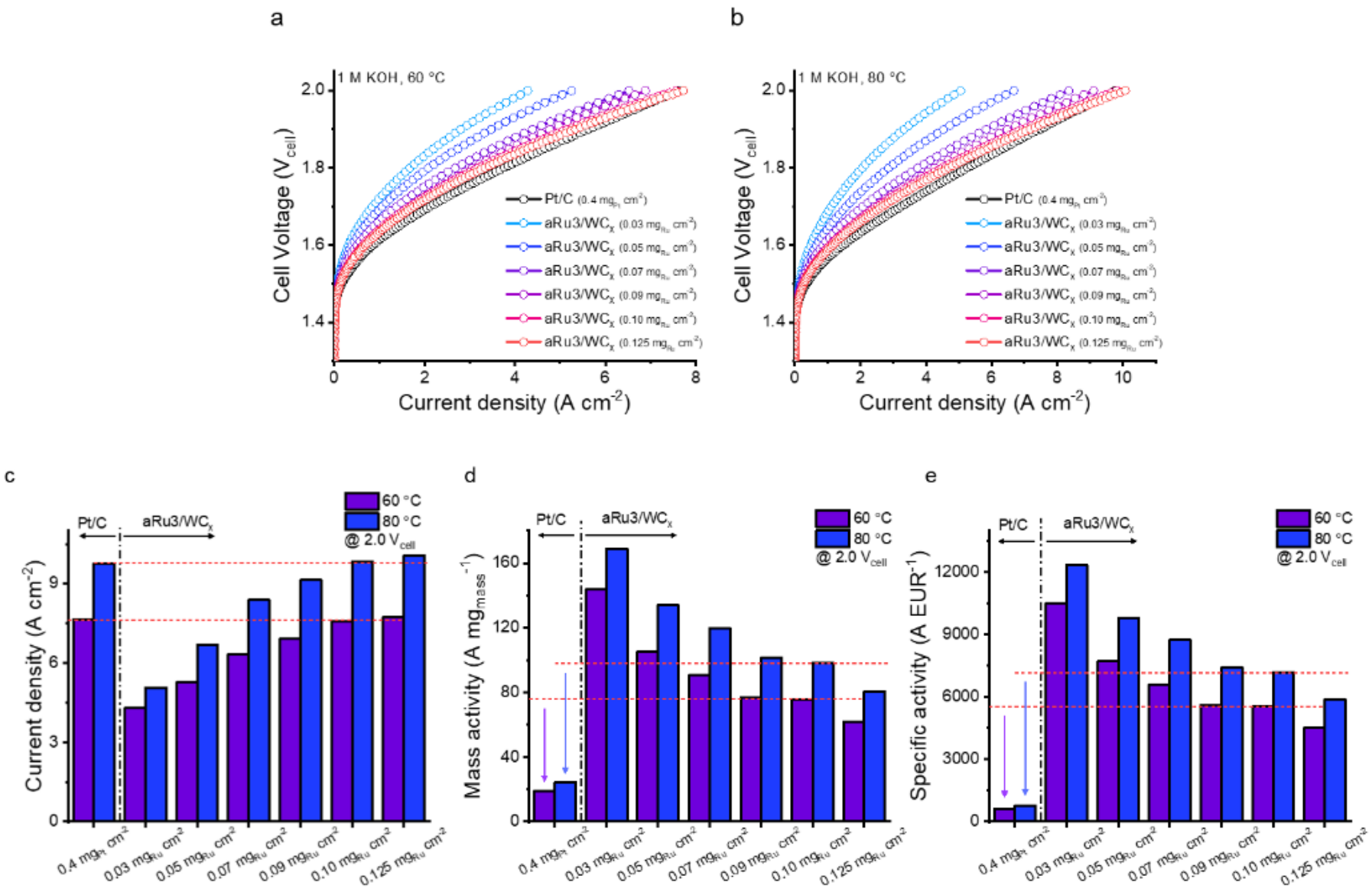

Supplementary Fig. 117 | Optimization of AEMWE performances of aRu3/WC$_x$ by varying Ru loadings. a,b, Polarization curves at (**a**) 60 ℃ and (**b**) 80 ℃. **c-e,** Performance metrics at 2.0 V_{cell} and the operating temperature of 60 ℃ or 80 ℃: (**c**) *j* (A cm^{-2}), (**d**) mass activity (A mg_{mass}^{-1}), and (**e**) *j* per noble metal cost (specific activity, A EUR^{-1}). The AEMWE polarization curves are shown without iR correction. All AEMWE voltages are reported as full-cell voltages (V_{cell})

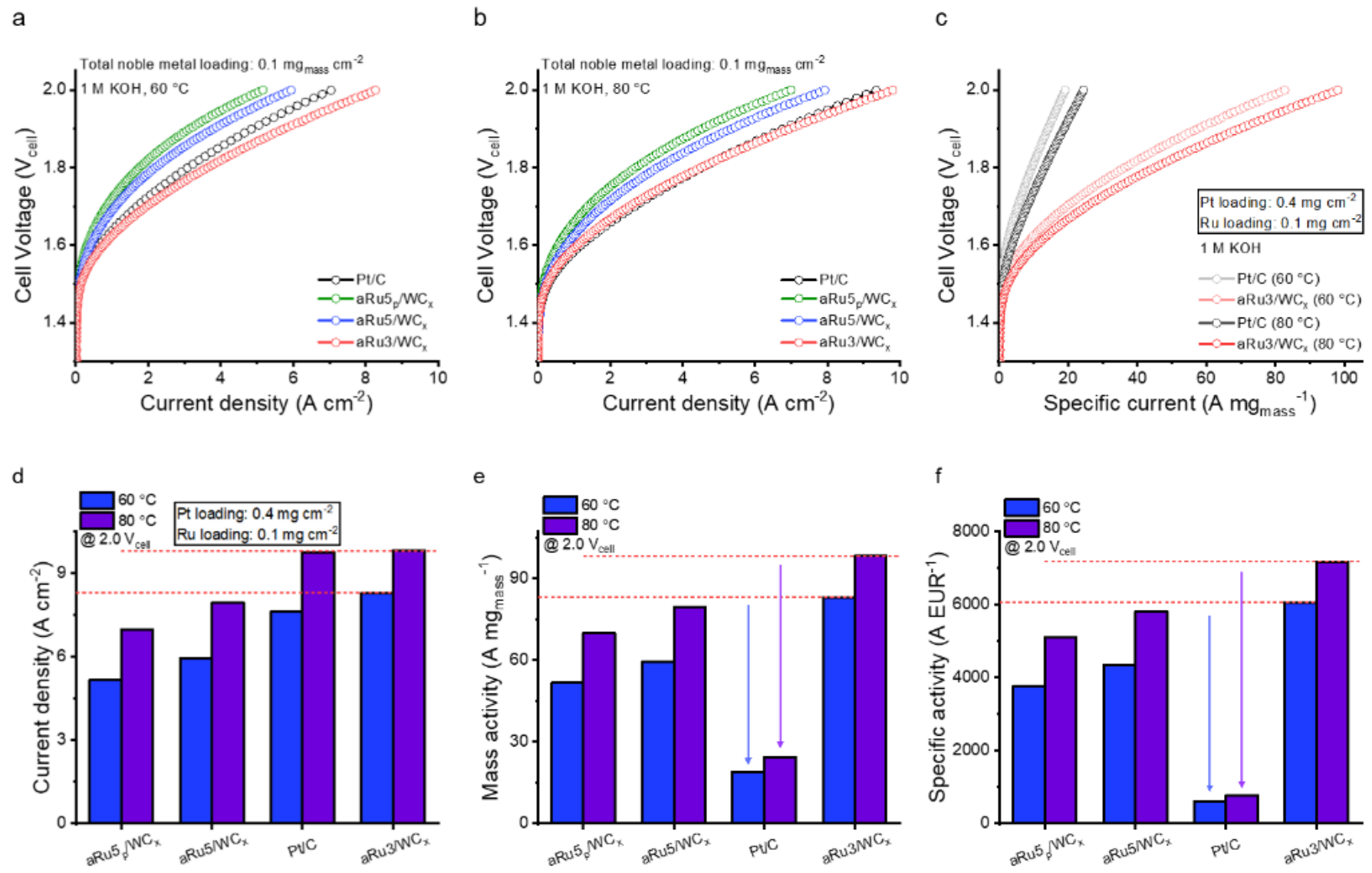

Supplementary Fig. 118 | Comparative AEMWE performance analysis of aRu3/WC$_x$. a,b, Polarization curves at (**a**) 60 ℃ and (**b**) 80 ℃ for aRu3/WC$_x$ (0.10 mg$_{Ru}$ cm^{-2}) and comparison samples, including commercial Pt/C (0.10 and 0.40 mg$_{Pt}$ cm^{-2}), aRu5/WC$_x$ (0.10 mg$_{Ru}$ cm^{-2}), and aRu5$_p$/WC$_x$ (0.10 mg$_{Ru}$ cm^{-2}). **c,** The corresponding polarization curves of specific activity for commercial Pt/C (0.40 mg$_{Pt}$ cm^{-2}) and aRu3/WC$_x$ (0.10 mg$_{Ru}$ cm^{-2}). **d-f,** Performance metrics at 2.0 V$_{cell}$ and the operating temperature of 60 ℃ or 80 ℃: (**d**) *j* (A cm^{-2}), (**e**) mass activity (A mg$_{mass}$$^{-1}$), and (**f**) *j* per noble metal cost (specific activity, A EUR^{-1}) of aRu3/WC$_x$, aRu5/WC$_x$, aRu5$_p$/WC$_x$ (0.10 mg$_{Ru}$ cm^{-2}), and commercial Pt/C (0.40 mg$_{Pt}$ cm^{-2}). The AEMWE polarization curves are shown without iR correction. All AEMWE voltages are reported as full-cell voltages (V$_{cell}$)

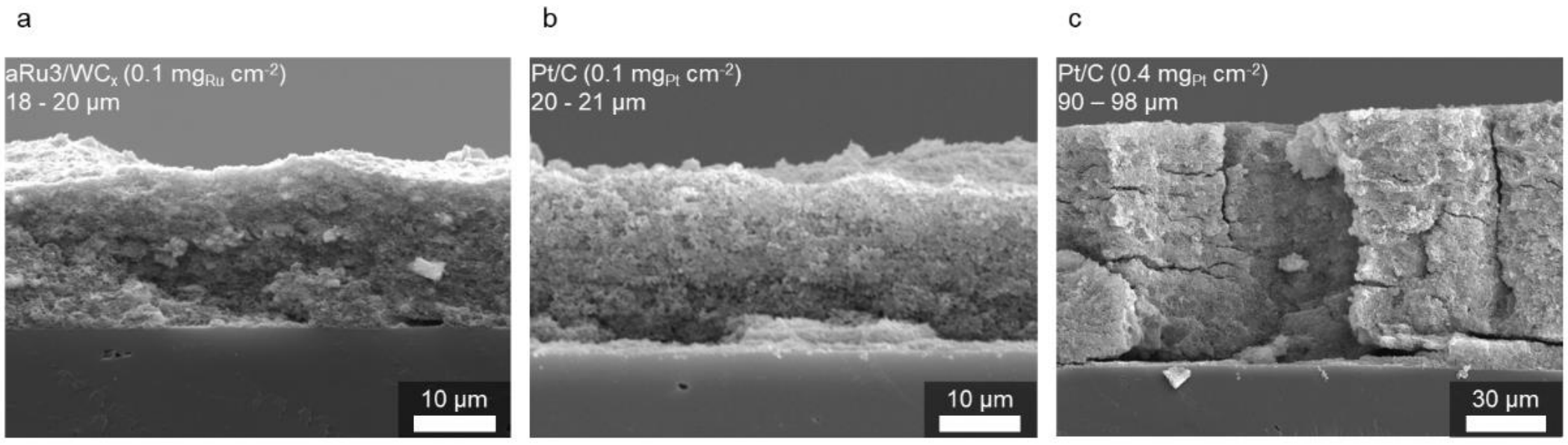

Supplementary Fig. 119 | Thickness of catalyst layers. a-c, Cross-sectional SEM images of spray-coated catalysts layers for (**a**) aRu3/WC$_x$ (0.10 mg$_{Ru}$ cm^{-2}), (**b**) Pt/C (0.10 mg$_{Pt}$ cm^{-2}), and (**c**) Pt/C (0.40 mg$_{Pt}$ cm^{-2}).

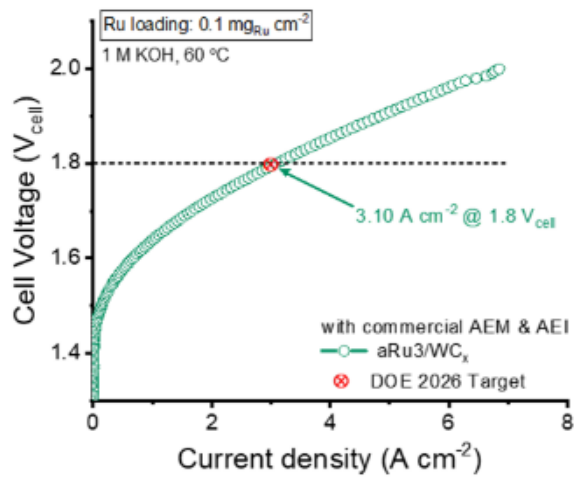

Supplementary Fig. 120 | AEMWE performance of aRu3/WC_x using commercial anion-exchange membrane (AEM) and anion-exchange ionomer (AEI) (PiperION-A). Polarization curve of AEMWE operated at 60 ℃ with aRu3/WC_x cathode (0.10 mg$_{Ru}$ cm^{-2} loading) and commercial membrane/ionomer components. The AEMWE polarization curves are shown without iR correction. All AEMWE voltages are reported as full-cell voltages (V_{cell})

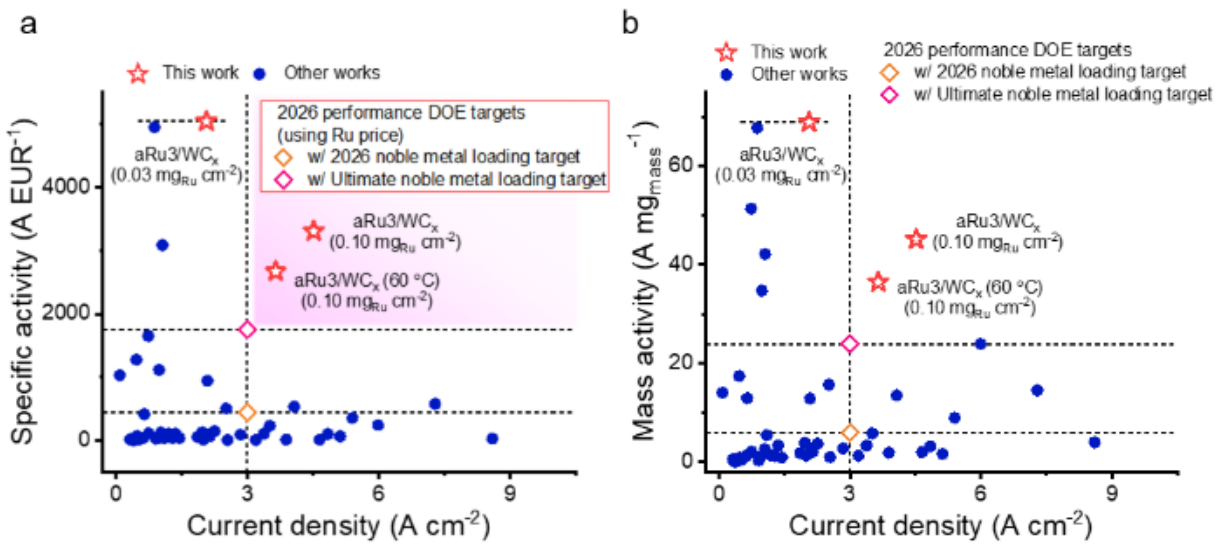

Supplementary Fig. 121 | Activity comparison of reported AEMWE electrocatalysts. Comparison of **a,** j (A cm^{-2}) and specific activity (A EUR^{-1}), and **b,** j and mass activity (A mg_{mass}^{-1}) for AEMWEs at 1.8 V_{cell}. The orange diamond represents the US department of energy (DOE) 2026 performance and noble metal loading targets, while the magenta diamond represents the US DOE 2026 performance and ultimate noble metal loading targets. The DOE specific activity target was estimated based on the price of Ru (Supplementary Table 1).

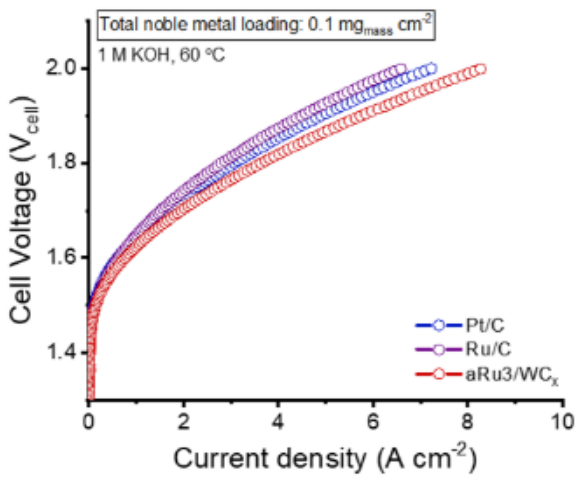

Supplementary Fig. 122 | Comparison of AEMWE performances for aRu3/WC$_x$ and commercial noble metal catalysts under identical total noble metal loading (0.10 mg$_{mass}$ cm^{-2}). Polarization curves of AEMWEs operated at 60 ℃ with aRu3/WC$_x$ (0.10 mg$_{Ru}$ cm^{-2}), commercial Pt/C (0.10 mg$_{Pt}$ cm^{-2}), and commercial Ru/C (0.10 mg$_{Ru}$ cm^{-2}) cathodes. The AEMWE polarization curves are shown without iR correction. All AEMWE voltages are reported as full-cell voltages (V_{cell})

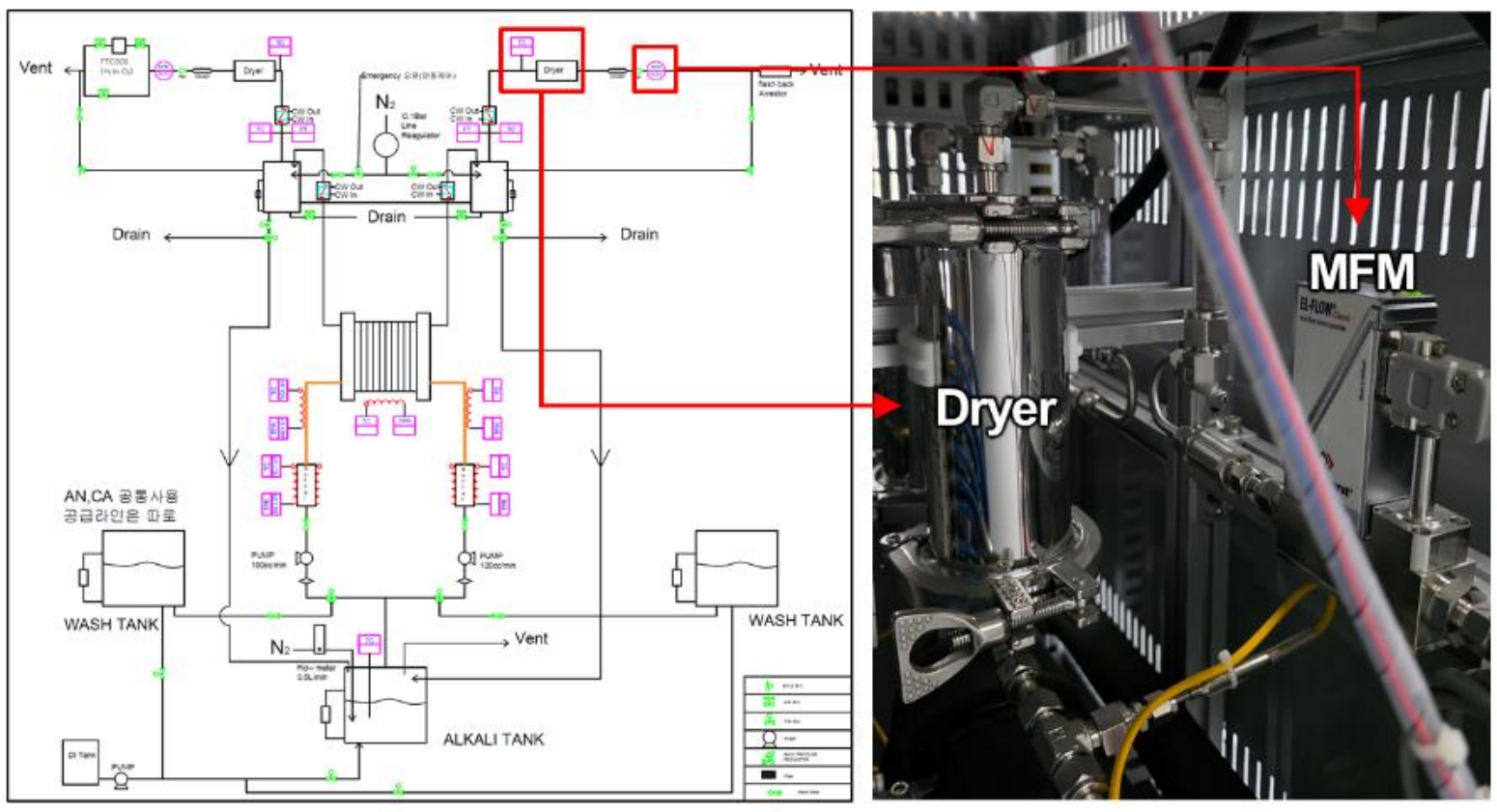

Supplementary Fig. 123 | Experimental setup for hydrogen quantification under high-current-density AEMWE operation. (left) Schematic flow diagram of the electrolyzer system. (right) Photograph of the experimental setup showing the dryer and mass flow meter (MFM) installed at the cathode outlet for real-time hydrogen flow measurement.

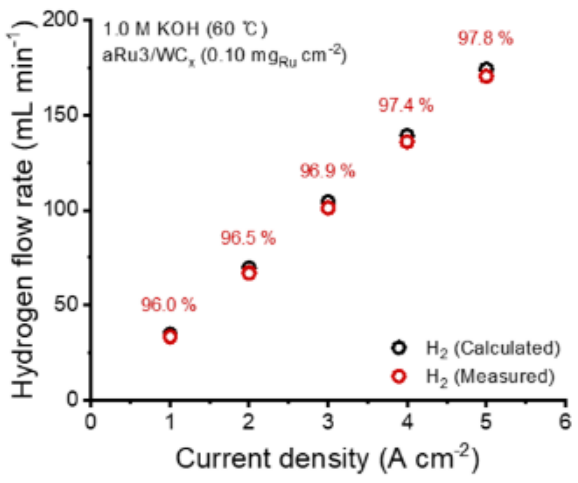

Supplementary Fig. 124 | Validation of Faradaic efficiency under high-current-density AEMWE operation. Comparison between the experimentally measured hydrogen evolution rate and the theoretical values calculated from Faraday's law as a function of current density during chronopotentiometric operation using aRu3/WC_x (0.10 mg_{Ru} cm^{-2}) in 1.0 M KOH at 60 ℃.

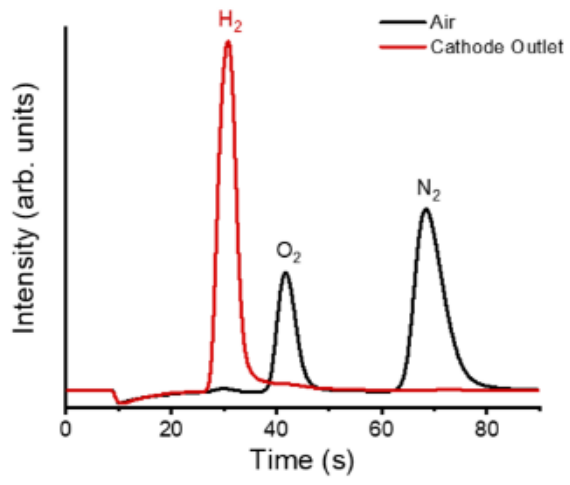

Supplementary Fig. 125 | Gas chromatography (GC) analysis. GC analysis of the cathode outlet stream under AEMWE operation. The cathode outlet stream is dominated by H_2 with no detectable impurity peaks, while a minor O_2 peak is attributed to oxygen crossover through the membrane.

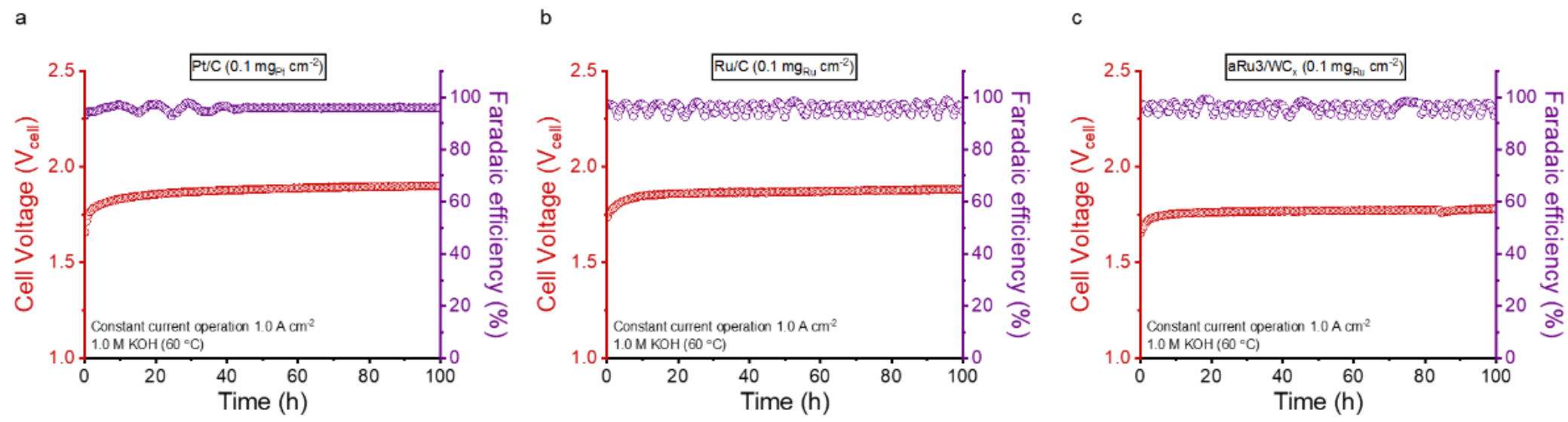

Supplementary Fig. 126 | AEMWE durability comparison of aRu3/WC$_x$ and commercial noble metal catalysts under identical total noble metal loading (0.10 mg$_{mass}$ cm^{-2}). a-c, Cell voltage profiles (red) and corresponding H_2 Faradaic efficiency (purple) during AEMWE operation (60 ℃, 1 M KOH) under a constant current density of 1.0 A cm^{-2} for 100 h, using (**a**) commercial Pt/C (0.10 mg$_{Pt}$ cm^{-2}), (**b**) commercial Ru/C (0.10 mg$_{Ru}$ cm^{-2}), and (**c**) aRu3/WC$_x$ (0.10 mg$_{Ru}$ cm^{-2}) cathodes. The AEMWE polarization curves are shown without iR correction. All AEMWE voltages are reported as full-cell voltages (V$_{cell}$)

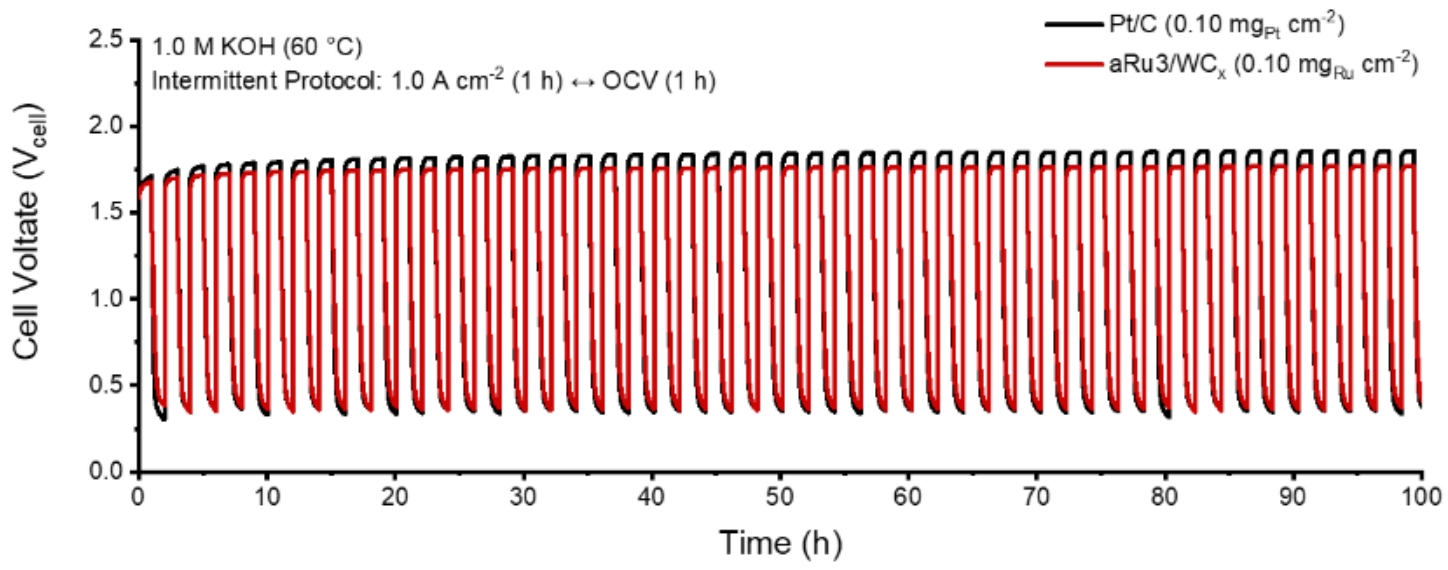

Supplementary Fig. 127 | Intermittent AEMWE operation stability test. Cell voltage profiles of AEMWEs employing commercial Pt/C and aRu3/WC_x cathodes during intermittent operation by alternating between galvanostatic operation at 1.0 A cm^{-2} and OCV every 1 h for a total operation time of 100 h in 1.0 M KOH at 60 ℃. All AEMWE voltages are reported as full-cell voltages (V_{cell})

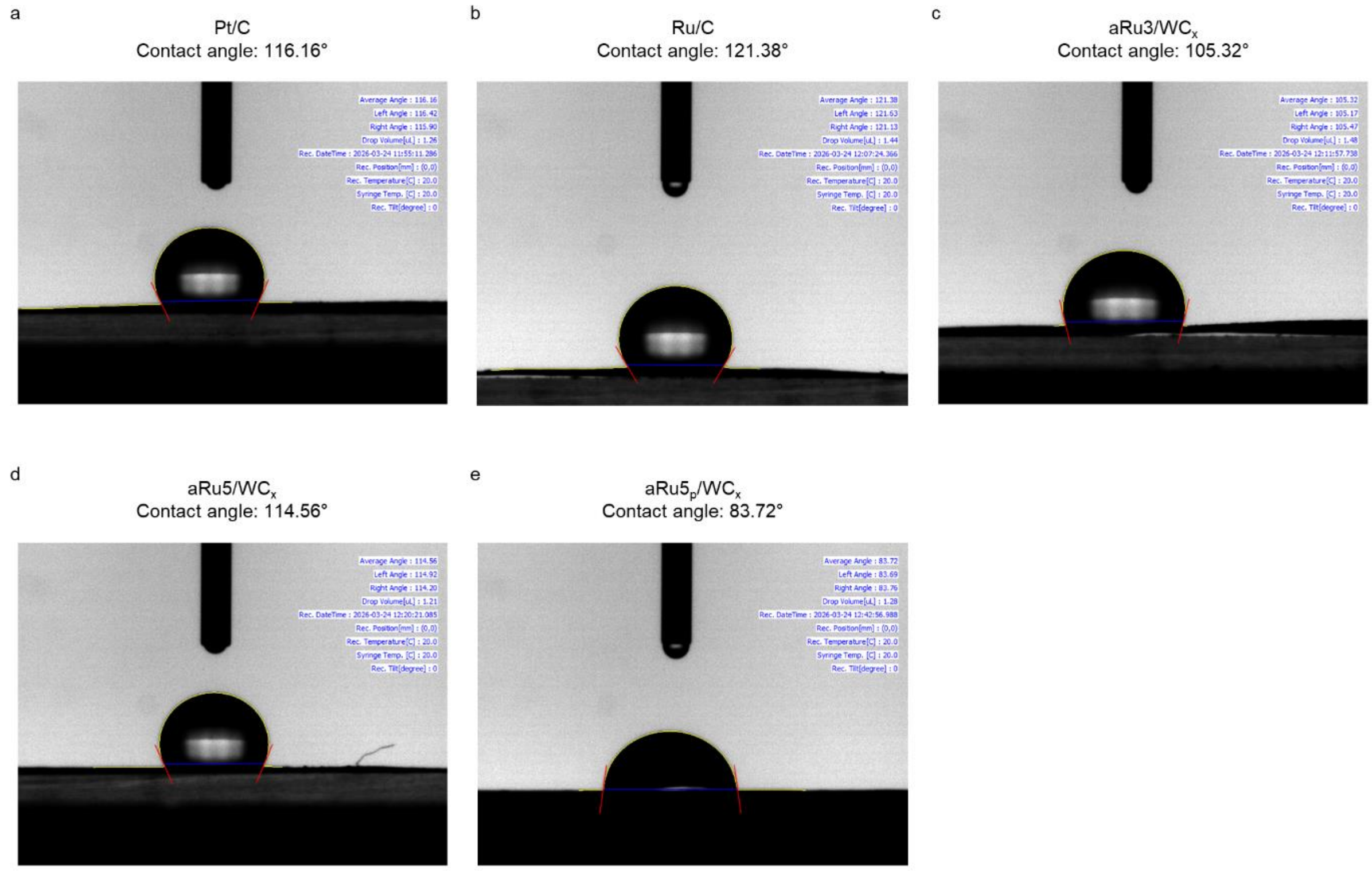

Supplementary Fig. 128 | Water contact angle analysis of catalyst layers. a-e, Water contact angle measurements for catalyst layers prepared with (**a**) commercial Pt/C, (**b**) commercial Ru/C, (**c**) aRu3/WC_x, (**d**) aRu5/WC_x, and (e) $aRu5_p$/WC_x. All catalyst layers were prepared with an identical noble metal loading of 0.10 mg_{mass} cm^{-2} on non-porous Grafoil substrates.

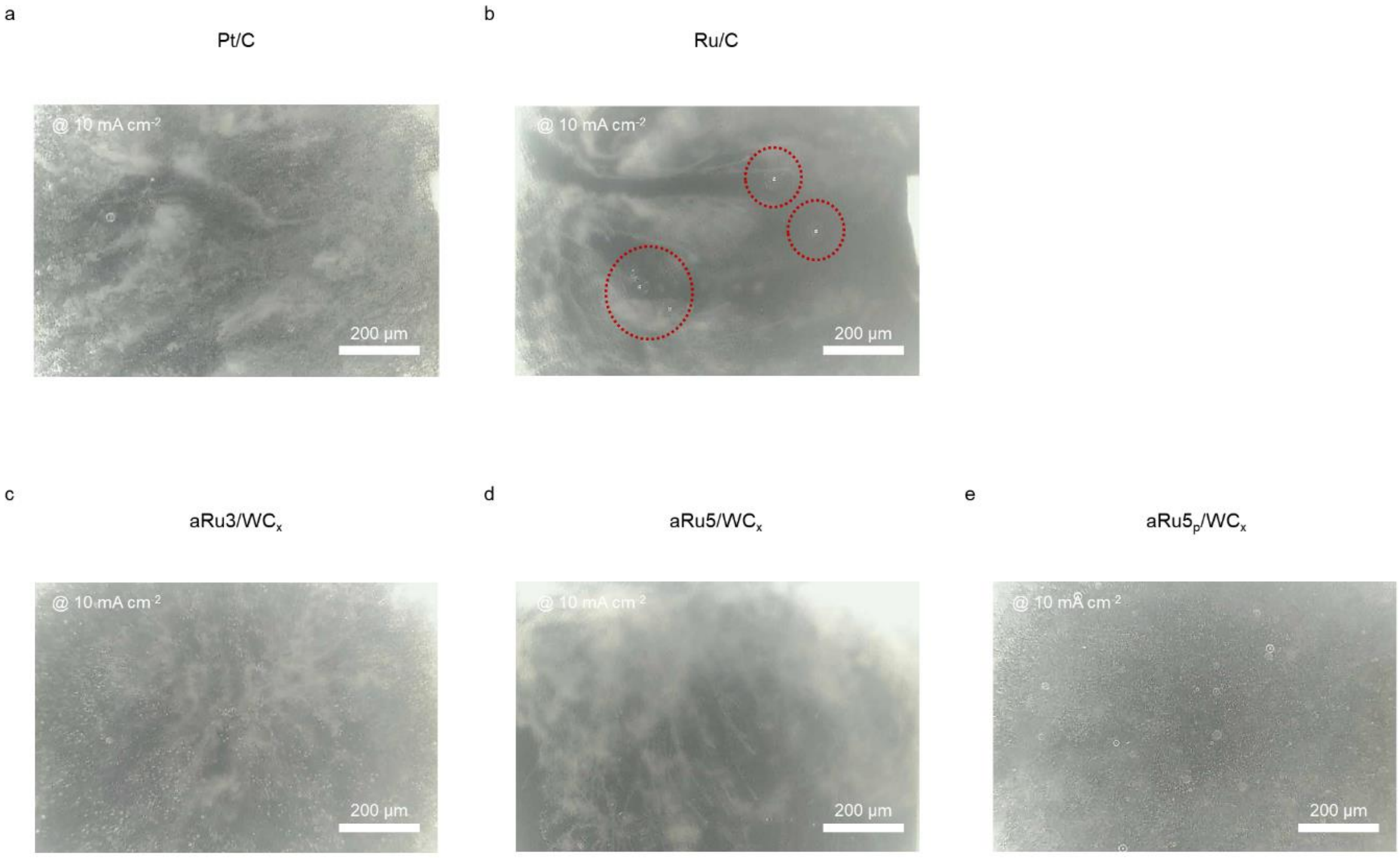

Supplementary Fig. 129 | In situ optical visualization of hydrogen bubble evolution on catalyst layers under alkaline HER conditions. a-e, Optical images of gas bubbles generated on catalyst layers prepared with (**a**) commercial Pt/C, (**b**) commercial Ru/C, (**c**) aRu3/WC_x, (**d**) aRu5/WC_x, and (**e**) $aRu5_p/WC_x$ at a current density of 10 mA cm^{-2}. Red dotted circles indicate localized bubble accumulation observed on the Ru/C catalyst layer.

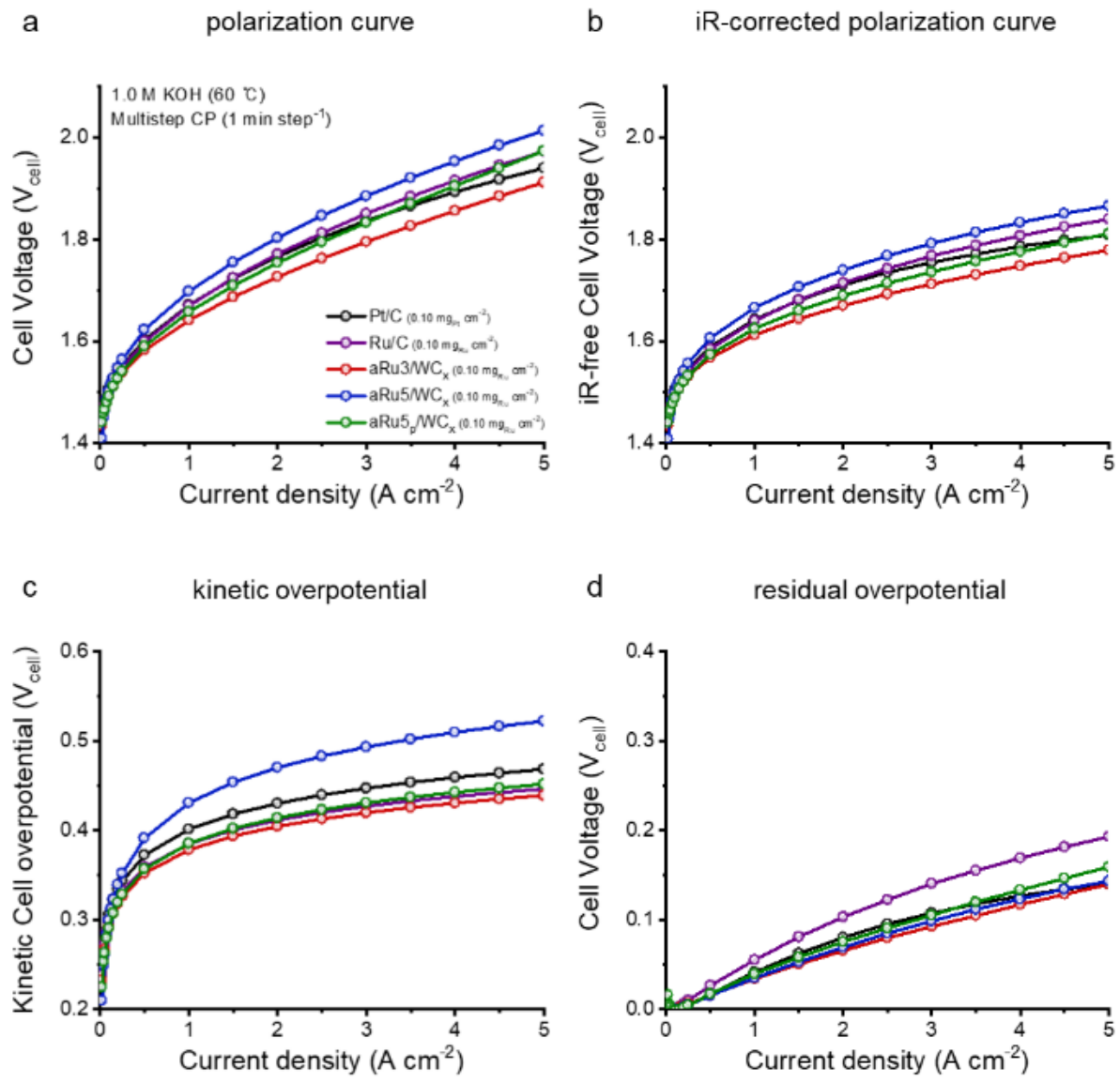

Supplementary Fig. 130 | Overpotential breakdown analysis of AEMWE performance. a, Single-cell polarization curves of AEMWEs using commercial Pt/C, commercial Ru/C, aRu3/WC_x, aRu5/WC_x, and aRu5$_p$/WC_x cathodes in 1.0 M KOH at 60 ℃ under multistep chronopotentiometric (CP) operation. Corresponding **b,** iR-corrected polarization curves, **c,** kinetic overpotentials, and **d,** residual overpotentials derived from electrochemical impedance spectroscopy (EIS)-based analysis. The ohmic resistance was determined from EIS at each current density and used for the overpotential breakdown analysis. All AEMWE voltages are reported as full-cell voltages (V_{cell})

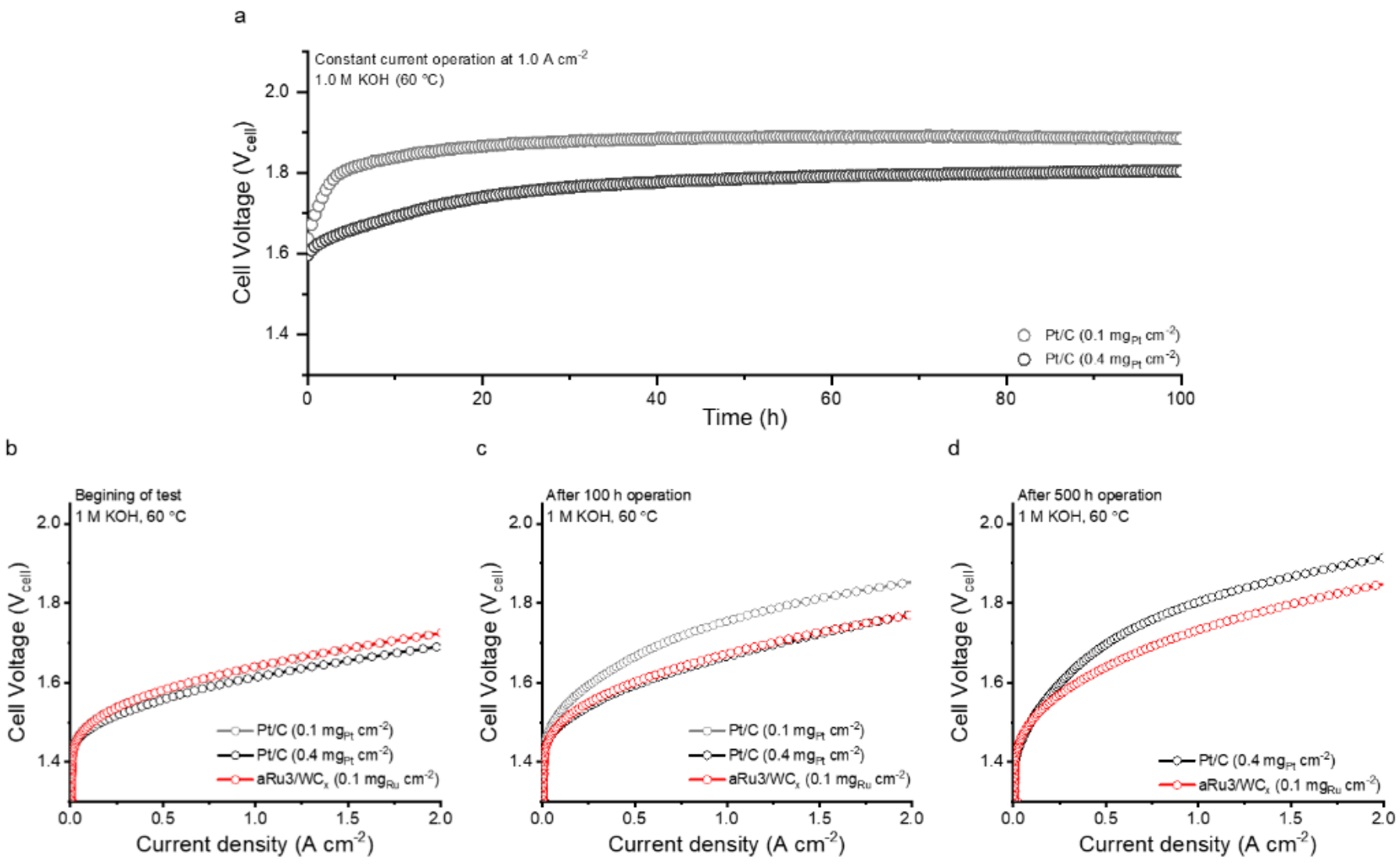

Supplementary Fig. 131 | Short-term and long-term durability of AEMWEs with different noble metal loadings (aRu3/WC$_x$: 0.10 mg$_{Ru}$ cm^{-2}, Pt/C: 0.10 and 0.40 mg$_{Pt}$ cm^{-2}). a, Cell voltage changes during the durability test at 1.0 A cm^{-2} and 60 ℃ for benchmark Pt/C. **b-d,** I-V polarization curves of AEMWEs at 60 ℃ before (BOT) and after (EOT) the durability test. The AEMWE polarization curves are shown without iR correction. All AEMWE voltages are reported as full-cell voltages (V$_{cell}$)

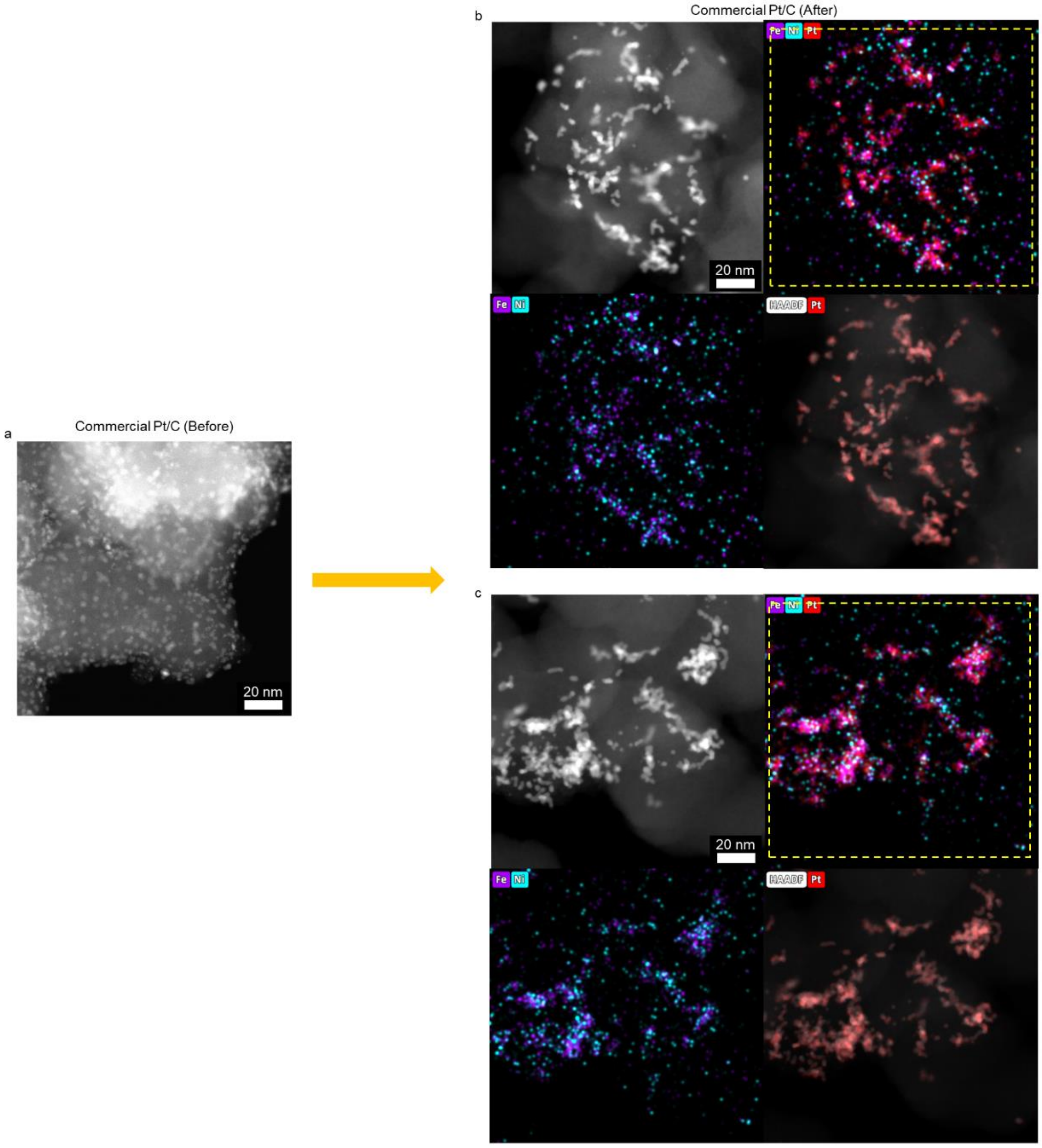

Supplementary Fig. 132 | STEM analysis of commercial Pt/C (0.10 mg_{Pt} cm^{-2}) before and after the AEMWE durability test. **a,** High-resolution HAADF-STEM image of commercial Pt/C before the AEMWE durability test. **b,c,** High-resolution STEM images and the corresponding EDS elemental mappings for platinum (red), iron (purple), and nickel (cyan) along with their overlay images, for commercial Pt/C after the AEMWE durability test. The detection of Fe and Ni species indicates partial migration and reduction of dissolved Fe/Ni ions from the anode during AEMWE operation, a phenomenon commonly reported in AEMWE.

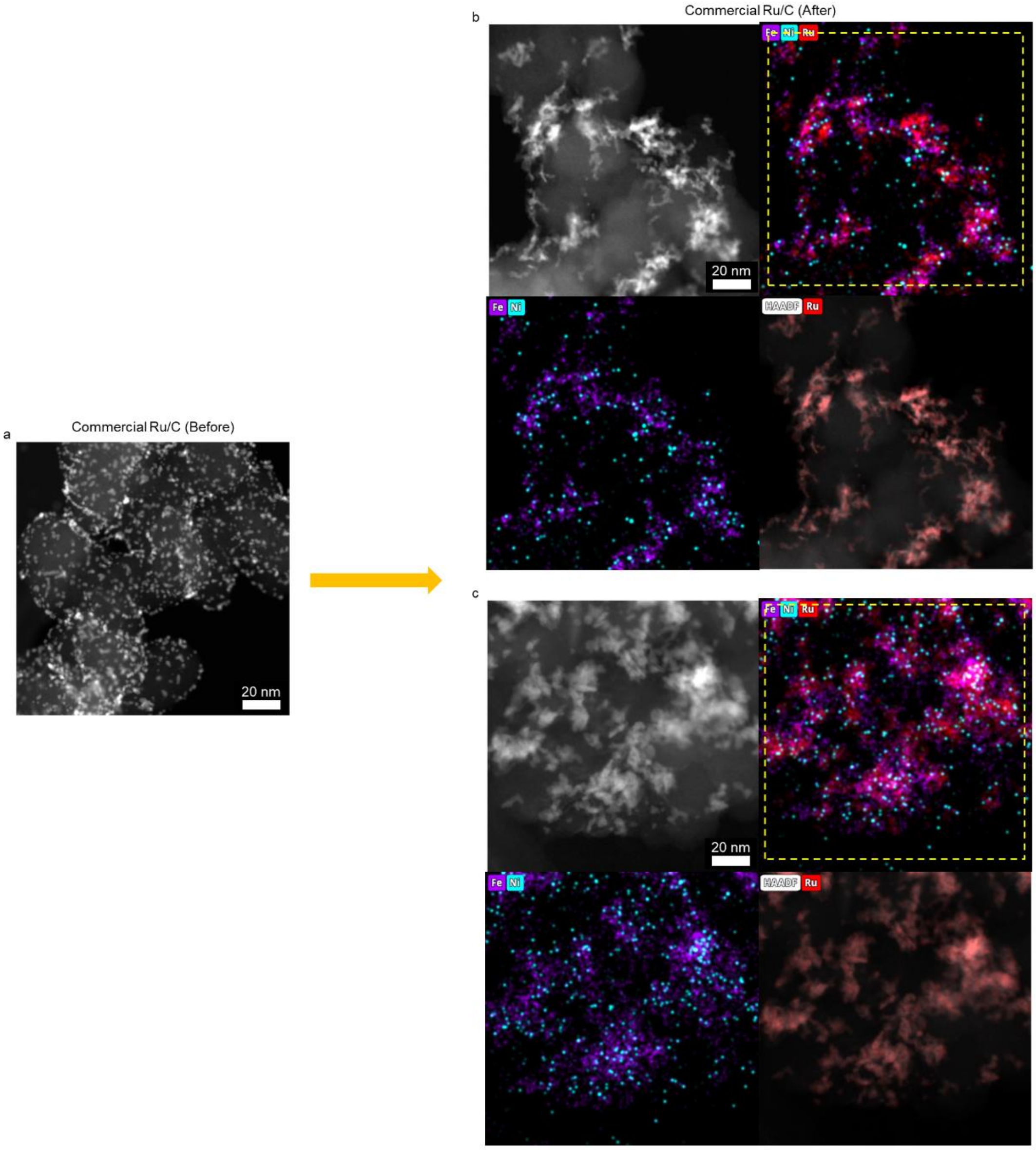

Supplementary Fig. 133 | STEM analysis of commercial Ru/C (0.10 mg_{Ru} cm^{-2}) before and after the AEMWE durability test. **a,** High-resolution HAADF-STEM image of commercial Ru/C before the AEMWE durability test. **b,c,** High-resolution STEM images and the corresponding EDS elemental mappings for ruthenium (red), iron (purple), and nickel (cyan) along with their overlay images, for commercial Ru/C after the AEMWE durability test. The detection of Fe and Ni species indicates partial migration and reduction of dissolved Fe/Ni ions from the anode during AEMWE operation, a phenomenon commonly reported in AEMWE.

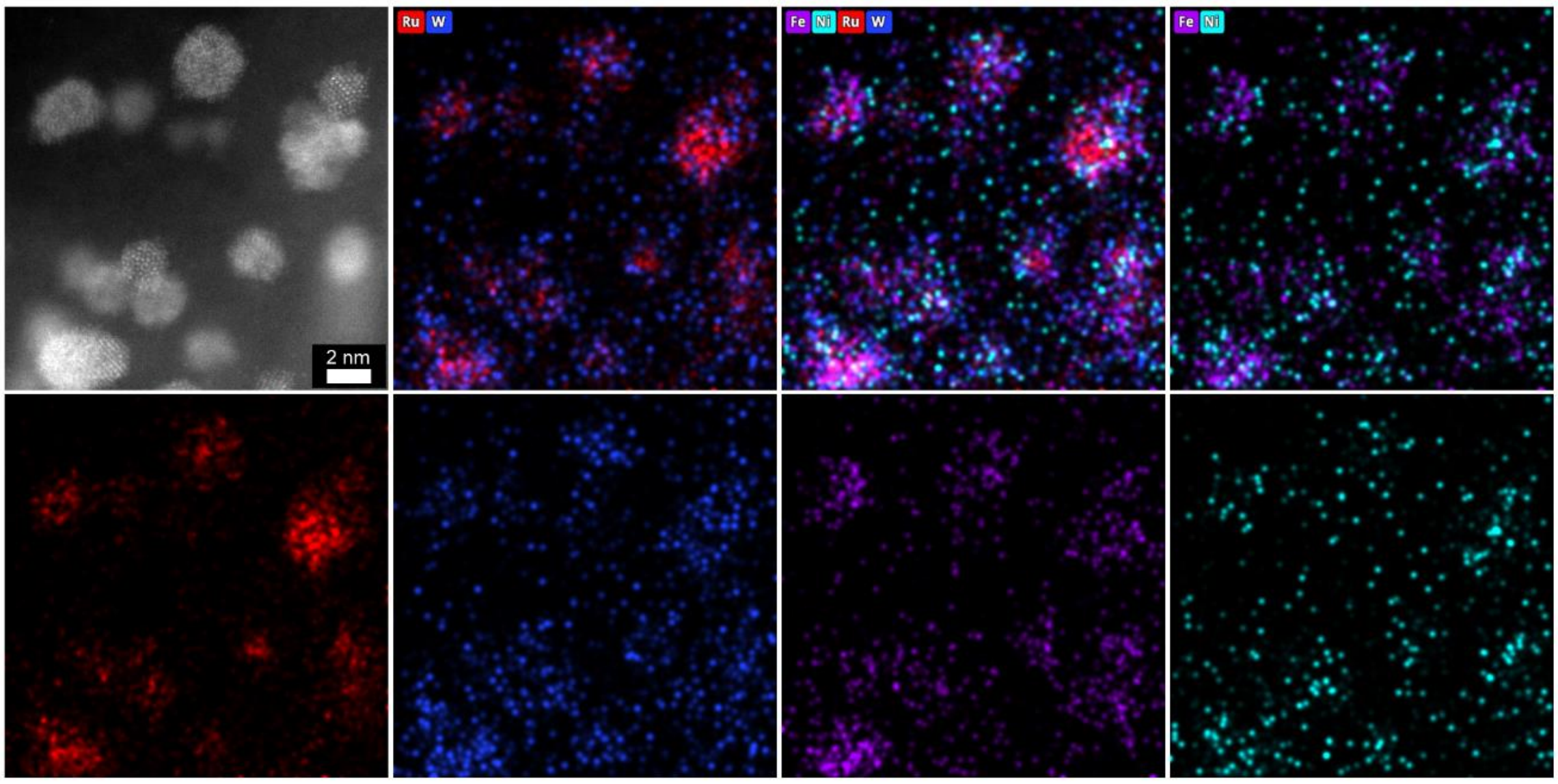

Supplementary Fig. 134 | STEM analysis of aRu3/WC_x (0.10 mg_{Ru} cm^{-2}) after the AEMWE durability test. High-resolution HAADF-STEM image and the corresponding EDS elemental mappings for tungsten (blue), ruthenium (red), iron (purple), and nickel (cyan) along with their overlay images. The detection of Fe and Ni species on the aRu3/WC_x surface indicates partial migration and reduction of dissolved Fe/Ni ions from the anode during AEMWE operation, a phenomenon commonly reported in AEMWE.

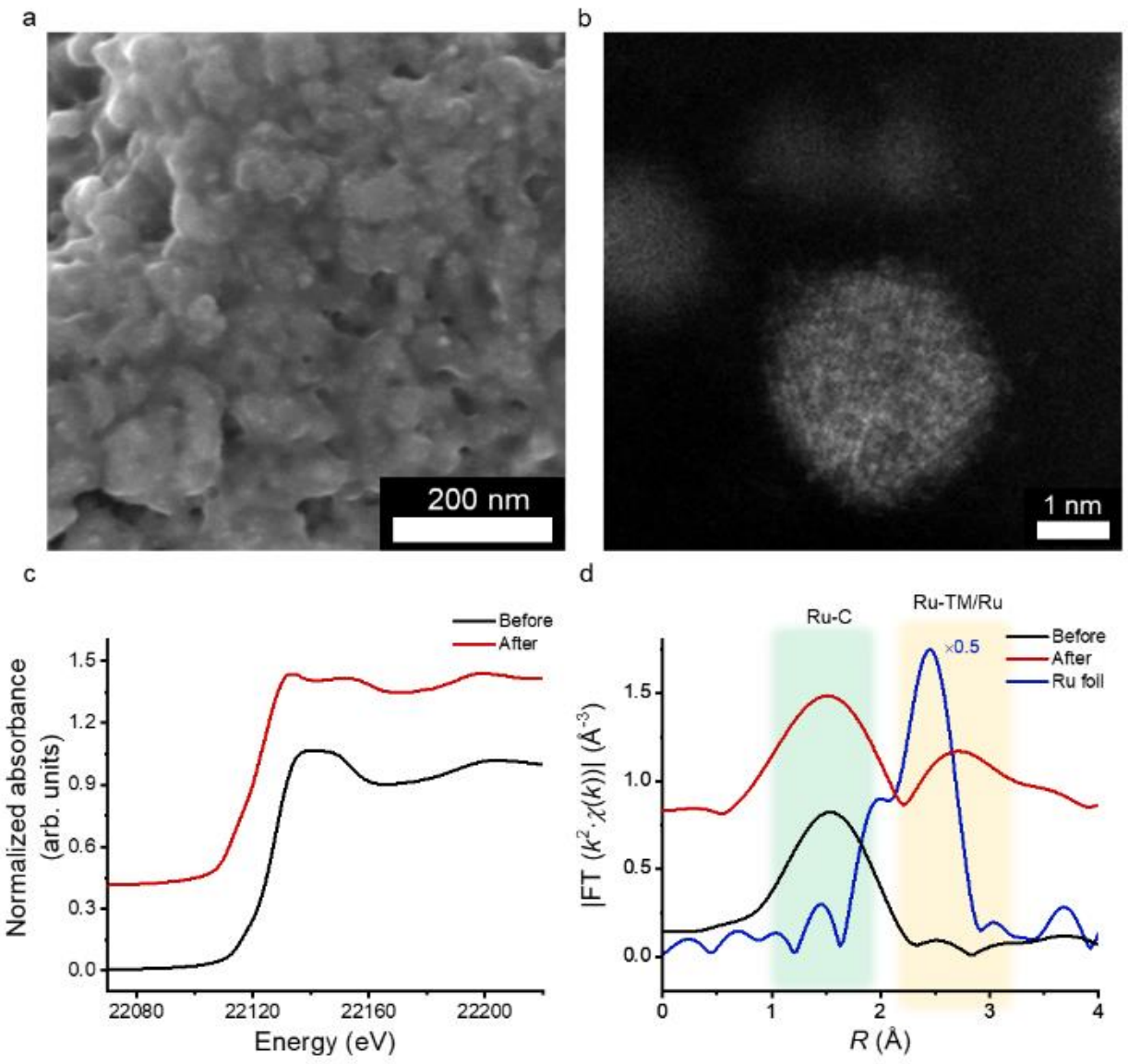

Supplementary Fig. 135 | Material characterization of aRu3/WC$_x$ after the AEMWE durability test. a, SEM image and **b,** high-resolution HAADF-STEM images of the used aRu3/WC$_x$ catalyst. **c,d,** Comparison of aRu3/WC$_x$ before and after the AEMWE durability test via (**c**) Ru K-edge XANES spectra and (**d**) FT-EXAFS spectra. (*R*: the radial distance in Fourier-transformed EXAFS *R* space)

Supplementary Tables

Supplementary Table 1 | Average costs of representative noble metals. 3-month average prices per unit gram (EUR g^{-1}) for Platinum, Ruthenium, and Iridium (June 1, 2024 to September 1, 2024, sourced from Heraeus.com).

Noble Metal	Platinum	Ruthenium	Iridium
Price (EUR g^{-1})	31.15	13.68	159.06

Supplementary Table 2 | Molecular interactions for BCP-precursor systems. The polymer solvation energy (ΔE_{solv}) (kcal mol^{-1}) of Ru precursors (HPL Ru, HPB Ru) in specific BCP microphases (PEO, PS).

Without considering the interaction between Ru precursor (Precursor solubility)

Moiety of block-copolymer	*E* (polymer + HPB Ru)	*E* (HPB Ru)	*E* (polymer)	ΔE_{solv}
PS	3843.1	384.5	3475.2	-16.6
PEO	1914.2	384.5	1510.7	19

Moiety of block-copolymer	*E* (polymer + HPL Ru)	*E* (HPL Ru)	*E* (polymer)	ΔE_{solv}
PS	3505.4	23.1	3475.2	7.1
PEO	1395.7	23.1	1510.7	-138.1

Supplementary Table 3 | Molecular interactions for BCP-precursor systems. The dispersion propensity (ΔE_{disp}) (kcal mol^{-1}) of Ru precursors (HPL Ru, HPB Ru) in specific BCP microphases (PEO, PS).

With considering the interaction between Ru precursor (Precursor dispersion)

Moiety of block-copolymer	*E* (polymer + HPB Ru)	*E* (HPB Ru)	*E* (polymer)	ΔE_{disp}
PS	3843.1	388.815	3475.2	-20.915
PEO	1914.2	388.815	1510.7	14.685

Moiety of block-copolymer	*E* (polymer + HPL Ru)	*E* (HPL Ru)	*E* (polymer)	ΔE_{disp}
PS	3505.4	-811.365	3475.2	841.565
PEO	1395.7	-811.365	1510.7	696.365

Supplementary Table 4 | Comparison of experimental and calculated values. Comparison of the experimentally determined (EXP*) cohesive energy of Ru[30] and density functional theory (DFT) calculated bulk modulus and lattice parameters of WC with molecular dynamic (MD)-calculated values.

Properties	EXP or DFT	MD
WC bulk modulus (GPa)	307.9 (DFT)	308.8
WC lattice parameter (Å)	a, b, c = 8.82 (DFT)	a, b, c = 8.67
E_{coh} (eV atom^{-1})	6.74 (EXP*)	6.71

Supplementary Table 5 | Lattice parameters. Lattice parameters used for MD simulation.

The number of Ru	Lattice parameter
1-116	a, b = 35.135 Å, c = 35.000 Å, α, β, γ = 90°
163-235	a, b = 70.270 Å, c = 70.000 Å, α, β, γ = 90°
321-37,414	a, b = 210.810 Å, c = 150.000 Å, α, β, γ = 90°

Supplementary Table 6 | Average CN of Ru-Ru as a function of the number of Ru atoms in Ru ALN. The dependence of Ru-Ru CN on the number of Ru atoms, based on MD simulations. (CN: coordination number)

Number of Ru	Ru-Ru Coordination Number	Number of Ru	Ru-Ru Coordination Number
1	0	55	4.051
2	1	116 (r = 7 Å)	4.338
3	1.932	163 (r = 8 Å)	4.450
4	2.110	200 (r = 8.5 Å)	4.493
5	2.170	235 (r = 9 Å)	3.981
6	2.411	321 (r = 10 Å)	4.069
13	3.174	4729 (r = 25 Å)	6.985
		37414 (r = 50 Å)	10.200

Supplementary Table 7 | Noble metal loading amounts for various catalysts. Metal weight composition was calculated using both metal mass ratio (analyzed by X-ray fluorescence) and C, H, N, S, and O contents (analyzed by elemental analyzer) for samples prepared via thermodynamic stabilization (aRu*N*/WC_x), samples prepared by IWI at different annealing *T* ($Ru_p/WC_x\_H_2$, $Ru/WC_x\_H_2$, Ru_p/WC_x_Ar, Ru/WC_x_Ar), and others (Ru/WC_x_R, Ru/C_R, RuNP/WC_x, RuNP/C, aRu/NC, aPt3/WC_x, aIr3/WC_x).

Noble Metal Contents	Catalysts					
	aRu1/WC_x	aRu2/WC_x	aRu3/WC_x	aRu4/WC_x	aRu5/WC_x	aRu5$_p$/WC_x
wt %	1.61	4.88	7.10	9.04	10.16	10.70
Ru : (Ru+W) atomic ratio	0.037	0.113	0.171	0.204	0.229	0.255

Noble Metal Contents	Catalysts					
	$Ru_p/WC_x\_H_2$	$Ru/WC_x\_H_2$	Ru_p/WC_x_Ar	Ru/WC_x_Ar	Ru/WC_x_R	Ru/C_R
wt %	8.48	9.65	8.44	8.82	18.18	15.10
Ru : (Ru+W) atomic ratio	0.176	0.197	0.173	0.183	0.345	-

Noble Metal Contents	Catalysts				
	RuNP/WC_x	RuNP/C	aRu/NC	aPt3/WC_x	aIr3/WC_x
wt %	8.00	16.12	5.47	11.89	11.61

Supplementary Table 8 | Variation of structural parameters based on the type of Ru precursors. Structural parameters extracted from the Ru K-edge EXAFS fitting results. CN: coordination number, *R*: interatomic distance, and σ^2: Debye-Waller factor. The goodness of fit is represented by the R-factor. (weighting: k^2)

Sample	Shell	CN	*R* (Å)	σ^2 (10^{-3} Å^2)	R-factor
Ru	Ru-Ru	6*	2.65 ± 0.01	2.56	0.007
	Ru-Ru	6*	2.70 ± 0.01		
RuO_2	Ru-O	2*	1.94 ± 0.01	0.73	0.014
	Ru-O	4*	1.98 ± 0.01		
aRu5/WC_x	Ru-C	3.54 ± 0.34	2.11 ± 0.01	7.68	0.012
	Ru-Ru	2.29 ± 0.31	2.75 ± 0.01	9.99	
	Ru-W	1.90 ± 0.55	2.84 ± 0.01		
aRu5_p/WC_x	Ru-O	1.51 ± 0.51	1.99 ± 0.03	6.88	0.019
	Ru-Ru	3.98 ± 0.41	2.70 ± 0.01	4.16	
	Ru-W	1.99 ± 0.65	2.72 ± 0.01		
aRu5/WO_x	Ru-O	4.02 ± 0.15	2.03 ± 0.00	6.60	0.003
	Ru-Ru	2.04 ± 0.23	2.66 ± 0.01	10.51	
	Ru-W	1.03 ± 0.37	2.68 ± 0.01		
aRu5_p/WO_x	Ru-O	2.56 ± 0.54	2.00 ± 0.02	9.21	0.012
	Ru-Ru	4.44 ± 0.34	2.67 ± 0.01	3.84	
	Ru-W	1.47 ± 0.56	2.69 ± 0.01		

Structural parameters extracted from the EXAFS fitting of reference metal foil. CN is the coordination number; *R* is the interatomic distance; σ^2 is the Debye-Waller factor (a measure of the static and thermal disorder in absorber-scatterer distance); ΔE_0 is edge energy shift (the zero kinetic energy difference between experiment and theoretical model); R-factor value is related with the goodness of the fitting.

* Value was assigned in curving fitting based on standard structure

R_{bkg} was set to 1.0 in background removal to remove the noise oscillation below 1 Å in *R*-space

S_0^2 value (0.744) was calculated from the fitting of metal foil with standard metal structure

Supplementary Table 9 | Structural parameters for samples obtained via thermodynamic stabilization (aRu*N*/WC_x). Structural parameters extracted from the Ru K-edge EXAFS fitting results. CN: coordination number, *R*: interatomic distance, and σ^2: Debye-Waller factor. The goodness of fit is represented by the R-factor. (weighting: k^2)

Sample	Shell	CN	*R* (Å)	σ^2 (10^{-3} Å^2)	R-factor
Ru	Ru-Ru	6*	2.65 ± 0.01	2.56	0.007
	Ru-Ru	6*	2.70 ± 0.01		
RuO_2	Ru-O	2*	1.94 ± 0.01	0.73	0.014
	Ru-O	4*	1.98 ± 0.01		
aRu5/WC_x	Ru-C	3.54 ± 0.34	2.11 ± 0.01	7.68	0.012
	Ru-Ru	2.29 ± 0.31	2.75 ± 0.01	9.99	
	Ru-W	1.90 ± 0.55	2.84 ± 0.01		
aRu4/WC_x	Ru-C	3.32 ± 0.27	2.08 ± 0.01	3.14	0.009
	Ru-Ru	2.00 ± 0.22	2.72 ± 0.01	5.64	
	Ru-W	2.12 ± 0.38	2.82 ± 0.01		
aRu3/WC_x	Ru-C	4.01 ± 0.17	2.11 ± 0.00	6.18	0.003
	Ru-Ru	1.72 ± 0.16	2.76 ± 0.01	8.78	
	Ru-W	2.28 ± 0.28	2.86 ± 0.01		
aRu2/WC_x	Ru-C	4.83 ± 0.27	2.10 ± 0.01	7.68	0.005
	Ru-Ru	1.69 ± 0.20	2.74 ± 0.01	7.77	
	Ru-W	2.71 ± 0.34	2.84 ± 0.01		
aRu1/WC_x	Ru-C	4.78 ± 0.48	2.10 ± 0.01	4.22	0.017
	Ru-Ru	1.56 ± 0.67	2.74 ± 0.04	12.50	
	Ru-W	2.86 ± 1.09	2.84 ± 0.04		

Structural parameters extracted from the EXAFS fitting of reference metal foil. CN is the coordination number; *R* is the interatomic distance; σ^2 is the Debye-Waller factor (a measure of the static and thermal disorder in absorber-scatterer distance); ΔE_0 is edge energy shift (the zero kinetic energy difference between experiment and theoretical model); R-factor value is related with the goodness of the fitting.

* Value was assigned in curving fitting based on standard structure

R_{bkg} was set to 1.0 in background removal to remove the noise oscillation below 1 Å in *R*-space

S_0^2 value (0.744) was calculated from the fitting of metal foil with standard metal structure

Supplementary Table 10 | Structural parameters for samples prepared by IWI. Structural parameters extracted from the Ru K-edge EXAFS fitting results. CN: coordination number, *R*: interatomic distance, and σ^2: Debye-Waller factor. The goodness of fit is represented by the R-factor. (weighting: k^2)

Sample	Shell	CN	*R* (Å)	σ^2 (10^{-3} Å^2)	R-factor
Ru	Ru-Ru	6*	2.65 ± 0.01	2.56	0.007
	Ru-Ru	6*	2.70 ± 0.01		
RuO_2	Ru-O	2*	1.94 ± 0.01	0.73	0.014
	Ru-O	4*	1.98 ± 0.01		
aRu5/WC_x	Ru-C	3.54 ± 0.34	2.11 ± 0.01	7.68	0.012
	Ru-Ru	2.29 ± 0.31	2.75 ± 0.01	9.99	
	Ru-W	1.90 ± 0.55	2.84 ± 0.01		
Ru_p/WC_x_H_2	Ru-O	1.79 ± 0.25	1.97 ± 0.01	2.50	0.007
	Ru-Cl	0.57 ± 0.18	2.31 ± 0.01	6.41	
	Ru-Ru	5.25 ± 0.35	2.66 ± 0.00		
Ru/WC_x_H_2	Ru-O	1.04 ± 0.37	1.97 ± 0.01	5.93	0.010
	Ru-C	3.52 ± 0.32	2.09 ± 0.01	6.54	
	Ru-Ru	5.35 ± 0.43	2.67 ± 0.01		
Ru_p/WC_x_Ar	Ru-O	1.13 ± 0.40	1.99 ± 0.03	9.50	0.016
	Ru-Ru	3.08 ± 0.24	2.70 ± 0.01	2.83	
	Ru-W	2.52 ± 0.39	2.72 ± 0.01		
Ru/WC_x_Ar	Ru-O	1.56 ± 0.34	1.98 ± 0.02	4.35	0.015
	Ru-Ru	4.15 ± 0.35	2.69 ± 0.01	4.57	
	Ru-W	2.50 ± 0.56	2.71 ± 0.01		

Structural parameters extracted from the EXAFS fitting of reference metal foil. CN is the coordination number; *R* is the interatomic distance; σ^2 is the Debye-Waller factor (a measure of the static and thermal disorder in absorber-scatterer distance); ΔE_0 is edge energy shift (the zero kinetic energy difference between experiment and theoretical model); R-factor value is related with the goodness of the fitting.
* Value was assigned in curving fitting based on standard structure
R_{bkg} was set to 1.0 in background removal to remove the noise oscillation below 1 Å in *R*-space
S_0^2 value (0.744) was calculated from the fitting of metal foil with standard metal structure

Supplementary Table 11 | Structural parameters for particulate Ru catalysts. Structural parameters extracted from the Ru K-edge EXAFS fitting results. CN: coordination number, *R*: interatomic distance, and σ^2: Debye-Waller factor. The goodness of fit is represented by the R-factor. (weighting: k^2)

Sample	Shell	CN	*R* (Å)	σ^2 (10^{-3} Å^2)	R-factor
Ru	Ru-Ru	6*	2.65 ± 0.01	2.56	0.007
	Ru-Ru	6*	2.70 ± 0.01		
RuO_2	Ru-O	2*	1.94 ± 0.01	0.73	0.014
	Ru-O	4*	1.98 ± 0.01		
Ru/WC_x_R	Ru-O	2.06 ± 0.33	1.97 ± 0.01	5.69	0.012
	Ru-Ru	4.51 ± 0.41	2.66 ± 0.01	6.93	
Ru/C_R	Ru-O	2.79 ± 0.37	1.97 ± 0.01	6.95	0.014
	Ru-Ru	3.70 ± 0.41	2.67 ± 0.01	6.45	

Structural parameters extracted from the EXAFS fitting of reference metal foil. CN is the coordination number; *R* is the interatomic distance; σ^2 is the Debye-Waller factor (a measure of the static and thermal disorder in absorber-scatterer distance); ΔE_0 is edge energy shift (the zero kinetic energy difference between experiment and theoretical model); R-factor value is related with the goodness of the fitting.
* Value was assigned in curving fitting based on standard structure
** For Pt and Ir, EXAFS fitting was conducted at the L_2-edge to avoid spectral overlap between the L_3-edge and the W L_2-edge. S_0^2 value was obtained from reference foil measurements at the corresponding L_2-edge
R_{bkg} was set to 1.0 in background removal to remove the noise oscillation below 1 Å in *R*-space
S_0^2 value (0.744) was calculated from the fitting of metal foil with standard metal structure

Supplementary Table 12 | Comparison of EXAFS fitting results using Ru-C and Ru-O first shell models for aRu3/WC_x. Structural parameters extracted from the Ru K-edge EXAFS fitting results. The experimentally observed first-shell coordination in aRu3/WC_x was fitted using either a Ru-C or Ru-O scattering path to evaluate the origin of the first shell contribution. CN: coordination number, *R*: interatomic distance, and ^{2}: Debye-Waller factor. The goodness of fit is represented by the R-factor. (weighting: k^2)

Sample	Shell	CN	*R* (Å)	σ^2 (10^{-3} Å^2)	R-factor
Ru	Ru-Ru	6*	2.65 ± 0.01	2.56	0.007
	Ru-Ru	6*	2.70 ± 0.01		
RuO_2	Ru-O	2*	1.94 ± 0.01	0.18	0.019
	Ru-O	4*	1.99 ± 0.01		
	Ru-Ru	2*	3.17 ± 0.01	3.30	
	Ru-O	4*	3.31 ± 0.05	0.18	
	Ru-Ru	8*	3.57 ± 0.01	3.30	
aRu3/WC_x	Ru-C	4.01 ± 0.17	2.11 ± 0.00	6.18	0.003
	Ru-Ru	1.72 ± 0.14	2.76 ± 0.01		
	Ru-W	2.28 ± 0.28	2.86 ± 0.01	8.78	
aRu3/WC_x (Ru-O fitting)	Ru-O	2.41 ± 0.93	2.02 ± 0.02	9.28	0.045
	Ru-Ru	1.96 ± 1.03	2.75 ± 0.03		
	Ru-W	2.70 ± 1.38	2.85 ± 0.03	10.11	

Structural parameters extracted from the EXAFS fitting of reference metal foil. CN is the coordination number; *R* is the interatomic distance; σ^2 is the Debye-Waller factor (a measure of the static and thermal disorder in absorber-scatterer distance); ΔE_0 is edge energy shift (the zero kinetic energy difference between experiment and theoretical model); R-factor value is related with the goodness of the fitting.
* Value was assigned in curving fitting based on standard structure

R_{bkg} was set to 1.0 in background removal to remove the noise oscillation below 1 Å in R-space
S_0^2 value (0.744) was calculated from the fitting of metal foil with standard metal structure

Supplementary Table 13 | Comparison of Ru-W coordination parameters obtained from EXAFS fitting and theoretical calculations. The shaded rows correspond to Ru-W alloy references, while the unshaded entries represent Ru ALNs structures.

Experimental parameters via EXAFS fitting

Sample	Coordination number of Ru-W	Bond length of Ru-W
aRu1/WC_x	2.86	2.84
aRu2/WC_x	2.71	2.84
aRu3/WC_x	2.28	2.86
aRu4/WC_x	2.12	2.82
aRu5/WC_x	1.90	2.84
$aRu5_p/WC_x$	1.99	2.72
Ru/WC_x_Ar	2.50	2.71
Ru_p/WC_x_Ar	2.52	2.72

Theoretical parameters

Sample	Coordination number of Ru-W (< 2.75 Å)	Coordination number of Ru-W (< 2.90 Å)
Ru_1/WC	1.295	2.642
Ru_2/WC	1.500	2.257
Ru_3/WC	1.643	2.250
Ru_4/WC	1.381	2.444
Ru_5/WC	1.465	2.285
Ru_6/WC	1.365	2.318
Ru_{13}/WC	1.661	2.158
Ru_{55}/WC	1.792	2.042
Ru_{116}/WC	1.793	2.042
Ru_{163}/WC	1.815	2.028
Ru_{200}/WC	1.799	2.028
Ru_{235}/WC	3.036	3.694

Supplementary Table 14 | Lattice parameters for theoretical calculations. Lattice parameters and the k-point meshes for the cells used in DFT calculations.

System	Lattice parameter	K-point meshes
Pt	a, b = 11.305 Å, c = 26.923 Å, α, β = 90°, γ = 120°	Γ (3 × 3 × 1)
Graphene	a, b = 9.917 Å, c = 24.271 Å, α, β, γ = 90°	
Ru	a, b = 10.973 Å, c = 26.497 Å, α, β = 90°, γ = 60°	
δ-WC	a = 10.144 Å, b = 11.713 Å, c = 24.279 Å, α, β, γ = 90°	
α-W_2C	a, b = 12.327 Å, c = 27.083 Å, α, β = 90°, γ = 120°	
γ-WC	a, b = 17.567 Å, c = 26.587 Å, α, β, γ = 90°	Γ (2 × 2 × 1)

Supplementary Table 15 | Variation of ΔG_H according to the geometric structure of noble metal. ΔG_H for Pt (111), Ru (0001), single-atom Ru on γ-WC (Ru_1/WC), Ru dimers on γ-WC (Ru_2/WC), and Ru_N/WC (N: 1 – 6, the number of Ru).

Catalysts	ΔG_{*H} (eV)
Pt	-0.1477
Ru	-0.2692
Ru_1/WC	0.3197
Ru_2/WC	-0.2761
Ru_3/WC	-0.2388
Ru_4/WC	-0.2108
Ru_5/WC	-0.3185
Ru_6/WC	-0.3091

Supplementary Table 16 | The binding energies of oxygenated species on various supports. The Gibbs free energy of $*H_2O$ and (*H + *OH) on Pt (111), Ru (0001), graphene, γ-WC, δ-WC, and α-W_2C.

Supports	ΔG_{*H2O} (eV)	ΔG_{*H+*OH} (eV)
Pt	-0.1540	0.2619
Ru	-0.2926	-0.3597
Carbon	-0.1094	2.8891
γ-WC	-0.5523	-0.4290
δ-WC	-0.6629	-2.2982
α-W_2C	-1.0280	-1.4547

Supplementary Table 17 | Electrochemical performance metrics for alkaline HER. Electrochemical responses (overpotential at -10 mA cm^{-2} (η_{10}), Tafel slope, and current density at an overpotential of 25 and 50 mV per noble metal mass ($j_{25/mass}$ & $j_{50/mass}$) for HER in H_2-saturated 1 M KOH solution. All potentials are referenced to the reversible hydrogen electrode (RHE).

Catalysts	Pt/C	aRu1/WC_x	aRu2/WC_x	aRu3/WC_x	aRu4/WC_x	aRu5/WC_x	aRu5_p/WC_x
η_{10} (mV)	42	68	39	22	30	37	84
Tafel slope (mV dec^{-1})	81.9	79.9	66.2	41.9	48.4	59.5	84.7
$j_{25/mass}$ (mA mg_{mass}^{-1})	-98.240	-149.022	-135.900	-300.484	-122.817	-72.215	-29.484
$j_{50/mass}$ (mA mg_{mass}^{-1})	-312.030	-526.769	-575.941	-1202.043	-559.029	-292.150	-82.807

Catalysts	Ru/WC_x_R	Ru/C_R	aRu0/WC_x
η_{10} (mV)	40	54	185
Tafel slope (mV dec^{-1})	78.1	80.2	95.7
$j_{25/mass}$ (mA mg_{mass}^{-1})	-102.049	-75.673	-
$j_{50/mass}$ (mA mg_{mass}^{-1})	-349.835	-209.239	-

Supplementary Table 18 | Charge transfer resistance. Charge transfer resistance (R_{ct}) calculated from EIS measurement at -40 mV (vs. RHE) in H_2-saturated 1 M KOH solution. All potentials are referenced to the reversible hydrogen electrode (RHE).

Catalysts	Pt/C	aRu1/WC_x	aRu2/WC_x	aRu3/WC_x	aRu4/WC_x	aRu5/WC_x
R_{ct} (Ω cm^{-2}) at 40 mV	17.41	29.85	14.56	5.708	9.782	13.19

Catalysts	aRu5$_p$/WC_x	Ru/WC_x_R	Ru/C_R
R_{ct} (Ω cm^{-2}) at 40 mV	77.70	20.24	21.31

Supplementary Table 19 | Structural parameters extracted from EXAFS fitting analyses. Structural parameters extracted from the Ru K-edge, Pt L_2-edge, Ir L_2-edge EXAFS fitting results. CN: coordination number, *R*: interatomic distance, and σ^2: Debye-Waller factor. The goodness of fit is represented by the R-factor. (weighting: k^2)

Sample	Shell	CN	*R* (Å)	σ^2 (10^{-3} Å^2)	R-factor
Ru	Ru-Ru	6*	2.65 ± 0.01	2.56	0.007
	Ru-Ru	6*	2.70 ± 0.01		
RuO_2	Ru-O	2*	1.94 ± 0.01	0.73	0.014
	Ru-O	4*	1.98 ± 0.01		
Ru/WC_x_R	Ru-O	2.06 ± 0.33	1.97 ± 0.01	5.69	0.012
	Ru-Ru	4.51 ± 0.41	2.66 ± 0.01	6.93	
Ru/C_R	Ru-O	2.79 ± 0.37	1.97 ± 0.01	6.95	0.014
	Ru-Ru	3.70 ± 0.41	2.67 ± 0.01	6.45	
RuNP/WC_x	Ru-O	2.93 ± 0.19	1.94 ± 0.01	5.87	0.003
	Ru-Cl	0.86 ± 0.14	2.30 ± 0.01		
	Ru-Ru	2.27 ± 0.23	2.66 ± 0.01	5.74	
RuNP/C	Ru-O	4.63 ± 0.19	1.97 ± 0.00	17.23	0.001
	Ru-Cl	1.45 ± 0.14	2.27 ± 0.01		
	Ru-Ru	2.35 ± 0.11	2.66 ± 0.00	5.00	
aRu/NC	Ru-N	1.53 ± 0.06	2.01 ± 0.00	1.75	0.001
	Ru-Cl	1.06 ± 0.03	2.33 ± 0.00		
	Ru-Ru	1.92 ± 0.08	2.61 ± 0.00	8.14	
aPt3/WC_x**	Pt-C	1.59 ± 0.30	2.08 ± 0.02	2.16	0.009
	Pt-Pt	4.10 ± 0.34	2.75 ± 0.01	2.07	
	Pt-W	1.93 ± 0.37	2.88 ± 0.01		
aIr3/WC_x**	Ir-C	2.46 ± 0.08	2.11 ± 0.00	2.69	0.001
	Ir-Ir	1.87 ± 0.09	2.73 ± 0.00	1.49	
	Ir-W	1.06 ± 0.09	2.88 ± 0.01		

Structural parameters extracted from the EXAFS fitting of reference metal foil. CN is the coordination number; *R* is the interatomic distance; σ^2 is the Debye-Waller factor (a measure of the static and thermal disorder in absorber-scatterer distance); ΔE_0 is edge energy shift (the zero kinetic energy difference between experiment and theoretical model); R-factor value is related with the goodness of the fitting.
* Value was assigned in curving fitting based on standard structure
** For Pt and Ir, EXAFS fitting was conducted at the L_2-edge to avoid spectral overlap between the L_3-edge and the W L_2-edge. S_0^2 value was obtained from reference foil measurements at the corresponding L_2-edge
R_{bkg} was set to 1.0 in background removal to remove the noise oscillation below 1 Å in *R*-space
S_0^2 value (0.744) was calculated from the fitting of metal foil with standard metal structure

Supplementary Table 20 | AEMWE performance metrics for various samples. Current density, mass activity, and specific activity of commercial Pt/C, aRu3/WC_x, aRu5/WC_x, and $aRu5_p/WC_x$ at 2.0 V_{cell}, measured at 60 or 80 ℃.

Catalysts at 2.0 V_{cell} (mg_{mass} cm^{-2})	Pt/C (0.4)	aRu3 /WC_x (0.03)	aRu3 /WC_x (0.05)	aRu3 /WC_x (0.07)	aRu3 /WC_x (0.09)	aRu3 /WC_x (0.10)	aRu3 /WC_x (0.125)	aRu5 /WC_x (0.10)	$aRu5_p$ /WC_x (0.10)
Current density 80 ℃, (A cm^{-2})	9.77	5.07	6.7	8.38	9.13	9.82	10.07	7.95	6.99
Current density per noble metal mass 80 ℃, (A mg_{mass}^{-1})	24.43	169.00	134.00	119.71	101.44	98.20	80.56	79.50	69.90
Current density per noble metal price 80 ℃, (A EUR^{-1})	784.1	12353.8	9795.3	8751.0	7415.5	7178.4	5888.9	5811.4	5109.6
Current density 60 ℃, (A cm^{-2})	7.63	4.31	5.27	6.34	6.91	7.58	7.74	5.94	5.16
Current density per noble metal mass 60 ℃, (A mg_{mass}^{-1})	19.08	143.67	105.40	90.57	76.78	75.80	61.92	59.40	51.60
Current density per noble metal price 60 ℃, (A EUR^{-1})	612.4	10502.0	7704.7	6620.7	5612.4	5540.9	4526.3	4342.1	3771.9

Supplementary Table 21 | Comparison of AEMWE performance. Comparison of AEMWE performance metrics reported AEMWE electrocatalysts with their corresponding references.

Membrane electrode assembly (MEA)			Current density		Specific activity @ 1.8 V_{cell}		Cell voltage (V_{cell})	Reference
Cathode (mg_{mass} cm^{-2})	Membrane	Anode (mg_{mass} cm^{-2})	@ 1.8 V_{cell} (A cm^{-2})	@ 2.0 V_{cell} (A cm^{-2})	per noble metal mass (A mg^{-1})	per noble metal price (A EUR^{-1})	@ 2 A cm^{-2}	
aRu3/WC_x/ CP (0.1)	HQPC-TMA-2.4	NiFe alloy/ Ni paper	4.52	9.82	45.17	3301.9	1.668	This work
aRu3/WC_x/ /CP (0.03)	HQPC-TMA-2.4	NiFe alloy/ Ni paper	2.07	5.07	68.90	5036.5	1.793	
Pt./C/ MPL-CP (0.16)	FAA-3-50	NiFeOOH/ SUS GDL	2.52	4.51	15.75	505.6	1.743	ACS Catal., 12, 135-145 (2022)
Pt/C/ CP (1)	FAA-3-50	$CoSb_2O_6$/ Ni foam	0.53	-	0.53	17.0	-	ACS Energy Lett., 6, 364-370 (2021)
Pt/C/ CC (1)	QPC-TMA	eCCO/ Ni foam	2.85	-	2.85	91.4	1.729	ACS Energy Lett., 7, 8, 2576-2583 (2022)
PtRu/C (0.6)	Branched PAP membrane	NiFeOOH/ Ni foam	3.52	6.25	5.86	231.4	1.677	ACS Energy Lett., 8, 10, 4043-4051 (2023)
Pt/C/ CP (0.4)	Sustainion X37-50	Ni_3Fe_1/ Ni paper	1.06	2.22	2.64	84.8	1.967	ACS Energy Lett., 8, 11, 4666-4673 (2023)
PtRu/C (0.4)	PiperION	IrO_2/Platinized Ti PTL (2.0)	3.19	5.75	1.33	9.8	1.707	ACS Energy Lett., 8, 12, 5240-5247 (2023)
Pt-Ni/C/ CP (0.7)	-	$Fe_{10}Ni_{45}Co_{45}$/ Ni foam	0.37	0.75	0.53	17.0	-	Adv. Energy Mater., 14, 2303350 (2024)
Ru/P-MoB/ NiFe foam (0.11)	Sustainion X37-50	RuO_2/ NiFe foam (0.760)	1.22	-	1.40	102.5	-	Adv. Energy Mater., 14, 2303384 (2024)
Pt/C/ CC (1)	PFPBPF-4-QA	$Co(OH)_2$/ Ni foam	1.86	3.9	1.86	59.8	1.814	Adv. Energy Mater., 2401725 (2024)
c-Ru_2P/Ir_2P HNT (0.25)	PiperION	NiFe/ Ni foam	5.99	12.23	23.96	244.5	1.631	Adv. Energy Mater., 14, 2401426 (2024)
c-PRP DWNT/C (0.25)	PiperION	IrO_2 (2)	4.65	9.40	2.07	14.3	1.660	Adv. Energy Mater., 14, 2304269 (2024)
Pt/C/ CP (0.6)	DURAION	Ni_3Fe LDH/ Ni fiber PTL	1.10	2.07	1.83	58.9	1.990	Adv. Funct. Mater., 32, 2203520 (2022)
MnRu/C60-3/ Ni foam (0.6006)	Versogen	NiFe-LDH	1.03	-	1.71	125.1	-	Adv. Funct. Mater., 2409406 (2024)
PtRu/C/ CP (1,5)	Sustainion X37-50	HEO-NS/SS	1.45	2.53	0.97	38.1	1.902	Adv. Funct. Mater., 34, 2309438 (2024)
PtRu/C/ SS (0.6)	-	FeNi nanoplate/C/ SS	2.26	-	3.77	148.7	-	Adv. Funct. Mater., 2496175 (2024)
Ru/Zn-N-C/ Ni foam (0.00639)	FAA-3-PK-130	NiFe-LDH/ Ni foam	0.09	0.42	14.08	1029.6	-	Adv. Mater., 36, 2308798 (2024)
Pt/C/ CP (0.5)	An-Be	FeCo/ Ni foam	1.97	3.56	3.94	126.5	1.830	Adv. Mater., 36, 2404981 (2024)
PtRu/C/ MPL-CP (0.42)	b-PDTP-Trip-5 AEM	IrO_2/ Ni paper (1.715)	8.60	16.00	4.03	30.3	1.596	Angew. Chem. Int. Ed., 63, e202316697 (2024)
Pt/C/ Ti felt (0.8)	Sustainion X37-50	Nei-Ir_1/CoGaOOH/ Ti felt (0.244)	0.46	0.81	0.44	16.3	2.700	Angew. Chem. Int. Ed., e202404418 (2024)

Membrane electrode assembly (MEA)			Current density		Specific activity @ 1.8 V_{cell}		Cell voltage (V_{cell})	Reference
Cathode (mg_{mass} cm^{-2})	Membrane	Anode (mg_{mass} cm^{-2})	@ 1.8 V_{cell} (A cm^{-2})	@ 2.0 V_{cell} (A cm^{-2})	per noble metal mass (A mg^{-1})	per noble metal price (A EUR^{-1})	@ 2 A cm^{-2}	
aRu3/WC_x/ CP (0.1)	HQPC-TMA-2.4	NiFe alloy/ Ni paper	4.52	9.82	45.17	3301.9	1.668	This work
aRu3/WC_x/ /CP (0.03)	HQPC-TMA-2.4	NiFe alloy/ Ni paper	2.07	5.07	68.90	5036.5	1.793	
Pt/C/ CC (0.4)	Sustainion X37-50	CuCo-oxide/ Ni foam	1.36	-	3.40	109.1	1.875	Appl. Catal. B-Environ., 278, 119276 (2020)
Pt/C/ MPL-CC (1)	Sustainion X37-50	$CoFeCe_{0.5}$/ Ni foam	2.15	-	2.15	69.0	1.783	Appl. Catal. B-Environ., 318, 121824 (2022)
Pt/C/ MPL-CC (1)	Sustainion X37-50	$Ni_{0.5}Co_{0.5}MoO_4$-nH_2O/ Ni foam	0.95	1.85	0.95	30.5	2.034	Appl. Catal. B-Environ., 328, 122504 (2023)
aPt/pNi-NOMC/CC (0.5)	Sustainion X37-50	Co_3O_4/ Ni foam	0.62	-	1.24	39.8	-	Appl. Catal. B-Environ., 343, 123568 (2024)
PtRu/C/ CP (0.9)	Xion Pention-72-15CL	Co@MXene/ PtTi PTL	2.11	5.57	2.34	92.6	1.800	Appl. Catal. B-Environ., 346, 123731 (2024)
Pt/C/ CP (0.6)	PBPA AEM	IrO_2/ Ni foam (0.857)	2.00	4.00	1.37	12.9	1.800	Chem. Eng. J., 467, 143442 (2023)
Pt/C (0.4)	QPC-TMA	IrO_2 (2)	2.55	4.45	1.06	7.7	1.738	Energy Environ. Sci., 13, 3633-3645 (2020)
Pt/C/ MPL-CP (0.233)	PFTP-13	IrO_2/ Ti felt (1.714)	3.89	7.68	2.00	13.9	1.679	Energy Environ. Sci., 14, 6338-6348 (2021)
Pt/C/ Ni foam (0.6)	Sustainion X37-50	NiCoFe-NDA/ Ni foam	0.33	0.54	0.54	17.4	-	Energy Environ. Sci., 14, 6546-6553 (2021)
Pt/C/ Ni foam (1)	Sustainion X37-50	La_2NiO_4@Ni-Fe/ Ni foam	0.45	0.80	0.45	14.4	-	Energy Environ. Sci., 16, 3331-3338 (2023)
Pt-$MoAl_{1-x}B$/C/ CC (0.027)	Sustainion X37-50	RuO2/ SS paper (3.8)	0.38	1	0.10	7.2	2.356	Energy Environ. Sci., 16, 4093-4104 (2023)
PtRu/C/ Ni paper (0.3)	PDTP	FeNiCo LDH/HPCC/ SS paper	4.07	10.29	13.57	535.7	1.708	Energy Environ. Sci., 16, 5019-5028 (2023)
NA-RuNi/C CP (0.01344)	Sustainion X37-50	NA-Ru_3Ni/C/ Ni foam (0.01344)	0.47	0.9	17.49	1278.2	2.430	Energy Environ. Sci., 16, 285-294 (2023)
Ru_1-Mo_2C/ CP (0.013)	Sustainion X37-50	NiFe LDH/ Ni foam	0.88	1.82	67.69	4948.3	2.030	Energy Environ. Sci., 17, 1397-1406 (2024)
Pt/C (1)	QAPPT	NiCo@FeNi LDH	3.38	5.60	3.38	108.6	1.674	Energy Environ. Sci., 17, 3396-3408 (2024)
PtRu/C/ CP (0.5)	HQPC-TMA_2.4	NiFe alloy/ Ni paper	7.29	14.60	14.58	578.3	1.613	Energy Environ. Sci., 17, 5399-5409 (2024)
Pt-AC/Cr-N-C/ CP (0.05)	Sustainion X37-50	NiFe LDH/ Ni foam	0.65	1.32	13.00	417.3	2.177	J. Am. Chem. Soc., 145, 21432-21441 (2023)
Ru SAs/WC_x (0.0252)	Sustainion X37-50	$NiFeOH_x$/ NF	1.06	1.85	42.18	3083.5	2.033	J. Am. Chem. Soc., 146, 7, 4883-4891 (2024)
Ir-Ni/Mo_5N_6/ CP (0.1)	Sustainion X37-50	Ir-Ni/Mo_5N_6/ CP (0.1)	1.10	2.10	5.50	34.6	1.980	J. Colloid Interf. Sci., 628, 306-314 (2022)
Pt/C/ MPL-CC (1)	Sustainion X37-50	Cu-Co oxide/ Ni foam	1.30	-	1.30	41.7	-	J. Mater. Chem. A, 8, 4290-4299 (2020)

Membrane electrode assembly (MEA)			Current density		Specific activity @ 1.8 V_{cell}		Cell voltage (V_{cell})	Reference
Cathode (mg_{mass} cm^{-2})	Membrane	Anode (mg_{mass} cm^{-2})	@ 1.8 V_{cell} (A cm^{-2})	@ 2.0 V_{cell} (A cm^{-2})	per noble metal mass (A mg^{-1})	per noble metal price (A EUR^{-1})	@ 2 A cm^{-2}	
aRu3/WC_x/ CP (0.1)	HQPC-TMA-2.4	NiFe alloy/ Ni paper	4.52	9.82	45.17	3301.9	1.668	This work
aRu3/WC_x/ /CP (0.03)	HQPC-TMA-2.4	NiFe alloy/ Ni paper	2.07	5.07	68.90	5036.5	1.793	
PtRu/C/ MPL-CP (1.5)	Sustainion X37-50	W/FeNiOOH/C/ SUS felt	4.84	7.87	3.23	103.6	1.615	Joule, 7, 1902-1919 (2023)
Pt/C/ CP (0.471)	Tokuyama A901	$CoFe_{0.25}Al_{1.75}O_4$/ KB/SSL mesh	0.63	-	1.34	42.9	-	Nat. Catal., 2, 763-772 (2019)
Pt/C/ CC (0.1)	AF2HLF8-25X	$Li_2Mn_{1-x}Ru_xO_3$/ Ti PTL (0.246)	0.75	1.59	2.17	115.7	2.118	Nat. Catal., 7, 546-559 (2024)
Pt_1/CoHPO/KB/ MPL-CP (0.0114)	Alkymer	Pt_1/CoHPO/KB/ Ti mesh (0.0171)	0.99	-	34.74	1115.1	1.930	Nat. Commun., 13, 3822 (2022)
Cl-Pt/NiFe-LDH/ Ni foam (0.0144)	Sustainion X37-50	NiFe-LDH/ Ni foam	0.74	1.51	51.39	1649.7	2.103	Nat. Commun., 13, 6875 (2022)
PtRu/C/ CP (0.6)	MTCP-50	NiFe/ Ni foam	5.40	9.21	9.00	355.4	1.583	Nat. Commun., 14, 2732 (2023)
Pt-Ru/RuO_2/ CC (0.5)	AEMION	NiFe-LDH/ Ni felt	0.49	1.61	0.98	70.4	2.074	Nat. Commun., 15, 1447 (2024)
Ru-Ga SA/N-C/ CP (0.080)	Sustainion X37-50	RuO_2/ Ni foam (0.760)	1.20	-	1.43	104.4	-	Nat. Commun., 15, 6741 (2024)
Pt/C/ AEM (1.5)	PiperION-A60-HCO3	$CoCrO_x$/ Pt-Ti felt	0.43	1.08	0.29	9.2	-	Nat. Commun., 15, 3416 (2024)
Ru/Ti_4O_7 (0.4196)	PiperION-A60-HCO3	Ru/$T_{i4}O_7$ (0.4196)	0.44	1.28	0.52	38.1	2.066	Nat. Commun., 15, 2728 (2024)
UP-RuNi SAs/ Carbon felt (1.1925)		UP-RuNi SAs/ Carbon felt (1.1925)	0.92	1.98	0.38	28.0	2.010	Nat. Commun., 15, 2218 (2024)
PtRu/C/ MPL-CP (3)	HTMA-DAPP	NiFe/platinized Ti GDL	5.12	-	1.71	67.4	1.639	Nat. Energy, 5, 378-385 (2020)
Ru SS/FeNiPiPHS$_x$/CP (0.081)	Sustainion X37-50	Ru SS/FeNiPiPHSx/ CP (0.081)	2.09	-	12.90	943.1	1.780	Nat. Synth., 3, 576-585 (2024)

Supplementary Table 22 | Dissolved metal concentrations after intermittent AEMWE operation. ICP-MS analysis of dissolved metal species in the catholyte collected after intermittent start-stop cycling tests. N/D, not detected; detection limit, 5 μg kg^{-1}

Element	aRu3/WC_x	commercial Pt/C
W	31.1 μg kg^{-1}	N/D
Pt	N/D	9.6 μg kg^{-1}
Ru	N/D	N/D

Supplementary References

1 Kim, S. et al. Self-assembly-assisted dynamic placement of noble metals selectively on multifunctional carbide supports for alkaline hydrogen electrocatalysis. *Energy Environ. Sci.* **18**, 659-673 (2025).

2 Kang, Y. et al. Mesoporous multimetallic nanospheres with exposed highly entropic alloy sites. *Nat. Commun.* **14**, 4182 (2023).

3 Zhang, J.-Y. et al. Mesoporous ruthenium titanium oxide solid solution with efficient three phase reaction interface for water electrolysis. *Nat. Commun.* **17**, 3752 (2026).

4 Gayrard, M. et al. Block-Copolymers Enable Direct Reduction and Structuration of Noble Metal-Based Films. *Small* **18**, 2104204 (2022).

5 Shim, J. et al. One-pot synthesis of intermetallic electrocatalysts in ordered, large-pore mesoporous carbon/silica toward formic acid oxidation. *ACS Nano* **6**, 6870-6881 (2012).

6 Hwang, J. et al. One-pot synthesis of tin-embedded carbon/silica nanocomposites for anode materials in lithium-ion batteries. *ACS Nano* **7**, 1036-1044 (2013).

7 Hwang, J. et al. Direct confinement of Ru nanoparticles inside nanochannels of large pore mesoporous aluminosilicate for Fischer–Tropsch synthesis. *J. Mater. Chem. A* **3**, 23725-23731 (2015).

8 Lee, J. et al. Direct access to thermally stable and highly crystalline mesoporous transition-metal oxides with uniform pores. *Nat. Mater.* **7**, 222-228 (2008).

9 Mun, Y. et al. Direct access to aggregation-free and small intermetallic nanoparticles in ordered, large-pore mesoporous carbon for an electrocatalyst. *RSC Adv.* **6**, 88255-88264 (2016).

10 Jung, K. T., Kim, W. B., Rhee, C. H. & Lee, J. S. Effects of transition metal addition on the solid-state transformation of molybdenum trioxide to molybdenum carbides. *Chem. Mater.* **16**, 307-314 (2004).

11 Kim, K.-W. et al. Small-angle X-ray scattering beamline BL4C SAXS at Pohang light source II. *Biodesign* **5**, 24-29 (2017).

12 Maliki, S. K., Kim, J. H., Chung, M. & Jin, K. S. Structural characterization of urea-induced BSA denaturation using size-exclusion chromatography coupled with small-angle X-ray scattering. *J. Phys. Chem. B* **130**, 171-178 (2025).

13 Lee, B. et al. In-situ grazing incidence small-angle X-ray scattering studies on nanopore evolution in low-k organosilicate dielectric thin films. *Macromol.* **38**, 3395-3405 (2005).

14 Yamashita, S. et al. Atomic number dependence of *Z* contrast in scanning transmission electron microscopy. *Sci. Rep.* **8**, 12325 (2018).

15 Tieu, P., Yan, X., Xu, M., Christopher, P. & Pan, X. Directly probing the local coordination, charge state, and stability of single atom catalysts by advanced electron microscopy: A review. *Small* **17**, 2006482 (2021).

16 Xu, M., Li, A., Gao, M. & Zhou, W. Single-atom electron microscopy for energy-related nanomaterials. *J. Mater. Chem. A* **8**, 16142-16165 (2020).

17 Ohyama, J., Sato, T., Yamamoto, Y., Arai, S. & Satsuma, A. Size specifically high activity of Ru nanoparticles for hydrogen oxidation reaction in alkaline electrolyte. *J. Am. Chem. Soc.* **135**, 8016-8021 (2013).

18 Wu, G. et al. A general synthesis approach for amorphous noble metal nanosheets. *Nat. Commun.* **10**, 4855 (2019).

19 Zhao, H. et al. Fine-tuning surface oxidation states of ruthenium nanoparticles to enhance hydrogen electrode reactions. *J. Mater. Chem. A* **11**, 18262-18271 (2023).

20 Duivenvoorden, F., Koningsberger, D., Uh, Y. & Gates, B. Structures of alumina-supported osmium clusters ($HOs_3(CO)_{10}${OA1}) and complexes ($Os^{II}(CO)_n${OA1}$_3$)(n= 2 or 3) determined by extended X-ray absorption fine structure spectroscopy. *J. Am. Chem. Soc.* **108**, 6254-6262 (1986).

21 Koningsberger, D., Martens, J., Prins, R., Short, D. & Sayers, D. The structure of a rhodium/titania catalyst in the strong metal-support interaction state as determined by EXAFS. *J. Phys. Chem.* **90**, 3047-3050 (1986).

22 Funke, H., Scheinost, A. & Chukalina, M. Wavelet analysis of extended X-ray absorption fine structure data. *Phys. Rev. B* **71**, 094110 (2005).

23 Jin, H., Chen, J., Mao, S. & Wang, Y. Transition metal induced the contraction of tungsten carbide lattice as superior hydrogen evolution reaction catalyst. *ACS Appl. Mater. Interfaces* **10**, 22094-22101 (2018).

24 Wang, L. et al. Tuning d-band center of tungsten carbide via Mo doping for efficient hydrogen evolution and Zn–H_2O cell over a wide pH range. *Nano Energy* **74**, 104850 (2020).

25 Hunt, S. T. et al. Self-assembly of noble metal monolayers on transition metal carbide nanoparticle catalysts. *Science* **352**, 974-978 (2016).

26 Ono, S., Kikegawa, T. & Ohishi, Y. A high-pressure and high-temperature synthesis of platinum carbide. *Solid State Commun.* **133**, 55-59 (2005).

27 Cao, L. et al. Identification of single-atom active sites in carbon-based cobalt catalysts during electrocatalytic hydrogen evolution. *Nat. Catal.* **2**, 134-141 (2018).

28 Hong, Y. et al. Double-walled tubular Heusler-type platinum-ruthenium phosphide as all-pH hydrogen evolution reaction catalyst outperforming platinum and ruthenium. *Adv. Energy Mater.* **14**, 2304269 (2024).

29 Kong, T.-H. et al. A Cathode Is the Key Contributor to the Initial Degradation of Anion Exchange Membrane Water Electrolyzers. *ACS Energy Lett.* **10**, 3647-3654 (2025).

30 Lejaeghere, K., Van Speybroeck, V., Van Oost, G. & Cottenier, S. Error estimates for solid-state density-functional theory predictions: an overview by means of the ground-state elemental crystals. *Crit. Rev. Solid State Mater. Sci.* **39**, 1-24 (2014).
