# Supplementary material for: Stabilizing ruthenium atom layer neighbors through molecular and metal support interactions for water electrolyzers
Source: Nat Commun. 2026 Sep 22;17:11021. doi: 10.1038/s41467-026-78037-3 (PMC13687079; doi:10.1038/s41467-026-78037-3)
Supplement: Supplementary file 3 [file 41467_2026_78037_MOESM2_ESM.pdf]

Description of Additional Supplementary Files

File name: Supplementary Data 1
Description: Structural models used for DFT calculations and MD simulations. The ZIP file contains the relevant computational structure files in CIF or plain text-based formats.

File name: Supplementary Movie 1
Description: Three-nanosecond trajectory of (**a,b**) a single Ru atom and (**c,d**) two Ru atoms on γ-WC at 1,200 K.

File name: Supplementary Movie 2
Description: Three-nanosecond trajectory of (**a,b**) three and (**c,d**) four Ru atoms in Ru atom-layer neighbors on γ-WC at 1,200 K.

File name: Supplementary Movie 3
Description: Three-nanosecond trajectory of (**a,b**) five and (**c,d**) six Ru atoms in Ru atom-layer neighbors on γ-WC at 1,200 K.

File name: Supplementary Movie 4
Description: Five-nanosecond trajectory of Ru atom-layer neighbors containing (**a**) 13, (**b**) 55, (**c**) 116, and (**d**) 163 Ru atoms on γ-WC at 1,200 K.

File name: Supplementary Movie 5
Description: Five-nanosecond trajectory of Ru atom-layer neighbors containing (**a**) 200, (**b**) 235, (**c**) 4,729, and (**d**) 37,414 Ru atoms on γ-WC at 1,200 K.

File name: Supplementary Movie 6
Description: DFT simulation showing the transformation of a two-atom Ru cluster into a two-dimensional Ru atom-layer neighbor structure on γ-WC at 298 K.

File name: Supplementary Movie 7
Description: DFT simulation showing the transformation of a four-atom Ru cluster into a two-dimensional Ru atom-layer neighbor structure on γ-WC at 298 K.

File name: Supplementary Movie 8
Description: Beam-induced aggregation of Ru atomic clusters in aRu/NC during prolonged HAADF-STEM observation. Sequential HAADF-STEM images were acquired from the same region under continuous electron-beam irradiation.
